# Temporal-spatial Generation of Astrocytes in the Developing Diencephalon

**DOI:** 10.1007/s12264-023-01131-9

**Published:** 2023-10-16

**Authors:** Wentong Hong, Pifang Gong, Xinjie Pan, Zhonggan Ren, Yitong Liu, Guibo Qi, Jun-Liszt Li, Wenzhi Sun, Woo-Ping Ge, Chun-Li Zhang, Shumin Duan, Song Qin

**Affiliations:** 1 Department of Anatomy, Histology and Embryology, School of Basic Medical Sciences, Fudan University, Shanghai, 200032 China; 2 Academy for Advanced Interdisciplinary Studies, Peking University, Beijing, 100871 China; 3 Chinese Institute for Brain Research, Beijing, 102206 China; 4 School of Basic Medical Sciences, Capital Medical University, Beijing, 100069 China; 5 Department of Molecular Biology, University of Texas Southwestern Medical Center, Dallas, Texas 75390-9148 USA; 6 Department of Neurobiology, Key Laboratory of Medical Neurobiology of Ministry of Health of China, Zhejiang University School of Medicine, Hangzhou, 310058 China; 7 State Key Laboratory of Medical Neurobiology and MOE Frontiers Center for Brain Science, Fudan University, Shanghai, 200032 China

**Keywords:** Radial glia, Astrocyte specification, Lineage tracing, Diencephalon, Third ventricle

## Abstract

Astrocytes are the largest glial population in the mammalian brain. However, we have a minimal understanding of astrocyte development, especially fate specification in different regions of the brain. Through lineage tracing of the progenitors of the third ventricle (3V) wall *via in-utero* electroporation in the embryonic mouse brain, we show the fate specification and migration pattern of astrocytes derived from radial glia along the 3V wall. Unexpectedly, radial glia located in different regions along the 3V wall of the diencephalon produce distinct cell types: radial glia in the upper region produce astrocytes and those in the lower region produce neurons in the diencephalon. With genetic fate mapping analysis, we reveal that the first population of astrocytes appears along the zona incerta in the diencephalon. Astrogenesis occurs at an early time point in the dorsal region relative to that in the ventral region of the developing diencephalon. With transcriptomic analysis of the region-specific 3V wall and lateral ventricle (LV) wall, we identified cohorts of differentially-expressed genes in the dorsal 3V wall compared to the ventral 3V wall and LV wall that may regulate astrogenesis in the dorsal diencephalon. Together, these results demonstrate that the generation of astrocytes shows a spatiotemporal pattern in the developing mouse diencephalon.

**Supplementary Information:**

The online version contains supplementary material available at 10.1007/s12264-023-01131-9.

## Introduction

Astrocytes, which are widely distributed throughout the mammalian nervous system (CNS), are also its most abundant cell type. The developmental genesis and dysfunction of astrocytes are closely associated with neurological disorders such as Rett syndrome and fragile X mental retardation [1, 2]. Although numerous lines of evidence have shown that astrocytes play pivotal roles in both normal and pathological conditions [3–5], an understanding of the development of the astrocyte lineage is still in its infancy.

The generation of astrocytes in the ventral spinal cord is modulated in a regionally restricted manner [6]. For example, the cross-repressive interactions between the basic helix-loop-helix transcription factors SCL and OLIG2 direct astrogenesis in the p2 progenitor domain of the spinal cord [7], and the combinatorial expression of homeodomain proteins PAX6 and NKX6.1 specifies the region-specific subtypes of ventral astrocytes [8]. The radial glia-derived astrocytes are allocated to spatial domains in both the spinal cord and brain of mice in accordance with their embryonic sites of origin in the ventricular zone (VZ) [4]. Consistent with this, it has been reported that cortical protoplasmic astrocytes are generated in a spatially restricted manner from radial glia which also gives rise to developmental columns of pyramidal neurons [9].

During CNS development, radial glial cells consist of a periventricular cell body and an elongated radial process extending from the VZ to the pial surface. These morphologically distinct bipolar cells serve as neural precursor cells (NPCs) capable of producing neurons and then astrocytes [10]. At perinatal stages, individual radial glia transforms into astrocytes in the cortex after neurogenesis is completed [11, 12]. To date, the stages in the development of the astrocyte lineage, including the specification and migration process, have been poorly defined.

In the current study, we explored the fate specification and migration of astrocytes derived from region-specific radial glia located in the third ventricle (3V) wall during the development of the diencephalon. Furthermore, we conducted genetic fate mapping analysis of astrocyte progression using astrocyte-specific *hGFAP-CreER*^*T2*^*; Ai14* transgenic mice. These findings suggest that astrocytes are generated with a spatiotemporal pattern in the developing diencephalon.

## Materials and Methods

### Animals

The following transgenic mice were used: *hGFAP-CreER*^*T2*^ (The Jackson Laboratory, stock 012849) [13] and Ai14 (Rosa-tdTomato; The Jackson Laboratory, stock 007914) [14]. Wild-type C57BL/6J and ICR mice were purchased from the Shanghai SLAC Laboratory. To obtain the exact embryonic time-point, one male and two female mice were placed together in one cage in the evening, and the female’s vulva was checked the next morning. E0.5 was logged for the first day of pregnancy if a vaginal plug was observed. All mice were housed under a 12-h light/dark cycle and had *ad libitum* access to food and water in a controlled animal facility. All animals were treated in accordance with protocols approved by the Animal Care and Use Committee of Shanghai Medical College of Fudan University.

### Tamoxifen Treatments

For the induction of CreER-dependent recombination in *hGFAP-CreER*^*T2*^*;Ai14* transgenic mice, an intraperitoneal injection of tamoxifen (T5648, Sigma; dissolved in a 1:9 mixture of ethanol/sesame oil) at 75 mg/kg body weight was administered to pregnant females in a single dose at various time points as indicated, and pups were perfusion-fixed at P14. Tamoxifen was given by intraperitoneal injection at a daily dose of 100 mg/kg body weight to 2-month-old adult *hGFAP-CreER*^*T2*^*;Ai14* transgenic mice for 4 days, and the mice were perfusion-fixed.

### Plasmids and *In Utero* Electroporation

cDNA encoding EYFP was cloned into the vector pCAGGS, in which expression is driven by the CAG promoter (modified chicken β-actin promoter with enhanced sequences from CMV). The lentiviral plasmid hGFAP-GFP was generated by sub-cloning the synthetic hGFAP promoter [15] into the CS-CDF-CG-PRE vector at the EcoRI and AgeI sites. For *in-utero* electroporation (IUE), a simple laparotomy under anesthesia was performed on wild-type ICR pregnant female mice at 14.5 days of gestation. While the embryos were still in the uterus, 1.5 µl of plasmid DNA solution (4 mg/ml) mixed with Fast Green (2 mg/ml) was directly injected into the LV or 3V of the embryonic forebrain using a glass micropipette. Five electrical pulses at 35 V with a duration of 50 ms/pulse and 950-ms intervals were applied through the uterus using an electroporator (ECM 830, BTX). During this procedure, the uterus was kept wet with warm saline. After the electroporation, the uterus was carefully repositioned in the abdominal cavity. The cavity was filled with warm saline to replenish the abdominal fluids. The abdominal wall and skin were separately sutured. Pregnant mice recovered under a warm lamp and then were returned to their home cage. Injected embryos were allowed to develop normally and were analyzed 24 h, 36 h, or 72 h after electroporation or at P1, P7, P14, or P21.

### Virus Preparation and *In Utero* Injection

As described previously [16], lentivirus was produced in HEK293T cells after transient transfections. Briefly, the third-generation replication-deficient lentivirus was generated by transient transfections of HEK293T cells with hGFAP-EGFP lentiviral vector together with the packaging plasmids (pMDL, VSVG, and pREV). The virus-containing culture supernatants were collected and concentrated by centrifugation. Viral titers were estimated by quantifying GFP-expressing cells of virus-transduced U251 glioma cells at 72 h after viral infection. Similar to the IUE method, a simple laparotomy under anesthesia was performed on wild-type ICR pregnant female mice at 14.5 days of gestation. While the embryos were still in the uterus, 1.5 μL of lentivirus (0.5–1 × 10^9^ plaque-forming units/mL) was manually injected into the 3V of the embryonic forebrain using a glass micropipette.

### Immunohistochemistry and Imaging

Embryonic brains were extracted at the indicated ages and fixed with 4% paraformaldehyde. Postnatal brains were extracted and fixed in 4% paraformaldehyde after transcardial perfusion. Brains were further post-fixed overnight and then cryoprotected with 30% sucrose in PBS at 4°C. Coronal sections were cut at 16 µm on a Cryostat (Leica) and mounted onto Superfrost-plus microscope slides (Fisher). For immunostaining, sections were washed with PBS and blocked with 5% bovine serum albumin (BSA) in PBS. After overnight incubation with primary antibodies diluted in the blocking solution with gentle agitation at 4°C, sections were washed and incubated for 2 h with corresponding secondary species-specific antibodies conjugated with Alexa Fluor 488, 555, or 647 (Jackson ImmunoResearch). Nuclei were counterstained with Hoechst 33342. The following primary antibodies were used: GFP (chicken, 1:500; AVES), GFAP (mouse, 1:1000; Sigma-Aldrich), Aldh1L1 (mouse, 1:500; Millipore), PCNA (mouse, 1:400; Santa Cruz), S100β (rabbit, 1:1000; Swant), NeuN (mouse, 1:500; Chemicon), SOX2 (rabbit, 1:500; Chemicon), OLIG2 (rabbit, 1:500; Millipore), NG2 (rabbit, 1:500; BBI Life Sciences), GS (mouse,1:500; Millipore), IBA1 (Rabbit, 1:1000; Waco), CD31 (rat, 1:1000; BD Biosciences), NFIA (rabbit, 1:500; Genetax), AQP4 (mouse, 1:200; Santa Cruz), and Collagen IV (rabbit, 1:500; Millipore).

Fluorescent images were acquired on a Leica SP8 or Nikon A1 confocal laser microscope system. The confocal images were analyzed by NIH ImageJ software. A Cell Counter software plugin in the ImageJ program was used to count cells.

### RNA Sequencing (RNA-Seq) and Analysis

Total RNA was isolated from the wall of the 3V or LV of E14.5 mouse embryos using the RNAprep pure Micro Kit according to the manufacturer’s instructions (Tiangen Biotech, Beijing, China). The extracted RNA was quantified using the Qubit® RNA HS Assay Kit with a Qubit ® 2.0 Fluorometer (Life Technologies, Grand Island, NY, USA). The quality of the RNA was analyzed using an Agilent 2100 bioanalyzer (Agilent Technologies, Palo Alto, CA). The RNA-seq libraries for next-generation sequencing and paired-end deep sequencing were constructed on an Illumina PE150 platform (Illumina, San Diego, CA) according to the manufacturer’s protocol. Trimmomatic was recruited to low-quality reads and adaptor trimming with a default setting [17] (http://www.usadellab. org/cms/?page=trimmomatic). Cleaned reads were mapped to the ensemble mouse reference genome GRCm38.p6 (http://asia.ensembl.org/Mus_musculus/ Info/Index) with Hisat2 [18]. The mapped reads were counted to genes using featureCounts (http://subread.sourceforge.net/). Principal component analysis (PCA) clustering plots, volcano plots, and heat maps were generated using the R programming language. Targeted gene set enrichment analysis (GSEA) was applied following previous protocols [19, 20]. A custom geneset that represents positive regulation of astrogenesis and astrocyte development was generated (Table S5). Genes were pre-ranked through the metrics algorithm (log fold change × −log10 (p-value [not adjusted p-val)] according to the statistical result of DESeq2. Pre-ranked (.rnk) file and custom geneset were used as input for GSEA v4.0.3 (https://www.gsea-msigdb.org/gsea/index.jsp). The number of permutations was set at 1000 and the enrichment statistic was set at “weighted”.

### Experimental Design and Statistical Analysis

Data are presented as the mean ± standard error of the mean (SEM). Statistical analysis was done and graphs were produced using GraphPad Prism 8.0 software (La Jolla, CA, USA). Data from two groups were analyzed with a two-tailed unpaired Student’s *t*-test. Data from multiple groups were analyzed with one-way ANOVA followed by Turkey’s multiple comparison *post hoc* test. A level of *P* <0.05 was considered statistically significant. For RNA-Seq analysis, differential expression analysis was applied using DESeq2 (https://bioconductor.org/packages/release/bioc/html/DESeq2.html) with a cutoff of FDR <0.01 and abs (log_2_FC) >1. For GSEA analysis, FDR q-val <0.25 and |NES| >1 were considered as significant enrichment.

## Results

### Astrocytes Derived from Region-specific Radial Glia in the 3V Wall

**Fig. 1:**
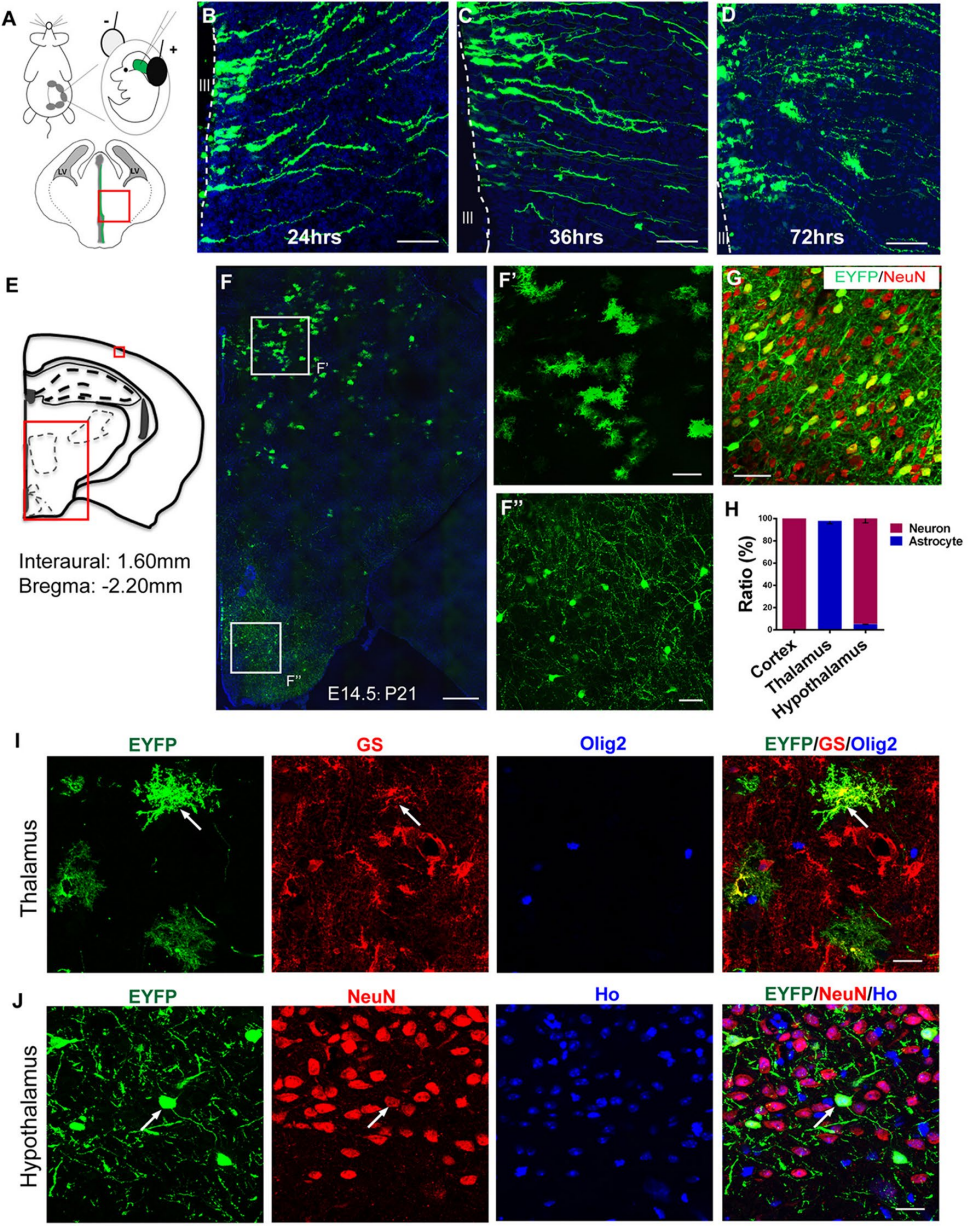
Astrocyte fate specification in the developing diencephalon. **A** Schematic showing the procedure of IUE (upper) and the brain region of the embryonic diencephalon analyzed in B, C, and D (lower). **B−D** The embryonic diencephalon at 24 h (**B**), 36 h (**C**), and 72 h (**D**) after IUE with CAG-EYFP plasmid at E14.5. Scale bars, 50 µm. **E** Schematic depicting the brain region analyzed in F and G. **F** EYFP-expressing radial glia differentiate into astrocyte-like cells in the thalamus (**F’**) and into neuron-like cells in the hypothalamus (**F’’**) at P21. Scale bars: F, 200 µm; F’, F’’, 50 µm. **G** Immunostaining of sections from IUE at P21 with NeuN, a neuronal marker, in the cortex at P21. Scale bar, 100 µm.** H** Quantification of the cellular fate of EYFP-expressing cells in the cortex, thalamus, and hypothalamus, *n* = 3 mice. **I, J** Fate identification by staining with antibodies against GS, NeuN, or OLIG2 in the thalamus (**I**) and hypothalamus (**J**). Scale bars, 50 µm. Ho, Hoechst staining. Note: Sections with a blue signal are counterstained with Hoechst (**B-D, F**).

By injecting the 3V of E (embryonic day) 14−15 mouse embryos *in utero* with plasmids with specific genes (e.g., GFP) and subsequently delivering these plasmids into the progenitors lining the ventricular wall *via* IUE, these transfected progenitors were temporally traceable in the diencephalon [21, 22]. To investigate how radial glia give rise to astrocytes in the developing diencephalon, we used the enhanced yellow fluorescent protein (EYFP) reporter plasmid under the control of the constitutive CAG promoter (CAG-EYFP) to label 3V progenitors (Fig. 1 A). The labeled cells in the 3V wall showed radial glial morphology 24 h after electroporation (Figs. 1B and S1A). Numerous EYFP-expressing radial glia were observed to elongate their long radial processes to the pial surface throughout the entire diencephalon 36 h post-IUE (Figs. 1C and S1B). 72 h post-IUE, we observed some EYFP^+^ radial glia that had migrated out of the VZ/subventricular zone (SVZ) of the 3V and showed astrocytic morphology in the parenchyma of the dorsal diencephalon (Figs. 1D and S1C).

The peak of neurogenesis occurs at E14.5 in rodent neocortex [23]. After the NPCs were labeled with CAG-EYFP plasmid by electroporation in the wall of the 3V *versus* the lateral ventricle (LV), their fate specification was lineage-traced after birth. Consistently, NPCs located within the dorsal ventricular wall of the LV at E14.5 differentiated into pyramidal-like neurons that migrated to the superficial layer of the cerebral cortex by P7 (Fig. S2A). In sharp contrast, EYFP-labeled radial glia at E14.5 produced two distinct cell populations in the diencephalon at P7, astrocyte-like cells on the dorsal side and neuron-like cells on the ventral side (Fig. S2B).

The fate of the progeny of radial glia that underwent IUE at E14.5 was also examined at P21, ~4 weeks post-electroporation (Fig. 1E). Accordingly, we also observed two populations - astrocyte-like cells in the thalamus and neuronal-like cells in the hypothalamus - in the diencephalon at P21 (Fig. 1F). By immunostaining, we further confirmed that 100% of EYFP^+^ cells in the cerebral cortex were positive for NeuN, a marker for mature neurons, at P21 (Fig. 1G and H). In contrast, we found that 98% of EYFP^+^ cells in the thalamus were glutamine synthetase-positive (GS)^+^ astrocytes, and 95% of EYFP^+^ cells in the hypothalamus were NeuN^+^ neurons at P21 (Fig. 1H–J).

### Tracing Astrocyte Migration in the Developing Diencephalon

**Fig. 2:**
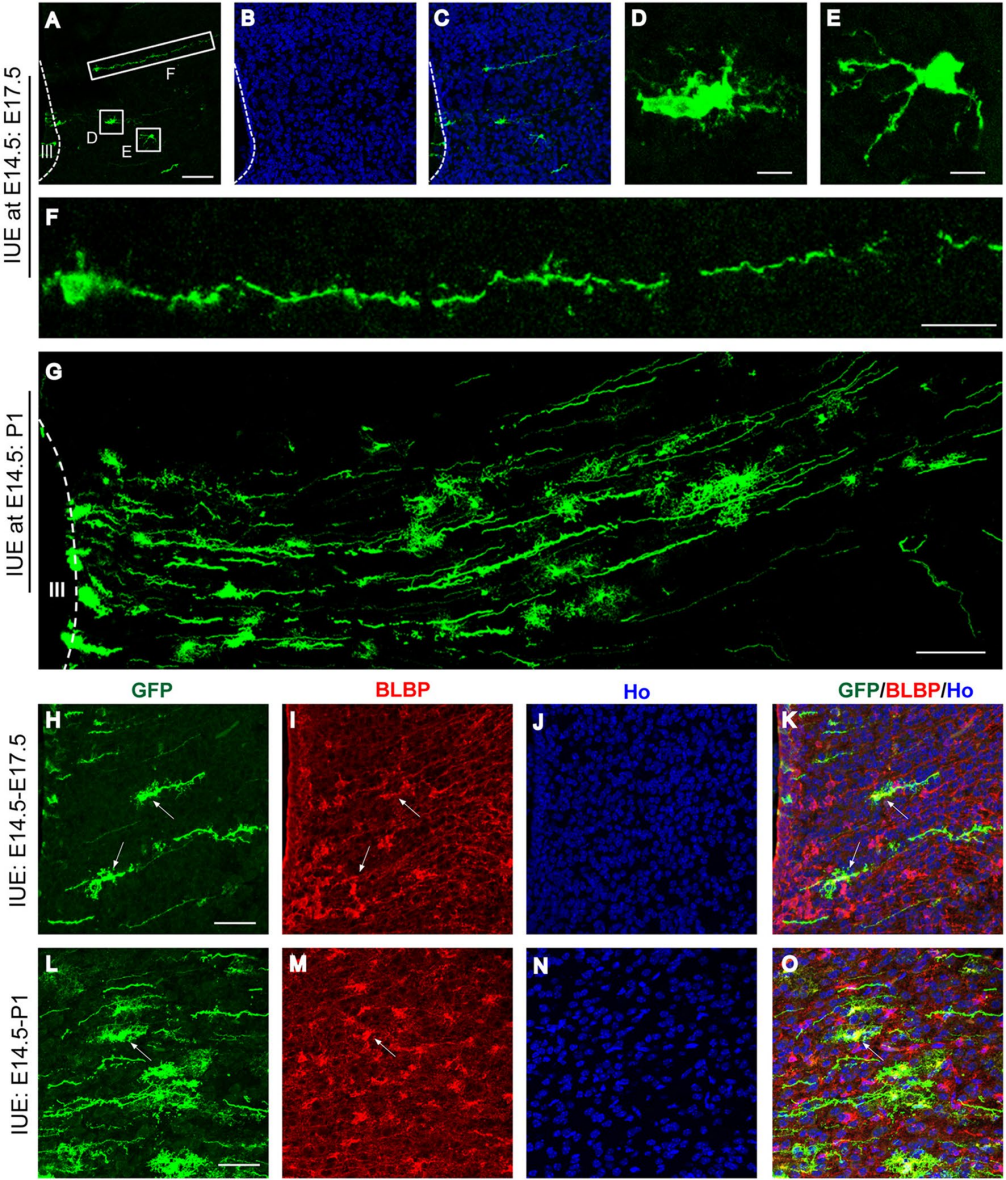
Lineage tracing of astrocyte migration in the developing diencephalon. **A-C** Embryonic mouse brains electroporated with hGFAP-EGFP plasmid at E14.5 and examined at E17.5. Radial migration of astrocyte progenitors in the dorsal diencephalon is shown. Hoechst staining reveals the nuclei (**B**). Scale bar, 200 µm. **D-F** Higher-magnification views of EGFP^+^ cells from the boxed regions show the different morphologies of the radial migrating astrocyte progenitors. Scale bars: **D, E,** 25 µm; **F**, 50 µm. **G** Brain electroporated with hGFAP-EGFP plasmid at E14.5 and examined at P1. Scale bar, 50 µm. **H-O** Immunostained sections from E17.5 (**H−K**) and P1 (**L−O**) brains from mice that underwent IUE at E14.5 show GFP-expressing cells co-labeled with BLBP in the diencephalon. Scale bars, 50 µm. Ho, Hoechst staining.

To understand the migration of astrocytes in the developing diencephalon, we next injected the enhanced green fluorescent protein (EGFP) reporter plasmid under the control of the human GFAP promoter (hGFAP-EGFP) and electroporated it into the progenitors lining the 3V wall of E14.5 mouse embryos. Similar to the results from the CAG-EYFP plasmid experiments, the hGFAP-EGFP−labeled cells in the 3V wall also showed radial glial morphology 24 h post-IUE (Fig. S3A). The radial migration of astrocyte progenitors with various morphologies in the dorsal diencephalon was observed when sections were examined at E17.5 (Figs 2A and S3B). The cell bodies of astrocyte progenitors were flat or round with multiple processes (Fig. 2D and E). Notably, some migrating astrocyte progenitors had quite long leading processes with spine-like protrusions (Fig. 2F). The radial migration pattern of astrocytes was found in the dorsal diencephalon when examined at P1 (Fig. 2G). In addition, the EGFP-expressing cells that migrated to the parenchyma of the diencephalon were stained with BLBP, an astrocyte marker [24, 25], at both E17.5 and P1 (Fig. 2H–O, arrows).

### Fate Specification of Migrating Astrocyte Progenitors in the Developing Diencephalon

**Fig. 3:**
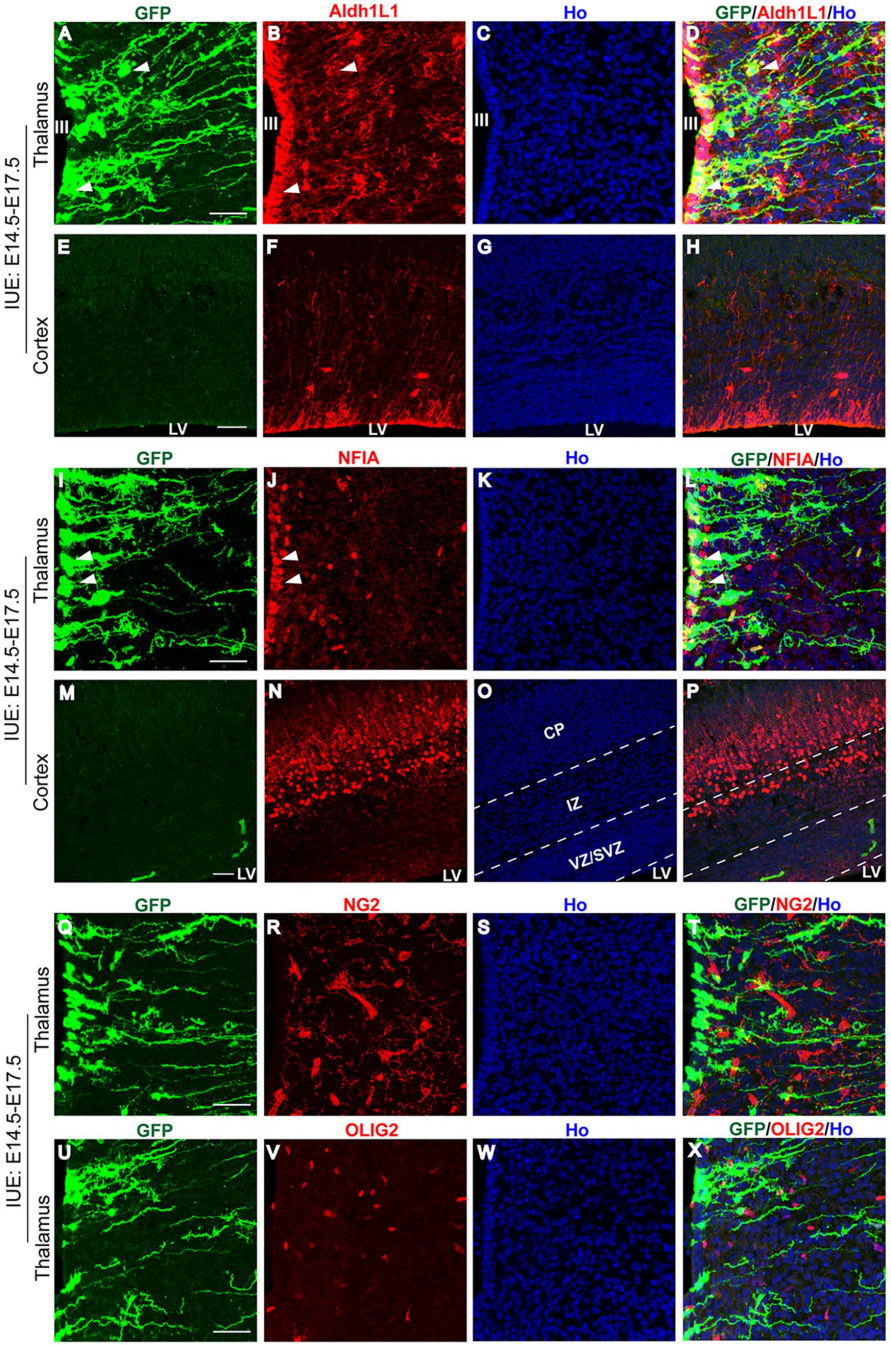
Early marker expression of migrating astrocyte progenitors in the developing diencephalon. **A-H** Sections from E17.5 mouse brains that were electroporated with hGFAP-EGFP plasmid at E14.5 are immunostained with an antibody against Aldh1L1 in the thalamus (**A-D**) and cortex (**E-H**). **I-P** Sections from E17.5 mouse brains that underwent IUE at E14.5 are immunostained with an antibody against NFIA in the thalamus (**I-L**) and cortex (**M-P**). III, third ventricle; LV, lateral ventricle; VZ/SVZ, ventricular zone/subventricular zone; IZ, intermediate zone; CP, cortical plate. **Q-X** Immunostaining with antibodies against NG2 or OLIG2 as indicated in the developing thalamus. Scale bars, 50 µm. Ho, Hoechst staining.

To further confirm the identity of the migrating progenitors labeled by hGFAP-EGFP, we stained the electroporated brains with an antibody for Aldh1L1, a broad astrocyte marker [26, 27]. The hGFAP-EGFP−labeled cells exhibited extensive colocalization with the staining of Aldh1L1 in the diencephalon at E17.5 (Fig. 3A–D). Compared with numerous Aldh1L1^+^ astrocyte progenitors in the diencephalon, very few cells were positive for Aldh1L1 in the cortex (Fig. 3E–H). These cortical Aldh1L1^+^ cells were mainly distributed in the ventricular wall of the LV and showed radial glial morphology (Fig. 3F).

We next stained electroporated brain sections with the antibody against nuclear factor I A (NFIA), a transcription factor known to regulate gliogenesis in the embryonic spinal cord [28] and developing cortex [29]. The hGFAP-EGFP-expressing cells were largely co-labelled with NFIA in the 3V wall at E17.5 (Fig. 3I–L). In contrast, weak expression of NIFA was detected in the wall of the LV but was highly expressed in the deep cortical layers and subplate (Fig. 3M–P). Furthermore, the EGFP-expressing cells were not stained positive for markers of NG2 glia (NG2, Fig. 3Q–T) or oligodendrocytes (OLIG2, Fig. 3U–X).

### Tracing Astrocyte Proliferation and Maturation in the Developing Diencephalon

**Fig. 4:**
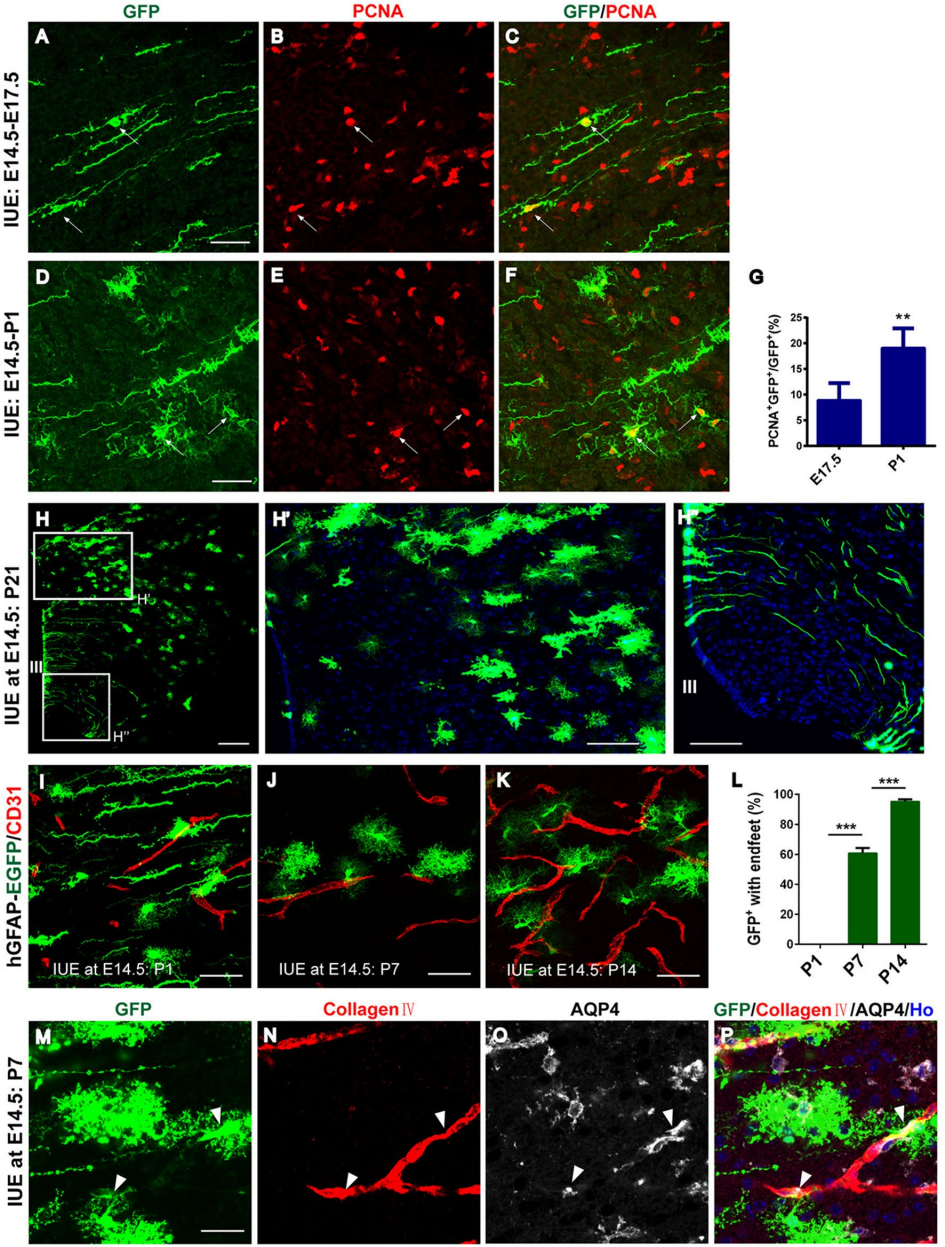
Lineage tracing of astrocyte proliferation and maturation in the developing diencephalon.** A−F** Immunostaining for PCNA of E17.5 (**A-C**) and P1 (**D-F**) brain sections from mice that underwent IUE with hGFAP-EGFP plasmid at E14.5. Scale bars, 50 µm. **G** Quantification of the ratio of astrocyte proliferation in the developing diencephalon, *n* = 3 independent animals. Error bars indicate SEM, **P <0.01. **H** E14.5 embryos were electroporated with hGFAP-EGFP plasmid and examined at P21. Scale bar, 200 µm. A higher-magnification view from **H** shows EGFP-expressing cells in the thalamus that exhibit astrocytic morphology (**H’**). Scale bar, 50 µm. A higher-magnification view from **H** indicates that EGFP-expressing cells in the hypothalamus are confined specifically to the wall of the 3V and have radial glial (tanycyte-like) morphology (**H’’**). Scale bar, 50 µm. **I-K** Sections from P1, P7, and P14 mouse brains that were electroporated with hGFAP-EGFP plasmid at E14.5 are immunostained with CD31 to label blood vessels. Scale bars, 50 µm. **L** Quantification of the ratio of endfeet enveloping blood vessels to the total numbers of GFP^+^ astrocytes at P1, P7, and P14 (*n* = 3 animals from independent pregnancies per time point). Error bars indicate SEM, *** P <0.001. **M-P** Immunostained sections from P7 mouse brains that underwent IUE at E14.5 show GFP-expressing astrocytes co-labeled with AQP4 and Collagen IV in the diencephalon. Scale bar, 25 µm. Note: Coronal sections with a blue signal are counterstained with Hoechst.

To characterize the proliferation profile of astrocytes in the early developing diencephalon, we stained brain sections at E17.5 and P1 for PCNA, a specific marker for proliferating cells, after electroporation at E14.5. As shown in Fig. 4A–D (arrows), 9% and 19% of EGFP^+^ cells were dividing cells (EGFP^+^PCNA^+^) at E17.5 and P1 (Fig. 4G), respectively. These data suggest that early migrating astrocytes undergo proliferation in the developing diencephalon.

The progeny of EGFP^+^ cells labeled in the 3V wall by IUE were examined at P21. All the EGFP-expressing cells in the dorsal part of the diencephalon showed astrocyte features with bushy morphology (Fig. 4H and H’). In contrast, the EGFP^+^ cells in the ventral diencephalon were confined to the wall of the 3V and had radial glial (tanycyte-like) morphology (Fig. 4H and H’’). These EGFP^+^ cells projected their long extended basal processes into the parenchyma, and some processes appeared to end at the pial surface of the hypothalamus (Fig. 4H’’).

Astrocytic endfeet is crucial for the structure of the blood-brain barrier and the regulation of cerebral blood flow [30, 31]. Endfeet of cortical astrocytes almost fully cover the blood vessels by P20 [25]. Staining with an antibody against CD31, a marker for endothelial cells [32], indicated that 61% and 95% of the EGFP^+^ astrocytes formed endfeet structures with blood vessels in the dorsal diencephalon at P7 and P14, respectively (Fig. 4J–L). By contrast, endfeet structures with blood vessels in EGFP^+^ astrocytes were rarely observed at P1 (Fig. 4K and L). In addition, staining with an antibody against AQP4, which is restrictively expressed on the perivascular endfeet in astrocytes [33], displayed co-localization with EGFP^+^ astrocytes and collagen IV^+^ blood vessels (Fig. 4M–P, arrows). These results indicate the temporal maturation of astrocytes in the postnatal diencephalon.

### Astrocyte Specification Traced Through a Lentivirus in the Developing Diencephalon

**Fig. 5:**
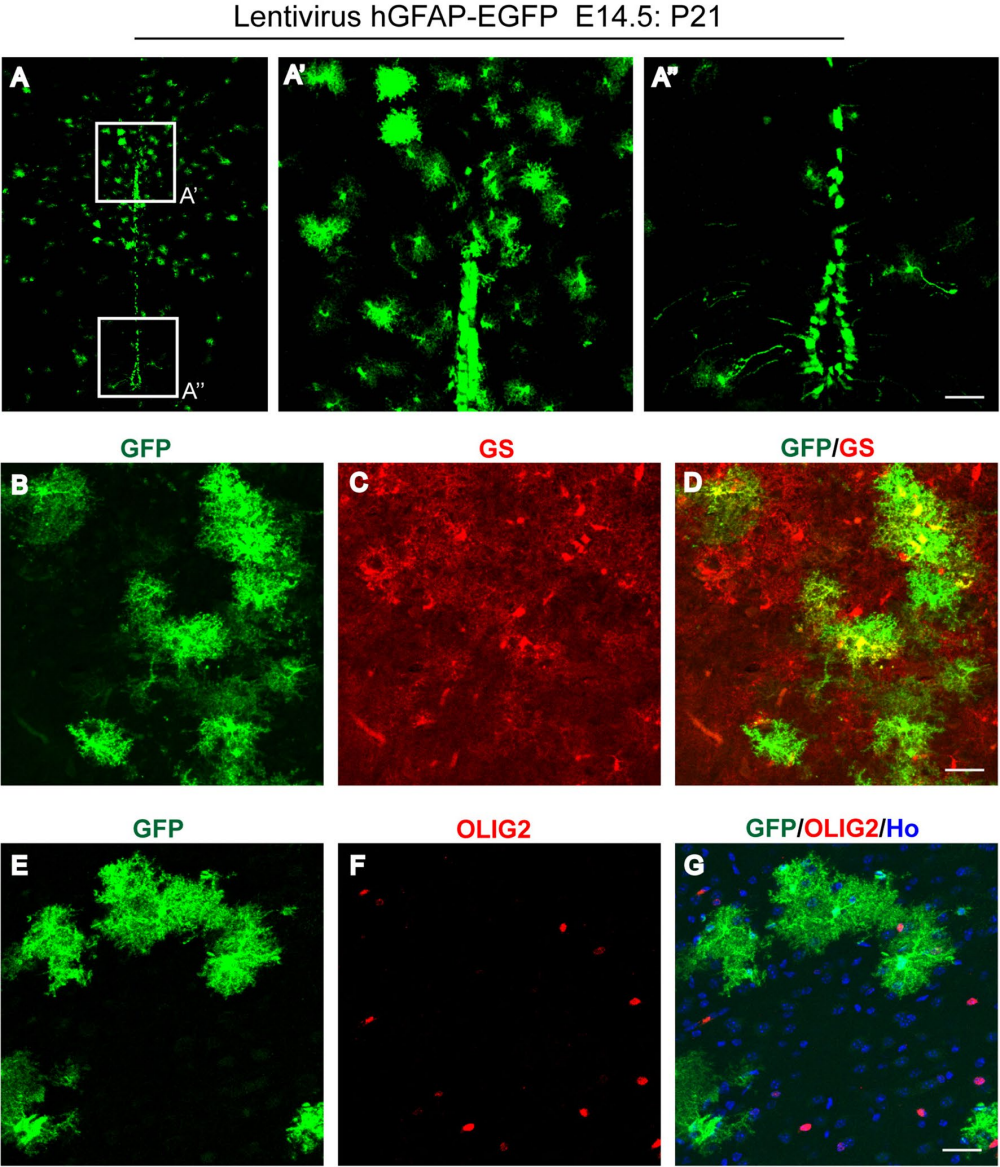
Astrocyte specification in the diencephalon as determined by hGFAP-EGFP lentiviral infection in the 3V at E14.5. **A** Brain sections from P21 mice that underwent lentiviral infection at E14.5 showed the GFP-expressing cells in the diencephalon. **A’, A’’** Higher-magnification views of EGFP+ cells in the boxed regions in the dorsal (**A’**) and ventral (**A’’**) diencephalon. Scale bars, 100 µm. **B–G** Immunostaining with the antibodies against GS or OLIG2 as indicated. Scale bars, 50 µm. Ho, Hoechst staining.

We next traced astrocyte specification by injecting the hGFAP-EGFP lentivirus into the 3V of E14.5 mouse embryos, since the IUE approach might be limited in its ability to detect the region-specific progenitors due to potentially insufficient levels of the episomal-based reporter plasmid. We found that the virus-infected EGFP^+^ cells at P21 were mainly distributed in the dorsal diencephalon with astrocytic morphology (Fig. 5A, A’ and A’’), similar to IUE-labeled cells in the diencephalon. In addition, the EGFP-expressing cells were GS^+^ and were not co-localized with the staining of OLIG2, a marker for the oligodendrocyte lineage (Fig. 5B–G).

### Astrocyte Lineage Development in the Diencephalon *Versus* the Telencephalon

The hGFAP promoter drives gene expression localized in the VZ of the developing embryonic telencephalon [34]. Crossing the inducible astrocyte-specific line *hGFAP-CreER*^*T2*^ with *Ai14* transgenic mice allowed robust expression of the red fluorescent protein tdTomato (tdT) after inducible astrocyte-specific Cre-mediated recombination [25]. We first checked the specificity of tamoxifen-induced tdT^+^ cells in the adult brain regions of *hGFAP-CreER*^*T2*^*;Ai14* transgenic mice. All tdT^+^ cells were S100β^+^ astrocytes in the cortex (Fig. S4A–D), thalamus (Fig. S4E–H), and hypothalamus (Fig. S4I–H). Furthermore, after 48 h of tamoxifen administration that began at E14.5, we found a few radial glia-like cells in the dorsal 3V wall (Fig. S5A and a1), whereas multiple tdT^+^ cells were present in the dorsal telencephalon (Fig. S5A and a2).

**Fig. 6:**
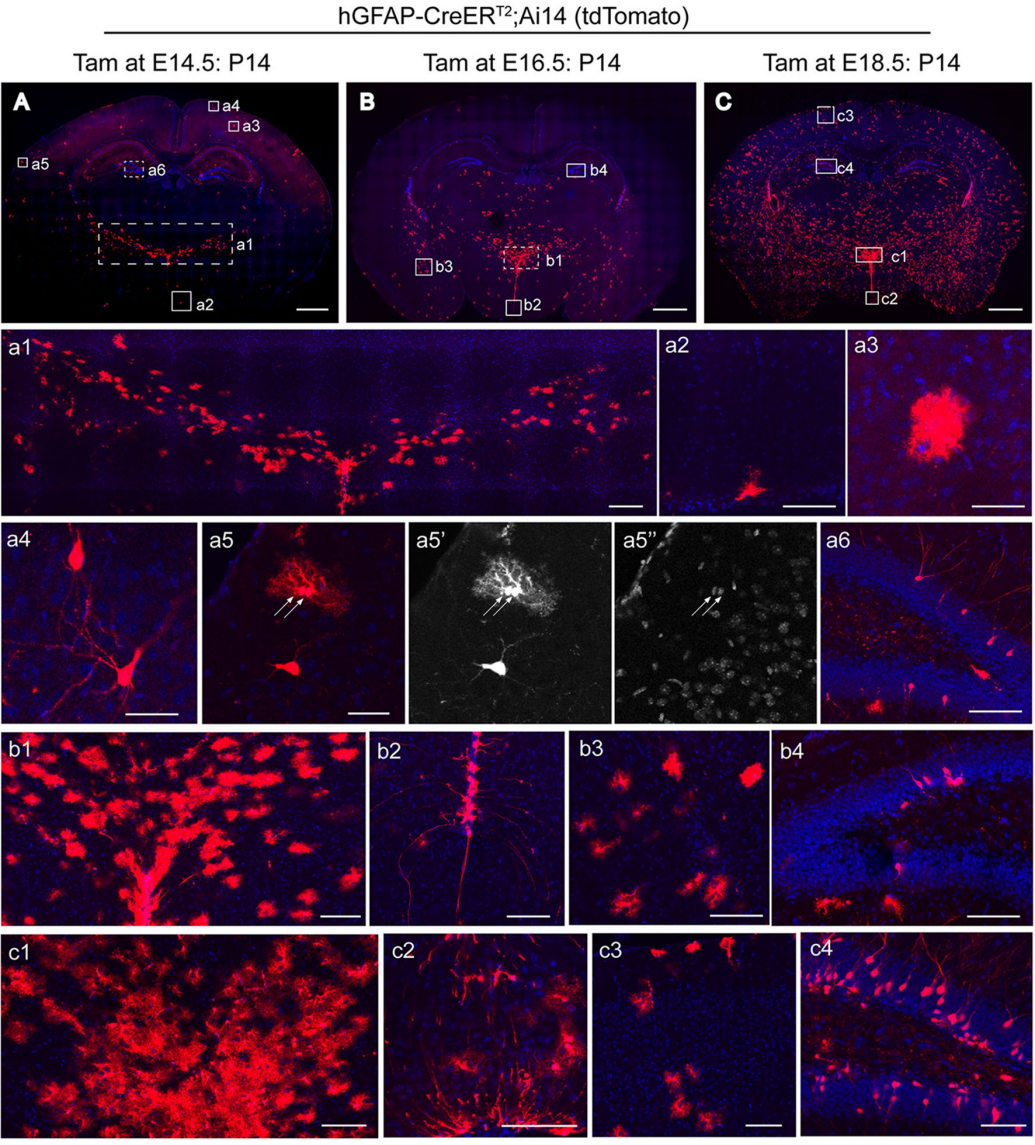
Astrocyte lineage progression in the forebrain at different developmental stages. **A−C** P14 *hGFAP-CreER*^*T2*^*; Ai14(tdT)* transgenic mice induced by tamoxifen at E14.5 (**A**), E16.5 (**B**), and E18.5 (**C**). Scale bars, 1 mm. **a1−a6** Higher-magnification views of tdT^+^ cells in the boxed regions induced at E14.5 (**A**) show the distribution of tdT^+^ astrocytes along the region of the zona incerta in the thalamus (**a1**), an astrocytes-like cell in the ventral region of the diencephalon (**a2**), a protoplasmic astrocyte (**a3**), pyramidal neurons (**a4**), dividing protoplasmic astrocytes (**a5**) in the cortex, and the distribution of tdT^+^ cells in the dentate gyrus (DG) (**a6**). Scale bars: **a1,** 200 µm; **a2, a6**, 100 µm; **a3-a5,** 50 µm. **b1−b4,** Higher-magnification views of tdT^+^ cells in the boxed regions induced at E16.5 (**B**) are shown in the thalamus (**b1**), hypothalamus (**b2**), amygdala (**b3**), and DG (**b4**). Scale bars, 100 µm. **c1−c4** Higher-magnification views of tdT^+^ cells in the boxed regions induced at E18.5 (**C**) are shown in the thalamus (**c1**), hypothalamus (**c2**), cortex (**c3**), and DG (**c4**). Scale bars, 100 µm. Note: Coronal sections with a blue signal are counterstained with Hoechst.

To determine the underlying pattern of temporal-spatial generation of astrocytes in the diencephalon *versus* the telencephalon, we performed a genetic fate mapping analysis of *hGFAP-CreER*^*T2*^*;Ai14* transgenic mice. We administered tamoxifen to timed-pregnant *hGFAP-CreER*^*T2*^*;Ai14* transgenic mice at E14.5, E16.5, and E18.5. In the P14 *hGFAP-CreER*^*T2*^*;Ai14* transgenic mice treated with tamoxifen at E14.5, we found that all tdT^+^ cells displayed astrocytic morphology in the diencephalon (Fig. 6A). Interestingly, these astrocyte-like cells were mostly distributed along the zona incerta in the dorsal region above the 3V (Fig. 6A and a1). In contrast, only a few scattered tdT^+^ astrocyte-like cells were observed in the ventral region of the diencephalon (Fig. 6A and a2). In the cerebral cortex, the tdT^+^ cells that were induced at E14.5 exhibited both pyramidal neuron-like and protoplasmic astrocyte-like cells (Fig. 6A, a3, and a4). Some tdT^+^ dividing protoplasmic astrocytes were found in cortical layers II/III (Fig. 6A and a5). In the hippocampal formation, most of the tdT^+^ cells displayed the morphology of pyramidal neurons in the hippocampal CA1/CA3 region or of granular neurons localized in the dentate gyrus (Fig. 6A and a6). Compared with the tdT^+^ cells induced at E14.5 in the diencephalon, there was a dramatic increase in tdT^+^ astrocyte-like cells, particularly in the dorsal region above the 3V, that was induced by tamoxifen at E16.5 (Fig. 6B and b1). In addition to scattered astrocytes in the ventral region of the diencephalon, we also observed tdT^+^ tanycytes lining the 3V wall with long processes (Fig. 6B and b2). The tdT^+^ cells induced at E18.5 showed a large population of astrocyte-like cells distributed across the entire forebrain (Fig. 6C). In the dentate gyrus, most of the tdT^+^ cells that were induced at E14.5, E16.5, or E18.5 showed a granular neuron morphology (Fig. 6a6, b4, and c4), suggesting that cells with an active hGFAP promoter continue to generate neurons during the development of the dentate gyrus.

### Temporal-spatial Generation of Astrocytes in the Developing Diencephalon

**Fig. 7:**
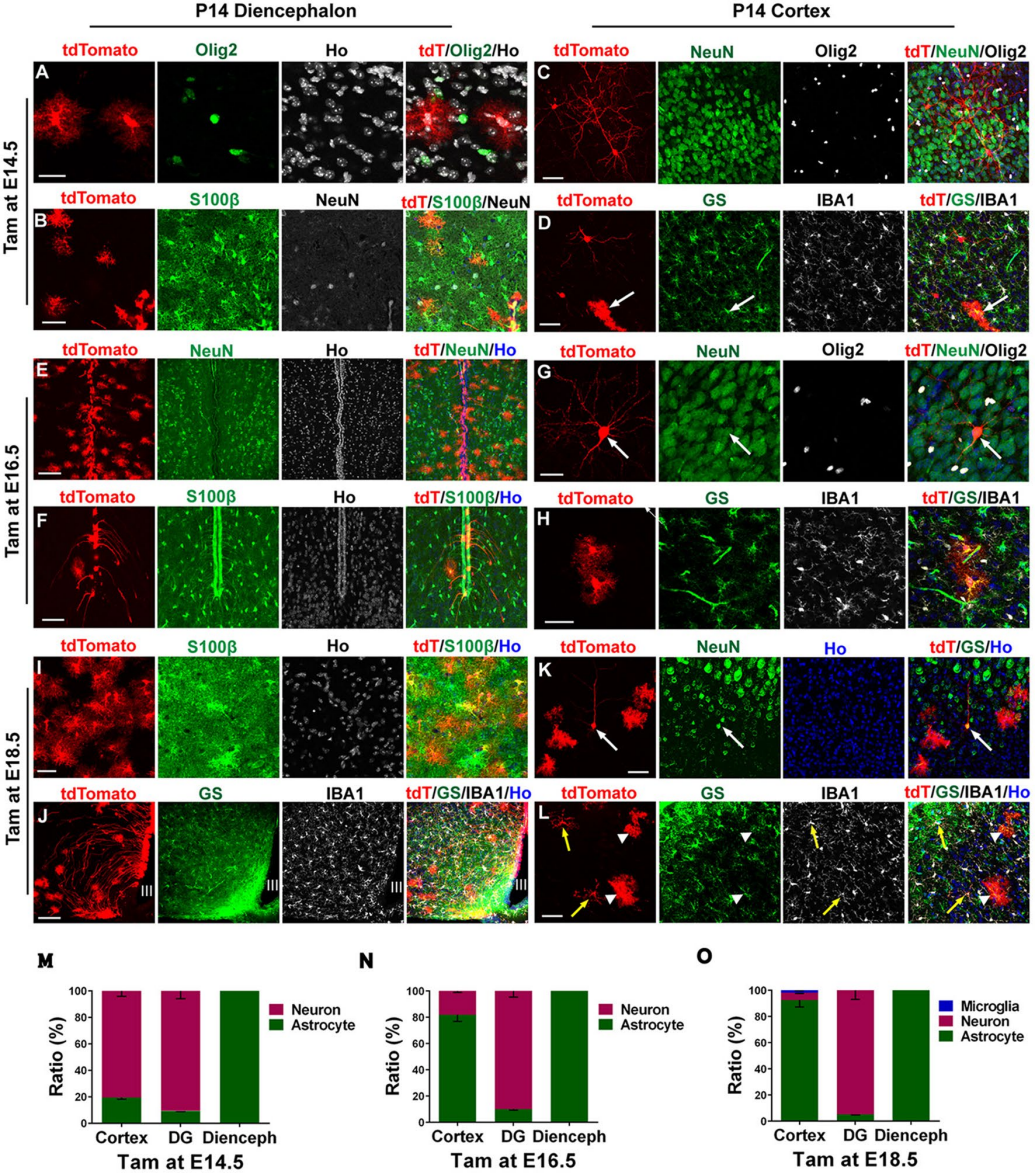
Cell fate identification in P14 *hGFAP-CreER*^*T2*^*; Ai14* transgenic mice induced by tamoxifen at different developmental stages. **A−D** Immunostaining with the indicated antibodies in the diencephalon (**A, B**) and cortex (**C, D**), with tdT-expressing cells induced by tamoxifen at E14.5. Scale bars, 50 µm. **E−H** Immunostaining with the indicated antibodies in the diencephalon (**E, F**) and cortex (**G, H**) with tdT-expressing cells induced by tamoxifen at E16.5. Scale bars: **E, F,** 50 µm; **G, H,** 100 µm. **I−L** Immunostaining with the indicated antibodies in the diencephalon (**I, J**) and cortex (**K, L**) with tdT-expressing cells induced by tamoxifen at E18.5. Scale bars: **I, K, L,** 50 µm; **J,** 100 µm. **M−O,** Quantification of the ratios of cell types in cortex, DG, and diencephalon of *hGFAP-CreER*^*T2*^*; Ai14* transgenic mice at E14.5 (**M**), E16.5 (**N**), and E18.5 (**O**); *n* = 3 independent animals per time point.

The cellular fates of tdT^+^ cells induced at timed developmental stages were assessed at P14. By immunostaining for cell type-specific markers, we found no cells in the diencephalon were NeuN^+^ or Olig2^+^ (Fig. 7A and B). In contrast, all tdT^+^ cells induced at E14.5, E16.5, or E18.5 were positive for S100β (Fig. 7B, F, and I). Although all tdT^+^ cells in the diencephalon were astrocytes, 80% of tdT^+^ cells induced at E14.5 were NeuN^+^ and only 20% of tdT^+^ cells were GS^+^ astrocytes in the cortex (Fig. 7C, D, and M). Notably, the ratio of tdT^+^ cells displaying co-localization with GS staining in the cerebral cortex was increased to 82.0% with induction at E16.5 and to 93% with induction at E18.5 (Fig. 7H, L, N, and O). Interestingly, in addition to the 6% of tdT^+^ cells that were NeuN^+^ in the cortex of mice induced at E18.5, we also found that 2% of tdT^+^ cells were IBA1^+^ microglia in the P14 cortex (Fig. 7K, L, and O).

The above analysis of tamoxifen-induced reporter expression at different developmental stages in *hGFAP-CreER*^*T2*^*;Ai14* transgenic mice indicates that astrocyte generation in the diencephalon precedes that in the telencephalon. The lineage tracing analysis also suggests that astrogenesis occurs at an early time point in the dorsal region relative to that in the ventral region of the developing diencephalon.

### Transcriptomic Profiling Reveals Spatial Generation of Astrocytes in the Developing Forebrain

**Fig. 8:**
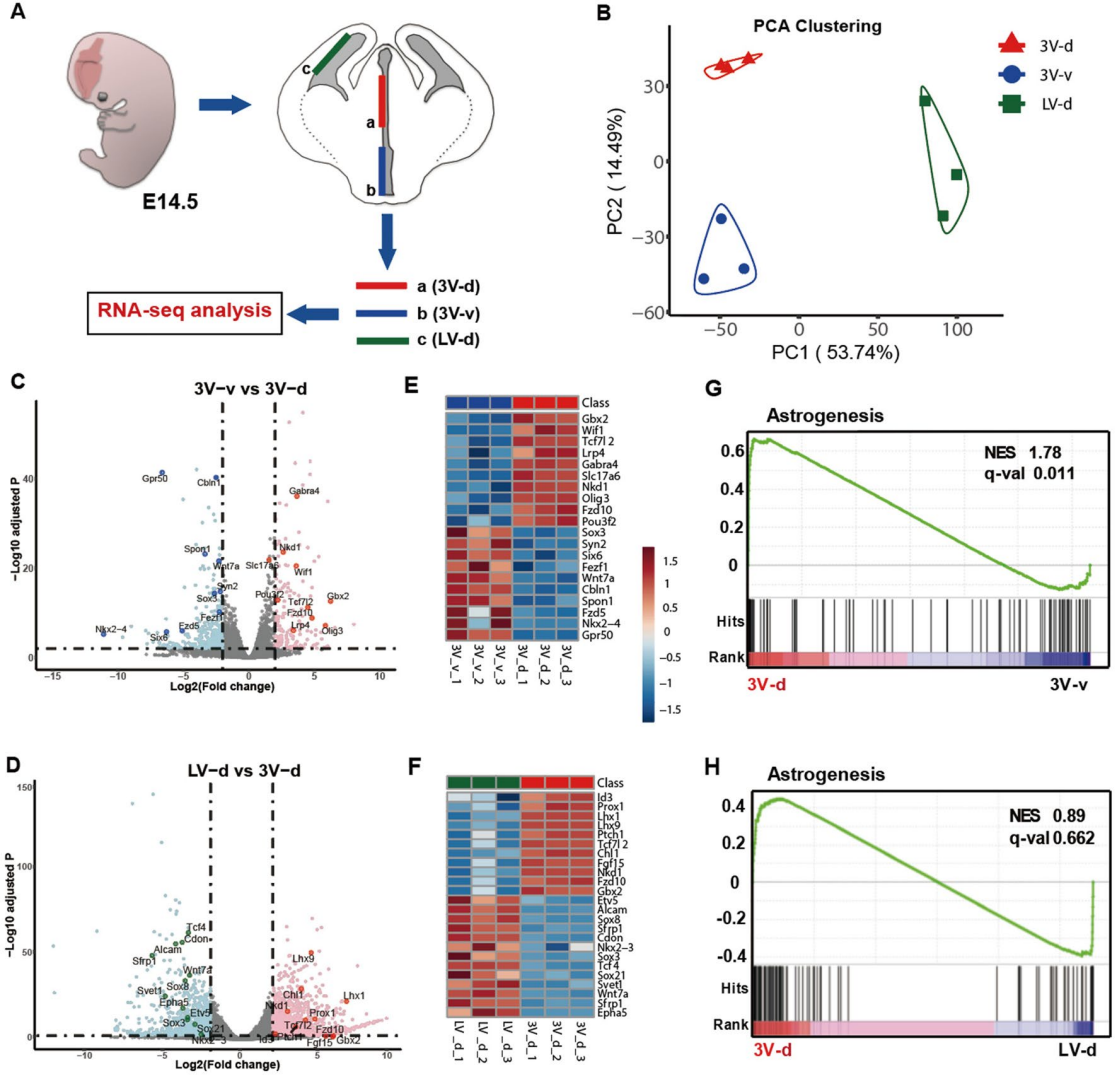
Transcriptomic profiles of cells located in the 3V wall *versus* the LV wall by RNA-Seq. **A** Schema showing the dissection of the dorsal 3V wall (a, 3V-d), the ventral 3V wall (b, 3V-v), and the dorsal LV wall (c, LV-d) of E14.5 mouse brains subjected to RNA-Seq analysis. **B** PCA cluster plots showing the variance of the three biological replicates of each group. The percentages on each axis represent the percentages of variation explained by the principal components. **C, D** Volcano plots of the differentially-expressed genes of the groups 3V-v *vs* 3V-d (**C**) and LV-d *vs* 3V-d (**D**). Significantly down-regulated genes are in light blue (sig = True) or in blue for highlighted genes, significantly up-regulated genes are in pink (sig = True) or in red for highlighted genes, and nonsignificant genes are in grey (sig = False). |log2FC| >2 and FDR <0.01. **E. F** Color scale heatmaps showing the marked genes that are highly differentially expressed of the groups 3V-v *vs* 3V-d (**E**) and LV-d *vs* 3V-d (**F**). **G, H** GSEA plots for gene sets showing the positive regulation of astrogenesis and astrocyte development, with black bars indicating gene sets represented among all genes pre-ranked by ranking metrics of groups 3V-v *vs* 3V-d (**G**), and LV-d *vs* 3V-d (**H**). The plot of false discovery rate (FDR) *versus* the normalized enrichment score (NES) based on GSEA from RNA-Seq data.

To determine the potential mechanisms governing astrogenesis in different regions of the developing diencephalon and telencephalon, we dissected the dorsal 3V wall (3V-d), the ventral 3V wall (3V-v), and the dorsal LV wall (LV-d) from E14.5 mouse brains and applied RNA-seq analysis (Fig. 8A). PCA analysis showed that the three biological replicates of each dissection group were clustered together, indicating reproducible sample dissections and high quality of RNA-seq data (Fig. 8B). Compared with the transcripts in the 3V-v group, 452 genes were up-regulated (Table S1) and 808 genes were down-regulated (Table S2) in the 3V-d group (Fig. 8C). Comparing the genes in the LV-d group, we found 3365 genes were up-regulated (Table S3) and 3070 genes were down-regulated (Table S4) in the 3V-d group (Fig. 8D). The up-regulated genes such as *Gbx2*, *Wif1*, *Slc17a6*, and *Id3* (Fig. 8E and F), may be involved in the induction of astrogenesis in the dorsal diencephalon. On the other hand, the down-regulated genes in the 3V-d group, such as *Sox3*, *Nkx2-3*, *AIcam*, and *Wnt7a* (Fig. 8E and F), may promote neurogenesis in the ventral diencephalon and the cortex. We also applied GSEA analysis of the differentially expressed genes with a geneset representing positive regulation of astrogenesis and astrocyte development (Table S5). Remarkably, we found the up-regulated genes in the 3V-d group were highly concordant with the geneset for astrogenesis (Fig. 8G and H).

## Discussion

Despite the impressive progress in understanding astrocyte differentiation from NPCs [35, 36], the spatiotemporal specification and lineage progression of astrocytes remain largely unexplored. By examining the production and expansion of astrocytes, especially in the developing mouse diencephalon, our results reveal new insights into the spatiotemporal regulation of astrocyte development in this brain region.

Radial glia translocate from the lateral VZ and become immature astrocytes in the cortex during the late embryonic or early postnatal phase [11, 37]. However, how the radial glia located within the 3V wall give rise to astrocytes in the diencephalon is not well characterized. With IUE labeling of the progenitors lining the 3V wall, we found that the radial glial cells expressing the EYFP reporter migrate out of the VZ/SVZ and later generate astrocytes in the parenchyma of the dorsal diencephalon. Interestingly, we found two distinct fates of EYFP-labeled radial glial cells based on their locations: radial glial cells from the dorsal VZ give rise to astrocytes in the thalamus, whereas those from the ventral VZ produce neurons in the hypothalamus. Our RNA-seq analysis revealed a cohort of genes that are differentially expressed in the dorsal 3V wall when compared to the ventral 3V wall or the dorsal LV wall. Future experiments are warranted to determine the key transcription factors or signaling pathways that govern the spatiotemporal behavior of radial glia.

Consistent with the CAG-EYFP labeling results of the radial glia based on IUE, progenitors in the 3V wall electroporated with the hGFAP-EGFP plasmid or transduced with the hGFAP-EGFP lentivirus differentiate into astrocytes in the dorsal diencephalon. We observed the varied morphological shapes of the astrocyte progenitors in the process of radial migration. These radially migrating cells in the dorsal diencephalon express markers for astrocytes (BLBP and Aldh1L1) but not oligodendrocytes or NG2 glia. Although evidence has shown that astrocytes and oligodendrocytes share a common lineage in the developing CNS [35, 38], our results reveal that these EYFP- or EGFP-labeled radial glial cells do not give rise to oligodendrocytes in the diencephalon. In addition, these IUE-labeled astrocytes undergo proliferation as early as E17.5. Unlike numerous astrocytic progeny derived from radial glia in the dorsal domain of the third VZ, the EGFP^+^ radial glial−like cells remain along the ventral portion of the 3V wall with long extended processes at P21. In the postnatal hypothalamus, GFAP^+^ alpha tanycytes proliferate in response to intracerebral infusion of EGF and FGF and have the potential for neurogenesis [39, 40]. Our results further indicate that these EGFP-positive tanycyte-like cells labeled at E14.5 by IUE remain largely quiescent 3 weeks after birth, similar to the adult progenitor B1 cells within the LV walls [41].

Expression of transgenes driven by the hGFAP promoter is detectable specifically in radial glia in the cortical VZ as early as E14.5 [42]. By using the StarTrack method, which is based on the combinatorial expression of six fluorescent reporter proteins under the control of the hGFAP promoter, researchers have analyzed the astrocyte lineage and revealed highly specific clonal distribution patterns in the rodent cortex [43, 44]. Our genetic fate mapping analysis of hGFAP-CreER^T2^;*Ai14* transgenic mice by tamoxifen treatment at timed embryonic stages demonstrates the temporal-spatial generation of astrocytes in the developing diencephalon as well as the telencephalon. In agreement with the sequential neuron-glia differentiation of neural precursor cells in the cortex [23], the progeny of cortical astrocytes is dramatically increased with tamoxifen induction at time points later than E14.5. The lineage tracing at E14.5−P14 of *hGFAP-CreER*^*T2*^*;Ai14* transgenic mice identified the first population of astrocytes that are distributed along the zona incerta in the diencephalon. It will be interesting to investigate which signals direct the progression of these astrocytes.

Compared with varied fate specifications of the induced tdT^+^ cells in the telencephalon, all tdT^+^ cells induced at each embryonic time point in the diencephalon give rise to S100β^+^ astrocytes. The temporal difference in the emergence of astrocytes between the telencephalon and diencephalon may be due to the relatively earlier gliogenesis in the diencephalon [45]. Unexpectedly, we found that 2% of tdT^+^ cells induced at E18.5 are IBA1^+^ microglia in the P14 cortex. To our knowledge, this result is the first showing that the hGFAP promoter can drive reporter expression in microglia in the developing cerebral cortex. This may have resulted from transient hGFAP promoter activity in very few cortical microglia during the late embryonic stage. Furthermore, microglia have recently been shown to phagocytose excessively produced astrocytes in the retina [46, 47], raising the possibility that IBA^+^ microglia may have engulfed tdT^+^ astrocytes during cortical development.

A study by *in vivo* clonal analysis demonstrated that astrogenesis in the cerebellum follows a well-defined spatiotemporal pattern and an orderly developmental program [48]. Further lineage tracing studies will be important to determine the timing of astrogenesis and regional differences of the astrocytic lineage in many other brain regions such as the olfactory bulb, hippocampus, striatum, and brain stem.

### Supplementary Information

Below is the link to the electronic supplementary material.

Supplementary file1 (PDF 217 kb)

Supplementary file2 (PDF 323 kb)

Supplementary file3 (PDF 1023 kb)

Supplementary file4 (PDF 926 kb)

Supplementary file5 (PDF 182 kb)

Supplementary file6 (PDF 2515 kb)
