# Supplementary material for: Temporal-spatial Generation of Astrocytes in the Developing Diencephalon
Source: Neurosci Bull. 2023 Oct 16;40(1):1–16. doi: 10.1007/s12264-023-01131-9 (PMC10774245; doi:10.1007/s12264-023-01131-9)
Supplement: Supplementary file 1 — Supplementary file1 (PDF 217 kb) [file 12264_2023_1131_MOESM1_ESM.pdf]

Table S1. List of genes that were up-regulated in the dorsal wall compared with the ventral wall of the 3V

SYMBOL	baseMean	log2FoldChange	lfcSE	stat	pvalue	padj	significant	3V-v_vs_3V-d_upregulated gene
Cdc45	259.8374	1.371127036	0.280601	4.88639	1.03E-06	3.31E-05	TRUE	Cdc45
Ngfr	999.0674	1.952654079	0.17012	11.47813	1.70E-30	1.67E-27	TRUE	Ngfr
Wnt3	1105.964	3.120032061	0.37473	8.326074	8.36E-17	1.70E-14	TRUE	Wnt3
Axin2	1825.932	1.840785196	0.200165	9.196345	3.70E-20	1.10E-17	TRUE	Axin2
Lhx2	7620.498	3.193278627	0.368857	8.657219	4.83E-18	1.11E-15	TRUE	Lhx2
Uhrf1	1579.91	1.118645488	0.190106	5.884336	4.00E-09	2.49E-07	TRUE	Uhrf1
Rnd2	4349.165	1.357788911	0.332846	4.079331	4.52E-05	0.000889	TRUE	Rnd2
Ube2c	965.2006	1.531696378	0.16509	9.277959	1.73E-20	5.50E-18	TRUE	Ube2c
Irx2	129.9255	4.818523538	1.349332	3.571044	0.000356	0.005044	TRUE	Irx2
Foxm1	1219.809	1.112007698	0.220994	5.031845	4.86E-07	1.69E-05	TRUE	Foxm1
Slc1a5	553.7854	1.185752325	0.207925	5.702776	1.18E-08	6.47E-07	TRUE	Slc1a5
Sipa1l2	2246.732	1.637119124	0.322731	5.072708	3.92E-07	1.40E-05	TRUE	Sipa1l2
Spag5	819.4417	1.148967585	0.249054	4.613332	3.96E-06	0.000109	TRUE	Spag5
Rgs20	313.2314	1.162953369	0.307559	3.78124	0.000156	0.002525	TRUE	Rgs20
Kcnn1	1586.786	1.070652888	0.226133	4.734606	2.19E-06	6.44E-05	TRUE	Kcnn1
Map3k20	1186.45	3.088153357	0.193366	15.9705	2.05E-57	2.12E-53	TRUE	Map3k20
Hdac9	493.4102	1.022067493	0.303837	3.363873	0.000769	0.009452	TRUE	Hdac9
Slc1a2	9730.8	2.396837	0.260203	9.211408	3.22E-20	9.66E-18	TRUE	Slc1a2
Spc25	595.2879	1.622992259	0.221435	7.329439	2.31E-13	3.07E-11	TRUE	Spc25
Asf1b	208.331	1.637877111	0.247609	6.614763	3.72E-11	3.44E-09	TRUE	Asf1b
Otx1	1350.926	3.936534054	1.016849	3.871307	0.000108	0.001843	TRUE	Otx1
Cdc20	1010.167	1.562092822	0.222558	7.018825	2.24E-12	2.60E-10	TRUE	Cdc20
Adamts4	351.1382	1.599295694	0.274827	5.819287	5.91E-09	3.54E-07	TRUE	Adamts4
Runx1t1	6207.538	1.161226398	0.188937	6.1461	7.94E-10	5.93E-08	TRUE	Runx1t1
Cav1	186.2664	1.193260585	0.321564	3.710799	0.000207	0.003175	TRUE	Cav1
Gprc5b	498.4969	1.035322837	0.248234	4.170762	3.04E-05	0.000639	TRUE	Gprc5b
Apobec3	100.971	1.680560315	0.433628	3.87558	0.000106	0.001817	TRUE	Apobec3
Nuak2	1440.09	1.060397595	0.254911	4.159869	3.18E-05	0.000666	TRUE	Nuak2
Gabra1	228.4632	1.165258657	0.288506	4.038941	5.37E-05	0.001034	TRUE	Gabra1
Kif11	1152.62	1.331846158	0.199541	6.674539	2.48E-11	2.33E-09	TRUE	Kif11
Gata3	108.0091	3.448659401	0.60382	5.711404	1.12E-08	6.20E-07	TRUE	Gata3
Ncapg	306.561	1.433257539	0.264111	5.42672	5.74E-08	2.54E-06	TRUE	Ncapg
Phf21b	6574.96	1.161537462	0.227663	5.102007	3.36E-07	1.23E-05	TRUE	Phf21b
Brca1	287.1647	1.182734298	0.206048	5.740092	9.46E-09	5.34E-07	TRUE	Brca1
Cdc6	148.4521	1.223533724	0.329329	3.715235	0.000203	0.003122	TRUE	Cdc6
Birc5	572.5251	1.361069426	0.189301	7.189992	6.48E-13	8.14E-11	TRUE	Birc5
Mybl2	480.2716	1.068884883	0.232633	4.594717	4.33E-06	0.000118	TRUE	Mybl2
Ypel2	967.9321	2.249855813	0.212562	10.58447	3.52E-26	1.92E-23	TRUE	Ypel2
Wnt9b	64.34272	3.72069932	0.53505	6.953934	3.55E-12	3.95E-10	TRUE	Wnt9b
Slc2a4	142.8159	1.394293005	0.300379	4.641773	3.45E-06	9.57E-05	TRUE	Slc2a4
Glra2	1091.712	1.882170781	0.340614	5.525815	3.28E-08	1.55E-06	TRUE	Glra2
E2f2	413.0253	1.019637544	0.210221	4.850315	1.23E-06	3.90E-05	TRUE	E2f2
Lhx9	8801.157	4.467012283	0.961046	4.648072	3.35E-06	9.32E-05	TRUE	Lhx9
Fbxo5	300.9952	1.072037186	0.294908	3.635163	0.000278	0.004088	TRUE	Fbxo5
Slc17a8	222.7785	2.625914671	0.384999	6.820578	9.07E-12	9.58E-10	TRUE	Slc17a8
Cdk1	554.0424	1.274793888	0.216494	5.888349	3.90E-09	2.45E-07	TRUE	Cdk1
Gamt	167.1656	1.036235016	0.278041	3.726909	0.000194	0.003008	TRUE	Gamt
E2f7	328.659	1.421708246	0.303883	4.678474	2.89E-06	8.17E-05	TRUE	E2f7
Wif1	156.6325	3.620235382	0.359459	10.07135	7.40E-24	3.06E-21	TRUE	Wif1
Wdpcp	326.5027	1.17951733	0.205425	5.741838	9.37E-09	5.33E-07	TRUE	Wdpcp
Hmmr	335.1073	1.283473444	0.212571	6.037865	1.56E-09	1.08E-07	TRUE	Hmmr
Pdlim4	209.9245	1.210526744	0.324099	3.73505	0.000188	0.002932	TRUE	Pdlim4
Fam49a	1340.93	1.446860527	0.270148	5.355802	8.52E-08	3.65E-06	TRUE	Fam49a
Rrm2	1607.857	1.961404436	0.221267	8.864442	7.69E-19	1.99E-16	TRUE	Rrm2
Cep112	528.1984	1.765217198	0.226585	7.790525	6.67E-15	1.11E-12	TRUE	Cep112
Pimreg	942.7659	1.59384404	0.175009	9.107216	8.45E-20	2.43E-17	TRUE	Pimreg
Cacna1g	5485.987	1.268007047	0.210654	6.019391	1.75E-09	1.20E-07	TRUE	Cacna1g
Aurkb	524.3977	1.537963117	0.207349	7.417275	1.20E-13	1.64E-11	TRUE	Aurkb
Top2a	3238.386	1.50360848	0.202327	7.431561	1.07E-13	1.48E-11	TRUE	Top2a
Dpf3	508.8896	1.392943424	0.343284	4.057697	4.96E-05	0.000964	TRUE	Dpf3
Pxdc1	143.8729	2.322976041	0.490125	4.739557	2.14E-06	6.33E-05	TRUE	Pxdc1
Mxd3	596.6057	1.60834974	0.309723	5.192858	2.07E-07	7.95E-06	TRUE	Mxd3
Rhobtb3	1804.767	1.027243677	0.181949	5.645793	1.64E-08	8.57E-07	TRUE	Rhobtb3
Hapln1	47.29481	2.443573971	0.618362	3.951691	7.76E-05	0.001409	TRUE	Hapln1
Kctd6	553.6348	2.300877863	0.203224	11.32189	1.02E-29	8.82E-27	TRUE	Kctd6
Ska3	223.4722	1.256139404	0.234915	5.3472	8.93E-08	3.79E-06	TRUE	Ska3
Amer2	6896.716	1.90977668	0.169928	11.23875	2.63E-29	2.18E-26	TRUE	Amer2

Pbk	530.0781	1.510355451	0.235058	6.42545	1.31E-10	1.12E-08	TRUE	Pbk
Nefm	3486.683	1.657101959	0.29622	5.594154	2.22E-08	1.11E-06	TRUE	Nefm
Sema5a	2456.885	2.757900861	0.305321	9.032798	1.67E-19	4.62E-17	TRUE	Sema5a
Rai14	1127.07	1.32934717	0.200391	6.633772	3.27E-11	3.05E-09	TRUE	Rai14
Shcbp1	201.3464	1.275350395	0.296752	4.297701	1.73E-05	0.000392	TRUE	Shcbp1
Atad2	586.4714	1.107592134	0.213722	5.182388	2.19E-07	8.30E-06	TRUE	Atad2
Gtse1	351.1934	1.387255872	0.288299	4.811864	1.50E-06	4.59E-05	TRUE	Gtse1
Rapgef3	465.3383	1.01784736	0.237967	4.277269	1.89E-05	0.000425	TRUE	Rapgef3
Kif21a	6120.96	1.199701044	0.177803	6.747375	1.51E-11	1.51E-09	TRUE	Kif21a
Boc	405.2023	1.954424488	0.329872	5.924804	3.13E-09	2.03E-07	TRUE	Boc
Crybg3	218.5303	1.54615249	0.388776	3.976976	6.98E-05	0.001292	TRUE	Crybg3
Mylk	338.915	1.118789735	0.319661	3.499921	0.000465	0.006288	TRUE	Mylk
Robo1	4679.027	1.523709006	0.198845	7.662798	1.82E-14	2.85E-12	TRUE	Robo1
St6gal1	2373.978	1.141059733	0.20882	5.464332	4.65E-08	2.11E-06	TRUE	St6gal1
Chaf1b	421.1892	1.152651641	0.230165	5.007932	5.50E-07	1.90E-05	TRUE	Chaf1b
Racgap1	1993.226	1.147644276	0.204675	5.607152	2.06E-08	1.03E-06	TRUE	Racgap1
Lima1	1371.873	1.334760064	0.238185	5.603876	2.10E-08	1.05E-06	TRUE	Lima1
Grm2	368.7778	1.455944645	0.304458	4.78209	1.73E-06	5.24E-05	TRUE	Grm2
Cdca3	901.9793	1.199709504	0.220964	5.429434	5.65E-08	2.52E-06	TRUE	Cdca3
Sgo1	236.6249	1.491074926	0.256746	5.807577	6.34E-09	3.73E-07	TRUE	Sgo1
Nfkbie	133.7141	1.121122787	0.260355	4.306127	1.66E-05	0.000379	TRUE	Nfkbie
Pim1	357.8113	1.217016841	0.230274	5.285084	1.26E-07	5.10E-06	TRUE	Pim1
Arhgap28	371.2172	1.631922993	0.31406	5.19621	2.03E-07	7.84E-06	TRUE	Arhgap28
Ndc80	245.6409	1.400545165	0.293061	4.77903	1.76E-06	5.30E-05	TRUE	Ndc80
Cyp1b1	87.44653	2.948787389	0.638608	4.617521	3.88E-06	0.000107	TRUE	Cyp1b1
Zfp521	2894.413	1.217635696	0.274106	4.442201	8.90E-06	0.000221	TRUE	Zfp521
Incenp	1423.926	1.002788082	0.152605	6.571121	4.99E-11	4.49E-09	TRUE	Incenp
Cd5	23.21497	3.594114941	0.941024	3.819365	0.000134	0.002215	TRUE	Cd5
Tmem132a	10126.81	1.537482045	0.162485	9.46231	3.01E-21	1.01E-18	TRUE	Tmem132a
Slc15a3	90.31055	3.190869741	0.611425	5.218741	1.80E-07	7.00E-06	TRUE	Slc15a3
Syt7	5027.418	1.094781189	0.226886	4.825238	1.40E-06	4.32E-05	TRUE	Syt7
Cemip2	5880.514	1.649464243	0.16145	10.21656	1.67E-24	7.52E-22	TRUE	Cemip2
Cdca5	297.2953	1.135120728	0.216375	5.246086	1.55E-07	6.15E-06	TRUE	Cdca5
Rcor2	7074.972	1.12599791	0.26608	4.231805	2.32E-05	0.000506	TRUE	Rcor2
Tcf7l2	26398.29	4.508075082	0.595459	7.570755	3.71E-14	5.53E-12	TRUE	Tcf7l2
Cep55	271.9083	1.38407653	0.2177	6.357735	2.05E-10	1.70E-08	TRUE	Cep55
Gsto1	577.9203	1.046710702	0.23421	4.469104	7.85E-06	0.000199	TRUE	Gsto1
Habp2	13.45447	5.235502249	1.272927	4.112965	3.91E-05	0.000789	TRUE	Habp2
Clybl	1031.704	2.952944811	0.686208	4.303282	1.68E-05	0.000383	TRUE	Clybl
Tk1	511.7391	1.126376225	0.205652	5.477093	4.32E-08	1.98E-06	TRUE	Tk1
930412O13F	5.382771	5.74567538	1.639164	3.505246	0.000456	0.006179	TRUE	4930412O13Rik
Sgo2a	120.5144	1.311989436	0.301299	4.354445	1.33E-05	0.000314	TRUE	Sgo2a
Epha4	2179.318	1.564551831	0.309178	5.060352	4.18E-07	1.49E-05	TRUE	Epha4
Efhd1	19.69976	3.679061132	0.836618	4.397539	1.09E-05	0.000264	TRUE	Efhd1
Tmem163	959.92	1.090712845	0.202171	5.395011	6.85E-08	2.99E-06	TRUE	Tmem163
Nek7	1155.047	2.993340189	0.338305	8.848045	8.91E-19	2.25E-16	TRUE	Nek7
Nr5a2	263.4436	3.585216256	0.863028	4.154228	3.26E-05	0.00068	TRUE	Nr5a2
Ube2t	136.1581	1.05224839	0.300446	3.502287	0.000461	0.00624	TRUE	Ube2t
Syt2	134.8376	1.202607543	0.281762	4.268171	1.97E-05	0.000438	TRUE	Syt2
Pou2f1	1838.365	1.014979471	0.237362	4.276091	1.90E-05	0.000427	TRUE	Pou2f1
Plxna2	4360.909	1.996648835	0.344186	5.801068	6.59E-09	3.85E-07	TRUE	Plxna2
Mcm10	267.8073	1.039897342	0.2555	4.070055	4.70E-05	0.000921	TRUE	Mcm10
Nuf2	480.8433	1.313318846	0.193853	6.77481	1.25E-11	1.28E-09	TRUE	Nuf2
Fibcd1	314.5836	1.281777198	0.300372	4.267302	1.98E-05	0.000438	TRUE	Fibcd1
Zeb2	1360.293	1.011995014	0.245533	4.121617	3.76E-05	0.000765	TRUE	Zeb2
Sapcd2	513.4718	1.189879099	0.352953	3.371209	0.000748	0.009259	TRUE	Sapcd2
Ola1	1304.489	1.013982618	0.188977	5.365631	8.07E-08	3.47E-06	TRUE	Ola1
Sp3	2446.716	1.052983371	0.234701	4.486493	7.24E-06	0.000186	TRUE	Sp3
Kif18a	194.3253	1.490215431	0.260418	5.7224	1.05E-08	5.84E-07	TRUE	Kif18a
Syt13	12466.4	4.126204645	0.252766	16.32422	6.64E-60	1.37E-55	TRUE	Syt13
Chst1	3856.035	2.349788263	0.181061	12.97785	1.63E-38	2.60E-35	TRUE	Chst1
Lrp4	3877.327	3.405082756	0.60366	5.640726	1.69E-08	8.72E-07	TRUE	Lrp4
Nusap1	839.4812	1.163811578	0.213001	5.463873	4.66E-08	2.11E-06	TRUE	Nusap1
Rad51	317.5346	1.084819143	0.252789	4.2914	1.78E-05	0.000403	TRUE	Rad51
Knl1	379.1423	1.510662728	0.279871	5.397715	6.75E-08	2.96E-06	TRUE	Knl1
Knstrn	722.3737	1.063543743	0.180702	5.885636	3.97E-09	2.48E-07	TRUE	Knstrn
Pcna	1586.533	1.018941006	0.211927	4.807971	1.52E-06	4.66E-05	TRUE	Pcna
Bub1	434.0061	1.35977066	0.23303	5.83517	5.37E-09	3.27E-07	TRUE	Bub1
Tpx2	1254.805	1.284191569	0.20097	6.389959	1.66E-10	1.39E-08	TRUE	Tpx2
E2f1	798.9514	1.178652226	0.248523	4.742627	2.11E-06	6.25E-05	TRUE	E2f1

Aurka	449.9339	1.20610616	0.198142	6.087088	1.15E-09	8.18E-08	TRUE	Aurka
Sall4	87.18212	3.326637071	0.478991	6.945096	3.78E-12	4.19E-10	TRUE	Sall4
Dsn1	301.0336	1.336407733	0.208733	6.402476	1.53E-10	1.29E-08	TRUE	Dsn1
Fam83d	244.2647	1.301804304	0.360245	3.613664	0.000302	0.004394	TRUE	Fam83d
Ect2	569.8428	1.543933113	0.237849	6.491223	8.51E-11	7.44E-09	TRUE	Ect2
Ccna2	921.8523	1.393903862	0.235634	5.915557	3.31E-09	2.13E-07	TRUE	Ccna2
Slc7a11	62.85122	2.357458113	0.387949	6.076721	1.23E-09	8.67E-08	TRUE	Slc7a11
Shox2	3490.921	6.092987849	1.061754	5.738604	9.55E-09	5.35E-07	TRUE	Shox2
Gpsm2	709.1985	1.045371285	0.185242	5.643265	1.67E-08	8.61E-07	TRUE	Gpsm2
Neurog2	7644.277	3.493467563	0.919134	3.800824	0.000144	0.002363	TRUE	Neurog2
Ndst3	781.2375	1.90214644	0.27159	7.003736	2.49E-12	2.87E-10	TRUE	Ndst3
Alpk1	153.1123	1.354245382	0.325321	4.162801	3.14E-05	0.000659	TRUE	Alpk1
Iqgap3	347.4654	1.198667299	0.235673	5.086157	3.65E-07	1.33E-05	TRUE	Iqgap3
Abca4	155.8706	1.552896133	0.311898	4.978862	6.40E-07	2.18E-05	TRUE	Abca4
Rwdd3	143.0843	1.436069036	0.423256	3.392908	0.000692	0.008685	TRUE	Rwdd3
Wls	708.2334	1.425637288	0.393804	3.620168	0.000294	0.004294	TRUE	Wls
Cth	53.56695	3.853640244	0.592233	6.506966	7.67E-11	6.78E-09	TRUE	Cth
Epha7	989.9359	1.246573297	0.219028	5.691401	1.26E-08	6.82E-07	TRUE	Epha7
Smc2	1027.342	1.16277881	0.217311	5.35075	8.76E-08	3.73E-06	TRUE	Smc2
Fmn2	1240.212	1.09751463	0.26483	4.144221	3.41E-05	0.000703	TRUE	Fmn2
Astn2	1120.081	1.219450728	0.151107	8.070126	7.02E-16	1.31E-13	TRUE	Astn2
Tal2	238.9613	4.670405158	0.319103	14.63604	1.65E-48	1.14E-44	TRUE	Tal2
Cntfr	3157.405	2.576385637	0.291519	8.837803	9.76E-19	2.43E-16	TRUE	Cntfr
Tpm2	182.034	1.536927221	0.320389	4.797069	1.61E-06	4.90E-05	TRUE	Tpm2
Pde4b	844.852	2.123158648	0.304916	6.963089	3.33E-12	3.72E-10	TRUE	Pde4b
Cachd1	2118.328	1.884950989	0.178396	10.5661	4.28E-26	2.21E-23	TRUE	Cachd1
Mycl	5989.234	1.072819088	0.179254	5.984901	2.17E-09	1.44E-07	TRUE	Mycl
Rnf220	7267.423	1.461418557	0.152571	9.57863	9.83E-22	3.39E-19	TRUE	Rnf220
Kif2c	640.0161	1.334435197	0.216727	6.157215	7.40E-10	5.59E-08	TRUE	Kif2c
Plk3	780.7592	1.469499479	0.319051	4.605839	4.11E-06	0.000112	TRUE	Plk3
Tal1	265.4283	2.66424044	0.373999	7.123665	1.05E-12	1.27E-10	TRUE	Tal1
Pla2g2f	13.73075	5.051026745	1.28459	3.932015	8.42E-05	0.001508	TRUE	Pla2g2f
Alpl	133.4358	2.912474785	0.538273	5.410778	6.28E-08	2.76E-06	TRUE	Alpl
Rspo1	126.7478	3.81133826	0.456403	8.350811	6.78E-17	1.40E-14	TRUE	Rspo1
Cdca8	1074.305	1.068794776	0.201018	5.316902	1.06E-07	4.38E-06	TRUE	Cdca8
Draxin	4181.961	1.026159149	0.299863	3.422092	0.000621	0.007984	TRUE	Draxin
Cenpa	1023.888	1.149008954	0.216386	5.309985	1.10E-07	4.48E-06	TRUE	Cenpa
Gabra4	465.1482	3.673503109	0.276877	13.26762	3.57E-40	7.38E-37	TRUE	Gabra4
Prkg2	595.2224	4.13338088	0.447638	9.233766	2.61E-20	7.95E-18	TRUE	Prkg2
Antxr2	299.4445	1.546450624	0.295485	5.233609	1.66E-07	6.48E-06	TRUE	Antxr2
Tesc	96.12541	1.706052682	0.323284	5.277256	1.31E-07	5.31E-06	TRUE	Tesc
Kntc1	322.8123	1.442026502	0.218601	6.596622	4.21E-11	3.85E-09	TRUE	Kntc1
Foxp2	3553.888	2.112632432	0.240761	8.774794	1.71E-18	4.03E-16	TRUE	Foxp2
Akr1b8	296.5723	2.304728078	0.285061	8.085028	6.22E-16	1.17E-13	TRUE	Akr1b8
Plxna4	3301.115	1.154465921	0.165226	6.987203	2.80E-12	3.21E-10	TRUE	Plxna4
Osbpl3	367.0507	1.524958163	0.356986	4.271756	1.94E-05	0.000433	TRUE	Osbpl3
Ptn	11623.85	2.102795997	0.308617	6.8136	9.52E-12	1.00E-09	TRUE	Ptn
Mad2l1	603.2963	1.036729279	0.204043	5.080938	3.76E-07	1.35E-05	TRUE	Mad2l1
Foxp1	3125.844	1.061649534	0.243898	4.352835	1.34E-05	0.000315	TRUE	Foxp1
Gas2	422.7603	1.169610562	0.337259	3.467991	0.000524	0.006917	TRUE	Gas2
Slc17a6	3939.763	1.548969366	0.149082	10.39003	2.75E-25	1.36E-22	TRUE	Slc17a6
Dbx1	392.2657	1.833462482	0.38011	4.823503	1.41E-06	4.35E-05	TRUE	Dbx1
Qprt	25.7609	2.871031614	0.594374	4.830343	1.36E-06	4.24E-05	TRUE	Qprt
Kif22	894.9158	1.003396484	0.18592	5.396918	6.78E-08	2.97E-06	TRUE	Kif22
Chst15	604.1781	1.719752212	0.216835	7.931164	2.17E-15	3.74E-13	TRUE	Chst15
Mki67	3344.82	1.322203749	0.171526	7.70849	1.27E-14	2.03E-12	TRUE	Mki67
Fgf15	482.5089	2.243186384	0.457552	4.902583	9.46E-07	3.09E-05	TRUE	Fgf15
Dock11	1420.057	1.104587529	0.217322	5.082716	3.72E-07	1.34E-05	TRUE	Dock11
Cenpi	182.7327	1.056493088	0.276728	3.817807	0.000135	0.002224	TRUE	Cenpi
Cdkl5	590.8758	1.780588155	0.177236	10.04641	9.53E-24	3.79E-21	TRUE	Cdkl5
Gpm6b	7335.306	1.364591758	0.169687	8.041836	8.85E-16	1.61E-13	TRUE	Gpm6b
Nkd1	2980.788	2.626520476	0.24355	10.7843	4.08E-27	2.41E-24	TRUE	Nkd1
Irx3	112.3358	2.498322297	0.51408	4.85979	1.18E-06	3.73E-05	TRUE	Irx3
Crnde	94.18629	2.610892213	0.470294	5.551616	2.83E-08	1.36E-06	TRUE	Crnde
Irx5	82.07729	4.100051621	0.593059	6.913398	4.73E-12	5.10E-10	TRUE	Irx5
Clmp	3210.216	1.269065992	0.164682	7.706172	1.30E-14	2.05E-12	TRUE	Clmp
Dixdc1	1761.919	1.153288745	0.22427	5.142406	2.71E-07	1.01E-05	TRUE	Dixdc1
Ccnb2	504.2469	1.111459042	0.184568	6.021947	1.72E-09	1.19E-07	TRUE	Ccnb2
Rora	1203.495	2.877453828	0.733701	3.921835	8.79E-05	0.00156	TRUE	Rora
Plscr1	133.6742	1.382098229	0.30325	4.557625	5.17E-06	0.000137	TRUE	Plscr1

Igdcc3	1273.347	1.748120198	0.223596	7.818201	5.36E-15	9.02E-13	TRUE	Igdcc3
Thsd7a	3660.933	1.978361224	0.279176	7.086433	1.38E-12	1.64E-10	TRUE	Thsd7a
'00025G04F	5257.19	2.052340663	0.166763	12.3069	8.32E-35	1.01E-31	TRUE	1700025G04Rik
Troap	286.6309	1.263716273	0.264648	4.775084	1.80E-06	5.38E-05	TRUE	Troap
Igdcc4	1214.803	1.178631654	0.212153	5.555578	2.77E-08	1.34E-06	TRUE	Igdcc4
Ank2	13940.37	1.013301507	0.139191	7.279959	3.34E-13	4.32E-11	TRUE	Ank2
Cdo1	2100.165	2.345719679	0.209182	11.21377	3.49E-29	2.78E-26	TRUE	Cdo1
Cip2a	577.6093	1.163763254	0.197187	5.901832	3.59E-09	2.28E-07	TRUE	Cip2a
Ttf2	317.6767	1.103168618	0.226155	4.877938	1.07E-06	3.44E-05	TRUE	Ttf2
Chst2	2337.764	1.805787186	0.181804	9.93263	3.00E-23	1.13E-20	TRUE	Chst2
Map3k13	1656.541	1.299384021	0.230164	5.64547	1.65E-08	8.57E-07	TRUE	Map3k13
Vav3	1126.909	2.970687649	0.294494	10.08744	6.28E-24	2.71E-21	TRUE	Vav3
Kcnk10	2083.308	1.016870629	0.151876	6.695421	2.15E-11	2.05E-09	TRUE	Kcnk10
Aspm	596.6529	1.23709873	0.272623	4.537757	5.69E-06	0.000148	TRUE	Aspm
Zbtb41	3671.273	1.497515109	0.274855	5.448381	5.08E-08	2.30E-06	TRUE	Zbtb41
Fancd2	183.3892	1.165826666	0.26075	4.471059	7.78E-06	0.000197	TRUE	Fancd2
Emid1	72.75003	1.378211098	0.362156	3.805575	0.000141	0.00233	TRUE	Emid1
Kif4	578.6052	1.020713288	0.204189	4.998857	5.77E-07	1.98E-05	TRUE	Kif4
Tmem132c	479.9376	2.557178341	0.374758	6.823538	8.88E-12	9.43E-10	TRUE	Tmem132c
Gbx2	3468.885	6.246647821	0.781626	7.991858	1.33E-15	2.35E-13	TRUE	Gbx2
Edil3	1695.996	1.677969277	0.226276	7.41559	1.21E-13	1.65E-11	TRUE	Edil3
Rsrc1	1794.848	1.585969842	0.149971	10.57519	3.88E-26	2.06E-23	TRUE	Rsrc1
Lrrn1	959.1902	1.051610761	0.219276	4.795823	1.62E-06	4.92E-05	TRUE	Lrrn1
Neurod1	835.4409	1.898825966	0.290194	6.543308	6.02E-11	5.37E-09	TRUE	Neurod1
B3galt1	1199.791	1.023205305	0.235138	4.351519	1.35E-05	0.000317	TRUE	B3galt1
Ncaph	486.9729	1.208396487	0.218267	5.536325	3.09E-08	1.47E-06	TRUE	Ncaph
Zc3h12c	1236.247	1.291273383	0.202688	6.370751	1.88E-10	1.57E-08	TRUE	Zc3h12c
Klhl35	312.3462	1.294055179	0.330202	3.918977	8.89E-05	0.001574	TRUE	Klhl35
Pdzrn3	3515.26	3.830232503	0.326399	11.73482	8.45E-32	8.75E-29	TRUE	Pdzrn3
Foxa1	110.032	4.03648365	0.541536	7.45377	9.07E-14	1.26E-11	TRUE	Foxa1
Prdm8	617.8612	2.210156129	0.363204	6.085158	1.16E-09	8.25E-08	TRUE	Prdm8
Igfbpl1	19396.31	1.511119886	0.307466	4.914754	8.89E-07	2.93E-05	TRUE	Igfbpl1
Melk	373.7057	1.297498298	0.225352	5.75764	8.53E-09	4.90E-07	TRUE	Melk
Pdzrn4	561.1903	1.126221866	0.289145	3.895005	9.82E-05	0.001702	TRUE	Pdzrn4
Megf11	1145.938	1.538354659	0.217627	7.068764	1.56E-12	1.84E-10	TRUE	Megf11
Cdh8	850.575	2.152676151	0.339002	6.350038	2.15E-10	1.77E-08	TRUE	Cdh8
Arhgap21	6785	1.014580888	0.165737	6.121647	9.26E-10	6.73E-08	TRUE	Arhgap21
Abcc12	16.50669	2.621492879	0.782634	3.349576	0.000809	0.009854	TRUE	Abcc12
Scd1	462.0218	2.094334636	0.217767	9.61731	6.76E-22	2.41E-19	TRUE	Scd1
Aff3	1736.153	1.094133753	0.236364	4.629021	3.67E-06	0.000102	TRUE	Aff3
Ppp1r14a	103.7653	1.303946575	0.349669	3.729091	0.000192	0.002991	TRUE	Ppp1r14a
Dlgap5	345.3142	1.442767495	0.243945	5.914309	3.33E-09	2.14E-07	TRUE	Dlgap5
Zbtb42	147.1939	1.323816448	0.335184	3.949527	7.83E-05	0.001419	TRUE	Zbtb42
Ckap2	356.044	1.096340797	0.250692	4.373254	1.22E-05	0.000291	TRUE	Ckap2
Rtkn2	410.1109	2.877798593	0.262523	10.96207	5.82E-28	3.57E-25	TRUE	Rtkn2
Zfp365	351.4626	2.213199127	0.342931	6.453766	1.09E-10	9.41E-09	TRUE	Zfp365
Pcdh18	485.6142	1.558988569	0.262587	5.937045	2.90E-09	1.89E-07	TRUE	Pcdh18
Igsf8	3112.626	1.682556659	0.327885	5.131549	2.87E-07	1.07E-05	TRUE	Igsf8
Cdon	597.2783	1.227166259	0.360556	3.403538	0.000665	0.00842	TRUE	Cdon
Slc22a23	2868.14	1.631400248	0.142746	11.42866	3.01E-30	2.71E-27	TRUE	Slc22a23
Cdk19	1125.151	1.123774133	0.177987	6.313798	2.72E-10	2.20E-08	TRUE	Cdk19
Prc1	650.0004	1.023362309	0.241417	4.238989	2.25E-05	0.000493	TRUE	Prc1
Arhgap18	1158.459	2.094063433	0.223413	9.373052	7.05E-21	2.32E-18	TRUE	Arhgap18
Camk1d	398.6901	1.744984622	0.454061	3.843061	0.000122	0.002042	TRUE	Camk1d
Akna	1030.712	1.324291633	0.213069	6.215321	5.12E-10	3.99E-08	TRUE	Akna
Neil3	331.6605	1.622762056	0.212956	7.620164	2.53E-14	3.91E-12	TRUE	Neil3
Cntn5	93.33233	1.53663089	0.386183	3.979019	6.92E-05	0.001283	TRUE	Cntn5
Lap3	713.579	1.244432515	0.244733	5.084868	3.68E-07	1.33E-05	TRUE	Lap3
Cited2	2546.152	1.819933947	0.25315	7.18916	6.52E-13	8.14E-11	TRUE	Cited2
Rhou	4619.207	2.808592797	0.245468	11.44179	2.59E-30	2.43E-27	TRUE	Rhou
Bub1b	766.7353	1.103520682	0.238957	4.618066	3.87E-06	0.000107	TRUE	Bub1b
Pclaf	1276.16	1.704047205	0.18747	9.089717	9.93E-20	2.78E-17	TRUE	Pclaf
Pif1	501.5704	1.130558443	0.269771	4.190806	2.78E-05	0.000593	TRUE	Pif1
Elmo1	1863.16	1.434906218	0.328392	4.36949	1.25E-05	0.000295	TRUE	Elmo1
Arhgap11a	917.9092	1.291635742	0.280093	4.611453	4.00E-06	0.000109	TRUE	Arhgap11a
Ccnb1	997.0436	1.454921566	0.242354	6.003296	1.93E-09	1.32E-07	TRUE	Ccnb1
Kif14	278.9678	1.553143787	0.265925	5.840535	5.20E-09	3.19E-07	TRUE	Kif14
Inava	553.4188	1.217645332	0.248607	4.897872	9.69E-07	3.15E-05	TRUE	Inava
Pnpla3	61.08944	1.589837476	0.391784	4.057948	4.95E-05	0.000964	TRUE	Pnpla3
'00046A07F	128.0933	2.714097212	0.339798	7.987387	1.38E-15	2.42E-13	TRUE	2700046A07Rik

Rapgef5	2103.597	1.287115426	0.231538	5.558972	2.71E-08	1.32E-06	TRUE	Rapgef5
Ncapg2	342.9093	1.142072105	0.282103	4.048423	5.16E-05	0.000998	TRUE	Ncapg2
Grap2	22.01563	3.309225495	0.936706	3.532834	0.000411	0.005679	TRUE	Grap2
Clspn	404.0913	1.348134023	0.295927	4.55563	5.22E-06	0.000138	TRUE	Clspn
Gpr153	1107.923	1.020469376	0.179982	5.669852	1.43E-08	7.59E-07	TRUE	Gpr153
Aqp6	12.37921	3.326481101	0.991913	3.353603	0.000798	0.009746	TRUE	Aqp6
Pxylp1	1133.684	2.133734389	0.267868	7.965612	1.64E-15	2.86E-13	TRUE	Pxylp1
Dmrta1	52.79903	2.160640757	0.48144	4.487876	7.19E-06	0.000185	TRUE	Dmrta1
Emx2	140.3972	2.267076734	0.329128	6.88814	5.65E-12	6.03E-10	TRUE	Emx2
Wfikkn2	79.20214	2.00846782	0.550441	3.648837	0.000263	0.003903	TRUE	Wfikkn2
Cdc25c	287.1212	1.220436391	0.319648	3.818063	0.000135	0.002224	TRUE	Cdc25c
Fzd1	1213.396	1.337673048	0.264554	5.056328	4.27E-07	1.51E-05	TRUE	Fzd1
Cnpy1	137.7239	3.467240463	0.407528	8.507975	1.77E-17	3.86E-15	TRUE	Cnpy1
Hs6st1	3113.721	1.422037979	0.19247	7.388358	1.49E-13	2.01E-11	TRUE	Hs6st1
Rtn4rl1	388.0813	2.219601338	0.33247	6.676104	2.45E-11	2.32E-09	TRUE	Rtn4rl1
Cenpe	898.4399	1.344414529	0.178907	7.514588	5.71E-14	8.32E-12	TRUE	Cenpe
Pou3f3	3518.613	2.291251655	0.607702	3.770352	0.000163	0.002618	TRUE	Pou3f3
Olig3	1801.446	5.851989368	0.956824	6.116058	9.59E-10	6.94E-08	TRUE	Olig3
Mms22l	278.5021	1.09923035	0.219729	5.002667	5.65E-07	1.94E-05	TRUE	Mms22l
Ankle1	349.4868	1.079334779	0.300924	3.58674	0.000335	0.004806	TRUE	Ankle1
Myorg	176.2855	1.423981815	0.26781	5.317135	1.05E-07	4.38E-06	TRUE	Myorg
Irx3os	19.29369	2.938447658	0.826423	3.555621	0.000377	0.005295	TRUE	Irx3os
Ticrr	293.3171	1.092634127	0.246044	4.440799	8.96E-06	0.000222	TRUE	Ticrr
Tifa	55.12977	1.377690519	0.398546	3.45679	0.000547	0.007148	TRUE	Tifa
Ttc6	57.61098	4.715143727	0.566856	8.318063	8.94E-17	1.80E-14	TRUE	Ttc6
Gpr156	785.8556	2.133328256	0.263419	8.098599	5.56E-16	1.06E-13	TRUE	Gpr156
Arl4a	2227.38	1.382549985	0.205077	6.741598	1.57E-11	1.56E-09	TRUE	Arl4a
Mis18bp1	388.8803	1.013820237	0.224932	4.507221	6.57E-06	0.00017	TRUE	Mis18bp1
Zfp473	66.22914	1.223663165	0.359102	3.407563	0.000655	0.008313	TRUE	Zfp473
Ckap2l	482.0629	1.239912461	0.226828	5.46632	4.59E-08	2.09E-06	TRUE	Ckap2l
Pou4f1	65.22484	2.862021272	0.561115	5.100596	3.39E-07	1.24E-05	TRUE	Pou4f1
Gli2	488.8395	1.069808251	0.238576	4.484145	7.32E-06	0.000187	TRUE	Gli2
Nrip1	881.8866	1.184303193	0.224702	5.270555	1.36E-07	5.47E-06	TRUE	Nrip1
Nrap	111.3159	2.681628418	0.35257	7.605947	2.83E-14	4.27E-12	TRUE	Nrap
Zfpm1	277.5079	1.20518417	0.23379	5.154989	2.54E-07	9.49E-06	TRUE	Zfpm1
Nckap5	744.1683	2.140307977	0.191732	11.16302	6.19E-29	4.74E-26	TRUE	Nckap5
610318N02R	838.7186	1.147945071	0.261325	4.39279	1.12E-05	0.000269	TRUE	2610318N02Rik
Prokr2	1946.201	4.67847978	0.331541	14.11133	3.23E-45	9.56E-42	TRUE	Prokr2
Tmem150c	766.5466	1.207930006	0.285877	4.225355	2.39E-05	0.00052	TRUE	Tmem150c
Rtn4rl2	123.7901	2.900316276	0.338429	8.569933	1.04E-17	2.28E-15	TRUE	Rtn4rl2
Lin28a	54.47141	2.357498553	0.467135	5.046715	4.49E-07	1.57E-05	TRUE	Lin28a
Rgs13	109.6408	4.066064337	0.66208	6.141349	8.18E-10	6.07E-08	TRUE	Rgs13
Eml5	4845.481	1.335058935	0.155449	8.588387	8.82E-18	1.98E-15	TRUE	Eml5
Nhlh1	872.1586	2.809626866	0.277449	10.12663	4.21E-24	1.85E-21	TRUE	Nhlh1
Kif18b	255.0498	1.120179673	0.239084	4.685303	2.80E-06	7.93E-05	TRUE	Kif18b
Flrt3	1250.103	1.724976059	0.262362	6.574789	4.87E-11	4.42E-09	TRUE	Flrt3
Fam181b	141.6755	1.761736392	0.418052	4.214155	2.51E-05	0.000541	TRUE	Fam181b
Btla	21.35866	3.16358738	0.856195	3.694936	0.00022	0.003338	TRUE	Btla
Mtcl1	4001.431	1.558141966	0.170557	9.135599	6.50E-20	1.90E-17	TRUE	Mtcl1
30043M19R	101.0269	1.857923942	0.318149	5.8398	5.23E-09	3.19E-07	TRUE	F730043M19Rik
330008L17R	67.87853	1.489634777	0.385487	3.864295	0.000111	0.001892	TRUE	A330008L17Rik
Nav2	4456.59	2.30909079	0.229317	10.06945	7.54E-24	3.06E-21	TRUE	Nav2
Robo2	3356.001	1.345804565	0.349888	3.846389	0.00012	0.002019	TRUE	Robo2
Sv2b	259.5762	2.455673909	0.436147	5.630377	1.80E-08	9.24E-07	TRUE	Sv2b
Jam2	1739.69	1.56519372	0.344046	4.549373	5.38E-06	0.000141	TRUE	Jam2
Adamts19	186.4789	3.874508356	0.347712	11.14286	7.76E-29	5.54E-26	TRUE	Adamts19
Tll1	213.3421	3.630462462	0.460972	7.875664	3.39E-15	5.75E-13	TRUE	Tll1
Shisa6	170.4611	1.280625618	0.356562	3.591593	0.000329	0.004734	TRUE	Shisa6
Pde5a	782.1863	1.794920135	0.371829	4.827267	1.38E-06	4.29E-05	TRUE	Pde5a
Hmgb2	4214.974	1.430628557	0.162512	8.803215	1.33E-18	3.24E-16	TRUE	Hmgb2
Pdlim1	120.5055	2.076631075	0.411636	5.044818	4.54E-07	1.59E-05	TRUE	Pdlim1
Cd47	2006.789	1.264826639	0.237472	5.32621	1.00E-07	4.21E-06	TRUE	Cd47
Cdca7	2252.546	3.545409867	0.243543	14.55764	5.22E-48	2.70E-44	TRUE	Cdca7
Abcd2	788.3558	2.853372382	0.221241	12.89711	4.67E-38	6.91E-35	TRUE	Abcd2
Kcnq3	961.4736	1.163129373	0.21891	5.313276	1.08E-07	4.45E-06	TRUE	Kcnq3
Dennd1b	953.2501	1.300645447	0.201295	6.461379	1.04E-10	8.99E-09	TRUE	Dennd1b
Pou3f4	1064.077	2.002440317	0.399735	5.009417	5.46E-07	1.89E-05	TRUE	Pou3f4
Foxb1	153.2031	3.208756514	0.94598	3.39199	0.000694	0.0087	TRUE	Foxb1
Ntng1	1802.585	2.108141847	0.289468	7.282825	3.27E-13	4.26E-11	TRUE	Ntng1
Gm5868	86.82321	1.344074997	0.397514	3.381202	0.000722	0.008963	TRUE	Gm5868

Pantr1	859.2581	2.608767363	0.377299	6.914324	4.70E-12	5.09E-10	TRUE	Pantr1
Tnfrsf19	875.1648	2.401806699	0.275582	8.715394	2.90E-18	6.74E-16	TRUE	Tnfrsf19
Irx1	101.7325	2.761077327	0.387559	7.124271	1.05E-12	1.27E-10	TRUE	Irx1
H2-K1	224.7332	1.232501203	0.362321	3.401684	0.00067	0.008463	TRUE	H2-K1
Rag1	30.83162	4.480448631	0.799871	5.601466	2.13E-08	1.06E-06	TRUE	Rag1
Cks2	395.9387	1.141839779	0.217405	5.252135	1.50E-07	6.00E-06	TRUE	Cks2
Grm4	645.7665	1.322362103	0.340098	3.888185	0.000101	0.001743	TRUE	Grm4
Plppr1	891.0354	1.91455093	0.359897	5.319725	1.04E-07	4.35E-06	TRUE	Plppr1
Gng8	270.7613	4.127347536	0.301239	13.70124	9.98E-43	2.30E-39	TRUE	Gng8
Gm7931	118.1474	1.043331118	0.300252	3.474853	0.000511	0.006777	TRUE	Gm7931
430093F15R	24.15571	3.363384087	0.709808	4.738445	2.15E-06	6.36E-05	TRUE	A430093F15Rik
Insm1	2970.329	1.338394507	0.192856	6.939873	3.92E-12	4.32E-10	TRUE	Insm1
Psrc1	676.6393	1.607796334	0.246747	6.515982	7.22E-11	6.42E-09	TRUE	Psrc1
Sp9	3600.352	2.711440532	0.302309	8.969101	2.99E-19	8.04E-17	TRUE	Sp9
Adgrv1	1852.478	1.361874562	0.339946	4.006144	6.17E-05	0.001168	TRUE	Adgrv1
Nr2f1	16581.38	1.100431449	0.295148	3.728403	0.000193	0.002997	TRUE	Nr2f1
Hist1h3c	71.64461	2.013132385	0.455363	4.420936	9.83E-06	0.000241	TRUE	Hist1h3c
Spdl1	178.2515	1.116870563	0.253605	4.403969	1.06E-05	0.000258	TRUE	Spdl1
Peg12	800.889	1.079973419	0.189395	5.702233	1.18E-08	6.47E-07	TRUE	Peg12
Nt5dc2	6086.864	1.11573867	0.191408	5.829098	5.57E-09	3.36E-07	TRUE	Nt5dc2
Apcdd1	2338.81	1.516268209	0.283562	5.34721	8.93E-08	3.79E-06	TRUE	Apcdd1
Klf12	912.6387	1.114710036	0.29643	3.760455	0.00017	0.0027	TRUE	Klf12
933415F23R	15.66049	3.44188821	0.991723	3.470613	0.000519	0.006868	TRUE	4933415F23Rik
Spc24	546.1954	1.412561243	0.210075	6.724066	1.77E-11	1.74E-09	TRUE	Spc24
Gas2l3	223.9437	1.450350351	0.294027	4.932714	8.11E-07	2.72E-05	TRUE	Gas2l3
Fjx1	2531.117	1.579838333	0.194683	8.114932	4.86E-16	9.32E-14	TRUE	Fjx1
Heg1	1415.908	1.177878055	0.33513	3.514688	0.00044	0.006015	TRUE	Heg1
Cenpw	202.2204	1.334771764	0.291503	4.578934	4.67E-06	0.000126	TRUE	Cenpw
Fign	2494.748	1.058237866	0.249992	4.233081	2.31E-05	0.000505	TRUE	Fign
Abracl	586.5944	1.858155025	0.274056	6.780197	1.20E-11	1.24E-09	TRUE	Abracl
310009B15R	238.9131	1.316348998	0.233217	5.644298	1.66E-08	8.59E-07	TRUE	2310009B15Rik
Kifc1	593.922	1.144655047	0.213867	5.352177	8.69E-08	3.71E-06	TRUE	Kifc1
Gm5940	14.21203	3.399073612	0.919693	3.69588	0.000219	0.003328	TRUE	Gm5940
Fzd10	578.3268	4.824800656	0.713528	6.76189	1.36E-11	1.38E-09	TRUE	Fzd10
Gm11892	14.39612	3.556474534	0.907195	3.920298	8.84E-05	0.001567	TRUE	Gm11892
Gm12131	11.56408	4.210405837	1.201222	3.505103	0.000456	0.006179	TRUE	Gm12131
Gm11549	34.86451	2.310594575	0.555869	4.156724	3.23E-05	0.000675	TRUE	Gm11549
Gm12843	22.31868	2.882394832	0.661329	4.358491	1.31E-05	0.000309	TRUE	Gm12843
610307P16R	298.7033	1.115216921	0.262235	4.252746	2.11E-05	0.000466	TRUE	2610307P16Rik
Gm14342	231.3544	1.750737435	0.301901	5.799052	6.67E-09	3.89E-07	TRUE	Gm14342
Gm12828	151.4125	5.345836894	0.506742	10.54942	5.11E-26	2.58E-23	TRUE	Gm12828
Gm13791	25.17558	5.119140071	0.899343	5.69209	1.25E-08	6.82E-07	TRUE	Gm13791
Emx2os	27.83264	3.391806834	0.673495	5.036131	4.75E-07	1.66E-05	TRUE	Emx2os
830082K12R	836.8814	1.165465406	0.205834	5.662154	1.49E-08	7.89E-07	TRUE	A830082K12Rik
Gm12827	31.94063	6.577272766	1.11186	5.915556	3.31E-09	2.13E-07	TRUE	Gm12827
230014O12R	25.3282	2.516685059	0.583933	4.309885	1.63E-05	0.000374	TRUE	C230014O12Rik
Gm13584	65.93048	3.933396186	0.629127	6.252148	4.05E-10	3.22E-08	TRUE	Gm13584
430040D24R	266.8317	1.152148374	0.286316	4.024047	5.72E-05	0.001093	TRUE	D430040D24Rik
230216N24R	730.7826	1.732675567	0.303712	5.704988	1.16E-08	6.42E-07	TRUE	B230216N24Rik
Cdk5r2	1727.78	1.025840148	0.201916	5.080541	3.76E-07	1.35E-05	TRUE	Cdk5r2
Sec14l5	222.545	3.00453614	0.330027	9.103917	8.71E-20	2.47E-17	TRUE	Sec14l5
Fancf	116.3753	1.213792386	0.287552	4.221128	2.43E-05	0.000529	TRUE	Fancf
130058E05R	239.5959	6.035949438	0.462503	13.05062	6.30E-39	1.09E-35	TRUE	D130058E05Rik
Gm20667	49.80219	1.535032934	0.403983	3.79975	0.000145	0.002372	TRUE	Gm20667
Mid1-ps1	95.96624	2.322200539	0.503909	4.608375	4.06E-06	0.000111	TRUE	Mid1-ps1
Pou3f2	5204.413	2.206886638	0.273574	8.066868	7.21E-16	1.33E-13	TRUE	Pou3f2
Fignl2	185.1449	1.470200678	0.284265	5.17194	2.32E-07	8.77E-06	TRUE	Fignl2
Gm26872	30.64791	5.091434422	0.985609	5.165773	2.39E-07	9.01E-06	TRUE	Gm26872
Pantr2	35.00602	3.104853433	0.546423	5.68214	1.33E-08	7.15E-07	TRUE	Pantr2
Gm26725	17.23712	3.673830685	1.040905	3.529457	0.000416	0.005742	TRUE	Gm26725
Gm26744	30.09177	3.226826937	0.628243	5.136269	2.80E-07	1.04E-05	TRUE	Gm26744
010110K18R	29.62071	1.994190393	0.56578	3.524674	0.000424	0.005822	TRUE	2010110K18Rik
Gm26911	49.52287	3.894475925	0.686692	5.671361	1.42E-08	7.54E-07	TRUE	Gm26911
Gm27010	94.29354	1.361765324	0.304608	4.470548	7.80E-06	0.000198	TRUE	Gm27010
Lockd	162.3803	1.019675471	0.284018	3.590174	0.00033	0.004753	TRUE	Lockd
Gm27197	90.85711	3.306498686	0.559147	5.913472	3.35E-09	2.14E-07	TRUE	Gm27197
Otx2os1	92.25194	4.825727018	0.544881	8.856486	8.26E-19	2.11E-16	TRUE	Otx2os1
Gm27243	19.71657	2.816431672	0.831884	3.385604	0.00071	0.008855	TRUE	Gm27243
Ppnr	97.77235	2.082430931	0.527898	3.944759	7.99E-05	0.001441	TRUE	Ppnr
Mir124-2hg	10317.69	1.660521859	0.202428	8.203016	2.34E-16	4.58E-14	TRUE	Mir124-2hg

Gm29506	5.593291	5.809273114	1.675073	3.468072	0.000524	0.006917	TRUE	Gm29506
Gm20125	28.24028	3.910549782	0.746581	5.237942	1.62E-07	6.37E-06	TRUE	Gm20125
430092D12R	155.8693	1.976422401	0.347953	5.680149	1.35E-08	7.19E-07	TRUE	9430092D12Rik
Gm37101	40.50179	2.040204145	0.498427	4.093283	4.25E-05	0.000845	TRUE	Gm37101
930420N18R	5.498276	5.776959993	1.630817	3.542371	0.000397	0.005512	TRUE	4930420N18Rik
Gm38154	7.314818	5.191628552	1.520315	3.414838	0.000638	0.008169	TRUE	Gm38154
Gm30340	138.6875	3.394110605	0.462528	7.338176	2.17E-13	2.89E-11	TRUE	Gm30340
500005C15R	10.36754	4.042683769	1.16932	3.457295	0.000546	0.007139	TRUE	1500005C15Rik
Gm38562	6.572777	6.039154497	1.611285	3.748035	0.000178	0.002811	TRUE	Gm38562
Gm43251	26.20653	8.025776516	1.291619	6.213732	5.17E-10	4.01E-08	TRUE	Gm43251
AI506816	750.8644	1.105362049	0.164644	6.713636	1.90E-11	1.83E-09	TRUE	AI506816
Gm43549	35.12469	1.962782793	0.500446	3.922065	8.78E-05	0.00156	TRUE	Gm43549
Gm30731	101.6018	2.063850722	0.312609	6.602023	4.06E-11	3.73E-09	TRUE	Gm30731
330102E08R	130.1564	1.407478322	0.320953	4.385308	1.16E-05	0.000277	TRUE	9330102E08Rik
Gm44593	25.82428	4.774782349	0.870394	5.485771	4.12E-08	1.89E-06	TRUE	Gm44593
Gm45072	59.51139	2.50846254	0.437039	5.739682	9.49E-09	5.34E-07	TRUE	Gm45072
Gm45426	16.64408	5.569305678	1.178384	4.726224	2.29E-06	6.69E-05	TRUE	Gm45426
030029H02R	133.4324	5.001229595	0.484719	10.31779	5.86E-25	2.75E-22	TRUE	C030029H02Rik
Gm42047	10152.54	1.375045304	0.316131	4.349601	1.36E-05	0.000319	TRUE	Gm42047
Gm35940	77.62734	2.49279119	0.49744	5.011238	5.41E-07	1.88E-05	TRUE	Gm35940
Gm20276	10.93316	4.941838399	1.261355	3.917882	8.93E-05	0.001577	TRUE	Gm20276
Gm48284	29.41234	2.523196872	0.545551	4.625043	3.75E-06	0.000103	TRUE	Gm48284
Gm4739	133.9457	1.899402103	0.353693	5.370195	7.87E-08	3.40E-06	TRUE	Gm4739
Gm34923	446.7958	4.499360159	0.408657	11.01011	3.42E-28	2.36E-25	TRUE	Gm34923
Gm48062	489.4732	1.50334292	0.305744	4.917002	8.79E-07	2.91E-05	TRUE	Gm48062
330084C13R	159.4227	1.165038913	0.249695	4.665842	3.07E-06	8.64E-05	TRUE	A330084C13Rik
10006O06R	156.8983	2.49544947	0.489051	5.102638	3.35E-07	1.23E-05	TRUE	3110006O06Rik
Gm30177	213.7004	1.713248349	0.487692	3.512975	0.000443	0.006046	TRUE	Gm30177
Gm46409	134.4168	2.263716394	0.394948	5.731677	9.94E-09	5.55E-07	TRUE	Gm46409
933429O19R	14.95772	4.01844722	1.05094	3.823668	0.000131	0.002188	TRUE	4933429O19Rik
Gm49152	25.44401	5.506660809	1.013061	5.435664	5.46E-08	2.45E-06	TRUE	Gm49152
Gm534	6.046301	5.920781944	1.633357	3.624916	0.000289	0.004225	TRUE	Gm534
Gm49153	19.39316	4.314370937	1.064635	4.05244	5.07E-05	0.000983	TRUE	Gm49153
Gm49154	5.637627	5.81238118	1.668416	3.483771	0.000494	0.006606	TRUE	Gm49154
Gm49659	99.09461	1.165898551	0.312861	3.726566	0.000194	0.003009	TRUE	Gm49659
