# Supplementary material for: Temporal-spatial Generation of Astrocytes in the Developing Diencephalon
Source: Neurosci Bull. 2023 Oct 16;40(1):1–16. doi: 10.1007/s12264-023-01131-9 (PMC10774245; doi:10.1007/s12264-023-01131-9)
Supplement: Supplementary file 2 — Supplementary file2 (PDF 323 kb) [file 12264_2023_1131_MOESM2_ESM.pdf]

Table S2. List of genes that were down-regulated in the dorsal wall compared with the ventral wall of the 3V

SYMBOL	baseMean	log2FoldChange	lfcSE	stat	pvalue	padj	significant	3V-v_vs_3V-d_downregulated gene
H19	2815.522	-2.223214433	0.328891	-6.75973	1.38E-11	1.40E-09	TRUE	H19
Wnt9a	42.45586	-2.645825957	0.786804	-3.36275	0.000772	0.009479	TRUE	Wnt9a
Slc22a18	5.804823	-6.115612978	1.659086	-3.68613	0.000228	0.003437	TRUE	Slc22a18
Glra1	30.68508	-3.714317726	0.954212	-3.89255	9.92E-05	0.001716	TRUE	Glra1
Cdh4	3527.971	-2.371350958	0.309546	-7.66075	1.85E-14	2.88E-12	TRUE	Cdh4
Lck	98.84887	-2.296618319	0.320034	-7.17617	7.17E-13	8.89E-11	TRUE	Lck
Loxl3	145.7351	-1.218635335	0.355969	-3.42343	0.000618	0.00795	TRUE	Loxl3
Hpn	15.9934	-5.790775153	1.533583	-3.77598	0.000159	0.002569	TRUE	Hpn
Gabrg1	73.56565	-2.608505132	0.474366	-5.49893	3.82E-08	1.77E-06	TRUE	Gabrg1
Fndc5	82.30762	-2.652903409	0.500439	-5.30115	1.15E-07	4.69E-06	TRUE	Fndc5
Nkx2-1	1974.639	-3.811326066	1.056658	-3.60696	0.00031	0.0045	TRUE	Nkx2-1
Itga3	597.6689	-1.021034032	0.258644	-3.94764	7.89E-05	0.001428	TRUE	Itga3
Tspan33	146.6331	-1.219603452	0.286507	-4.2568	2.07E-05	0.000458	TRUE	Tspan33
Aif1l	1021.781	-1.346505855	0.364194	-3.69723	0.000218	0.003314	TRUE	Aif1l
Npas1	171.8406	-2.967802669	0.513922	-5.77481	7.70E-09	4.45E-07	TRUE	Npas1
Mov10	291.1516	-1.552891806	0.355576	-4.36725	1.26E-05	0.000298	TRUE	Mov10
Peg3	13126.97	-1.956837792	0.37097	-5.27492	1.33E-07	5.36E-06	TRUE	Peg3
Rec8	528.1066	-1.398359816	0.254455	-5.49551	3.90E-08	1.80E-06	TRUE	Rec8
Shh	160.7052	-2.781899922	0.566367	-4.91184	9.02E-07	2.97E-05	TRUE	Shh
Dennd1c	720.4865	-1.108476455	0.199785	-5.54836	2.88E-08	1.38E-06	TRUE	Dennd1c
Ppp1r17	192.4363	-2.294588596	0.285122	-8.04775	8.43E-16	1.54E-13	TRUE	Ppp1r17
Bcam	204.3659	-2.672682603	0.48285	-5.53523	3.11E-08	1.47E-06	TRUE	Bcam
Crhr2	25.16729	-3.737185406	0.734948	-5.08496	3.68E-07	1.33E-05	TRUE	Crhr2
Stat5a	102.017	-3.565875732	0.645747	-5.5221	3.35E-08	1.58E-06	TRUE	Stat5a
Pax2	15.02102	-4.433679789	1.160286	-3.82119	0.000133	0.002203	TRUE	Pax2
Eno2	509.2976	-1.105049041	0.199456	-5.54032	3.02E-08	1.44E-06	TRUE	Eno2
Pde1c	519.2596	-1.137968704	0.291968	-3.89759	9.72E-05	0.00169	TRUE	Pde1c
Col26a1	401.1815	-1.549071801	0.391394	-3.95783	7.56E-05	0.001377	TRUE	Col26a1
Ndrg2	890.4477	-1.097687629	0.281749	-3.89597	9.78E-05	0.001697	TRUE	Ndrg2
Stxbp2	745.9141	-1.346601712	0.305986	-4.40086	1.08E-05	0.00026	TRUE	Stxbp2
Slc25a18	126.8809	-2.173368608	0.554684	-3.91821	8.92E-05	0.001577	TRUE	Slc25a18
Matk	319.9761	-1.504380003	0.325922	-4.61577	3.92E-06	0.000108	TRUE	Matk
Rasa4	762.7182	-1.659249324	0.312367	-5.31186	1.09E-07	4.46E-06	TRUE	Rasa4
Syt5	1585.807	-1.054017571	0.152189	-6.9257	4.34E-12	4.73E-10	TRUE	Syt5
Chd5	4312.124	-1.467478588	0.209323	-7.01059	2.37E-12	2.74E-10	TRUE	Chd5
Ccn4	16.70762	-7.667395658	1.438642	-5.32961	9.84E-08	4.16E-06	TRUE	Ccn4
Prlr	44.0354	-3.326657953	0.545783	-6.09521	1.09E-09	7.80E-08	TRUE	Prlr
Slc1a6	80.67616	-2.545980934	0.406213	-6.2676	3.67E-10	2.93E-08	TRUE	Slc1a6
Mef2c	383.7022	-1.135869133	0.228688	-4.9669	6.80E-07	2.30E-05	TRUE	Mef2c
Trh	208.6881	-5.021012435	0.382217	-13.1366	2.03E-39	3.82E-36	TRUE	Trh
Ephb3	957.2077	-1.299887959	0.367415	-3.53792	0.000403	0.005592	TRUE	Ephb3
Vax1	736.2331	-4.456085612	1.113517	-4.00181	6.29E-05	0.001188	TRUE	Vax1
Neurl1a	519.2329	-1.102359869	0.232803	-4.73515	2.19E-06	6.44E-05	TRUE	Neurl1a
Nphs1	22.07241	-2.804854652	0.806889	-3.47614	0.000509	0.006749	TRUE	Nphs1
P4htm	615.3244	-1.092388977	0.244906	-4.46045	8.18E-06	0.000205	TRUE	P4htm
.30043K22	102.4447	-1.214892324	0.359178	-3.38242	0.000718	0.008937	TRUE	D130043K22Rik
Hap1	13455.73	-1.169815695	0.249245	-4.69344	2.69E-06	7.69E-05	TRUE	Hap1
Fgfrl1	165.8156	-1.353910998	0.331748	-4.08114	4.48E-05	0.000885	TRUE	Fgfrl1
Mgst1	173.3695	-2.092583801	0.434549	-4.81553	1.47E-06	4.52E-05	TRUE	Mgst1
Syn2	408.0471	-2.179003234	0.253132	-8.60816	7.42E-18	1.69E-15	TRUE	Syn2
Fxyd5	98.04093	-1.75125802	0.507826	-3.44854	0.000564	0.007323	TRUE	Fxyd5
Pou6f2	616.9712	-1.012640811	0.299034	-3.38638	0.000708	0.008836	TRUE	Pou6f2
Rnf112	2202.538	-2.24898358	0.219742	-10.2347	1.39E-24	6.38E-22	TRUE	Rnf112
Sptbn4	1053.941	-1.210159635	0.259641	-4.6609	3.15E-06	8.83E-05	TRUE	Sptbn4
Etv5	177.9826	-2.270517435	0.47057	-4.82504	1.40E-06	4.32E-05	TRUE	Etv5
Bicc1	121.9066	-1.425276674	0.306759	-4.64624	3.38E-06	9.39E-05	TRUE	Bicc1
Prrt1	1221.564	-1.074612634	0.212622	-5.05409	4.32E-07	1.53E-05	TRUE	Prrt1
Itga2	89.77433	-1.800234986	0.516031	-3.48862	0.000486	0.006509	TRUE	Itga2
Lrrc27	178.8575	-1.228048974	0.278671	-4.4068	1.05E-05	0.000255	TRUE	Lrrc27
Stk32c	599.5589	-1.772710973	0.260752	-6.79844	1.06E-11	1.11E-09	TRUE	Stk32c
Fli1	187.1149	-1.191048342	0.304473	-3.91183	9.16E-05	0.001612	TRUE	Fli1
Eef1a2	1758.683	-1.886574504	0.203728	-9.26027	2.04E-20	6.30E-18	TRUE	Eef1a2
Sulf1	546.8257	-4.072320369	0.452583	-8.99796	2.30E-19	6.26E-17	TRUE	Sulf1
Matn4	20.90441	-2.898285536	0.764637	-3.79041	0.00015	0.002443	TRUE	Matn4
Sdc4	107.104	-2.02281599	0.515171	-3.92649	8.62E-05	0.001539	TRUE	Sdc4
Aldoc	179.6965	-1.87215275	0.53298	-3.51262	0.000444	0.006048	TRUE	Aldoc
Pipox	300.412	-1.219909662	0.362016	-3.36977	0.000752	0.009296	TRUE	Pipox
Rarb	37.04885	-2.532113975	0.742557	-3.40999	0.00065	0.00827	TRUE	Rarb
Etv4	87.67524	-2.173943834	0.449965	-4.83136	1.36E-06	4.22E-05	TRUE	Etv4

Slc12a5	2284.087	-1.834065949	0.263374	-6.96372	3.31E-12	3.72E-10	TRUE		Slc12a5
Jph2	33.83555	-1.8870911	0.508958	-3.70776	0.000209	0.003206	TRUE		Jph2
Eya2	66.21444	-2.323292351	0.5117	-4.54034	5.62E-06	0.000147	TRUE		Eya2
Cadps2	280.065	-1.654134616	0.336034	-4.92253	8.54E-07	2.84E-05	TRUE		Cadps2
Anxa6	510.864	-1.162162318	0.341986	-3.39828	0.000678	0.008553	TRUE		Anxa6
Dusp14	69.92726	-2.629360779	0.59109	-4.44833	8.65E-06	0.000216	TRUE		Dusp14
Cacng2	1775.172	-1.032839845	0.181081	-5.70375	1.17E-08	6.45E-07	TRUE		Cacng2
Scn1b	47.87828	-2.435233907	0.600916	-4.05254	5.07E-05	0.000983	TRUE		Scn1b
Gyg	187.1924	-1.186185957	0.352265	-3.36731	0.000759	0.009369	TRUE		Gyg
Cyb561	841.9097	-1.002130424	0.237497	-4.21955	2.45E-05	0.000531	TRUE		Cyb561
Tmc4	237.5924	-1.719043723	0.410844	-4.18418	2.86E-05	0.000606	TRUE		Tmc4
Hey2	73.15627	-1.916636822	0.410129	-4.67326	2.96E-06	8.37E-05	TRUE		Hey2
Fuca2	194.679	-1.560273546	0.317756	-4.91029	9.09E-07	2.98E-05	TRUE		Fuca2
Crybg1	81.65546	-3.613940927	0.67937	-5.31955	1.04E-07	4.35E-06	TRUE		Crybg1
Nts	17.08205	-4.077922251	1.09448	-3.7259	0.000195	0.003015	TRUE		Nts
Cfap54	86.86937	-1.279113559	0.355121	-3.60191	0.000316	0.004579	TRUE		Cfap54
Tmcc3	327.6046	-1.732303491	0.357508	-4.84549	1.26E-06	3.98E-05	TRUE		Tmcc3
Meis1	471.7979	-1.175282771	0.336076	-3.49707	0.00047	0.006339	TRUE		Meis1
Rab36	820.114	-1.043480829	0.263628	-3.95816	7.55E-05	0.001376	TRUE		Rab36
Ddc	464.0827	-3.070423573	0.24001	-12.7929	1.80E-37	2.48E-34	TRUE		Ddc
Shc2	876.2377	-1.758340244	0.292119	-6.01927	1.75E-09	1.20E-07	TRUE		Shc2
Tns3	354.3106	-1.40573264	0.345657	-4.06684	4.77E-05	0.000933	TRUE		Tns3
Myl7	16.14084	-5.567633375	1.370438	-4.06267	4.85E-05	0.000948	TRUE		Myl7
Gria1	2332.351	-1.347435056	0.260803	-5.16649	2.39E-07	8.99E-06	TRUE		Gria1
Abca8b	108.7102	-2.374799687	0.425709	-5.57846	2.43E-08	1.20E-06	TRUE		Abca8b
Asic2	603.2228	-1.203402338	0.281377	-4.27683	1.90E-05	0.000426	TRUE		Asic2
Slc9a3r1	492.875	-1.354740784	0.27442	-4.93675	7.94E-07	2.67E-05	TRUE		Slc9a3r1
Tekt1	187.9239	-2.235199582	0.294443	-7.59127	3.17E-14	4.75E-12	TRUE		Tekt1
Pld2	171.1959	-2.169215061	0.368514	-5.88639	3.95E-09	2.48E-07	TRUE		Pld2
Rph3al	82.74557	-3.592168946	0.736957	-4.87433	1.09E-06	3.49E-05	TRUE		Rph3al
Doc2b	320.7466	-2.829602885	0.462987	-6.11162	9.86E-10	7.09E-08	TRUE		Doc2b
Pygl	60.25173	-1.677822992	0.442608	-3.79077	0.00015	0.002442	TRUE		Pygl
Gsc	170.7604	-8.988824727	1.48489	-6.05353	1.42E-09	9.94E-08	TRUE		Gsc
30447C04I	345.7068	-3.748101713	0.460398	-8.14101	3.92E-16	7.58E-14	TRUE		4930447C04Rik
Six6	1278.923	-6.292180622	1.145588	-5.49253	3.96E-08	1.83E-06	TRUE		Six6
Smoc1	687.5028	-2.284056769	0.424012	-5.38677	7.17E-08	3.12E-06	TRUE		Smoc1
Papln	46.10732	-3.87615284	0.822518	-4.71255	2.45E-06	7.08E-05	TRUE		Papln
Meg3	104200.3	-1.713265982	0.205614	-8.33246	7.92E-17	1.62E-14	TRUE		Meg3
Gng4	518.6705	-1.825635913	0.318066	-5.7398	9.48E-09	5.34E-07	TRUE		Gng4
Gpld1	66.13465	-1.799839035	0.374335	-4.8081	1.52E-06	4.66E-05	TRUE		Gpld1
Sema4d	584.8496	-1.009863659	0.262013	-3.85425	0.000116	0.001965	TRUE		Sema4d
Ror2	54.01701	-1.646565935	0.458015	-3.595	0.000324	0.004689	TRUE		Ror2
00001L19I	54.28611	-1.378598003	0.385761	-3.57371	0.000352	0.005007	TRUE		1700001L19Rik
Slc28a3	10.56132	-5.215963793	1.463029	-3.56518	0.000364	0.00513	TRUE		Slc28a3
Pcsk1	51.67089	-2.398274092	0.663305	-3.61564	0.0003	0.004367	TRUE		Pcsk1
Mctp1	59.46842	-1.858568807	0.539839	-3.44282	0.000576	0.007461	TRUE		Mctp1
Marveld2	72.33004	-1.929968011	0.568314	-3.39596	0.000684	0.008615	TRUE		Marveld2
Cartpt	191.8793	-1.953580296	0.552799	-3.53398	0.000409	0.005667	TRUE		Cartpt
Rasgrf2	288.1876	-3.610002348	0.507632	-7.11146	1.15E-12	1.37E-10	TRUE		Rasgrf2
Fezf2	259.2421	-2.099530292	0.519605	-4.04063	5.33E-05	0.001028	TRUE		Fezf2
Fam107a	45.76756	-3.737549411	0.886644	-4.21539	2.49E-05	0.000539	TRUE		Fam107a
Hr	78.19446	-1.516652443	0.428676	-3.538	0.000403	0.005592	TRUE		Hr
Dmtn	2368.423	-1.059514193	0.141857	-7.46887	8.09E-14	1.15E-11	TRUE		Dmtn
Gfra2	398.2757	-1.490414615	0.210383	-7.08429	1.40E-12	1.65E-10	TRUE		Gfra2
Cpne6	97.80866	-1.562600432	0.37839	-4.1296	3.63E-05	0.000745	TRUE		Cpne6
Khdrbs3	2152.188	-1.284560783	0.1998	-6.42924	1.28E-10	1.10E-08	TRUE		Khdrbs3
Sybu	988.2767	-1.505958929	0.308101	-4.88788	1.02E-06	3.29E-05	TRUE		Sybu
Col14a1	46.21144	-2.487178403	0.523771	-4.7486	2.05E-06	6.09E-05	TRUE		Col14a1
Col2a1	4528.096	-1.497479811	0.375638	-3.9865	6.71E-05	0.00125	TRUE		Col2a1
Bcl6	27.35624	-2.967438335	0.692945	-4.28236	1.85E-05	0.000416	TRUE		Bcl6
Hes1	426.1621	-1.735545806	0.334562	-5.18752	2.13E-07	8.12E-06	TRUE		Hes1
Ly6h	8225.051	-1.342119588	0.276209	-4.85908	1.18E-06	3.74E-05	TRUE		Ly6h
Alcam	507.2443	-2.697270782	0.215616	-12.5096	6.62E-36	8.56E-33	TRUE		Alcam
Igsf11	142.1963	-2.144132929	0.53219	-4.02888	5.60E-05	0.001073	TRUE		Igsf11
Grik1	238.6113	-1.205385687	0.235106	-5.12699	2.94E-07	1.09E-05	TRUE		Grik1
Zfp641	452.2646	-1.082189755	0.237878	-4.54934	5.38E-06	0.000141	TRUE		Zfp641
Faim2	345.9714	-1.580724626	0.204432	-7.73229	1.06E-14	1.69E-12	TRUE		Faim2
Sncg	72.32007	-2.286546346	0.542276	-4.21657	2.48E-05	0.000536	TRUE		Sncg
Dlx2	1901.643	-1.984565471	0.265815	-7.46597	8.27E-14	1.16E-11	TRUE		Dlx2
Prkn	154.3192	-1.075128756	0.27668	-3.88582	0.000102	0.001759	TRUE		Prkn
Slc5a7	55.90663	-2.382293426	0.656614	-3.62815	0.000285	0.004184	TRUE		Slc5a7
Calcr	170.4716	-5.771620846	1.40857	-4.0975	4.18E-05	0.000833	TRUE		Calcr

Cpne5	400.769	-1.446666306	0.266223	-5.43405	5.51E-08	2.46E-06	TRUE	Cpne5
Pi16	261.8865	-1.490461603	0.310097	-4.80644	1.54E-06	4.69E-05	TRUE	Pi16
Clip4	445.0081	-1.187412834	0.277157	-4.28427	1.83E-05	0.000413	TRUE	Clip4
Sox8	157.9517	-2.130684116	0.566409	-3.76174	0.000169	0.002689	TRUE	Sox8
Rgs11	113.7019	-3.601981033	1.030459	-3.49551	0.000473	0.006368	TRUE	Rgs11
Pkdcc	544.4497	-1.514610288	0.235235	-6.43871	1.20E-10	1.03E-08	TRUE	Pkdcc
Adcyap1	292.935	-2.072699317	0.250167	-8.28527	1.18E-16	2.35E-14	TRUE	Adcyap1
Dtna	454.6062	-2.331524799	0.379252	-6.14769	7.86E-10	5.90E-08	TRUE	Dtna
Aqp4	34.43177	-5.015513553	1.137002	-4.41117	1.03E-05	0.000251	TRUE	Aqp4
Mal2	48.65485	-3.035835319	0.763651	-3.97542	7.03E-05	0.001298	TRUE	Mal2
Ppp2r2b	1519.453	-1.097427488	0.281047	-3.90478	9.43E-05	0.00165	TRUE	Ppp2r2b
Rax	146.07	-6.885984022	1.939991	-3.54949	0.000386	0.005389	TRUE	Rax
Galr1	11.55395	-7.147117948	1.521248	-4.69819	2.62E-06	7.53E-05	TRUE	Galr1
Sall3	626.3304	-1.568542406	0.281093	-5.58015	2.40E-08	1.19E-06	TRUE	Sall3
Camk2a	2165.472	-2.240210822	0.204035	-10.9795	4.79E-28	3.20E-25	TRUE	Camk2a
Asrgl1	416.2754	-1.042988496	0.186547	-5.59103	2.26E-08	1.12E-06	TRUE	Asrgl1
Aldh3b1	28.88887	-1.891567623	0.552911	-3.42111	0.000624	0.008003	TRUE	Aldh3b1
Gal	27.44584	-2.293033182	0.56754	-4.0403	5.34E-05	0.001029	TRUE	Gal
Plce1	381.2348	-1.888376914	0.323771	-5.83245	5.46E-09	3.31E-07	TRUE	Plce1
Vwa2	42.16516	-2.589651097	0.724801	-3.57291	0.000353	0.005015	TRUE	Vwa2
Hspa12a	1620.737	-1.360619575	0.248007	-5.48622	4.11E-08	1.89E-06	TRUE	Hspa12a
St8sia5	44.81713	-1.837435844	0.518669	-3.5426	0.000396	0.005511	TRUE	St8sia5
Caly	795.8859	-1.359653318	0.192462	-7.06452	1.61E-12	1.88E-10	TRUE	Caly
Adgra1	427.3398	-1.354041491	0.249513	-5.42673	5.74E-08	2.54E-06	TRUE	Adgra1
Fgf14	300.8903	-1.411985703	0.288121	-4.90066	9.55E-07	3.11E-05	TRUE	Fgf14
Dock9	524.0681	-1.324702359	0.337249	-3.92797	8.57E-05	0.001531	TRUE	Dock9
Gaa	1646.561	-1.052283261	0.208119	-5.05617	4.28E-07	1.51E-05	TRUE	Gaa
Nptx1	729.0477	-1.305678979	0.193542	-6.74623	1.52E-11	1.52E-09	TRUE	Nptx1
Col7a1	201.8984	-1.070504756	0.308103	-3.4745	0.000512	0.006782	TRUE	Col7a1
Tmem47	1104.339	-1.768935252	0.383244	-4.61569	3.92E-06	0.000108	TRUE	Tmem47
Slco3a1	772.9663	-1.220271122	0.19336	-6.31086	2.77E-10	2.23E-08	TRUE	Slco3a1
Pdgfa	637.2088	-1.609793095	0.380361	-4.23227	2.31E-05	0.000506	TRUE	Pdgfa
Tspan17	467.7383	-1.640247564	0.275128	-5.96176	2.50E-09	1.63E-07	TRUE	Tspan17
Unc5a	1790.643	-1.561834725	0.30287	-5.15678	2.51E-07	9.42E-06	TRUE	Unc5a
Gria4	1235.722	-1.635731511	0.278273	-5.87815	4.15E-09	2.58E-07	TRUE	Gria4
Col5a2	593.3557	-1.13498351	0.335764	-3.3803	0.000724	0.008985	TRUE	Col5a2
10300C02I	89.68035	-1.944731103	0.450094	-4.32072	1.56E-05	0.000359	TRUE	2010300C02Rik
Igfbp5	5439.234	-1.264441698	0.304733	-4.14934	3.33E-05	0.000689	TRUE	Igfbp5
Speg	1000.768	-1.085583809	0.199597	-5.43889	5.36E-08	2.41E-06	TRUE	Speg
33407L21I	111.2493	-2.15369376	0.390942	-5.50899	3.61E-08	1.68E-06	TRUE	4933407L21Rik
Ngef	155.0215	-1.321590634	0.295739	-4.46878	7.87E-06	0.000199	TRUE	Ngef
Ppfia4	609.6225	-1.499094126	0.275667	-5.43807	5.39E-08	2.42E-06	TRUE	Ppfia4
Cfap45	69.82973	-1.884850888	0.429878	-4.38461	1.16E-05	0.000277	TRUE	Cfap45
Tbx19	40.06601	-6.58609985	1.777976	-3.70427	0.000212	0.003239	TRUE	Tbx19
Lamb3	91.251	-2.091436995	0.379148	-5.51614	3.47E-08	1.63E-06	TRUE	Lamb3
Ddr2	69.94328	-1.48198547	0.360173	-4.11465	3.88E-05	0.000785	TRUE	Ddr2
Enkur	75.72797	-2.269687846	0.674376	-3.36561	0.000764	0.009404	TRUE	Enkur
Pter	66.94454	-2.346754075	0.592385	-3.96153	7.45E-05	0.00136	TRUE	Pter
Plxdc2	183.1113	-1.141554783	0.307806	-3.70869	0.000208	0.003197	TRUE	Plxdc2
Nr5a1	646.3005	-5.8207166	1.386968	-4.19672	2.71E-05	0.00058	TRUE	Nr5a1
Lypd6b	76.78973	-3.140720314	0.643027	-4.88428	1.04E-06	3.34E-05	TRUE	Lypd6b
Ak8	15.50416	-4.412849507	1.101432	-4.00646	6.16E-05	0.001167	TRUE	Ak8
Kcnj3	116.1528	-1.172448452	0.274303	-4.27428	1.92E-05	0.000429	TRUE	Kcnj3
Dnm1	3170.33	-1.204831722	0.226211	-5.32614	1.00E-07	4.21E-06	TRUE	Dnm1
Col5a1	333.9379	-1.329706672	0.357485	-3.71962	0.0002	0.00308	TRUE	Col5a1
Ifih1	26.02176	-2.302178844	0.663971	-3.46729	0.000526	0.006927	TRUE	Ifih1
Wdsub1	210.3947	-1.335025283	0.311545	-4.28518	1.83E-05	0.000412	TRUE	Wdsub1
Itga6	742.225	-1.54822063	0.248598	-6.2278	4.73E-10	3.71E-08	TRUE	Itga6
Gfra4	177.5486	-1.222920759	0.259205	-4.71797	2.38E-06	6.93E-05	TRUE	Gfra4
Fsip1	34.00041	-2.916622819	0.62183	-4.69038	2.73E-06	7.79E-05	TRUE	Fsip1
Slc27a2	260.9917	-1.394802364	0.303041	-4.60269	4.17E-06	0.000114	TRUE	Slc27a2
Hdc	51.12449	-2.169116957	0.469998	-4.61517	3.93E-06	0.000108	TRUE	Hdc
Prom2	37.57473	-5.017126135	1.13911	-4.40443	1.06E-05	0.000257	TRUE	Prom2
Pdyn	215.3073	-1.721375541	0.355554	-4.84139	1.29E-06	4.04E-05	TRUE	Pdyn
Nkx2-2	553.7696	-1.812280083	0.374009	-4.84555	1.26E-06	3.98E-05	TRUE	Nkx2-2
Acss1	561.8833	-1.760852729	0.409423	-4.30081	1.70E-05	0.000387	TRUE	Acss1
Ccm2l	97.09768	-1.071789754	0.307322	-3.48752	0.000488	0.006527	TRUE	Ccm2l
Tpd52	532.748	-1.245481051	0.344511	-3.61522	0.0003	0.004371	TRUE	Tpd52
Zdbf2	5698.594	-1.189123245	0.309431	-3.84294	0.000122	0.002042	TRUE	Zdbf2
Nfatc2	117.4389	-1.230949043	0.317514	-3.87683	0.000106	0.001812	TRUE	Nfatc2
Ntsr1	113.95	-3.900432384	0.634881	-6.14356	8.07E-10	6.01E-08	TRUE	Ntsr1
Col9a3	864.6335	-1.270821161	0.354842	-3.58137	0.000342	0.004889	TRUE	Col9a3

Ghrh	29.52125	-2.054039798	0.571948	-3.59131	0.000329	0.004736	TRUE	Ghrh
Pld1	74.1482	-1.685448683	0.449308	-3.75121	0.000176	0.002778	TRUE	Pld1
Anxa5	428.9144	-1.877307442	0.338008	-5.55404	2.79E-08	1.35E-06	TRUE	Anxa5
Trpc4	80.36351	-2.845389297	0.617384	-4.60878	4.05E-06	0.000111	TRUE	Trpc4
Mme	43.57825	-3.047137261	0.740812	-4.11324	3.90E-05	0.000789	TRUE	Mme
Syt6	244.1914	-3.477241099	1.035359	-3.35849	0.000784	0.009604	TRUE	Syt6
Hmgcs2	50.81912	-1.821127358	0.527789	-3.45048	0.00056	0.00728	TRUE	Hmgcs2
Notch2	1198.899	-2.311468125	0.351377	-6.57832	4.76E-11	4.34E-09	TRUE	Notch2
Slc6a17	1635.202	-1.482225302	0.281172	-5.2716	1.35E-07	5.45E-06	TRUE	Slc6a17
Kcnc4	85.60737	-1.857972289	0.52784	-3.51995	0.000432	0.005905	TRUE	Kcnc4
Gask1b	47.21879	-2.650582948	0.727665	-3.64259	0.00027	0.00399	TRUE	Gask1b
Olfm3	81.75337	-1.730771238	0.476966	-3.62871	0.000285	0.004178	TRUE	Olfm3
Npy2r	8.648905	-4.90453469	1.396678	-3.51157	0.000445	0.006062	TRUE	Npy2r
Ntrk1	12.70005	-4.265203186	1.129624	-3.77577	0.00016	0.002569	TRUE	Ntrk1
Rorc	80.2814	-3.15087353	0.588468	-5.35437	8.59E-08	3.67E-06	TRUE	Rorc
Dapp1	38.03416	-1.751208355	0.522356	-3.35252	0.000801	0.009779	TRUE	Dapp1
Lmo4	3618.859	-1.251867491	0.237331	-5.27476	1.33E-07	5.36E-06	TRUE	Lmo4
Cfap206	44.45218	-2.457855866	0.609426	-4.03306	5.51E-05	0.001055	TRUE	Cfap206
Bspry	53.61659	-3.053954518	0.698552	-4.37183	1.23E-05	0.000292	TRUE	Bspry
Slc44a1	449.4259	-1.048430024	0.262262	-3.99764	6.40E-05	0.001207	TRUE	Slc44a1
Epb41l4b	233.1733	-1.109494001	0.285938	-3.88018	0.000104	0.00179	TRUE	Epb41l4b
Tnfrsf8	15.70596	-3.262183318	0.958504	-3.40341	0.000665	0.00842	TRUE	Tnfrsf8
Col9a2	811.4966	-2.284830439	0.303605	-7.52567	5.24E-14	7.70E-12	TRUE	Col9a2
Vwa5b1	154.0361	-2.326632901	0.410497	-5.66784	1.45E-08	7.65E-07	TRUE	Vwa5b1
Kif17	91.29437	-1.77957957	0.448729	-3.96582	7.31E-05	0.00134	TRUE	Kif17
Hpca	211.328	-1.685848855	0.39673	-4.24936	2.14E-05	0.000473	TRUE	Hpca
Csmd2	2690.722	-1.219381487	0.20332	-5.99735	2.01E-09	1.36E-07	TRUE	Csmd2
Ptpru	628.5053	-1.627733465	0.399653	-4.07287	4.64E-05	0.000911	TRUE	Ptpru
Arhgef19	277.1402	-2.323415281	0.509396	-4.56112	5.09E-06	0.000135	TRUE	Arhgef19
Per3	191.0624	-1.233803474	0.30917	-3.9907	6.59E-05	0.001232	TRUE	Per3
Agtrap	174.9609	-1.323608374	0.298619	-4.43243	9.32E-06	0.00023	TRUE	Agtrap
Ttll10	13.05156	-7.318248667	1.686775	-4.3386	1.43E-05	0.000335	TRUE	Ttll10
Ablim2	109.5378	-1.418511383	0.37119	-3.82152	0.000133	0.002201	TRUE	Ablim2
Pcdh7	616.9493	-1.506299598	0.328364	-4.58728	4.49E-06	0.000121	TRUE	Pcdh7
Ppp2r2c	1112.449	-1.903698773	0.385648	-4.93637	7.96E-07	2.67E-05	TRUE	Ppp2r2c
Cgref1	405.0237	-1.031063664	0.271088	-3.80342	0.000143	0.002344	TRUE	Cgref1
Klf3	264.4793	-1.517075974	0.275069	-5.51526	3.48E-08	1.63E-06	TRUE	Klf3
Crybb3	57.57246	-2.237499104	0.573034	-3.90465	9.44E-05	0.00165	TRUE	Crybb3
Cdkl2	75.30189	-2.876103708	0.695084	-4.13778	3.51E-05	0.000722	TRUE	Cdkl2
Naaa	73.74663	-1.806636163	0.452314	-3.9942	6.49E-05	0.001219	TRUE	Naaa
Ajm1	643.6477	-1.103232941	0.187273	-5.89104	3.84E-09	2.42E-07	TRUE	Ajm1
Rimbp2	630.3829	-1.290885918	0.375139	-3.44109	0.000579	0.007504	TRUE	Rimbp2
Wdr66	121.2064	-2.446562525	0.394563	-6.20069	5.62E-10	4.33E-08	TRUE	Wdr66
Cabp1	249.8412	-1.432725992	0.303632	-4.71863	2.37E-06	6.93E-05	TRUE	Cabp1
Rasal1	26.51564	-3.639877966	0.720745	-5.05016	4.41E-07	1.55E-05	TRUE	Rasal1
Rph3a	1555.311	-1.301268277	0.289216	-4.4993	6.82E-06	0.000176	TRUE	Rph3a
Col1a2	164.8308	-2.792511954	0.576893	-4.84061	1.29E-06	4.05E-05	TRUE	Col1a2
Tspan12	344.9799	-1.111504825	0.318724	-3.48736	0.000488	0.006527	TRUE	Tspan12
Fezf1	1843.584	-2.254903053	0.311958	-7.22823	4.89E-13	6.21E-11	TRUE	Fezf1
Dlx6	1734.789	-1.823942397	0.303936	-6.00108	1.96E-09	1.33E-07	TRUE	Dlx6
Dlx5	1055.683	-1.407047529	0.32583	-4.31834	1.57E-05	0.000362	TRUE	Dlx5
Dync1i1	107.434	-1.49051733	0.404976	-3.68051	0.000233	0.003506	TRUE	Dync1i1
Dbpht2	348.744	-4.035970496	0.361737	-11.1572	6.60E-29	4.88E-26	TRUE	Dbpht2
Rab19	12.65404	-4.860418903	1.360049	-3.57371	0.000352	0.005007	TRUE	Rab19
Tgfa	249.9999	-2.205325489	0.448875	-4.91301	8.97E-07	2.96E-05	TRUE	Tgfa
Adamts9	120.3972	-2.499626122	0.477231	-5.23777	1.63E-07	6.37E-06	TRUE	Adamts9
00003E16I	124.4093	-1.531071819	0.432176	-3.5427	0.000396	0.005511	TRUE	1700003E16Rik
Wnt7a	604.3076	-2.289983265	0.221586	-10.3345	4.92E-25	2.37E-22	TRUE	Wnt7a
Rasgef1a	191.8266	-2.092030649	0.358697	-5.83231	5.47E-09	3.31E-07	TRUE	Rasgef1a
Fam234b	2356.084	-1.061306344	0.168794	-6.28758	3.22E-10	2.59E-08	TRUE	Fam234b
Slc6a11	182.9274	-2.202011613	0.298436	-7.37851	1.60E-13	2.15E-11	TRUE	Slc6a11
Slc6a1	3729.858	-1.322742838	0.21608	-6.12155	9.27E-10	6.73E-08	TRUE	Slc6a1
Timp4	225.9788	-3.557093604	0.839232	-4.23851	2.25E-05	0.000494	TRUE	Timp4
Nr2f2	8402.147	-1.207536636	0.222453	-5.42828	5.69E-08	2.53E-06	TRUE	Nr2f2
Synm	179.6024	-1.058040641	0.301783	-3.50596	0.000455	0.006167	TRUE	Synm
Rasgrp4	50.7263	-1.509630039	0.418062	-3.61102	0.000305	0.004436	TRUE	Rasgrp4
Xylt1	264.1607	-1.824012314	0.295008	-6.18293	6.29E-10	4.82E-08	TRUE	Xylt1
Syt3	430.2006	-1.009502718	0.266991	-3.78104	0.000156	0.002525	TRUE	Syt3
Myh14	259.8019	-3.032385691	0.48659	-6.23191	4.61E-10	3.63E-08	TRUE	Myh14
Rgs10	232.3026	-1.384638491	0.368265	-3.7599	0.00017	0.002702	TRUE	Rgs10
Ern2	44.4155	-3.137231508	0.539209	-5.81821	5.95E-09	3.54E-07	TRUE	Ern2
Vwa3a	68.98558	-1.364037008	0.38585	-3.53515	0.000408	0.005646	TRUE	Vwa3a

Tmem159	105.9335	-1.790171076	0.340968	-5.25025	1.52E-07	6.05E-06	TRUE	Tmem159
Trim66	126.5101	-1.492253739	0.398336	-3.74621	0.00018	0.002826	TRUE	Trim66
Igsf1	95.49287	-2.935599867	0.642339	-4.57017	4.87E-06	0.00013	TRUE	Igsf1
Gpc4	199.3447	-3.388227695	0.342478	-9.89326	4.45E-23	1.62E-20	TRUE	Gpc4
Slc9a9	157.773	-1.216408107	0.302887	-4.01605	5.92E-05	0.001126	TRUE	Slc9a9
Col4a6	66.68028	-2.87929751	0.684337	-4.20742	2.58E-05	0.000556	TRUE	Col4a6
Col4a5	164.8477	-1.910415478	0.389721	-4.90201	9.49E-07	3.10E-05	TRUE	Col4a5
Pak3	8107.437	-1.284359957	0.171241	-7.50032	6.37E-14	9.21E-12	TRUE	Pak3
Gabre	94.60576	-1.819750099	0.380903	-4.77747	1.78E-06	5.32E-05	TRUE	Gabre
Gabrq	195.9368	-1.479747647	0.360426	-4.10555	4.03E-05	0.000811	TRUE	Gabrq
Zfp92	72.38685	-1.592210743	0.379181	-4.19908	2.68E-05	0.000574	TRUE	Zfp92
Plp1	887.7217	-1.586645048	0.269994	-5.87658	4.19E-09	2.60E-07	TRUE	Plp1
Rnf128	30.56678	-2.58618739	0.577479	-4.47841	7.52E-06	0.000192	TRUE	Rnf128
Sfrp1	445.8766	-2.675809135	0.278728	-9.60009	7.99E-22	2.80E-19	TRUE	Sfrp1
Kcnu1	86.30955	-1.859822728	0.314372	-5.916	3.30E-09	2.13E-07	TRUE	Kcnu1
Nr3c2	36.16845	-2.289470646	0.586852	-3.90127	9.57E-05	0.00167	TRUE	Nr3c2
Cbln1	4658.016	-2.505587861	0.179126	-13.9879	1.85E-44	4.78E-41	TRUE	Cbln1
Sall1	414.5815	-2.10259717	0.350627	-5.99668	2.01E-09	1.36E-07	TRUE	Sall1
Pmfbp1	227.7819	-3.697411466	0.372902	-9.91523	3.57E-23	1.32E-20	TRUE	Pmfbp1
Mt3	382.7468	-2.855929137	0.836535	-3.414	0.00064	0.008184	TRUE	Mt3
Mt1	56.17488	-3.622630528	0.622548	-5.81904	5.92E-09	3.54E-07	TRUE	Mt1
Cx3cl1	554.4225	-1.335186209	0.193254	-6.90898	4.88E-12	5.24E-10	TRUE	Cx3cl1
Gse1	1323.308	-1.825716989	0.302826	-6.02894	1.65E-09	1.14E-07	TRUE	Gse1
Cdh13	588.9839	-1.667828861	0.274971	-6.06547	1.32E-09	9.26E-08	TRUE	Cdh13
Bean1	184.2076	-1.932258614	0.363766	-5.31182	1.09E-07	4.46E-06	TRUE	Bean1
Vstm5	155.7259	-1.795882952	0.366782	-4.89633	9.76E-07	3.16E-05	TRUE	Vstm5
Bmper	360.334	-1.188610583	0.287351	-4.13644	3.53E-05	0.000725	TRUE	Bmper
Usp2	210.5785	-1.488096059	0.430885	-3.45358	0.000553	0.00721	TRUE	Usp2
Grik4	880.8197	-1.175927616	0.30153	-3.89987	9.62E-05	0.001678	TRUE	Grik4
Rab27a	74.23229	-2.373633364	0.662909	-3.58063	0.000343	0.004896	TRUE	Rab27a
Drd2	141.9846	-1.411559966	0.414015	-3.40944	0.000651	0.008276	TRUE	Drd2
Tent5a	336.9504	-1.101283924	0.289985	-3.79773	0.000146	0.002386	TRUE	Tent5a
Htr3a	71.95802	-1.816457793	0.382999	-4.74272	2.11E-06	6.25E-05	TRUE	Htr3a
Celf6	1279.408	-1.012249062	0.215651	-4.69391	2.68E-06	7.68E-05	TRUE	Celf6
Stra6	180.9594	-1.261784355	0.256545	-4.91838	8.73E-07	2.89E-05	TRUE	Stra6
Gsta4	229.0847	-2.094544435	0.412612	-5.07631	3.85E-07	1.38E-05	TRUE	Gsta4
Plscr4	39.44065	-1.915577982	0.497788	-3.84818	0.000119	0.002007	TRUE	Plscr4
Ephb1	2034.117	-2.235142826	0.209915	-10.6478	1.78E-26	9.98E-24	TRUE	Ephb1
Mst1r	105.1037	-1.550162022	0.454707	-3.40915	0.000652	0.00828	TRUE	Mst1r
Pon2	329.2257	-1.116087537	0.291087	-3.8342	0.000126	0.002106	TRUE	Pon2
Ablim3	1097.937	-2.107936845	0.282767	-7.45467	9.01E-14	1.26E-11	TRUE	Ablim3
Arap1	283.9122	-1.484896523	0.340447	-4.3616	1.29E-05	0.000305	TRUE	Arap1
Rims3	2184.801	-1.456590526	0.251462	-5.79249	6.93E-09	4.02E-07	TRUE	Rims3
Styk1	75.93596	-2.174566656	0.473101	-4.59641	4.30E-06	0.000117	TRUE	Styk1
Itgbl1	91.55147	-1.690213568	0.377375	-4.47887	7.50E-06	0.000192	TRUE	Itgbl1
Resp18	365.7542	-1.169624773	0.281737	-4.15147	3.30E-05	0.000686	TRUE	Resp18
Mgll	214.0425	-1.643822281	0.347363	-4.73229	2.22E-06	6.50E-05	TRUE	Mgll
Ptprm	339.8567	-1.453148318	0.312359	-4.65217	3.28E-06	9.16E-05	TRUE	Ptprm
Tnxb	64.226	-2.703395271	0.627776	-4.3063	1.66E-05	0.000379	TRUE	Tnxb
Plppr5	82.9565	-1.900937839	0.389191	-4.88433	1.04E-06	3.34E-05	TRUE	Plppr5
Adamts15	122.4922	-1.759118891	0.398453	-4.41487	1.01E-05	0.000248	TRUE	Adamts15
Ndn	23905.92	-1.48690654	0.224333	-6.62811	3.40E-11	3.16E-09	TRUE	Ndn
Lgi3	38.95917	-2.192871955	0.545174	-4.02234	5.76E-05	0.0011	TRUE	Lgi3
Ucp2	1005.898	-1.167148875	0.338789	-3.44507	0.000571	0.007404	TRUE	Ucp2
Gucy1a1	201.0342	-1.664428894	0.380539	-4.37388	1.22E-05	0.00029	TRUE	Gucy1a1
Slc16a2	543.17	-1.229918766	0.312792	-3.93206	8.42E-05	0.001508	TRUE	Slc16a2
Phka1	266.9903	-1.655068168	0.353112	-4.68709	2.77E-06	7.88E-05	TRUE	Phka1
Scn11a	16.28199	-3.437457072	0.926761	-3.70911	0.000208	0.003194	TRUE	Scn11a
Tmem63c	299.2393	-1.634691434	0.321907	-5.07815	3.81E-07	1.37E-05	TRUE	Tmem63c
Faah	308.9977	-2.348878181	0.34729	-6.76344	1.35E-11	1.37E-09	TRUE	Faah
Rhov	223.1837	-1.122369576	0.313262	-3.58285	0.00034	0.004865	TRUE	Rhov
Cpne2	455.2825	-1.093474648	0.205658	-5.31695	1.06E-07	4.38E-06	TRUE	Cpne2
Neurl1b	540.3866	-1.122078283	0.25067	-4.47632	7.59E-06	0.000193	TRUE	Neurl1b
Inpp5j	89.27817	-2.56105321	0.511111	-5.01075	5.42E-07	1.88E-05	TRUE	Inpp5j
Pik3ip1	588.2136	-1.072527451	0.21223	-5.05361	4.34E-07	1.53E-05	TRUE	Pik3ip1
Pcdh11x	191.9942	-2.805632156	0.341137	-8.22436	1.96E-16	3.87E-14	TRUE	Pcdh11x
Dusp5	61.94971	-1.582749783	0.375483	-4.21523	2.50E-05	0.000539	TRUE	Dusp5
Tle2	849.1612	-2.252252632	0.387693	-5.80937	6.27E-09	3.70E-07	TRUE	Tle2
Cpne7	129.4295	-4.029417956	0.534891	-7.53316	4.95E-14	7.32E-12	TRUE	Cpne7
Parm1	238.1812	-2.049572286	0.459391	-4.4615	8.14E-06	0.000204	TRUE	Parm1
Egln3	89.33946	-2.534518997	0.522123	-4.85425	1.21E-06	3.82E-05	TRUE	Egln3
Brinp3	114.3677	-2.622231301	0.376424	-6.96616	3.26E-12	3.68E-10	TRUE	Brinp3

Pcdh17	1312.381	-1.571393527	0.34322	-4.57838	4.69E-06	0.000126	TRUE	Pcdh17
Iqcg	112.1855	-1.642569949	0.42893	-3.82946	0.000128	0.002142	TRUE	Iqcg
Misp	136.8138	-1.431272368	0.322197	-4.44222	8.90E-06	0.000221	TRUE	Misp
Syt1	4166.901	-1.350377841	0.274679	-4.91621	8.82E-07	2.92E-05	TRUE	Syt1
Prr5	251.7527	-1.014009625	0.260206	-3.89696	9.74E-05	0.001693	TRUE	Prr5
Lmo1	357.99	-1.022173232	0.274637	-3.7219	0.000198	0.003056	TRUE	Lmo1
Prickle1	298.2185	-1.700543687	0.489586	-3.47343	0.000514	0.006805	TRUE	Prickle1
Sostdc1	21.40848	-4.243459966	1.045757	-4.05779	4.95E-05	0.000964	TRUE	Sostdc1
Macrod1	75.66703	-1.75660179	0.494011	-3.5558	0.000377	0.005295	TRUE	Macrod1
Zdhhc23	66.37451	-1.901591918	0.506697	-3.75292	0.000175	0.002762	TRUE	Zdhhc23
Tmem255a	400.39	-1.837989726	0.283051	-6.49348	8.39E-11	7.39E-09	TRUE	Tmem255a
Ppfibp2	49.79057	-1.674435579	0.432008	-3.87594	0.000106	0.001815	TRUE	Ppfibp2
Fxyd7	275.7947	-3.502970612	0.347853	-10.0702	7.48E-24	3.06E-21	TRUE	Fxyd7
Alx1	21.7599	-3.777011188	0.848008	-4.45398	8.43E-06	0.000211	TRUE	Alx1
Etl4	169.8383	-3.229717315	0.933163	-3.46104	0.000538	0.007066	TRUE	Etl4
Zcchc12	4002.932	-1.196317184	0.260132	-4.59888	4.25E-06	0.000116	TRUE	Zcchc12
Fam135b	178.1935	-1.8810303	0.503766	-3.73394	0.000189	0.00294	TRUE	Fam135b
Wnt4	143.3092	-2.298695666	0.450899	-5.09803	3.43E-07	1.25E-05	TRUE	Wnt4
Kirrel2	41.4404	-2.15737848	0.623369	-3.46084	0.000539	0.007066	TRUE	Kirrel2
Frem2	169.6145	-2.690284546	0.571334	-4.70877	2.49E-06	7.19E-05	TRUE	Frem2
Islr	94.75177	-1.789554265	0.296843	-6.02863	1.65E-09	1.14E-07	TRUE	Islr
Spry1	294.0853	-1.063752845	0.232439	-4.57648	4.73E-06	0.000126	TRUE	Spry1
Map4k1	137.1614	-1.160608557	0.279486	-4.15266	3.29E-05	0.000684	TRUE	Map4k1
Arhgef4	563.4314	-1.382405877	0.261057	-5.29542	1.19E-07	4.83E-06	TRUE	Arhgef4
Kcnh3	32.49333	-3.917209031	0.639118	-6.12909	8.84E-10	6.49E-08	TRUE	Kcnh3
Atoh8	59.31342	-2.058400976	0.52049	-3.95474	7.66E-05	0.001392	TRUE	Atoh8
Kcnk2	1248.787	-1.257015999	0.265448	-4.73545	2.19E-06	6.44E-05	TRUE	Kcnk2
Armc3	23.28271	-2.586928573	0.632198	-4.09196	4.28E-05	0.000848	TRUE	Armc3
Ccdc33	40.01393	-1.735695037	0.447275	-3.8806	0.000104	0.00179	TRUE	Ccdc33
Ppp1r16b	234.2756	-1.437311452	0.317227	-4.53086	5.87E-06	0.000153	TRUE	Ppp1r16b
Vstm2l	1145.243	-1.027525535	0.197918	-5.19168	2.08E-07	7.97E-06	TRUE	Vstm2l
Fgd3	849.617	-1.275959296	0.223097	-5.71929	1.07E-08	5.94E-07	TRUE	Fgd3
Rara	625.3008	-1.072186224	0.226412	-4.73555	2.18E-06	6.44E-05	TRUE	Rara
Nod1	74.42339	-1.145286615	0.340255	-3.36596	0.000763	0.009403	TRUE	Nod1
Fkbp14	238.6444	-1.189395569	0.309654	-3.84105	0.000123	0.002055	TRUE	Fkbp14
AW551984	1675.969	-2.732054586	0.325417	-8.39555	4.64E-17	9.79E-15	TRUE	AW551984
Rbm24	205.8092	-1.016253882	0.230574	-4.40749	1.05E-05	0.000254	TRUE	Rbm24
Spon1	1033.064	-3.349405976	0.313205	-10.694	1.09E-26	6.24E-24	TRUE	Spon1
Mical2	164.4114	-2.302106061	0.37721	-6.10299	1.04E-09	7.46E-08	TRUE	Mical2
Satb2	421.0592	-2.742408799	0.319853	-8.57397	1.00E-17	2.23E-15	TRUE	Satb2
Ankrd35	205.2587	-2.189647573	0.307798	-7.11392	1.13E-12	1.36E-10	TRUE	Ankrd35
Txnip	939.3324	-1.113153202	0.256983	-4.33162	1.48E-05	0.000345	TRUE	Txnip
Rgs4	1032.344	-2.603823989	0.35624	-7.30918	2.69E-13	3.54E-11	TRUE	Rgs4
Susd1	65.96541	-1.945004162	0.505519	-3.84754	0.000119	0.002011	TRUE	Susd1
Vill	70.80885	-2.156155471	0.541476	-3.98199	6.83E-05	0.001271	TRUE	Vill
Nipal2	106.9615	-1.831882222	0.356142	-5.14369	2.69E-07	1.00E-05	TRUE	Nipal2
Tsga13	41.02695	-1.949363962	0.526259	-3.70419	0.000212	0.003239	TRUE	Tsga13
Prune2	241.9207	-1.664189256	0.311628	-5.34031	9.28E-08	3.93E-06	TRUE	Prune2
Tekt5	20.93871	-6.171841044	1.218449	-5.06532	4.08E-07	1.45E-05	TRUE	Tekt5
Rftn1	1264.416	-1.122353439	0.317114	-3.53927	0.000401	0.005574	TRUE	Rftn1
Cdh6	1210.461	-2.096141598	0.376999	-5.56007	2.70E-08	1.32E-06	TRUE	Cdh6
Prss23	44.58373	-1.446039383	0.431138	-3.35401	0.000797	0.009738	TRUE	Prss23
Atp8b1	42.7387	-3.465942808	0.785455	-4.41265	1.02E-05	0.00025	TRUE	Atp8b1
Rsph4a	15.21052	-3.367552549	0.980568	-3.43429	0.000594	0.007671	TRUE	Rsph4a
Grin3a	386.7379	-1.605082233	0.261866	-6.1294	8.82E-10	6.49E-08	TRUE	Grin3a
Ccdc151	240.8937	-1.555517931	0.351044	-4.43112	9.37E-06	0.000231	TRUE	Ccdc151
Sdk1	498.5199	-1.055339119	0.218321	-4.83389	1.34E-06	4.17E-05	TRUE	Sdk1
Gpr37	126.773	-2.804792711	0.440605	-6.36577	1.94E-10	1.62E-08	TRUE	Gpr37
Negr1	2382.705	-1.387769953	0.267701	-5.18403	2.17E-07	8.25E-06	TRUE	Negr1
Prdm13	101.3289	-4.095665657	1.107811	-3.69708	0.000218	0.003314	TRUE	Prdm13
Lrfn2	205.9278	-2.104056097	0.238605	-8.81817	1.16E-18	2.87E-16	TRUE	Lrfn2
Pvr	113.3652	-2.539209012	0.451518	-5.62372	1.87E-08	9.53E-07	TRUE	Pvr
Pitpnm3	168.7476	-1.892785354	0.442825	-4.27434	1.92E-05	0.000429	TRUE	Pitpnm3
Kazn	1643.872	-1.539012522	0.202139	-7.61362	2.67E-14	4.06E-12	TRUE	Kazn
Ildr2	922.398	-1.295322594	0.278646	-4.64863	3.34E-06	9.31E-05	TRUE	Ildr2
Plekhg1	461.4195	-1.319512698	0.39367	-3.35182	0.000803	0.009798	TRUE	Plekhg1
Col16a1	114.2271	-1.963372516	0.478503	-4.10315	4.08E-05	0.000817	TRUE	Col16a1
Reps2	506.9776	-1.312141675	0.227707	-5.76242	8.29E-09	4.78E-07	TRUE	Reps2
Dlk1	10010.67	-3.456698394	0.315132	-10.969	5.38E-28	3.48E-25	TRUE	Dlk1
Begain	1912.278	-1.782803353	0.229035	-7.78397	7.03E-15	1.16E-12	TRUE	Begain
Kcnd3	752.2922	-1.550783874	0.31672	-4.89639	9.76E-07	3.16E-05	TRUE	Kcnd3
Trim62	705.9167	-1.386531141	0.249474	-5.55783	2.73E-08	1.33E-06	TRUE	Trim62

Fzd7	280.5232	-2.080458755	0.334522	-6.2192	5.00E-10	3.90E-08	TRUE	Fzd7
Grid1	364.3238	-1.36472493	0.33446	-4.08038	4.50E-05	0.000886	TRUE	Grid1
Nbl1	246.3892	-1.800064678	0.451479	-3.98704	6.69E-05	0.001249	TRUE	Nbl1
Pla2g2d	5.575164	-6.058410914	1.739274	-3.4833	0.000495	0.006614	TRUE	Pla2g2d
Nkx6-2	31.18059	-4.193667516	1.072574	-3.90991	9.23E-05	0.001622	TRUE	Nkx6-2
Ak7	66.2043	-1.468187547	0.396661	-3.70137	0.000214	0.003268	TRUE	Ak7
Shtn1	2662.787	-3.175401663	0.35706	-8.89319	5.94E-19	1.58E-16	TRUE	Shtn1
Ptchd1	59.37526	-1.745374809	0.499737	-3.49259	0.000478	0.006425	TRUE	Ptchd1
Mpped1	295.1153	-1.919062362	0.285413	-6.72382	1.77E-11	1.74E-09	TRUE	Mpped1
Myrip	174.2906	-2.322851436	0.482793	-4.81128	1.50E-06	4.60E-05	TRUE	Myrip
Gck	544.714	-2.844436835	0.391833	-7.25932	3.89E-13	4.97E-11	TRUE	Gck
Abca8a	5.649914	-6.119684224	1.763857	-3.46949	0.000521	0.006889	TRUE	Abca8a
Ptpre	151.5957	-1.917509205	0.328056	-5.84506	5.06E-09	3.12E-07	TRUE	Ptpre
Dlx1	7463.39	-1.881349167	0.306896	-6.13024	8.77E-10	6.49E-08	TRUE	Dlx1
S100a10	230.1762	-2.092380612	0.427265	-4.89715	9.72E-07	3.16E-05	TRUE	S100a10
Csdc2	2699.794	-1.252857556	0.223147	-5.61449	1.97E-08	9.98E-07	TRUE	Csdc2
Fam92b	107.946	-2.38783376	0.429076	-5.56506	2.62E-08	1.29E-06	TRUE	Fam92b
Dlgap3	2208.275	-1.341204203	0.201546	-6.65458	2.84E-11	2.66E-09	TRUE	Dlgap3
Crtac1	385.6835	-1.919460714	0.405585	-4.73257	2.22E-06	6.50E-05	TRUE	Crtac1
Golga7b	1081.352	-1.015341154	0.222981	-4.55349	5.28E-06	0.000139	TRUE	Golga7b
Asb4	837.7894	-1.850345139	0.231158	-8.00469	1.20E-15	2.14E-13	TRUE	Asb4
Pbxip1	961.4322	-1.627384341	0.364063	-4.47007	7.82E-06	0.000198	TRUE	Pbxip1
Dusp15	127.1626	-1.725366002	0.289352	-5.96285	2.48E-09	1.63E-07	TRUE	Dusp15
Lrrtm3	154.602	-1.520554031	0.283849	-5.35691	8.47E-08	3.64E-06	TRUE	Lrrtm3
Onecut1	55.32641	-2.030744699	0.517248	-3.92606	8.63E-05	0.001541	TRUE	Onecut1
Synpo	53.39036	-2.355931545	0.530824	-4.43825	9.07E-06	0.000224	TRUE	Synpo
Lor	229.7856	-1.548057765	0.265966	-5.8205	5.87E-09	3.53E-07	TRUE	Lor
Tmem130	7072.312	-1.143101357	0.180716	-6.32541	2.53E-10	2.05E-08	TRUE	Tmem130
Elfn2	474.6642	-2.080023645	0.268502	-7.74677	9.43E-15	1.54E-12	TRUE	Elfn2
Clrn1	28.18685	-2.635093479	0.566423	-4.65217	3.28E-06	9.16E-05	TRUE	Clrn1
Chil5	12.12109	-6.22168921	1.586363	-3.92198	8.78E-05	0.00156	TRUE	Chil5
Ccdc60	86.53186	-2.49329208	0.570585	-4.36971	1.24E-05	0.000295	TRUE	Ccdc60
00052L18I	142.9857	-1.184129856	0.309312	-3.82826	0.000129	0.002151	TRUE	2900052L18Rik
Tafa2	817.579	-2.572178897	0.293126	-8.775	1.71E-18	4.03E-16	TRUE	Tafa2
Foxo1	269.6032	-1.227507373	0.262347	-4.67894	2.88E-06	8.16E-05	TRUE	Foxo1
Nkx2-3	394.3354	-4.832965014	1.17483	-4.11376	3.89E-05	0.000788	TRUE	Nkx2-3
Snhg11	7422.524	-1.071004558	0.235509	-4.54761	5.43E-06	0.000142	TRUE	Snhg11
BC024139	790.7999	-3.061746698	0.308043	-9.93936	2.81E-23	1.08E-20	TRUE	BC024139
Shisa2	129.1954	-2.094245549	0.377144	-5.55291	2.81E-08	1.36E-06	TRUE	Shisa2
Garem2	1396.951	-1.141773954	0.287315	-3.97395	7.07E-05	0.001304	TRUE	Garem2
Zbtb7c	435.6307	-1.025606151	0.245821	-4.17217	3.02E-05	0.000636	TRUE	Zbtb7c
Plppr4	344.201	-2.621370534	0.313838	-8.35263	6.68E-17	1.40E-14	TRUE	Plppr4
Dok7	109.0522	-1.30296476	0.368775	-3.53322	0.000411	0.005679	TRUE	Dok7
Syt16	1017.701	-1.227391681	0.291978	-4.20371	2.63E-05	0.000564	TRUE	Syt16
Sstr3	27.93225	-2.398686118	0.596387	-4.02203	5.77E-05	0.001101	TRUE	Sstr3
Wdr72	10.65191	-4.165010705	1.091255	-3.81672	0.000135	0.002232	TRUE	Wdr72
Fzd5	491.9092	-5.093153607	0.90802	-5.60907	2.03E-08	1.02E-06	TRUE	Fzd5
Lingo2	175.9648	-1.205071484	0.321532	-3.7479	0.000178	0.002811	TRUE	Lingo2
Sox3	588.0332	-2.625578672	0.309088	-8.49459	1.99E-17	4.24E-15	TRUE	Sox3
Shroom2	957.926	-1.306307484	0.342112	-3.81836	0.000134	0.002223	TRUE	Shroom2
Adra2c	45.47187	-3.027156109	0.643291	-4.70574	2.53E-06	7.29E-05	TRUE	Adra2c
Onecut3	338.9736	-3.428485371	0.795448	-4.31013	1.63E-05	0.000374	TRUE	Onecut3
Penk	35.52469	-3.271511827	0.647796	-5.05022	4.41E-07	1.55E-05	TRUE	Penk
Smtnl2	92.55325	-2.078030191	0.589615	-3.52438	0.000424	0.005822	TRUE	Smtnl2
Npas4	367.9421	-1.367725949	0.260587	-5.24863	1.53E-07	6.08E-06	TRUE	Npas4
C2cd4c	157.1517	-1.617978773	0.316512	-5.1119	3.19E-07	1.17E-05	TRUE	C2cd4c
Hpcal4	398.4206	-1.954313565	0.36684	-5.32743	9.96E-08	4.20E-06	TRUE	Hpcal4
Ar	25.52417	-4.568134476	0.922102	-4.95405	7.27E-07	2.46E-05	TRUE	Ar
Hrk	179.3792	-1.392889924	0.339484	-4.10296	4.08E-05	0.000817	TRUE	Hrk
Vwa5b2	429.3572	-1.225647208	0.312486	-3.92224	8.77E-05	0.00156	TRUE	Vwa5b2
Krt1	158.5469	-1.075221896	0.256758	-4.18769	2.82E-05	0.000599	TRUE	Krt1
Dmrta2	356.0665	-1.756233975	0.274356	-6.40129	1.54E-10	1.30E-08	TRUE	Dmrta2
Cyp4x1	109.1419	-1.870751601	0.3618	-5.17068	2.33E-07	8.81E-06	TRUE	Cyp4x1
Cdh19	7.275027	-6.467042048	1.630582	-3.96609	7.31E-05	0.001339	TRUE	Cdh19
Sfn	118.2092	-1.173487974	0.333758	-3.51598	0.000438	0.00599	TRUE	Sfn
Ctdspl	636.8882	-1.076200682	0.257208	-4.18416	2.86E-05	0.000606	TRUE	Ctdspl
Dlk2	509.3017	-1.778001686	0.237889	-7.47407	7.78E-14	1.11E-11	TRUE	Dlk2
Mroh7	65.82933	-2.109387314	0.411285	-5.12877	2.92E-07	1.08E-05	TRUE	Mroh7
Baiap3	659.5441	-3.372201963	0.307647	-10.9613	5.87E-28	3.57E-25	TRUE	Baiap3
Fgfbp3	244.2564	-1.304028041	0.326466	-3.99437	6.49E-05	0.001219	TRUE	Fgfbp3
Tshz2	3002.108	-1.162583902	0.159287	-7.29866	2.91E-13	3.81E-11	TRUE	Tshz2
Sox1ot	3712.589	-3.559380462	0.292911	-12.1517	5.62E-34	6.12E-31	TRUE	Sox1ot

Eno4	89.14654	-3.973237302	0.591382	-6.71857	1.84E-11	1.79E-09	TRUE	Eno4
Col6a3	81.61095	-3.318170304	0.60973	-5.44203	5.27E-08	2.38E-06	TRUE	Col6a3
Nwd1	254.6531	-2.048999647	0.42843	-4.78258	1.73E-06	5.24E-05	TRUE	Nwd1
Bend7	128.7033	-1.882194374	0.435136	-4.32553	1.52E-05	0.000352	TRUE	Bend7
Amigo2	429.7917	-1.592825512	0.365478	-4.3582	1.31E-05	0.000309	TRUE	Amigo2
Igf2	163.331	-3.337483742	0.611755	-5.45559	4.88E-08	2.21E-06	TRUE	Igf2
Myof	24.72933	-3.213456498	0.894409	-3.59283	0.000327	0.004718	TRUE	Myof
Krt90	6.167115	-6.241380112	1.863795	-3.34875	0.000812	0.009874	TRUE	Krt90
30114P18I	146.6353	-2.068163183	0.617626	-3.34857	0.000812	0.009874	TRUE	E130114P18Rik
Dact2	28.93589	-2.554023717	0.678659	-3.76334	0.000168	0.002676	TRUE	Dact2
Rimkla	689.5344	-1.203080533	0.19466	-6.18041	6.39E-10	4.88E-08	TRUE	Rimkla
Efna5	3120.192	-1.139708975	0.164529	-6.92712	4.30E-12	4.70E-10	TRUE	Efna5
Fam183b	45.22304	-3.356660909	0.589547	-5.69363	1.24E-08	6.77E-07	TRUE	Fam183b
Tex50	27.74604	-2.413458716	0.604243	-3.99418	6.49E-05	0.001219	TRUE	Tex50
Kcnk3	185.9796	-1.297291345	0.284293	-4.56322	5.04E-06	0.000134	TRUE	Kcnk3
Chchd10	86.10716	-2.305415085	0.511505	-4.50712	6.57E-06	0.00017	TRUE	Chchd10
Htr1b	84.11306	-2.186187587	0.56439	-3.87354	0.000107	0.001829	TRUE	Htr1b
Tmie	47.63536	-2.021091492	0.489949	-4.1251	3.71E-05	0.000756	TRUE	Tmie
Cfap46	133.5862	-1.265613714	0.342152	-3.69898	0.000216	0.003294	TRUE	Cfap46
Grm5	365.1978	-1.067159311	0.299299	-3.56553	0.000363	0.005127	TRUE	Grm5
C1ql3	19.46839	-4.087868012	1.00729	-4.05828	4.94E-05	0.000964	TRUE	C1ql3
Catsperg1	238.3965	-1.645126124	0.269004	-6.11561	9.62E-10	6.94E-08	TRUE	Catsperg1
Hmx2	297.0291	-7.782603406	1.888559	-4.12092	3.77E-05	0.000766	TRUE	Hmx2
Kcnk12	49.2334	-3.672537502	0.638217	-5.75438	8.70E-09	4.98E-07	TRUE	Kcnk12
Lgr4	656.5224	-1.727926405	0.407743	-4.23779	2.26E-05	0.000495	TRUE	Lgr4
Pla2g4e	45.63791	-1.498853266	0.415533	-3.60706	0.00031	0.0045	TRUE	Pla2g4e
Lypd6	59.05709	-2.862566562	0.465409	-6.15064	7.72E-10	5.81E-08	TRUE	Lypd6
Trhde	411.3088	-2.304479798	0.443682	-5.19399	2.06E-07	7.92E-06	TRUE	Trhde
Scg2	977.4283	-2.85263163	0.728079	-3.91802	8.93E-05	0.001577	TRUE	Scg2
Minar2	2846.616	-1.088616584	0.176694	-6.16102	7.23E-10	5.48E-08	TRUE	Minar2
Gja1	387.2286	-1.566044451	0.332963	-4.70336	2.56E-06	7.36E-05	TRUE	Gja1
P4ha3	114.2618	-2.069023394	0.400848	-5.16162	2.45E-07	9.20E-06	TRUE	P4ha3
Cited1	153.0701	-1.596970199	0.274907	-5.80913	6.28E-09	3.70E-07	TRUE	Cited1
Pcdh19	2059.449	-1.787282884	0.248617	-7.1889	6.53E-13	8.14E-11	TRUE	Pcdh19
00026A02I	435.0138	-1.755209355	0.327111	-5.36579	8.06E-08	3.47E-06	TRUE	2900026A02Rik
Fhad1	62.22639	-1.860924565	0.490149	-3.79665	0.000147	0.002394	TRUE	Fhad1
Usp29	2214.039	-1.497520166	0.276359	-5.41875	6.00E-08	2.64E-06	TRUE	Usp29
Lrrc3	478.5711	-1.089533024	0.255707	-4.26087	2.04E-05	0.000451	TRUE	Lrrc3
Rspo2	51.78153	-3.810559164	0.851792	-4.47358	7.69E-06	0.000195	TRUE	Rspo2
Gpr17	65.58585	-2.379551578	0.534821	-4.44925	8.62E-06	0.000215	TRUE	Gpr17
Il1rapl1	59.92286	-1.59880274	0.381678	-4.18888	2.80E-05	0.000597	TRUE	Il1rapl1
Actn2	48.63968	-2.018775243	0.505753	-3.99162	6.56E-05	0.001229	TRUE	Actn2
Adarb2	219.8757	-1.418106739	0.402283	-3.52515	0.000423	0.005817	TRUE	Adarb2
Isoc2b	66.6367	-1.47856689	0.388721	-3.80367	0.000143	0.002344	TRUE	Isoc2b
Pcdh15	352.2556	-1.536254407	0.228874	-6.71221	1.92E-11	1.84E-09	TRUE	Pcdh15
Gm9885	43.74703	-1.44526862	0.407589	-3.5459	0.000391	0.005454	TRUE	Gm9885
Sh2d6	47.23679	-1.685366106	0.461953	-3.64835	0.000264	0.003908	TRUE	Sh2d6
Gsx1	298.3822	-2.98297535	0.350833	-8.50255	1.85E-17	4.00E-15	TRUE	Gsx1
Ptprt	720.0326	-1.514579019	0.367162	-4.1251	3.71E-05	0.000756	TRUE	Ptprt
Cdh22	1369.477	-2.579134	0.210754	-12.2377	1.96E-34	2.25E-31	TRUE	Cdh22
Ebf4	792.1647	-1.150910607	0.236579	-4.8648	1.15E-06	3.65E-05	TRUE	Ebf4
Nebl	298.1097	-1.738361855	0.313256	-5.54933	2.87E-08	1.38E-06	TRUE	Nebl
Tmem179	2073.015	-1.934307684	0.241277	-8.01696	1.08E-15	1.95E-13	TRUE	Tmem179
Nkx2-4	354.2567	-11.09746877	2.108655	-5.26282	1.42E-07	5.68E-06	TRUE	Nkx2-4
Bsx	192.9909	-6.482037442	1.652121	-3.92346	8.73E-05	0.001556	TRUE	Bsx
Vsnl1	85.51613	-1.896619548	0.516836	-3.66967	0.000243	0.003635	TRUE	Vsnl1
Smim10l2a	367.0245	-1.287270863	0.215773	-5.96586	2.43E-09	1.60E-07	TRUE	Smim10l2a
Tmem158	407.3393	-1.496541352	0.271129	-5.51967	3.40E-08	1.60E-06	TRUE	Tmem158
31415C17I	55.20439	-1.54011097	0.388111	-3.96822	7.24E-05	0.00133	TRUE	4931415C17Rik
30416C01I	40.35149	-2.63523652	0.649225	-4.05905	4.93E-05	0.000962	TRUE	5330416C01Rik
Sec14l3	14.96721	-4.600805246	1.084312	-4.24306	2.20E-05	0.000485	TRUE	Sec14l3
Lrtm2	183.4442	-1.991558591	0.352859	-5.64406	1.66E-08	8.59E-07	TRUE	Lrtm2
Gabrg3	165.6341	-2.482287818	0.436903	-5.68155	1.33E-08	7.16E-07	TRUE	Gabrg3
Gabra5	181.9944	-3.089942862	0.456688	-6.76598	1.32E-11	1.36E-09	TRUE	Gabra5
Wdr86	57.0817	-2.167945961	0.577633	-3.75315	0.000175	0.002761	TRUE	Wdr86
Tns1	585.1609	-1.048095042	0.177648	-5.89984	3.64E-09	2.30E-07	TRUE	Tns1
Nell1	140.0392	-2.253855399	0.482679	-4.66947	3.02E-06	8.50E-05	TRUE	Nell1
Maf	751.851	-2.724248889	0.724851	-3.75836	0.000171	0.002715	TRUE	Maf
Zcchc24	310.4653	-1.529518778	0.421929	-3.62506	0.000289	0.004225	TRUE	Zcchc24
Ghr	130.9453	-1.600018126	0.441802	-3.62157	0.000293	0.004274	TRUE	Ghr
Tcf7l1	341.2388	-1.952639449	0.326406	-5.98224	2.20E-09	1.46E-07	TRUE	Tcf7l1
Dnaaf3	92.98329	-2.135736104	0.521653	-4.09417	4.24E-05	0.000842	TRUE	Dnaaf3

00034H15I	40.0991	-1.844921307	0.506674	-3.64124	0.000271	0.004003	TRUE	1700034H15Rik
Clca3a1	19.68681	-3.470053172	0.805041	-4.3104	1.63E-05	0.000374	TRUE	Clca3a1
Car10	453.3988	-1.782180204	0.369072	-4.82882	1.37E-06	4.26E-05	TRUE	Car10
Sertm1	105.6557	-1.91156205	0.530493	-3.60337	0.000314	0.004556	TRUE	Sertm1
Gpr50	313.5386	-6.623761255	0.466829	-14.1888	1.07E-45	3.71E-42	TRUE	Gpr50
Adap1	707.2023	-2.0931307	0.270186	-7.74699	9.41E-15	1.54E-12	TRUE	Adap1
Slit3	143.387	-2.215116534	0.45106	-4.91092	9.07E-07	2.98E-05	TRUE	Slit3
Med12l	237.3109	-1.514092903	0.438276	-3.45466	0.000551	0.007191	TRUE	Med12l
Nbeal2	718.5927	-1.0951915	0.210942	-5.1919	2.08E-07	7.97E-06	TRUE	Nbeal2
Grm7	132.3495	-1.498550019	0.446136	-3.35895	0.000782	0.009598	TRUE	Grm7
Gulp1	132.3526	-1.605135634	0.407445	-3.93951	8.16E-05	0.001468	TRUE	Gulp1
Magel2	7662.918	-2.460239873	0.245099	-10.0377	1.04E-23	4.06E-21	TRUE	Magel2
Slc35f3	24.25774	-3.590813176	0.870354	-4.12569	3.70E-05	0.000755	TRUE	Slc35f3
Arhgap24	92.54252	-1.156221868	0.313625	-3.68664	0.000227	0.003433	TRUE	Arhgap24
Lmcd1	51.20294	-2.811100368	0.678766	-4.14149	3.45E-05	0.000711	TRUE	Lmcd1
330018L16I	34.28255	-2.568508943	0.612128	-4.19603	2.72E-05	0.000581	TRUE	A830018L16Rik
Cacnb2	208.6936	-1.109655093	0.329747	-3.36517	0.000765	0.009413	TRUE	Cacnb2
Gm10037	81.38811	-1.308040669	0.382756	-3.41743	0.000632	0.008097	TRUE	Gm10037
Tspan7	2662.033	-1.058757568	0.169491	-6.24668	4.19E-10	3.33E-08	TRUE	Tspan7
Qrfpr	11.67663	-3.565237607	1.032305	-3.45367	0.000553	0.00721	TRUE	Qrfpr
Panx2	446.7602	-1.15392646	0.197546	-5.84131	5.18E-09	3.18E-07	TRUE	Panx2
Anks1b	909.3271	-1.126966442	0.302356	-3.72728	0.000194	0.003005	TRUE	Anks1b
Gda	69.57372	-3.749448321	0.577539	-6.49212	8.46E-11	7.42E-09	TRUE	Gda
Kcnt1	532.7342	-1.078672818	0.255593	-4.22027	2.44E-05	0.00053	TRUE	Kcnt1
Ntrk3	2282.899	-1.138811567	0.254378	-4.47685	7.58E-06	0.000193	TRUE	Ntrk3
Skap2	69.16054	-1.998773637	0.357886	-5.58494	2.34E-08	1.16E-06	TRUE	Skap2
Ddn	7.635134	-6.507378808	1.637725	-3.97343	7.08E-05	0.001306	TRUE	Ddn
Nrsn2	670.9862	-1.777900927	0.191838	-9.26773	1.90E-20	5.96E-18	TRUE	Nrsn2
Nhs	394.9267	-1.058022349	0.188615	-5.60944	2.03E-08	1.02E-06	TRUE	Nhs
00035N22	62.86263	-3.711149772	0.639083	-5.807	6.36E-09	3.73E-07	TRUE	1500035N22Rik
Shisal2a	82.81964	-1.309180494	0.372719	-3.51251	0.000444	0.006048	TRUE	Shisal2a
Hydin	32.54237	-3.301147486	0.929734	-3.55064	0.000384	0.005374	TRUE	Hydin
Chpt1	289.3392	-1.15564274	0.267383	-4.32205	1.55E-05	0.000358	TRUE	Chpt1
Tmem26	53.7553	-1.793041261	0.50322	-3.56314	0.000366	0.005162	TRUE	Tmem26
Caln1	187.6476	-1.172934406	0.332589	-3.52667	0.000421	0.005787	TRUE	Caln1
Chst8	270.8327	-2.478887225	0.48662	-5.09409	3.50E-07	1.27E-05	TRUE	Chst8
Sntb1	30.27468	-3.117500632	0.844673	-3.69078	0.000224	0.003385	TRUE	Sntb1
Plaat3	44.52363	-2.919281134	0.622446	-4.69002	2.73E-06	7.79E-05	TRUE	Plaat3
Kcnd2	451.723	-1.3232494	0.389606	-3.39638	0.000683	0.008607	TRUE	Kcnd2
Lsamp	1332.033	-1.368533794	0.334249	-4.09435	4.23E-05	0.000842	TRUE	Lsamp
Sfmbt2	94.67483	-2.047014742	0.446104	-4.58865	4.46E-06	0.000121	TRUE	Sfmbt2
Sox21	219.1125	-1.35532212	0.341792	-3.96534	7.33E-05	0.001341	TRUE	Sox21
Tmem91	137.0509	-1.224147502	0.36393	-3.36369	0.000769	0.009453	TRUE	Tmem91
Unc13c	143.9971	-2.431271154	0.588478	-4.13146	3.60E-05	0.000739	TRUE	Unc13c
Trank1	3180.279	-2.066351285	0.307811	-6.71305	1.91E-11	1.83E-09	TRUE	Trank1
Erbb2	359.5657	-1.497426193	0.374497	-3.9985	6.37E-05	0.001204	TRUE	Erbb2
Otof	107.0567	-1.663740708	0.341256	-4.87534	1.09E-06	3.48E-05	TRUE	Otof
Sim2	134.7935	-2.204292323	0.327363	-6.73348	1.66E-11	1.64E-09	TRUE	Sim2
10204K13I	89.4542	-1.266471641	0.362542	-3.49331	0.000477	0.006412	TRUE	2010204K13Rik
Cyp26b1	37.53039	-2.793398124	0.552013	-5.06038	4.18E-07	1.49E-05	TRUE	Cyp26b1
Wscd2	491.1079	-1.000596158	0.190921	-5.2409	1.60E-07	6.30E-06	TRUE	Wscd2
Jazf1	394.5444	-1.059973146	0.276118	-3.83884	0.000124	0.00207	TRUE	Jazf1
Unc5d	505.3172	-2.139379603	0.299801	-7.136	9.61E-13	1.18E-10	TRUE	Unc5d
Klk8	55.23463	-1.859365354	0.502624	-3.69932	0.000216	0.003292	TRUE	Klk8
Paqr9	216.7266	-1.857640331	0.324038	-5.73279	9.88E-09	5.53E-07	TRUE	Paqr9
Gm16551	287.609	-3.999676693	0.279549	-14.3076	1.96E-46	8.12E-43	TRUE	Gm16551
Usp51	176.8961	-1.8023469	0.46304	-3.89242	9.92E-05	0.001716	TRUE	Usp51
Cbln4	241.261	-3.240883925	0.425525	-7.6162	2.61E-14	4.01E-12	TRUE	Cbln4
Gbx1	78.31921	-2.552141613	0.653759	-3.90379	9.47E-05	0.001654	TRUE	Gbx1
Nnat	149070.1	-1.079141844	0.213729	-5.04911	4.44E-07	1.56E-05	TRUE	Nnat
230006K03I	41.05659	-2.443650545	0.454828	-5.3727	7.76E-08	3.36E-06	TRUE	A230006K03Rik
Efcab1	372.2124	-1.920043454	0.247925	-7.74445	9.60E-15	1.55E-12	TRUE	Efcab1
Ube2u	11.92055	-5.384026124	1.393019	-3.86501	0.000111	0.001888	TRUE	Ube2u
Ahnak	418.335	-1.320353843	0.323773	-4.07802	4.54E-05	0.000894	TRUE	Ahnak
Fat1	1263.726	-1.50411327	0.266341	-5.64733	1.63E-08	8.53E-07	TRUE	Fat1
Hs3st3b1	161.9858	-3.370863281	0.994494	-3.38953	0.0007	0.008761	TRUE	Hs3st3b1
Fam217b	158.1117	-1.497735158	0.421443	-3.55383	0.00038	0.00532	TRUE	Fam217b
Etnk2	349.9932	-1.479648563	0.329944	-4.48455	7.31E-06	0.000187	TRUE	Etnk2
Cntnap5a	175.6452	-1.319873999	0.299304	-4.40981	1.03E-05	0.000252	TRUE	Cntnap5a
Gad1	2029.266	-1.351036353	0.2683	-5.03555	4.76E-07	1.66E-05	TRUE	Gad1
00086L19I	74.79253	-3.949651091	0.588239	-6.71437	1.89E-11	1.83E-09	TRUE	1700086L19Rik
Map3k5	114.6493	-2.663723647	0.381628	-6.97989	2.95E-12	3.36E-10	TRUE	Map3k5

Grid2	111.058	-1.7696605	0.349502	-5.06338	4.12E-07	1.47E-05	TRUE	Grid2
Fam189a2	211.3193	-1.939691637	0.342714	-5.6598	1.52E-08	7.98E-07	TRUE	Fam189a2
Rtl4	17.46374	-3.435907562	0.924447	-3.71672	0.000202	0.003109	TRUE	Rtl4
Cdr1os	328.0686	-1.287633641	0.359848	-3.57827	0.000346	0.004937	TRUE	Cdr1os
Fndc1	47.24365	-2.321888996	0.46733	-4.96842	6.75E-07	2.29E-05	TRUE	Fndc1
Zfp9	763.4565	-1.26482735	0.313	-4.04099	5.32E-05	0.001028	TRUE	Zfp9
Nup62cl	302.3673	-6.330274524	0.947945	-6.67789	2.42E-11	2.30E-09	TRUE	Nup62cl
Drc1	181.8966	-2.071487939	0.336207	-6.16134	7.21E-10	5.48E-08	TRUE	Drc1
H2-T-ps	42.56644	-4.681287111	0.889088	-5.26527	1.40E-07	5.62E-06	TRUE	H2-T-ps
Arhgdig	177.9349	-2.477837722	0.385655	-6.42501	1.32E-10	1.12E-08	TRUE	Arhgdig
AW146154	89.14551	-1.642314323	0.425276	-3.86176	0.000113	0.001907	TRUE	AW146154
Amy1	423.0362	-1.462592075	0.257502	-5.67991	1.35E-08	7.19E-07	TRUE	Amy1
30028M14	1003.567	-1.774598964	0.306155	-5.7964	6.78E-09	3.94E-07	TRUE	D930028M14Rik
Arhgap40	37.80339	-2.079100975	0.50431	-4.12266	3.75E-05	0.000762	TRUE	Arhgap40
33422C13I	125.4889	-1.541849729	0.357246	-4.31593	1.59E-05	0.000365	TRUE	4833422C13Rik
Ctxn2	32.21022	-2.510337058	0.518695	-4.83972	1.30E-06	4.07E-05	TRUE	Ctxn2
Pak6	1258.114	-1.934673755	0.202183	-9.56894	1.08E-21	3.66E-19	TRUE	Pak6
Lrrc55	388.4098	-1.22639805	0.342861	-3.57695	0.000348	0.004959	TRUE	Lrrc55
Rprm	811.9285	-1.787838284	0.314329	-5.68779	1.29E-08	6.94E-07	TRUE	Rprm
Gm10863	9.384343	-6.842670317	1.594967	-4.29016	1.79E-05	0.000404	TRUE	Gm10863
Dio3	184.9195	-4.851886216	0.931353	-5.20951	1.89E-07	7.34E-06	TRUE	Dio3
Ass1	72.78346	-1.775171298	0.395021	-4.49387	6.99E-06	0.00018	TRUE	Ass1
Erich3	174.4539	-1.397349739	0.348106	-4.01415	5.97E-05	0.001133	TRUE	Erich3
Klhdc7a	13.76485	-3.386710565	0.907429	-3.7322	0.00019	0.002956	TRUE	Klhdc7a
Fam43b	41.34664	-4.726422855	0.780861	-6.05283	1.42E-09	9.95E-08	TRUE	Fam43b
Foxd1	200.9311	-1.10008778	0.268949	-4.09032	4.31E-05	0.000852	TRUE	Foxd1
Cfap74	147.4854	-1.200726265	0.302304	-3.97191	7.13E-05	0.001313	TRUE	Cfap74
Arhgap8	13.30534	-5.557329768	1.486238	-3.73919	0.000185	0.002895	TRUE	Arhgap8
Zan	40.30685	-2.758799059	0.668189	-4.12877	3.65E-05	0.000747	TRUE	Zan
Ccdc13	171.7371	-1.486728786	0.357807	-4.15511	3.25E-05	0.000678	TRUE	Ccdc13
Gm2974	131.1693	-1.072527137	0.28726	-3.73365	0.000189	0.002941	TRUE	Gm2974
B3gnt7	53.1853	-1.719635209	0.446782	-3.84894	0.000119	0.002004	TRUE	B3gnt7
Prdm12	406.2472	-3.152855292	0.487957	-6.46134	1.04E-10	8.99E-09	TRUE	Prdm12
Clec2l	233.2153	-1.296246519	0.326027	-3.97589	7.01E-05	0.001297	TRUE	Clec2l
Gm16204	37.01251	-3.741014172	0.828047	-4.51788	6.25E-06	0.000162	TRUE	Gm16204
Ftl2-ps	193.7047	-1.971257942	0.446692	-4.41302	1.02E-05	0.000249	TRUE	Ftl2-ps
Gm12459	19.22912	-2.626207888	0.705914	-3.72029	0.000199	0.003074	TRUE	Gm12459
Gm14776	158.8295	-1.960261086	0.533311	-3.67564	0.000237	0.003571	TRUE	Gm14776
Rnf138rt1	41.17739	-2.811769273	0.537018	-5.23589	1.64E-07	6.41E-06	TRUE	Rnf138rt1
Znf41-ps	70.28705	-1.256325479	0.368443	-3.40983	0.00065	0.00827	TRUE	Znf41-ps
Tmem240	453.0488	-1.225853515	0.286594	-4.27732	1.89E-05	0.000425	TRUE	Tmem240
Gm12371	20.12039	-2.92630474	0.865362	-3.3816	0.000721	0.008959	TRUE	Gm12371
Dlx1as	200.1774	-1.613750583	0.407402	-3.96107	7.46E-05	0.001362	TRUE	Dlx1as
Gm11655	61.63701	-1.771310222	0.502962	-3.52176	0.000429	0.005872	TRUE	Gm11655
Crocc2	42.07358	-3.833539859	0.685953	-5.58864	2.29E-08	1.14E-06	TRUE	Crocc2
30570G19I	129.5415	-1.495940266	0.311874	-4.79662	1.61E-06	4.90E-05	TRUE	4930570G19Rik
Gm13425	312.8001	-3.35283463	0.889883	-3.76773	0.000165	0.002633	TRUE	Gm13425
Dmrta2os	53.26843	-2.321065123	0.520083	-4.46288	8.09E-06	0.000203	TRUE	Dmrta2os
Foxd2os	30.07871	-3.360289703	0.76666	-4.38302	1.17E-05	0.000279	TRUE	Foxd2os
Gm15398	40.97278	-3.077826676	0.660661	-4.65871	3.18E-06	8.91E-05	TRUE	Gm15398
Gm15624	30.04797	-1.916240714	0.514058	-3.72767	0.000193	0.003003	TRUE	Gm15624
Gm13052	52.35739	-1.680292221	0.445944	-3.76794	0.000165	0.002633	TRUE	Gm13052
Rtl1	726.3189	-2.735538312	0.293588	-9.31761	1.19E-20	3.85E-18	TRUE	Rtl1
AW822252	65.09967	-1.674488215	0.428477	-3.908	9.31E-05	0.001631	TRUE	AW822252
Gm2415	275.7036	-1.348130189	0.303561	-4.44106	8.95E-06	0.000222	TRUE	Gm2415
Gm11454	46.23927	-2.65879832	0.663469	-4.00742	6.14E-05	0.001164	TRUE	Gm11454
Igf2os	28.42716	-3.226783126	0.771253	-4.18382	2.87E-05	0.000607	TRUE	Igf2os
Gm14397	14.87016	-2.785014723	0.757839	-3.67494	0.000238	0.003579	TRUE	Gm14397
Gm13889	253.0685	-1.25662072	0.306662	-4.09774	4.17E-05	0.000833	TRUE	Gm13889
30024F11I	96.81321	-4.922364987	0.554947	-8.86998	7.32E-19	1.92E-16	TRUE	9130024F11Rik
Gm14004	28.12905	-2.714617003	0.632961	-4.28876	1.80E-05	0.000406	TRUE	Gm14004
Lbhd2	15.17072	-5.692733178	1.406953	-4.04614	5.21E-05	0.001007	TRUE	Lbhd2
BC064078	40.94439	-2.611868989	0.458937	-5.69113	1.26E-08	6.82E-07	TRUE	BC064078
10035D17I	268.3081	-1.211372688	0.281101	-4.30938	1.64E-05	0.000374	TRUE	2610035D17Rik
Cers1	666.3801	-1.009474978	0.208629	-4.83862	1.31E-06	4.08E-05	TRUE	Cers1
Gm43517	11.08992	-4.063523499	1.212575	-3.35115	0.000805	0.00981	TRUE	Gm43517
30011K09I	140.1491	-1.437592695	0.298307	-4.81917	1.44E-06	4.44E-05	TRUE	A830011K09Rik
Dlx6os1	1455.781	-1.692737475	0.318362	-5.31702	1.05E-07	4.38E-06	TRUE	Dlx6os1
Samd15	21.86371	-2.10488681	0.62833	-3.34997	0.000808	0.009846	TRUE	Samd15
Col6a5	45.78812	-3.382852373	0.532487	-6.35294	2.11E-10	1.74E-08	TRUE	Col6a5
Peg10	9771.018	-2.260536384	0.636287	-3.5527	0.000381	0.00534	TRUE	Peg10
Gm19345	22.26802	-2.16068978	0.584975	-3.69365	0.000221	0.003352	TRUE	Gm19345

Gm20515	73.61702	-2.925531267	0.487994	-5.99502	2.03E-09	1.37E-07	TRUE		Gm20515
Gm20467	23.61667	-6.304818004	1.265119	-4.98358	6.24E-07	2.13E-05	TRUE		Gm20467
Six3os1	5329.43	-1.533617089	0.291953	-5.25296	1.50E-07	5.98E-06	TRUE		Six3os1
Gm21846	35.74094	-2.575438736	0.610456	-4.21888	2.46E-05	0.000532	TRUE		Gm21846
Dlx6os2	262.4524	-2.155656218	0.38143	-5.65152	1.59E-08	8.35E-07	TRUE		Dlx6os2
Nim1k	144.6397	-1.388277676	0.282214	-4.91923	8.69E-07	2.89E-05	TRUE		Nim1k
Sox1	1973.904	-1.420185695	0.1983	-7.16179	7.96E-13	9.81E-11	TRUE		Sox1
830030K20	80.7629	-1.420599518	0.400237	-3.5494	0.000386	0.005389	TRUE		D830030K20Rik
Kcnj11	131.0174	-2.395853841	0.431191	-5.55636	2.75E-08	1.34E-06	TRUE		Kcnj11
Gm5796	71.67385	-1.293554556	0.345873	-3.73997	0.000184	0.002888	TRUE		Gm5796
32441J04	55.0431	-2.011238549	0.423782	-4.74593	2.08E-06	6.16E-05	TRUE		4932441J04Rik
Gm2694	974.6127	-2.067831859	0.26125	-7.91516	2.47E-15	4.22E-13	TRUE		Gm2694
Mirg	1109.617	-1.321210877	0.248877	-5.3087	1.10E-07	4.51E-06	TRUE		Mirg
Rian	17085.36	-1.054836528	0.166486	-6.3359	2.36E-10	1.93E-08	TRUE		Rian
Gm26736	194.906	-1.214188986	0.344178	-3.52779	0.000419	0.005771	TRUE		Gm26736
Gm26592	10.47224	-3.768361442	1.066701	-3.53272	0.000411	0.005679	TRUE		Gm26592
Gm26777	80.21167	-2.244701415	0.391016	-5.74069	9.43E-09	5.34E-07	TRUE		Gm26777
Rpl31-ps1	66.5575	-9.645767563	1.957179	-4.9284	8.29E-07	2.77E-05	TRUE		Rpl31-ps16
Gm26811	101.6633	-1.07138564	0.314298	-3.40882	0.000652	0.008281	TRUE		Gm26811
Tunar	365.0001	-1.12466695	0.289123	-3.88993	0.0001	0.001732	TRUE		Tunar
Rassf10	52.33034	-2.145353888	0.545501	-3.93281	8.40E-05	0.001506	TRUE		Rassf10
Gm26973	54.57512	-4.649708633	0.939521	-4.94902	7.46E-07	2.52E-05	TRUE		Gm26973
830012L14	321.5637	-1.235266299	0.259639	-4.75763	1.96E-06	5.84E-05	TRUE		B830012L14Rik
Gm27239	38.62068	-2.146497531	0.543896	-3.94652	7.93E-05	0.001433	TRUE		Gm27239
Gm29374	138.9484	-1.02879859	0.299271	-3.43768	0.000587	0.007593	TRUE		Gm29374
Gm29514	13.89169	-3.530312023	0.885886	-3.98506	6.75E-05	0.001257	TRUE		Gm29514
30024G19	414.0968	-1.421924863	0.331523	-4.28906	1.79E-05	0.000406	TRUE		B130024G19Rik
Gm28905	7.506795	-5.53306167	1.620991	-3.41338	0.000642	0.008198	TRUE		Gm28905
Gm37474	57.31503	-2.032474891	0.605799	-3.35503	0.000794	0.009708	TRUE		Gm37474
Gm38170	37.39857	-6.985765883	1.288984	-5.41959	5.97E-08	2.64E-06	TRUE		Gm38170
Gm37626	46.57355	-2.16657652	0.497703	-4.35315	1.34E-05	0.000315	TRUE		Gm37626
700039I01	85.38261	-2.137843014	0.359102	-5.95331	2.63E-09	1.72E-07	TRUE		1700039I01Rik
Gm37899	179.5538	-1.181327964	0.284669	-4.14983	3.33E-05	0.000689	TRUE		Gm37899
Gm37519	84.50404	-2.055842162	0.504603	-4.07418	4.62E-05	0.000907	TRUE		Gm37519
Gm38178	12.33535	-3.556209523	0.946136	-3.75867	0.000171	0.002713	TRUE		Gm38178
Gm37720	68.32895	-1.850482153	0.521404	-3.54904	0.000387	0.005393	TRUE		Gm37720
Gm37562	58.74347	-3.535559845	0.591174	-5.98058	2.22E-09	1.47E-07	TRUE		Gm37562
Gm37460	74.97186	-2.92315266	0.468522	-6.2391	4.40E-10	3.48E-08	TRUE		Gm37460
Gm42639	178.0449	-1.945501258	0.334396	-5.81795	5.96E-09	3.54E-07	TRUE		Gm42639
Gm42480	26.85311	-2.80433995	0.670317	-4.1836	2.87E-05	0.000607	TRUE		Gm42480
Gm42685	218.6702	-1.080062784	0.31995	-3.37573	0.000736	0.009121	TRUE		Gm42685
Gm43048	29.98182	-3.127440996	0.695703	-4.49537	6.94E-06	0.000179	TRUE		Gm43048
Lhfpl3	49.0675	-2.104436592	0.573678	-3.66832	0.000244	0.003651	TRUE		Lhfpl3
30093G08	1455.871	-2.705390379	0.307439	-8.79976	1.37E-18	3.30E-16	TRUE		C130093G08Rik
Gm42583	284.9673	-1.301234194	0.237898	-5.46971	4.51E-08	2.06E-06	TRUE		Gm42583
Gm42888	32.39186	-2.169740132	0.537401	-4.03747	5.40E-05	0.001038	TRUE		Gm42888
Gm43461	90.20023	-1.365658356	0.321513	-4.2476	2.16E-05	0.000476	TRUE		Gm43461
10300M13	13.29971	-5.470146068	1.369941	-3.99298	6.52E-05	0.001224	TRUE		2610300M13Rik
Gm44242	41.1478	-2.020936034	0.466653	-4.33071	1.49E-05	0.000346	TRUE		Gm44242
Gm44241	40.48702	-2.186858534	0.530602	-4.12146	3.76E-05	0.000765	TRUE		Gm44241
Gm44094	31.38947	-2.036187634	0.583763	-3.48804	0.000487	0.006519	TRUE		Gm44094
Gm44228	54.91072	-2.299284824	0.509798	-4.51019	6.48E-06	0.000168	TRUE		Gm44228
Gm44229	56.19919	-2.245851282	0.404794	-5.54813	2.89E-08	1.38E-06	TRUE		Gm44229
330118I20	10.53372	-3.848303806	1.082755	-3.55418	0.000379	0.005317	TRUE		9330118I20Rik
Gm44643	24.88764	-3.100509555	0.720333	-4.30427	1.68E-05	0.000382	TRUE		Gm44643
30209E15	18.46412	-6.026907898	1.400729	-4.30269	1.69E-05	0.000384	TRUE		B230209E15Rik
Gm44812	190.7816	-1.542120681	0.348784	-4.42142	9.81E-06	0.000241	TRUE		Gm44812
Gm35842	128.891	-1.371490793	0.347634	-3.94522	7.97E-05	0.00144	TRUE		Gm35842
Gm44586	188.5695	-1.515273508	0.253141	-5.98588	2.15E-09	1.44E-07	TRUE		Gm44586
Gm44587	156.5252	-2.344097039	0.313153	-7.48546	7.13E-14	1.02E-11	TRUE		Gm44587
Gm44799	1201.845	-1.467346116	0.343497	-4.27178	1.94E-05	0.000433	TRUE		Gm44799
Gm44644	86.34541	-2.81867929	0.42891	-6.57173	4.97E-11	4.49E-09	TRUE		Gm44644
230103L15	690.6843	-1.714026084	0.235665	-7.27314	3.51E-13	4.52E-11	TRUE		A230103L15Rik
Gm44541	126.7035	-1.392070812	0.337598	-4.12346	3.73E-05	0.00076	TRUE		Gm44541
Gm44863	266.9839	-1.71604407	0.308628	-5.56024	2.69E-08	1.32E-06	TRUE		Gm44863
Gm44585	27.68066	-2.323688788	0.600633	-3.86874	0.000109	0.001861	TRUE		Gm44585
Gm44724	26.21464	-2.618044249	0.668296	-3.91749	8.95E-05	0.001577	TRUE		Gm44724
30162G02	2418.93	-1.50626616	0.258944	-5.81695	5.99E-09	3.55E-07	TRUE		9330162G02Rik
Gm44831	142.6931	-1.086145361	0.312099	-3.48013	0.000501	0.006667	TRUE		Gm44831
Svet1	150.8817	-2.148542555	0.404531	-5.31119	1.09E-07	4.46E-06	TRUE		Svet1
Gm39244	336.4323	-1.250495783	0.204203	-6.1238	9.14E-10	6.68E-08	TRUE		Gm39244
Gm45259	53.36348	-2.353831649	0.604173	-3.89596	9.78E-05	0.001697	TRUE		Gm45259

Gm6145	28.44612	-3.096027473	0.83529	-3.70653	0.00021	0.003219	TRUE	Gm6145
Gm47757	42.4549	-4.069584303	0.862924	-4.71604	2.40E-06	6.98E-05	TRUE	Gm47757
Gm19169	38.06139	-4.050620751	0.669945	-6.0462	1.48E-09	1.03E-07	TRUE	Gm19169
Gm47163	166.2401	-1.112138765	0.271162	-4.10138	4.11E-05	0.000821	TRUE	Gm47163
Gm47592	76.17833	-2.091717497	0.40841	-5.12162	3.03E-07	1.12E-05	TRUE	Gm47592
Gm5136	59.7436	-1.601269637	0.43084	-3.71662	0.000202	0.003109	TRUE	Gm5136
Gm48536	123.155	-1.38230424	0.303979	-4.54737	5.43E-06	0.000142	TRUE	Gm48536
Gm48280	5.414756	-6.024633157	1.645998	-3.66017	0.000252	0.003748	TRUE	Gm48280
Gm47917	13.17933	-3.714275871	1.041184	-3.56736	0.000361	0.005098	TRUE	Gm47917
Gm47098	42.73658	-2.969039822	0.737048	-4.02829	5.62E-05	0.001075	TRUE	Gm47098
Gm34785	64.24686	-2.771542616	0.492748	-5.62467	1.86E-08	9.52E-07	TRUE	Gm34785
Gm33195	50.76492	-2.061769315	0.547106	-3.7685	0.000164	0.002631	TRUE	Gm33195
Gm47860	80.09119	-1.405118081	0.356879	-3.93724	8.24E-05	0.001481	TRUE	Gm47860
Gm36101	13.00484	-5.466284041	1.556874	-3.51106	0.000446	0.006066	TRUE	Gm36101
Gm10000	19.42504	-7.883136001	1.402198	-5.62198	1.89E-08	9.58E-07	TRUE	Gm10000
Gm30339	5.637212	-6.115580484	1.722974	-3.54943	0.000386	0.005389	TRUE	Gm30339
Gm1043	184.8091	-1.30829083	0.369013	-3.54538	0.000392	0.005461	TRUE	Gm1043
Gm5231	18.81685	-6.826105343	1.64706	-4.14442	3.41E-05	0.000703	TRUE	Gm5231
Gm32341	23.98014	-2.800548362	0.804604	-3.48065	0.0005	0.006666	TRUE	Gm32341
