# Supplementary material for: Temporal-spatial Generation of Astrocytes in the Developing Diencephalon
Source: Neurosci Bull. 2023 Oct 16;40(1):1–16. doi: 10.1007/s12264-023-01131-9 (PMC10774245; doi:10.1007/s12264-023-01131-9)
Supplement: Supplementary file 3 — Supplementary file3 (PDF 1023 kb) [file 12264_2023_1131_MOESM3_ESM.pdf]

Table S3. List of genes that were up-regulated in the dorsal wall of 3V compared with those in the dorsal wall of LV

SYMBOL	baseMean	log2FoldChange	lfcSE	stat	pvalue	padj	significant	LV-d_vs_3V-d_upregulated gene
Gnai3	1013.091	-1.562374515	0.409179	-3.81831	0.000134	0.000659	TRUE	Gnai3
Narf	1119.793	-1.798534247	0.267902	-6.7134	1.90E-11	3.61E-10	TRUE	Narf
Klf6	293.8467	-2.60509953	0.329818	-7.89861	2.82E-15	8.79E-14	TRUE	Klf6
Cox5a	274.8709	-1.494286172	0.420242	-3.55578	0.000377	0.00163	TRUE	Cox5a
Ngfr	358.7236	-4.730355729	0.327678	-14.436	3.07E-47	1.64E-44	TRUE	Ngfr
Axin2	746.4873	-2.258691348	0.338173	-6.6791	2.40E-11	4.49E-10	TRUE	Axin2
Gna12	1269.969	-1.078166044	0.299976	-3.59417	0.000325	0.001436	TRUE	Gna12
Nalcn	163.2887	-3.075102835	0.710931	-4.32546	1.52E-05	9.50E-05	TRUE	Nalcn
Th	25.93005	-5.124225841	1.139743	-4.49595	6.93E-06	4.66E-05	TRUE	Th
Drp2	685.1153	-2.007076765	0.265646	-7.55547	4.17E-14	1.12E-12	TRUE	Drp2
Gmpr	44.64932	-1.261406134	0.446347	-2.82606	0.004712	0.014743	TRUE	Gmpr
Mid2	38.21727	-1.670683475	0.616145	-2.71151	0.006698	0.019833	TRUE	Mid2
Dgke	354.9931	-1.012090406	0.236837	-4.27337	1.93E-05	0.000117	TRUE	Dgke
Ckmt1	39.29191	-1.587263053	0.540486	-2.93673	0.003317	0.010925	TRUE	Ckmt1
Ndufa9	320.3367	-1.129761642	0.342819	-3.2955	0.000982	0.003789	TRUE	Ndufa9
Egfl6	11.34935	-2.552146883	1.092805	-2.33541	0.019522	0.048688	TRUE	Egfl6
Galnt1	544.9214	-1.008776103	0.427428	-2.36011	0.01827	0.046056	TRUE	Galnt1
Raf1	781.8132	-1.018215981	0.221125	-4.6047	4.13E-06	2.93E-05	TRUE	Raf1
Gabra2	386.0011	-1.329375121	0.368538	-3.60716	0.00031	0.001376	TRUE	Gabra2
Atp5f1	1592.177	-1.498365277	0.472123	-3.17367	0.001505	0.005521	TRUE	Atp5f1
C1d	469.9782	-1.614653958	0.373452	-4.32359	1.54E-05	9.57E-05	TRUE	C1d
Sema4f	195.8196	-1.418827864	0.398875	-3.55707	0.000375	0.001623	TRUE	Sema4f
Rab5b	758.3702	-1.669601276	0.351087	-4.75553	1.98E-06	1.51E-05	TRUE	Rab5b
Aire	6.412692	-4.815607729	1.526547	-3.15458	0.001607	0.005838	TRUE	Aire
Ddx3x	3192.613	-1.132814331	0.349493	-3.24131	0.00119	0.00449	TRUE	Ddx3x
Usp32	634.7188	-1.356799146	0.254936	-5.32213	1.03E-07	1.05E-06	TRUE	Usp32
Car4	8.008662	-3.466933024	1.31243	-2.64161	0.008251	0.023698	TRUE	Car4
Dnajc5	2004.026	-1.407162594	0.194657	-7.22892	4.87E-13	1.14E-11	TRUE	Dnajc5
Il4	105.7854	-1.055626213	0.306013	-3.44961	0.000561	0.002314	TRUE	Il4
Gm38393	126.9366	-3.093658248	0.541737	-5.71063	1.13E-08	1.39E-07	TRUE	Gm38393
Kctd10	373.6005	-2.311381271	0.231026	-10.0048	1.45E-23	1.06E-21	TRUE	Kctd10
Calm1	8184.801	-1.223491153	0.219778	-5.56695	2.59E-08	2.99E-07	TRUE	Calm1
Sema6b	591.483	-2.152865677	0.426975	-5.04213	4.60E-07	4.09E-06	TRUE	Sema6b
Ramp2	53.75224	-1.19349186	0.451326	-2.64441	0.008183	0.023523	TRUE	Ramp2
Gabrg1	4.582812	-4.32878617	1.617362	-2.67645	0.007441	0.02166	TRUE	Gabrg1
Srr	152.7766	-1.70477252	0.342664	-4.97506	6.52E-07	5.60E-06	TRUE	Srr
Cyp51	1509.887	-1.688125138	0.324739	-5.19841	2.01E-07	1.91E-06	TRUE	Cyp51
Nkx2-1	73.88068	-2.002053826	0.728386	-2.74862	0.005985	0.018104	TRUE	Nkx2-1
Itga3	120.5146	-1.384153057	0.400617	-3.45505	0.00055	0.002274	TRUE	Itga3
Tubb5	25840.15	-1.005344902	0.256292	-3.92265	8.76E-05	0.00045	TRUE	Tubb5
Jup	656.2163	-3.262900474	0.252674	-12.9135	3.78E-38	1.02E-35	TRUE	Jup
Ergic1	1023.327	-1.235884393	0.36593	-3.37738	0.000732	0.002927	TRUE	Ergic1
Gstt1	22.33684	-1.676976423	0.653353	-2.56672	0.010267	0.02847	TRUE	Gstt1
Eef1e1	329.7761	-1.99692721	0.448391	-4.45354	8.45E-06	5.59E-05	TRUE	Eef1e1
Rin2	120.6793	-1.011298745	0.377538	-2.67867	0.007392	0.021532	TRUE	Rin2
Rac1	1228.288	-1.609665693	0.482907	-3.33329	0.000858	0.003372	TRUE	Rac1
Ugp2	253.2534	-1.10679154	0.26578	-4.16432	3.12E-05	0.00018	TRUE	Ugp2
Kcnh6	13.73927	-2.802397385	1.033193	-2.71237	0.00668	0.019787	TRUE	Kcnh6
Uba1	4323.063	-1.568434648	0.17424	-9.00159	2.22E-19	1.09E-17	TRUE	Uba1
Grik3	666.4355	-3.124397946	0.489572	-6.3819	1.75E-10	2.85E-09	TRUE	Grik3
Sipa1l2	1027.938	-1.349628587	0.186814	-7.22447	5.03E-13	1.17E-11	TRUE	Sipa1l2
Bbc3	224.7206	-2.396967968	0.344324	-6.96137	3.37E-12	7.11E-11	TRUE	Bbc3
Peg3	1615.743	-1.400113789	0.210581	-6.64883	2.95E-11	5.44E-10	TRUE	Peg3
Ncan	5921.454	-1.545631449	0.185875	-8.31545	9.14E-17	3.36E-15	TRUE	Ncan
Nr2f6	179.6697	-1.005771043	0.304187	-3.30642	0.000945	0.003666	TRUE	Nr2f6
Slc9a3r2	116.0802	-1.283231589	0.292079	-4.39345	1.12E-05	7.16E-05	TRUE	Slc9a3r2
Mien1	54.25687	-1.418920992	0.604144	-2.34865	0.018842	0.047241	TRUE	Mien1
Tgfb1	22.12496	-1.661308923	0.660144	-2.51659	0.01185	0.032172	TRUE	Tgfb1
Shh	8.832159	-5.284307197	1.52646	-3.4618	0.000537	0.002221	TRUE	Shh
Lcp2	4.100231	-4.179076973	1.744514	-2.39555	0.016595	0.042555	TRUE	Lcp2
Ykt6	1652.03	-1.09139106	0.246505	-4.42746	9.54E-06	6.21E-05	TRUE	Ykt6
Grin2d	189.2868	-2.365798157	0.470449	-5.02881	4.94E-07	4.36E-06	TRUE	Grin2d
Kdelr1	567.2463	-1.03523794	0.41966	-2.46685	0.013631	0.036136	TRUE	Kdelr1
Ggct	32.23778	-1.679986973	0.60164	-2.79234	0.005233	0.016158	TRUE	Ggct
Jag2	608.7556	-1.490723951	0.310556	-4.80017	1.59E-06	1.24E-05	TRUE	Jag2
Kcnn1	572.2786	-2.115257531	0.279116	-7.57842	3.50E-14	9.44E-13	TRUE	Kcnn1
Prkar2b	927.6289	-2.287634506	0.215819	-10.5998	2.99E-26	2.80E-24	TRUE	Prkar2b
Pafah1b2	2538.83	-1.424749228	0.267833	-5.31954	1.04E-07	1.06E-06	TRUE	Pafah1b2
Slc2a3	170.3691	-1.231653066	0.329554	-3.73733	0.000186	0.000878	TRUE	Slc2a3

Edar	6.484029	-3.872970713	1.421276	-2.725	0.00643	0.019204	TRUE	Edar
Grk5	186.9807	-1.078110669	0.304575	-3.53972	0.000401	0.001719	TRUE	Grk5
Sult2b1	23.57386	-3.335009807	0.791752	-4.21219	2.53E-05	0.000149	TRUE	Sult2b1
Car11	187.5217	-1.02760255	0.404396	-2.54108	0.011051	0.030341	TRUE	Car11
Mob3a	534.4454	-1.541677484	0.210156	-7.33586	2.20E-13	5.35E-12	TRUE	Mob3a
Rabac1	353.7306	-1.311153208	0.251151	-5.22057	1.78E-07	1.72E-06	TRUE	Rabac1
Rab3b	79.7033	-3.401200038	0.54205	-6.27469	3.50E-10	5.50E-09	TRUE	Rab3b
Pex19	959.3609	-1.304786035	0.36023	-3.6221	0.000292	0.001308	TRUE	Pex19
Dusp3	50.97139	-1.61311399	0.593826	-2.71647	0.006598	0.019596	TRUE	Dusp3
Slc25a1	751.9396	-1.15521756	0.195427	-5.91124	3.40E-09	4.55E-08	TRUE	Slc25a1
As3mt	64.22038	-1.41392666	0.418423	-3.37918	0.000727	0.002913	TRUE	As3mt
Cp	12.5399	-2.070630009	0.832322	-2.48778	0.012854	0.034353	TRUE	Cp
Calb2	459.9876	-5.74077378	1.36887	-4.1938	2.74E-05	0.00016	TRUE	Calb2
Itpkc	84.30382	-1.532825801	0.442389	-3.46488	0.00053	0.002201	TRUE	Itpkc
Lin7b	23.10835	-3.463025573	0.789846	-4.38443	1.16E-05	7.43E-05	TRUE	Lin7b
Zfp81	489.5373	-1.107963584	0.31994	-3.46304	0.000534	0.002213	TRUE	Zfp81
Efnb3	2979.314	-3.191564775	0.297151	-10.7406	6.56E-27	6.80E-25	TRUE	Efnb3
Mmd	548.1568	-1.602818275	0.265834	-6.0294	1.65E-09	2.30E-08	TRUE	Mmd
Hlf	84.43916	-4.222624528	0.718163	-5.87976	4.11E-09	5.42E-08	TRUE	Hlf
Grm3	21.8416	-6.588476453	1.28948	-5.10941	3.23E-07	2.94E-06	TRUE	Grm3
Gstm7	563.8286	-2.606144004	0.257584	-10.1176	4.61E-24	3.67E-22	TRUE	Gstm7
Cavin1	82.24514	-1.758439875	0.578712	-3.03854	0.002377	0.008241	TRUE	Cavin1
Map3k20	531.7265	-2.853715145	0.314997	-9.0595	1.31E-19	6.58E-18	TRUE	Map3k20
Cacna1e	781.1892	-2.241266261	0.435932	-5.14132	2.73E-07	2.53E-06	TRUE	Cacna1e
Cacna1b	825.7309	-2.838538117	0.391864	-7.24369	4.37E-13	1.03E-11	TRUE	Cacna1b
330159F19R	1268.345	-2.137330939	0.20116	-10.625	2.28E-26	2.16E-24	TRUE	9330159F19Rik
Cul3	1052.188	-1.218128589	0.313359	-3.88732	0.000101	0.000514	TRUE	Cul3
Col26a1	46.96929	-3.690630716	0.648522	-5.69084	1.26E-08	1.55E-07	TRUE	Col26a1
Bid	80.18389	-1.119184539	0.456905	-2.44949	0.014306	0.037595	TRUE	Bid
Zfp324	228.2703	-1.025947305	0.301476	-3.40308	0.000666	0.002699	TRUE	Zfp324
Gab2	301.511	-2.263097177	0.328913	-6.88054	5.96E-12	1.21E-10	TRUE	Gab2
Coro1c	2113.411	-1.27881408	0.282326	-4.52957	5.91E-06	4.05E-05	TRUE	Coro1c
Sgce	283.2421	-1.231503366	0.248365	-4.95844	7.11E-07	6.05E-06	TRUE	Sgce
Hdac9	185.9346	-1.730739671	0.360839	-4.79643	1.62E-06	1.26E-05	TRUE	Hdac9
Adgre1	4.644968	-4.35689514	1.604266	-2.71582	0.006611	0.019626	TRUE	Adgre1
Rab11a	746.5521	-1.938233467	0.388017	-4.99523	5.88E-07	5.09E-06	TRUE	Rab11a
Ulk2	1166.763	-1.807867047	0.272714	-6.62918	3.38E-11	6.17E-10	TRUE	Ulk2
Rasa4	108.2483	-1.682406022	0.479763	-3.50674	0.000454	0.001919	TRUE	Rasa4
Syt5	306.6364	-1.453187002	0.282873	-5.13725	2.79E-07	2.58E-06	TRUE	Syt5
Prkacb	951.5011	-2.033515894	0.409197	-4.96952	6.71E-07	5.75E-06	TRUE	Prkacb
Chd5	655.6211	-1.703958107	0.379296	-4.49242	7.04E-06	4.73E-05	TRUE	Chd5
Cstb	80.45834	-1.569093916	0.464783	-3.37597	0.000736	0.002941	TRUE	Cstb
Slc1a2	3671.041	-5.078059916	1.232812	-4.11909	3.80E-05	0.000215	TRUE	Slc1a2
Plekha8	530.4687	-1.018782825	0.243605	-4.18211	2.89E-05	0.000168	TRUE	Plekha8
Ubqln1	1340.734	-1.082542547	0.277452	-3.90173	9.55E-05	0.000487	TRUE	Ubqln1
Cadm3	1420.891	-2.100763293	0.29867	-7.03372	2.01E-12	4.37E-11	TRUE	Cadm3
Crbn	188.7961	-1.102123981	0.301897	-3.65066	0.000262	0.001187	TRUE	Crbn
Nid1	133.0479	-2.516581895	0.814164	-3.091	0.001995	0.00705	TRUE	Nid1
Pafah1b3	1604.696	-1.251495166	0.183916	-6.80473	1.01E-11	1.99E-10	TRUE	Pafah1b3
Prkaca	980.2318	-1.630181683	0.472265	-3.45184	0.000557	0.002298	TRUE	Prkaca
Kbtbd4	74.74625	-1.010188288	0.337913	-2.98949	0.002794	0.00946	TRUE	Kbtbd4
Adcy9	66.41583	-1.842728976	0.453289	-4.06524	4.80E-05	0.000264	TRUE	Adcy9
Eif4g2	14160.85	-1.593797663	0.307757	-5.17875	2.23E-07	2.11E-06	TRUE	Eif4g2
Pcyt1a	433.1655	-1.747436434	0.261021	-6.69462	2.16E-11	4.07E-10	TRUE	Pcyt1a
Slc30a4	120.7889	-1.975468468	0.43659	-4.52476	6.05E-06	4.12E-05	TRUE	Slc30a4
Bloc1s6	391.8241	-1.243381903	0.273623	-4.54414	5.52E-06	3.79E-05	TRUE	Bloc1s6
Apc	2619.919	-2.105871446	0.247307	-8.51523	1.66E-17	6.55E-16	TRUE	Apc
Reep5	689.4303	-2.492686507	0.273402	-9.11731	7.70E-20	4.01E-18	TRUE	Reep5
Nr2c1	136.3682	-1.428371793	0.337807	-4.22837	2.35E-05	0.00014	TRUE	Nr2c1
Tuft1	14.26742	-1.898285691	0.790405	-2.40166	0.016321	0.04196	TRUE	Tuft1
Crkl	959.0414	-1.382299171	0.337497	-4.09574	4.21E-05	0.000235	TRUE	Crkl
Fblim1	232.9184	-2.490178912	0.58742	-4.23918	2.24E-05	0.000134	TRUE	Fblim1
Ccdc159	51.95669	-2.207466879	0.460535	-4.79327	1.64E-06	1.28E-05	TRUE	Ccdc159
Vax1	15.14489	-3.381189786	1.416325	-2.3873	0.016973	0.043358	TRUE	Vax1
Atp6v1b2	1558.554	-1.440982138	0.201202	-7.16186	7.96E-13	1.81E-11	TRUE	Atp6v1b2
Crip2	1063.568	-1.594724173	0.25903	-6.15652	7.44E-10	1.11E-08	TRUE	Crip2
Pgrmc1	2064.414	-1.919372703	0.34882	-5.50247	3.75E-08	4.16E-07	TRUE	Pgrmc1
Tek	37.99731	-1.816067009	0.677239	-2.68157	0.007328	0.02137	TRUE	Tek
Adamts4	142.8866	-2.141809096	0.433994	-4.93511	8.01E-07	6.73E-06	TRUE	Adamts4
Neurl1a	96.11898	-1.792451828	0.48122	-3.72481	0.000195	0.000917	TRUE	Neurl1a
Epha2	19.66773	-1.919086273	0.671364	-2.85849	0.004257	0.013527	TRUE	Epha2
Zdhhc24	146.5164	-1.30599604	0.288256	-4.53068	5.88E-06	4.03E-05	TRUE	Zdhhc24

Runx1t1	1996.35	-3.896710697	0.422237	-9.22873	2.74E-20	1.48E-18	TRUE	Runx1t1
Aplp1	3663.223	-2.956452495	0.178802	-16.5348	2.06E-61	1.95E-58	TRUE	Aplp1
Cdc42	2273.063	-1.566318871	0.459702	-3.40725	0.000656	0.002661	TRUE	Cdc42
130043K22F	15.73616	-2.227947114	0.865505	-2.57416	0.010048	0.027956	TRUE	D130043K22Rik
B4galnt1	309.5863	-2.024369699	0.343176	-5.89893	3.66E-09	4.86E-08	TRUE	B4galnt1
Cnp	150.9687	-2.028547194	0.430789	-4.70892	2.49E-06	1.86E-05	TRUE	Cnp
Sulf2	287.5759	-3.371285385	0.487026	-6.92219	4.45E-12	9.22E-11	TRUE	Sulf2
Sod2	513.555	-1.104546088	0.393888	-2.80421	0.005044	0.015635	TRUE	Sod2
Stambp	245.0353	-1.331530725	0.2556	-5.20944	1.89E-07	1.82E-06	TRUE	Stambp
Hap1	2232.987	-2.075893045	0.257646	-8.05717	7.81E-16	2.60E-14	TRUE	Hap1
Syngr3	381.5025	-1.395302104	0.213219	-6.54398	5.99E-11	1.05E-09	TRUE	Syngr3
Gatad1	598.0872	-1.032654109	0.325921	-3.16842	0.001533	0.005609	TRUE	Gatad1
Ppp2r1a	3290.576	-1.063816708	0.279036	-3.81247	0.000138	0.000673	TRUE	Ppp2r1a
Tgfbr1	1265.845	-1.197491384	0.216058	-5.54245	2.98E-08	3.40E-07	TRUE	Tgfbr1
Gabrb2	104.0003	-6.266447799	1.720796	-3.6416	0.000271	0.001224	TRUE	Gabrb2
Bcl2l1	1256.291	-1.857395712	0.278462	-6.67019	2.55E-11	4.76E-10	TRUE	Bcl2l1
Rhoa	1251.676	-1.302708217	0.466793	-2.79076	0.005258	0.01622	TRUE	Rhoa
Hnrnpa0	7458.687	-1.180716706	0.418323	-2.8225	0.004765	0.014877	TRUE	Hnrnpa0
Id3	126.6756	-2.167796202	0.578691	-3.74604	0.00018	0.000851	TRUE	Id3
Hmgcll1	181.2153	-2.939084204	0.403806	-7.27846	3.38E-13	8.07E-12	TRUE	Hmgcll1
Fzd3	1953.567	-1.581642617	0.331346	-4.77339	1.81E-06	1.39E-05	TRUE	Fzd3
Clstn3	569.7363	-2.134236395	0.339609	-6.28439	3.29E-10	5.19E-09	TRUE	Clstn3
Carhsp1	3090.945	-2.707175085	0.296795	-9.12135	7.42E-20	3.88E-18	TRUE	Carhsp1
Herpud2	319.1312	-1.356367447	0.419325	-3.23465	0.001218	0.004582	TRUE	Herpud2
Arpc5	1426.884	-1.610394871	0.229821	-7.00717	2.43E-12	5.25E-11	TRUE	Arpc5
Pou2f2	3372.531	-2.278476717	0.478574	-4.76097	1.93E-06	1.47E-05	TRUE	Pou2f2
Rala	1208.135	-1.117585866	0.23007	-4.85759	1.19E-06	9.57E-06	TRUE	Rala
Pdcl	473.3686	-1.400108204	0.44868	-3.1205	0.001805	0.006456	TRUE	Pdcl
Nf2	1724.099	-1.418646057	0.186313	-7.61431	2.65E-14	7.29E-13	TRUE	Nf2
Bcl2l13	525.6702	-1.126235873	0.364294	-3.09156	0.001991	0.00704	TRUE	Bcl2l13
Lnpk	289.6056	-1.161290656	0.424757	-2.73401	0.006257	0.018764	TRUE	Lnpk
Met	12.64294	-5.8005173	1.382606	-4.19535	2.72E-05	0.000159	TRUE	Met
Elk1	343.6993	-1.68819668	0.224703	-7.51301	5.78E-14	1.51E-12	TRUE	Elk1
Tnpo1	1628.01	-1.464947678	0.338114	-4.3327	1.47E-05	9.23E-05	TRUE	Tnpo1
330409D20F	13.11948	-2.878210147	0.899735	-3.19895	0.001379	0.005112	TRUE	6330409D20Rik
Cbx5	4562.738	-1.211710472	0.280501	-4.3198	1.56E-05	9.72E-05	TRUE	Cbx5
Ppp2cb	615.3332	-1.365835572	0.403364	-3.38611	0.000709	0.002849	TRUE	Ppp2cb
G0s2	15.54151	-3.412325372	1.053132	-3.24017	0.001195	0.004505	TRUE	G0s2
Pou6f2	90.03318	-6.467992592	0.907599	-7.12649	1.03E-12	2.32E-11	TRUE	Pou6f2
Nuak2	581.92	-1.377256565	0.255672	-5.38682	7.17E-08	7.57E-07	TRUE	Nuak2
Ick	1138.617	-1.246653318	0.191622	-6.50579	7.73E-11	1.32E-09	TRUE	Ick
Snap47	868.4221	-1.801347896	0.212823	-8.46406	2.58E-17	1.00E-15	TRUE	Snap47
Wnt3a	3.801813	-4.056402148	1.739854	-2.33146	0.019729	0.049093	TRUE	Wnt3a
Cacna2d2	706.3838	-3.29646972	0.371877	-8.86442	7.69E-19	3.50E-17	TRUE	Cacna2d2
Epn3	12.93519	-3.4895432	1.321745	-2.6401	0.008288	0.023781	TRUE	Epn3
Spire2	418.6854	-1.262682177	0.348696	-3.62116	0.000293	0.001311	TRUE	Spire2
Prox1	607.5467	-4.723439406	0.626809	-7.53569	4.86E-14	1.28E-12	TRUE	Prox1
AI597479	310.2478	-1.737277414	0.363537	-4.77882	1.76E-06	1.36E-05	TRUE	AI597479
Ebf3	334.8594	-3.092905419	0.364562	-8.4839	2.18E-17	8.47E-16	TRUE	Ebf3
Plcd1	222.2912	-2.425289569	0.45762	-5.29979	1.16E-07	1.17E-06	TRUE	Plcd1
Gabra1	69.01375	-8.246334797	1.221008	-6.75371	1.44E-11	2.78E-10	TRUE	Gabra1
Sptbn4	172.2489	-2.024082054	0.613774	-3.29776	0.000975	0.003766	TRUE	Sptbn4
Evi5l	1103.604	-1.400163483	0.204623	-6.84266	7.77E-12	1.56E-10	TRUE	Evi5l
Cdh23	20.47072	-2.392323513	0.756796	-3.16112	0.001572	0.00573	TRUE	Cdh23
Gfod2	956.2162	-1.227068297	0.202023	-6.0739	1.25E-09	1.78E-08	TRUE	Gfod2
Atp6v0d1	916.1184	-1.234168155	0.247468	-4.98719	6.13E-07	5.29E-06	TRUE	Atp6v0d1
Bcas1	48.80148	-3.348037952	0.842215	-3.97528	7.03E-05	0.000372	TRUE	Bcas1
Atad1	783.4647	-1.471095534	0.375446	-3.91826	8.92E-05	0.000458	TRUE	Atad1
Pten	720.417	-1.397518216	0.323679	-4.3176	1.58E-05	9.81E-05	TRUE	Pten
St3gal1	211.5985	-1.698081845	0.347561	-4.88571	1.03E-06	8.44E-06	TRUE	St3gal1
Clip3	2527.163	-1.115215655	0.32266	-3.45632	0.000548	0.002264	TRUE	Clip3
Chp1	484.0147	-1.297647703	0.264422	-4.9075	9.22E-07	7.64E-06	TRUE	Chp1
Klhl3	171.5224	-1.930357582	0.518252	-3.72475	0.000196	0.000917	TRUE	Klhl3
Ube2z	1052.218	-1.269675444	0.246326	-5.15445	2.54E-07	2.37E-06	TRUE	Ube2z
Ankrd52	686.2823	-1.144877513	0.256235	-4.46807	7.89E-06	5.24E-05	TRUE	Ankrd52
Rbsn	479.9547	-1.030996238	0.235452	-4.3788	1.19E-05	7.61E-05	TRUE	Rbsn
Camta1	1555.557	-1.396491588	0.181345	-7.70074	1.35E-14	3.88E-13	TRUE	Camta1
Kif1a	3760.581	-1.223433832	0.189185	-6.46685	1.00E-10	1.68E-09	TRUE	Kif1a
Chfr	778.8457	-1.245367298	0.191856	-6.49115	8.52E-11	1.45E-09	TRUE	Chfr
Nol3	20.03141	-3.808189873	0.965098	-3.94591	7.95E-05	0.000413	TRUE	Nol3
931428F04F	710.5429	-2.456395219	0.256023	-9.59444	8.44E-22	5.20E-20	TRUE	4931428F04Rik
Dnajb9	123.9753	-1.721520649	0.439762	-3.91466	9.05E-05	0.000464	TRUE	Dnajb9

Ppp1cb	1911.734	-1.69578156	0.426953	-3.97182	7.13E-05	0.000377	TRUE		Ppp1cb
Trappc2l	191.5375	-1.098151733	0.276737	-3.96822	7.24E-05	0.000382	TRUE		Trappc2l
Gata2	68.66141	-4.509225668	0.705274	-6.39358	1.62E-10	2.66E-09	TRUE		Gata2
Clic3	24.20803	-1.387912214	0.575349	-2.4123	0.015852	0.040993	TRUE		Clic3
Casd1	564.6617	-1.351860277	0.239327	-5.6486	1.62E-08	1.94E-07	TRUE		Casd1
Map2	5560.314	-2.268951304	0.261033	-8.69219	3.56E-18	1.51E-16	TRUE		Map2
Abca1	198.9643	-1.399246816	0.434669	-3.21911	0.001286	0.004803	TRUE		Abca1
Nipsnap3b	232.1517	-1.875420687	0.311963	-6.01168	1.84E-09	2.54E-08	TRUE		Nipsnap3b
Cd83	33.19239	-7.192773387	1.292145	-5.56654	2.60E-08	2.99E-07	TRUE		Cd83
Prrt1	195.4365	-2.860055762	0.475311	-6.01722	1.77E-09	2.47E-08	TRUE		Prrt1
Rnf5	707.3759	-1.639419942	0.373339	-4.39124	1.13E-05	7.23E-05	TRUE		Rnf5
Gata3	48.64807	-3.636276929	0.832876	-4.36593	1.27E-05	8.04E-05	TRUE		Gata3
Serac1	136.061	-1.327459003	0.381888	-3.47604	0.000509	0.002124	TRUE		Serac1
Psma2	650.5906	-1.144510124	0.347079	-3.29755	0.000975	0.003767	TRUE		Psma2
Arnt2	2984.417	-1.421732062	0.191871	-7.40983	1.26E-13	3.18E-12	TRUE		Arnt2
Prune1	520.7572	-1.846152847	0.291454	-6.33429	2.38E-10	3.80E-09	TRUE		Prune1
Capza2	1010.847	-1.364524224	0.410045	-3.32775	0.000876	0.00343	TRUE		Capza2
Cnih1	499.8557	-1.269442909	0.402699	-3.15234	0.00162	0.005871	TRUE		Cnih1
Eps8	60.82955	-4.144998896	0.624527	-6.63702	3.20E-11	5.86E-10	TRUE		Eps8
Qdpr	278.016	-1.238027702	0.290006	-4.26897	1.96E-05	0.000119	TRUE		Qdpr
Tnr	52.464	-1.991480282	0.806276	-2.46997	0.013512	0.035881	TRUE		Tnr
Sqstm1	1300.77	-1.190424383	0.189635	-6.27744	3.44E-10	5.41E-09	TRUE		Sqstm1
Lcorl	384.836	-1.031071047	0.271971	-3.7911	0.00015	0.000725	TRUE		Lcorl
Dstn	1720.519	-1.675262932	0.362391	-4.62281	3.79E-06	2.71E-05	TRUE		Dstn
Stk32c	59.0774	-8.023665811	1.260981	-6.36304	1.98E-10	3.20E-09	TRUE		Stk32c
Tenm1	83.81373	-4.850916407	0.695719	-6.97252	3.11E-12	6.60E-11	TRUE		Tenm1
Camk1g	259.212	-2.384407022	0.455457	-5.23519	1.65E-07	1.61E-06	TRUE		Camk1g
Syt14	333.1227	-1.086590988	0.283844	-3.82812	0.000129	0.000636	TRUE		Syt14
Kcnq2	1519.659	-1.069990113	0.413578	-2.58715	0.009677	0.027142	TRUE		Kcnq2
Eef1a2	199.1166	-2.301090373	0.430655	-5.34324	9.13E-08	9.41E-07	TRUE		Eef1a2
Ndufa1	142.6856	-1.047190012	0.387919	-2.6995	0.006944	0.020461	TRUE		Ndufa1
Cd34	124.3137	-1.500374837	0.562558	-2.66706	0.007652	0.0222	TRUE		Cd34
Atxn10	1628.178	-1.568507059	0.356499	-4.39975	1.08E-05	6.98E-05	TRUE		Atxn10
H3f3b	8570.763	-1.440665242	0.401317	-3.58984	0.000331	0.001457	TRUE		H3f3b
Tox4	679.9615	-1.196102898	0.249634	-4.79144	1.66E-06	1.29E-05	TRUE		Tox4
Rnd3	2761.288	-2.836729291	0.320092	-8.86224	7.84E-19	3.56E-17	TRUE		Rnd3
Cntnap1	43.61475	-1.308637423	0.522152	-2.50624	0.012202	0.032991	TRUE		Cntnap1
Glod4	307.3213	-1.157199206	0.398515	-2.90378	0.003687	0.011984	TRUE		Glod4
Stac2	48.96511	-2.584161831	0.846922	-3.05124	0.002279	0.007941	TRUE		Stac2
Cacnb4	177.5673	-1.771315883	0.277906	-6.3738	1.84E-10	3.00E-09	TRUE		Cacnb4
Plxdc1	11.40243	-3.005441418	0.954557	-3.14852	0.001641	0.005939	TRUE		Plxdc1
Arl5b	751.6601	-1.70173974	0.265098	-6.41929	1.37E-10	2.28E-09	TRUE		Arl5b
Timp2	485.0231	-1.094955632	0.260897	-4.19689	2.71E-05	0.000159	TRUE		Timp2
Top2b	3517.587	-1.687175157	0.255786	-6.59605	4.22E-11	7.61E-10	TRUE		Top2b
Abr	2593.727	-2.155276044	0.290882	-7.40945	1.27E-13	3.19E-12	TRUE		Abr
Ttpal	633.7963	-1.027407167	0.186195	-5.5179	3.43E-08	3.86E-07	TRUE		Ttpal
Serinc3	565.2694	-1.519825709	0.286046	-5.31321	1.08E-07	1.09E-06	TRUE		Serinc3
Mmp9	72.62331	-2.936419007	0.486062	-6.04124	1.53E-09	2.15E-08	TRUE		Mmp9
Slc12a5	318.9933	-1.098561383	0.343517	-3.19798	0.001384	0.005125	TRUE		Slc12a5
Crk	1318.689	-1.412472662	0.375422	-3.76236	0.000168	0.000804	TRUE		Crk
Pitpna	1220.834	-1.366701503	0.352527	-3.87687	0.000106	0.000533	TRUE		Pitpna
Rab5a	502.0909	-1.903206109	0.351343	-5.41694	6.06E-08	6.50E-07	TRUE		Rab5a
Nkiras2	690.0415	-1.83157863	0.303482	-6.03522	1.59E-09	2.22E-08	TRUE		Nkiras2
Ppp2r5c	1101.294	-1.423220958	0.278757	-5.1056	3.30E-07	3.00E-06	TRUE		Ppp2r5c
Gdap1l1	1169.801	-1.372267696	0.351355	-3.90564	9.40E-05	0.00048	TRUE		Gdap1l1
Rac3	483.7107	-1.059477203	0.321728	-3.29308	0.000991	0.003819	TRUE		Rac3
Cyb5r3	1101.576	-1.057732564	0.204598	-5.16981	2.34E-07	2.20E-06	TRUE		Cyb5r3
Mafk	173.4255	-1.212855579	0.36741	-3.30109	0.000963	0.003731	TRUE		Mafk
Stk4	444.0395	-1.265692152	0.288631	-4.38515	1.16E-05	7.41E-05	TRUE		Stk4
Mtmr4	1321.986	-1.473777166	0.18054	-8.16318	3.26E-16	1.12E-14	TRUE		Mtmr4
Mapt	6120.626	-2.571735621	0.454273	-5.66121	1.50E-08	1.81E-07	TRUE		Mapt
Ypel2	410.8537	-2.496592121	0.267699	-9.32613	1.10E-20	6.17E-19	TRUE		Ypel2
330403K07R	3404.102	-1.527348951	0.233082	-6.55284	5.65E-11	9.92E-10	TRUE		6330403K07Rik
Wnt9b	26.47033	-5.168975145	1.03507	-4.99384	5.92E-07	5.13E-06	TRUE		Wnt9b
Trpv2	117.5663	-3.383959032	0.655485	-5.16253	2.44E-07	2.28E-06	TRUE		Trpv2
Pip4k2b	1244.931	-1.778922222	0.26118	-6.81109	9.69E-12	1.91E-10	TRUE		Pip4k2b
Glra2	390.417	-4.649034289	1.627395	-2.85673	0.00428	0.013591	TRUE		Glra2
Crhr1	23.24897	-1.661833066	0.626214	-2.65378	0.00796	0.022972	TRUE		Crhr1
Sp2	235.801	-1.276197596	0.334468	-3.81561	0.000136	0.000665	TRUE		Sp2
Lhx1	216.1251	-6.750207413	0.657702	-10.2633	1.03E-24	8.59E-23	TRUE		Lhx1
Acsl1	63.84786	-2.038440086	0.490079	-4.15941	3.19E-05	0.000184	TRUE		Acsl1
Lsp1	62.9529	-2.793557569	0.796157	-3.5088	0.00045	0.001905	TRUE		Lsp1

Pank3	929.2764	-1.593655046	0.336167	-4.74067	2.13E-06	1.61E-05	TRUE	Pank3
Arrb1	1093.872	-2.794989985	0.36787	-7.59776	3.01E-14	8.25E-13	TRUE	Arrb1
Natd1	677.4871	-1.54989254	0.310468	-4.99211	5.97E-07	5.17E-06	TRUE	Natd1
Ywhah	1544.485	-1.723837319	0.388337	-4.43902	9.04E-06	5.93E-05	TRUE	Ywhah
Cacng2	289.9177	-2.923066804	0.465043	-6.28559	3.27E-10	5.15E-09	TRUE	Cacng2
Rab5c	1271.726	-1.019101502	0.315484	-3.23028	0.001237	0.004648	TRUE	Rab5c
Rnf145	1247.732	-1.366217653	0.338458	-4.0366	5.42E-05	0.000294	TRUE	Rnf145
Atp6v1e1	825.8411	-1.398090088	0.264148	-5.29283	1.20E-07	1.21E-06	TRUE	Atp6v1e1
Lhx9	3837.708	-4.470343137	0.288219	-15.5102	2.96E-54	2.28E-51	TRUE	Lhx9
Calm3	7523.012	-2.154919795	0.205419	-10.4903	9.57E-26	8.59E-24	TRUE	Calm3
Cops3	418.342	-1.140763486	0.405222	-2.81516	0.004875	0.015187	TRUE	Cops3
Ubb	6928.716	-1.289914588	0.330571	-3.90209	9.54E-05	0.000487	TRUE	Ubb
Cyb561	150.6686	-2.277560669	0.576993	-3.9473	7.90E-05	0.000411	TRUE	Cyb561
Sema6a	569.9499	-3.774456352	0.324578	-11.6288	2.94E-31	5.03E-29	TRUE	Sema6a
Fmc1	104.4799	-1.490738014	0.323771	-4.60429	4.14E-06	2.93E-05	TRUE	Fmc1
Akt3	1405.333	-1.222553007	0.243091	-5.02919	4.93E-07	4.36E-06	TRUE	Akt3
Rgs17	133.8599	-2.567124605	0.347441	-7.38866	1.48E-13	3.70E-12	TRUE	Rgs17
Hdac2	2589.155	-1.002344008	0.330562	-3.03224	0.002427	0.008376	TRUE	Hdac2
Clvs2	73.22002	-5.246570761	0.711114	-7.37796	1.61E-13	3.99E-12	TRUE	Clvs2
Lrp11	301.9334	-2.673016907	0.353581	-7.55985	4.04E-14	1.08E-12	TRUE	Lrp11
Aig1	81.4361	-2.121771356	0.564791	-3.75674	0.000172	0.00082	TRUE	Aig1
Grm1	69.67974	-2.378301021	0.839723	-2.83224	0.004622	0.014512	TRUE	Grm1
Wasf1	634.9512	-2.181979108	0.228041	-9.56834	1.09E-21	6.66E-20	TRUE	Wasf1
Gtf3c6	595.4528	-1.056597562	0.267521	-3.94959	7.83E-05	0.000408	TRUE	Gtf3c6
Fyn	1805.194	-1.325987036	0.185758	-7.13824	9.45E-13	2.14E-11	TRUE	Fyn
Prep	465.2952	-1.85586458	0.420835	-4.40995	1.03E-05	6.69E-05	TRUE	Prep
Arfgef3	316.4303	-2.371894673	0.434525	-5.45859	4.80E-08	5.25E-07	TRUE	Arfgef3
Hebp2	29.42477	-3.069311873	0.67342	-4.55779	5.17E-06	3.58E-05	TRUE	Hebp2
Fam184a	106.9616	-1.220676176	0.381415	-3.20039	0.001372	0.005088	TRUE	Fam184a
Gopc	323.8263	-1.221350121	0.371746	-3.28544	0.001018	0.003911	TRUE	Gopc
Vta1	345.4635	-1.501684835	0.224663	-6.68417	2.32E-11	4.35E-10	TRUE	Vta1
Pkib	6.832083	-3.088545033	1.254029	-2.4629	0.013782	0.036504	TRUE	Pkib
Serinc1	1679.531	-1.792126363	0.25813	-6.94273	3.85E-12	8.06E-11	TRUE	Serinc1
Rspo3	388.6666	-2.573434542	0.447916	-5.74535	9.17E-09	1.15E-07	TRUE	Rspo3
Mgat4c	33.05573	-3.806024892	0.899164	-4.23285	2.31E-05	0.000137	TRUE	Mgat4c
Lin7a	42.86827	-1.419741943	0.597398	-2.37654	0.017476	0.044436	TRUE	Lin7a
Sim1	37.333	-4.907481751	1.082197	-4.53474	5.77E-06	3.96E-05	TRUE	Sim1
Zwint	2113.486	-1.187456229	0.29677	-4.00127	6.30E-05	0.000337	TRUE	Zwint
Ube2d1	869.9657	-1.557845313	0.260133	-5.98864	2.12E-09	2.90E-08	TRUE	Ube2d1
Slc17a8	83.68937	-8.524130722	1.222093	-6.97502	3.06E-12	6.50E-11	TRUE	Slc17a8
Rhobtb1	267.2221	-1.440788547	0.263624	-5.46532	4.62E-08	5.08E-07	TRUE	Rhobtb1
Arid5b	267.0727	-3.451063069	0.382323	-9.02657	1.77E-19	8.73E-18	TRUE	Arid5b
Dusp6	114.7999	-3.527497531	0.580413	-6.07757	1.22E-09	1.75E-08	TRUE	Dusp6
Kitl	730.3671	-4.216289003	0.674375	-6.25214	4.05E-10	6.31E-09	TRUE	Kitl
Sgk1	104.6905	-3.761578601	0.470559	-7.99386	1.31E-15	4.25E-14	TRUE	Sgk1
Enpp3	23.06064	-4.620219137	1.176899	-3.92576	8.65E-05	0.000445	TRUE	Enpp3
Stx7	782.0108	-2.487554843	0.239659	-10.3796	3.07E-25	2.65E-23	TRUE	Stx7
Fgd6	92.22063	-1.689241271	0.369716	-4.56902	4.90E-06	3.42E-05	TRUE	Fgd6
Tmcc3	34.04993	-4.657387567	0.82331	-5.65691	1.54E-08	1.86E-07	TRUE	Tmcc3
Tcp11l2	62.68176	-1.723768969	0.388047	-4.44217	8.91E-06	5.86E-05	TRUE	Tcp11l2
Cry1	175.387	-1.258653786	0.409478	-3.0738	0.002113	0.007425	TRUE	Cry1
Btbd11	113.5848	-2.551511842	0.369833	-6.89909	5.23E-12	1.07E-10	TRUE	Btbd11
Vps26a	384.8014	-1.106189392	0.451084	-2.45229	0.014195	0.037378	TRUE	Vps26a
Fam241b	244.5366	-2.727911331	0.317649	-8.58783	8.86E-18	3.60E-16	TRUE	Fam241b
H2afy2	2164.113	-1.867646249	0.308065	-6.06251	1.34E-09	1.91E-08	TRUE	H2afy2
Npffr1	17.32426	-5.272436304	1.262068	-4.17762	2.95E-05	0.000171	TRUE	Npffr1
Sgpl1	811.3228	-1.190386538	0.268243	-4.43771	9.09E-06	5.97E-05	TRUE	Sgpl1
Pcbd1	60.63984	-1.962686002	0.527938	-3.71764	0.000201	0.00094	TRUE	Pcbd1
Unc5b	260.4364	-2.804257185	0.312261	-8.9805	2.70E-19	1.31E-17	TRUE	Unc5b
Slc29a3	143.71	-1.078394739	0.325687	-3.31114	0.000929	0.003612	TRUE	Slc29a3
Vsir	20.37108	-2.708981364	0.939063	-2.88477	0.003917	0.012615	TRUE	Vsir
Slc16a7	51.4573	-2.143702414	0.56509	-3.79356	0.000149	0.000719	TRUE	Slc16a7
Lrig3	51.80396	-2.72512863	0.507826	-5.36626	8.04E-08	8.38E-07	TRUE	Lrig3
Plek	23.68788	-2.817663976	0.719842	-3.91428	9.07E-05	0.000465	TRUE	Plek
Srgap1	1135.628	-3.080009835	0.299698	-10.2771	8.94E-25	7.54E-23	TRUE	Srgap1
Egfr	10.15247	-3.815066469	1.1861	-3.21648	0.001298	0.004841	TRUE	Egfr
Rab21	480.0832	-1.454386827	0.263096	-5.52798	3.24E-08	3.66E-07	TRUE	Rab21
Apc2	10393.12	-1.292569642	0.259467	-4.98164	6.30E-07	5.43E-06	TRUE	Apc2
Lgr5	3.86365	-4.092938181	1.721087	-2.37811	0.017402	0.044279	TRUE	Lgr5
Rab1a	1390.354	-1.589499904	0.401596	-3.95796	7.56E-05	0.000396	TRUE	Rab1a
Gamt	61.16785	-1.841150618	0.435233	-4.23026	2.33E-05	0.000139	TRUE	Gamt
Ptprr	19.45787	-6.423158785	1.404366	-4.57371	4.79E-06	3.35E-05	TRUE	Ptprr

Actr2	1610.234	-1.975236389	0.328609	-6.0109	1.85E-09	2.55E-08	TRUE	Actr2
Ptprb	70.72122	-1.465967942	0.461281	-3.17804	0.001483	0.00545	TRUE	Ptprb
Meis1	79.00349	-1.824184356	0.450458	-4.04962	5.13E-05	0.00028	TRUE	Meis1
Yeats4	1114.32	-1.159139519	0.304887	-3.80187	0.000144	0.000698	TRUE	Yeats4
Cobl	121.5199	-5.968441757	0.730384	-8.17165	3.04E-16	1.05E-14	TRUE	Cobl
Snrpd3	977.2461	-1.458493509	0.503161	-2.89866	0.003748	0.012152	TRUE	Snrpd3
Nav3	611.0717	-3.621162693	0.613259	-5.90479	3.53E-09	4.71E-08	TRUE	Nav3
Ddc	22.63212	-5.601345236	1.24133	-4.51237	6.41E-06	4.34E-05	TRUE	Ddc
Cpm	58.132	-3.662522679	0.575613	-6.36282	1.98E-10	3.20E-09	TRUE	Cpm
Osbpl8	585.327	-1.999692253	0.270909	-7.38141	1.57E-13	3.90E-12	TRUE	Osbpl8
Wif1	64.15305	-5.636117225	0.852722	-6.60956	3.85E-11	7.00E-10	TRUE	Wif1
Irak3	5.009035	-4.463050511	1.547193	-2.88461	0.003919	0.012619	TRUE	Irak3
Prmt2	1533.815	-2.427108744	0.217607	-11.1537	6.87E-29	8.90E-27	TRUE	Prmt2
Txnrd1	2137.924	-1.204027215	0.260682	-4.61875	3.86E-06	2.76E-05	TRUE	Txnrd1
Aldh1l2	252.7633	-1.986713238	0.394225	-5.03954	4.67E-07	4.14E-06	TRUE	Aldh1l2
Wdr82	2548.754	-1.418847346	0.281059	-5.04821	4.46E-07	3.97E-06	TRUE	Wdr82
Glyctk	165.3798	-4.820450458	0.441414	-10.9205	9.20E-28	1.00E-25	TRUE	Glyctk
Fbxw11	1049.038	-1.611907596	0.367149	-4.39034	1.13E-05	7.26E-05	TRUE	Fbxw11
Papolg	250.4547	-1.102599163	0.33025	-3.33868	0.000842	0.003314	TRUE	Papolg
Nsg2	5464.314	-2.135276262	0.214245	-9.96653	2.14E-23	1.53E-21	TRUE	Nsg2
Cpeb4	513.8995	-2.873730389	0.583557	-4.92451	8.46E-07	7.05E-06	TRUE	Cpeb4
Sptbn1	2174.711	-2.146459267	0.307149	-6.98834	2.78E-12	5.93E-11	TRUE	Sptbn1
Wdpcp	126.0973	-1.694120742	0.337638	-5.01756	5.23E-07	4.59E-06	TRUE	Wdpcp
Mdh1	1145.575	-1.121242714	0.281358	-3.98512	6.74E-05	0.000358	TRUE	Mdh1
Ccng1	263.3049	-1.93600765	0.318087	-6.0864	1.15E-09	1.66E-08	TRUE	Ccng1
Hcn2	70.5745	-1.50779995	0.41486	-3.63448	0.000279	0.001253	TRUE	Hcn2
Cyfip2	1550.34	-2.099590922	0.285043	-7.36588	1.76E-13	4.34E-12	TRUE	Cyfip2
Flt4	38.78228	-1.350260571	0.530354	-2.54596	0.010898	0.029985	TRUE	Flt4
Hnrnpab	5207.593	-1.534584458	0.427754	-3.58754	0.000334	0.001468	TRUE	Hnrnpab
Rasgef1c	54.66136	-1.681882609	0.56716	-2.96545	0.003022	0.010112	TRUE	Rasgef1c
Rnf130	1395.833	-1.41384697	0.278028	-5.08526	3.67E-07	3.31E-06	TRUE	Rnf130
Vdac1	1725.134	-1.393786228	0.38517	-3.61862	0.000296	0.001323	TRUE	Vdac1
Adcy1	367.311	-2.189080291	0.35868	-6.10316	1.04E-09	1.51E-08	TRUE	Adcy1
921536K21R	17.31655	-2.193625915	0.867676	-2.52816	0.011466	0.031289	TRUE	4921536K21Rik
Osbp2	75.66504	-2.323570971	0.50032	-4.64417	3.41E-06	2.47E-05	TRUE	Osbp2
Gabrg2	512.6715	-4.160000899	0.377326	-11.025	2.90E-28	3.45E-26	TRUE	Gabrg2
Patz1	907.4991	-1.092236219	0.353647	-3.08849	0.002012	0.007105	TRUE	Patz1
Rtn4	3235.857	-1.514602336	0.217931	-6.9499	3.66E-12	7.67E-11	TRUE	Rtn4
Dynll2	3832.721	-2.146842001	0.255496	-8.40263	4.37E-17	1.66E-15	TRUE	Dynll2
Xbp1	606.9336	-1.504493408	0.231693	-6.49348	8.39E-11	1.43E-09	TRUE	Xbp1
Rnf187	4927.306	-1.100866469	0.206216	-5.33842	9.38E-08	9.64E-07	TRUE	Rnf187
Galnt10	244.3261	-1.0334075	0.244585	-4.22514	2.39E-05	0.000142	TRUE	Galnt10
Bzw2	1876.895	-1.070428592	0.259827	-4.11977	3.79E-05	0.000215	TRUE	Bzw2
Nampt	523.194	-1.603361852	0.356061	-4.50305	6.70E-06	4.52E-05	TRUE	Nampt
Tspan13	1375.532	-1.939996761	0.318048	-6.0997	1.06E-09	1.55E-08	TRUE	Tspan13
Fam49a	524.0314	-2.180890757	0.327444	-6.66035	2.73E-11	5.07E-10	TRUE	Fam49a
Rgs9	65.19444	-1.677945546	0.532187	-3.15292	0.001616	0.005866	TRUE	Rgs9
Trib2	859.7534	-1.072387534	0.386307	-2.776	0.005503	0.016843	TRUE	Trib2
Lratd1	248.8158	-2.419833113	0.311249	-7.77459	7.57E-15	2.26E-13	TRUE	Lratd1
Prkar1a	3246.341	-1.546731604	0.262172	-5.89968	3.64E-09	4.84E-08	TRUE	Prkar1a
Fkbp1b	47.74575	-2.244424474	0.462603	-4.85173	1.22E-06	9.84E-06	TRUE	Fkbp1b
Cmpk2	44.9775	-4.169340394	0.762178	-5.4703	4.49E-08	4.95E-07	TRUE	Cmpk2
Mboat2	701.1851	-1.062465723	0.218382	-4.86517	1.14E-06	9.26E-06	TRUE	Mboat2
Klf11	215.9912	-1.862806639	0.357506	-5.21055	1.88E-07	1.81E-06	TRUE	Klf11
Efr3b	2139.932	-2.227444951	0.198912	-11.1982	4.16E-29	5.63E-27	TRUE	Efr3b
Cbll1	240.5661	-1.527824069	0.551784	-2.76888	0.005625	0.017168	TRUE	Cbll1
Kif3c	1096.981	-1.822079357	0.194024	-9.39102	5.94E-21	3.42E-19	TRUE	Kif3c
Sh3yl1	131.5145	-2.983086192	0.388899	-7.6706	1.71E-14	4.82E-13	TRUE	Sh3yl1
Rab10	1267.535	-1.569452654	0.338938	-4.6305	3.65E-06	2.62E-05	TRUE	Rab10
Ace	13.05911	-3.644123884	1.011967	-3.60103	0.000317	0.001403	TRUE	Ace
Rasl10b	463.2622	-1.791205568	0.521347	-3.43573	0.000591	0.002424	TRUE	Rasl10b
Tmem132e	75.19651	-2.051341519	0.457105	-4.48768	7.20E-06	4.82E-05	TRUE	Tmem132e
Asic2	92.66243	-2.667674809	0.509153	-5.23943	1.61E-07	1.57E-06	TRUE	Asic2
Pecam1	148.6723	-1.252660145	0.328778	-3.81005	0.000139	0.000679	TRUE	Pecam1
Cep112	257.0129	-1.236273161	0.337666	-3.66123	0.000251	0.001146	TRUE	Cep112
Sumo2	1779.53	-1.56587946	0.500194	-3.13054	0.001745	0.00627	TRUE	Sumo2
Pafah1b1	2584.886	-1.479085413	0.275592	-5.36694	8.01E-08	8.36E-07	TRUE	Pafah1b1
Camkk1	223.5131	-1.75754866	0.342966	-5.12455	2.98E-07	2.74E-06	TRUE	Camkk1
Ube2g1	797.8189	-1.739057656	0.349041	-4.98239	6.28E-07	5.41E-06	TRUE	Ube2g1
Mxra7	183.5043	-2.203709015	0.478128	-4.60904	4.05E-06	2.87E-05	TRUE	Mxra7
Rflnb	60.24086	-1.13247891	0.442993	-2.55643	0.010575	0.029202	TRUE	Rflnb
Doc2b	18.06427	-4.060474524	1.039205	-3.90729	9.33E-05	0.000477	TRUE	Doc2b

Ywhae	7383.153	-1.092007722	0.330989	-3.29923	0.00097	0.003751	TRUE	Ywhae
Spag9	3075.853	-1.286285458	0.247269	-5.20197	1.97E-07	1.88E-06	TRUE	Spag9
Cacna1g	1947.599	-2.719396649	0.279975	-9.71299	2.65E-22	1.72E-20	TRUE	Cacna1g
230052G05F	63.05033	-2.297130279	0.421773	-5.44636	5.14E-08	5.60E-07	TRUE	A230052G05Rik
Vamp2	1244.059	-1.783184077	0.259287	-6.87725	6.10E-12	1.24E-10	TRUE	Vamp2
Ntn1	216.5843	-4.020840414	0.448855	-8.95799	3.31E-19	1.57E-17	TRUE	Ntn1
Adam11	550.2096	-1.327884117	0.265351	-5.00426	5.61E-07	4.89E-06	TRUE	Adam11
Ccdc103	8.540886	-2.550056896	1.077847	-2.36588	0.017987	0.04547	TRUE	Ccdc103
Klhl28	252.0146	-1.129542044	0.380804	-2.9662	0.003015	0.010092	TRUE	Klhl28
Klhdc2	1478.99	-1.135264305	0.263333	-4.31114	1.62E-05	0.000101	TRUE	Klhdc2
Sec23a	623.8856	-1.84104935	0.322757	-5.70414	1.17E-08	1.44E-07	TRUE	Sec23a
Psma6	829.0342	-1.444682892	0.381391	-3.78794	0.000152	0.000733	TRUE	Psma6
Nova1	1732.913	-1.657159161	0.32175	-5.15046	2.60E-07	2.42E-06	TRUE	Nova1
Akap5	253.4069	-1.401009595	0.31017	-4.51691	6.27E-06	4.26E-05	TRUE	Akap5
Rab15	195.7444	-3.144380241	0.344933	-9.11591	7.80E-20	4.05E-18	TRUE	Rab15
Fut8	342.9214	-2.604368688	0.316785	-8.22124	2.01E-16	7.13E-15	TRUE	Fut8
Atl1	439.375	-2.549664876	0.259505	-9.82512	8.78E-23	5.95E-21	TRUE	Atl1
Trim9	544.8991	-2.422630328	0.296317	-8.1758	2.94E-16	1.02E-14	TRUE	Trim9
Rtn1	5402.921	-1.526388199	0.31165	-4.89777	9.69E-07	7.98E-06	TRUE	Rtn1
Clmn	58.2083	-2.210689823	0.563578	-3.9226	8.76E-05	0.00045	TRUE	Clmn
Six6	7.015049	-4.946209163	1.846692	-2.67842	0.007397	0.021545	TRUE	Six6
Prkch	60.32966	-2.915914616	0.682591	-4.27183	1.94E-05	0.000118	TRUE	Prkch
Hif1a	893.715	-1.047338792	0.333657	-3.13897	0.001695	0.006117	TRUE	Hif1a
Snapc1	318.1716	-1.051611741	0.244	-4.30988	1.63E-05	0.000101	TRUE	Snapc1
Atp6v1d	552.6855	-1.093616437	0.225944	-4.84021	1.30E-06	1.04E-05	TRUE	Atp6v1d
Vti1b	553.0715	-1.023442181	0.229144	-4.46637	7.96E-06	5.28E-05	TRUE	Vti1b
Susd6	346.1303	-1.189191583	0.346393	-3.43307	0.000597	0.002445	TRUE	Susd6
Pfkp	902.9938	-1.644370169	0.495113	-3.3212	0.000896	0.003498	TRUE	Pfkp
Unc79	500.3211	-2.295255942	0.420251	-5.46163	4.72E-08	5.17E-07	TRUE	Unc79
Dpf3	166.4547	-4.944840499	0.523579	-9.4443	3.58E-21	2.09E-19	TRUE	Dpf3
Acot3	3.621585	-3.993194654	1.664798	-2.39861	0.016458	0.042264	TRUE	Acot3
Fam161b	134.024	-1.538338469	0.309457	-4.97109	6.66E-07	5.71E-06	TRUE	Fam161b
Tmed10	986.7865	-1.038017943	0.254388	-4.08046	4.49E-05	0.00025	TRUE	Tmed10
Gpatch2l	253.5442	-1.279644449	0.27911	-4.58473	4.55E-06	3.19E-05	TRUE	Gpatch2l
Esrrb	8.403484	-3.540372635	1.223784	-2.89297	0.003816	0.012326	TRUE	Esrrb
Vash1	696.6688	-2.075450772	0.250998	-8.2688	1.35E-16	4.85E-15	TRUE	Vash1
Evl	3144.919	-1.495228288	0.208242	-7.18023	6.96E-13	1.59E-11	TRUE	Evl
Degs2	49.01341	-1.259840497	0.425528	-2.96065	0.00307	0.010239	TRUE	Degs2
Cdc42bpb	1174.961	-1.094011761	0.245804	-4.45076	8.56E-06	5.65E-05	TRUE	Cdc42bpb
Ppp1r13b	254.0228	-2.434921646	0.493119	-4.93779	7.90E-07	6.65E-06	TRUE	Ppp1r13b
Klc1	4417.888	-1.475593811	0.166042	-8.88687	6.29E-19	2.89E-17	TRUE	Klc1
Hecw1	667.342	-1.772355089	0.399207	-4.43969	9.01E-06	5.92E-05	TRUE	Hecw1
Ggps1	480.3831	-1.340480736	0.24963	-5.36987	7.88E-08	8.23E-07	TRUE	Ggps1
Gng4	53.69066	-4.157465558	0.646289	-6.43283	1.25E-10	2.09E-09	TRUE	Gng4
Amph	204.3567	-1.064995064	0.27644	-3.85254	0.000117	0.000581	TRUE	Amph
Gpld1	7.928217	-3.031425358	1.244069	-2.4367	0.014822	0.038727	TRUE	Gpld1
Exoc2	392.9959	-1.348654606	0.344595	-3.91374	9.09E-05	0.000466	TRUE	Exoc2
Gcnt2	53.65398	-1.616351853	0.442264	-3.65472	0.000257	0.001172	TRUE	Gcnt2
Kif13a	410.9234	-1.136698777	0.28023	-4.05631	4.99E-05	0.000273	TRUE	Kif13a
Spin1	2184.539	-1.535798067	0.310162	-4.9516	7.36E-07	6.24E-06	TRUE	Spin1
Pxdc1	68.86086	-1.890044866	0.761906	-2.48068	0.013113	0.034946	TRUE	Pxdc1
Ptch1	567.2766	-3.487358369	0.597686	-5.83476	5.39E-09	7.02E-08	TRUE	Ptch1
Ctsl	271.4197	-1.165251891	0.382212	-3.0487	0.002298	0.007995	TRUE	Ctsl
Drd1	8.052094	-3.343162272	1.287569	-2.59649	0.009418	0.026498	TRUE	Drd1
Ptdss1	369.5699	-1.213263438	0.329772	-3.67909	0.000234	0.001077	TRUE	Ptdss1
700001L19R	7.023326	-3.266795321	1.255065	-2.60289	0.009244	0.026083	TRUE	1700001L19Rik
Trpc7	6.735274	-4.894006933	1.565965	-3.12523	0.001777	0.006366	TRUE	Trpc7
Hnrnpk	6227.612	-1.19220337	0.395954	-3.01097	0.002604	0.008903	TRUE	Hnrnpk
210016F16R	292.3364	-1.005991532	0.260691	-3.85895	0.000114	0.000569	TRUE	2210016F16Rik
Golm1	596.7671	-1.679814862	0.460909	-3.64457	0.000268	0.001213	TRUE	Golm1
Agtpbp1	487.107	-1.476818128	0.248731	-5.93741	2.90E-09	3.92E-08	TRUE	Agtpbp1
Tppp	88.86514	-2.900901644	0.464448	-6.24591	4.21E-10	6.54E-09	TRUE	Tppp
Rhobtb3	778.9632	-1.051250502	0.304413	-3.45337	0.000554	0.002286	TRUE	Rhobtb3
Spata9	10.83466	-3.36174455	1.036829	-3.24233	0.001186	0.004476	TRUE	Spata9
Glrx	68.13151	-4.568726286	0.617979	-7.39302	1.44E-13	3.59E-12	TRUE	Glrx
Hapln1	21.22782	-2.62445733	0.933772	-2.8106	0.004945	0.015372	TRUE	Hapln1
Vcan	1391.908	-1.018995762	0.287719	-3.54164	0.000398	0.001707	TRUE	Vcan
Atg10	49.32911	-1.301845813	0.418305	-3.11219	0.001857	0.00662	TRUE	Atg10
Ocln	6.18033	-4.769432523	1.528232	-3.12088	0.001803	0.006451	TRUE	Ocln
Serf1	222.7835	-1.379304334	0.253909	-5.43227	5.56E-08	6.03E-07	TRUE	Serf1
Cartpt	17.16521	-6.239306228	1.302465	-4.79038	1.66E-06	1.29E-05	TRUE	Cartpt
Ankra2	248.6934	-1.767914582	0.434272	-4.07099	4.68E-05	0.000259	TRUE	Ankra2

Arhgef28	56.73642	-1.389402022	0.470844	-2.95088	0.003169	0.010504	TRUE		Arhgef28
Hmgcr	1931.696	-1.662888131	0.239549	-6.94175	3.87E-12	8.10E-11	TRUE		Hmgcr
Scamp1	608.1712	-2.352120093	0.346195	-6.79421	1.09E-11	2.14E-10	TRUE		Scamp1
Pde4d	280.2328	-1.311853009	0.39918	-3.28637	0.001015	0.0039	TRUE		Pde4d
Rab3c	2742.58	-3.452388508	0.238693	-14.4637	2.05E-47	1.13E-44	TRUE		Rab3c
Plk2	468.7423	-2.122815646	0.321925	-6.59414	4.28E-11	7.68E-10	TRUE		Plk2
Nln	425.3863	-2.289638075	0.231638	-9.88454	4.86E-23	3.43E-21	TRUE		Nln
Trim23	253.5299	-1.081500277	0.399893	-2.70447	0.006841	0.020189	TRUE		Trim23
Parp8	221.6796	-1.184545291	0.388462	-3.04932	0.002294	0.007984	TRUE		Parp8
Fgf10	23.08835	-6.668111336	1.282549	-5.19911	2.00E-07	1.91E-06	TRUE		Fgf10
Ptprg	683.7681	-1.030849044	0.259805	-3.96778	7.25E-05	0.000382	TRUE		Ptprg
Map3k1	334.8967	-2.210585893	0.317433	-6.96395	3.31E-12	6.99E-11	TRUE		Map3k1
Il6st	288.2601	-1.108820882	0.388909	-2.85111	0.004357	0.01378	TRUE		Il6st
Plpp1	39.37937	-1.74239476	0.562606	-3.09701	0.001955	0.00693	TRUE		Plpp1
Ndufs4	500.4603	-1.071541081	0.365577	-2.93109	0.003378	0.011099	TRUE		Ndufs4
Samd8	461.3592	-1.323979368	0.3773	-3.50909	0.00045	0.001904	TRUE		Samd8
Vdac2	902.3633	-1.202833087	0.37272	-3.22718	0.00125	0.004686	TRUE		Vdac2
Ap3m1	573.1928	-1.063537465	0.416628	-2.55273	0.010688	0.029487	TRUE		Ap3m1
Ero1l	192.1123	-1.338665661	0.350319	-3.82128	0.000133	0.000653	TRUE		Ero1l
Otx2	528.4772	-5.86994585	2.272542	-2.58299	0.009795	0.027403	TRUE		Otx2
Arf4	1693.233	-1.499318711	0.466363	-3.21492	0.001305	0.004864	TRUE		Arf4
Sh3bp5	806.5743	-3.006325491	0.292595	-10.2747	9.16E-25	7.67E-23	TRUE		Sh3bp5
Capn7	568.5634	-1.202954464	0.248658	-4.83779	1.31E-06	1.05E-05	TRUE		Capn7
Arhgef3	17.31489	-2.819923683	0.799772	-3.52591	0.000422	0.001799	TRUE		Arhgef3
Ebpl	146.2656	-1.537604337	0.308881	-4.97798	6.43E-07	5.52E-06	TRUE		Ebpl
Kpna3	753.3981	-1.172889848	0.374396	-3.13275	0.001732	0.006228	TRUE		Kpna3
Spryd7	147.1822	-1.516639299	0.629907	-2.40772	0.016053	0.041385	TRUE		Spryd7
Mapk8	1321.504	-1.785146175	0.200745	-8.89262	5.97E-19	2.75E-17	TRUE		Mapk8
Pspc1	799.4131	-1.196730299	0.284633	-4.20447	2.62E-05	0.000154	TRUE		Pspc1
Gdf10	5.945058	-4.701262946	1.744722	-2.69456	0.007048	0.020685	TRUE		Gdf10
Zmym2	1225.705	-1.06066964	0.282106	-3.75983	0.00017	0.000811	TRUE		Zmym2
Micu2	443.1181	-1.008333454	0.224732	-4.48682	7.23E-06	4.84E-05	TRUE		Micu2
Fgf9	37.64873	-5.440195322	1.128392	-4.82119	1.43E-06	1.13E-05	TRUE		Fgf9
Extl3	1342.817	-1.421889382	0.257839	-5.51464	3.49E-08	3.92E-07	TRUE		Extl3
Atp8a2	324.8553	-2.576832484	0.297174	-8.67113	4.28E-18	1.78E-16	TRUE		Atp8a2
Amer2	2807.898	-2.461692325	0.215233	-11.4373	2.72E-30	4.26E-28	TRUE		Amer2
Mtmr6	685.3728	-1.593119048	0.216573	-7.35605	1.89E-13	4.65E-12	TRUE		Mtmr6
Cacna2d3	142.4662	-4.297183058	0.503701	-8.53122	1.45E-17	5.75E-16	TRUE		Cacna2d3
Wnt5a	135.5161	-3.376314534	0.465288	-7.2564	3.98E-13	9.38E-12	TRUE		Wnt5a
Lcp1	79.85364	-1.63598548	0.53875	-3.03663	0.002392	0.008285	TRUE		Lcp1
Gpalpp1	328.7917	-1.434823962	0.291219	-4.92697	8.35E-07	6.97E-06	TRUE		Gpalpp1
Akap11	1095.694	-1.366632714	0.281165	-4.86061	1.17E-06	9.45E-06	TRUE		Akap11
Trim35	2866.535	-1.605697909	0.262444	-6.11826	9.46E-10	1.39E-08	TRUE		Trim35
Stmn4	1267.388	-2.400428457	0.388225	-6.18309	6.29E-10	9.54E-09	TRUE		Stmn4
Dpysl2	5320.98	-1.928630215	0.330378	-5.83765	5.29E-09	6.90E-08	TRUE		Dpysl2
Nefm	1225.426	-4.100368851	1.001682	-4.09348	4.25E-05	0.000237	TRUE		Nefm
Rhobtb2	1033.242	-1.685246308	0.253339	-6.65215	2.89E-11	5.32E-10	TRUE		Rhobtb2
Klhl1	12.35602	-5.767873564	1.391907	-4.14386	3.42E-05	0.000195	TRUE		Klhl1
Dmtn	383.4836	-2.858444095	0.542083	-5.27307	1.34E-07	1.34E-06	TRUE		Dmtn
Gfra2	49.0621	-3.395672368	0.839125	-4.04668	5.19E-05	0.000283	TRUE		Gfra2
Rb1	486.4155	-1.056463698	0.421688	-2.50532	0.012234	0.033051	TRUE		Rb1
Uchl3	330.113	-1.448388704	0.276003	-5.24772	1.54E-07	1.51E-06	TRUE		Uchl3
Spry2	126.059	-1.214314347	0.383612	-3.16548	0.001548	0.00566	TRUE		Spry2
Ttc33	168.9625	-1.672539137	0.547317	-3.05589	0.002244	0.007837	TRUE		Ttc33
Rem2	407.0781	-3.874828333	0.422308	-9.17535	4.50E-20	2.39E-18	TRUE		Rem2
Slc7a8	181.8859	-3.667692188	1.417702	-2.58707	0.00968	0.027143	TRUE		Slc7a8
Psmb5	547.0524	-1.080621647	0.443246	-2.43797	0.01477	0.038615	TRUE		Psmb5
Sub1	1184.865	-1.550996028	0.442949	-3.50152	0.000463	0.001953	TRUE		Sub1
Jph4	1006.151	-1.539309616	0.370157	-4.15853	3.20E-05	0.000184	TRUE		Jph4
Emc9	46.92168	-2.07074076	0.461268	-4.48924	7.15E-06	4.79E-05	TRUE		Emc9
Sdr39u1	65.92608	-1.623605681	0.416886	-3.8946	9.84E-05	0.000501	TRUE		Sdr39u1
Skor1	16.90908	-4.582550511	1.181869	-3.87738	0.000106	0.000532	TRUE		Skor1
Dnah5	16.38142	-6.171615898	1.305818	-4.72625	2.29E-06	1.72E-05	TRUE		Dnah5
Trio	2125.631	-1.274250882	0.236482	-5.38835	7.11E-08	7.51E-07	TRUE		Trio
11-Mar	45.86943	-2.420590148	0.552172	-4.38376	1.17E-05	7.45E-05	TRUE		11-Mar
Retreg1	42.19106	-2.953676992	0.572604	-5.15833	2.49E-07	2.33E-06	TRUE		Retreg1
Rnf19a	513.9044	-1.540915058	0.25544	-6.03239	1.62E-09	2.26E-08	TRUE		Rnf19a
Ywhaz	7068.099	-1.974561856	0.479985	-4.1138	3.89E-05	0.000219	TRUE		Ywhaz
Rrm2b	102.2581	-1.57853619	0.409105	-3.85851	0.000114	0.000569	TRUE		Rrm2b
Baalc	20.61616	-2.906419703	0.868759	-3.34548	0.000821	0.003247	TRUE		Baalc
Lrp12	612.6488	-1.970134737	0.36178	-5.44566	5.16E-08	5.62E-07	TRUE		Lrp12
Zfpm2	158.833	-1.964243103	0.498592	-3.93958	8.16E-05	0.000423	TRUE		Zfpm2

Oxr1	342.3358	-1.063012416	0.374106	-2.84147	0.004491	0.014141	TRUE	Oxr1
Csmd3	35.34638	-3.368229698	0.788829	-4.26991	1.96E-05	0.000119	TRUE	Csmd3
Eif3h	2290.293	-1.031250905	0.280417	-3.67757	0.000235	0.001083	TRUE	Eif3h
Ebag9	189.4392	-1.400816955	0.305405	-4.58675	4.50E-06	3.16E-05	TRUE	Ebag9
Myc	107.7015	-1.123126938	0.448224	-2.50573	0.01222	0.03303	TRUE	Myc
Sqle	1186.443	-1.368695604	0.24238	-5.64691	1.63E-08	1.95E-07	TRUE	Sqle
Mtss1	2263.421	-1.312152925	0.195966	-6.69583	2.14E-11	4.04E-10	TRUE	Mtss1
Fbxo32	124.1365	-2.603047069	0.371118	-7.01408	2.31E-12	5.01E-11	TRUE	Fbxo32
Zhx1	430.935	-1.668569609	0.246042	-6.78164	1.19E-11	2.32E-10	TRUE	Zhx1
Derl1	737.5597	-1.266546656	0.206756	-6.1258	9.02E-10	1.33E-08	TRUE	Derl1
Fam49b	743.0223	-1.654266252	0.353101	-4.68497	2.80E-06	2.07E-05	TRUE	Fam49b
Rbx1	1405.636	-1.084920596	0.350933	-3.09153	0.001991	0.00704	TRUE	Rbx1
Syngr1	335.175	-1.979965931	0.3178	-6.23023	4.66E-10	7.19E-09	TRUE	Syngr1
Cacna1i	73.44854	-2.673093044	0.707849	-3.77636	0.000159	0.000765	TRUE	Cacna1i
Nptxr	520.1578	-4.077775116	0.435041	-9.37332	7.03E-21	4.02E-19	TRUE	Nptxr
Enpp2	102.8295	-3.513936804	0.568686	-6.17905	6.45E-10	9.77E-09	TRUE	Enpp2
Parvb	180.6318	-3.750739327	0.391952	-9.56939	1.08E-21	6.61E-20	TRUE	Parvb
Parvg	6.913505	-3.244191362	1.25689	-2.58113	0.009848	0.027525	TRUE	Parvg
Ttll1	518.9784	-1.432408935	0.253727	-5.64548	1.65E-08	1.97E-07	TRUE	Ttll1
Nell2	884.7554	-2.7927874	0.311464	-8.96665	3.06E-19	1.47E-17	TRUE	Nell2
3-Sep	5989.416	-1.487965619	0.173049	-8.59851	8.08E-18	3.31E-16	TRUE	3-Sep
Rapgef3	190.3952	-1.41546684	0.487857	-2.9014	0.003715	0.012065	TRUE	Rapgef3
Shisa9	113.8465	-1.888421188	0.559817	-3.37328	0.000743	0.002964	TRUE	Shisa9
310013L24R	629.4187	-1.164602007	0.200901	-5.79689	6.76E-09	8.67E-08	TRUE	1810013L24Rik
Zfp251	620.554	-1.086233852	0.258996	-4.19402	2.74E-05	0.00016	TRUE	Zfp251
Apod	43.78396	-1.98184928	0.527793	-3.75497	0.000173	0.000825	TRUE	Apod
Ly6h	1388.753	-1.435507093	0.420565	-3.41328	0.000642	0.002608	TRUE	Ly6h
Lynx1	34.99797	-2.44547291	0.575295	-4.25082	2.13E-05	0.000128	TRUE	Lynx1
Mapk8ip2	1767.705	-1.456930855	0.194106	-7.50584	6.10E-14	1.59E-12	TRUE	Mapk8ip2
Acr	50.2801	-1.992029929	0.525983	-3.78725	0.000152	0.000735	TRUE	Acr
Shank3	505.6623	-1.659688212	0.258323	-6.42485	1.32E-10	2.20E-09	TRUE	Shank3
Yaf2	278.8549	-1.749384063	0.479081	-3.65154	0.000261	0.001185	TRUE	Yaf2
Tagln3	2077.665	-1.813642941	0.31689	-5.72326	1.05E-08	1.30E-07	TRUE	Tagln3
Cd200	540.7968	-3.925098832	0.594156	-6.60618	3.94E-11	7.16E-10	TRUE	Cd200
Ube2v2	218.8944	-2.047859503	0.395363	-5.17969	2.22E-07	2.10E-06	TRUE	Ube2v2
Snai2	3.494031	-3.944849705	1.694524	-2.328	0.019912	0.049466	TRUE	Snai2
Crybg3	95.32182	-1.50268359	0.500403	-3.00295	0.002674	0.009112	TRUE	Crybg3
Cldnd1	331.6132	-1.461604062	0.523356	-2.79276	0.005226	0.016145	TRUE	Cldnd1
Tomm70a	1126.898	-1.734284799	0.399805	-4.33783	1.44E-05	9.05E-05	TRUE	Tomm70a
Tfg	414.3409	-1.138382655	0.405713	-2.80588	0.005018	0.015571	TRUE	Tfg
P2rx6	11.861	-2.494854206	1.07359	-2.32384	0.020134	0.049905	TRUE	P2rx6
Ncam2	35.43662	-2.434800868	0.625383	-3.89329	9.89E-05	0.000503	TRUE	Ncam2
Aifm3	130.2927	-5.237364237	0.603459	-8.67891	4.00E-18	1.68E-16	TRUE	Aifm3
Snap29	267.3336	-1.827324827	0.375906	-4.86112	1.17E-06	9.42E-06	TRUE	Snap29
Ncbp2	726.1084	-1.487152059	0.538865	-2.75978	0.005784	0.017581	TRUE	Ncbp2
Pak2	1105.871	-1.05043797	0.296457	-3.54331	0.000395	0.001698	TRUE	Pak2
Spag6l	13.50709	-3.194950344	1.025896	-3.1143	0.001844	0.006575	TRUE	Spag6l
Fgd4	714.1121	-1.139261434	0.245233	-4.64562	3.39E-06	2.46E-05	TRUE	Fgd4
Fyttd1	747.7324	-1.233365202	0.294621	-4.18627	2.84E-05	0.000165	TRUE	Fyttd1
Lrch3	189.9954	-1.01086472	0.392374	-2.57628	0.009987	0.027836	TRUE	Lrch3
Snx4	456.2999	-1.062406749	0.297288	-3.57366	0.000352	0.001534	TRUE	Snx4
Gsk3b	2618.738	-1.67326298	0.253934	-6.58936	4.42E-11	7.92E-10	TRUE	Gsk3b
Itgb5	80.70527	-2.068475054	0.39621	-5.22066	1.78E-07	1.72E-06	TRUE	Itgb5
Stxbp5l	25.02612	-4.1483982	0.92865	-4.46713	7.93E-06	5.26E-05	TRUE	Stxbp5l
Mylk	115.2521	-2.788872582	0.357899	-7.79234	6.58E-15	1.98E-13	TRUE	Mylk
Adcy5	537.9465	-1.732863228	0.310153	-5.58712	2.31E-08	2.70E-07	TRUE	Adcy5
Ap2m1	3279.35	-1.48181873	0.297999	-4.97257	6.61E-07	5.67E-06	TRUE	Ap2m1
Pdia5	58.3828	-1.354615522	0.386767	-3.5024	0.000461	0.001947	TRUE	Pdia5
Slc49a4	384.2904	-1.259878161	0.255591	-4.92927	8.25E-07	6.91E-06	TRUE	Slc49a4
Cxadr	1736.021	-1.90853931	0.31845	-5.99322	2.06E-09	2.83E-08	TRUE	Cxadr
Robo1	1580.512	-4.544122491	0.248698	-18.2717	1.39E-74	2.90E-71	TRUE	Robo1
St6gal1	886.5711	-2.034510242	0.31506	-6.45754	1.06E-10	1.78E-09	TRUE	St6gal1
App	2145.701	-1.732066035	0.271952	-6.36902	1.90E-10	3.08E-09	TRUE	App
Ets2	105.4176	-2.395548202	0.57613	-4.158	3.21E-05	0.000185	TRUE	Ets2
Kpna1	859.9748	-1.635105995	0.289362	-5.65072	1.60E-08	1.92E-07	TRUE	Kpna1
Dop1b	568.8586	-1.39037012	0.24622	-5.64686	1.63E-08	1.95E-07	TRUE	Dop1b
Rcan1	459.2478	-1.097052434	0.279474	-3.92542	8.66E-05	0.000446	TRUE	Rcan1
Ifngr2	310.3048	-1.519033965	0.329395	-4.61159	4.00E-06	2.84E-05	TRUE	Ifngr2
Zfp641	93.31336	-1.134602708	0.391642	-2.89704	0.003767	0.0122	TRUE	Zfp641
Faim2	47.26032	-2.199909006	0.472634	-4.65457	3.25E-06	2.37E-05	TRUE	Faim2
Prr13	404.1005	-1.116500546	0.282796	-3.94807	7.88E-05	0.00041	TRUE	Prr13
Cdkn1a	87.87453	-1.847789317	0.380488	-4.85637	1.20E-06	9.62E-06	TRUE	Cdkn1a

Nus1	516.7916	-1.476857597	0.396369	-3.72597	0.000195	0.000914	TRUE	Nus1
Mospd1	193.957	-1.126859482	0.413086	-2.72791	0.006374	0.01906	TRUE	Mospd1
Akirin1	594.0069	-1.765815468	0.369379	-4.7805	1.75E-06	1.35E-05	TRUE	Akirin1
Grm2	125.1747	-4.087117573	1.47909	-2.76327	0.005723	0.017423	TRUE	Grm2
Zfp605	193.3306	-1.115256511	0.382143	-2.91843	0.003518	0.011522	TRUE	Zfp605
Ache	78.64298	-2.197245612	0.457884	-4.7987	1.60E-06	1.25E-05	TRUE	Ache
Tmem176a	91.68515	-2.634874205	0.558811	-4.71514	2.42E-06	1.81E-05	TRUE	Tmem176a
Dlx2	192.4414	-2.970946783	0.589251	-5.0419	4.61E-07	4.09E-06	TRUE	Dlx2
Celsr3	3185.547	-1.406939871	0.306728	-4.58693	4.50E-06	3.16E-05	TRUE	Celsr3
Mpc1	66.94541	-1.05879176	0.388029	-2.72864	0.00636	0.019034	TRUE	Mpc1
Pde10a	789.8208	-1.291678811	0.247809	-5.21239	1.86E-07	1.80E-06	TRUE	Pde10a
Paqr4	389.8731	-2.649378014	0.264607	-10.0125	1.34E-23	9.93E-22	TRUE	Paqr4
Flywch2	163.2883	-1.003178486	0.275585	-3.64018	0.000272	0.001229	TRUE	Flywch2
Tnfrsf21	566.7311	-2.627047089	0.31107	-8.4452	3.04E-17	1.17E-15	TRUE	Tnfrsf21
Slc5a7	3.883246	-4.100087064	1.728726	-2.37174	0.017705	0.044935	TRUE	Slc5a7
Nfkbie	59.76601	-1.225891394	0.485699	-2.52398	0.011604	0.03161	TRUE	Nfkbie
Clic5	6.262752	-4.783805785	1.490148	-3.21029	0.001326	0.004935	TRUE	Clic5
Enpp5	215.6211	-2.71849808	0.414833	-6.55324	5.63E-11	9.90E-10	TRUE	Enpp5
Fbxl17	275.9093	-1.69056764	0.390368	-4.33071	1.49E-05	9.30E-05	TRUE	Fbxl17
Gm20517	7.025433	-3.132736767	1.303539	-2.40325	0.01625	0.041803	TRUE	Gm20517
Glp1r	6.039459	-4.736868627	1.561214	-3.03409	0.002413	0.008335	TRUE	Glp1r
Abcg1	269.4576	-1.933560552	0.294004	-6.57664	4.81E-11	8.56E-10	TRUE	Abcg1
Rsph1	6.832262	-4.90842562	1.477439	-3.32225	0.000893	0.003488	TRUE	Rsph1
Slc37a1	190.0661	-2.154746141	0.299439	-7.19595	6.20E-13	1.43E-11	TRUE	Slc37a1
Arhgap28	128.1728	-3.992012961	0.462299	-8.63514	5.87E-18	2.42E-16	TRUE	Arhgap28
Emilin2	21.18529	-3.304091992	0.856922	-3.85577	0.000115	0.000575	TRUE	Emilin2
Lbh	1449.362	-3.283209214	0.368085	-8.9197	4.68E-19	2.18E-17	TRUE	Lbh
Ehd3	125.6345	-1.132550344	0.314196	-3.6046	0.000313	0.001387	TRUE	Ehd3
Spast	1794.045	-1.559854777	0.224039	-6.96241	3.34E-12	7.06E-11	TRUE	Spast
Yipf4	194.6306	-1.067927808	0.429331	-2.48742	0.012867	0.034383	TRUE	Yipf4
Crim1	258.7259	-2.416925423	0.347574	-6.9537	3.56E-12	7.49E-11	TRUE	Crim1
Strn	278.9591	-1.280197487	0.316679	-4.04257	5.29E-05	0.000287	TRUE	Strn
Pja2	1064.532	-2.379347582	0.265825	-8.95081	3.53E-19	1.68E-17	TRUE	Pja2
Qpct	16.03163	-2.534897799	0.796428	-3.18283	0.001458	0.005365	TRUE	Qpct
Man2a1	194.2027	-2.129807125	0.3228	-6.59791	4.17E-11	7.52E-10	TRUE	Man2a1
Cyp1b1	34.02418	-7.228275959	1.398193	-5.16973	2.34E-07	2.20E-06	TRUE	Cyp1b1
Vapa	969.7459	-1.605455628	0.431747	-3.71851	0.0002	0.000938	TRUE	Vapa
Twsg1	769.1945	-1.104489713	0.346612	-3.18653	0.00144	0.005306	TRUE	Twsg1
Nrxn1	1233.893	-3.074528846	0.449885	-6.83404	8.26E-12	1.65E-10	TRUE	Nrxn1
Atp6v0c	1058.27	-1.915328441	0.355196	-5.39232	6.96E-08	7.35E-07	TRUE	Atp6v0c
Prepl	494.7978	-1.681046838	0.246419	-6.8219	8.98E-12	1.78E-10	TRUE	Prepl
Abca3	609.6794	-1.436272502	0.250993	-5.72236	1.05E-08	1.30E-07	TRUE	Abca3
Epas1	146.4525	-1.339609038	0.411456	-3.25578	0.001131	0.004289	TRUE	Epas1
Cript	435.7378	-1.005881826	0.374903	-2.68305	0.007295	0.021303	TRUE	Cript
St6gal2	312.0094	-3.458005793	0.369418	-9.36068	7.92E-21	4.52E-19	TRUE	St6gal2
Dusp1	351.3156	-1.92501312	0.366877	-5.24703	1.55E-07	1.52E-06	TRUE	Dusp1
Mllt1	1979.803	-1.024433688	0.199339	-5.13916	2.76E-07	2.56E-06	TRUE	Mllt1
Nudt3	2060.355	-1.08218151	0.197975	-5.46625	4.60E-08	5.05E-07	TRUE	Nudt3
Cul2	363.9618	-1.251136824	0.379303	-3.29851	0.000972	0.003758	TRUE	Cul2
Sos1	633.6228	-1.205634514	0.289902	-4.15877	3.20E-05	0.000184	TRUE	Sos1
Syt4	427.0885	-1.259949755	0.282954	-4.45284	8.47E-06	5.60E-05	TRUE	Syt4
Tpgs2	506.9364	-1.332000203	0.289631	-4.59896	4.25E-06	3.00E-05	TRUE	Tpgs2
Slc39a6	810.7475	-1.670042576	0.382378	-4.36752	1.26E-05	7.98E-05	TRUE	Slc39a6
Zfp397	607.9509	-1.16903589	0.322599	-3.62381	0.00029	0.0013	TRUE	Zfp397
Mapre2	1498.931	-3.122019025	0.204945	-15.2334	2.12E-52	1.47E-49	TRUE	Mapre2
Ccny	644.7536	-1.024380313	0.325561	-3.14651	0.001652	0.005975	TRUE	Ccny
Mib1	1218.596	-1.233567146	0.293474	-4.20332	2.63E-05	0.000155	TRUE	Mib1
Psd2	1007.572	-2.157724729	0.26197	-8.23653	1.77E-16	6.32E-15	TRUE	Psd2
Epb41l4a	355.915	-3.107395614	0.354992	-8.75343	2.07E-18	8.93E-17	TRUE	Epb41l4a
Npc1	338.7696	-1.035722671	0.341009	-3.03723	0.002388	0.008271	TRUE	Npc1
Zfp521	1057.828	-2.346073105	0.237374	-9.88343	4.91E-23	3.45E-21	TRUE	Zfp521
Ndfip1	1723.24	-2.342971394	0.328661	-7.12885	1.01E-12	2.29E-11	TRUE	Ndfip1
Spry4	18.24	-1.802200952	0.720971	-2.49969	0.01243	0.033495	TRUE	Spry4
Ap3s1	683.338	-1.229669625	0.446357	-2.7549	0.005871	0.017804	TRUE	Ap3s1
Dpysl3	12080.75	-3.149733314	0.248013	-12.6998	5.92E-37	1.49E-34	TRUE	Dpysl3
Jakmip2	829.2742	-1.34516001	0.287602	-4.67715	2.91E-06	2.15E-05	TRUE	Jakmip2
Sec11c	125.6	-1.089603212	0.284293	-3.83268	0.000127	0.000626	TRUE	Sec11c
Pmaip1	22.30896	-6.619241971	1.291346	-5.12585	2.96E-07	2.72E-06	TRUE	Pmaip1
Gnal	211.2977	-2.960891569	0.475108	-6.23203	4.60E-10	7.11E-09	TRUE	Gnal
Ldlrad4	32.8036	-1.197518699	0.492272	-2.43264	0.014989	0.039091	TRUE	Ldlrad4
Mapk4	161.896	-2.465193394	0.544472	-4.52768	5.96E-06	4.08E-05	TRUE	Mapk4
Smad2	898.8684	-1.514830971	0.287722	-5.26492	1.40E-07	1.39E-06	TRUE	Smad2

Grpel2	462.5849	-1.047061669	0.23708	-4.4165	1.00E-05	6.51E-05	TRUE	Grpel2
Fbn2	297.8003	-1.724257774	0.246088	-7.00666	2.44E-12	5.27E-11	TRUE	Fbn2
Slc27a6	10.97434	-3.93711095	1.187324	-3.31595	0.000913	0.003557	TRUE	Slc27a6
Isoc1	529.3294	-1.465149158	0.51345	-2.85354	0.004324	0.013698	TRUE	Isoc1
Csf1r	43.8049	-1.959438533	0.546429	-3.5859	0.000336	0.001476	TRUE	Csf1r
Gnaq	2443.068	-1.17242156	0.225151	-5.20726	1.92E-07	1.84E-06	TRUE	Gnaq
Tle4	806.9871	-1.756802906	0.217457	-8.07887	6.54E-16	2.19E-14	TRUE	Tle4
Cyb5a	265.3034	-1.122057661	0.361927	-3.10024	0.001934	0.006865	TRUE	Cyb5a
Cbln2	478.3081	-3.830401963	0.563997	-6.79153	1.11E-11	2.17E-10	TRUE	Cbln2
Rab3il1	200.3984	-1.135036583	0.25013	-4.53779	5.68E-06	3.91E-05	TRUE	Rab3il1
Rfk	311.5069	-1.075835196	0.401065	-2.68245	0.007309	0.021329	TRUE	Rfk
Tmem132a	3680.797	-3.039658311	0.2	-15.1983	3.63E-52	2.44E-49	TRUE	Tmem132a
Slc15a3	44.65616	-1.657398496	0.608451	-2.72397	0.00645	0.019256	TRUE	Slc15a3
Syt7	1942.621	-1.733342152	0.296	-5.85588	4.74E-09	6.22E-08	TRUE	Syt7
Zfand5	1508.335	-1.454413505	0.298889	-4.86606	1.14E-06	9.22E-06	TRUE	Zfand5
Cemip2	2316.767	-2.372405129	0.230866	-10.2761	9.03E-25	7.59E-23	TRUE	Cemip2
Rtn3	5061.709	-1.643684881	0.26445	-6.21549	5.12E-10	7.87E-09	TRUE	Rtn3
Lipo3	87.64704	-2.483905549	0.467078	-5.31797	1.05E-07	1.07E-06	TRUE	Lipo3
Ehd1	1294.391	-1.177613851	0.213136	-5.52517	3.29E-08	3.71E-07	TRUE	Ehd1
Ppp2r5b	1007.855	-1.001870134	0.226863	-4.41619	1.00E-05	6.51E-05	TRUE	Ppp2r5b
Lipa	94.56988	-1.462934228	0.379544	-3.85445	0.000116	0.000577	TRUE	Lipa
Jak2	522.1739	-1.224723288	0.211095	-5.80176	6.56E-09	8.45E-08	TRUE	Jak2
Htr7	4.305957	-4.237736537	1.652337	-2.56469	0.010327	0.028604	TRUE	Htr7
Tnks2	1999.503	-1.578312832	0.25266	-6.24679	4.19E-10	6.51E-09	TRUE	Tnks2
Pitpnm1	537.8249	-1.801398231	0.246265	-7.31489	2.58E-13	6.21E-12	TRUE	Pitpnm1
Cnih2	1996.98	-2.260188647	0.25556	-8.84406	9.23E-19	4.15E-17	TRUE	Cnih2
Apba1	380.1464	-3.576873843	0.361892	-9.8838	4.89E-23	3.45E-21	TRUE	Apba1
Peli3	79.98306	-1.794307051	0.525096	-3.4171	0.000633	0.002577	TRUE	Peli3
Smarca2	1101.12	-1.424887091	0.260404	-5.47184	4.45E-08	4.91E-07	TRUE	Smarca2
Vldlr	589.7935	-2.463224769	0.233804	-10.5354	5.93E-26	5.42E-24	TRUE	Vldlr
Capn1	22.62374	-3.590491024	0.987981	-3.63417	0.000279	0.001254	TRUE	Capn1
Vti1a	362.9953	-1.151231655	0.279142	-4.12418	3.72E-05	0.000211	TRUE	Vti1a
Tcf7l2	11648.09	-4.100920311	0.562128	-7.29534	2.98E-13	7.15E-12	TRUE	Tcf7l2
Hhex	8.604057	-2.560730693	1.028257	-2.49036	0.012761	0.034148	TRUE	Hhex
Tm9sf3	1310.035	-1.518638241	0.339625	-4.47151	7.77E-06	5.17E-05	TRUE	Tm9sf3
Lcor	282.4518	-1.069527328	0.455324	-2.34894	0.018827	0.04723	TRUE	Lcor
Slit1	785.0859	-2.034179359	0.344972	-5.89665	3.71E-09	4.93E-08	TRUE	Slit1
Mxi1	551.4263	-1.046440193	0.392357	-2.66706	0.007652	0.0222	TRUE	Mxi1
Fundc1	192.8481	-1.495171625	0.369111	-4.05074	5.11E-05	0.000279	TRUE	Fundc1
Gk	56.97649	-1.920881814	0.44747	-4.29276	1.76E-05	0.000109	TRUE	Gk
Gsto1	212.2056	-2.064509528	0.425943	-4.84692	1.25E-06	1.01E-05	TRUE	Gsto1
Habp2	5.696519	-4.641166862	1.57864	-2.93998	0.003282	0.010823	TRUE	Habp2
Gfra1	188.5722	-3.40098575	0.426439	-7.97531	1.52E-15	4.91E-14	TRUE	Gfra1
Hspa12a	271.1716	-1.422662319	0.345795	-4.11417	3.89E-05	0.000219	TRUE	Hspa12a
Slc18a2	230.7639	-3.455250712	0.716728	-4.82087	1.43E-06	1.13E-05	TRUE	Slc18a2
Btbd1	1001.168	-1.347650193	0.249878	-5.39323	6.92E-08	7.32E-07	TRUE	Btbd1
Hdgfl3	1730.542	-1.312368502	0.205525	-6.38544	1.71E-10	2.79E-09	TRUE	Hdgfl3
Tollip	939.3319	-1.147004718	0.3016	-3.80307	0.000143	0.000695	TRUE	Tollip
Pycr1	142.3397	-1.342964891	0.467971	-2.86976	0.004108	0.013118	TRUE	Pycr1
Rab40b	25.29003	-4.164557064	0.932003	-4.46839	7.88E-06	5.24E-05	TRUE	Rab40b
Entpd7	90.07856	-1.97900635	0.497906	-3.97466	7.05E-05	0.000373	TRUE	Entpd7
Cutc	33.68216	-1.173258191	0.47375	-2.47653	0.013266	0.035315	TRUE	Cutc
Scd3	7.353809	-3.358101769	1.398944	-2.40045	0.016375	0.042078	TRUE	Scd3
Scd2	6225.414	-1.036183636	0.419817	-2.46818	0.01358	0.036029	TRUE	Scd2
Ndufb8	296.6882	-1.771263731	0.326475	-5.42542	5.78E-08	6.23E-07	TRUE	Ndufb8
Sfxn3	638.8054	-2.125248264	0.21913	-9.69857	3.06E-22	1.95E-20	TRUE	Sfxn3
Kcnip2	56.19284	-3.076170084	0.577876	-5.32323	1.02E-07	1.04E-06	TRUE	Kcnip2
Actr1a	2787.19	-1.078322126	0.248259	-4.34353	1.40E-05	8.84E-05	TRUE	Actr1a
Sacm1l	80.96188	-2.745794917	0.524056	-5.2395	1.61E-07	1.57E-06	TRUE	Sacm1l
Fam120c	69.83914	-1.079741995	0.422072	-2.5582	0.010522	0.029077	TRUE	Fam120c
Gnl3l	798.0663	-1.792182695	0.299677	-5.98038	2.23E-09	3.05E-08	TRUE	Gnl3l
Maged2	1165.743	-1.292623807	0.175341	-7.37208	1.68E-13	4.15E-12	TRUE	Maged2
Alas2	44.51777	-1.098508902	0.446728	-2.45901	0.013932	0.036808	TRUE	Alas2
Acot9	74.39078	-1.986942395	0.425961	-4.66461	3.09E-06	2.27E-05	TRUE	Acot9
Jph3	542.1325	-2.850822771	0.252283	-11.3001	1.31E-29	1.83E-27	TRUE	Jph3
Kdm5c	1232.984	-1.03335373	0.272308	-3.7948	0.000148	0.000716	TRUE	Kdm5c
Gdf11	751.2691	-1.989465913	0.226844	-8.7702	1.78E-18	7.78E-17	TRUE	Gdf11
Dnajc14	648.323	-1.615382341	0.376003	-4.29619	1.74E-05	0.000107	TRUE	Dnajc14
Cdh9	6.072151	-4.743004828	1.573035	-3.01519	0.002568	0.0088	TRUE	Cdh9
Rnf41	834.4243	-1.048842628	0.270554	-3.87664	0.000106	0.000533	TRUE	Rnf41
Aatk	526.6128	-1.775648326	0.2462	-7.21222	5.50E-13	1.28E-11	TRUE	Aatk
Agap2	1985.429	-2.871437673	0.473189	-6.06827	1.29E-09	1.85E-08	TRUE	Agap2

Usp33	230.6384	-1.502478913	0.403776	-3.72107	0.000198	0.00093	TRUE	Usp33
Caly	106.621	-3.28507748	0.426843	-7.69622	1.40E-14	4.01E-13	TRUE	Caly
Adgra1	54.51751	-4.215743868	0.648993	-6.49582	8.26E-11	1.41E-09	TRUE	Adgra1
Dpysl4	1732.2	-1.40980459	0.211086	-6.6788	2.41E-11	4.49E-10	TRUE	Dpysl4
Cd151	425.2324	-1.347215072	0.382125	-3.52559	0.000423	0.0018	TRUE	Cd151
Clybl	416.3446	-4.704194202	0.547892	-8.58598	9.01E-18	3.65E-16	TRUE	Clybl
Dock9	87.83363	-1.371924613	0.565554	-2.42581	0.015274	0.039715	TRUE	Dock9
Nptx1	117.746	-1.824171497	0.302939	-6.02158	1.73E-09	2.40E-08	TRUE	Nptx1
Pde8a	26.65675	-2.078855908	0.573221	-3.62662	0.000287	0.001288	TRUE	Pde8a
Dach2	87.57322	-5.045064855	0.703635	-7.17	7.50E-13	1.71E-11	TRUE	Dach2
Klhl4	17.16995	-3.174511787	0.974368	-3.25802	0.001122	0.004262	TRUE	Klhl4
Copg2	633.3673	-1.053233913	0.252525	-4.17081	3.04E-05	0.000175	TRUE	Copg2
Podxl	625.9323	-1.899375078	0.291012	-6.52679	6.72E-11	1.17E-09	TRUE	Podxl
Bach1	531.6961	-1.193396656	0.30937	-3.85751	0.000115	0.000571	TRUE	Bach1
Phf6	1034.343	-1.22769192	0.36089	-3.40184	0.000669	0.00271	TRUE	Phf6
Shisa5	185.1748	-1.916558739	0.285204	-6.71997	1.82E-11	3.46E-10	TRUE	Shisa5
Arhgef9	350.0559	-2.564234939	0.322564	-7.94953	1.87E-15	5.98E-14	TRUE	Arhgef9
Cnksr2	140.7476	-1.312786634	0.363619	-3.61033	0.000306	0.001362	TRUE	Cnksr2
Rps6ka6	448.53	-1.200690979	0.382067	-3.14262	0.001674	0.006047	TRUE	Rps6ka6
Tmem47	122.5429	-3.096118231	0.58645	-5.27942	1.30E-07	1.29E-06	TRUE	Tmem47
8-Mar	1080.939	-1.864685031	0.240194	-7.76325	8.28E-15	2.45E-13	TRUE	8-Mar
Jmjd8	246.8441	-1.06231967	0.390988	-2.71701	0.006587	0.019572	TRUE	Jmjd8
Fbxl16	992.6325	-1.543156438	0.271976	-5.67387	1.40E-08	1.70E-07	TRUE	Fbxl16
Sdc3	2859.609	-2.769135975	0.292587	-9.46432	2.95E-21	1.74E-19	TRUE	Sdc3
Gdap1	529.5967	-1.92895032	0.267755	-7.20415	5.84E-13	1.35E-11	TRUE	Gdap1
St8sia2	2439.201	-2.619798671	0.219132	-11.9553	6.09E-33	1.20E-30	TRUE	St8sia2
Dhtkd1	95.27242	-1.577803365	0.392544	-4.01944	5.83E-05	0.000314	TRUE	Dhtkd1
Fam20c	831.4261	-1.830229817	0.290674	-6.29651	3.04E-10	4.81E-09	TRUE	Fam20c
Prkar1b	479.8719	-3.519640556	0.317255	-11.094	1.34E-28	1.65E-26	TRUE	Prkar1b
Arl10	296.0852	-2.428636588	0.429002	-5.66113	1.50E-08	1.81E-07	TRUE	Arl10
333439L19R	1757.449	-1.4170099	0.239841	-5.90812	3.46E-09	4.63E-08	TRUE	4833439L19Rik
Snca	80.45656	-2.255103066	0.436302	-5.16868	2.36E-07	2.22E-06	TRUE	Snca
Alkbh8	567.8363	-1.51695108	0.254539	-5.9596	2.53E-09	3.44E-08	TRUE	Alkbh8
Sntg1	34.09887	-4.058046624	0.756028	-5.36758	7.98E-08	8.33E-07	TRUE	Sntg1
Stau2	569.9956	-1.274732626	0.2247	-5.67303	1.40E-08	1.70E-07	TRUE	Stau2
Tfap2b	195.1063	-6.591766609	0.746592	-8.82914	1.05E-18	4.71E-17	TRUE	Tfap2b
Slco5a1	501.9778	-2.706842923	0.272346	-9.93898	2.82E-23	2.01E-21	TRUE	Slco5a1
Idh1	2020.454	-2.190728007	0.260707	-8.40303	4.35E-17	1.66E-15	TRUE	Idh1
Klf7	1531.503	-1.299218967	0.32132	-4.04338	5.27E-05	0.000286	TRUE	Klf7
Adam23	361.4153	-1.375058396	0.33555	-4.09793	4.17E-05	0.000233	TRUE	Adam23
Nrp2	1683.947	-3.344004396	0.227088	-14.7256	4.42E-49	2.49E-46	TRUE	Nrp2
Maip1	130.1295	-1.096118052	0.346812	-3.16055	0.001575	0.005737	TRUE	Maip1
Coq10b	133.9726	-1.049234471	0.350943	-2.98976	0.002792	0.009453	TRUE	Coq10b
Slc39a10	857.2886	-2.566597387	0.429247	-5.9793	2.24E-09	3.07E-08	TRUE	Slc39a10
Slc40a1	39.64238	-2.763116562	0.67885	-4.07029	4.70E-05	0.000259	TRUE	Slc40a1
Kansl1l	176.6859	-1.043917245	0.307252	-3.39759	0.00068	0.002747	TRUE	Kansl1l
Ica1l	83.63217	-1.43829947	0.522862	-2.75082	0.005945	0.018001	TRUE	Ica1l
Als2	434.6782	-1.12385351	0.277574	-4.04884	5.15E-05	0.000281	TRUE	Als2
Col5a2	113.0198	-1.234024713	0.346104	-3.56547	0.000363	0.001578	TRUE	Col5a2
Chst10	530.7766	-1.834825332	0.241723	-7.5906	3.18E-14	8.66E-13	TRUE	Chst10
Ormdl1	240.3033	-1.203952222	0.38929	-3.09268	0.001984	0.007019	TRUE	Ormdl1
Inpp1	201.2433	-1.109365641	0.395977	-2.80159	0.005085	0.015753	TRUE	Inpp1
Nabp1	49.24226	-1.648833375	0.523653	-3.14871	0.00164	0.005936	TRUE	Nabp1
Tmeff2	93.50616	-2.821583483	0.749467	-3.76478	0.000167	0.000797	TRUE	Tmeff2
Mgat4a	177.0695	-3.157003593	0.322908	-9.77678	1.42E-22	9.34E-21	TRUE	Mgat4a
Coa5	393.2787	-1.108519291	0.406555	-2.72662	0.006399	0.019121	TRUE	Coa5
Inpp4a	750.3504	-1.255007366	0.247317	-5.0745	3.89E-07	3.49E-06	TRUE	Inpp4a
Cnga3	6.753579	-4.892850813	1.457722	-3.35651	0.000789	0.003135	TRUE	Cnga3
Sema4c	1211.477	-1.071741729	0.294344	-3.64112	0.000271	0.001225	TRUE	Sema4c
B3gat2	117.9325	-1.352398259	0.45415	-2.97786	0.002903	0.00978	TRUE	B3gat2
Agfg1	651.5454	-1.185315176	0.325082	-3.6462	0.000266	0.001206	TRUE	Agfg1
Sphkap	14.38654	-2.325990801	0.900228	-2.58378	0.009772	0.027358	TRUE	Sphkap
Ppm1f	546.1632	-1.10174176	0.300691	-3.66403	0.000248	0.001135	TRUE	Ppm1f
Igfbp5	907.3486	-1.716806592	0.395263	-4.34345	1.40E-05	8.84E-05	TRUE	Igfbp5
Tmem169	415.6857	-4.999089199	0.319978	-15.6232	5.06E-55	4.05E-52	TRUE	Tmem169
Ptprn	327.4813	-3.839969058	0.470064	-8.16903	3.11E-16	1.07E-14	TRUE	Ptprn
Dnpep	715.847	-1.13931713	0.179573	-6.3446	2.23E-10	3.58E-09	TRUE	Dnpep
Itm2c	988.9005	-1.876527254	0.309817	-6.0569	1.39E-09	1.97E-08	TRUE	Itm2c
Ecel1	472.6501	-6.429867781	0.629607	-10.2125	1.74E-24	1.44E-22	TRUE	Ecel1
Serpine2	81.31002	-3.177023349	0.467727	-6.79247	1.10E-11	2.16E-10	TRUE	Serpine2
Ngef	25.55267	-1.668569249	0.578065	-2.88647	0.003896	0.012558	TRUE	Ngef
Inpp5d	17.83559	-1.965470612	0.70249	-2.79786	0.005144	0.015918	TRUE	Inpp5d

Cln8	199.2468	-1.048478433	0.33905	-3.0924	0.001985	0.007024	TRUE	Cln8
Pam	884.8243	-1.182087245	0.185707	-6.36534	1.95E-10	3.16E-09	TRUE	Pam
Actr3	1590.15	-1.480405312	0.346475	-4.27277	1.93E-05	0.000118	TRUE	Actr3
Lypd1	28.25047	-4.450595261	0.938046	-4.74454	2.09E-06	1.58E-05	TRUE	Lypd1
Tmem163	317.7436	-3.294550472	0.46216	-7.12859	1.01E-12	2.29E-11	TRUE	Tmem163
Rgs2	712.9296	-3.977588774	0.368307	-10.7996	3.46E-27	3.64E-25	TRUE	Rgs2
Steap3	6.227995	-3.084988548	1.296032	-2.38033	0.017297	0.044062	TRUE	Steap3
Nek7	521.624	-2.587390568	0.388529	-6.65945	2.75E-11	5.10E-10	TRUE	Nek7
Nr5a2	108.1429	-6.357642885	1.692764	-3.75578	0.000173	0.000823	TRUE	Nr5a2
Cd55	8.848355	-5.287249917	1.496632	-3.53277	0.000411	0.001759	TRUE	Cd55
Pfkfb2	230.8216	-1.125564546	0.259532	-4.3369	1.45E-05	9.08E-05	TRUE	Pfkfb2
Arl8a	1228.789	-2.063486925	0.173859	-11.8688	1.72E-32	3.23E-30	TRUE	Arl8a
Lrrn2	544.0652	-3.016879455	0.340462	-8.86114	7.92E-19	3.59E-17	TRUE	Lrrn2
Syt2	54.24877	-1.50503554	0.471058	-3.19501	0.001398	0.005169	TRUE	Syt2
Atp2b4	419.1292	-1.33830137	0.253315	-5.28316	1.27E-07	1.27E-06	TRUE	Atp2b4
Xpr1	2530.065	-2.896203894	0.288855	-10.0265	1.17E-23	8.77E-22	TRUE	Xpr1
Glul	1226.042	-1.282268984	0.406708	-3.1528	0.001617	0.005866	TRUE	Glul
Rgs16	366.314	-1.597035215	0.305703	-5.22414	1.75E-07	1.69E-06	TRUE	Rgs16
Rnf2	910.2806	-1.809367555	0.459264	-3.93971	8.16E-05	0.000423	TRUE	Rnf2
Desi2	275.52	-1.214400436	0.464891	-2.61223	0.008995	0.025488	TRUE	Desi2
Opn3	20.02365	-2.56055635	0.76118	-3.36393	0.000768	0.003057	TRUE	Opn3
Rgs7	86.57567	-4.689756104	0.558515	-8.39683	4.59E-17	1.73E-15	TRUE	Rgs7
Atp1b1	340.8091	-4.293137959	0.495242	-8.66876	4.37E-18	1.82E-16	TRUE	Atp1b1
Kifap3	1110.109	-1.545394924	0.194229	-7.95657	1.77E-15	5.66E-14	TRUE	Kifap3
Astn1	1280.152	-1.184859285	0.213861	-5.54033	3.02E-08	3.43E-07	TRUE	Astn1
Ralgps2	1489.078	-1.046870566	0.180118	-5.81213	6.17E-09	7.98E-08	TRUE	Ralgps2
Smyd2	122.5309	-1.006718392	0.366402	-2.74758	0.006004	0.018146	TRUE	Smyd2
Esrrg	164.9579	-4.486012779	0.522592	-8.58415	9.15E-18	3.70E-16	TRUE	Esrrg
Bpnt1	172.2493	-1.0660284	0.450869	-2.36439	0.01806	0.04562	TRUE	Bpnt1
Lpgat1	943.4231	-1.453578891	0.26554	-5.47405	4.40E-08	4.86E-07	TRUE	Lpgat1
Lamb3	8.113894	-3.362118649	1.239321	-2.71287	0.00667	0.019765	TRUE	Lamb3
Phyh	110.942	-1.213302749	0.429297	-2.82625	0.00471	0.014739	TRUE	Phyh
Uhmk1	579.4536	-1.260549532	0.270607	-4.65822	3.19E-06	2.33E-05	TRUE	Uhmk1
Optn	21.52296	-3.133503601	0.750644	-4.17442	2.99E-05	0.000173	TRUE	Optn
Hsd17b7	135.8799	-1.420185626	0.476348	-2.98141	0.002869	0.009679	TRUE	Hsd17b7
Mgst3	146.2765	-2.396577695	0.569517	-4.20809	2.58E-05	0.000152	TRUE	Mgst3
Pip4k2a	170.0256	-1.259782132	0.291938	-4.31525	1.59E-05	9.90E-05	TRUE	Pip4k2a
Plxdc2	30.2512	-1.885753134	0.569819	-3.30939	0.000935	0.003631	TRUE	Plxdc2
Psmb7	575.7277	-1.702999874	0.413408	-4.11942	3.80E-05	0.000215	TRUE	Psmb7
Kif5c	6420.765	-1.688842017	0.239567	-7.04956	1.79E-12	3.93E-11	TRUE	Kif5c
Mindy3	229.2836	-1.616883182	0.328919	-4.91575	8.84E-07	7.35E-06	TRUE	Mindy3
Pfkfb3	468.2556	-1.671505893	0.232134	-7.20062	5.99E-13	1.38E-11	TRUE	Pfkfb3
Abi2	1229.638	-1.473563304	0.223642	-6.58892	4.43E-11	7.93E-10	TRUE	Abi2
Gad2	835.9466	-4.411222413	0.40156	-10.9852	4.50E-28	5.24E-26	TRUE	Gad2
Stxbp1	1408.465	-2.393747091	0.224406	-10.667	1.45E-26	1.41E-24	TRUE	Stxbp1
Ak1	275.0536	-1.782414342	0.244376	-7.29373	3.01E-13	7.23E-12	TRUE	Ak1
Olfm1	1395.586	-1.798183016	0.207014	-8.68628	3.74E-18	1.58E-16	TRUE	Olfm1
Gsn	96.58013	-3.495153414	0.470888	-7.42248	1.15E-13	2.91E-12	TRUE	Gsn
Ttll11	80.98285	-1.695494225	0.445122	-3.80905	0.00014	0.000681	TRUE	Ttll11
Mrrf	177.755	-1.136263537	0.269377	-4.21812	2.46E-05	0.000146	TRUE	Mrrf
Ndufa8	506.3766	-1.116567815	0.395237	-2.82506	0.004727	0.014781	TRUE	Ndufa8
Strbp	1715.24	-1.089889296	0.191965	-5.67754	1.37E-08	1.66E-07	TRUE	Strbp
Tmem141	63.9132	-1.333982769	0.398488	-3.34761	0.000815	0.003225	TRUE	Tmem141
Grin1	107.4962	-1.832430618	0.630392	-2.90681	0.003651	0.01188	TRUE	Grin1
Rbms1	185.0774	-1.441930223	0.396927	-3.63273	0.00028	0.00126	TRUE	Rbms1
Galnt3	9.304269	-5.358501594	1.411406	-3.79657	0.000147	0.000712	TRUE	Galnt3
Dusp19	60.1962	-1.482691598	0.396586	-3.73864	0.000185	0.000874	TRUE	Dusp19
Slc25a12	494.4595	-1.356882417	0.202721	-6.69334	2.18E-11	4.11E-10	TRUE	Slc25a12
Ube2e3	1532.4	-1.66445248	0.392043	-4.24558	2.18E-05	0.000131	TRUE	Ube2e3
Zfp385b	7.863381	-5.115929894	1.450741	-3.52642	0.000421	0.001796	TRUE	Zfp385b
Cers6	534.1954	-1.749810741	0.286702	-6.10324	1.04E-09	1.51E-08	TRUE	Cers6
Lrp2	25.38857	-6.80622029	1.306934	-5.20778	1.91E-07	1.83E-06	TRUE	Lrp2
Tfpi	28.25308	-1.616995222	0.596881	-2.70907	0.006747	0.01995	TRUE	Tfpi
Atf2	883.9728	-1.03781287	0.272087	-3.81427	0.000137	0.000669	TRUE	Atf2
Ola1	507.0353	-1.537294223	0.390313	-3.93861	8.20E-05	0.000424	TRUE	Ola1
Sp3	970.0738	-1.514572525	0.331277	-4.57192	4.83E-06	3.37E-05	TRUE	Sp3
Lin7c	1720.975	-2.193775204	0.338368	-6.4834	8.97E-11	1.51E-09	TRUE	Lin7c
230118H07F	190.0254	-1.254410248	0.344721	-3.63891	0.000274	0.001234	TRUE	B230118H07Rik
Tcp11l1	237.5185	-1.143592193	0.250233	-4.57011	4.87E-06	3.40E-05	TRUE	Tcp11l1
Hipk3	232.3496	-1.152658956	0.322142	-3.57811	0.000346	0.001514	TRUE	Hipk3
Caprin1	4830.765	-1.277150568	0.342406	-3.72993	0.000192	0.000902	TRUE	Caprin1
Cat	593.9411	-1.818979766	0.339879	-5.35184	8.71E-08	9.01E-07	TRUE	Cat

Pamr1	16.54778	-3.679046976	1.056318	-3.4829	0.000496	0.002076	TRUE	Pamr1
Trim44	1815.272	-1.590104174	0.280296	-5.67296	1.40E-08	1.70E-07	TRUE	Trim44
Gatm	43.6203	-1.193901889	0.447133	-2.67012	0.007582	0.022023	TRUE	Gatm
Syt13	5307.855	-4.885498317	1.519956	-3.21424	0.001308	0.004874	TRUE	Syt13
Chst1	1499.7	-3.924011234	0.341485	-11.491	1.46E-30	2.36E-28	TRUE	Chst1
Mapk8ip1	2501.045	-1.085644392	0.191705	-5.6631	1.49E-08	1.80E-07	TRUE	Mapk8ip1
Frmd5	730.8916	-1.466858312	0.234815	-6.24688	4.19E-10	6.51E-09	TRUE	Frmd5
Arhgap1	521.7256	-1.408850708	0.204087	-6.9032	5.08E-12	1.04E-10	TRUE	Arhgap1
Map1a	878.3012	-1.365036118	0.239331	-5.70354	1.17E-08	1.45E-07	TRUE	Map1a
Lamp5	3.644734	-4.004977408	1.66274	-2.40866	0.016011	0.041302	TRUE	Lamp5
Snap25	718.0065	-2.019088196	0.320279	-6.30416	2.90E-10	4.59E-09	TRUE	Snap25
Slx4ip	214.8822	-1.636772544	0.328974	-4.97539	6.51E-07	5.59E-06	TRUE	Slx4ip
Rassf2	695.2431	-1.415547459	0.28727	-4.92759	8.32E-07	6.96E-06	TRUE	Rassf2
Rasgrp1	47.36118	-2.797122318	0.516878	-5.41158	6.25E-08	6.68E-07	TRUE	Rasgrp1
Chgb	302.2896	-3.145853295	0.319143	-9.8572	6.38E-23	4.43E-21	TRUE	Chgb
Slc27a2	31.85722	-5.444696632	1.015763	-5.3602	8.31E-08	8.65E-07	TRUE	Slc27a2
Dusp2	13.99659	-2.815917695	1.058248	-2.66092	0.007793	0.022562	TRUE	Dusp2
Mall	3.934088	-4.105448373	1.759601	-2.33317	0.019639	0.048928	TRUE	Mall
Pdyn	24.25325	-4.686659065	1.187659	-3.94613	7.94E-05	0.000413	TRUE	Pdyn
Tgm6	5.583144	-4.611943426	1.591425	-2.898	0.003756	0.012176	TRUE	Tgm6
Otor	4.411874	-4.281640334	1.612987	-2.65448	0.007943	0.02294	TRUE	Otor
Pcsk2	346.9393	-4.048053502	0.438792	-9.22544	2.82E-20	1.52E-18	TRUE	Pcsk2
Bfsp1	24.45077	-2.700089522	0.854075	-3.16142	0.00157	0.005726	TRUE	Bfsp1
Dtd1	145.745	-2.511016141	0.616459	-4.07329	4.64E-05	0.000256	TRUE	Dtd1
Nkx2-2	53.35651	-7.873358676	1.24075	-6.34564	2.21E-10	3.55E-09	TRUE	Nkx2-2
Napb	618.8394	-1.478263716	0.275122	-5.37313	7.74E-08	8.10E-07	TRUE	Napb
Snph	161.8553	-1.573427824	0.486295	-3.23554	0.001214	0.004568	TRUE	Snph
Kif3b	592.959	-1.559465232	0.225209	-6.92453	4.37E-12	9.10E-11	TRUE	Kif3b
Mapre1	2539.184	-1.132484086	0.232402	-4.87296	1.10E-06	8.94E-06	TRUE	Mapre1
Pkia	1358.57	-2.666970924	0.342345	-7.7903	6.69E-15	2.01E-13	TRUE	Pkia
Stmn2	3285.69	-3.621964868	0.431172	-8.40028	4.45E-17	1.69E-15	TRUE	Stmn2
Zdbf2	988.7709	-1.734776833	0.32146	-5.39656	6.79E-08	7.20E-07	TRUE	Zdbf2
Phactr3	548.1617	-2.725773579	0.255341	-10.675	1.33E-26	1.30E-24	TRUE	Phactr3
Fabp5	1886.664	-1.3818514	0.351774	-3.92823	8.56E-05	0.000442	TRUE	Fabp5
Snx16	100.2939	-1.059098069	0.371643	-2.84977	0.004375	0.01383	TRUE	Snx16
Ptpn1	1320.895	-1.323503241	0.248858	-5.31831	1.05E-07	1.07E-06	TRUE	Ptpn1
Gid8	366.8973	-1.221462476	0.438282	-2.78693	0.005321	0.016379	TRUE	Gid8
Nkain4	192.7061	-1.494171157	0.502236	-2.97504	0.00293	0.009858	TRUE	Nkain4
Chrna4	666.015	-7.452650213	0.521491	-14.291	2.49E-46	1.21E-43	TRUE	Chrna4
Stmn3	2404.654	-2.56985135	0.241793	-10.6283	2.20E-26	2.09E-24	TRUE	Stmn3
Zgpat	502.6854	-1.439747197	0.400505	-3.59483	0.000325	0.001434	TRUE	Zgpat
Zbtb46	291.59	-2.33893561	0.523498	-4.4679	7.90E-06	5.25E-05	TRUE	Zbtb46
Oprl1	625.9386	-2.872621782	0.380576	-7.54809	4.42E-14	1.18E-12	TRUE	Oprl1
Armc1	511.0556	-1.089413773	0.322396	-3.37911	0.000727	0.002913	TRUE	Armc1
Mmp24	547.9578	-3.879137419	0.321197	-12.0771	1.40E-33	2.80E-31	TRUE	Mmp24
Epb41l1	1164.094	-2.364274312	0.363748	-6.49976	8.04E-11	1.38E-09	TRUE	Epb41l1
Rab5if	196.4106	-1.161457328	0.428609	-2.70983	0.006732	0.019911	TRUE	Rab5if
Skil	624.6801	-1.904356504	0.257139	-7.40595	1.30E-13	3.27E-12	TRUE	Skil
Gnb4	293.935	-1.710496114	0.237291	-7.20843	5.66E-13	1.31E-11	TRUE	Gnb4
Pex5l	65.58859	-6.019419675	0.889758	-6.76523	1.33E-11	2.58E-10	TRUE	Pex5l
Gm8325	32.38026	-1.250900065	0.496115	-2.52139	0.011689	0.031802	TRUE	Gm8325
Slc7a11	22.92094	-6.656244053	1.282482	-5.19013	2.10E-07	1.99E-06	TRUE	Slc7a11
Ppm1l	941.0999	-1.883726951	0.312114	-6.03538	1.59E-09	2.22E-08	TRUE	Ppm1l
Bche	21.114	-3.328903468	0.805496	-4.13274	3.58E-05	0.000204	TRUE	Bche
Dclk1	2304.727	-3.196067414	0.249739	-12.7977	1.69E-37	4.35E-35	TRUE	Dclk1
Nbea	1987.453	-1.722463129	0.357147	-4.82284	1.42E-06	1.12E-05	TRUE	Nbea
Pfn2	2356.287	-2.598642039	0.357209	-7.27486	3.47E-13	8.25E-12	TRUE	Pfn2
Slc33a1	99.29792	-1.295290551	0.497209	-2.60512	0.009184	0.025942	TRUE	Slc33a1
Ssr3	1228.509	-1.130881804	0.469066	-2.41092	0.015912	0.041123	TRUE	Ssr3
Serpini1	318.3024	-1.833279981	0.377905	-4.85117	1.23E-06	9.86E-06	TRUE	Serpini1
Sike1	869.3397	-1.115448839	0.260378	-4.28397	1.84E-05	0.000112	TRUE	Sike1
Vangl1	57.27165	-2.020294058	0.442828	-4.56226	5.06E-06	3.51E-05	TRUE	Vangl1
Gdap2	195.943	-1.219317491	0.287234	-4.24503	2.19E-05	0.000131	TRUE	Gdap2
Gstm4	52.37733	-1.79911496	0.425643	-4.22681	2.37E-05	0.000141	TRUE	Gstm4
Kcnc4	8.590068	-3.452278794	1.243275	-2.77676	0.00549	0.016816	TRUE	Kcnc4
Il6ra	4.089956	-4.168407835	1.612637	-2.58484	0.009742	0.027289	TRUE	Il6ra
Chrnb2	1013.106	-2.443333871	0.214232	-11.4051	3.94E-30	6.00E-28	TRUE	Chrnb2
Olfm3	8.286911	-5.191629846	1.441088	-3.60258	0.000315	0.001396	TRUE	Olfm3
Ndst3	345.7626	-1.888287623	0.380398	-4.96398	6.91E-07	5.89E-06	TRUE	Ndst3
Lef1	439.6459	-1.990634298	0.37053	-5.3724	7.77E-08	8.13E-07	TRUE	Lef1
Snx7	30.91736	-2.417765891	0.748772	-3.22897	0.001242	0.004667	TRUE	Snx7
Ints12	148.9772	-1.351259693	0.296467	-4.55788	5.17E-06	3.58E-05	TRUE	Ints12

Pitx2	23.1854	-5.704618615	1.21748	-4.68559	2.79E-06	2.06E-05	TRUE	Pitx2
Papss1	574.6655	-1.33269801	0.352183	-3.78411	0.000154	0.000744	TRUE	Papss1
Sema4a	502.8309	-3.454129685	0.386824	-8.92946	4.28E-19	2.01E-17	TRUE	Sema4a
Sh3d19	162.4744	-2.050425746	0.356148	-5.75723	8.55E-09	1.07E-07	TRUE	Sh3d19
Rnf115	466.1752	-1.242881993	0.345364	-3.59876	0.00032	0.001414	TRUE	Rnf115
Bcar3	50.6915	-1.400585279	0.452994	-3.09184	0.001989	0.007035	TRUE	Bcar3
Gclm	216.2755	-1.430792278	0.568794	-2.51548	0.011887	0.032256	TRUE	Gclm
Abca4	51.78523	-5.274916298	0.812432	-6.49275	8.43E-11	1.43E-09	TRUE	Abca4
Celf3	4428.119	-1.810811185	0.263571	-6.8703	6.41E-12	1.30E-10	TRUE	Celf3
Wls	272.3469	-2.234444841	0.324025	-6.89591	5.35E-12	1.09E-10	TRUE	Wls
Bcl10	185.9719	-1.238566287	0.335197	-3.69504	0.00022	0.001019	TRUE	Bcl10
Trp53inp1	503.801	-1.071627112	0.377927	-2.83554	0.004575	0.01438	TRUE	Trp53inp1
Calb1	354.7067	-6.381429362	1.260007	-5.0646	4.09E-07	3.67E-06	TRUE	Calb1
Faxc	596.2561	-1.863099215	0.445649	-4.18064	2.91E-05	0.000169	TRUE	Faxc
Sdcbp	745.9438	-1.660125082	0.398328	-4.16773	3.08E-05	0.000178	TRUE	Sdcbp
Tstd3	119.1072	-1.698730797	0.301297	-5.63806	1.72E-08	2.05E-07	TRUE	Tstd3
Spaca1	4.339855	-4.256824576	1.59532	-2.66832	0.007623	0.022132	TRUE	Spaca1
Pdlim5	81.79999	-2.351979417	0.455242	-5.16644	2.39E-07	2.24E-06	TRUE	Pdlim5
Rngtt	293.9072	-1.091441253	0.39216	-2.78315	0.005383	0.016535	TRUE	Rngtt
Rragd	481.9655	-2.167911619	0.411357	-5.27014	1.36E-07	1.36E-06	TRUE	Rragd
Akirin2	579.852	-1.226701538	0.40929	-2.99714	0.002725	0.009265	TRUE	Akirin2
C9orf72	301.6335	-1.673052486	0.262974	-6.36205	1.99E-10	3.21E-09	TRUE	C9orf72
Stra6l	18.99805	-2.285823912	0.849715	-2.69011	0.007143	0.020934	TRUE	Stra6l
Coro2a	44.07199	-2.598911518	0.571513	-4.54742	5.43E-06	3.74E-05	TRUE	Coro2a
Nr4a3	30.04295	-1.419946855	0.59843	-2.37279	0.017654	0.044819	TRUE	Nr4a3
Tmeff1	1830.26	-2.121875895	0.3616	-5.86801	4.41E-09	5.79E-08	TRUE	Tmeff1
Brinp1	296.9982	-5.47351588	0.595763	-9.1874	4.02E-20	2.15E-18	TRUE	Brinp1
Fmn2	485.9264	-1.714411588	0.361295	-4.74518	2.08E-06	1.58E-05	TRUE	Fmn2
Zfp618	1275.824	-1.287767409	0.380252	-3.38662	0.000708	0.002844	TRUE	Zfp618
Astn2	363.9972	-4.122710426	0.325561	-12.6634	9.43E-37	2.31E-34	TRUE	Astn2
Ugcg	498.7772	-1.373578709	0.357115	-3.84632	0.00012	0.000595	TRUE	Ugcg
Ptbp3	327.978	-1.09770105	0.401126	-2.73655	0.006209	0.018644	TRUE	Ptbp3
Hsdl2	119.6856	-1.249907971	0.471939	-2.64845	0.008086	0.023272	TRUE	Hsdl2
Snx30	432.3743	-1.465746748	0.407106	-3.60041	0.000318	0.001406	TRUE	Snx30
Zfp37	318.8569	-2.059136047	0.408063	-5.04612	4.51E-07	4.01E-06	TRUE	Zfp37
Dnaja1	3120.797	-1.488751819	0.266082	-5.59508	2.21E-08	2.58E-07	TRUE	Dnaja1
Tal2	101.0022	-7.137282392	0.967425	-7.37761	1.61E-13	4.00E-12	TRUE	Tal2
Chmp5	630.1966	-1.064727069	0.310654	-3.42737	0.000609	0.002493	TRUE	Chmp5
Rad23b	1658.619	-1.231563301	0.373574	-3.29671	0.000978	0.003776	TRUE	Rad23b
Epb41l4b	35.22445	-3.075933665	0.868611	-3.54121	0.000398	0.00171	TRUE	Epb41l4b
Dcaf12	691.172	-1.255080909	0.323682	-3.87751	0.000106	0.000532	TRUE	Dcaf12
Fam219a	508.6704	-2.195734625	0.216476	-10.1431	3.56E-24	2.85E-22	TRUE	Fam219a
Enho	653.1578	-1.032233322	0.37663	-2.74071	0.006131	0.018452	TRUE	Enho
Tesk1	323.3655	-1.476854887	0.259172	-5.69835	1.21E-08	1.49E-07	TRUE	Tesk1
Tpm2	80.62796	-1.499568424	0.44153	-3.3963	0.000683	0.002759	TRUE	Tpm2
Gne	229.1827	-1.051735623	0.288326	-3.64773	0.000265	0.001199	TRUE	Gne
Glipr2	343.3749	-2.748039206	0.264364	-10.3949	2.62E-25	2.27E-23	TRUE	Glipr2
Sh3gl2	72.43582	-1.370463205	0.4767	-2.8749	0.004042	0.012938	TRUE	Sh3gl2
Plin2	199.7728	-1.528573735	0.393358	-3.88596	0.000102	0.000516	TRUE	Plin2
Prkaa2	142.5428	-3.077319444	0.533135	-5.77212	7.83E-09	9.90E-08	TRUE	Prkaa2
Sgip1	363.7365	-1.944739171	0.438789	-4.43206	9.33E-06	6.11E-05	TRUE	Sgip1
Pde4b	387.9722	-1.856523548	0.353271	-5.25525	1.48E-07	1.46E-06	TRUE	Pde4b
Dnajc6	294.9831	-4.280261642	0.480623	-8.90565	5.31E-19	2.46E-17	TRUE	Dnajc6
Jak1	355.5891	-1.183347367	0.301334	-3.92703	8.60E-05	0.000444	TRUE	Jak1
Bend5	379.7205	-1.354855746	0.321148	-4.21878	2.46E-05	0.000145	TRUE	Bend5
Eps15	546.3098	-1.418837105	0.265721	-5.33958	9.32E-08	9.59E-07	TRUE	Eps15
Rnf11	684.0846	-1.541426199	0.33594	-4.58839	4.47E-06	3.14E-05	TRUE	Rnf11
Tm2d1	139.3906	-1.346195637	0.399678	-3.3682	0.000757	0.003014	TRUE	Tm2d1
Btf3l4	748.4504	-1.403745924	0.396777	-3.53787	0.000403	0.001729	TRUE	Btf3l4
Laptm5	73.552	-1.407793761	0.362275	-3.88598	0.000102	0.000516	TRUE	Laptm5
Dmrtb1	954.1326	-6.456948824	0.624673	-10.3365	4.82E-25	4.10E-23	TRUE	Dmrtb1
Tmem59	626.7215	-1.13833423	0.29637	-3.84093	0.000123	0.000607	TRUE	Tmem59
Exo5	291.3661	-1.487019555	0.425064	-3.49835	0.000468	0.001973	TRUE	Exo5
Ccdc30	186.4204	-2.141490222	0.446591	-4.7952	1.63E-06	1.27E-05	TRUE	Ccdc30
Ndufs5	259.3679	-1.032144298	0.285024	-3.62126	0.000293	0.001311	TRUE	Ndufs5
Mycl	2037.554	-2.701739273	0.348907	-7.74344	9.68E-15	2.83E-13	TRUE	Mycl
Ephb2	3635.568	-1.305071356	0.170224	-7.66681	1.76E-14	4.96E-13	TRUE	Ephb2
Rnf220	3256.971	-1.319666882	0.210795	-6.26043	3.84E-10	6.01E-09	TRUE	Rnf220
Plk3	324.0158	-1.79649152	0.270677	-6.63702	3.20E-11	5.86E-10	TRUE	Plk3
Ptch2	153.3014	-2.253233851	0.480781	-4.68661	2.78E-06	2.05E-05	TRUE	Ptch2
Pik3r3	1792.761	-1.736630917	0.179	-9.70185	2.96E-22	1.90E-20	TRUE	Pik3r3
Tal1	112.9509	-3.112937327	0.448515	-6.94054	3.91E-12	8.16E-11	TRUE	Tal1

Cmpk1	811.4703	-1.493729403	0.362928	-4.11578	3.86E-05	0.000218	TRUE	Cmpk1
Pla2g2f	5.669563	-4.635264376	1.574665	-2.94365	0.003244	0.010709	TRUE	Pla2g2f
Vwa5b1	11.1067	-5.608319767	1.379169	-4.06645	4.77E-05	0.000263	TRUE	Vwa5b1
Kif17	9.769709	-2.819710069	1.032721	-2.73037	0.006326	0.018948	TRUE	Kif17
Fabp3	87.7253	-5.296252892	0.678931	-7.80087	6.15E-15	1.86E-13	TRUE	Fabp3
Khdrbs1	3741.076	-1.419511419	0.372232	-3.81351	0.000137	0.000671	TRUE	Khdrbs1
Rnf19b	481.6469	-1.851783177	0.233984	-7.91415	2.49E-15	7.84E-14	TRUE	Rnf19b
Ccdc28b	339.1345	-1.004504036	0.28305	-3.54885	0.000387	0.001666	TRUE	Ccdc28b
Nipal3	219.0095	-2.106982365	0.353018	-5.96848	2.39E-09	3.27E-08	TRUE	Nipal3
Stmn1	20454.25	-1.503535337	0.289108	-5.2006	1.99E-07	1.90E-06	TRUE	Stmn1
Psmb2	490.9792	-1.100436429	0.385867	-2.85185	0.004347	0.013752	TRUE	Psmb2
Map3k6	5.709419	-4.652776075	1.528164	-3.04468	0.002329	0.00809	TRUE	Map3k6
Rspo1	59.90872	-3.08173526	0.872739	-3.53111	0.000414	0.001768	TRUE	Rspo1
Epha10	75.10742	-3.16637227	0.590376	-5.36332	8.17E-08	8.51E-07	TRUE	Epha10
Stx12	997.3662	-1.540093892	0.327193	-4.70699	2.51E-06	1.88E-05	TRUE	Stx12
Sesn2	125.7283	-1.229512696	0.296805	-4.14249	3.44E-05	0.000196	TRUE	Sesn2
Casp9	244.4598	-1.626187444	0.253956	-6.40342	1.52E-10	2.51E-09	TRUE	Casp9
Cdk14	414.2899	-1.729773011	0.259953	-6.65418	2.85E-11	5.26E-10	TRUE	Cdk14
Acot7	725.4098	-1.748349314	0.189057	-9.24775	2.29E-20	1.25E-18	TRUE	Acot7
Errfi1	137.3589	-2.128730508	0.344845	-6.173	6.70E-10	1.01E-08	TRUE	Errfi1
Slc25a33	47.78242	-2.101220888	0.580054	-3.62246	0.000292	0.001306	TRUE	Slc25a33
Klhl7	1221.951	-1.115430838	0.279941	-3.98452	6.76E-05	0.000359	TRUE	Klhl7
Draxin	1544.959	-1.895013914	0.268183	-7.06613	1.59E-12	3.51E-11	TRUE	Draxin
Trp73	53.13101	-2.675320817	1.052797	-2.54116	0.011049	0.030341	TRUE	Trp73
Lrrc47	504.7574	-1.627592556	0.353212	-4.60797	4.07E-06	2.88E-05	TRUE	Lrrc47
Tprgl	751.4649	-1.285072209	0.253008	-5.07919	3.79E-07	3.41E-06	TRUE	Tprgl
Ssu72	318.7114	-1.061270194	0.371369	-2.85773	0.004267	0.013556	TRUE	Ssu72
Prkcz	587.0778	-2.109538003	0.291609	-7.23413	4.69E-13	1.10E-11	TRUE	Prkcz
Prxl2b	114.4143	-1.900119415	0.344343	-5.5181	3.43E-08	3.86E-07	TRUE	Prxl2b
Gnb1	7026.763	-1.370422646	0.254478	-5.38523	7.24E-08	7.63E-07	TRUE	Gnb1
Cptp	159.0048	-1.059681791	0.348083	-3.04434	0.002332	0.008097	TRUE	Cptp
Cd38	5.554436	-4.616577004	1.618352	-2.85264	0.004336	0.013726	TRUE	Cd38
Sorcs2	636.7099	-3.672489807	0.652222	-5.63074	1.79E-08	2.13E-07	TRUE	Sorcs2
Afap1	1253.36	-1.504930615	0.237594	-6.33405	2.39E-10	3.80E-09	TRUE	Afap1
Ablim2	13.89603	-3.323171527	0.977677	-3.39905	0.000676	0.002734	TRUE	Ablim2
Ppp2r2c	127.5698	-2.191494258	0.759768	-2.88443	0.003921	0.012625	TRUE	Ppp2r2c
Crmp1	7957.773	-2.771429289	0.202977	-13.6539	1.91E-42	6.87E-40	TRUE	Crmp1
Stk32b	110.0683	-2.898421581	0.524041	-5.5309	3.19E-08	3.60E-07	TRUE	Stk32b
Nsg1	2433.294	-2.436262574	0.218658	-11.1419	7.84E-29	1.00E-26	TRUE	Nsg1
Rnf32	82.39789	-1.308617686	0.328456	-3.98415	6.77E-05	0.000359	TRUE	Rnf32
Dnajb6	1120.167	-1.54574691	0.270805	-5.70797	1.14E-08	1.41E-07	TRUE	Dnajb6
Slc30a3	31.33171	-7.109493226	1.283195	-5.54046	3.02E-08	3.43E-07	TRUE	Slc30a3
Ociad2	115.4202	-2.609023109	0.339765	-7.67891	1.60E-14	4.55E-13	TRUE	Ociad2
Cgref1	85.05527	-1.186173243	0.347958	-3.40896	0.000652	0.002646	TRUE	Cgref1
Dpysl5	5440.323	-2.329300657	0.162496	-14.3345	1.33E-46	6.77E-44	TRUE	Dpysl5
Tada2b	269.3774	-2.01222087	0.331613	-6.06797	1.30E-09	1.85E-08	TRUE	Tada2b
Apbb2	708.779	-2.005224979	0.258271	-7.76404	8.23E-15	2.44E-13	TRUE	Apbb2
Gnpda2	40.8004	-1.522936108	0.581518	-2.6189	0.008821	0.02508	TRUE	Gnpda2
Gabra4	204.705	-3.403850657	0.679776	-5.00731	5.52E-07	4.82E-06	TRUE	Gabra4
Gabrb1	271.5633	-3.080775139	0.424356	-7.25988	3.87E-13	9.15E-12	TRUE	Gabrb1
Slc10a4	86.88813	-6.03974745	1.688296	-3.57742	0.000347	0.001517	TRUE	Slc10a4
Lnx1	22.95372	-5.012181771	1.051669	-4.76593	1.88E-06	1.44E-05	TRUE	Lnx1
Chic2	294.0143	-1.295085496	0.366382	-3.53479	0.000408	0.001747	TRUE	Chic2
Pdgfra	40.65484	-7.485131951	1.298723	-5.76346	8.24E-09	1.04E-07	TRUE	Pdgfra
Pigg	82.67976	-1.165745893	0.460883	-2.52937	0.011427	0.031202	TRUE	Pigg
Dr1	359.5522	-1.326565882	0.521245	-2.54499	0.010928	0.030049	TRUE	Dr1
Dipk1a	129.8105	-1.413149377	0.403594	-3.50142	0.000463	0.001953	TRUE	Dipk1a
Brdt	19.47579	-2.096735212	0.708038	-2.96133	0.003063	0.010219	TRUE	Brdt
Rufy3	3459.261	-2.393790395	0.186873	-12.8097	1.45E-37	3.82E-35	TRUE	Rufy3
Sparcl1	103.9275	-1.36369208	0.363888	-3.74756	0.000179	0.000847	TRUE	Sparcl1
Hsd17b11	39.93751	-1.867860369	0.468881	-3.98366	6.79E-05	0.00036	TRUE	Hsd17b11
Klhl8	206.1147	-2.248284469	0.309134	-7.27284	3.52E-13	8.35E-12	TRUE	Klhl8
Prkg2	311.0906	-1.958923505	0.34778	-5.63265	1.77E-08	2.11E-07	TRUE	Prkg2
Bmp3	37.73209	-2.607547933	0.800838	-3.25602	0.00113	0.004287	TRUE	Bmp3
Fgf5	6.299385	-4.790599918	1.497909	-3.19819	0.001383	0.005123	TRUE	Fgf5
Antxr2	133.7116	-1.385570605	0.330198	-4.19618	2.71E-05	0.000159	TRUE	Antxr2
Tesc	36.59498	-2.391205314	0.63729	-3.75215	0.000175	0.000834	TRUE	Tesc
Tmed2	859.5952	-1.017895589	0.368869	-2.75951	0.005789	0.017586	TRUE	Tmed2
G3bp2	2278.281	-1.498750538	0.261485	-5.73168	9.94E-09	1.24E-07	TRUE	G3bp2
Ajm1	104.4715	-2.570150731	0.567731	-4.52705	5.98E-06	4.09E-05	TRUE	Ajm1
Rimbp2	85.33095	-4.023599626	0.79401	-5.06744	4.03E-07	3.62E-06	TRUE	Rimbp2
Scarb2	1160.306	-1.701476939	0.409496	-4.15505	3.25E-05	0.000187	TRUE	Scarb2

Vps33a	668.5406	-1.058629149	0.217933	-4.85758	1.19E-06	9.57E-06	TRUE	Vps33a
Arpc3	645.5645	-1.176648037	0.271379	-4.33581	1.45E-05	9.12E-05	TRUE	Arpc3
Rnf34	427.9428	-1.457524916	0.441559	-3.30086	0.000964	0.003733	TRUE	Rnf34
Pgam5	480.448	-1.118384504	0.391359	-2.8577	0.004267	0.013556	TRUE	Pgam5
Gpc2	486.1036	-1.806951496	0.312053	-5.79052	7.02E-09	8.94E-08	TRUE	Gpc2
Uncx	268.7146	-3.857759323	0.384598	-10.0306	1.12E-23	8.44E-22	TRUE	Uncx
Tes	102.9084	-1.222543435	0.354398	-3.44963	0.000561	0.002314	TRUE	Tes
Foxp2	1711.698	-1.470322667	0.31683	-4.64074	3.47E-06	2.51E-05	TRUE	Foxp2
Tmem106b	285.4568	-1.397973473	0.381128	-3.66799	0.000244	0.001119	TRUE	Tmem106b
Radil	77.46234	-2.889671701	0.522321	-5.53236	3.16E-08	3.58E-07	TRUE	Radil
Actb	20952.36	-1.491086458	0.30169	-4.94245	7.71E-07	6.50E-06	TRUE	Actb
Lhx5	477.2206	-5.025945021	0.427417	-11.7589	6.36E-32	1.12E-29	TRUE	Lhx5
Dtx1	749.0615	-1.648440642	0.330663	-4.98526	6.19E-07	5.34E-06	TRUE	Dtx1
Rph3a	220.9861	-2.959625666	0.632951	-4.67591	2.93E-06	2.16E-05	TRUE	Rph3a
Mtus2	272.9192	-2.668723595	0.460338	-5.79732	6.74E-09	8.65E-08	TRUE	Mtus2
Limk1	1214.388	-1.594686783	0.228848	-6.96831	3.21E-12	6.79E-11	TRUE	Limk1
Fezf1	141.6245	-6.715653603	1.724902	-3.89335	9.89E-05	0.000503	TRUE	Fezf1
Gcc1	227.6979	-1.238363563	0.390082	-3.17463	0.0015	0.005506	TRUE	Gcc1
Actl6b	934.3168	-1.165662375	0.426348	-2.73406	0.006256	0.018764	TRUE	Actl6b
Tfr2	30.98543	-1.84092884	0.545501	-3.37475	0.000739	0.002952	TRUE	Tfr2
Zkscan1	1259.049	-1.41833544	0.308737	-4.59399	4.35E-06	3.06E-05	TRUE	Zkscan1
Tpk1	19.27745	-2.189503581	0.892047	-2.45447	0.014109	0.037177	TRUE	Tpk1
Dlx6	233.6634	-1.38391493	0.563738	-2.45489	0.014093	0.037148	TRUE	Dlx6
Dlx5	145.0674	-2.897220048	0.370965	-7.80995	5.72E-15	1.74E-13	TRUE	Dlx5
Akr1b8	131.8206	-2.357688666	0.633241	-3.72321	0.000197	0.000923	TRUE	Akr1b8
Klhdc10	1781.203	-1.103375045	0.19403	-5.68662	1.30E-08	1.58E-07	TRUE	Klhdc10
Gars	1082.352	-1.012861422	0.229678	-4.40992	1.03E-05	6.69E-05	TRUE	Gars
Nt5c3	358.1082	-1.388019258	0.276265	-5.02422	5.05E-07	4.45E-06	TRUE	Nt5c3
Avl9	778.4502	-1.521348291	0.292474	-5.20165	1.98E-07	1.89E-06	TRUE	Avl9
Abcg2	119.8719	-1.274495425	0.451015	-2.82584	0.004716	0.014748	TRUE	Abcg2
Herc3	182.5132	-1.406177654	0.317286	-4.43189	9.34E-06	6.11E-05	TRUE	Herc3
Tmem176b	367.6286	-1.184067828	0.289892	-4.08452	4.42E-05	0.000246	TRUE	Tmem176b
Npy	39.63594	-4.070872668	0.777141	-5.23827	1.62E-07	1.58E-06	TRUE	Npy
Osbpl3	134.2808	-3.099068092	0.38004	-8.15458	3.50E-16	1.20E-14	TRUE	Osbpl3
921507P07R	32.01436	-1.534273361	0.578238	-2.65336	0.007969	0.022991	TRUE	4921507P07Rik
Cbx3	1410.54	-1.272615445	0.442529	-2.87578	0.00403	0.012914	TRUE	Cbx3
Ptn	5824.229	-1.265512285	0.37731	-3.35404	0.000796	0.003158	TRUE	Ptn
Mtpn	1337.459	-1.557696078	0.382946	-4.06766	4.75E-05	0.000262	TRUE	Mtpn
Fam131b	711.985	-1.488621843	0.287482	-5.17814	2.24E-07	2.11E-06	TRUE	Fam131b
Ccdc184	260.012	-4.784955068	0.479489	-9.97928	1.88E-23	1.37E-21	TRUE	Ccdc184
Qrfprl	8.418326	-5.215095201	1.468996	-3.55011	0.000385	0.00166	TRUE	Qrfprl
Hpgds	28.51653	-2.548532071	0.600618	-4.24318	2.20E-05	0.000132	TRUE	Hpgds
Mkrn1	2167.871	-1.742	0.183023	-9.51794	1.77E-21	1.06E-19	TRUE	Mkrn1
Slc37a3	613.4228	-1.211697128	0.234946	-5.15734	2.50E-07	2.34E-06	TRUE	Slc37a3
Gfpt1	762.2138	-1.756886953	0.337004	-5.21325	1.86E-07	1.79E-06	TRUE	Gfpt1
Pcyox1	965.6073	-1.292343909	0.277667	-4.6543	3.25E-06	2.37E-05	TRUE	Pcyox1
Add2	3757.941	-2.164383338	0.198786	-10.888	1.32E-27	1.42E-25	TRUE	Add2
Prickle2	230.7084	-5.243241346	0.613434	-8.54736	1.26E-17	5.04E-16	TRUE	Prickle2
700003E16R	14.68758	-3.48562252	1.009857	-3.4516	0.000557	0.002299	TRUE	1700003E16Rik
Cntn3	27.25086	-1.655447065	0.576203	-2.87303	0.004066	0.012999	TRUE	Cntn3
Chl1	2258.041	-3.835491309	0.323788	-11.8457	2.27E-32	4.18E-30	TRUE	Chl1
Plxna1	2269.082	-2.274706223	0.264974	-8.58464	9.11E-18	3.69E-16	TRUE	Plxna1
Chchd6	331.3788	-1.064981445	0.266186	-4.00089	6.31E-05	0.000337	TRUE	Chchd6
Aldh1l1	45.14908	-3.469056175	0.676902	-5.1249	2.98E-07	2.74E-06	TRUE	Aldh1l1
Slc41a3	10.66408	-2.714850101	1.1035	-2.46022	0.013885	0.036708	TRUE	Slc41a3
Cntn6	45.13422	-4.536774007	0.824136	-5.50489	3.69E-08	4.12E-07	TRUE	Cntn6
Grip2	82.64828	-1.305379995	0.47182	-2.76669	0.005663	0.017269	TRUE	Grip2
Ret	15.76098	-6.119192607	1.358984	-4.50277	6.71E-06	4.53E-05	TRUE	Ret
Zfp248	129.1918	-1.249763076	0.294178	-4.24832	2.15E-05	0.000129	TRUE	Zfp248
Gabarapl1	979.1344	-1.37050886	0.204883	-6.68922	2.24E-11	4.22E-10	TRUE	Gabarapl1
Ptpro	414.8613	-3.232775173	0.282029	-11.4625	2.03E-30	3.24E-28	TRUE	Ptpro
Strap	1367.702	-1.015562326	0.334477	-3.03627	0.002395	0.008292	TRUE	Strap
Slco1c1	27.05166	-2.662402793	0.732183	-3.63625	0.000277	0.001245	TRUE	Slco1c1
Kcnj8	36.29332	-1.998074674	0.663006	-3.01366	0.002581	0.008836	TRUE	Kcnj8
Abcc9	65.73689	-2.090203153	0.601519	-3.47488	0.000511	0.002131	TRUE	Abcc9
Sspn	22.23711	-2.929157058	0.772385	-3.79235	0.000149	0.000722	TRUE	Sspn
Bhlhe41	14.70053	-3.185543226	0.986608	-3.22878	0.001243	0.004669	TRUE	Bhlhe41
Kras	992.6765	-1.131312677	0.364544	-3.10336	0.001913	0.006801	TRUE	Kras
Cmas	1129.414	-1.541451015	0.208567	-7.39069	1.46E-13	3.65E-12	TRUE	Cmas
St8sia1	542.6871	-2.889690831	0.262457	-11.0101	3.41E-28	4.04E-26	TRUE	St8sia1
Ccdc91	258.2661	-1.314410213	0.280052	-4.69345	2.69E-06	1.99E-05	TRUE	Ccdc91
Far2	38.92295	-1.756667472	0.479824	-3.66106	0.000251	0.001146	TRUE	Far2

Slc6a1	552.1758	-2.34923044	0.433354	-5.42104	5.93E-08	6.36E-07	TRUE		Slc6a1
Necap1	355.2026	-1.807926557	0.352183	-5.13348	2.84E-07	2.63E-06	TRUE		Necap1
Zfp606	575.9329	-1.701298114	0.483624	-3.51781	0.000435	0.00185	TRUE		Zfp606
Zik1	592.598	-1.449056725	0.356509	-4.06457	4.81E-05	0.000265	TRUE		Zik1
Ppm1n	10.89349	-5.581788455	1.369678	-4.07526	4.60E-05	0.000255	TRUE		Ppm1n
Vasp	421.4077	-1.058342266	0.206186	-5.13294	2.85E-07	2.64E-06	TRUE		Vasp
Nova2	3414.346	-1.434326487	0.20729	-6.91942	4.53E-12	9.38E-11	TRUE		Nova2
Zfp939	87.24703	-1.097979818	0.374263	-2.93371	0.003349	0.011021	TRUE		Zfp939
Psd3	428.3786	-1.166551223	0.277189	-4.2085	2.57E-05	0.000152	TRUE		Psd3
Slc17a6	1642.655	-1.787412608	0.208393	-8.5771	9.73E-18	3.92E-16	TRUE		Slc17a6
Fam189a1	462.5494	-5.168502773	0.532773	-9.70113	2.98E-22	1.91E-20	TRUE		Fam189a1
Nr2f2	1134.045	-5.599254272	0.400555	-13.9788	2.10E-44	8.94E-42	TRUE		Nr2f2
Lrrc28	181.8572	-1.519948303	0.36711	-4.14031	3.47E-05	0.000198	TRUE		Lrrc28
Tyrobp	4.609196	-4.344423665	1.581345	-2.7473	0.006009	0.018159	TRUE		Tyrobp
Dpf1	1210.667	-1.12401787	0.327664	-3.43039	0.000603	0.002469	TRUE		Dpf1
Lrfn1	708.1281	-1.461958007	0.23007	-6.3544	2.09E-10	3.37E-09	TRUE		Lrfn1
Ccdc90b	168.8603	-1.432722805	0.398627	-3.59415	0.000325	0.001436	TRUE		Ccdc90b
Sytl2	5.734444	-4.65574326	1.505308	-3.09288	0.001982	0.007016	TRUE		Sytl2
Me3	8.559989	-2.953242624	1.125557	-2.62381	0.008695	0.024786	TRUE		Me3
Sh3gl3	152.1097	-2.861835715	0.385934	-7.41535	1.21E-13	3.06E-12	TRUE		Sh3gl3
Rab30	242.285	-1.709203802	0.300703	-5.68404	1.32E-08	1.60E-07	TRUE		Rab30
Qprt	10.24485	-3.827846916	1.163478	-3.29	0.001002	0.003856	TRUE		Qprt
Sez6l2	560.7532	-2.436966464	0.428976	-5.68089	1.34E-08	1.63E-07	TRUE		Sez6l2
Kctd13	399.6001	-1.40118011	0.242393	-5.78061	7.44E-09	9.45E-08	TRUE		Kctd13
Fchsd2	633.6546	-1.085411276	0.326475	-3.32464	0.000885	0.003464	TRUE		Fchsd2
Rab6a	1186.52	-2.164538644	0.339319	-6.37906	1.78E-10	2.90E-09	TRUE		Rab6a
Coro1a	512.5725	-1.767402259	0.349391	-5.05852	4.23E-07	3.78E-06	TRUE		Coro1a
Pgm2l1	1770.6	-2.535264031	0.195247	-12.9849	1.49E-38	4.14E-36	TRUE		Pgm2l1
Slco2b1	32.29908	-2.305759109	0.619683	-3.72087	0.000199	0.00093	TRUE		Slco2b1
Dgat2	98.78325	-2.918830149	0.524021	-5.57006	2.55E-08	2.94E-07	TRUE		Dgat2
Acer3	51.85489	-3.82021504	0.625518	-6.10729	1.01E-09	1.48E-08	TRUE		Acer3
Dkk3	67.252	-2.15839636	0.551792	-3.91161	9.17E-05	0.000469	TRUE		Dkk3
Pak1	1934.799	-2.096549678	0.249999	-8.38623	5.02E-17	1.90E-15	TRUE		Pak1
Tgfb1i1	159.3227	-1.480953468	0.478587	-3.09443	0.001972	0.006987	TRUE		Tgfb1i1
Stx1b	834.2683	-1.922589049	0.236282	-8.13684	4.06E-16	1.38E-14	TRUE		Stx1b
Rgs10	33.63186	-2.412335706	0.546715	-4.41242	1.02E-05	6.62E-05	TRUE		Rgs10
Ptpn5	1311.338	-3.118382409	0.403297	-7.73222	1.06E-14	3.07E-13	TRUE		Ptpn5
Acadsb	479.8513	-1.046386896	0.215021	-4.86644	1.14E-06	9.21E-06	TRUE		Acadsb
Ubfd1	1084.96	-1.784265772	0.36876	-4.83855	1.31E-06	1.04E-05	TRUE		Ubfd1
Cdr2	333.7335	-3.158664049	0.294299	-10.7328	7.14E-27	7.29E-25	TRUE		Cdr2
Thumpd1	607.727	-1.214741317	0.38442	-3.15994	0.001578	0.005746	TRUE		Thumpd1
Atp6ap2	383.1248	-1.613849525	0.481032	-3.35497	0.000794	0.003149	TRUE		Atp6ap2
Usp9x	947.6499	-1.629228559	0.302849	-5.37968	7.46E-08	7.85E-07	TRUE		Usp9x
Cask	1196.726	-1.554674824	0.191212	-8.13064	4.27E-16	1.45E-14	TRUE		Cask
Tmem9b	267.9662	-1.409997906	0.281883	-5.00208	5.67E-07	4.94E-06	TRUE		Tmem9b
Trim66	14.36302	-5.984105071	1.377154	-4.34527	1.39E-05	8.77E-05	TRUE		Trim66
Stk33	25.74976	-3.050389983	0.716568	-4.25695	2.07E-05	0.000125	TRUE		Stk33
Tub	1438.527	-3.204616575	0.318492	-10.0618	8.15E-24	6.20E-22	TRUE		Tub
Fgf15	177.9524	-5.417956903	2.014923	-2.68891	0.007168	0.020994	TRUE		Fgf15
Fgf3	4.494265	-4.309960176	1.620593	-2.65949	0.007826	0.022648	TRUE		Fgf3
Dock11	557.0371	-1.623216629	0.327967	-4.94933	7.45E-07	6.30E-06	TRUE		Dock11
Smarca1	301.9175	-3.22423235	0.27274	-11.8216	3.02E-32	5.52E-30	TRUE		Smarca1
Rab33a	200.6119	-1.278769977	0.306347	-4.17426	2.99E-05	0.000173	TRUE		Rab33a
Igsf1	4.805116	-4.406890812	1.648366	-2.67349	0.007507	0.021825	TRUE		Igsf1
Arhgef6	26.76686	-1.899742336	0.591975	-3.20916	0.001331	0.004951	TRUE		Arhgef6
Rbmx	2325.987	-1.022820492	0.350647	-2.91695	0.003535	0.011564	TRUE		Rbmx
Fgf13	363.3754	-1.525298211	0.334499	-4.55994	5.12E-06	3.55E-05	TRUE		Fgf13
Wdr13	1256.621	-1.074084959	0.232817	-4.61343	3.96E-06	2.82E-05	TRUE		Wdr13
Ebp	85.59954	-1.612928807	0.546551	-2.9511	0.003166	0.010498	TRUE		Ebp
Slc38a5	39.06273	-2.733968292	0.543896	-5.02664	4.99E-07	4.41E-06	TRUE		Slc38a5
Vbp1	753.5116	-1.404664495	0.327368	-4.29078	1.78E-05	0.000109	TRUE		Vbp1
Fundc2	220.1456	-1.475018396	0.437259	-3.37333	0.000743	0.002964	TRUE		Fundc2
Rab39b	181.3432	-2.256101968	0.466372	-4.83756	1.31E-06	1.05E-05	TRUE		Rab39b
Heph	36.15198	-2.096519704	0.860664	-2.43593	0.014853	0.0388	TRUE		Heph
Gpr165	16.75358	-4.481180349	1.094021	-4.09606	4.20E-05	0.000235	TRUE		Gpr165
Magee2	46.00472	-3.521373373	0.672901	-5.23312	1.67E-07	1.62E-06	TRUE		Magee2
Magee1	615.216	-3.396926588	0.322483	-10.5336	6.04E-26	5.50E-24	TRUE		Magee1
610002M06F	160.8766	-1.864765047	0.496981	-3.75218	0.000175	0.000834	TRUE		2610002M06Rik
Sh3bgrl	1921.939	-1.192401928	0.35124	-3.39483	0.000687	0.002771	TRUE		Sh3bgrl
Cstf2	557.935	-1.169027086	0.272797	-4.28534	1.82E-05	0.000112	TRUE		Cstf2
Acsl4	195.0069	-1.640220492	0.361555	-4.53657	5.72E-06	3.92E-05	TRUE		Acsl4
Dcx	4874.771	-2.246362305	0.327174	-6.86595	6.60E-12	1.34E-10	TRUE		Dcx

Lrch2	592.9561	-1.245250316	0.260775	-4.77519	1.80E-06	1.38E-05	TRUE	Lrch2
Cdkl5	288.8164	-1.223795109	0.287828	-4.25183	2.12E-05	0.000128	TRUE	Cdkl5
Pdha1	447.5632	-1.347821951	0.318555	-4.23105	2.33E-05	0.000138	TRUE	Pdha1
Nlgn3	530.0385	-1.649481388	0.23883	-6.90651	4.97E-12	1.02E-10	TRUE	Nlgn3
Gpm6b	3319.854	-1.168797652	0.314459	-3.71686	0.000202	0.000943	TRUE	Gpm6b
Gabra3	261.7687	-5.066033766	0.444933	-11.3861	4.91E-30	7.41E-28	TRUE	Gabra3
Gabrq	25.82957	-2.430978534	0.698313	-3.48121	0.000499	0.002087	TRUE	Gabrq
Cetn2	227.092	-1.309208087	0.48812	-2.68215	0.007315	0.021345	TRUE	Cetn2
Zfp92	8.259126	-3.516946226	1.242784	-2.82989	0.004656	0.014601	TRUE	Zfp92
Bgn	133.0976	-1.76847722	0.297225	-5.94997	2.68E-09	3.64E-08	TRUE	Bgn
Atp2b3	43.98767	-3.661099165	0.701257	-5.22077	1.78E-07	1.72E-06	TRUE	Atp2b3
Hcfc1	2065.396	-1.009446753	0.259567	-3.88896	0.000101	0.00051	TRUE	Hcfc1
L1cam	3114.835	-1.035742148	0.334672	-3.0948	0.001969	0.00698	TRUE	L1cam
Tceal6	80.47437	-3.09505212	0.498522	-6.20845	5.35E-10	8.19E-09	TRUE	Tceal6
Psmd10	155.1752	-1.062331085	0.333674	-3.18374	0.001454	0.00535	TRUE	Psmd10
Tsc22d3	386.6248	-2.18346069	0.505956	-4.31552	1.59E-05	9.89E-05	TRUE	Tsc22d3
Rbm41	72.76473	-1.720809934	0.463071	-3.71608	0.000202	0.000945	TRUE	Rbm41
Morc4	33.98641	-1.489609957	0.629825	-2.36512	0.018024	0.045542	TRUE	Morc4
Mcf2l	2115.112	-2.393570321	0.201714	-11.8662	1.77E-32	3.30E-30	TRUE	Mcf2l
Proz	86.57017	-1.320687769	0.462784	-2.85379	0.00432	0.013693	TRUE	Proz
Lamp1	893.9451	-1.238020913	0.4358	-2.8408	0.0045	0.014164	TRUE	Lamp1
Agpat5	454.9618	-1.140192504	0.24692	-4.61765	3.88E-06	2.77E-05	TRUE	Agpat5
Erlin2	611.3278	-1.614995888	0.35599	-4.53664	5.72E-06	3.92E-05	TRUE	Erlin2
Adgra2	68.31144	-1.404534366	0.424228	-3.3108	0.00093	0.003615	TRUE	Adgra2
Col4a1	602.2271	-1.144698618	0.340072	-3.36605	0.000763	0.003036	TRUE	Col4a1
Col4a2	473.256	-1.290623406	0.303084	-4.2583	2.06E-05	0.000124	TRUE	Col4a2
Leprotl1	687.2855	-1.887047294	0.230546	-8.18513	2.72E-16	9.48E-15	TRUE	Leprotl1
Gpm6a	3555.175	-3.52648447	0.34905	-10.1031	5.35E-24	4.18E-22	TRUE	Gpm6a
Asb5	4.039531	-4.154299743	1.629857	-2.54887	0.010807	0.029771	TRUE	Asb5
Tnks	1240.618	-2.245006195	0.400053	-5.61177	2.00E-08	2.36E-07	TRUE	Tnks
Dusp4	777.0016	-2.608681206	0.311598	-8.37195	5.67E-17	2.12E-15	TRUE	Dusp4
Saraf	795.0439	-1.401395928	0.270641	-5.17806	2.24E-07	2.11E-06	TRUE	Saraf
Plat	115.7638	-1.939808048	0.351954	-5.51153	3.56E-08	3.98E-07	TRUE	Plat
Adam9	209.0102	-1.54750048	0.322759	-4.7946	1.63E-06	1.27E-05	TRUE	Adam9
Slit2	812.6776	-2.411905122	0.229716	-10.4995	8.68E-26	7.87E-24	TRUE	Slit2
Tenm3	860.2266	-2.181713209	0.40348	-5.40724	6.40E-08	6.81E-07	TRUE	Tenm3
Klhl2	328.5171	-1.053324307	0.344771	-3.05514	0.00225	0.007853	TRUE	Klhl2
Casp3	1651.357	-1.608114531	0.229881	-6.99544	2.64E-12	5.66E-11	TRUE	Casp3
Slc25a4	2850.343	-1.558751282	0.339358	-4.59323	4.36E-06	3.07E-05	TRUE	Slc25a4
Pdlim3	136.8846	-4.865592398	0.520913	-9.34051	9.59E-21	5.43E-19	TRUE	Pdlim3
Sh3rf1	781.622	-1.753585516	0.284102	-6.17238	6.73E-10	1.02E-08	TRUE	Sh3rf1
Cbln1	335.5344	-3.419782104	0.626018	-5.46275	4.69E-08	5.14E-07	TRUE	Cbln1
Nkd1	1265.604	-2.955068318	0.335229	-8.81508	1.20E-18	5.30E-17	TRUE	Nkd1
Aktip	456.3383	-1.100959908	0.378194	-2.9111	0.003602	0.011744	TRUE	Aktip
Cdh11	200.6297	-2.594680504	0.498104	-5.20911	1.90E-07	1.82E-06	TRUE	Cdh11
Smad1	690.2805	-1.600234696	0.310875	-5.14751	2.64E-07	2.46E-06	TRUE	Smad1
Vps35	755.5874	-1.727709551	0.466815	-3.70106	0.000215	0.000997	TRUE	Vps35
Dnaja2	1261.917	-1.020656861	0.286544	-3.56195	0.000368	0.001596	TRUE	Dnaja2
Itfg1	689.2663	-1.457526906	0.258584	-5.63656	1.73E-08	2.07E-07	TRUE	Itfg1
Tbc1d9	872.0368	-2.115863704	0.231726	-9.13087	6.79E-20	3.58E-18	TRUE	Tbc1d9
Irx3	43.40585	-4.427154082	0.806618	-5.48854	4.05E-08	4.49E-07	TRUE	Irx3
Crnde	44.60492	-2.081559182	0.620383	-3.35528	0.000793	0.003146	TRUE	Crnde
St3gal2	545.6545	-1.518320059	0.280298	-5.41681	6.07E-08	6.50E-07	TRUE	St3gal2
Cx3cl1	77.46517	-2.828483222	0.412557	-6.85598	7.08E-12	1.42E-10	TRUE	Cx3cl1
Kifc3	94.8741	-2.642502086	0.525666	-5.02696	4.98E-07	4.40E-06	TRUE	Kifc3
Map1lc3b	1969.34	-1.048579983	0.234565	-4.47032	7.81E-06	5.20E-05	TRUE	Map1lc3b
30548M08R	479.8024	-1.709439996	0.205663	-8.31186	9.42E-17	3.44E-15	TRUE	6430548M08Rik
Cotl1	2242.689	-1.4996075	0.283389	-5.2917	1.21E-07	1.22E-06	TRUE	Cotl1
Hsbp1	1065.834	-1.243059424	0.321677	-3.86431	0.000111	0.000558	TRUE	Hsbp1
Rab3a	748.5316	-1.93843582	0.273299	-7.09273	1.31E-12	2.94E-11	TRUE	Rab3a
Pgr	6.18737	-4.771851244	1.567286	-3.04466	0.002329	0.00809	TRUE	Pgr
Cdh5	99.97811	-1.88634201	0.558091	-3.37999	0.000725	0.002907	TRUE	Cdh5
Bean1	22.54961	-1.538776705	0.589266	-2.61134	0.009019	0.025551	TRUE	Bean1
Hsd11b2	45.6234	-5.80045068	1.061588	-5.46394	4.66E-08	5.11E-07	TRUE	Hsd11b2
Has3	167.2631	-2.113610771	0.544938	-3.87862	0.000105	0.00053	TRUE	Has3
Cyb5b	1853.079	-1.101211177	0.29021	-3.79454	0.000148	0.000717	TRUE	Cyb5b
Panx1	1257.959	-1.942241445	0.257266	-7.54955	4.37E-14	1.17E-12	TRUE	Panx1
931406C07R	209.0966	-1.538715773	0.319433	-4.81702	1.46E-06	1.15E-05	TRUE	4931406C07Rik
Gabarapl2	686.2581	-1.396054609	0.309966	-4.50389	6.67E-06	4.51E-05	TRUE	Gabarapl2
Chst5	11.45112	-2.554728385	1.056496	-2.41812	0.015601	0.040449	TRUE	Chst5
Cdh15	5.405289	-4.57674532	1.587529	-2.88294	0.00394	0.012671	TRUE	Cdh15
Dbndd1	516.7847	-1.270068852	0.330097	-3.84757	0.000119	0.000592	TRUE	Dbndd1

Vps26b	952.6752	-2.263577319	0.40661	-5.56695	2.59E-08	2.99E-07	TRUE	Vps26b
Nectin1	649.3683	-1.249060775	0.223729	-5.58291	2.37E-08	2.76E-07	TRUE	Nectin1
Sc5d	501.2866	-1.519554225	0.369043	-4.11755	3.83E-05	0.000216	TRUE	Sc5d
Ubash3b	218.9453	-2.682608144	0.389582	-6.88587	5.74E-12	1.17E-10	TRUE	Ubash3b
Clmp	1425.925	-1.181363219	0.365753	-3.22995	0.001238	0.004652	TRUE	Clmp
Barx2	5.02242	-4.469077178	1.599843	-2.79345	0.005215	0.01612	TRUE	Barx2
Kcnj5	84.29576	-8.5362612	1.262474	-6.76153	1.37E-11	2.64E-10	TRUE	Kcnj5
Ets1	150.5383	-1.275124595	0.376734	-3.38468	0.000713	0.002861	TRUE	Ets1
Kirrel3	244.1548	-1.494196742	0.373712	-3.99826	6.38E-05	0.00034	TRUE	Kirrel3
Alg9	267.4851	-1.116598723	0.4418	-2.52738	0.011492	0.031342	TRUE	Alg9
Dixdc1	705.8384	-1.582576482	0.340969	-4.64141	3.46E-06	2.50E-05	TRUE	Dixdc1
Arcn1	1307.737	-1.102755983	0.295989	-3.72567	0.000195	0.000915	TRUE	Arcn1
Ddx6	3302.441	-1.454418319	0.300928	-4.83312	1.34E-06	1.07E-05	TRUE	Ddx6
Ddx25	307.8258	-1.441427629	0.278184	-5.18156	2.20E-07	2.08E-06	TRUE	Ddx25
Hyou1	1008.233	-1.101179106	0.244168	-4.50992	6.49E-06	4.39E-05	TRUE	Hyou1
Fez1	2004.026	-1.133202864	0.291558	-3.88672	0.000102	0.000515	TRUE	Fez1
Olfm2	317.9591	-1.906756012	0.269206	-7.08289	1.41E-12	3.13E-11	TRUE	Olfm2
Pde4a	328.1953	-1.750270365	0.416275	-4.2046	2.62E-05	0.000154	TRUE	Pde4a
Scg3	897.5414	-2.866409338	0.200779	-14.2764	3.07E-46	1.42E-43	TRUE	Scg3
Yipf2	54.83216	-1.518620453	0.402885	-3.76937	0.000164	0.000785	TRUE	Yipf2
Lysmd2	47.47375	-1.31486336	0.483422	-2.71991	0.00653	0.019438	TRUE	Lysmd2
Tmod2	1604.259	-3.374170798	0.353908	-9.53402	1.51E-21	9.14E-20	TRUE	Tmod2
Polr2m	2285.148	-1.125412521	0.314909	-3.57377	0.000352	0.001534	TRUE	Polr2m
Nedd4	10962.85	-1.264565746	0.28603	-4.4211	9.82E-06	6.38E-05	TRUE	Nedd4
Cgnl1	59.29456	-2.111512487	0.538277	-3.92272	8.76E-05	0.00045	TRUE	Cgnl1
Rora	520.6524	-3.0928427	0.676047	-4.57489	4.76E-06	3.33E-05	TRUE	Rora
Fem1b	2472.662	-1.882034026	0.265838	-7.07962	1.45E-12	3.20E-11	TRUE	Fem1b
Drd2	18.30696	-3.158041787	0.815409	-3.87295	0.000108	0.00054	TRUE	Drd2
Elovl4	254.6007	-2.213260262	0.37759	-5.86154	4.59E-09	6.01E-08	TRUE	Elovl4
Ptpn9	445.9175	-1.078123275	0.367604	-2.93284	0.003359	0.011046	TRUE	Ptpn9
Celf6	217.865	-2.695255717	0.473599	-5.69101	1.26E-08	1.54E-07	TRUE	Celf6
Chrna3	100.5105	-2.705491931	0.418755	-6.4608	1.04E-10	1.75E-09	TRUE	Chrna3
Tmem266	56.69959	-1.561924897	0.615302	-2.53847	0.011134	0.030513	TRUE	Tmem266
Rcn2	1315.157	-1.453415133	0.479966	-3.02816	0.00246	0.008475	TRUE	Rcn2
Col12a1	96.89118	-1.080058025	0.409143	-2.63981	0.008295	0.023795	TRUE	Col12a1
Nptn	339.775	-1.139915062	0.290295	-3.92675	8.61E-05	0.000444	TRUE	Nptn
Hcn4	333.7574	-4.372613369	0.481555	-9.08019	1.08E-19	5.55E-18	TRUE	Hcn4
Neo1	1843.946	-1.840891968	0.1627	-11.3147	1.11E-29	1.57E-27	TRUE	Neo1
Ooep	28.72479	-2.865891335	0.713134	-4.01873	5.85E-05	0.000315	TRUE	Ooep
Ctsh	13.41616	-2.441183146	0.996063	-2.45083	0.014253	0.037489	TRUE	Ctsh
Zic1	8492.844	-5.120916847	1.129523	-4.5337	5.80E-06	3.97E-05	TRUE	Zic1
Lactb	104.4751	-1.211806547	0.314585	-3.85208	0.000117	0.000582	TRUE	Lactb
Aph1b	311.7265	-1.831048357	0.259288	-7.06182	1.64E-12	3.62E-11	TRUE	Aph1b
Dapk2	7.603903	-5.062630391	1.458189	-3.47186	0.000517	0.002152	TRUE	Dapk2
Csnk1g1	604.6854	-1.067939678	0.209504	-5.09747	3.44E-07	3.12E-06	TRUE	Csnk1g1
Spg21	463.0497	-1.161754302	0.308786	-3.76233	0.000168	0.000804	TRUE	Spg21
Atp1b3	728.1816	-1.186768314	0.320103	-3.70745	0.000209	0.000973	TRUE	Atp1b3
Me1	84.58915	-2.537033366	0.38636	-6.5665	5.15E-11	9.12E-10	TRUE	Me1
Dync1li1	1267.584	-1.359385185	0.20027	-6.78775	1.14E-11	2.23E-10	TRUE	Dync1li1
Tgfbr2	37.96882	-1.761000182	0.578117	-3.0461	0.002318	0.008056	TRUE	Tgfbr2
Clstn2	168.5381	-3.644778967	0.4541	-8.02638	1.00E-15	3.29E-14	TRUE	Clstn2
Copb2	765.0499	-1.245590623	0.348538	-3.57376	0.000352	0.001534	TRUE	Copb2
Faim	347.9365	-1.008285087	0.214491	-4.70083	2.59E-06	1.93E-05	TRUE	Faim
Mras	602.9071	-1.296944809	0.350682	-3.69835	0.000217	0.001007	TRUE	Mras
Nck1	182.9264	-1.094674869	0.413237	-2.64902	0.008073	0.023246	TRUE	Nck1
Map4	2537.29	-1.709994753	0.173531	-9.8541	6.58E-23	4.54E-21	TRUE	Map4
Pth1r	180.9776	-1.101046464	0.401532	-2.74212	0.006104	0.018391	TRUE	Pth1r
Trib1	192.7295	-2.30475451	0.435087	-5.29723	1.18E-07	1.19E-06	TRUE	Trib1
Fbxl2	138.2573	-1.294674908	0.370177	-3.49745	0.00047	0.001979	TRUE	Fbxl2
Wdr48	493.9312	-1.212410762	0.274876	-4.41076	1.03E-05	6.67E-05	TRUE	Wdr48
Amotl2	1422.368	-1.098728791	0.197671	-5.55837	2.72E-08	3.12E-07	TRUE	Amotl2
Ephb1	195.5215	-1.821195207	0.342933	-5.31065	1.09E-07	1.11E-06	TRUE	Ephb1
Rab6b	1846.249	-1.800886275	0.17671	-10.1912	2.17E-24	1.77E-22	TRUE	Rab6b
Trf	65.80475	-1.492093293	0.362338	-4.11796	3.82E-05	0.000216	TRUE	Trf
Atp2c1	756.0041	-1.618559643	0.296478	-5.45929	4.78E-08	5.24E-07	TRUE	Atp2c1
Col6a4	8.025945	-5.143260887	1.425057	-3.60916	0.000307	0.001367	TRUE	Col6a4
Mst1r	15.39737	-2.037155405	0.77238	-2.63751	0.008352	0.023931	TRUE	Mst1r
Bsn	1121.105	-2.664599276	0.335961	-7.93128	2.17E-15	6.87E-14	TRUE	Bsn
Klhdc8b	748.5424	-1.62668975	0.191344	-8.5014	1.87E-17	7.32E-16	TRUE	Klhdc8b
Thsd7a	1281.924	-7.241890672	1.351331	-5.35908	8.36E-08	8.69E-07	TRUE	Thsd7a
Oas3	3.850111	-4.083354482	1.674395	-2.4387	0.01474	0.038547	TRUE	Oas3
700025G04F	2126.404	-2.726382289	0.206015	-13.2339	5.59E-40	1.67E-37	TRUE	1700025G04Rik

Cd59a	23.22545	-3.114534959	0.760166	-4.09718	4.18E-05	0.000234	TRUE	Cd59a
Lmo2	119.7261	-1.704757962	0.456968	-3.73058	0.000191	0.0009	TRUE	Lmo2
Kank1	31.90819	-1.584271562	0.568509	-2.78671	0.005325	0.016388	TRUE	Kank1
Abtb2	166.9584	-2.329175901	0.447964	-5.19947	2.00E-07	1.91E-06	TRUE	Abtb2
Ablim3	108.738	-2.475959082	0.641258	-3.8611	0.000113	0.000565	TRUE	Ablim3
Heyl	55.41447	-1.407622513	0.580181	-2.42618	0.015259	0.039689	TRUE	Heyl
Ank2	5558.984	-1.442596572	0.33027	-4.36793	1.25E-05	7.97E-05	TRUE	Ank2
410131K14R	488.0887	-1.459215675	0.318968	-4.5748	4.77E-06	3.33E-05	TRUE	2410131K14Rik
Zswim6	498.6122	-1.766859676	0.223862	-7.89263	2.96E-15	9.20E-14	TRUE	Zswim6
Smap2	506.3549	-1.17508056	0.299254	-3.9267	8.61E-05	0.000444	TRUE	Smap2
Ccdc85a	35.98452	-5.685475658	1.139274	-4.99044	6.02E-07	5.21E-06	TRUE	Ccdc85a
Atg12	664.1137	-1.157892088	0.372862	-3.10542	0.0019	0.006756	TRUE	Atg12
Lrig2	687.5055	-1.004442454	0.223287	-4.49843	6.85E-06	4.61E-05	TRUE	Lrig2
Odf4	7.03291	-3.916955302	1.439196	-2.72163	0.006496	0.019364	TRUE	Odf4
Itgbl1	9.88395	-3.779593369	1.180708	-3.20112	0.001369	0.005076	TRUE	Itgbl1
Hspa13	409.1322	-2.080855136	0.355008	-5.86142	4.59E-09	6.01E-08	TRUE	Hspa13
Ift57	135.7699	-1.215274473	0.353244	-3.44032	0.000581	0.002386	TRUE	Ift57
Asic4	118.5411	-9.025258385	1.244027	-7.25487	4.02E-13	9.48E-12	TRUE	Asic4
Cdo1	856.8118	-3.044118528	0.314055	-9.69296	3.23E-22	2.06E-20	TRUE	Cdo1
Micall1	309.8067	-1.604047667	0.309247	-5.18695	2.14E-07	2.03E-06	TRUE	Micall1
Resp18	53.30075	-4.399252748	0.788272	-5.58088	2.39E-08	2.78E-07	TRUE	Resp18
Gas7	173.2889	-2.090637021	0.521152	-4.01157	6.03E-05	0.000324	TRUE	Gas7
Slc22a15	383.6836	-1.052620722	0.262641	-4.00783	6.13E-05	0.000329	TRUE	Slc22a15
Atp1a1	1049.001	-1.213477516	0.234481	-5.17515	2.28E-07	2.15E-06	TRUE	Atp1a1
Tmed7	723.1934	-1.534709228	0.531895	-2.88536	0.00391	0.012597	TRUE	Tmed7
Dpyd	36.3496	-1.743588776	0.645306	-2.70196	0.006893	0.020331	TRUE	Dpyd
Galnt9	119.5549	-3.673381689	0.451473	-8.13644	4.07E-16	1.38E-14	TRUE	Galnt9
Fem1c	542.8679	-1.727744158	0.370512	-4.66312	3.11E-06	2.28E-05	TRUE	Fem1c
Tnxb	3.723572	-4.035648334	1.658247	-2.43368	0.014946	0.038993	TRUE	Tnxb
Chst2	879.6246	-3.188194649	0.328505	-9.70516	2.87E-22	1.85E-20	TRUE	Chst2
Map2k4	595.9915	-1.024735038	0.275391	-3.72102	0.000198	0.00093	TRUE	Map2k4
Prrg3	265.3552	-1.618643981	0.330809	-4.89299	9.93E-07	8.15E-06	TRUE	Prrg3
Nudt15	46.81689	-1.372862964	0.431198	-3.18384	0.001453	0.005349	TRUE	Nudt15
Cacul1	604.243	-1.104114862	0.281883	-3.91693	8.97E-05	0.000461	TRUE	Cacul1
Snap91	665.2525	-2.317446396	0.279972	-8.27743	1.26E-16	4.52E-15	TRUE	Snap91
Terf2ip	423.31	-1.512362465	0.475752	-3.17889	0.001478	0.005435	TRUE	Terf2ip
Armcx2	1636.691	-1.568207483	0.179755	-8.72413	2.68E-18	1.15E-16	TRUE	Armcx2
Armcx1	594.7391	-1.394680444	0.222523	-6.26757	3.67E-10	5.75E-09	TRUE	Armcx1
Ttc7b	189.1502	-1.024040519	0.315989	-3.24075	0.001192	0.004497	TRUE	Ttc7b
Fam20b	535.7828	-2.17739265	0.43516	-5.00366	5.63E-07	4.90E-06	TRUE	Fam20b
Rbfox2	4820.376	-2.081995492	0.179904	-11.5728	5.66E-31	9.36E-29	TRUE	Rbfox2
Tmem35a	785.08	-3.227221498	0.366251	-8.8115	1.23E-18	5.45E-17	TRUE	Tmem35a
Ndn	3222.58	-2.495952293	0.306095	-8.15418	3.52E-16	1.21E-14	TRUE	Ndn
Cplx1	130.1937	-1.561800154	0.532281	-2.93416	0.003344	0.011009	TRUE	Cplx1
Map3k13	583.6615	-2.95768614	0.291982	-10.1297	4.08E-24	3.26E-22	TRUE	Map3k13
Pcgf3	1291.536	-1.366809726	0.373071	-3.66367	0.000249	0.001136	TRUE	Pcgf3
Vps8	408.1121	-1.526741655	0.244177	-6.25261	4.04E-10	6.30E-09	TRUE	Vps8
Gabrb3	472.3842	-3.739621871	0.346924	-10.7794	4.31E-27	4.51E-25	TRUE	Gabrb3
Adra2a	15.22861	-2.34782226	0.862058	-2.72351	0.006459	0.01928	TRUE	Adra2a
Vav3	447.6393	-5.501089645	0.568764	-9.672	3.97E-22	2.50E-20	TRUE	Vav3
Egr3	6.962982	-4.936589247	1.452638	-3.39836	0.000678	0.00274	TRUE	Egr3
St18	410.5244	-2.079078559	0.26297	-7.90613	2.66E-15	8.32E-14	TRUE	St18
Mtss2	1210.921	-1.107231745	0.224434	-4.93343	8.08E-07	6.78E-06	TRUE	Mtss2
Nrxn2	1728.057	-2.36244671	0.305923	-7.72235	1.14E-14	3.30E-13	TRUE	Nrxn2
Atp6v1h	641.6247	-1.369469327	0.319463	-4.28678	1.81E-05	0.000111	TRUE	Atp6v1h
Map9	470.593	-1.86046921	0.323067	-5.75878	8.47E-09	1.06E-07	TRUE	Map9
Zdhhc15	126.3049	-1.257705583	0.465681	-2.70079	0.006918	0.020391	TRUE	Zdhhc15
Vhl	548.621	-1.125742307	0.276927	-4.06512	4.80E-05	0.000264	TRUE	Vhl
Zswim5	573.9004	-1.78798515	0.221762	-8.06263	7.47E-16	2.49E-14	TRUE	Zswim5
Trim36	621.4292	-1.687074063	0.217296	-7.76393	8.23E-15	2.44E-13	TRUE	Trim36
Ppp3r1	872.7139	-1.966443211	0.339269	-5.79611	6.79E-09	8.69E-08	TRUE	Ppp3r1
Zbtb41	1296.666	-3.38133361	0.427311	-7.91305	2.51E-15	7.89E-14	TRUE	Zbtb41
Pqlc1	244.0013	-2.499403105	0.272602	-9.16868	4.79E-20	2.54E-18	TRUE	Pqlc1
Zfp551	20.17674	-2.933624339	0.805274	-3.64301	0.000269	0.001219	TRUE	Zfp551
Kctd7	122.2882	-1.314713083	0.318707	-4.12515	3.71E-05	0.00021	TRUE	Kctd7
Exoc3	855.0845	-1.005529048	0.17896	-5.61875	1.92E-08	2.27E-07	TRUE	Exoc3
Tspoap1	1652.744	-1.23049228	0.274605	-4.48096	7.43E-06	4.97E-05	TRUE	Tspoap1
Irf2bpl	1149.159	-2.065288611	0.383908	-5.37964	7.46E-08	7.85E-07	TRUE	Irf2bpl
Nsf	562.0606	-1.673322324	0.255898	-6.53903	6.19E-11	1.08E-09	TRUE	Nsf
Hsdl1	1432.657	-1.537890928	0.195901	-7.85034	4.15E-15	1.28E-13	TRUE	Hsdl1
Zdhhc14	69.97468	-1.469185775	0.419285	-3.50402	0.000458	0.001936	TRUE	Zdhhc14
Med13	729.9884	-1.199656099	0.451041	-2.65975	0.00782	0.022634	TRUE	Med13

Tmem132d	28.85417	-6.990683853	1.422173	-4.91549	8.86E-07	7.36E-06	TRUE	Tmem132d
Tmem132c	199.7231	-3.364441879	0.621429	-5.41404	6.16E-08	6.60E-07	TRUE	Tmem132c
Zbed4	421.2658	-1.059111156	0.349016	-3.03456	0.002409	0.008326	TRUE	Zbed4
Ina	5081.37	-3.580043067	0.606784	-5.90003	3.63E-09	4.84E-08	TRUE	Ina
Tulp4	2585.626	-1.066713026	0.192341	-5.54595	2.92E-08	3.33E-07	TRUE	Tulp4
Barhl2	452.4287	-4.91539654	1.615807	-3.04207	0.00235	0.008154	TRUE	Barhl2
Cmip	1207.927	-1.646795133	0.375054	-4.39082	1.13E-05	7.24E-05	TRUE	Cmip
Neurl1b	99.40259	-1.436519219	0.494314	-2.90608	0.00366	0.011902	TRUE	Neurl1b
Eda2r	14.91361	-2.87816166	0.959964	-2.9982	0.002716	0.009237	TRUE	Eda2r
Rasd2	15.23848	-4.335885134	1.129717	-3.83803	0.000124	0.000614	TRUE	Rasd2
Edil3	597.0448	-4.275956413	0.437604	-9.7713	1.50E-22	9.83E-21	TRUE	Edil3
Zfp418	36.30844	-1.714184904	0.556624	-3.07961	0.002073	0.007304	TRUE	Zfp418
Daam1	778.3243	-1.187084741	0.247547	-4.79539	1.62E-06	1.26E-05	TRUE	Daam1
Hid1	615.892	-1.670268201	0.318107	-5.25066	1.52E-07	1.49E-06	TRUE	Hid1
Slc41a2	42.12078	-3.548021466	0.819266	-4.33073	1.49E-05	9.30E-05	TRUE	Slc41a2
Chst11	323.7242	-3.305512993	0.365666	-9.03971	1.57E-19	7.81E-18	TRUE	Chst11
Ppm1h	123.572	-3.409746528	0.609492	-5.59441	2.21E-08	2.59E-07	TRUE	Ppm1h
Zyg11b	1405.817	-2.322083207	0.216818	-10.7098	9.15E-27	9.21E-25	TRUE	Zyg11b
Tiparp	171.8769	-1.165614264	0.301556	-3.86533	0.000111	0.000556	TRUE	Tiparp
Lrrn1	365.0373	-1.747368536	0.537711	-3.24964	0.001155	0.004373	TRUE	Lrrn1
Tmx4	1702.588	-2.689407243	0.275834	-9.7501	1.84E-22	1.20E-20	TRUE	Tmx4
Mast4	400.5687	-1.328083452	0.349421	-3.80081	0.000144	0.000701	TRUE	Mast4
B3galt1	380.1404	-3.733447236	0.310037	-12.0419	2.14E-33	4.25E-31	TRUE	B3galt1
Unc13a	2243.937	-1.54354386	0.34789	-4.43687	9.13E-06	5.99E-05	TRUE	Unc13a
Grip1	493.6796	-1.168860751	0.30386	-3.84671	0.00012	0.000594	TRUE	Grip1
Celf5	1798.618	-2.559062377	0.307651	-8.31808	8.94E-17	3.29E-15	TRUE	Celf5
Nrip3	21.13889	-2.697972555	0.727301	-3.70957	0.000208	0.000966	TRUE	Nrip3
Larp6	52.84196	-5.304015429	0.795592	-6.66675	2.62E-11	4.86E-10	TRUE	Larp6
Ttc21b	226.4188	-1.113592151	0.273499	-4.07166	4.67E-05	0.000258	TRUE	Ttc21b
Acot11	11.72287	-3.184184698	1.169195	-2.7234	0.006461	0.019281	TRUE	Acot11
Mfsd12	160.4799	-1.115803079	0.352782	-3.16287	0.001562	0.005704	TRUE	Mfsd12
Sncb	26.70388	-2.965593324	0.712169	-4.16417	3.12E-05	0.00018	TRUE	Sncb
Mdga2	213.8083	-2.198788696	0.351881	-6.24867	4.14E-10	6.44E-09	TRUE	Mdga2
Cdhr2	12.51424	-2.283440267	0.853146	-2.67649	0.00744	0.02166	TRUE	Cdhr2
Synrg	974.4141	-1.058232545	0.204492	-5.17495	2.28E-07	2.15E-06	TRUE	Synrg
Atcay	2061.455	-1.676368783	0.21943	-7.63966	2.18E-14	6.06E-13	TRUE	Atcay
Vat1	1844.655	-1.137524064	0.259549	-4.38269	1.17E-05	7.48E-05	TRUE	Vat1
Eef2	33108.22	-1.099843073	0.233388	-4.71252	2.45E-06	1.83E-05	TRUE	Eef2
Zc4h2	437.5106	-2.444491792	0.541552	-4.51386	6.37E-06	4.32E-05	TRUE	Zc4h2
Mtmr9	568.267	-1.820322493	0.338509	-5.37747	7.55E-08	7.93E-07	TRUE	Mtmr9
Secisbp2l	657.0107	-1.308842373	0.273235	-4.79018	1.67E-06	1.29E-05	TRUE	Secisbp2l
Fam167a	241.4969	-1.569809969	0.537044	-2.92306	0.003466	0.011366	TRUE	Fam167a
Dcbld2	336.0555	-1.384757377	0.33674	-4.11224	3.92E-05	0.000221	TRUE	Dcbld2
Eif2s3x	848.8925	-2.224549998	0.489262	-4.54675	5.45E-06	3.75E-05	TRUE	Eif2s3x
Ap2b1	3267.815	-1.194406693	0.179092	-6.66924	2.57E-11	4.78E-10	TRUE	Ap2b1
Zc3h12c	523.8552	-1.472472248	0.290057	-5.07649	3.84E-07	3.46E-06	TRUE	Zc3h12c
L10059E24R	243.701	-1.291225708	0.529747	-2.43744	0.014792	0.038658	TRUE	1110059E24Rik
Ccdc186	549.6565	-1.02056606	0.249601	-4.08879	4.34E-05	0.000242	TRUE	Ccdc186
Ano4	21.85082	-3.327278752	0.942784	-3.52921	0.000417	0.00178	TRUE	Ano4
Arl6ip5	473.0986	-1.829484377	0.37331	-4.90072	9.55E-07	7.87E-06	TRUE	Arl6ip5
Leprot	403.062	-1.950455436	0.379872	-5.13451	2.83E-07	2.62E-06	TRUE	Leprot
Rims4	214.9374	-6.091446113	0.660715	-9.21947	2.99E-20	1.60E-18	TRUE	Rims4
Spcs2	899.296	-1.557553489	0.485136	-3.21055	0.001325	0.004932	TRUE	Spcs2
Pcyt1b	611.9225	-3.302801553	0.261831	-12.6143	1.76E-36	4.27E-34	TRUE	Pcyt1b
Ssc5d	49.40739	-2.327591059	0.476758	-4.88213	1.05E-06	8.58E-06	TRUE	Ssc5d
Vps13c	421.4345	-1.23680118	0.310956	-3.97742	6.97E-05	0.000368	TRUE	Vps13c
Nat14	271.8121	-1.575376681	0.262333	-6.00526	1.91E-09	2.64E-08	TRUE	Nat14
Ror1	5.778173	-4.672826802	1.564543	-2.9867	0.00282	0.009536	TRUE	Ror1
Gdpd5	1220.696	-1.400793826	0.413732	-3.38575	0.00071	0.002852	TRUE	Gdpd5
Kcnh4	352.3137	-3.285563849	0.603735	-5.44206	5.27E-08	5.72E-07	TRUE	Kcnh4
Pdzrn3	1541.414	-3.71878434	0.379274	-9.805	1.07E-22	7.20E-21	TRUE	Pdzrn3
Hacd2	89.85805	-1.45547338	0.411484	-3.53713	0.000405	0.001733	TRUE	Hacd2
Brsk1	1319.296	-1.554388447	0.200651	-7.74671	9.43E-15	2.76E-13	TRUE	Brsk1
Kank4	15.56151	-2.155441029	0.79561	-2.70917	0.006745	0.019948	TRUE	Kank4
Foxa1	45.72032	-5.97322145	1.031239	-5.79228	6.94E-09	8.85E-08	TRUE	Foxa1
Tab3	262.5874	-1.130833743	0.241166	-4.68903	2.75E-06	2.03E-05	TRUE	Tab3
Fbxl21	13.91611	-2.61572586	1.042805	-2.50836	0.012129	0.032811	TRUE	Fbxl21
Tdrd7	124.6482	-1.695756074	0.569862	-2.97573	0.002923	0.009837	TRUE	Tdrd7
Capn5	208.3181	-2.832116392	0.344934	-8.2106	2.20E-16	7.75E-15	TRUE	Capn5
Igfbpl1	8170.384	-1.692923442	0.258547	-6.54785	5.84E-11	1.02E-09	TRUE	Igfbpl1
Pcdh17	208.3145	-1.128783915	0.34984	-3.22657	0.001253	0.004694	TRUE	Pcdh17
Dcaf10	301.9787	-1.151822491	0.29156	-3.95055	7.80E-05	0.000407	TRUE	Dcaf10

Kcnh8	79.223	-2.585257523	0.617135	-4.18913	2.80E-05	0.000163	TRUE	Kcnh8
Chrna5	4.007313	-4.14289441	1.675581	-2.47251	0.013417	0.035664	TRUE	Chrna5
Ric8b	432.9729	-1.587287075	0.292731	-5.42233	5.88E-08	6.33E-07	TRUE	Ric8b
Midn	3859.885	-1.315841767	0.265955	-4.9476	7.51E-07	6.35E-06	TRUE	Midn
Cbarp	1490.364	-1.700437597	0.292002	-5.82337	5.77E-09	7.48E-08	TRUE	Cbarp
Gtf3c4	902.216	-1.122120641	0.2263	-4.95856	7.10E-07	6.05E-06	TRUE	Gtf3c4
Kcnc2	68.50917	-1.652054898	0.512581	-3.22301	0.001269	0.004747	TRUE	Kcnc2
Rnf38	2333.525	-1.197259521	0.235001	-5.0947	3.49E-07	3.16E-06	TRUE	Rnf38
Cd99l2	747.1146	-1.456488629	0.188459	-7.72841	1.09E-14	3.16E-13	TRUE	Cd99l2
Zdhhc17	795.7761	-1.051328754	0.188278	-5.58392	2.35E-08	2.74E-07	TRUE	Zdhhc17
Ids	836.1065	-3.074901978	0.306946	-10.0177	1.27E-23	9.48E-22	TRUE	Ids
Syt1	561.768	-3.462160839	0.480278	-7.20866	5.65E-13	1.31E-11	TRUE	Syt1
Dcdc2a	10.80418	-5.572689624	1.368076	-4.07338	4.63E-05	0.000256	TRUE	Dcdc2a
Ibtk	709.3841	-1.036516679	0.256159	-4.04639	5.20E-05	0.000283	TRUE	Ibtk
Gsx2	11.12283	-5.616633951	1.400974	-4.00909	6.10E-05	0.000327	TRUE	Gsx2
Dock4	657.8044	-2.137953642	0.340035	-6.28746	3.23E-10	5.09E-09	TRUE	Dock4
Tmem59l	141.6941	-1.710969794	0.522841	-3.27245	0.001066	0.004073	TRUE	Tmem59l
Rusc2	1814.64	-3.101550888	0.256343	-12.0992	1.07E-33	2.20E-31	TRUE	Rusc2
Fam214b	1033.529	-1.490812797	0.182977	-8.14753	3.71E-16	1.27E-14	TRUE	Fam214b
Ripor2	126.0109	-2.172234245	0.538928	-4.03066	5.56E-05	0.000301	TRUE	Ripor2
Tmtc2	332.8929	-1.943996181	0.258444	-7.52192	5.40E-14	1.42E-12	TRUE	Tmtc2
Prtg	46.40121	-2.386724752	0.50954	-4.68408	2.81E-06	2.08E-05	TRUE	Prtg
)31439G07F	2236.337	-1.848568599	0.229693	-8.04798	8.42E-16	2.79E-14	TRUE	5031439G07Rik
Smug1	253.4431	-1.060130887	0.318507	-3.32843	0.000873	0.003424	TRUE	Smug1
Arl5a	771.1992	-1.075190686	0.26664	-4.03236	5.52E-05	0.000299	TRUE	Arl5a
Myrf	32.34594	-3.08823016	0.628759	-4.91163	9.03E-07	7.49E-06	TRUE	Myrf
Prr5	41.46274	-2.533117277	0.571143	-4.43517	9.20E-06	6.03E-05	TRUE	Prr5
Mgat5	763.5313	-1.897978544	0.242688	-7.82065	5.26E-15	1.61E-13	TRUE	Mgat5
Surf6	365.5812	-1.089430374	0.245171	-4.44356	8.85E-06	5.83E-05	TRUE	Surf6
Pphln1	422.2224	-1.411191328	0.482003	-2.92777	0.003414	0.011212	TRUE	Pphln1
Rorb	167.7527	-1.815631153	0.341264	-5.3203	1.04E-07	1.06E-06	TRUE	Rorb
Arhgap36	29.01363	-3.584250784	0.760038	-4.71588	2.41E-06	1.80E-05	TRUE	Arhgap36
Sh3bp4	259.608	-1.241020052	0.392798	-3.15944	0.001581	0.005752	TRUE	Sh3bp4
Pdzrn4	183.1198	-3.630771311	0.356077	-10.1966	2.05E-24	1.68E-22	TRUE	Pdzrn4
Kctd1	213.9325	-1.036176306	0.387937	-2.67099	0.007563	0.02197	TRUE	Kctd1
Armh4	93.74959	-2.378732701	0.51275	-4.63917	3.50E-06	2.53E-05	TRUE	Armh4
Igfbp7	86.81188	-1.168867628	0.475403	-2.45869	0.013945	0.036837	TRUE	Igfbp7
Pnpla8	722.7247	-1.268001384	0.242349	-5.23213	1.68E-07	1.63E-06	TRUE	Pnpla8
Naa30	553.9229	-1.426162144	0.306566	-4.65206	3.29E-06	2.39E-05	TRUE	Naa30
Ap5m1	161.6522	-1.602194654	0.412575	-3.8834	0.000103	0.00052	TRUE	Ap5m1
Lrrn3	505.5665	-1.846730032	0.350797	-5.26438	1.41E-07	1.39E-06	TRUE	Lrrn3
Kidins220	3647.038	-1.898870377	0.217723	-8.7215	2.75E-18	1.17E-16	TRUE	Kidins220
Igsf10	59.7224	-1.245229159	0.459279	-2.71127	0.006703	0.019844	TRUE	Igsf10
i30008M17I	1863.356	-1.206734594	0.227637	-5.30115	1.15E-07	1.16E-06	TRUE	C530008M17Rik
Pcdh8	563.4451	-2.858392964	0.343815	-8.31376	9.27E-17	3.40E-15	TRUE	Pcdh8
Arhgap26	96.97897	-2.035858345	0.522289	-3.89796	9.70E-05	0.000494	TRUE	Arhgap26
Megf11	432.0806	-2.713486811	0.312714	-8.67721	4.06E-18	1.70E-16	TRUE	Megf11
1-Mar	134.7168	-2.202765727	0.53187	-4.14155	3.45E-05	0.000197	TRUE	1-Mar
Tbc1d24	729.2743	-1.759000884	0.234361	-7.50552	6.12E-14	1.59E-12	TRUE	Tbc1d24
Rnf39	5.53567	-3.552281861	1.421406	-2.49913	0.01245	0.033539	TRUE	Rnf39
Tmem255a	39.46385	-4.369924242	0.794476	-5.50038	3.79E-08	4.21E-07	TRUE	Tmem255a
Cdh8	375.2907	-2.147311354	0.344278	-6.23714	4.46E-10	6.91E-09	TRUE	Cdh8
Greb1	131.6163	-2.173548087	0.333251	-6.52226	6.93E-11	1.20E-09	TRUE	Greb1
Rnf113a1	48.82864	-1.338465853	0.498259	-2.68628	0.007225	0.021136	TRUE	Rnf113a1
Ndrg4	576.1362	-2.428509968	0.226927	-10.7017	9.99E-27	9.96E-25	TRUE	Ndrg4
Bicral	363.7092	-1.129549054	0.282937	-3.99223	6.55E-05	0.000348	TRUE	Bicral
Fxyd7	9.647338	-5.410806829	1.42747	-3.79049	0.00015	0.000727	TRUE	Fxyd7
Chst12	100.4477	-1.056583357	0.343765	-3.07356	0.002115	0.00743	TRUE	Chst12
Mag	6.91557	-4.93017492	1.481364	-3.32813	0.000874	0.003426	TRUE	Mag
Colec11	6.568505	-4.849455412	1.502274	-3.22808	0.001246	0.004676	TRUE	Colec11
Tcaf1	2981.995	-1.81793683	0.179787	-10.1116	4.91E-24	3.87E-22	TRUE	Tcaf1
Zcchc12	550.2562	-4.960896598	0.428119	-11.5877	4.76E-31	8.00E-29	TRUE	Zcchc12
Cab39	1375.076	-1.611634973	0.344035	-4.68451	2.81E-06	2.07E-05	TRUE	Cab39
Cyld	288.4167	-1.41333299	0.285093	-4.95744	7.14E-07	6.08E-06	TRUE	Cyld
Micall2	140.7935	-1.927217969	0.548731	-3.51214	0.000445	0.001887	TRUE	Micall2
Ttll7	190.3766	-2.507491658	0.335925	-7.46444	8.37E-14	2.14E-12	TRUE	Ttll7
Kcnk9	5.577518	-4.621709201	1.693144	-2.72966	0.00634	0.018981	TRUE	Kcnk9
Dnajc12	23.47642	-2.722862109	0.735779	-3.70065	0.000215	0.000998	TRUE	Dnajc12
Dner	799.2081	-5.140914243	0.29227	-17.5896	2.96E-69	5.14E-66	TRUE	Dner
Wdr44	85.5889	-1.207441783	0.504552	-2.39309	0.016707	0.042789	TRUE	Wdr44
Klhl13	660.4502	-3.031084837	0.306796	-9.87979	5.09E-23	3.56E-21	TRUE	Klhl13
Fam135b	17.45308	-4.003130548	1.102539	-3.63083	0.000283	0.001269	TRUE	Fam135b

Dpp10	52.08927	-1.463957782	0.495035	-2.95728	0.003104	0.010329	TRUE	Dpp10
Plch1	337.8611	-1.602868496	0.44913	-3.56883	0.000359	0.00156	TRUE	Plch1
Gtdc1	253.8415	-1.036057372	0.266695	-3.8848	0.000102	0.000518	TRUE	Gtdc1
Neto2	1125.838	-2.496253922	0.184391	-13.5378	9.35E-42	3.19E-39	TRUE	Neto2
Trim67	1417.301	-4.025957105	0.409609	-9.82878	8.46E-23	5.76E-21	TRUE	Trim67
Stox1	22.26322	-1.570290522	0.669605	-2.3451	0.019022	0.047618	TRUE	Stox1
Lrfn3	341.2843	-1.29073053	0.277927	-4.64413	3.42E-06	2.47E-05	TRUE	Lrfn3
Bcorl1	531.5583	-1.125557681	0.210671	-5.34273	9.16E-08	9.43E-07	TRUE	Bcorl1
Wnt8b	6.804618	-4.897268214	1.923528	-2.54598	0.010897	0.029985	TRUE	Wnt8b
Zic4	3449.317	-5.457074329	1.622051	-3.3643	0.000767	0.003053	TRUE	Zic4
Zdhhc9	454.8947	-2.272244236	0.267221	-8.50324	1.84E-17	7.22E-16	TRUE	Zdhhc9
Hk1	649.4733	-1.654529213	0.208565	-7.93291	2.14E-15	6.79E-14	TRUE	Hk1
Tspan15	123.4323	-1.094927062	0.383983	-2.8515	0.004351	0.013765	TRUE	Tspan15
Sh3glb1	1452.684	-1.532155346	0.196382	-7.80193	6.10E-15	1.85E-13	TRUE	Sh3glb1
Scd1	193.5526	-2.41609066	0.483347	-4.99867	5.77E-07	5.01E-06	TRUE	Scd1
Rnf139	129.9096	-1.026700863	0.426261	-2.40862	0.016013	0.041302	TRUE	Rnf139
Psd	1641.415	-1.443614037	0.388425	-3.71658	0.000202	0.000944	TRUE	Psd
Aff3	763.7504	-1.051432874	0.196229	-5.3582	8.41E-08	8.73E-07	TRUE	Aff3
Lrrc20	329.4878	-1.434218053	0.35104	-4.08562	4.40E-05	0.000245	TRUE	Lrrc20
Cyb561d2	73.46902	-1.158334464	0.38533	-3.00608	0.002646	0.009029	TRUE	Cyb561d2
Spry1	46.36809	-2.705991662	0.542226	-4.99053	6.02E-07	5.21E-06	TRUE	Spry1
Syn1	484.7644	-2.597962748	0.268967	-9.65905	4.50E-22	2.82E-20	TRUE	Syn1
Mospd3	397.8333	-1.200704539	0.295119	-4.06855	4.73E-05	0.000261	TRUE	Mospd3
Aagab	468.7023	-1.689293996	0.271718	-6.21708	5.06E-10	7.80E-09	TRUE	Aagab
Dzank1	141.3167	-3.000354017	0.376134	-7.97682	1.50E-15	4.87E-14	TRUE	Dzank1
Man1c1	121.2728	-2.028771716	0.466928	-4.34494	1.39E-05	8.78E-05	TRUE	Man1c1
Paqr7	56.08702	-4.278766301	0.693257	-6.17198	6.74E-10	1.02E-08	TRUE	Paqr7
Ctbp1	4398.792	-1.012318422	0.292401	-3.46209	0.000536	0.002219	TRUE	Ctbp1
Endod1	67.02988	-1.631880579	0.50594	-3.22544	0.001258	0.004709	TRUE	Endod1
Vgf	65.10998	-3.274728396	0.903487	-3.62454	0.000289	0.001297	TRUE	Vgf
Tulp1	30.38457	-1.603402992	0.586571	-2.73352	0.006266	0.018787	TRUE	Tulp1
Azin1	1146.556	-1.281181465	0.416787	-3.07395	0.002112	0.007424	TRUE	Azin1
Fbxo27	40.06104	-1.49484386	0.516594	-2.89365	0.003808	0.012305	TRUE	Fbxo27
Zmat4	29.69159	-3.890541568	0.817184	-4.76091	1.93E-06	1.47E-05	TRUE	Zmat4
Nenf	262.3112	-1.212757489	0.334458	-3.62604	0.000288	0.00129	TRUE	Nenf
Shank2	414.3206	-1.788734694	0.343326	-5.21002	1.89E-07	1.82E-06	TRUE	Shank2
Vash2	766.712	-1.535874298	0.275187	-5.58121	2.39E-08	2.78E-07	TRUE	Vash2
Pkd2l1	8.053459	-3.33282723	1.317441	-2.52977	0.011414	0.031174	TRUE	Pkd2l1
Gch1	7.284205	-5.003147631	1.447618	-3.45612	0.000548	0.002265	TRUE	Gch1
Clba1	87.8273	-1.655417041	0.347704	-4.761	1.93E-06	1.47E-05	TRUE	Clba1
Kcnmb2	70.33541	-5.704859967	1.70546	-3.34506	0.000823	0.00325	TRUE	Kcnmb2
Wdtc1	773.5901	-1.270709631	0.185199	-6.86133	6.82E-12	1.37E-10	TRUE	Wdtc1
Zbtb42	63.61591	-1.238517226	0.378058	-3.276	0.001053	0.004029	TRUE	Zbtb42
Zfp60	1181.391	-1.56808404	0.238731	-6.56841	5.09E-11	9.02E-10	TRUE	Zfp60
Kctd8	5.648467	-4.638124232	1.521355	-3.04868	0.002298	0.007995	TRUE	Kctd8
Cdkn1c	1115.971	-1.169225821	0.249434	-4.68751	2.77E-06	2.05E-05	TRUE	Cdkn1c
Atp8a1	609.842	-1.18090674	0.205845	-5.73688	9.64E-09	1.20E-07	TRUE	Atp8a1
Fam13a	77.04363	-1.043346237	0.360636	-2.89307	0.003815	0.012324	TRUE	Fam13a
Tmem33	703.6596	-1.537827018	0.403327	-3.81286	0.000137	0.000672	TRUE	Tmem33
Eef1a1	52428.23	-1.002394061	0.282127	-3.55299	0.000381	0.001644	TRUE	Eef1a1
Phyhipl	277.703	-2.48487112	0.379853	-6.54166	6.08E-11	1.06E-09	TRUE	Phyhipl
Fam222b	562.1771	-1.364076753	0.21635	-6.30494	2.88E-10	4.57E-09	TRUE	Fam222b
Slc32a1	185.1788	-2.965338488	0.530095	-5.59398	2.22E-08	2.60E-07	TRUE	Slc32a1
Vopp1	1047.593	-1.974840945	0.415007	-4.75857	1.95E-06	1.49E-05	TRUE	Vopp1
Smim14	373.2563	-2.125722384	0.327652	-6.48773	8.71E-11	1.47E-09	TRUE	Smim14
Vstm2l	170.3372	-5.449094198	0.60414	-9.01959	1.89E-19	9.26E-18	TRUE	Vstm2l
Cpe	2512.261	-2.12652707	0.372062	-5.71552	1.09E-08	1.36E-07	TRUE	Cpe
Zfp365	149.3903	-2.709586521	0.575422	-4.70887	2.49E-06	1.86E-05	TRUE	Zfp365
Dusp8	1214.746	-2.188983528	0.259038	-8.45043	2.90E-17	1.12E-15	TRUE	Dusp8
Pcdh18	220.0644	-1.315062463	0.326058	-4.03321	5.50E-05	0.000298	TRUE	Pcdh18
Fgd3	150.6594	-1.488752616	0.530039	-2.80876	0.004973	0.015444	TRUE	Fgd3
430571L13R	63.34879	-2.39229922	0.522983	-4.57434	4.78E-06	3.34E-05	TRUE	6430571L13Rik
Parp11	779.3341	-1.02515882	0.212301	-4.82881	1.37E-06	1.09E-05	TRUE	Parp11
Acer2	90.15642	-2.967484089	0.394311	-7.52575	5.24E-14	1.38E-12	TRUE	Acer2
Rapgefl1	237.8181	-1.284965267	0.384788	-3.33941	0.00084	0.003307	TRUE	Rapgefl1
Phf2	1291.908	-1.109787212	0.292941	-3.78843	0.000152	0.000732	TRUE	Phf2
Mturn	1908.066	-2.602912347	0.323536	-8.0452	8.61E-16	2.85E-14	TRUE	Mturn
Cdkn2aip	265.4675	-1.324316679	0.379951	-3.4855	0.000491	0.002058	TRUE	Cdkn2aip
Kcna6	165.0924	-4.467298792	0.567691	-7.86924	3.57E-15	1.10E-13	TRUE	Kcna6
Opa1	724.252	-1.311648908	0.275946	-4.75327	2.00E-06	1.52E-05	TRUE	Opa1
AW551984	95.63927	-8.718133611	1.242488	-7.01667	2.27E-12	4.93E-11	TRUE	AW551984
Atg5	200.0489	-1.095808662	0.31444	-3.48495	0.000492	0.002061	TRUE	Atg5

Pde4dip	784.5674	-1.829775776	0.24651	-7.42273	1.15E-13	2.91E-12	TRUE		Pde4dip
Mylip	22.37192	-3.094275863	0.847113	-3.65273	0.000259	0.001179	TRUE		Mylip
Slc43a2	387.0143	-1.301177223	0.292244	-4.45236	8.49E-06	5.61E-05	TRUE		Slc43a2
Btbd10	357.34	-1.323245273	0.251594	-5.25944	1.44E-07	1.43E-06	TRUE		Btbd10
Sobp	1242.257	-2.128185273	0.356173	-5.97514	2.30E-09	3.14E-08	TRUE		Sobp
Slc22a23	1293.683	-1.441227027	0.180858	-7.96883	1.60E-15	5.16E-14	TRUE		Slc22a23
Atg9b	8.760861	-2.715842205	1.166596	-2.32801	0.019912	0.049466	TRUE		Atg9b
Snx10	474.1694	-1.357419718	0.265276	-5.11701	3.10E-07	2.84E-06	TRUE		Snx10
Spats2l	178.251	-3.32591294	0.363295	-9.15487	5.44E-20	2.88E-18	TRUE		Spats2l
Kcnh2	969.3	-3.135379151	0.540782	-5.79786	6.72E-09	8.63E-08	TRUE		Kcnh2
Tsr1	158.4131	-1.017918183	0.292304	-3.4824	0.000497	0.002079	TRUE		Tsr1
Ankrd35	16.23543	-5.210784582	1.242636	-4.19333	2.75E-05	0.000161	TRUE		Ankrd35
Lasp1	1627.823	-1.829259885	0.230903	-7.92221	2.33E-15	7.37E-14	TRUE		Lasp1
Ncoa6	883.05	-1.076273411	0.241952	-4.44829	8.66E-06	5.72E-05	TRUE		Ncoa6
Pcp4l1	10.87686	-5.585056826	1.45195	-3.84659	0.00012	0.000594	TRUE		Pcp4l1
Trp53inp2	2045.873	-2.448895333	0.216542	-11.3091	1.18E-29	1.67E-27	TRUE		Trp53inp2
Hjv	4.939266	-4.440694919	1.555723	-2.85443	0.004311	0.013668	TRUE		Hjv
Foxq1	17.16103	-1.96791772	0.717539	-2.74259	0.006096	0.018371	TRUE		Foxq1
Fig4	183.7287	-1.302740893	0.321864	-4.04748	5.18E-05	0.000282	TRUE		Fig4
Egr1	9.283073	-3.020179026	1.141197	-2.6465	0.008133	0.023397	TRUE		Egr1
Hdhd3	28.51231	-5.328017138	1.0437	-5.10493	3.31E-07	3.01E-06	TRUE		Hdhd3
Spsb2	44.16824	-1.23633317	0.475251	-2.60143	0.009284	0.026169	TRUE		Spsb2
Dennd2a	985.6064	-1.109087116	0.261	-4.24938	2.14E-05	0.000129	TRUE		Dennd2a
Abhd17c	536.1487	-1.378667815	0.307334	-4.48589	7.26E-06	4.86E-05	TRUE		Abhd17c
Uqcrfs1	699.0777	-1.180687314	0.373693	-3.15951	0.00158	0.005752	TRUE		Uqcrfs1
Sv2a	640.2593	-2.239854296	0.404995	-5.53057	3.19E-08	3.61E-07	TRUE		Sv2a
Jarid2	1028.505	-1.25616867	0.217558	-5.77394	7.74E-09	9.82E-08	TRUE		Jarid2
Rgs4	78.39446	-2.245688563	0.553379	-4.05814	4.95E-05	0.000271	TRUE		Rgs4
Susd4	412.1798	-1.575260408	0.49833	-3.16108	0.001572	0.00573	TRUE		Susd4
Akap12	285.0937	-1.36034607	0.376994	-3.6084	0.000308	0.00137	TRUE		Akap12
Samd10	626.1692	-2.336029436	0.213257	-10.9541	6.35E-28	7.20E-26	TRUE		Samd10
Dock10	31.62049	-1.222749731	0.497035	-2.46009	0.01389	0.036717	TRUE		Dock10
Med30	144.5002	-1.134623364	0.319282	-3.55368	0.00038	0.00164	TRUE		Med30
Polr3k	785.3282	-1.128819774	0.336466	-3.35493	0.000794	0.003149	TRUE		Polr3k
Ramac	419.3426	-1.291068673	0.368296	-3.50552	0.000456	0.001927	TRUE		Ramac
Herc1	2110.918	-1.068974351	0.2319	-4.60964	4.03E-06	2.87E-05	TRUE		Herc1
Scube3	410.6236	-2.893931968	0.40504	-7.1448	9.01E-13	2.04E-11	TRUE		Scube3
Pbx3	475.7896	-3.291841571	0.290659	-11.3255	9.82E-30	1.41E-27	TRUE		Pbx3
Wdr26	1969.281	-1.102353283	0.23933	-4.60601	4.10E-06	2.91E-05	TRUE		Wdr26
Shank1	1838.085	-2.19201646	0.391017	-5.60594	2.07E-08	2.44E-07	TRUE		Shank1
Mvb12b	2945.352	-1.627998643	0.203298	-8.00796	1.17E-15	3.80E-14	TRUE		Mvb12b
Smurf1	864.8907	-1.549478122	0.201447	-7.69174	1.45E-14	4.13E-13	TRUE		Smurf1
Hace1	203.1828	-1.504458914	0.342114	-4.39754	1.09E-05	7.05E-05	TRUE		Hace1
Ralgps1	1030.25	-1.155803656	0.316214	-3.65513	0.000257	0.00117	TRUE		Ralgps1
Agbl3	40.13781	-2.073646559	0.558995	-3.7096	0.000208	0.000966	TRUE		Agbl3
Bpgm	623.5479	-2.143709456	0.348962	-6.1431	8.09E-10	1.20E-08	TRUE		Bpgm
Zfhx3	5481.292	-4.106745763	0.407036	-10.0894	6.15E-24	4.77E-22	TRUE		Zfhx3
Rnf146	140.7765	-1.90420366	0.404986	-4.7019	2.58E-06	1.92E-05	TRUE		Rnf146
Ctu1	253.4517	-1.001288456	0.241127	-4.15253	3.29E-05	0.000189	TRUE		Ctu1
Irs2	403.5985	-1.989991314	0.341925	-5.81996	5.89E-09	7.63E-08	TRUE		Irs2
Soga3	1375.783	-1.329935099	0.353532	-3.76185	0.000169	0.000805	TRUE		Soga3
930402G23R	3.642262	-4.000867229	1.679735	-2.38184	0.017226	0.043914	TRUE		3930402G23Rik
Sccpdh	337.3128	-1.328466653	0.321773	-4.12859	3.65E-05	0.000207	TRUE		Sccpdh
Cnst	572.9125	-1.062368913	0.227727	-4.6651	3.08E-06	2.26E-05	TRUE		Cnst
Lmtk2	570.1668	-1.267132342	0.242148	-5.23289	1.67E-07	1.63E-06	TRUE		Lmtk2
Ppp1r9b	2098.977	-1.634131908	0.268719	-6.08118	1.19E-09	1.71E-08	TRUE		Ppp1r9b
Tspyl5	145.7743	-2.872748971	0.446627	-6.4321	1.26E-10	2.10E-09	TRUE		Tspyl5
Bmp6	4.583002	-4.338834761	1.733804	-2.50249	0.012332	0.033278	TRUE		Bmp6
Cpq	9.206839	-3.693897676	1.341661	-2.75323	0.005901	0.017885	TRUE		Cpq
Arhgap18	456.0025	-3.204560637	0.355016	-9.02652	1.77E-19	8.73E-18	TRUE		Arhgap18
St6galnac5	208.9641	-6.039663983	0.667536	-9.0477	1.46E-19	7.30E-18	TRUE		St6galnac5
Arpin	137.9635	-1.483375017	0.349463	-4.24473	2.19E-05	0.000131	TRUE		Arpin
Pigk	475.7743	-1.744784703	0.415439	-4.19986	2.67E-05	0.000157	TRUE		Pigk
Myo16	323.7517	-3.049564581	0.449829	-6.77938	1.21E-11	2.35E-10	TRUE		Myo16
Ak5	44.97871	-3.84902112	0.654073	-5.8847	3.99E-09	5.27E-08	TRUE		Ak5
Hrh3	167.3281	-2.860945991	0.604023	-4.73649	2.17E-06	1.64E-05	TRUE		Hrh3
Zfp503	153.6923	-5.88580739	1.355475	-4.34225	1.41E-05	8.88E-05	TRUE		Zfp503
Ss18l1	929.2018	-1.434433356	0.264402	-5.4252	5.79E-08	6.24E-07	TRUE		Ss18l1
6-Mar	2084.325	-2.142706683	0.322056	-6.65321	2.87E-11	5.29E-10	TRUE		6-Mar
Atp6v1g1	1080.413	-1.035995425	0.394945	-2.62314	0.008712	0.024828	TRUE		Atp6v1g1
Htr5a	13.25915	-3.384784851	1.213219	-2.78992	0.005272	0.016253	TRUE		Htr5a
Mycbpap	65.96421	-2.251298776	0.58758	-3.83148	0.000127	0.000629	TRUE		Mycbpap

Adgrg6	8.849633	-5.281712813	1.404372	-3.76091	0.000169	0.000808	TRUE	Adgrg6
Gipc2	6.645148	-4.872488837	1.491665	-3.26648	0.001089	0.004152	TRUE	Gipc2
Camk1d	166.2	-2.102310041	0.367056	-5.72749	1.02E-08	1.27E-07	TRUE	Camk1d
Ube2h	1204.688	-1.277674048	0.276938	-4.61358	3.96E-06	2.82E-05	TRUE	Ube2h
AW209491	114.2546	-1.061860683	0.335749	-3.16266	0.001563	0.005706	TRUE	AW209491
Abhd2	754.782	-1.500831252	0.288151	-5.20849	1.90E-07	1.83E-06	TRUE	Abhd2
Fn3krp	171.2746	-1.317680299	0.256938	-5.1284	2.92E-07	2.69E-06	TRUE	Fn3krp
Vstm2b	245.1874	-1.767167726	0.371592	-4.75567	1.98E-06	1.51E-05	TRUE	Vstm2b
Prrc2b	5963.536	-1.008689935	0.245387	-4.11061	3.95E-05	0.000222	TRUE	Prrc2b
Megf9	856.505	-1.81742864	0.355095	-5.11815	3.09E-07	2.83E-06	TRUE	Megf9
Pcsk1n	1252.327	-3.260907901	0.4333	-7.52574	5.24E-14	1.38E-12	TRUE	Pcsk1n
Rftn1	187.7433	-3.866495301	0.634973	-6.08923	1.13E-09	1.64E-08	TRUE	Rftn1
Atp6v0e2	835.3044	-1.246916966	0.268439	-4.64507	3.40E-06	2.46E-05	TRUE	Atp6v0e2
Picalm	1520.204	-1.059397298	0.253379	-4.18108	2.90E-05	0.000168	TRUE	Picalm
4-Mar	249.8502	-3.954196953	0.565759	-6.98919	2.76E-12	5.90E-11	TRUE	4-Mar
Wdr17	98.94648	-1.444360653	0.313476	-4.60756	4.07E-06	2.89E-05	TRUE	Wdr17
Heatr5b	330.1158	-1.012676715	0.268942	-3.7654	0.000166	0.000795	TRUE	Heatr5b
Cntnap2	588.3097	-1.875718115	0.312715	-5.99817	2.00E-09	2.75E-08	TRUE	Cntnap2
Tcta	170.0382	-1.822057917	0.511766	-3.56034	0.00037	0.001605	TRUE	Tcta
Zdhhc2	302.0551	-1.824941083	0.374398	-4.87433	1.09E-06	8.89E-06	TRUE	Zdhhc2
Tspyl4	942.0502	-3.576981451	0.342346	-10.4485	1.49E-25	1.32E-23	TRUE	Tspyl4
Cntn5	34.79082	-3.022929	0.633933	-4.76853	1.86E-06	1.42E-05	TRUE	Cntn5
Cdnf	4.532387	-4.315665847	1.58171	-2.72848	0.006363	0.019034	TRUE	Cdnf
Dse	32.5618	-2.205298263	0.658911	-3.34688	0.000817	0.003231	TRUE	Dse
Uhrf1bp1	435.5445	-1.001697645	0.27169	-3.68692	0.000227	0.001049	TRUE	Uhrf1bp1
Tusc3	560.8397	-2.614918814	0.31778	-8.22871	1.89E-16	6.71E-15	TRUE	Tusc3
Ncam1	7214.045	-1.246216916	0.256402	-4.86041	1.17E-06	9.45E-06	TRUE	Ncam1
Ajap1	70.08119	-4.357022662	0.612494	-7.11358	1.13E-12	2.54E-11	TRUE	Ajap1
Grin3a	43.12221	-4.438878357	0.721459	-6.15264	7.62E-10	1.14E-08	TRUE	Grin3a
Rcan2	540.8733	-4.830977426	0.533146	-9.06126	1.29E-19	6.51E-18	TRUE	Rcan2
Rbms3	173.9343	-1.272065504	0.359658	-3.53687	0.000405	0.001734	TRUE	Rbms3
Tmem246	229.5846	-1.978298369	0.281125	-7.03707	1.96E-12	4.28E-11	TRUE	Tmem246
Lonrf1	142.903	-1.547889985	0.608141	-2.54528	0.010919	0.030032	TRUE	Lonrf1
Zfp189	101.8104	-1.403430993	0.41076	-3.41667	0.000634	0.002579	TRUE	Zfp189
Cpeb3	104.5675	-3.624029877	0.475036	-7.62896	2.37E-14	6.54E-13	TRUE	Cpeb3
Dusp26	398.7317	-4.004022543	0.375539	-10.6621	1.53E-26	1.47E-24	TRUE	Dusp26
Sdk1	93.10303	-1.635218221	0.364223	-4.48961	7.14E-06	4.78E-05	TRUE	Sdk1
Gm5422	10.9697	-2.298471802	0.928119	-2.47648	0.013268	0.035315	TRUE	Gm5422
Ncoa7	219.3749	-1.290583139	0.282954	-4.56111	5.09E-06	3.53E-05	TRUE	Ncoa7
Lmbrd2	329.2093	-1.042004011	0.289074	-3.60463	0.000313	0.001387	TRUE	Lmbrd2
Ldb2	112.5892	-1.761548359	0.325686	-5.40874	6.35E-08	6.77E-07	TRUE	Ldb2
Ralyl	135.4675	-1.688365745	0.287423	-5.87414	4.25E-09	5.59E-08	TRUE	Ralyl
Fnbp1l	4565.426	-1.680931586	0.211646	-7.9422	1.99E-15	6.33E-14	TRUE	Fnbp1l
Alg2	1400.424	-2.664661312	0.351876	-7.57274	3.65E-14	9.85E-13	TRUE	Alg2
Bahcc1	1657.268	-1.383442716	0.212573	-6.50809	7.61E-11	1.31E-09	TRUE	Bahcc1
Orai2	325.1882	-2.272167326	0.289623	-7.84526	4.32E-15	1.33E-13	TRUE	Orai2
Dnajc28	62.79976	-1.510428248	0.419227	-3.60289	0.000315	0.001395	TRUE	Dnajc28
Ypel5	445.2135	-1.315701223	0.247981	-5.30565	1.12E-07	1.14E-06	TRUE	Ypel5
Cpeb2	211.0476	-2.925128804	0.352913	-8.28853	1.15E-16	4.15E-15	TRUE	Cpeb2
Zfp597	195.954	-1.779386258	0.345121	-5.15584	2.52E-07	2.36E-06	TRUE	Zfp597
Olig2	133.8125	-4.650625224	0.525154	-8.85574	8.31E-19	3.75E-17	TRUE	Olig2
Slc45a1	137.0716	-2.738944841	0.401337	-6.82455	8.82E-12	1.75E-10	TRUE	Slc45a1
Srrm3	1265.505	-1.075625919	0.406606	-2.64538	0.00816	0.023459	TRUE	Srrm3
Txlnb	14.46248	-2.099406575	0.767154	-2.73662	0.006207	0.018643	TRUE	Txlnb
Cited2	1076.795	-2.012359822	0.27479	-7.32327	2.42E-13	5.84E-12	TRUE	Cited2
Pak7	523.6885	-2.083304011	0.381865	-5.45561	4.88E-08	5.34E-07	TRUE	Pak7
Rhbdd2	513.308	-1.022696414	0.194787	-5.25033	1.52E-07	1.49E-06	TRUE	Rhbdd2
Pik3cd	448.1696	-1.742935977	0.424661	-4.1043	4.06E-05	0.000227	TRUE	Pik3cd
Dag1	808.0121	-1.258250697	0.351829	-3.57631	0.000348	0.001521	TRUE	Dag1
Stk32a	75.53535	-2.165770298	0.43106	-5.02429	5.05E-07	4.45E-06	TRUE	Stk32a
Rhou	1933.384	-3.34387747	0.361975	-9.23786	2.51E-20	1.37E-18	TRUE	Rhou
Tbc1d16	3531.069	-1.448270225	0.283566	-5.10735	3.27E-07	2.98E-06	TRUE	Tbc1d16
Dtx4	1066.809	-2.465025127	0.319207	-7.72234	1.14E-14	3.30E-13	TRUE	Dtx4
Phtf2	255.0191	-1.112493419	0.299549	-3.7139	0.000204	0.000953	TRUE	Phtf2
Magi2	791.4063	-2.142991375	0.324505	-6.60388	4.01E-11	7.26E-10	TRUE	Magi2
Gnaz	413.9732	-1.978349241	0.255551	-7.74151	9.82E-15	2.87E-13	TRUE	Gnaz
Slc7a5	649.524	-2.025706306	0.42281	-4.79106	1.66E-06	1.29E-05	TRUE	Slc7a5
Cox15	287.073	-1.03127072	0.299066	-3.4483	0.000564	0.002325	TRUE	Cox15
Rab11fip2	285.0478	-1.069436842	0.345947	-3.09133	0.001993	0.007043	TRUE	Rab11fip2
Disp2	782.1945	-2.966439087	0.404446	-7.33457	2.22E-13	5.40E-12	TRUE	Disp2
Klhl42	234.0735	-1.411782064	0.357575	-3.94821	7.87E-05	0.00041	TRUE	Klhl42
Plpp6	85.48858	-1.744532024	0.442749	-3.94023	8.14E-05	0.000422	TRUE	Plpp6

Rgl3	33.3318	-3.975122071	0.870585	-4.56604	4.97E-06	3.46E-05	TRUE	Rgl3
Tax1bp3	305.7251	-2.227668671	0.248268	-8.97283	2.89E-19	1.39E-17	TRUE	Tax1bp3
Fmo1	11.60342	-5.670517938	1.433491	-3.95574	7.63E-05	0.000399	TRUE	Fmo1
Dnm3	245.9692	-3.092892576	0.537981	-5.74907	8.97E-09	1.12E-07	TRUE	Dnm3
Plekha1	909.206	-1.12410014	0.252778	-4.44699	8.71E-06	5.75E-05	TRUE	Plekha1
Pacsin1	151.5605	-1.893020245	0.478731	-3.95424	7.68E-05	0.000401	TRUE	Pacsin1
Slc25a24	107.4976	-2.54578412	0.416039	-6.11909	9.41E-10	1.39E-08	TRUE	Slc25a24
Fam102b	296.2794	-1.283937081	0.447531	-2.86893	0.004119	0.013148	TRUE	Fam102b
Bcor	709.0179	-1.049009542	0.250761	-4.1833	2.87E-05	0.000167	TRUE	Bcor
Cacng5	24.04286	-2.707645561	0.92318	-2.93295	0.003358	0.011043	TRUE	Cacng5
Wdr47	897.2439	-1.101820409	0.194439	-5.66666	1.46E-08	1.76E-07	TRUE	Wdr47
Abhd13	268.4812	-1.176882746	0.370685	-3.17489	0.001499	0.005504	TRUE	Abhd13
330417C22R	107.6282	-1.52949918	0.405584	-3.77111	0.000163	0.00078	TRUE	5330417C22Rik
Spns2	253.9648	-1.248102418	0.351409	-3.5517	0.000383	0.001651	TRUE	Spns2
Cdh12	11.17167	-5.623612158	1.494685	-3.76241	0.000168	0.000804	TRUE	Cdh12
9-Mar	851.3543	-1.077322229	0.229028	-4.70389	2.55E-06	1.90E-05	TRUE	9-Mar
Manea	187.4289	-1.888730463	0.646971	-2.91934	0.003508	0.011492	TRUE	Manea
Necab1	14.13019	-5.962123626	1.44828	-4.11669	3.84E-05	0.000217	TRUE	Necab1
Adam22	444.4146	-1.219337614	0.23595	-5.16777	2.37E-07	2.22E-06	TRUE	Adam22
Tex2	334.7357	-1.712933403	0.229587	-7.46092	8.59E-14	2.20E-12	TRUE	Tex2
Otud6b	320.8139	-1.526209766	0.423307	-3.60544	0.000312	0.001384	TRUE	Otud6b
Wdr7	488.7942	-1.993777973	0.24483	-8.14353	3.84E-16	1.31E-14	TRUE	Wdr7
Rundc3b	282.5501	-3.039120374	0.375118	-8.10176	5.42E-16	1.82E-14	TRUE	Rundc3b
Pogk	1555.081	-1.226136678	0.21816	-5.62035	1.91E-08	2.25E-07	TRUE	Pogk
Ildr2	132.6112	-3.079361819	0.444453	-6.92843	4.26E-12	8.88E-11	TRUE	Ildr2
Erc2	675.3434	-2.333800774	0.40453	-5.76917	7.97E-09	1.00E-07	TRUE	Erc2
Ppip5k2	806.8439	-1.041549412	0.25288	-4.11875	3.81E-05	0.000215	TRUE	Ppip5k2
Ppp1r14c	42.42442	-3.566877436	0.837338	-4.25978	2.05E-05	0.000124	TRUE	Ppp1r14c
St8sia4	403.9847	-1.911962565	0.287824	-6.64281	3.08E-11	5.65E-10	TRUE	St8sia4
Sh3pxd2b	1482.678	-1.665626406	0.198702	-8.38254	5.18E-17	1.95E-15	TRUE	Sh3pxd2b
Il17rd	96.64828	-2.51949977	0.51662	-4.87689	1.08E-06	8.79E-06	TRUE	Il17rd
Kcna2	87.84197	-2.157957611	0.755956	-2.85461	0.004309	0.013662	TRUE	Kcna2
C1qtnf4	477.7225	-1.30230095	0.444235	-2.93156	0.003373	0.011084	TRUE	C1qtnf4
Iqsec3	308.6321	-5.215106302	0.425242	-12.2638	1.42E-34	3.14E-32	TRUE	Iqsec3
Gpr161	1019.455	-1.954486888	0.255688	-7.64402	2.11E-14	5.87E-13	TRUE	Gpr161
Reps2	71.95979	-2.781766602	0.707621	-3.93115	8.45E-05	0.000437	TRUE	Reps2
Dlk1	401.4042	-3.428317624	0.536331	-6.39217	1.64E-10	2.68E-09	TRUE	Dlk1
Osbpl10	39.10893	-2.721656902	0.537508	-5.06348	4.12E-07	3.69E-06	TRUE	Osbpl10
Atp1a3	1682.081	-2.641794734	0.342564	-7.71183	1.24E-14	3.57E-13	TRUE	Atp1a3
Rfx3	578.2958	-1.192650057	0.331164	-3.60139	0.000317	0.001401	TRUE	Rfx3
Cables1	76.41787	-1.499345947	0.514963	-2.91156	0.003596	0.011731	TRUE	Cables1
Sh3kbp1	389.0488	-2.350607712	0.234689	-10.0158	1.30E-23	9.63E-22	TRUE	Sh3kbp1
Iffo2	114.8072	-2.040204995	0.4901	-4.16284	3.14E-05	0.000181	TRUE	Iffo2
Ghitm	509.2495	-1.194624097	0.444161	-2.68962	0.007153	0.020955	TRUE	Ghitm
Irgq	1395.821	-1.795230701	0.193498	-9.27779	1.73E-20	9.57E-19	TRUE	Irgq
Fam117b	770.1696	-2.621477215	0.400364	-6.54774	5.84E-11	1.02E-09	TRUE	Fam117b
Nacad	2119.151	-1.371845031	0.39563	-3.4675	0.000525	0.002183	TRUE	Nacad
Grid1	60.79711	-1.299425754	0.438365	-2.96425	0.003034	0.010146	TRUE	Grid1
Ostc	484.4763	-1.086239648	0.439981	-2.46883	0.013555	0.035968	TRUE	Ostc
Elmo1	631.6969	-3.902969607	0.34362	-11.3584	6.74E-30	9.82E-28	TRUE	Elmo1
Msantd4	778.4436	-1.8495071	0.384621	-4.80865	1.52E-06	1.19E-05	TRUE	Msantd4
Pnmal1	803.0014	-5.040279474	0.373842	-13.4824	1.99E-41	6.57E-39	TRUE	Pnmal1
Phf8	485.9833	-1.436629028	0.27416	-5.24011	1.60E-07	1.57E-06	TRUE	Phf8
Wnk3	446.3217	-2.053626703	0.26425	-7.77153	7.75E-15	2.32E-13	TRUE	Wnk3
Car8	15.01937	-6.046133747	1.316035	-4.5942	4.34E-06	3.06E-05	TRUE	Car8
Rusc1	389.2826	-1.639119041	0.317876	-5.15647	2.52E-07	2.35E-06	TRUE	Rusc1
Tox	387.1807	-3.887581998	0.523586	-7.42492	1.13E-13	2.87E-12	TRUE	Tox
Sntb2	91.15425	-3.225639117	0.526608	-6.12531	9.05E-10	1.33E-08	TRUE	Sntb2
Slc7a1	369.5279	-1.280597578	0.44995	-2.84609	0.004426	0.013965	TRUE	Slc7a1
Atp1b2	266.3785	-1.232187985	0.323193	-3.81255	0.000138	0.000673	TRUE	Atp1b2
Ankrd42	83.47147	-1.45134767	0.513173	-2.82818	0.004681	0.014664	TRUE	Ankrd42
Pik3r1	531.8178	-1.889277897	0.271116	-6.96851	3.20E-12	6.78E-11	TRUE	Pik3r1
Stx3	145.6863	-2.031795188	0.387998	-5.23661	1.64E-07	1.60E-06	TRUE	Stx3
Camsap2	1353.209	-1.553085753	0.211532	-7.3421	2.10E-13	5.13E-12	TRUE	Camsap2
Sdk2	180.5639	-1.170570956	0.481921	-2.42897	0.015142	0.03944	TRUE	Sdk2
Tmtc4	283.7557	-1.435380517	0.333277	-4.30687	1.66E-05	0.000103	TRUE	Tmtc4
Mbp	284.214	-1.898623783	0.252782	-7.51093	5.87E-14	1.53E-12	TRUE	Mbp
Bicdl1	411.9948	-1.634606568	0.309371	-5.28365	1.27E-07	1.27E-06	TRUE	Bicdl1
Kctd12b	32.13624	-4.089631214	0.860562	-4.75228	2.01E-06	1.53E-05	TRUE	Kctd12b
Klf8	125.8779	-2.711702097	0.447117	-6.06485	1.32E-09	1.88E-08	TRUE	Klf8
Pcca	234.0562	-1.393129236	0.29269	-4.75975	1.94E-06	1.48E-05	TRUE	Pcca
Pnpla3	20.58994	-4.823969176	1.045398	-4.61448	3.94E-06	2.81E-05	TRUE	Pnpla3

Rims1	196.9619	-3.532137202	0.468296	-7.54254	4.61E-14	1.22E-12	TRUE	Rims1
Bivm	297.3863	-1.412564105	0.524925	-2.69098	0.007124	0.02089	TRUE	Bivm
Amot	224.2513	-1.124485005	0.382868	-2.93701	0.003314	0.010917	TRUE	Amot
Kcnj2	21.91086	-2.399860655	0.794312	-3.02131	0.002517	0.008647	TRUE	Kcnj2
Rasl12	42.61337	-1.369506775	0.455807	-3.00457	0.00266	0.009071	TRUE	Rasl12
Zic5	1635.347	-4.78464683	0.395895	-12.0856	1.26E-33	2.57E-31	TRUE	Zic5
Prrxl1	6.246267	-4.776840266	1.530956	-3.12017	0.001807	0.006462	TRUE	Prrxl1
Pgm5	6.315077	-4.800975872	1.540923	-3.11565	0.001835	0.006548	TRUE	Pgm5
Plekha6	2634.189	-1.455324994	0.245066	-5.93851	2.88E-09	3.89E-08	TRUE	Plekha6
Gpr155	68.72919	-2.311049202	0.472016	-4.89613	9.77E-07	8.04E-06	TRUE	Gpr155
Enc1	3700.545	-2.337461616	0.277421	-8.42568	3.59E-17	1.38E-15	TRUE	Enc1
Ydjc	75.93406	-1.780404928	0.388371	-4.58429	4.56E-06	3.19E-05	TRUE	Ydjc
700046A07F	58.88022	-2.519263029	0.934746	-2.69513	0.007036	0.020664	TRUE	2700046A07Rik
Fam169a	156.4972	-1.274093703	0.389099	-3.27447	0.001059	0.004048	TRUE	Fam169a
Fhdc1	37.78941	-1.602268718	0.468353	-3.42107	0.000624	0.002543	TRUE	Fhdc1
Ankrd13a	1012.799	-1.294043874	0.198223	-6.52821	6.66E-11	1.16E-09	TRUE	Ankrd13a
Dlx1	849.363	-2.212372849	0.362311	-6.10628	1.02E-09	1.49E-08	TRUE	Dlx1
Tdrkh	406.2075	-1.083401884	0.341694	-3.17068	0.001521	0.005574	TRUE	Tdrkh
Nol4	392.3477	-1.811410044	0.232225	-7.80025	6.18E-15	1.87E-13	TRUE	Nol4
Fam222a	84.4144	-3.175351656	0.446456	-7.11234	1.14E-12	2.56E-11	TRUE	Fam222a
Tnfrsf18	14.88139	-3.431109432	0.98846	-3.47117	0.000518	0.002157	TRUE	Tnfrsf18
Elmod1	77.9265	-1.440271837	0.573506	-2.51134	0.012027	0.03256	TRUE	Elmod1
Rapgef5	698.2603	-3.926041138	0.419749	-9.35331	8.49E-21	4.82E-19	TRUE	Rapgef5
Zbed3	388.6118	-1.461123199	0.494507	-2.95471	0.00313	0.0104	TRUE	Zbed3
Mat2b	277.1068	-1.055070681	0.424971	-2.48269	0.013039	0.034785	TRUE	Mat2b
Tbca	367.6861	-1.021767204	0.389658	-2.62221	0.008736	0.024882	TRUE	Tbca
Tmcc2	1087.592	-2.215823054	0.251189	-8.82136	1.13E-18	5.04E-17	TRUE	Tmcc2
Svop	730.1039	-1.779016037	0.371608	-4.78734	1.69E-06	1.31E-05	TRUE	Svop
Arsb	78.78126	-1.995089341	0.766319	-2.60347	0.009229	0.026053	TRUE	Arsb
Inpp5f	2021.737	-1.731103578	0.161384	-10.7266	7.63E-27	7.72E-25	TRUE	Inpp5f
Csdc2	456.3923	-1.704941309	0.231952	-7.35042	1.98E-13	4.84E-12	TRUE	Csdc2
Klhdc8a	91.99423	-2.495346243	0.377106	-6.6171	3.66E-11	6.68E-10	TRUE	Klhdc8a
Klhl23	671.1001	-1.378801389	0.306547	-4.49784	6.86E-06	4.63E-05	TRUE	Klhl23
Cmklr1	12.7121	-3.271895206	1.035521	-3.15966	0.00158	0.00575	TRUE	Cmklr1
510010F05F	863.8268	-1.619278235	0.216509	-7.47904	7.49E-14	1.93E-12	TRUE	0610010F05Rik
Sgsm1	470.1739	-2.453880716	0.333589	-7.356	1.90E-13	4.65E-12	TRUE	Sgsm1
Lyn	40.58123	-2.033901001	0.739805	-2.74924	0.005973	0.018078	TRUE	Lyn
Rabif	315.9194	-1.102726102	0.259015	-4.25739	2.07E-05	0.000125	TRUE	Rabif
Isl1	1019.173	-4.389775902	1.426403	-3.07751	0.002087	0.007346	TRUE	Isl1
Fam92b	7.540634	-5.050800644	1.490576	-3.38849	0.000703	0.002827	TRUE	Fam92b
Sestd1	1373.431	-1.134574779	0.241094	-4.70594	2.53E-06	1.88E-05	TRUE	Sestd1
Ehbp1	943.801	-1.012359415	0.23007	-4.40023	1.08E-05	6.97E-05	TRUE	Ehbp1
Arl15	257.116	-2.069535219	0.307329	-6.73395	1.65E-11	3.16E-10	TRUE	Arl15
Grap2	9.257225	-3.549003514	1.32058	-2.68746	0.0072	0.021079	TRUE	Grap2
Osbpl6	217.2012	-1.046135924	0.289869	-3.609	0.000307	0.001368	TRUE	Osbpl6
Lgalsl	292.1904	-1.257307071	0.289584	-4.34177	1.41E-05	8.89E-05	TRUE	Lgalsl
Snx18	200.6137	-1.166960942	0.449707	-2.59493	0.009461	0.026597	TRUE	Snx18
Rbm45	365.506	-1.131600481	0.388455	-2.91308	0.003579	0.011686	TRUE	Rbm45
Dlgap3	337.1619	-1.98760651	0.284422	-6.98822	2.78E-12	5.93E-11	TRUE	Dlgap3
Crtac1	46.51833	-1.517874056	0.499804	-3.03694	0.00239	0.008278	TRUE	Crtac1
Agps	426.4695	-1.00712083	0.403015	-2.49896	0.012456	0.033546	TRUE	Agps
Frmpd3	105.3033	-1.773375964	0.368765	-4.80896	1.52E-06	1.19E-05	TRUE	Frmpd3
Mgat3	776.3745	-2.323560471	0.250636	-9.27067	1.85E-20	1.02E-18	TRUE	Mgat3
Mindy2	347.4567	-1.658657294	0.268789	-6.17085	6.79E-10	1.03E-08	TRUE	Mindy2
Reln	423.5921	-2.513083421	0.444209	-5.65743	1.54E-08	1.85E-07	TRUE	Reln
Tfap2e	9.338559	-3.578801805	1.244443	-2.87583	0.00403	0.012914	TRUE	Tfap2e
Ago4	437.2674	-1.503489628	0.26935	-5.58192	2.38E-08	2.77E-07	TRUE	Ago4
Usp22	3552.377	-1.985513065	0.263145	-7.54531	4.51E-14	1.20E-12	TRUE	Usp22
Klhl14	11.83841	-5.705517423	1.364898	-4.18018	2.91E-05	0.000169	TRUE	Klhl14
Dnal1	315.4133	-1.126846207	0.374231	-3.0111	0.002603	0.0089	TRUE	Dnal1
Kcnj12	22.0538	-6.602135522	1.285728	-5.13494	2.82E-07	2.61E-06	TRUE	Kcnj12
Golga7b	173.2011	-3.390373917	0.625976	-5.41614	6.09E-08	6.53E-07	TRUE	Golga7b
Asb4	89.00569	-2.899744176	0.435872	-6.65275	2.88E-11	5.31E-10	TRUE	Asb4
Xkr7	99.02904	-3.003728764	0.473807	-6.33956	2.30E-10	3.68E-09	TRUE	Xkr7
Rgs8	864.2758	-3.648636274	0.38731	-9.42045	4.49E-21	2.60E-19	TRUE	Rgs8
Selenok	991.1051	-1.166882446	0.442273	-2.63838	0.00833	0.02388	TRUE	Selenok
Mapk6	2420.244	-2.16455289	0.372179	-5.81589	6.03E-09	7.81E-08	TRUE	Mapk6
Dhx9	1876.282	-1.217352617	0.350125	-3.47691	0.000507	0.002118	TRUE	Dhx9
Commd10	240.7237	-1.401377445	0.248092	-5.64863	1.62E-08	1.94E-07	TRUE	Commd10
Map3k9	211.2449	-1.673034649	0.313082	-5.34376	9.10E-08	9.39E-07	TRUE	Map3k9
Ttc9	272.5683	-1.392229863	0.311364	-4.4714	7.77E-06	5.17E-05	TRUE	Ttc9
Sgtb	360.7846	-2.287251347	0.263394	-8.68375	3.83E-18	1.61E-16	TRUE	Sgtb

Bex2	1384.421	-1.171989834	0.259571	-4.51511	6.33E-06	4.29E-05	TRUE	Bex2
Nmnat2	934.7236	-3.543378834	0.431677	-8.2084	2.24E-16	7.88E-15	TRUE	Nmnat2
Maneal	345.4139	-1.156683723	0.222167	-5.20636	1.93E-07	1.84E-06	TRUE	Maneal
Trim46	956.1297	-1.06541126	0.302134	-3.52629	0.000421	0.001797	TRUE	Trim46
Lgr6	3.703435	-4.028149349	1.656111	-2.43229	0.015004	0.039123	TRUE	Lgr6
Aqp11	50.23673	-1.366097119	0.564089	-2.42178	0.015445	0.040089	TRUE	Aqp11
Spata46	11.31998	-2.687317584	0.951429	-2.82451	0.004735	0.014802	TRUE	Spata46
Gpr153	364.9858	-3.11805731	0.588222	-5.30081	1.15E-07	1.17E-06	TRUE	Gpr153
Hecw2	93.90949	-2.596662387	0.405461	-6.40423	1.51E-10	2.50E-09	TRUE	Hecw2
Nrep	8013.118	-2.286244435	0.214905	-10.6384	1.98E-26	1.89E-24	TRUE	Nrep
Lhfpl4	2045.513	-2.131448237	0.209405	-10.1786	2.47E-24	2.00E-22	TRUE	Lhfpl4
Greb1l	61.4924	-3.177857473	0.517905	-6.13598	8.46E-10	1.25E-08	TRUE	Greb1l
Sbk1	9588.624	-1.888749781	0.273175	-6.91407	4.71E-12	9.71E-11	TRUE	Sbk1
Gng2	2436.583	-3.339548281	0.254107	-13.1423	1.88E-39	5.38E-37	TRUE	Gng2
Onecut1	4.707377	-4.378175911	1.685973	-2.59682	0.009409	0.026484	TRUE	Onecut1
Ptgir	4.027326	-4.139578664	1.728359	-2.39509	0.016616	0.042598	TRUE	Ptgir
Edem3	468.6635	-2.201112423	0.357849	-6.15096	7.70E-10	1.15E-08	TRUE	Edem3
Aqp6	4.933781	-4.435285723	1.601285	-2.76983	0.005609	0.017128	TRUE	Aqp6
Crppa	37.64308	-1.348529252	0.54055	-2.49474	0.012605	0.033808	TRUE	Crppa
Lor	27.05282	-3.659833206	0.897591	-4.07739	4.55E-05	0.000253	TRUE	Lor
Fam13c	143.3586	-2.349261501	0.3398	-6.91366	4.72E-12	9.73E-11	TRUE	Fam13c
B3galnt1	235.3716	-2.166755431	0.472278	-4.58788	4.48E-06	3.15E-05	TRUE	B3galnt1
Pcdhb19	35.43255	-1.781820867	0.578639	-3.07933	0.002075	0.007306	TRUE	Pcdhb19
Gprasp1	2897.639	-1.148775686	0.201414	-5.70355	1.17E-08	1.45E-07	TRUE	Gprasp1
Tmem130	1020.191	-4.039439791	1.436321	-2.81235	0.004918	0.015295	TRUE	Tmem130
510009E07R	791.4784	-1.588898382	0.23045	-6.89476	5.40E-12	1.10E-10	TRUE	2510009E07Rik
Rnf227	375.6085	-2.682009528	0.340218	-7.88321	3.19E-15	9.90E-14	TRUE	Rnf227
Gpr149	21.42181	-6.559413054	1.294842	-5.0658	4.07E-07	3.65E-06	TRUE	Gpr149
Rab9b	128.1451	-6.009144566	0.673765	-8.91875	4.72E-19	2.19E-17	TRUE	Rab9b
Zbtb37	304.3609	-1.06422449	0.414654	-2.56654	0.010272	0.028482	TRUE	Zbtb37
Tril	79.88353	-1.733535354	0.506806	-3.42051	0.000625	0.002548	TRUE	Tril
Sorcs1	147.5966	-1.636412051	0.502705	-3.25521	0.001133	0.004296	TRUE	Sorcs1
Zc2hc1a	595.1028	-1.646033855	0.250133	-6.58062	4.68E-11	8.34E-10	TRUE	Zc2hc1a
Pxylp1	595.4483	-1.029290137	0.287249	-3.58327	0.000339	0.001489	TRUE	Pxylp1
Ubxn10	16.05927	-2.952958684	1.033994	-2.85587	0.004292	0.013618	TRUE	Ubxn10
700019D03R	9.169995	-5.336183106	1.398442	-3.8158	0.000136	0.000665	TRUE	1700019D03Rik
Diras1	461.8099	-2.487372606	0.467684	-5.3185	1.05E-07	1.07E-06	TRUE	Diras1
Fem1a	758.8637	-1.15742089	0.196408	-5.89293	3.79E-09	5.03E-08	TRUE	Fem1a
Pde12	199.2848	-1.341532455	0.366911	-3.65629	0.000256	0.001166	TRUE	Pde12
Tmem145	775.5822	-1.534326903	0.281878	-5.44323	5.23E-08	5.69E-07	TRUE	Tmem145
Kbtbd7	269.3324	-1.530959296	0.457206	-3.34851	0.000812	0.003215	TRUE	Kbtbd7
Gm8399	25.74484	-2.71748981	0.971204	-2.79806	0.005141	0.015912	TRUE	Gm8399
S1pr2	65.95603	-1.377129726	0.431139	-3.19417	0.001402	0.005182	TRUE	S1pr2
Mgat2	218.8799	-1.058582674	0.338527	-3.12702	0.001766	0.006338	TRUE	Mgat2
Mrpl50	389.8113	-1.002675346	0.356862	-2.8097	0.004959	0.015408	TRUE	Mrpl50
Pcdhb21	12.75966	-3.424200875	1.359858	-2.51806	0.0118	0.032059	TRUE	Pcdhb21
Rell2	83.56723	-1.738579836	0.406509	-4.27685	1.90E-05	0.000116	TRUE	Rell2
Ccdc141	35.02167	-1.716632339	0.541478	-3.17027	0.001523	0.005578	TRUE	Ccdc141
Pcdhb14	10.27799	-2.9596406	1.097914	-2.69569	0.007024	0.02065	TRUE	Pcdhb14
Gpr22	45.64479	-4.119766059	0.673862	-6.11367	9.74E-10	1.43E-08	TRUE	Gpr22
Tafa2	54.83614	-3.456702359	0.659634	-5.24034	1.60E-07	1.57E-06	TRUE	Tafa2
Bmerb1	461.418	-2.063954815	0.224759	-9.18296	4.19E-20	2.24E-18	TRUE	Bmerb1
.30201H02R	9.761064	-3.757108215	1.172628	-3.20401	0.001355	0.005034	TRUE	E130201H02Rik
Nkrf	151.2629	-1.189578166	0.504168	-2.35949	0.0183	0.046127	TRUE	Nkrf
Rnf182	249.6504	-1.124685917	0.278176	-4.04307	5.28E-05	0.000287	TRUE	Rnf182
Spink10	26.37823	-3.49035094	0.739033	-4.72286	2.33E-06	1.75E-05	TRUE	Spink10
Pced1b	29.88826	-1.840065405	0.577272	-3.18752	0.001435	0.00529	TRUE	Pced1b
Osbpl1a	164.8727	-1.452294194	0.29455	-4.93055	8.20E-07	6.87E-06	TRUE	Osbpl1a
Gm1821	409.3971	-1.320665187	0.359724	-3.67133	0.000241	0.001106	TRUE	Gm1821
Cnr1	704.4193	-1.375094932	0.463039	-2.96972	0.002981	0.009995	TRUE	Cnr1
Slc25a53	133.8072	-1.165172133	0.280752	-4.15018	3.32E-05	0.00019	TRUE	Slc25a53
Snhg11	1128.939	-3.644915469	1.264016	-2.8836	0.003932	0.012648	TRUE	Snhg11
Ccdc89	11.19257	-5.62012763	1.367185	-4.11073	3.94E-05	0.000222	TRUE	Ccdc89
Tmem74b	73.79085	-1.233741813	0.513295	-2.40357	0.016236	0.041772	TRUE	Tmem74b
Cxxc4	716.1702	-3.310505165	0.304316	-10.8785	1.46E-27	1.55E-25	TRUE	Cxxc4
Slc16a13	186.0209	-1.688800707	0.323402	-5.22199	1.77E-07	1.71E-06	TRUE	Slc16a13
Sptssa	422.1865	-1.432836531	0.439081	-3.26326	0.001101	0.004192	TRUE	Sptssa
Camsap3	610.3035	-1.6028899	0.243064	-6.59452	4.27E-11	7.68E-10	TRUE	Camsap3
Gm9791	36.4233	-2.79354709	0.567771	-4.9202	8.65E-07	7.19E-06	TRUE	Gm9791
Dock5	40.86944	-1.798907584	0.706392	-2.54661	0.010877	0.029944	TRUE	Dock5
Zfand3	1388.201	-1.20371694	0.281569	-4.27504	1.91E-05	0.000117	TRUE	Zfand3
510039O18R	443.7478	-1.046609562	0.298694	-3.50396	0.000458	0.001937	TRUE	2510039O18Rik

Hs3st5	38.48919	-3.381170324	0.94892	-3.56318	0.000366	0.00159	TRUE	Hs3st5
Tram1l1	367.1953	-3.251393972	0.356959	-9.1086	8.35E-20	4.29E-18	TRUE	Tram1l1
Dact1	228.3652	-2.565784661	0.350208	-7.32647	2.36E-13	5.71E-12	TRUE	Dact1
Cage1	12.12238	-2.029208622	0.823088	-2.46536	0.013688	0.036277	TRUE	Cage1
Zbtb7c	70.96056	-3.144350141	0.653837	-4.80908	1.52E-06	1.19E-05	TRUE	Zbtb7c
Csrnp3	1593.948	-4.184121447	0.292577	-14.3009	2.16E-46	1.07E-43	TRUE	Csrnp3
Plppr4	27.25785	-1.791849828	0.604151	-2.9659	0.003018	0.0101	TRUE	Plppr4
Fzd1	528.8507	-1.283504523	0.352649	-3.63961	0.000273	0.001231	TRUE	Fzd1
Cnpy1	68.40027	-1.872021739	0.485027	-3.85962	0.000114	0.000567	TRUE	Cnpy1
Gm12231	15.65734	-1.88504242	0.766364	-2.45972	0.013904	0.03674	TRUE	Gm12231
Scml4	83.84361	-2.700823784	0.4678	-5.77346	7.77E-09	9.83E-08	TRUE	Scml4
Isca1	474.2057	-1.294645974	0.302489	-4.27998	1.87E-05	0.000114	TRUE	Isca1
Cyb5d1	122.3739	-1.66645148	0.463723	-3.59364	0.000326	0.001439	TRUE	Cyb5d1
Zfp354c	750.1107	-1.095724391	0.310633	-3.52739	0.00042	0.001791	TRUE	Zfp354c
Lsm11	533.4922	-1.250412764	0.376418	-3.32187	0.000894	0.003492	TRUE	Lsm11
Ankrd50	642.405	-1.040644716	0.229019	-4.54392	5.52E-06	3.80E-05	TRUE	Ankrd50
Coa4	60.01562	-1.056223494	0.452445	-2.33448	0.019571	0.048789	TRUE	Coa4
Syt16	162.9093	-2.32419189	0.413405	-5.62208	1.89E-08	2.23E-07	TRUE	Syt16
Ubtd2	201.7678	-1.534725171	0.460156	-3.33523	0.000852	0.003351	TRUE	Ubtd2
Tubg2	114.8731	-2.315703037	0.429306	-5.39406	6.89E-08	7.29E-07	TRUE	Tubg2
)030612E09R	60.84227	-2.753337788	0.475236	-5.79363	6.89E-09	8.79E-08	TRUE	9030612E09Rik
Ankrd34b	6.692253	-3.929161648	1.595094	-2.46328	0.013767	0.036475	TRUE	Ankrd34b
.130308A19R	590.3175	-1.110063061	0.20472	-5.42235	5.88E-08	6.33E-07	TRUE	E130308A19Rik
Lingo2	27.42501	-2.122379007	0.680383	-3.11939	0.001812	0.006475	TRUE	Lingo2
Ccdc73	42.8794	-1.563752224	0.669825	-2.33457	0.019566	0.048786	TRUE	Ccdc73
Tubb2b	17159.45	-1.769051272	0.201831	-8.765	1.87E-18	8.13E-17	TRUE	Tubb2b
Amer3	373.1805	-4.286821383	0.420785	-10.1877	2.25E-24	1.83E-22	TRUE	Amer3
Lrrc3b	9.987325	-2.858112306	1.132081	-2.52465	0.011581	0.031562	TRUE	Lrrc3b
Vcpip1	412.1669	-1.183276769	0.290304	-4.07599	4.58E-05	0.000254	TRUE	Vcpip1
Asxl3	605.9825	-2.185048363	0.261786	-8.3467	7.02E-17	2.60E-15	TRUE	Asxl3
Hs6st1	1162.618	-2.552410658	0.325864	-7.83275	4.77E-15	1.46E-13	TRUE	Hs6st1
Dcaf12l1	50.99353	-1.339992556	0.569978	-2.35095	0.018725	0.047005	TRUE	Dcaf12l1
Rtn4rl1	141.0301	-6.12164343	0.710715	-8.61336	7.10E-18	2.92E-16	TRUE	Rtn4rl1
Zfp423	1492.704	-2.983688014	0.268493	-11.1127	1.09E-28	1.37E-26	TRUE	Zfp423
Nyap1	2606.636	-2.205711199	0.221205	-9.97132	2.04E-23	1.47E-21	TRUE	Nyap1
Sh2d5	159.3747	-2.137428114	0.395074	-5.4102	6.30E-08	6.72E-07	TRUE	Sh2d5
Cxcr4	94.94432	-1.405888891	0.587474	-2.39311	0.016706	0.042789	TRUE	Cxcr4
Epcam	20.89466	-2.62438611	0.748588	-3.50578	0.000455	0.001925	TRUE	Epcam
Kcnk13	4.234535	-4.221005208	1.604985	-2.62993	0.00854	0.0244	TRUE	Kcnk13
Tmem60	97.10787	-1.193639874	0.452615	-2.63721	0.008359	0.023949	TRUE	Tmem60
Gprin3	4.599044	-4.343387092	1.634674	-2.65704	0.007883	0.022795	TRUE	Gprin3
Bhlhe23	7.146863	-4.96945997	1.598501	-3.10883	0.001878	0.006688	TRUE	Bhlhe23
Gpr150	5.085025	-4.478695498	1.618054	-2.76795	0.005641	0.017207	TRUE	Gpr150
Onecut3	12.57144	-5.792857595	1.554739	-3.72594	0.000195	0.000914	TRUE	Onecut3
Kcna5	7.193119	-4.988003036	1.489512	-3.34875	0.000812	0.003213	TRUE	Kcna5
Mtus1	750.3847	-1.476531774	0.186817	-7.90363	2.71E-15	8.48E-14	TRUE	Mtus1
Vwc2l	9.797026	-5.433633133	1.423121	-3.81811	0.000134	0.00066	TRUE	Vwc2l
Pid1	228.4466	-1.854799635	0.434507	-4.26874	1.97E-05	0.000119	TRUE	Pid1
Spred2	320.083	-1.048294294	0.379616	-2.76146	0.005754	0.017504	TRUE	Spred2
Pcdhb4	20.36382	-2.348606681	0.817782	-2.87192	0.00408	0.013036	TRUE	Pcdhb4
Thtpa	81.51377	-1.850112058	0.441504	-4.19047	2.78E-05	0.000163	TRUE	Thtpa
Pnoc	109.8222	-6.400265252	1.676323	-3.81804	0.000135	0.00066	TRUE	Pnoc
Basp1	5630.054	-1.516477016	0.235167	-6.44852	1.13E-10	1.89E-09	TRUE	Basp1
230219D22R	1063.981	-1.162175203	0.308365	-3.76883	0.000164	0.000786	TRUE	B230219D22Rik
Gm9800	2311.502	-1.049795475	0.400542	-2.62094	0.008769	0.024955	TRUE	Gm9800
Lyrm2	202.4151	-1.049773541	0.335156	-3.13219	0.001735	0.006239	TRUE	Lyrm2
Slitrk6	56.05241	-2.430702297	0.936224	-2.59628	0.009424	0.026511	TRUE	Slitrk6
Adra1a	4.663869	-4.361554515	1.579174	-2.76192	0.005746	0.017485	TRUE	Adra1a
Pcdhb8	20.70839	-3.294633661	0.788672	-4.17744	2.95E-05	0.000171	TRUE	Pcdhb8
Npas4	57.99502	-1.874977416	0.446798	-4.19648	2.71E-05	0.000159	TRUE	Npas4
Gpr158	31.82403	-3.951549354	0.747913	-5.28344	1.27E-07	1.27E-06	TRUE	Gpr158
Ing1	750.733	-1.056455181	0.275492	-3.83479	0.000126	0.000621	TRUE	Ing1
C2cd2	156.6802	-1.005832544	0.290433	-3.46322	0.000534	0.002212	TRUE	C2cd2
Onecut2	944.5446	-1.506350977	0.316624	-4.75753	1.96E-06	1.49E-05	TRUE	Onecut2
Snx12	605.9143	-1.506693177	0.40264	-3.74204	0.000183	0.000864	TRUE	Snx12
Lrrc8d	407.6512	-1.446314727	0.384104	-3.76542	0.000166	0.000795	TRUE	Lrrc8d
Hpcal4	38.52903	-4.439317325	0.990748	-4.48077	7.44E-06	4.97E-05	TRUE	Hpcal4
Tmem229b	535.7806	-1.981588037	0.231802	-8.54861	1.25E-17	5.00E-16	TRUE	Tmem229b
Olig1	42.40478	-6.585394156	1.179594	-5.58276	2.37E-08	2.76E-07	TRUE	Olig1
Nxph1	67.88528	-2.236976709	0.425516	-5.2571	1.46E-07	1.44E-06	TRUE	Nxph1
Gsg1l	44.01968	-1.708590401	0.700038	-2.44071	0.014658	0.038367	TRUE	Gsg1l
Zfp84	244.8847	-1.217322918	0.344823	-3.53028	0.000415	0.001773	TRUE	Zfp84

Pcdhb20	48.73388	-2.807051425	0.531463	-5.28174	1.28E-07	1.28E-06	TRUE	Pcdhb20
Pnma2	241.292	-3.185122189	0.284861	-11.1813	5.03E-29	6.72E-27	TRUE	Pnma2
Hepacam	3.829531	-4.068736696	1.740828	-2.33724	0.019427	0.048508	TRUE	Hepacam
Usp27x	150.1097	-2.186910392	0.304289	-7.18695	6.63E-13	1.52E-11	TRUE	Usp27x
Pnma3	134.0771	-2.33113105	0.479285	-4.86377	1.15E-06	9.31E-06	TRUE	Pnma3
Myorg	74.94667	-1.632735994	0.380742	-4.28829	1.80E-05	0.00011	TRUE	Myorg
Ccbe1	17.51108	-6.270941711	1.37712	-4.55366	5.27E-06	3.64E-05	TRUE	Ccbe1
Gm11223	194.1474	-2.720081832	0.417555	-6.5143	7.30E-11	1.26E-09	TRUE	Gm11223
Zfp322a	380.0367	-1.236252585	0.364214	-3.39431	0.000688	0.002776	TRUE	Zfp322a
Pcdhb17	77.9324	-3.26106385	0.455598	-7.15777	8.20E-13	1.87E-11	TRUE	Pcdhb17
Irx3os	7.849209	-3.437759028	1.329871	-2.58503	0.009737	0.027278	TRUE	Irx3os
Hnrnpa1	13479.03	-1.07833899	0.388926	-2.77261	0.005561	0.016998	TRUE	Hnrnpa1
Cmtr2	130.1162	-1.536553227	0.413638	-3.71473	0.000203	0.00095	TRUE	Cmtr2
Ppm1e	700.5931	-1.137485261	0.304245	-3.73871	0.000185	0.000874	TRUE	Ppm1e
Camk2n1	918.2575	-2.839460691	0.316143	-8.98158	2.67E-19	1.30E-17	TRUE	Camk2n1
Nexmif	266.2157	-3.004677729	0.544775	-5.51545	3.48E-08	3.90E-07	TRUE	Nexmif
30403N24F	96.2534	-1.365773972	0.504509	-2.70714	0.006787	0.02005	TRUE	5930403N24Rik
Sox18	38.25609	-2.170542361	0.678257	-3.20018	0.001373	0.005091	TRUE	Sox18
Golph3l	234.4014	-1.283498467	0.34562	-3.71361	0.000204	0.000953	TRUE	Golph3l
Fam43a	81.14772	-2.509290054	0.457242	-5.48788	4.07E-08	4.50E-07	TRUE	Fam43a
Zfp319	895.7058	-1.307005706	0.181733	-7.19191	6.39E-13	1.47E-11	TRUE	Zfp319
Zfp518b	704.0282	-1.407004072	0.284334	-4.94841	7.48E-07	6.33E-06	TRUE	Zfp518b
Cfap58	6.24002	-4.771906229	1.605424	-2.97237	0.002955	0.009927	TRUE	Cfap58
Bdh1	630.926	-1.48218927	0.402947	-3.67837	0.000235	0.00108	TRUE	Bdh1
Oacyl	4.309345	-4.246111295	1.615797	-2.62787	0.008592	0.024535	TRUE	Oacyl
Slitrk4	56.49096	-1.897979092	0.463006	-4.09925	4.14E-05	0.000232	TRUE	Slitrk4
Mapk10	985.5496	-3.613663672	0.486686	-7.42504	1.13E-13	2.87E-12	TRUE	Mapk10
Ttc6	24.43343	-5.101765645	1.05073	-4.85545	1.20E-06	9.66E-06	TRUE	Ttc6
Epm2aip1	904.5823	-1.619415206	0.35141	-4.60834	4.06E-06	2.88E-05	TRUE	Epm2aip1
Gpr61	6.640787	-3.046390263	1.266671	-2.40504	0.016171	0.041615	TRUE	Gpr61
Mpeg1	34.38183	-3.700192033	0.700349	-5.28336	1.27E-07	1.27E-06	TRUE	Mpeg1
Lrrc75b	498.5789	-2.160883092	0.473544	-4.56322	5.04E-06	3.50E-05	TRUE	Lrrc75b
Atp10d	44.7814	-1.753143752	0.491991	-3.56337	0.000366	0.00159	TRUE	Atp10d
Brox	961.7377	-1.094471129	0.21002	-5.21126	1.88E-07	1.81E-06	TRUE	Brox
Ckap4	917.6513	-1.677608264	0.29703	-5.64794	1.62E-08	1.94E-07	TRUE	Ckap4
Vat1l	506.6309	-4.01621154	0.283103	-14.1864	1.11E-45	5.04E-43	TRUE	Vat1l
Mbtps2	321.1826	-1.091154	0.30495	-3.57814	0.000346	0.001514	TRUE	Mbtps2
Atxn1	197.6729	-1.295449881	0.312401	-4.14676	3.37E-05	0.000193	TRUE	Atxn1
Myct1	6.474087	-3.152073691	1.317638	-2.39222	0.016747	0.04286	TRUE	Myct1
Spsb4	502.9276	-1.041028951	0.283244	-3.67537	0.000238	0.001091	TRUE	Spsb4
Fbxo41	286.0026	-2.073516897	0.427814	-4.84677	1.25E-06	1.01E-05	TRUE	Fbxo41
Nipa1	166.3574	-1.774131134	0.345722	-5.13167	2.87E-07	2.65E-06	TRUE	Nipa1
Lrrc4b	3413.025	-1.483695625	0.19498	-7.60949	2.75E-14	7.56E-13	TRUE	Lrrc4b
Cltc	1886.449	-1.598713321	0.305609	-5.23123	1.68E-07	1.64E-06	TRUE	Cltc
Cd24a	6833.467	-2.972847247	0.267763	-11.1025	1.22E-28	1.51E-26	TRUE	Cd24a
Cyp4x1	10.21739	-5.493027821	1.394728	-3.93842	8.20E-05	0.000424	TRUE	Cyp4x1
Helt	11.14963	-3.076568661	1.072547	-2.86847	0.004125	0.013163	TRUE	Helt
Rab2a	1572.559	-1.69364247	0.30255	-5.59788	2.17E-08	2.55E-07	TRUE	Rab2a
Dusp18	325.8628	-3.228944842	0.474489	-6.80509	1.01E-11	1.99E-10	TRUE	Dusp18
Mageh1	445.362	-1.696324529	0.335783	-5.05185	4.38E-07	3.91E-06	TRUE	Mageh1
Taf9b	73.35995	-2.13818515	0.425373	-5.02661	4.99E-07	4.41E-06	TRUE	Taf9b
Gap43	3377.986	-4.290107369	0.38473	-11.151	7.08E-29	9.11E-27	TRUE	Gap43
Pcdhb13	4.689115	-4.368406496	1.619823	-2.69684	0.007	0.020596	TRUE	Pcdhb13
Zfp768	402.9231	-1.063127611	0.283385	-3.75153	0.000176	0.000836	TRUE	Zfp768
Atmin	493.18	-1.220688838	0.225677	-5.409	6.34E-08	6.76E-07	TRUE	Atmin
Flrt2	156.3748	-1.454703205	0.455037	-3.19689	0.001389	0.005141	TRUE	Flrt2
Dlk2	73.30474	-1.230652659	0.493655	-2.49294	0.012669	0.033966	TRUE	Dlk2
Xxylt1	122.3753	-1.093335714	0.370945	-2.94743	0.003204	0.010596	TRUE	Xxylt1
Arl4a	723.2364	-4.861771079	0.279328	-17.4053	7.53E-68	1.12E-64	TRUE	Arl4a
30462N17F	241.3919	-1.067993619	0.280945	-3.80143	0.000144	0.000699	TRUE	8030462N17Rik
Klhl34	33.67721	-5.029160691	0.954406	-5.26941	1.37E-07	1.36E-06	TRUE	Klhl34
Dlgap2	21.39673	-2.053768221	0.648247	-3.16819	0.001534	0.005612	TRUE	Dlgap2
Rnf152	277.2511	-1.672090699	0.459819	-3.63641	0.000276	0.001245	TRUE	Rnf152
Mroh7	5.423747	-4.5754953	1.559706	-2.93356	0.003351	0.011023	TRUE	Mroh7
Baiap3	31.66106	-2.077270912	0.64187	-3.23628	0.001211	0.004561	TRUE	Baiap3
Tspyl1	795.3589	-2.057486624	0.29902	-6.88078	5.95E-12	1.21E-10	TRUE	Tspyl1
Cltb	329.4856	-1.340676043	0.345411	-3.8814	0.000104	0.000524	TRUE	Cltb
Lxn	52.63214	-2.374400235	0.495727	-4.78974	1.67E-06	1.29E-05	TRUE	Lxn
Gal3st3	153.0148	-2.41990716	0.409611	-5.90782	3.47E-09	4.63E-08	TRUE	Gal3st3
Yipf6	227.0335	-1.476967787	0.335143	-4.40697	1.05E-05	6.78E-05	TRUE	Yipf6
Ppp1r2	742.1695	-1.280906576	0.331169	-3.86784	0.00011	0.000551	TRUE	Ppp1r2
Rnf150	273.439	-2.219174865	0.339492	-6.53676	6.29E-11	1.09E-09	TRUE	Rnf150

Zc3hav1l	366.6571	-1.641356429	0.345066	-4.75664	1.97E-06	1.50E-05	TRUE	Zc3hav1l
Diras2	75.91857	-2.104675192	0.534238	-3.93958	8.16E-05	0.000423	TRUE	Diras2
Tshz2	477.4392	-2.538743324	0.579472	-4.38114	1.18E-05	7.53E-05	TRUE	Tshz2
Pcdhb16	17.80498	-3.767821865	0.958379	-3.93145	8.44E-05	0.000436	TRUE	Pcdhb16
Marcksl1	11904.83	-1.595347764	0.480004	-3.32362	0.000889	0.003474	TRUE	Marcksl1
Ino80c	180.1016	-1.807069114	0.500268	-3.6122	0.000304	0.001354	TRUE	Ino80c
Tmem196	21.85694	-4.001993587	0.880621	-4.54451	5.51E-06	3.79E-05	TRUE	Tmem196
Tmem229a	41.64965	-2.180632677	0.813445	-2.68074	0.007346	0.02142	TRUE	Tmem229a
Rgmb	1773.627	-2.982384303	0.225509	-13.2251	6.28E-40	1.84E-37	TRUE	Rgmb
Arxes2	284.3311	-2.047575153	0.384524	-5.32497	1.01E-07	1.03E-06	TRUE	Arxes2
Zbtb33	378.0809	-2.918826892	0.56158	-5.19752	2.02E-07	1.92E-06	TRUE	Zbtb33
Arf1	3005.732	-1.00639592	0.300156	-3.35291	0.0008	0.003169	TRUE	Arf1
Cracr2b	45.21659	-2.896120655	0.564823	-5.12748	2.94E-07	2.70E-06	TRUE	Cracr2b
Gpr85	456.0502	-3.301580828	0.375525	-8.7919	1.47E-18	6.44E-17	TRUE	Gpr85
Amigo2	62.61332	-1.644010206	0.548397	-2.99785	0.002719	0.009247	TRUE	Amigo2
Dip2c	437.6424	-1.225158755	0.247643	-4.94727	7.53E-07	6.36E-06	TRUE	Dip2c
Slitrk3	87.9843	-1.79962976	0.446847	-4.0274	5.64E-05	0.000305	TRUE	Slitrk3
Ankrd46	713.4203	-1.232495376	0.317255	-3.88488	0.000102	0.000518	TRUE	Ankrd46
Lhfp	80.11449	-2.083554667	0.477973	-4.35915	1.31E-05	8.27E-05	TRUE	Lhfp
Pou4f1	28.63692	-2.992836013	0.69067	-4.33324	1.47E-05	9.22E-05	TRUE	Pou4f1
Arxes1	172.7201	-2.068536833	0.57173	-3.61803	0.000297	0.001326	TRUE	Arxes1
Mlf1	41.24952	-2.26115706	0.564882	-4.00288	6.26E-05	0.000335	TRUE	Mlf1
Timm29	790.3196	-1.555430201	0.349267	-4.45341	8.45E-06	5.59E-05	TRUE	Timm29
Inka2	111.1631	-1.827939943	0.361954	-5.05019	4.41E-07	3.94E-06	TRUE	Inka2
Spata33	54.83193	-2.030705325	0.619792	-3.27643	0.001051	0.004024	TRUE	Spata33
Bdnf	12.95777	-3.677421105	1.05811	-3.47546	0.00051	0.002127	TRUE	Bdnf
Zdhhc22	26.61147	-3.402161527	1.0008	-3.39944	0.000675	0.002732	TRUE	Zdhhc22
Zbtb8b	41.88434	-2.870972877	0.777904	-3.69065	0.000224	0.001036	TRUE	Zbtb8b
Nrip1	296.282	-3.094794626	0.296486	-10.4383	1.66E-25	1.46E-23	TRUE	Nrip1
Nhlh2	1028.364	-3.670533336	0.265247	-13.8382	1.50E-43	5.79E-41	TRUE	Nhlh2
Ctxn1	1750.119	-1.464894477	0.298848	-4.9018	9.50E-07	7.84E-06	TRUE	Ctxn1
Mex3d	1084.32	-2.116818412	0.400374	-5.2871	1.24E-07	1.25E-06	TRUE	Mex3d
Speer4b	6.663255	-4.878406672	1.535683	-3.1767	0.00149	0.005471	TRUE	Speer4b
Tbc1d12	121.6205	-1.660159371	0.409609	-4.05304	5.06E-05	0.000276	TRUE	Tbc1d12
Fndc9	59.95981	-1.409660676	0.509648	-2.76595	0.005676	0.017303	TRUE	Fndc9
Klhl11	55.50195	-1.952654395	0.562101	-3.47385	0.000513	0.002138	TRUE	Klhl11
Dcun1d3	228.7226	-1.420841696	0.246991	-5.7526	8.79E-09	1.10E-07	TRUE	Dcun1d3
Cep120	917.851	-1.426132006	0.275145	-5.1832	2.18E-07	2.07E-06	TRUE	Cep120
Slc35e4	108.1151	-1.203867005	0.433955	-2.77418	0.005534	0.016928	TRUE	Slc35e4
Lonrf2	666.0469	-3.933830837	0.337294	-11.6629	1.97E-31	3.40E-29	TRUE	Lonrf2
Vstm2a	86.59239	-1.17133031	0.429739	-2.72568	0.006417	0.019173	TRUE	Vstm2a
Cdk5r1	4198.846	-1.724191318	0.281882	-6.1167	9.55E-10	1.40E-08	TRUE	Cdk5r1
Rnf24	428.9971	-3.051022115	0.407768	-7.48225	7.31E-14	1.88E-12	TRUE	Rnf24
Efna5	469.5335	-3.335285666	0.301302	-11.0696	1.76E-28	2.15E-26	TRUE	Efna5
Fkrp	447.6884	-1.28095095	0.246612	-5.19419	2.06E-07	1.96E-06	TRUE	Fkrp
Mrgpre	139.2343	-3.789948386	0.738925	-5.129	2.91E-07	2.68E-06	TRUE	Mrgpre
Nrsn1	155.2724	-4.275650767	0.452497	-9.44901	3.42E-21	2.00E-19	TRUE	Nrsn1
Elfn1	20.11615	-4.822819786	1.076648	-4.47948	7.48E-06	5.00E-05	TRUE	Elfn1
Ndnf	73.00141	-2.469129158	0.676747	-3.64853	0.000264	0.001196	TRUE	Ndnf
Armcx3	296.1715	-1.599117755	0.395316	-4.04516	5.23E-05	0.000285	TRUE	Armcx3
Zadh2	197.3258	-1.082512562	0.464122	-2.33239	0.01968	0.049013	TRUE	Zadh2
Gpr137c	382.1839	-1.273918228	0.282574	-4.50826	6.54E-06	4.42E-05	TRUE	Gpr137c
Dcaf5	996.3114	-1.516832158	0.191108	-7.93704	2.07E-15	6.58E-14	TRUE	Dcaf5
Frmd3	46.57967	-3.148795886	0.651039	-4.83657	1.32E-06	1.05E-05	TRUE	Frmd3
Catsperg2	3.628625	-3.998758288	1.682127	-2.3772	0.017444	0.044373	TRUE	Catsperg2
Nrap	44.40371	-5.760226258	1.061448	-5.42676	5.74E-08	6.20E-07	TRUE	Nrap
Plcxd3	37.23977	-3.511027119	0.64678	-5.42847	5.68E-08	6.14E-07	TRUE	Plcxd3
Zfp518a	165.323	-1.196029354	0.328573	-3.64007	0.000273	0.001229	TRUE	Zfp518a
Frmpd4	4.37192	-4.265242464	1.619258	-2.63407	0.008437	0.024141	TRUE	Frmpd4
Rtl5	1251.744	-1.810490878	0.239216	-7.56845	3.78E-14	1.02E-12	TRUE	Rtl5
Pdp1	513.6632	-1.44126176	0.349919	-4.11884	3.81E-05	0.000215	TRUE	Pdp1
Kcnk3	27.41428	-2.277700573	0.798336	-2.85306	0.00433	0.013714	TRUE	Kcnk3
Scn3b	613.2969	-3.5041254	0.326263	-10.7402	6.59E-27	6.80E-25	TRUE	Scn3b
Lix1l	1075.367	-1.102895652	0.343017	-3.21528	0.001303	0.004859	TRUE	Lix1l
Prmt6	199.467	-1.550418092	0.557687	-2.78009	0.005434	0.016665	TRUE	Prmt6
Smcr8	316.344	-1.291730206	0.335297	-3.8525	0.000117	0.000581	TRUE	Smcr8
Tmem200a	309.7216	-4.901512276	1.34614	-3.64116	0.000271	0.001225	TRUE	Tmem200a
Aff4	1993.745	-1.555780814	0.266143	-5.84565	5.05E-09	6.59E-08	TRUE	Aff4
Htr1b	6.597582	-4.863850922	1.542226	-3.15379	0.001612	0.00585	TRUE	Htr1b
Sall2	1310.894	-1.237737738	0.246332	-5.02468	5.04E-07	4.45E-06	TRUE	Sall2
Lingo1	225.7923	-3.931507619	0.677223	-5.80534	6.42E-09	8.29E-08	TRUE	Lingo1
Zfpm1	106.8481	-1.944227895	0.330076	-5.89025	3.86E-09	5.11E-08	TRUE	Zfpm1

Grm5	60.41794	-2.435448435	0.517856	-4.70295	2.56E-06	1.91E-05	TRUE	Grm5
Gpr3	7.157212	-3.310592386	1.368302	-2.41949	0.015542	0.040326	TRUE	Gpr3
Aftph	575.4781	-1.000418307	0.231638	-4.31889	1.57E-05	9.76E-05	TRUE	Aftph
Morn4	998.2521	-1.229112688	0.176791	-6.95236	3.59E-12	7.55E-11	TRUE	Morn4
Zbtb14	337.4255	-1.154588985	0.253776	-4.54964	5.37E-06	3.71E-05	TRUE	Zbtb14
Catsperg1	35.88294	-1.455826518	0.555426	-2.6211	0.008765	0.02495	TRUE	Catsperg1
Lig4	174.8565	-1.390166529	0.289505	-4.80188	1.57E-06	1.23E-05	TRUE	Lig4
Tmsb4x	12762.06	-1.256004074	0.269892	-4.65372	3.26E-06	2.37E-05	TRUE	Tmsb4x
Arhgap23	340.0808	-2.076592437	0.485533	-4.27693	1.89E-05	0.000116	TRUE	Arhgap23
Fam161a	41.01377	-2.944812349	0.543186	-5.42137	5.91E-08	6.35E-07	TRUE	Fam161a
Zbtb12	1180.143	-1.019030904	0.26735	-3.81159	0.000138	0.000675	TRUE	Zbtb12
Arl4c	963.8426	-3.38368707	0.247747	-13.6578	1.81E-42	6.63E-40	TRUE	Arl4c
Slc35c1	77.53032	-1.495597396	0.503932	-2.96786	0.002999	0.010041	TRUE	Slc35c1
Lrrc4	144.7133	-2.252086645	0.520694	-4.32517	1.52E-05	9.51E-05	TRUE	Lrrc4
Pgrmc2	476.3287	-1.081647938	0.309987	-3.48933	0.000484	0.00203	TRUE	Pgrmc2
BC030500	21.3067	-4.44520859	1.220916	-3.64088	0.000272	0.001226	TRUE	BC030500
Ankrd55	14.54437	-6.003077006	1.368033	-4.38811	1.14E-05	7.32E-05	TRUE	Ankrd55
Pitpnb	459.1242	-1.320144951	0.463653	-2.84727	0.00441	0.013921	TRUE	Pitpnb
Rap2c	547.725	-1.541070779	0.451706	-3.41166	0.000646	0.002623	TRUE	Rap2c
Zfp697	316.2562	-1.511122473	0.315011	-4.79704	1.61E-06	1.26E-05	TRUE	Zfp697
Grem2	13.7497	-5.919843896	1.336289	-4.43006	9.42E-06	6.15E-05	TRUE	Grem2
Sarm1	579.2315	-1.799652633	0.198834	-9.05103	1.42E-19	7.10E-18	TRUE	Sarm1
Slc25a44	346.9516	-1.028610289	0.270975	-3.79596	0.000147	0.000713	TRUE	Slc25a44
Ubqln2	1816.026	-2.529697649	0.462002	-5.47551	4.36E-08	4.82E-07	TRUE	Ubqln2
Mchr1	9.219154	-5.345448806	1.437685	-3.7181	0.000201	0.000939	TRUE	Mchr1
Eif5a2	101.2068	-1.749150469	0.65631	-2.66513	0.007696	0.022312	TRUE	Eif5a2
Lgr4	73.03087	-2.967610712	0.488248	-6.07808	1.22E-09	1.74E-08	TRUE	Lgr4
Dscam	309.4901	-1.074218201	0.408378	-2.63045	0.008527	0.024367	TRUE	Dscam
Nsun3	86.60099	-1.336589592	0.451884	-2.95781	0.003098	0.010314	TRUE	Nsun3
Synpo2	11.42043	-3.115161137	1.068316	-2.91596	0.003546	0.011597	TRUE	Synpo2
Neto1	37.21314	-3.4531674	0.618387	-5.58415	2.35E-08	2.74E-07	TRUE	Neto1
6-Sep	764.6306	-1.64870606	0.341511	-4.82768	1.38E-06	1.10E-05	TRUE	6-Sep
Fam171a1	675.232	-1.949024049	0.269486	-7.23237	4.75E-13	1.11E-11	TRUE	Fam171a1
Prokr2	822.5033	-6.976408036	1.532767	-4.55151	5.33E-06	3.68E-05	TRUE	Prokr2
Gpd1l	327.1281	-1.671502411	0.314128	-5.32109	1.03E-07	1.05E-06	TRUE	Gpd1l
Tmem150c	272.2672	-2.490752631	0.465579	-5.3498	8.81E-08	9.11E-07	TRUE	Tmem150c
Trhde	31.81994	-3.764725596	0.763052	-4.93377	8.07E-07	6.77E-06	TRUE	Trhde
Ccdc96	91.6595	-2.750317413	0.393428	-6.99064	2.74E-12	5.85E-11	TRUE	Ccdc96
Scg2	52.27654	-6.212666501	1.027972	-6.04361	1.51E-09	2.12E-08	TRUE	Scg2
Pgbd5	245.5206	-2.524525814	0.293942	-8.58852	8.81E-18	3.59E-16	TRUE	Pgbd5
B3galt6	207.1716	-1.335714186	0.374555	-3.56613	0.000362	0.001575	TRUE	B3galt6
Minar2	521.2529	-1.658197328	0.218533	-7.58786	3.25E-14	8.84E-13	TRUE	Minar2
Pdik1l	369.0139	-1.415000477	0.235102	-6.01867	1.76E-09	2.44E-08	TRUE	Pdik1l
Cdr2l	1167.554	-1.24317709	0.24918	-4.98908	6.07E-07	5.24E-06	TRUE	Cdr2l
Map10	68.26947	-1.247949284	0.374073	-3.33612	0.00085	0.003341	TRUE	Map10
Zfp169	122.423	-1.088718083	0.372216	-2.92496	0.003445	0.011304	TRUE	Zfp169
Kcns2	10.36003	-3.714955355	1.370565	-2.71053	0.006718	0.019877	TRUE	Kcns2
Prkca	166.092	-1.053201596	0.354658	-2.96962	0.002982	0.009995	TRUE	Prkca
Lin28a	22.388	-4.57349997	1.175433	-3.89091	9.99E-05	0.000507	TRUE	Lin28a
Creg2	11.93241	-4.711050598	1.371206	-3.4357	0.000591	0.002424	TRUE	Creg2
Fbll1	82.20577	-3.765329956	0.638821	-5.89419	3.77E-09	4.99E-08	TRUE	Fbll1
Mb21d2	261.0142	-3.53452245	0.319388	-11.0665	1.82E-28	2.21E-26	TRUE	Mb21d2
Rgs13	44.86973	-7.626926488	1.29861	-5.87315	4.28E-09	5.62E-08	TRUE	Rgs13
Sv2c	49.9414	-6.142760629	1.010578	-6.07846	1.21E-09	1.74E-08	TRUE	Sv2c
Camk2n2	207.8908	-1.425338965	0.292327	-4.87584	1.08E-06	8.83E-06	TRUE	Camk2n2
Bzw1	2694.445	-1.281174141	0.419147	-3.05662	0.002238	0.007821	TRUE	Bzw1
Swsap1	121.4278	-1.044070041	0.428938	-2.43408	0.01493	0.03896	TRUE	Swsap1
Pcdhb9	14.48005	-4.344396722	1.125288	-3.8607	0.000113	0.000565	TRUE	Pcdhb9
Islr2	3997.257	-3.599508712	0.296263	-12.1497	5.76E-34	1.21E-31	TRUE	Islr2
Taf7	216.0609	-1.239268027	0.34409	-3.60158	0.000316	0.001401	TRUE	Taf7
Gfod1	114.2312	-2.265540896	0.422999	-5.3559	8.51E-08	8.83E-07	TRUE	Gfod1
Plekhm3	148.1338	-1.525151851	0.296566	-5.14271	2.71E-07	2.52E-06	TRUE	Plekhm3
Zfp46	551.3884	-2.061097133	0.460917	-4.47174	7.76E-06	5.17E-05	TRUE	Zfp46
Samd3	147.6748	-9.34463073	1.226181	-7.62092	2.52E-14	6.94E-13	TRUE	Samd3
Ncald	410.8011	-3.343132563	0.361174	-9.2563	2.12E-20	1.16E-18	TRUE	Ncald
Plpp7	71.91365	-2.024811208	0.405034	-4.99912	5.76E-07	5.01E-06	TRUE	Plpp7
Pcdh1	493.3374	-3.507938813	0.319922	-10.965	5.63E-28	6.45E-26	TRUE	Pcdh1
Flrt3	501.4507	-2.290751809	0.314538	-7.28291	3.27E-13	7.82E-12	TRUE	Flrt3
Ywhag	6017.612	-2.748080977	0.366276	-7.50277	6.25E-14	1.62E-12	TRUE	Ywhag
Vamp7	207.7216	-1.621331371	0.545975	-2.96961	0.002982	0.009995	TRUE	Vamp7
Pcdhb11	11.89928	-3.07646614	0.964654	-3.18919	0.001427	0.005263	TRUE	Pcdhb11
Ufsp1	25.94138	-1.419333783	0.556203	-2.55183	0.010716	0.029548	TRUE	Ufsp1

Usp29	353.598	-1.372579828	0.372361	-3.68616	0.000228	0.001052	TRUE	Usp29
Gm5124	183.3667	-1.542973593	0.353725	-4.36207	1.29E-05	8.17E-05	TRUE	Gm5124
Pcdhb2	4.382766	-4.267431183	1.616532	-2.63987	0.008294	0.023795	TRUE	Pcdhb2
Rap2a	545.0886	-2.235969098	0.384251	-5.81904	5.92E-09	7.67E-08	TRUE	Rap2a
Lrrc3	80.87149	-2.366625646	0.559224	-4.23198	2.32E-05	0.000138	TRUE	Lrrc3
Dcun1d4	717.5485	-1.440612159	0.361955	-3.98009	6.89E-05	0.000365	TRUE	Dcun1d4
Trim32	615.47	-1.660494614	0.30129	-5.51129	3.56E-08	3.98E-07	TRUE	Trim32
Pcdhb6	9.508405	-2.83650937	1.121485	-2.52925	0.011431	0.031205	TRUE	Pcdhb6
Pcbp1	2229.272	-1.020528745	0.389945	-2.61711	0.008868	0.025199	TRUE	Pcbp1
Tmem198	413.627	-1.01036924	0.383485	-2.6347	0.008421	0.024106	TRUE	Tmem198
Kcnf1	155.3255	-6.334598818	0.68722	-9.21772	3.03E-20	1.63E-18	TRUE	Kcnf1
Rtl8c	327.2041	-3.409431026	0.305268	-11.1687	5.81E-29	7.56E-27	TRUE	Rtl8c
Arf3	1679.507	-1.792730166	0.262079	-6.84042	7.90E-12	1.58E-10	TRUE	Arf3
Mest	902.9519	-2.528774027	0.503584	-5.02155	5.13E-07	4.51E-06	TRUE	Mest
Xkr4	58.40664	-4.165137173	1.586909	-2.62469	0.008673	0.024736	TRUE	Xkr4
Btla	8.412909	-5.213224054	1.443751	-3.61089	0.000305	0.001359	TRUE	Btla
Slc6a7	40.00213	-6.44574844	1.303668	-4.94432	7.64E-07	6.45E-06	TRUE	Slc6a7
Dock8	3.715517	-4.032664527	1.654442	-2.43748	0.01479	0.038658	TRUE	Dock8
Mtcl1	1911.282	-1.085005606	0.241868	-4.48595	7.26E-06	4.86E-05	TRUE	Mtcl1
Acvr2a	512.2278	-1.907147256	0.305102	-6.25086	4.08E-10	6.36E-09	TRUE	Acvr2a
Gpr17	4.575673	-4.331216264	1.596139	-2.71356	0.006656	0.019738	TRUE	Gpr17
Epx	4.47213	-4.298840456	1.586145	-2.71024	0.006723	0.019889	TRUE	Epx
Cdc42se2	1755.362	-1.503464144	0.283784	-5.29791	1.17E-07	1.18E-06	TRUE	Cdc42se2
Tbc1d30	264.7705	-4.150833874	0.421226	-9.85418	6.57E-23	4.54E-21	TRUE	Tbc1d30
Slc39a1	287.9411	-1.04292969	0.391656	-2.66287	0.007748	0.022444	TRUE	Slc39a1
Ankrd44	224.3576	-1.656759874	0.450703	-3.67595	0.000237	0.001089	TRUE	Ankrd44
630019K06R	173.0125	-2.411597215	0.345854	-6.97288	3.11E-12	6.59E-11	TRUE	B630019K06Rik
Trpm3	66.61077	-3.233760371	0.703097	-4.59931	4.24E-06	2.99E-05	TRUE	Trpm3
Acot4	13.51104	-2.397442396	0.840107	-2.85373	0.004321	0.013693	TRUE	Acot4
Atp6v1a	969.5053	-1.012686774	0.208852	-4.84883	1.24E-06	9.97E-06	TRUE	Atp6v1a
Nav2	1934.138	-2.350717934	0.451407	-5.20754	1.91E-07	1.83E-06	TRUE	Nav2
Robo2	1417.131	-1.512505985	0.33267	-4.54657	5.45E-06	3.76E-05	TRUE	Robo2
Adarb2	26.1707	-6.849048555	1.292205	-5.30028	1.16E-07	1.17E-06	TRUE	Adarb2
Cpne8	19.97931	-3.80432263	0.949669	-4.00594	6.18E-05	0.000331	TRUE	Cpne8
Dlg2	288.2927	-2.732077977	0.548249	-4.98328	6.25E-07	5.39E-06	TRUE	Dlg2
Rap1b	680.2384	-1.92508706	0.470665	-4.09014	4.31E-05	0.000241	TRUE	Rap1b
Zfp608	770.9179	-1.212305232	0.265587	-4.56462	5.00E-06	3.48E-05	TRUE	Zfp608
Reep1	1404.369	-3.234029221	0.240064	-13.4715	2.30E-41	7.49E-39	TRUE	Reep1
Senp7	604.6248	-1.003984052	0.20357	-4.93189	8.14E-07	6.82E-06	TRUE	Senp7
Prkg1	40.10656	-2.610883528	0.828149	-3.15267	0.001618	0.005867	TRUE	Prkg1
Ctif	932.3036	-1.543363295	0.327398	-4.71403	2.43E-06	1.82E-05	TRUE	Ctif
Sv2b	103.4621	-4.014608178	0.579893	-6.92302	4.42E-12	9.19E-11	TRUE	Sv2b
Jam2	718.6028	-1.876179522	0.284763	-6.58857	4.44E-11	7.94E-10	TRUE	Jam2
Fank1	19.15143	-1.997400029	0.75895	-2.6318	0.008494	0.024293	TRUE	Fank1
Gsx1	14.53194	-5.99855722	1.336382	-4.48865	7.17E-06	4.80E-05	TRUE	Gsx1
Ptprt	89.67863	-3.050016295	0.636221	-4.79396	1.64E-06	1.27E-05	TRUE	Ptprt
Spag16	4.084693	-4.16523478	1.625007	-2.56321	0.010371	0.02871	TRUE	Spag16
Gpr21	54.78091	-2.195864177	0.470066	-4.6714	2.99E-06	2.20E-05	TRUE	Gpr21
Mllt11	1993.39	-2.871116429	0.214369	-13.3934	6.61E-41	2.09E-38	TRUE	Mllt11
Arhgap20	42.26316	-3.127999858	0.649827	-4.81359	1.48E-06	1.17E-05	TRUE	Arhgap20
Ndfip2	372.1948	-2.08557375	0.335966	-6.20769	5.38E-10	8.23E-09	TRUE	Ndfip2
Pom121	774.7369	-1.302524165	0.399912	-3.25703	0.001126	0.004273	TRUE	Pom121
Adamts19	92.80619	-2.122434438	0.730927	-2.90376	0.003687	0.011984	TRUE	Adamts19
Cstf2t	788.3328	-1.487158527	0.415827	-3.57639	0.000348	0.001521	TRUE	Cstf2t
930563E22R	35.5313	-1.405487905	0.558219	-2.51781	0.011809	0.032077	TRUE	4930563E22Rik
Tanc2	1192.583	-1.14307524	0.256418	-4.45785	8.28E-06	5.48E-05	TRUE	Tanc2
Tll1	91.51011	-4.407127944	1.435989	-3.06905	0.002147	0.007526	TRUE	Tll1
Mast1	847.7681	-2.042493852	0.425288	-4.80261	1.57E-06	1.23E-05	TRUE	Mast1
Dusp7	317.4743	-1.068276413	0.440772	-2.42365	0.015365	0.039907	TRUE	Dusp7
Sox14	15.44346	-3.792164405	1.324821	-2.8624	0.004204	0.013385	TRUE	Sox14
Lysmd1	89.78529	-1.064684193	0.406279	-2.62058	0.008778	0.024971	TRUE	Lysmd1
Ubxn7	1222.637	-1.438069327	0.290657	-4.94765	7.51E-07	6.35E-06	TRUE	Ubxn7
Ppfia2	436.4309	-1.629006047	0.332798	-4.89487	9.84E-07	8.08E-06	TRUE	Ppfia2
Mat2a	1953.5	-1.124695211	0.325335	-3.45704	0.000546	0.002259	TRUE	Mat2a
Shisa6	60.92652	-2.624072368	0.439839	-5.96598	2.43E-09	3.32E-08	TRUE	Shisa6
Stum	9.907609	-5.450100122	1.435839	-3.79576	0.000147	0.000714	TRUE	Stum
Pde5a	375.8896	-1.28040782	0.290965	-4.40055	1.08E-05	6.96E-05	TRUE	Pde5a
Gm9930	5.204579	-4.517047444	1.529459	-2.95336	0.003143	0.010438	TRUE	Gm9930
Tmem179	231.1471	-2.233493971	0.370812	-6.02326	1.71E-09	2.38E-08	TRUE	Tmem179
Nt5dc3	267.4368	-2.119610761	0.408235	-5.19214	2.08E-07	1.97E-06	TRUE	Nt5dc3
Tceal5	90.79686	-1.757948657	0.414532	-4.24081	2.23E-05	0.000133	TRUE	Tceal5
Spock3	78.00946	-5.896622784	1.674323	-3.5218	0.000429	0.001824	TRUE	Spock3

Cthrc1	14.74989	-1.950720488	0.778654	-2.50525	0.012237	0.033054	TRUE	Cthrc1
Lifr	107.8523	-1.218876964	0.373957	-3.25941	0.001116	0.004243	TRUE	Lifr
Eapp	423.5798	-1.005108563	0.233779	-4.2994	1.71E-05	0.000106	TRUE	Eapp
Rhob	1295.264	-2.120094515	0.240014	-8.83321	1.02E-18	4.56E-17	TRUE	Rhob
Pnma1	28.90905	-2.975806947	1.061074	-2.80452	0.005039	0.015627	TRUE	Pnma1
Spcs3	492.1073	-1.027516985	0.422908	-2.42965	0.015114	0.039376	TRUE	Spcs3
Vapb	1119.715	-1.036622232	0.234274	-4.42483	9.65E-06	6.28E-05	TRUE	Vapb
430021M05R	4.385681	-4.276200959	1.723068	-2.48174	0.013074	0.034865	TRUE	9430021M05Rik
Cggbp1	1013.291	-1.085382069	0.353853	-3.06732	0.00216	0.007565	TRUE	Cggbp1
Mettl7a1	88.29951	-1.070857298	0.351506	-3.04648	0.002315	0.008049	TRUE	Mettl7a1
Slc8a1	347.1645	-2.642781028	0.352573	-7.4957	6.59E-14	1.71E-12	TRUE	Slc8a1
Irs4	17.49592	-6.268632777	1.477361	-4.24313	2.20E-05	0.000132	TRUE	Irs4
600014C10R	128.0398	-1.206305106	0.344771	-3.49885	0.000467	0.00197	TRUE	1600014C10Rik
Srsf12	103.699	-2.663724193	0.418041	-6.37193	1.87E-10	3.03E-09	TRUE	Srsf12
Adam10	890.2993	-1.424211277	0.323203	-4.40655	1.05E-05	6.79E-05	TRUE	Adam10
Cadm4	3105.953	-1.345151084	0.268969	-5.00113	5.70E-07	4.96E-06	TRUE	Cadm4
Usp46	304.7115	-1.58222996	0.261648	-6.04718	1.47E-09	2.08E-08	TRUE	Usp46
Atrnl1	695.2525	-1.870511897	0.250294	-7.47326	7.82E-14	2.01E-12	TRUE	Atrnl1
Smim10l2a	65.67618	-1.310206775	0.401428	-3.26387	0.001099	0.004186	TRUE	Smim10l2a
Zfp667	192.1182	-2.51811329	0.400306	-6.29047	3.17E-10	5.00E-09	TRUE	Zfp667
Zfp174	35.68725	-1.461127559	0.474427	-3.07977	0.002072	0.007301	TRUE	Zfp174
Nyap2	96.48886	-2.275777452	0.40875	-5.56766	2.58E-08	2.98E-07	TRUE	Nyap2
Lrtm2	16.78623	-3.681464453	0.971314	-3.79019	0.000151	0.000728	TRUE	Lrtm2
Agap1	2182.007	-2.569701531	0.306025	-8.39702	4.58E-17	1.73E-15	TRUE	Agap1
Cntn1	135.5603	-6.363455256	0.761491	-8.35657	6.46E-17	2.40E-15	TRUE	Cntn1
Gabrg3	11.7881	-3.169022914	1.075041	-2.94782	0.0032	0.010585	TRUE	Gabrg3
Smyd3	89.42109	-1.770060663	0.41757	-4.23896	2.25E-05	0.000134	TRUE	Smyd3
Gabra5	8.921694	-3.513983903	1.193704	-2.94376	0.003242	0.010707	TRUE	Gabra5
Zfp78	40.15207	-1.108311811	0.457469	-2.4227	0.015406	0.040001	TRUE	Zfp78
Pld5	24.16474	-5.761341626	1.21091	-4.75786	1.96E-06	1.49E-05	TRUE	Pld5
430025C20R	7.869592	-3.445313518	1.257153	-2.74057	0.006133	0.018457	TRUE	9430025C20Rik
Sec23ip	474.2491	-1.013377397	0.34072	-2.97422	0.002937	0.009876	TRUE	Sec23ip
Stam2	337.6663	-1.26446708	0.288945	-4.37614	1.21E-05	7.70E-05	TRUE	Stam2
Map6	3191.13	-3.595760277	0.401909	-8.9467	3.66E-19	1.73E-17	TRUE	Map6
Nell1	10.64251	-5.547236581	1.372528	-4.04162	5.31E-05	0.000288	TRUE	Nell1
Atp10b	5.352589	-4.552576667	1.579927	-2.88151	0.003958	0.012715	TRUE	Atp10b
Nap1l5	324.9179	-3.437241709	0.423696	-8.11253	4.96E-16	1.68E-14	TRUE	Nap1l5
Zfp458	33.61821	-1.370855163	0.563073	-2.4346	0.014908	0.038914	TRUE	Zfp458
Epm2a	54.50298	-2.010277875	0.514283	-3.90889	9.27E-05	0.000474	TRUE	Epm2a
Epha6	5.563506	-4.617974269	1.572051	-2.93755	0.003308	0.010902	TRUE	Epha6
Unc80	58.10771	-4.270874402	0.604327	-7.06716	1.58E-12	3.49E-11	TRUE	Unc80
Slain1	364.8635	-1.456865851	0.269453	-5.40676	6.42E-08	6.83E-07	TRUE	Slain1
Rras2	242.0612	-1.726392596	0.398737	-4.32966	1.49E-05	9.34E-05	TRUE	Rras2
Paqr3	122.1928	-2.115233922	0.374547	-5.64745	1.63E-08	1.95E-07	TRUE	Paqr3
Nap1l3	80.87521	-3.469588845	0.495698	-6.9994	2.57E-12	5.53E-11	TRUE	Nap1l3
Rtl6	1227.221	-1.70026489	0.234941	-7.237	4.59E-13	1.08E-11	TRUE	Rtl6
Fmnl1	59.52622	-4.888745646	0.735361	-6.64809	2.97E-11	5.46E-10	TRUE	Fmnl1
Gm14137	12.80682	-4.865970116	1.267113	-3.8402	0.000123	0.000608	TRUE	Gm14137
Fto	1215.015	-1.071426668	0.194451	-5.51	3.59E-08	4.01E-07	TRUE	Fto
Prr18	256.5003	-2.793528057	0.281925	-9.90876	3.81E-23	2.70E-21	TRUE	Prr18
Triqk	47.3245	-2.92812899	0.528693	-5.53843	3.05E-08	3.47E-07	TRUE	Triqk
Cldn23	10.68196	-5.558001269	1.404485	-3.95732	7.58E-05	0.000397	TRUE	Cldn23
330182L06R	60.98896	-1.798328659	0.747697	-2.40516	0.016165	0.041612	TRUE	9330182L06Rik
Zfp709	94.31927	-1.629007107	0.389965	-4.17732	2.95E-05	0.000171	TRUE	Zfp709
Otulinl	23.05181	-1.469382903	0.58514	-2.51116	0.012033	0.032573	TRUE	Otulinl
Gm5468	13.62607	-2.437669756	0.842773	-2.89244	0.003823	0.012345	TRUE	Gm5468
Ticam2	7.516038	-3.243255819	1.242521	-2.61022	0.009048	0.025628	TRUE	Ticam2
Pgm3	110.6615	-1.057732675	0.42785	-2.47221	0.013428	0.03569	TRUE	Pgm3
Car10	45.75613	-6.663599809	1.204882	-5.5305	3.19E-08	3.61E-07	TRUE	Car10
Cfl1	7039.688	-1.333073132	0.371697	-3.58645	0.000335	0.001473	TRUE	Cfl1
Spock1	346.3677	-4.307057949	0.54108	-7.96011	1.72E-15	5.51E-14	TRUE	Spock1
Kcnq3	396.7944	-1.439045371	0.518255	-2.77671	0.005491	0.016817	TRUE	Kcnq3
Dennd1b	410.7279	-1.299592422	0.288843	-4.4993	6.82E-06	4.60E-05	TRUE	Dennd1b
Synpr	84.89283	-3.004634771	0.472237	-6.36256	1.98E-10	3.21E-09	TRUE	Synpr
Gm9996	9.91127	-2.597734555	1.109046	-2.34231	0.019165	0.047906	TRUE	Gm9996
Adap1	65.05466	-3.689733551	0.618181	-5.9687	2.39E-09	3.27E-08	TRUE	Adap1
BC043934	12.49012	-5.778461545	1.346378	-4.29186	1.77E-05	0.000109	TRUE	BC043934
Chn1	655.5482	-1.126499806	0.210452	-5.35275	8.66E-08	8.97E-07	TRUE	Chn1
Rab31	805.9493	-2.232495613	0.405624	-5.50386	3.72E-08	4.13E-07	TRUE	Rab31
Rlim	865.8887	-1.248267203	0.362015	-3.44811	0.000565	0.002326	TRUE	Rlim
Ptprn2	392.1949	-4.166057096	1.168181	-3.56628	0.000362	0.001574	TRUE	Ptprn2
Tug1	1983.054	-1.18028633	0.226594	-5.20882	1.90E-07	1.82E-06	TRUE	Tug1

Fry	455.1291	-2.53851023	0.343	-7.4009	1.35E-13	3.39E-12	TRUE	Fry
Nfil3	126.2756	-1.617682464	0.426234	-3.79529	0.000147	0.000715	TRUE	Nfil3
Dnah9	58.30831	-1.454797514	0.430583	-3.37867	0.000728	0.002915	TRUE	Dnah9
330011M18I	16.09071	-3.948531846	1.249871	-3.15915	0.001582	0.005756	TRUE	C330011M18Rik
Grm7	15.04803	-6.052803572	1.433889	-4.22125	2.43E-05	0.000144	TRUE	Grm7
St8sia3	296.3312	-3.401824458	0.317976	-10.6984	1.04E-26	1.03E-24	TRUE	St8sia3
Tsnax	1229.058	-1.280044646	0.209584	-6.10754	1.01E-09	1.48E-08	TRUE	Tsnax
Pou3f4	503.134	-1.553977124	0.401082	-3.87446	0.000107	0.000537	TRUE	Pou3f4
Hist3h2ba	1455.184	-1.000390162	0.238978	-4.18611	2.84E-05	0.000165	TRUE	Hist3h2ba
Gm5620	21.31937	-2.518456348	0.738339	-3.41098	0.000647	0.002629	TRUE	Gm5620
Mab21l1	35.44879	-5.565062675	1.27378	-4.36893	1.25E-05	7.94E-05	TRUE	Mab21l1
Magel2	603.5464	-2.544426505	0.594752	-4.27813	1.88E-05	0.000115	TRUE	Magel2
Skap1	12.14463	-2.4824074	0.870431	-2.85193	0.004345	0.01375	TRUE	Skap1
Ebf1	458.6623	-2.930213545	0.398122	-7.3601	1.84E-13	4.53E-12	TRUE	Ebf1
Ado	423.1317	-1.29076272	0.324097	-3.98265	6.82E-05	0.000361	TRUE	Ado
Scn3a	1041.624	-3.834337354	0.274722	-13.9571	2.85E-44	1.19E-41	TRUE	Scn3a
Cep170	2054.215	-2.022192079	0.257299	-7.85932	3.86E-15	1.19E-13	TRUE	Cep170
Ryr3	117.7909	-1.321516384	0.512396	-2.57909	0.009906	0.027662	TRUE	Ryr3
Fam136a	478.5843	-1.702740136	0.418336	-4.07027	4.70E-05	0.000259	TRUE	Fam136a
Eif1a	398.0241	-1.816572782	0.286206	-6.34707	2.19E-10	3.53E-09	TRUE	Eif1a
Gnai1	293.0987	-2.211751138	0.417155	-5.30198	1.15E-07	1.16E-06	TRUE	Gnai1
Mex3b	1712.679	-1.044675261	0.251878	-4.14755	3.36E-05	0.000192	TRUE	Mex3b
Tmem178b	779.6201	-2.481247653	0.301963	-8.21707	2.09E-16	7.35E-15	TRUE	Tmem178b
Mab21l2	24.04549	-6.727118677	1.589411	-4.23246	2.31E-05	0.000138	TRUE	Mab21l2
Xlr3a	32.29833	-1.809635434	0.625184	-2.89457	0.003797	0.012279	TRUE	Xlr3a
Abat	540.9518	-3.220844165	0.289318	-11.1325	8.71E-29	1.10E-26	TRUE	Abat
Camk2b	517.437	-2.776523849	0.575044	-4.82837	1.38E-06	1.09E-05	TRUE	Camk2b
Eml1	651.4194	-1.185355288	0.246861	-4.80171	1.57E-06	1.23E-05	TRUE	Eml1
Sdhc	711.452	-1.148911338	0.441514	-2.60221	0.009263	0.026128	TRUE	Sdhc
Tpm3-rs7	105.8735	-1.701985621	0.314965	-5.40372	6.53E-08	6.94E-07	TRUE	Tpm3-rs7
Chsy3	31.04786	-3.541254337	0.699255	-5.06432	4.10E-07	3.67E-06	TRUE	Chsy3
Sez6l	2145.489	-2.347942529	0.44348	-5.29436	1.19E-07	1.20E-06	TRUE	Sez6l
Gm5148	9.158676	-5.335730175	1.422594	-3.75071	0.000176	0.000838	TRUE	Gm5148
Arhgap35	2546.102	-1.664726391	0.243735	-6.83007	8.49E-12	1.69E-10	TRUE	Arhgap35
Kcnh1	18.02534	-4.680399487	1.217043	-3.84571	0.00012	0.000596	TRUE	Kcnh1
Idi1	216.4552	-1.330956528	0.518737	-2.56576	0.010295	0.028538	TRUE	Idi1
Spock2	1212.58	-3.168742872	0.406326	-7.79852	6.26E-15	1.89E-13	TRUE	Spock2
Syt17	27.73931	-2.488577768	0.62514	-3.98083	6.87E-05	0.000364	TRUE	Syt17
Znrf2	367.2578	-1.627375919	0.340904	-4.7737	1.81E-06	1.39E-05	TRUE	Znrf2
Rnf207	22.45018	-3.553499529	0.888383	-3.99996	6.34E-05	0.000338	TRUE	Rnf207
Anks1b	181.7237	-1.225092124	0.448132	-2.73377	0.006261	0.018775	TRUE	Anks1b
Samd12	64.12313	-5.103275496	0.828115	-6.16252	7.16E-10	1.08E-08	TRUE	Samd12
Nkx2-9	6.024837	-4.731095457	1.526763	-3.09878	0.001943	0.006894	TRUE	Nkx2-9
Tubb2a	2052.032	-3.10906616	0.221798	-14.0175	1.22E-44	5.29E-42	TRUE	Tubb2a
Ccser2	574.5378	-1.439990712	0.25432	-5.66211	1.50E-08	1.81E-07	TRUE	Ccser2
Zfp958	80.7141	-1.038879279	0.364807	-2.84775	0.004403	0.013903	TRUE	Zfp958
Thra	3160.24	-1.53058842	0.210302	-7.27804	3.39E-13	8.09E-12	TRUE	Thra
Abi1	488.477	-1.170968599	0.336613	-3.47868	0.000504	0.002105	TRUE	Abi1
Col25a1	86.4832	-2.625870841	0.662265	-3.96499	7.34E-05	0.000386	TRUE	Col25a1
Gm10052	536.2066	-1.175301483	0.29214	-4.02307	5.74E-05	0.00031	TRUE	Gm10052
Gm10053	20.06929	-1.698783127	0.702772	-2.41726	0.015638	0.040534	TRUE	Gm10053
Fam57b	1764.625	-1.267171694	0.428354	-2.95823	0.003094	0.010302	TRUE	Fam57b
Kcnc1	311.6089	-2.649440618	0.289652	-9.14697	5.86E-20	3.09E-18	TRUE	Kcnc1
Grin2a	22.53409	-4.992402129	1.084403	-4.60383	4.15E-06	2.94E-05	TRUE	Grin2a
Hnrnpa3	1752.095	-1.2690989	0.374291	-3.39068	0.000697	0.002808	TRUE	Hnrnpa3
Sh2d3c	213.6732	-2.01307283	0.255198	-7.88828	3.06E-15	9.52E-14	TRUE	Sh2d3c
Frem1	23.09642	-2.66274829	0.979312	-2.719	0.006548	0.019486	TRUE	Frem1
Tma7-ps	41.87492	-1.493903639	0.471169	-3.17064	0.001521	0.005574	TRUE	Tma7-ps
Pde1a	18.15378	-2.62468255	0.790004	-3.32237	0.000893	0.003488	TRUE	Pde1a
Tafa1	28.53428	-4.341555728	0.937516	-4.63091	3.64E-06	2.62E-05	TRUE	Tafa1
Gm12715	96.10319	-2.477883021	0.514482	-4.81627	1.46E-06	1.16E-05	TRUE	Gm12715
Foxb1	59.94884	-8.041705547	1.238379	-6.49374	8.37E-11	1.43E-09	TRUE	Foxb1
Nrsn2	76.79602	-2.802963616	0.829304	-3.3799	0.000725	0.002907	TRUE	Nrsn2
Arhgef12	1344.556	-1.104449466	0.213824	-5.16522	2.40E-07	2.25E-06	TRUE	Arhgef12
Syn3	44.86225	-1.996102512	0.553776	-3.60453	0.000313	0.001387	TRUE	Syn3
Rcan3	351.6807	-1.453183807	0.240173	-6.05058	1.44E-09	2.04E-08	TRUE	Rcan3
Ntng1	684.7784	-3.882857101	0.5959	-6.51596	7.22E-11	1.25E-09	TRUE	Ntng1
Unc5c	1472.519	-5.759427737	1.277168	-4.50953	6.50E-06	4.40E-05	TRUE	Unc5c
Grb2	801.8142	-1.132881289	0.223385	-5.07144	3.95E-07	3.55E-06	TRUE	Grb2
Nptx2	83.19694	-3.297600699	0.560564	-5.88265	4.04E-09	5.33E-08	TRUE	Nptx2
Pcnx2	136.217	-1.789670534	0.521232	-3.43354	0.000596	0.002443	TRUE	Pcnx2
Casc4	675.7806	-1.48628192	0.273445	-5.43539	5.47E-08	5.93E-07	TRUE	Casc4

Cend1	212.0919	-1.396170359	0.34516	-4.045	5.23E-05	0.000285	TRUE	Cend1
Nrg2	19.94612	-1.814414426	0.670422	-2.70638	0.006802	0.020088	TRUE	Nrg2
Zfp941	95.12426	-2.4859884	0.387902	-6.4088	1.47E-10	2.43E-09	TRUE	Zfp941
Zfp128	102.3481	-1.475741842	0.394519	-3.74061	0.000184	0.000868	TRUE	Zfp128
Rnf14	1008.741	-1.809352863	0.237984	-7.60284	2.90E-14	7.94E-13	TRUE	Rnf14
Samd5	567.1943	-2.948607776	0.573748	-5.1392	2.76E-07	2.56E-06	TRUE	Samd5
Zfp266	1409.178	-1.461593466	0.337983	-4.32446	1.53E-05	9.53E-05	TRUE	Zfp266
)30017M01I	19.64744	-1.967951226	0.708748	-2.77666	0.005492	0.016817	TRUE	A930017M01Rik
Fam78b	617.9658	-3.681517209	0.313018	-11.7613	6.17E-32	1.11E-29	TRUE	Fam78b
Fhit	7.655608	-2.889587313	1.172653	-2.46415	0.013734	0.036396	TRUE	Fhit
Slc9a6	579.7089	-1.593401726	0.299979	-5.31171	1.09E-07	1.10E-06	TRUE	Slc9a6
Ipmk	380.8428	-1.690805503	0.373404	-4.52809	5.95E-06	4.07E-05	TRUE	Ipmk
Rxfp3	7.663655	-5.078234521	1.50296	-3.37882	0.000728	0.002914	TRUE	Rxfp3
H3f3a	7115.5	-1.481037584	0.371811	-3.98331	6.80E-05	0.00036	TRUE	H3f3a
Tsga10	319.8962	-1.598546902	0.241778	-6.61163	3.80E-11	6.92E-10	TRUE	Tsga10
B9d1os	6.153418	-3.800741722	1.509396	-2.51806	0.0118	0.032059	TRUE	B9d1os
Kcnd2	69.96937	-1.902767884	0.462065	-4.11797	3.82E-05	0.000216	TRUE	Kcnd2
Tmem263	334.6594	-1.02702026	0.437356	-2.34825	0.018862	0.047263	TRUE	Tmem263
Irx1	38.31715	-7.397289451	1.242327	-5.95438	2.61E-09	3.55E-08	TRUE	Irx1
Galnt13	34.05293	-1.859238653	0.520126	-3.57459	0.000351	0.00153	TRUE	Galnt13
Gm11847	273.2953	-1.611774768	0.411663	-3.91528	9.03E-05	0.000463	TRUE	Gm11847
Lsamp	200.1631	-2.208070075	0.415428	-5.31516	1.07E-07	1.08E-06	TRUE	Lsamp
Gapdhs	31.97693	-2.215614898	0.574235	-3.85837	0.000114	0.00057	TRUE	Gapdhs
Ppm1b	868.7896	-1.339437397	0.25844	-5.18278	2.19E-07	2.07E-06	TRUE	Ppm1b
Fnip2	415.2872	-1.016628297	0.320678	-3.17025	0.001523	0.005578	TRUE	Fnip2
Mmgt1	546.4306	-1.235738548	0.419196	-2.94788	0.0032	0.010584	TRUE	Mmgt1
Rag1	12.86645	-5.826051016	1.393867	-4.17977	2.92E-05	0.000169	TRUE	Rag1
Nol4l	1331.223	-1.466540878	0.1826	-8.03145	9.63E-16	3.18E-14	TRUE	Nol4l
Tmem151a	23.4732	-1.628608172	0.698246	-2.33243	0.019678	0.049013	TRUE	Tmem151a
Zic2	2745.599	-4.518449017	0.431704	-10.4665	1.23E-25	1.10E-23	TRUE	Zic2
Dpp6	610.8938	-2.652657313	0.665448	-3.98627	6.71E-05	0.000357	TRUE	Dpp6
Ksr2	218.4637	-3.654110868	0.482055	-7.58028	3.45E-14	9.33E-13	TRUE	Ksr2
Akap6	635.5913	-2.719875809	0.309083	-8.79983	1.37E-18	6.02E-17	TRUE	Akap6
Spry3	35.40286	-2.861449779	0.670361	-4.26852	1.97E-05	0.000119	TRUE	Spry3
Gdpd1	971.2074	-1.119521113	0.20092	-5.57196	2.52E-08	2.91E-07	TRUE	Gdpd1
Tac1	7.336684	-5.017560193	1.554324	-3.22813	0.001246	0.004676	TRUE	Tac1
Gmfb	883.9046	-1.602468832	0.452106	-3.54445	0.000393	0.001691	TRUE	Gmfb
Iah1	165.7073	-1.217588692	0.393506	-3.09421	0.001973	0.006989	TRUE	Iah1
Gm6055	123.854	-1.875794397	0.547626	-3.42532	0.000614	0.002509	TRUE	Gm6055
Btbd3	477.7794	-1.812584483	0.405711	-4.46767	7.91E-06	5.25E-05	TRUE	Btbd3
Cttnbp2nl	838.6596	-1.824026503	0.26372	-6.91654	4.63E-12	9.55E-11	TRUE	Cttnbp2nl
Unc13c	9.808481	-5.434949896	1.451997	-3.74309	0.000182	0.000861	TRUE	Unc13c
Lancl2	536.0455	-2.770821173	0.475034	-5.83289	5.45E-09	7.09E-08	TRUE	Lancl2
Erbb4	273.0883	-3.029491093	0.530749	-5.70795	1.14E-08	1.41E-07	TRUE	Erbb4
Lhfpl5	21.56282	-1.540130406	0.608065	-2.53284	0.011314	0.030956	TRUE	Lhfpl5
Opcml	81.97028	-1.541878486	0.499848	-3.08469	0.002038	0.007191	TRUE	Opcml
Morf4l1	2494.887	-1.580308615	0.265922	-5.94274	2.80E-09	3.80E-08	TRUE	Morf4l1
Trank1	280.7212	-4.712492998	0.62767	-7.50791	6.01E-14	1.57E-12	TRUE	Trank1
Rpp25	51.57625	-2.236935079	0.458643	-4.87729	1.08E-06	8.78E-06	TRUE	Rpp25
Otof	12.91441	-2.205450886	0.87831	-2.51102	0.012038	0.032582	TRUE	Otof
Tmem65	492.0658	-1.981830538	0.290075	-6.83212	8.37E-12	1.67E-10	TRUE	Tmem65
Tubb3	15379.01	-2.901741622	0.164796	-17.608	2.14E-69	4.05E-66	TRUE	Tubb3
Dgkk	12.25594	-2.598623815	1.112304	-2.33625	0.019478	0.048608	TRUE	Dgkk
Zfp706	1624.982	-1.34966756	0.326494	-4.13382	3.57E-05	0.000203	TRUE	Zfp706
Arf2	1020.169	-1.878126304	0.343477	-5.46798	4.55E-08	5.01E-07	TRUE	Arf2
Ap3b2	1000.86	-1.419683998	0.425904	-3.33334	0.000858	0.003372	TRUE	Ap3b2
Fbxl12os	50.72629	-1.655977289	0.538476	-3.0753	0.002103	0.007395	TRUE	Fbxl12os
Tubb4a	1706.569	-3.58634967	0.279511	-12.8308	1.10E-37	2.94E-35	TRUE	Tubb4a
Ncs1	1808.039	-1.144438433	0.197986	-5.78041	7.45E-09	9.45E-08	TRUE	Ncs1
Sim2	12.77493	-3.464289344	1.3268	-2.61101	0.009027	0.025572	TRUE	Sim2
Shisal1	253.1319	-3.476780807	0.31827	-10.924	8.85E-28	9.76E-26	TRUE	Shisal1
Kcnc3	268.9183	-1.8025951	0.283353	-6.36166	2.00E-10	3.22E-09	TRUE	Kcnc3
Actg1	15738.66	-1.659639493	0.248463	-6.67963	2.40E-11	4.48E-10	TRUE	Actg1
Cfl2	1015.919	-1.205292362	0.473421	-2.54592	0.010899	0.029985	TRUE	Cfl2
Gm10123	70.93082	-1.39841467	0.404939	-3.45339	0.000554	0.002286	TRUE	Gm10123
.30023H24I	89.99178	-1.017685892	0.404999	-2.51281	0.011977	0.032455	TRUE	9130023H24Rik
Kdr	180.2488	-1.369215326	0.335666	-4.0791	4.52E-05	0.000251	TRUE	Kdr
Ccdc177	727.3324	-1.353788819	0.222321	-6.08934	1.13E-09	1.64E-08	TRUE	Ccdc177
Ccni	3024.324	-1.018996228	0.269547	-3.7804	0.000157	0.000754	TRUE	Ccni
Sox7	24.28459	-2.25310069	0.663462	-3.39597	0.000684	0.002761	TRUE	Sox7
Ctnna2	1143.973	-2.619448975	0.275893	-9.49443	2.21E-21	1.32E-19	TRUE	Ctnna2
Zfp26	456.7226	-1.147246587	0.324359	-3.53697	0.000405	0.001734	TRUE	Zfp26

Kcnma1	287.224	-4.338565427	1.495289	-2.90149	0.003714	0.012063	TRUE		Kcnma1
Clip2	2574.984	-1.166584839	0.242492	-4.81082	1.50E-06	1.18E-05	TRUE		Clip2
Grm4	221.5276	-3.313187266	1.390557	-2.38263	0.017189	0.04383	TRUE		Grm4
Hacd1	59.91793	-1.284291237	0.402179	-3.19333	0.001406	0.005196	TRUE		Hacd1
Luzp2	35.69543	-2.279133256	0.65471	-3.48113	0.000499	0.002087	TRUE		Luzp2
Usp31	376.2869	-1.891573149	0.398224	-4.75002	2.03E-06	1.55E-05	TRUE		Usp31
Mapk1	1470.13	-1.125455781	0.344857	-3.26354	0.0011	0.00419	TRUE		Mapk1
Alg11	239.5747	-1.723794986	0.459513	-3.75135	0.000176	0.000836	TRUE		Alg11
Stk24	276.5932	-1.109879587	0.348918	-3.18092	0.001468	0.0054	TRUE		Stk24
Wscd2	93.35178	-1.735685133	0.343402	-5.05438	4.32E-07	3.86E-06	TRUE		Wscd2
Sorcs3	123.5855	-6.567904605	0.7878	-8.33702	7.62E-17	2.82E-15	TRUE		Sorcs3
Plppr1	397.5329	-1.704258554	0.432868	-3.93713	8.25E-05	0.000427	TRUE		Plppr1
630045J12R	1190.1	-1.953122875	0.195085	-10.0117	1.35E-23	9.98E-22	TRUE		D630045J12Rik
Gng8	120.1816	-3.938703749	0.456183	-8.63404	5.92E-18	2.44E-16	TRUE		Gng8
Egfem1	65.51712	-4.935105931	0.701982	-7.03024	2.06E-12	4.48E-11	TRUE		Egfem1
Brwd3	337.2992	-1.176297583	0.242097	-4.85878	1.18E-06	9.52E-06	TRUE		Brwd3
Crb1	437.3634	-4.515789428	0.347364	-13.0002	1.22E-38	3.44E-36	TRUE		Crb1
Pcdhb5	34.31496	-2.005164997	0.560443	-3.57782	0.000346	0.001515	TRUE		Pcdhb5
Cycs	500.0857	-1.178563135	0.478903	-2.46096	0.013857	0.036646	TRUE		Cycs
Slc22a8	19.20583	-1.877247185	0.686077	-2.7362	0.006215	0.018661	TRUE		Slc22a8
Lin28b	305.7327	-4.238864026	0.552113	-7.67754	1.62E-14	4.59E-13	TRUE		Lin28b
Slc24a3	165.6575	-4.208775097	0.548448	-7.67397	1.67E-14	4.71E-13	TRUE		Slc24a3
Gm7964	491.3926	-1.411419547	0.484521	-2.91302	0.003579	0.011687	TRUE		Gm7964
Zar1	6.787101	-4.90176916	1.472993	-3.32776	0.000875	0.00343	TRUE		Zar1
Gm5089	4.79291	-4.394612823	1.608171	-2.73268	0.006282	0.018827	TRUE		Gm5089
Cnnm2	167.7969	-2.174164407	0.368092	-5.90657	3.49E-09	4.66E-08	TRUE		Cnnm2
Fam172a	621.4216	-1.586666354	0.340281	-4.66282	3.12E-06	2.28E-05	TRUE		Fam172a
Paqr9	26.24263	-1.827708489	0.727132	-2.51358	0.011951	0.0324	TRUE		Paqr9
Cntn4	24.43012	-6.751143274	1.348937	-5.00479	5.59E-07	4.88E-06	TRUE		Cntn4
Hhip	4.431857	-4.286270351	1.597741	-2.68271	0.007303	0.021318	TRUE		Hhip
Scn1a	165.016	-1.253139041	0.43173	-2.9026	0.003701	0.012024	TRUE		Scn1a
Selenop	69.90598	-2.341494656	0.798692	-2.93166	0.003372	0.011082	TRUE		Selenop
Mir135a-2	17.63227	-2.384241733	0.873425	-2.72976	0.006338	0.018978	TRUE		Mir135a-2
Mir153	10.59811	-4.380229384	1.399159	-3.13062	0.001744	0.00627	TRUE		Mir153
Kndc1	285.5616	-3.871582814	0.643931	-6.01242	1.83E-09	2.53E-08	TRUE		Kndc1
Slc31a1	422.0121	-1.046353514	0.411419	-2.54328	0.010982	0.030181	TRUE		Slc31a1
Slc31a2	70.12684	-1.662208882	0.420496	-3.95297	7.72E-05	0.000403	TRUE		Slc31a2
Cacng3	28.92554	-3.418784228	0.885901	-3.85911	0.000114	0.000568	TRUE		Cacng3
Gpr139	19.47976	-6.424084251	1.312032	-4.89629	9.77E-07	8.03E-06	TRUE		Gpr139
Wdr6	3706.485	-2.398561791	0.180472	-13.2905	2.63E-40	7.94E-38	TRUE		Wdr6
Nrxn3	699.5449	-2.529102248	0.30859	-8.19566	2.49E-16	8.71E-15	TRUE		Nrxn3
Gm16551	7.342365	-5.014855198	1.440058	-3.4824	0.000497	0.002079	TRUE		Gm16551
Gm10167	19.94064	-2.816315979	0.726867	-3.8746	0.000107	0.000537	TRUE		Gm10167
Lsm14a	930.8018	-1.09422587	0.405934	-2.69557	0.007027	0.020651	TRUE		Lsm14a
931406P16R	148.3888	-1.455624798	0.371192	-3.92149	8.80E-05	0.000452	TRUE		4931406P16Rik
Gm5113	212.1301	-1.401700419	0.265279	-5.28387	1.26E-07	1.27E-06	TRUE		Gm5113
Zbtb16	99.74207	-1.239867859	0.506455	-2.44813	0.01436	0.037713	TRUE		Zbtb16
Fxyd6	2300.436	-1.457318838	0.220922	-6.59654	4.21E-11	7.59E-10	TRUE		Fxyd6
Vkorc1l1	644.6401	-1.067468985	0.294367	-3.62632	0.000287	0.001289	TRUE		Vkorc1l1
Zbtb6	315.3417	-1.237352352	0.303951	-4.0709	4.68E-05	0.000259	TRUE		Zbtb6
Rnasel	1137.181	-1.162663139	0.176942	-6.57086	5.00E-11	8.89E-10	TRUE		Rnasel
Nck2	458.6242	-1.146609427	0.339275	-3.37959	0.000726	0.002909	TRUE		Nck2
Gm3608	66.97826	-1.314030701	0.416785	-3.15278	0.001617	0.005866	TRUE		Gm3608
Asb18	10.25694	-5.491309071	1.431263	-3.83669	0.000125	0.000617	TRUE		Asb18
Frat1	212.4128	-2.323502326	0.65307	-3.55781	0.000374	0.001619	TRUE		Frat1
Bmpr2	1062.759	-1.185465346	0.329933	-3.59305	0.000327	0.001442	TRUE		Bmpr2
Tspan6	504.355	-1.58336186	0.450707	-3.51306	0.000443	0.001881	TRUE		Tspan6
Zfp563	218.7723	-1.577094954	0.548678	-2.87436	0.004049	0.012954	TRUE		Zfp563
Zfp763	267.1507	-1.017651841	0.389265	-2.61429	0.008941	0.025348	TRUE		Zfp763
Gm7666	23.04311	-2.108436594	0.674714	-3.12493	0.001778	0.006372	TRUE		Gm7666
Cbln4	9.958256	-5.457494847	1.435531	-3.80172	0.000144	0.000699	TRUE		Cbln4
S1pr3	58.33213	-2.829735508	0.505141	-5.60187	2.12E-08	2.49E-07	TRUE		S1pr3
Nnat	26051.75	-1.999075678	0.226797	-8.81438	1.20E-18	5.32E-17	TRUE		Nnat
Blcap	1264.657	-1.677266155	0.190371	-8.81049	1.25E-18	5.49E-17	TRUE		Blcap
930444P10R	24.30408	-1.888552004	0.693092	-2.72482	0.006434	0.019211	TRUE		4930444P10Rik
Zic3	1221.203	-5.424941431	0.494607	-10.9682	5.44E-28	6.26E-26	TRUE		Zic3
Vxn	153.8713	-1.307739491	0.372103	-3.51446	0.000441	0.001872	TRUE		Vxn
Lrch1	98.96805	-1.20509854	0.418453	-2.87989	0.003978	0.01277	TRUE		Lrch1
Gm14164	8.372887	-5.204010497	1.414089	-3.68012	0.000233	0.001073	TRUE		Gm14164
500009C09R	414.5934	-2.005547558	0.30708	-6.53104	6.53E-11	1.14E-09	TRUE		1500009C09Rik
Insm1	1318.636	-1.226758516	0.470551	-2.60707	0.009132	0.025812	TRUE		Insm1
Macrod2	284.62	-1.736248167	0.322956	-5.37611	7.61E-08	7.97E-07	TRUE		Macrod2

Efcc1	32.38489	-4.300188482	0.814248	-5.28118	1.28E-07	1.28E-06	TRUE	Efcc1
430041D05R	370.9295	-1.818824895	0.339568	-5.35628	8.50E-08	8.82E-07	TRUE	D430041D05Rik
Myadm	122.8652	-2.510236989	0.378305	-6.63549	3.23E-11	5.92E-10	TRUE	Myadm
Gjd2	100.0332	-4.32996919	0.656905	-6.59147	4.35E-11	7.81E-10	TRUE	Gjd2
Efcab1	34.80896	-4.737741146	0.856966	-5.52851	3.23E-08	3.65E-07	TRUE	Efcab1
Gpr88	55.95444	-7.945467001	1.316159	-6.03686	1.57E-09	2.21E-08	TRUE	Gpr88
Tmem167b	435.1239	-2.173052242	0.493723	-4.40136	1.08E-05	6.94E-05	TRUE	Tmem167b
Trp53i11	4600.74	-2.026344556	0.185108	-10.9469	6.88E-28	7.68E-26	TRUE	Trp53i11
Gstm6	66.43254	-4.528797855	0.622617	-7.27381	3.49E-13	8.30E-12	TRUE	Gstm6
Csde1	4199.426	-1.466126104	0.223931	-6.54721	5.86E-11	1.02E-09	TRUE	Csde1
Sp9	1395.919	-5.277982724	1.021597	-5.1664	2.39E-07	2.24E-06	TRUE	Sp9
Zfp619	84.46533	-2.103706674	0.588065	-3.57734	0.000347	0.001517	TRUE	Zfp619
Gm5641	216.6909	-1.715221401	0.668787	-2.56467	0.010327	0.028604	TRUE	Gm5641
Slc7a14	38.52617	-4.653451545	0.856912	-5.43049	5.62E-08	6.08E-07	TRUE	Slc7a14
Zbtb10	420.107	-1.210908929	0.334599	-3.61898	0.000296	0.001322	TRUE	Zbtb10
Gprin1	1459.882	-2.998112233	0.248189	-12.08	1.35E-33	2.73E-31	TRUE	Gprin1
Fam8a1	1121.188	-1.576970567	0.292949	-5.38309	7.32E-08	7.72E-07	TRUE	Fam8a1
Dusp22	124.8822	-1.189963317	0.348252	-3.41696	0.000633	0.002577	TRUE	Dusp22
Epc2	758.7722	-1.042018485	0.220033	-4.73573	2.18E-06	1.65E-05	TRUE	Epc2
Tmem19	346.0023	-1.120237665	0.215363	-5.20162	1.98E-07	1.89E-06	TRUE	Tmem19
Scyl2	606.7453	-1.112548806	0.276099	-4.02953	5.59E-05	0.000302	TRUE	Scyl2
Marcks	10059.31	-1.074709337	0.354042	-3.03554	0.002401	0.008305	TRUE	Marcks
Ccdc92b	78.44007	-2.65676928	0.570852	-4.65404	3.25E-06	2.37E-05	TRUE	Ccdc92b
Insyn2b	61.56737	-4.171097186	0.689915	-6.04582	1.49E-09	2.09E-08	TRUE	Insyn2b
Mfhas1	368.5828	-1.643274896	0.275214	-5.97089	2.36E-09	3.22E-08	TRUE	Mfhas1
Scn2b	91.00206	-3.356578148	0.598009	-5.61292	1.99E-08	2.35E-07	TRUE	Scn2b
Ccnd1	542.6154	-1.952746384	0.427448	-4.56839	4.91E-06	3.42E-05	TRUE	Ccnd1
Plpp4	7.449075	-5.037084366	1.494752	-3.36985	0.000752	0.002999	TRUE	Plpp4
Capza1	914.359	-1.436910665	0.390966	-3.67528	0.000238	0.001091	TRUE	Capza1
Tlnrd1	443.5736	-2.235427088	0.331059	-6.75235	1.45E-11	2.80E-10	TRUE	Tlnrd1
Nsmce3	355.0661	-2.808140596	0.246822	-11.3772	5.43E-30	7.97E-28	TRUE	Nsmce3
Mkrn3	185.15	-1.583621185	0.267753	-5.91449	3.33E-09	4.46E-08	TRUE	Mkrn3
Rasal2	631.2786	-1.004641015	0.286924	-3.50142	0.000463	0.001953	TRUE	Rasal2
Fv1	37.16841	-1.643940721	0.600658	-2.7369	0.006202	0.018632	TRUE	Fv1
Vsig10l	36.18454	-1.582439418	0.541023	-2.9249	0.003446	0.011305	TRUE	Vsig10l
Lrrc8b	298.8196	-1.31155131	0.467701	-2.80425	0.005043	0.015635	TRUE	Lrrc8b
Etnk2	51.81702	-1.973556516	0.492434	-4.00776	6.13E-05	0.000329	TRUE	Etnk2
Cntnap5a	22.3512	-4.976596247	1.065681	-4.66988	3.01E-06	2.22E-05	TRUE	Cntnap5a
Eid2b	237.5436	-2.030899197	0.386782	-5.25076	1.51E-07	1.49E-06	TRUE	Eid2b
Pnmal2	2248.998	-4.402668906	0.504946	-8.71909	2.80E-18	1.20E-16	TRUE	Pnmal2
Zfp804a	27.05355	-3.15877467	0.750746	-4.20751	2.58E-05	0.000152	TRUE	Zfp804a
Ccnyl1	304.1006	-1.03959838	0.258827	-4.01658	5.90E-05	0.000318	TRUE	Ccnyl1
Gad1	294.3875	-2.455639252	0.334109	-7.34982	1.98E-13	4.86E-12	TRUE	Gad1
Klhl9	1024.638	-2.15140746	0.614729	-3.49977	0.000466	0.001963	TRUE	Klhl9
Rraga	601.5803	-2.295424905	0.398314	-5.76285	8.27E-09	1.04E-07	TRUE	Rraga
Ndufb6	145.7686	-1.154776577	0.495398	-2.33101	0.019753	0.049141	TRUE	Ndufb6
Slc25a16	105.0666	-1.607516408	0.691546	-2.32453	0.020097	0.049831	TRUE	Slc25a16
Bves	34.29356	-5.595310292	1.016907	-5.50228	3.75E-08	4.17E-07	TRUE	Bves
Tbpl1	800.9451	-1.461554319	0.287096	-5.09082	3.57E-07	3.22E-06	TRUE	Tbpl1
Grid2	11.93172	-3.082536896	0.966572	-3.18914	0.001427	0.005263	TRUE	Grid2
Nutf2-ps1	59.31326	-1.714201546	0.548234	-3.12677	0.001767	0.006341	TRUE	Nutf2-ps1
Gprin2	332.6994	-4.149290426	0.671594	-6.17827	6.48E-10	9.82E-09	TRUE	Gprin2
Akr1c19	6.485407	-3.147246038	1.270843	-2.4765	0.013268	0.035315	TRUE	Akr1c19
Fam189a2	21.03936	-2.876596008	0.852434	-3.37457	0.000739	0.002954	TRUE	Fam189a2
Rimbp3	48.83598	-2.694555331	0.572657	-4.70536	2.53E-06	1.89E-05	TRUE	Rimbp3
Gng3	920.4035	-2.520520778	0.249129	-10.1173	4.63E-24	3.67E-22	TRUE	Gng3
Foxr2	6.777202	-3.217061338	1.27989	-2.51355	0.011952	0.0324	TRUE	Foxr2
Tmem28	135.2636	-1.242362123	0.382953	-3.24416	0.001178	0.004453	TRUE	Tmem28
Apcdd1	946.6433	-1.98201125	0.309923	-6.39518	1.60E-10	2.64E-09	TRUE	Apcdd1
Lrrtm2	119.1443	-2.40804101	0.389351	-6.18475	6.22E-10	9.45E-09	TRUE	Lrrtm2
5-Sep	242.956	-1.441828115	0.354869	-4.06298	4.84E-05	0.000266	TRUE	5-Sep
Tuba1a	25522.08	-2.555377881	0.228718	-11.1726	5.55E-29	7.28E-27	TRUE	Tuba1a
Klf12	329.2622	-2.192675631	0.414163	-5.29424	1.20E-07	1.20E-06	TRUE	Klf12
Nanos1	256.9781	-1.822520178	0.323603	-5.63197	1.78E-08	2.12E-07	TRUE	Nanos1
Atp5l-ps1	7.458279	-2.784213885	1.151856	-2.41715	0.015642	0.040536	TRUE	Atp5l-ps1
Ppp1r3e	55.97508	-2.910833085	0.514753	-5.65482	1.56E-08	1.88E-07	TRUE	Ppp1r3e
Fzd10os	13.39833	-5.876689439	1.390893	-4.22512	2.39E-05	0.000142	TRUE	Fzd10os
Zfp9	132.4061	-1.616048412	0.639362	-2.5276	0.011485	0.031331	TRUE	Zfp9
Smim10l1	512.4935	-1.675570853	0.276809	-6.05316	1.42E-09	2.01E-08	TRUE	Smim10l1
Myo18b	63.37972	-8.122534856	1.228596	-6.61123	3.81E-11	6.93E-10	TRUE	Myo18b
Cep170b	1068.784	-1.874022652	0.199876	-9.37592	6.86E-21	3.94E-19	TRUE	Cep170b
Rybp	285.5467	-1.194982762	0.396436	-3.01431	0.002576	0.00882	TRUE	Rybp

Gpr27	270.1586	-1.96557842	0.385661	-5.09664	3.46E-07	3.13E-06	TRUE	Gpr27
Gm12258	186.6738	-1.594903728	0.325017	-4.90714	9.24E-07	7.65E-06	TRUE	Gm12258
Bhlhb9	466.6528	-1.930420981	0.353445	-5.46173	4.72E-08	5.17E-07	TRUE	Bhlhb9
Gprasp2	763.7748	-1.449244581	0.220854	-6.56199	5.31E-11	9.38E-10	TRUE	Gprasp2
Srp54a	30.98873	-1.416298668	0.591466	-2.39456	0.01664	0.042645	TRUE	Srp54a
Lrrc61	159.9116	-2.714562196	0.631049	-4.30166	1.70E-05	0.000105	TRUE	Lrrc61
Drc1	15.17822	-6.059069398	1.324095	-4.57601	4.74E-06	3.32E-05	TRUE	Drc1
Vma21	462.3129	-1.706973466	0.393052	-4.34287	1.41E-05	8.86E-05	TRUE	Vma21
Bnip3l-ps	51.36795	-1.419532448	0.554492	-2.56006	0.010465	0.028949	TRUE	Bnip3l-ps
Ccdc160	17.73421	-4.634683385	1.082448	-4.28167	1.85E-05	0.000113	TRUE	Ccdc160
Gm8773	9.828195	-2.89791661	1.129561	-2.56552	0.010302	0.028554	TRUE	Gm8773
Nudt10	222.7774	-2.111330922	0.468665	-4.50499	6.64E-06	4.48E-05	TRUE	Nudt10
Nudt11	242.5216	-2.74425202	0.482211	-5.69098	1.26E-08	1.54E-07	TRUE	Nudt11
Gm10509	19.50528	-2.102241453	0.7177	-2.92914	0.003399	0.011166	TRUE	Gm10509
H3f3aos	27.203	-1.509818197	0.536071	-2.81645	0.004856	0.015135	TRUE	H3f3aos
Dok6	79.61923	-1.984686516	0.514878	-3.85467	0.000116	0.000577	TRUE	Dok6
Rab18	703.6212	-1.748276746	0.436754	-4.00288	6.26E-05	0.000335	TRUE	Rab18
Pgap1	754.3879	-1.635847675	0.369101	-4.43197	9.34E-06	6.11E-05	TRUE	Pgap1
933415F23R	6.102605	-4.74480167	1.503088	-3.1567	0.001596	0.0058	TRUE	4933415F23Rik
730409E04R	103.7353	-3.730184821	0.418542	-8.91232	5.00E-19	2.32E-17	TRUE	5730409E04Rik
Gm10575	53.7895	-3.139055506	0.523714	-5.99384	2.05E-09	2.82E-08	TRUE	Gm10575
Ggh	61.74717	-1.151741498	0.446488	-2.57956	0.009893	0.027632	TRUE	Ggh
Exoc8	292.8229	-1.545453603	0.332149	-4.6529	3.27E-06	2.38E-05	TRUE	Exoc8
Rbm15b	2361.6	-1.210372708	0.294252	-4.11339	3.90E-05	0.00022	TRUE	Rbm15b
Gm10629	4.091414	-4.174096636	1.644089	-2.53885	0.011122	0.030501	TRUE	Gm10629
Dnajb14	296.1562	-1.031012288	0.361401	-2.85282	0.004333	0.01372	TRUE	Dnajb14
930028M14I	106.3212	-3.738607883	0.607842	-6.15062	7.72E-10	1.15E-08	TRUE	D930028M14Rik
S100a16	76.51878	-1.102561522	0.394716	-2.79331	0.005217	0.016122	TRUE	S100a16
Gm10699	35.73538	-2.420538839	0.630977	-3.83618	0.000125	0.000618	TRUE	Gm10699
Zfp558	168.8279	-1.852696507	0.339753	-5.45306	4.95E-08	5.41E-07	TRUE	Zfp558
Tox2	332.0093	-3.442915147	0.308072	-11.1757	5.36E-29	7.07E-27	TRUE	Tox2
Sox2	2338.635	-1.738436873	0.384151	-4.5254	6.03E-06	4.12E-05	TRUE	Sox2
Fam83c	13.51052	-2.855099548	1.045879	-2.72986	0.006336	0.018975	TRUE	Fam83c
BC029722	92.34517	-1.63949587	0.435843	-3.76166	0.000169	0.000805	TRUE	BC029722
Kif5a	2465.021	-1.667778269	0.347065	-4.80537	1.54E-06	1.21E-05	TRUE	Kif5a
Tspyl3	85.70397	-2.548252212	0.522733	-4.87486	1.09E-06	8.87E-06	TRUE	Tspyl3
Ccl28	17.8314	-2.721730018	0.942223	-2.88863	0.003869	0.01248	TRUE	Ccl28
Syndig1	37.43347	-2.644061728	0.76202	-3.46981	0.000521	0.002166	TRUE	Syndig1
Fndc10	84.58001	-1.219973453	0.352064	-3.4652	0.00053	0.0022	TRUE	Fndc10
Pdzd8	389.9209	-1.386258536	0.441651	-3.13881	0.001696	0.006119	TRUE	Pdzd8
Atxn7l3b	4457.2	-1.177966511	0.246323	-4.7822	1.73E-06	1.34E-05	TRUE	Atxn7l3b
Ube2n	1505.666	-1.281394931	0.51253	-2.50014	0.012415	0.033457	TRUE	Ube2n
Plxnc1	413.5695	-2.554696106	0.49716	-5.13858	2.77E-07	2.57E-06	TRUE	Plxnc1
Hspa12b	96.34705	-1.17909625	0.314726	-3.74642	0.000179	0.000851	TRUE	Hspa12b
Papolb	4.645442	-4.355232656	1.587926	-2.74272	0.006093	0.018366	TRUE	Papolb
Sptbn5	8.239849	-5.179732555	1.412105	-3.66809	0.000244	0.001119	TRUE	Sptbn5
Ranbp6	373.1866	-1.806001625	0.296129	-6.09869	1.07E-09	1.55E-08	TRUE	Ranbp6
Pak6	124.8939	-3.730228717	0.529916	-7.03928	1.93E-12	4.22E-11	TRUE	Pak6
Grem1	7.34252	-3.342712523	1.28399	-2.60338	0.009231	0.026056	TRUE	Grem1
Ano3	4.118689	-4.177880895	1.612763	-2.59051	0.009583	0.026905	TRUE	Ano3
Dcdc5	40.15991	-6.533754021	1.261421	-5.17968	2.22E-07	2.10E-06	TRUE	Dcdc5
Fjx1	969.898	-2.504214622	0.407037	-6.1523	7.64E-10	1.14E-08	TRUE	Fjx1
Nxpe3	129.5471	-1.686480232	0.291427	-5.78696	7.17E-09	9.12E-08	TRUE	Nxpe3
Vdac3-ps1	160.9846	-1.419708964	0.327983	-4.32861	1.50E-05	9.39E-05	TRUE	Vdac3-ps1
Yae1d1	298.7667	-1.056829514	0.424524	-2.48945	0.012794	0.034219	TRUE	Yae1d1
Lrrc55	61.33946	-2.035082915	0.608777	-3.3429	0.000829	0.003272	TRUE	Lrrc55
Ttc30b	39.67838	-1.760802564	0.75249	-2.33997	0.019285	0.048196	TRUE	Ttc30b
Sp5	9.396744	-5.371628322	1.395736	-3.8486	0.000119	0.00059	TRUE	Sp5
Gm13597	30.79246	-2.713965338	0.65751	-4.12764	3.67E-05	0.000208	TRUE	Gm13597
Scn2a	271.1858	-3.563078442	0.391139	-9.10949	8.28E-20	4.27E-18	TRUE	Scn2a
Fign	791.1294	-3.757715402	0.984785	-3.81577	0.000136	0.000665	TRUE	Fign
Zbtb2	239.1696	-1.230579209	0.391389	-3.14414	0.001666	0.00602	TRUE	Zbtb2
Gm10837	17.25912	-5.266049287	1.234253	-4.26659	1.98E-05	0.00012	TRUE	Gm10837
Alg10b	1078.141	-2.12217731	0.345392	-6.14426	8.03E-10	1.20E-08	TRUE	Alg10b
Slitrk1	129.7463	-2.24197377	0.360196	-6.22432	4.84E-10	7.45E-09	TRUE	Slitrk1
Zfp652	1016.862	-1.298759029	0.230603	-5.63201	1.78E-08	2.12E-07	TRUE	Zfp652
Selenot	764.6459	-1.861932305	0.543548	-3.42552	0.000614	0.002508	TRUE	Selenot
Selenoi	580.6053	-1.381244065	0.32431	-4.25902	2.05E-05	0.000124	TRUE	Selenoi
Ywhaq	3383.436	-1.498537766	0.318339	-4.70736	2.51E-06	1.87E-05	TRUE	Ywhaq
Ass1	7.173081	-4.980136421	1.44248	-3.45248	0.000555	0.002293	TRUE	Ass1
Snord91a	8.953959	-2.750241089	1.13382	-2.42564	0.015281	0.039728	TRUE	Snord91a
Erich3	22.55559	-3.429087696	0.825993	-4.15147	3.30E-05	0.000189	TRUE	Erich3

Chml	145.3063	-1.950746806	0.362803	-5.37688	7.58E-08	7.95E-07	TRUE	Chml
Foxd1	30.6246	-4.25492912	0.904952	-4.70183	2.58E-06	1.92E-05	TRUE	Foxd1
AI593442	70.15018	-5.693515031	1.711604	-3.32642	0.00088	0.003443	TRUE	AI593442
F8a	155.6543	-2.030622153	0.427623	-4.74863	2.05E-06	1.55E-05	TRUE	F8a
Nkain1	986.8414	-2.167548041	0.31585	-6.86259	6.76E-12	1.36E-10	TRUE	Nkain1
Bnip3	93.70991	-1.258678715	0.432282	-2.9117	0.003595	0.011727	TRUE	Bnip3
Ccdc47	538.8685	-1.089371266	0.379963	-2.86705	0.004143	0.013217	TRUE	Ccdc47
Psme3	956.2817	-1.078025809	0.396995	-2.71546	0.006618	0.019639	TRUE	Psme3
Fam174b	101.4984	-2.091156373	0.362196	-5.77355	7.76E-09	9.83E-08	TRUE	Fam174b
Tmem8b	668.8745	-1.476200365	0.270795	-5.45136	5.00E-08	5.46E-07	TRUE	Tmem8b
Dact3	580.8963	-1.387360483	0.299383	-4.63406	3.59E-06	2.59E-05	TRUE	Dact3
Hist3h2a	179.4715	-1.89670159	0.286335	-6.62407	3.49E-11	6.38E-10	TRUE	Hist3h2a
Wdr92	111.9419	-1.185026033	0.373626	-3.17169	0.001516	0.005557	TRUE	Wdr92
Col22a1	63.84538	-1.61439887	0.489269	-3.29961	0.000968	0.003747	TRUE	Col22a1
Prnp	556.0815	-2.067125821	0.228696	-9.03877	1.58E-19	7.86E-18	TRUE	Prnp
Prox1os	23.48505	-6.690160895	1.273865	-5.25186	1.51E-07	1.48E-06	TRUE	Prox1os
Slc8a3	69.50551	-1.800144291	0.470401	-3.82683	0.00013	0.00064	TRUE	Slc8a3
Jrkl	102.3951	-1.73011927	0.450382	-3.84145	0.000122	0.000606	TRUE	Jrkl
Srp54c	146.9184	-1.615246581	0.455326	-3.54745	0.000389	0.001675	TRUE	Srp54c
Capn3	28.21287	-3.847395749	0.803135	-4.79047	1.66E-06	1.29E-05	TRUE	Capn3
Fam155a	312.8272	-3.801468274	0.518788	-7.32759	2.34E-13	5.67E-12	TRUE	Fam155a
Gm6565	56.10636	-2.112668081	0.539841	-3.9135	9.10E-05	0.000466	TRUE	Gm6565
Ccdc13	24.97003	-1.537625805	0.6038	-2.54658	0.010878	0.029944	TRUE	Ccdc13
Trappc2	151.202	-1.667228945	0.521834	-3.19494	0.001399	0.00517	TRUE	Trappc2
Lemd1	161.8373	-2.451552543	0.413411	-5.93007	3.03E-09	4.08E-08	TRUE	Lemd1
Arpc4	641.6524	-1.304625322	0.341282	-3.82272	0.000132	0.000649	TRUE	Arpc4
Cldn34c1	47.81765	-1.708757678	0.530148	-3.22317	0.001268	0.004745	TRUE	Cldn34c1
Rab7	1374.329	-1.455091945	0.305204	-4.76761	1.86E-06	1.43E-05	TRUE	Rab7
Nhsl2	101.4795	-1.170810572	0.380204	-3.07943	0.002074	0.007306	TRUE	Nhsl2
Apoo	165.0527	-1.051660933	0.373553	-2.81529	0.004873	0.015183	TRUE	Apoo
Tmsb10	4290.616	-1.251607564	0.27439	-4.56143	5.08E-06	3.53E-05	TRUE	Tmsb10
Gm14597	126.5687	-1.892509862	0.39037	-4.84799	1.25E-06	1.00E-05	TRUE	Gm14597
Prrt4	27.61795	-2.448462848	0.640198	-3.82454	0.000131	0.000645	TRUE	Prrt4
Tmlhe	34.87089	-1.762038864	0.559251	-3.15071	0.001629	0.005899	TRUE	Tmlhe
Gm10698	74.73701	-1.722004994	0.444752	-3.87183	0.000108	0.000542	TRUE	Gm10698
Gm8107	6.104284	-4.750839031	1.533857	-3.09731	0.001953	0.006924	TRUE	Gm8107
Ywhaq-ps3	36.32503	-1.176970045	0.458892	-2.56481	0.010323	0.028601	TRUE	Ywhaq-ps3
Hist2h3c2	8.570977	-2.555856742	1.010392	-2.52957	0.01142	0.031188	TRUE	Hist2h3c2
Gm12430	15.46459	-2.271657506	0.973503	-2.33349	0.019623	0.048892	TRUE	Gm12430
Gm5940	5.942016	-3.744671716	1.465964	-2.55441	0.010637	0.029364	TRUE	Gm5940
Gm13509	480.462	-1.740852443	0.339274	-5.13112	2.88E-07	2.66E-06	TRUE	Gm13509
Gm12926	6.815654	-3.087938881	1.254714	-2.46107	0.013852	0.03664	TRUE	Gm12926
Fzd10	248.4395	-5.907043299	2.030123	-2.9097	0.003618	0.01179	TRUE	Fzd10
Gm12391	5.37981	-3.586469712	1.439839	-2.49088	0.012743	0.034103	TRUE	Gm12391
Hmgb1-ps2	257.1069	-1.282063257	0.537844	-2.38371	0.017139	0.043708	TRUE	Hmgb1-ps2
Gm13991	6.636485	-3.191881408	1.327728	-2.40402	0.016216	0.041726	TRUE	Gm13991
Gm15753	18.49672	-1.888438901	0.698932	-2.70189	0.006895	0.020332	TRUE	Gm15753
Nap1l2	241.4688	-5.477561837	0.658968	-8.31233	9.38E-17	3.43E-15	TRUE	Nap1l2
Gm12183	99.84477	-1.187810796	0.407885	-2.91212	0.00359	0.011715	TRUE	Gm12183
Gm13050	232.8372	-1.155552904	0.323067	-3.57683	0.000348	0.001519	TRUE	Gm13050
Dynlt1-ps1	297.1643	-1.304801306	0.35153	-3.71178	0.000206	0.00096	TRUE	Dynlt1-ps1
Gm13736	13.70503	-2.109854568	0.83674	-2.52152	0.011685	0.031795	TRUE	Gm13736
Gm14494	65.87321	-1.410370571	0.430339	-3.27735	0.001048	0.004012	TRUE	Gm14494
Gm7816	9.935359	-3.791491186	1.201676	-3.15517	0.001604	0.005829	TRUE	Gm7816
AA414768	19.3254	-1.68105411	0.718114	-2.34093	0.019236	0.048078	TRUE	AA414768
Gm8806	13.53251	-3.274794832	0.989234	-3.31043	0.000932	0.003619	TRUE	Gm8806
Gm4459	16.95177	-3.141430331	0.947873	-3.31419	0.000919	0.003576	TRUE	Gm4459
Gm14584	16.44678	-2.310541997	0.890383	-2.595	0.009459	0.026596	TRUE	Gm14584
Gm12131	4.805853	-4.395455577	1.636325	-2.68617	0.007228	0.02114	TRUE	Gm12131
Ldha-ps2	6.248573	-4.782442813	1.524972	-3.13609	0.001712	0.00617	TRUE	Ldha-ps2
Gm13416	8.7826	-3.489753942	1.204679	-2.89683	0.00377	0.012206	TRUE	Gm13416
Gm10241	21.86587	-1.629623817	0.659705	-2.47023	0.013503	0.03586	TRUE	Gm10241
933408N05F	10.33409	-2.966002906	1.111282	-2.66899	0.007608	0.022091	TRUE	4933408N05Rik
Gm16105	12.79044	-4.698645343	1.343038	-3.49852	0.000468	0.001972	TRUE	Gm16105
Ccdc85c	1330.077	-1.142807286	0.237713	-4.8075	1.53E-06	1.20E-05	TRUE	Ccdc85c
Dlx1as	25.05503	-2.256200207	0.685284	-3.29236	0.000994	0.003827	TRUE	Dlx1as
Gm11549	12.50464	-5.783943808	1.365957	-4.23435	2.29E-05	0.000137	TRUE	Gm11549
Gm15631	9.083546	-5.324968079	1.456557	-3.65586	0.000256	0.001167	TRUE	Gm15631
930095G15F	479.4951	-1.084015668	0.277167	-3.91105	9.19E-05	0.00047	TRUE	B930095G15Rik
Gm15663	86.47352	-3.165184162	0.628224	-5.03831	4.70E-07	4.17E-06	TRUE	Gm15663
833418N02F	23.61779	-1.718421493	0.614956	-2.79438	0.0052	0.016076	TRUE	4833418N02Rik
Gm15478	3.847473	-4.085384143	1.708371	-2.39139	0.016785	0.042946	TRUE	Gm15478

700095J03R	11.09975	-3.85115793	1.171487	-3.28741	0.001011	0.003887	TRUE	1700095J03Rik
Gm14344	4.609417	-4.347140747	1.63971	-2.65116	0.008021	0.023121	TRUE	Gm14344
610035F20R	277.8238	-2.992618914	0.445056	-6.72415	1.77E-11	3.37E-10	TRUE	2610035F20Rik
Gm11346	41.9111	-1.896744631	0.613351	-3.09243	0.001985	0.007024	TRUE	Gm11346
Gm9816	205.4612	-1.218090616	0.391954	-3.10774	0.001885	0.00671	TRUE	Gm9816
Ttc39aos1	12.77744	-5.809826613	1.366962	-4.25017	2.14E-05	0.000128	TRUE	Ttc39aos1
Gm15912	17.3752	-2.853433003	0.790789	-3.60834	0.000308	0.00137	TRUE	Gm15912
Gm14634	26.56472	-2.070229408	0.687233	-3.01241	0.002592	0.008868	TRUE	Gm14634
Rtl1	54.95201	-1.618433811	0.692746	-2.33626	0.019478	0.048608	TRUE	Rtl1
530051G07R	8.407831	-3.536638514	1.20499	-2.93499	0.003336	0.010985	TRUE	9530051G07Rik
Wipf3	146.0067	-2.94848844	0.385043	-7.65756	1.89E-14	5.31E-13	TRUE	Wipf3
Gm12473	9.134941	-2.292540917	0.967944	-2.36846	0.017862	0.045247	TRUE	Gm12473
Gm15728	19.40055	-1.752169243	0.7307	-2.39793	0.016488	0.042327	TRUE	Gm15728
Gm15829	8.017282	-3.46877692	1.228958	-2.82253	0.004765	0.014877	TRUE	Gm15829
Gm14342	80.58508	-5.00869632	0.675849	-7.41097	1.25E-13	3.16E-12	TRUE	Gm14342
Pcsk2os1	37.98438	-3.429642271	0.678206	-5.05694	4.26E-07	3.81E-06	TRUE	Pcsk2os1
Gm13571	4.279598	-4.23894815	1.632404	-2.59675	0.009411	0.026485	TRUE	Gm13571
330040F15R	4.602475	-4.336468539	1.59244	-2.72316	0.006466	0.019292	TRUE	A330040F15Rik
700069I18R	46.31079	-2.200053995	0.516848	-4.25668	2.07E-05	0.000125	TRUE	2700069I18Rik
030055H07R	314.1475	-5.308965397	0.483359	-10.9835	4.59E-28	5.31E-26	TRUE	D030055H07Rik
230369F24R	26.34769	-2.565523968	0.653349	-3.92673	8.61E-05	0.000444	TRUE	B230369F24Rik
Gm16172	22.65537	-1.616490166	0.631778	-2.55864	0.010508	0.029056	TRUE	Gm16172
Gm14204	291.9227	-3.220167003	0.363587	-8.85667	8.24E-19	3.73E-17	TRUE	Gm14204
Chn1os3	10.07202	-2.42227507	1.025859	-2.36122	0.018215	0.045934	TRUE	Chn1os3
Gm13595	5.418386	-4.578687002	1.566531	-2.92282	0.003469	0.011372	TRUE	Gm13595
Gm15577	21.73561	-6.576184081	1.334033	-4.92955	8.24E-07	6.90E-06	TRUE	Gm15577
Gm12828	64.3285	-8.146511734	1.25144	-6.50971	7.53E-11	1.30E-09	TRUE	Gm12828
Gm13716	6.416843	-4.823062855	1.53053	-3.15124	0.001626	0.005889	TRUE	Gm13716
Gm14697	4.031255	-4.147398819	1.618866	-2.56192	0.01041	0.028814	TRUE	Gm14697
Gm11611	25.72236	-3.164942049	0.86446	-3.66118	0.000251	0.001146	TRUE	Gm11611
Pcsk2os2	7.472643	-3.361217298	1.252595	-2.6834	0.007288	0.021283	TRUE	Pcsk2os2
330082K12R	355.9462	-1.221322213	0.31168	-3.91852	8.91E-05	0.000458	TRUE	A830082K12Rik
230056P14R	59.97728	-1.42958066	0.396448	-3.60598	0.000311	0.001381	TRUE	A230056P14Rik
Lhx1os	56.52187	-5.265706845	0.823325	-6.39566	1.60E-10	2.63E-09	TRUE	Lhx1os
Gm12827	13.8053	-5.925007896	1.346789	-4.39936	1.09E-05	6.99E-05	TRUE	Gm12827
Gm9392	16.46568	-2.14295862	0.773987	-2.76873	0.005628	0.017171	TRUE	Gm9392
230004M16R	15.32244	-2.795424044	0.892993	-3.1304	0.001746	0.006272	TRUE	A230004M16Rik
Prkag2os2	9.550116	-2.753131974	1.147366	-2.39952	0.016416	0.042174	TRUE	Prkag2os2
Gm14696	4.605322	-4.344222518	1.654151	-2.62626	0.008633	0.024639	TRUE	Gm14696
Tmem170b	528.3515	-1.473498295	0.250256	-5.88796	3.91E-09	5.17E-08	TRUE	Tmem170b
Gm16008	26.03629	-3.78349066	0.890249	-4.24992	2.14E-05	0.000129	TRUE	Gm16008
Cers1	135.2204	-1.262726889	0.296783	-4.25471	2.09E-05	0.000126	TRUE	Cers1
730405O15R	10.59397	-2.228233918	0.910735	-2.44663	0.01442	0.037814	TRUE	5730405O15Rik
Gm16054	5.591002	-4.62414086	1.538038	-3.00652	0.002643	0.009018	TRUE	Gm16054
Gm14286	45.92068	-1.224282202	0.489479	-2.5012	0.012377	0.033391	TRUE	Gm14286
230014O12R	9.625153	-4.397967513	1.303385	-3.37427	0.00074	0.002956	TRUE	C230014O12Rik
Gm13584	36.63252	-1.722067801	0.563713	-3.05487	0.002252	0.007857	TRUE	Gm13584
430040D24R	96.94638	-2.383712552	0.381061	-6.25545	3.96E-10	6.19E-09	TRUE	D430040D24Rik
Gm13414	8.475113	-3.4239963	1.234422	-2.77376	0.005541	0.016947	TRUE	Gm13414
500009L16R	112.3573	-1.184361346	0.331012	-3.578	0.000346	0.001514	TRUE	1500009L16Rik
930447M23R	19.54834	-3.466131683	0.853059	-4.06318	4.84E-05	0.000266	TRUE	4930447M23Rik
Gm22755	5.647705	-4.635287957	1.544198	-3.00175	0.002684	0.009144	TRUE	Gm22755
Gm25555	3.585492	-3.981448442	1.682856	-2.36589	0.017987	0.04547	TRUE	Gm25555
Gm23257	4.510916	-4.314952966	1.613114	-2.67492	0.007475	0.021744	TRUE	Gm23257
230216N24R	254.319	-4.700643116	0.471628	-9.96685	2.13E-23	1.53E-21	TRUE	B230216N24Rik
Gm15688	9.788209	-5.43127042	1.409568	-3.85314	0.000117	0.00058	TRUE	Gm15688
Slc5a3	254.3059	-2.939642058	0.477835	-6.152	7.65E-10	1.14E-08	TRUE	Slc5a3
Gm16536	395.7889	-1.061301522	0.23508	-4.51463	6.34E-06	4.30E-05	TRUE	Gm16536
Zfp882	163.617	-1.530519598	0.429657	-3.56219	0.000368	0.001595	TRUE	Zfp882
Umad1	112.0605	-1.926414016	0.316884	-6.07924	1.21E-09	1.73E-08	TRUE	Umad1
Etohd2	32.98695	-1.966441134	0.545655	-3.60381	0.000314	0.00139	TRUE	Etohd2
Gm15689	19.24677	-6.405313398	1.296402	-4.94084	7.78E-07	6.55E-06	TRUE	Gm15689
Pakap.2	4.103649	-4.178548811	1.782236	-2.34455	0.01905	0.047676	TRUE	Pakap.2
Nwd2	21.63966	-6.569692255	1.326535	-4.95252	7.33E-07	6.22E-06	TRUE	Nwd2
Dlx6os1	172.7065	-2.948874604	0.494886	-5.9587	2.54E-09	3.46E-08	TRUE	Dlx6os1
Cdk5r2	528.5706	-4.511575635	1.198218	-3.76524	0.000166	0.000796	TRUE	Cdk5r2
Ttbk2	726.8824	-2.158283784	0.27808	-7.76138	8.40E-15	2.48E-13	TRUE	Ttbk2
Kcne1l	92.99307	-1.555569355	0.592587	-2.62505	0.008664	0.024713	TRUE	Kcne1l
930480K15R	15.89027	-3.866777725	1.015722	-3.80693	0.000141	0.000686	TRUE	4930480K15Rik
BC035947	28.47682	-2.478148964	0.704023	-3.51998	0.000432	0.001836	TRUE	BC035947
Zfp712	44.65612	-1.976234455	0.551765	-3.58166	0.000341	0.001497	TRUE	Zfp712
Zfp493	21.04725	-1.894139265	0.721884	-2.62388	0.008693	0.024784	TRUE	Zfp493

Ccdc188	3.617779	-3.995997987	1.703039	-2.34639	0.018956	0.047464	TRUE	Ccdc188
Klhl33	6.219687	-4.775163689	1.480128	-3.22618	0.001255	0.004699	TRUE	Klhl33
Gm17112	21.88179	-2.17548685	0.686484	-3.16903	0.001529	0.0056	TRUE	Gm17112
Ccdc71l	182.793	-1.781639273	0.386099	-4.61446	3.94E-06	2.81E-05	TRUE	Ccdc71l
Tcerg1l	67.97121	-4.234751575	0.77922	-5.4346	5.49E-08	5.95E-07	TRUE	Tcerg1l
Smim13	316.5751	-1.029560984	0.257306	-4.0013	6.30E-05	0.000337	TRUE	Smim13
Gm6682	11.43158	-2.661061007	1.040049	-2.55859	0.01051	0.029056	TRUE	Gm6682
Eid1	1667.999	-2.092133988	0.370686	-5.64396	1.66E-08	1.98E-07	TRUE	Eid1
Gm4202	169.3763	-1.297343844	0.414402	-3.13064	0.001744	0.00627	TRUE	Gm4202
Gm17023	6.025698	-4.728562383	1.487132	-3.17965	0.001475	0.005422	TRUE	Gm17023
Mpc1-ps	65.1435	-1.659278244	0.482032	-3.44226	0.000577	0.002372	TRUE	Mpc1-ps
Gm3756	168.7754	-3.032292738	0.388978	-7.79554	6.41E-15	1.94E-13	TRUE	Gm3756
Sec14l5	87.74577	-5.147036119	0.735077	-7.00204	2.52E-12	5.44E-11	TRUE	Sec14l5
Zfp964	40.8234	-2.355053986	0.592612	-3.97402	7.07E-05	0.000373	TRUE	Zfp964
Cox16	30.84633	-1.770308065	0.720855	-2.45585	0.014055	0.037073	TRUE	Cox16
830073O21F	4.486432	-4.309112698	1.728968	-2.4923	0.012692	0.034015	TRUE	A830073O21Rik
Gm6395	26.94887	-2.213946214	0.653061	-3.39011	0.000699	0.002812	TRUE	Gm6395
Gm9844	70.49235	-1.470878484	0.447953	-3.28356	0.001025	0.003933	TRUE	Gm9844
130317F20R	131.4378	-1.759049552	0.483289	-3.63975	0.000273	0.00123	TRUE	E130317F20Rik
Peg10	937.6411	-1.904255024	0.39764	-4.78889	1.68E-06	1.30E-05	TRUE	Peg10
Bend4	177.1752	-1.800800286	0.325341	-5.53511	3.11E-08	3.53E-07	TRUE	Bend4
Dynlt1a	194.5132	-2.017786311	0.274712	-7.34511	2.06E-13	5.02E-12	TRUE	Dynlt1a
730060N03F	3.570397	-3.972698547	1.68922	-2.3518	0.018683	0.046933	TRUE	A730060N03Rik
130058E05F	102.4089	-8.814570256	1.222746	-7.20883	5.64E-13	1.31E-11	TRUE	D130058E05Rik
Gm18588	13.81138	-2.93275254	0.985488	-2.97594	0.002921	0.009834	TRUE	Gm18588
Six3os1	619.9159	-4.699620843	1.307425	-3.59456	0.000325	0.001435	TRUE	Six3os1
Tomm20	1234.46	-1.445177621	0.372304	-3.88171	0.000104	0.000524	TRUE	Tomm20
Hmgcs1	3026.481	-1.877447057	0.383347	-4.89752	9.71E-07	7.99E-06	TRUE	Hmgcs1
Gm8494	74.41496	-1.19356107	0.48901	-2.44077	0.014656	0.038366	TRUE	Gm8494
Zfp955a	178.0886	-1.006034155	0.35446	-2.83821	0.004537	0.014273	TRUE	Zfp955a
Smarce1-ps	83.95975	-1.191395378	0.369003	-3.22869	0.001244	0.00467	TRUE	Smarce1-ps1
Tmem121b	252.6665	-2.226097362	0.360939	-6.16752	6.94E-10	1.05E-08	TRUE	Tmem121b
Gm14403	3.853818	-4.088702821	1.689935	-2.41944	0.015544	0.040327	TRUE	Gm14403
430019H16F	3355.935	-2.338310802	0.20664	-11.3159	1.10E-29	1.56E-27	TRUE	D430019H16Rik
Edaradd	23.84201	-4.549976342	0.992774	-4.58309	4.58E-06	3.21E-05	TRUE	Edaradd
Pou3f2	2460.863	-1.639133237	0.212527	-7.71259	1.23E-14	3.55E-13	TRUE	Pou3f2
Gm21092	27.77275	-2.087965763	0.669348	-3.1194	0.001812	0.006475	TRUE	Gm21092
Tmem200c	51.81812	-6.196516257	1.009906	-6.13574	8.48E-10	1.25E-08	TRUE	Tmem200c
Zfp748	176.9999	-1.280393466	0.282727	-4.52872	5.93E-06	4.06E-05	TRUE	Zfp748
Fignl2	62.46051	-4.758878414	0.732553	-6.49629	8.23E-11	1.41E-09	TRUE	Fignl2
Gm9824	105.2107	-1.562574735	0.590595	-2.64576	0.008151	0.023435	TRUE	Gm9824
Gm8281	94.44904	-1.408011266	0.340192	-4.13887	3.49E-05	0.000199	TRUE	Gm8281
Rab11b-ps2	118.1529	-1.789664009	0.599868	-2.98343	0.00285	0.009624	TRUE	Rab11b-ps2
Syne1	118.5402	-3.796556135	0.515961	-7.35822	1.86E-13	4.59E-12	TRUE	Syne1
Dynlt1b	258.0745	-1.351074805	0.231918	-5.82566	5.69E-09	7.39E-08	TRUE	Dynlt1b
Gm4617	123.7892	-1.181288365	0.423323	-2.79052	0.005262	0.016228	TRUE	Gm4617
Tmem151b	815.5012	-1.55282584	0.30277	-5.12873	2.92E-07	2.69E-06	TRUE	Tmem151b
Galntl6	16.77409	-4.567405933	1.157237	-3.94682	7.92E-05	0.000412	TRUE	Galntl6
Gm26872	13.97121	-4.837386945	1.343766	-3.59987	0.000318	0.001408	TRUE	Gm26872
Gm26743	20.88737	-1.908112499	0.74151	-2.57328	0.010074	0.028007	TRUE	Gm26743
Gm26724	35.66372	-2.111520979	0.571083	-3.6974	0.000218	0.00101	TRUE	Gm26724
Gm26725	7.019464	-4.944153378	1.517782	-3.25749	0.001124	0.004268	TRUE	Gm26725
310010J17R	11.07486	-2.758637903	1.058226	-2.60685	0.009138	0.025822	TRUE	2310010J17Rik
Gm2694	86.97869	-4.471306544	0.632261	-7.07194	1.53E-12	3.38E-11	TRUE	Gm2694
700109K24F	22.88718	-2.098580035	0.772782	-2.71562	0.006615	0.019636	TRUE	1700109K24Rik
Gm26648	13.41569	-2.58869708	1.04843	-2.46912	0.013545	0.035949	TRUE	Gm26648
130017N08F	172.6329	-1.406452354	0.320934	-4.38237	1.17E-05	7.49E-05	TRUE	D130017N08Rik
Gm26871	255.1246	-3.644222902	0.643976	-5.65894	1.52E-08	1.84E-07	TRUE	Gm26871
Gm10516	20.72849	-1.621531296	0.661971	-2.44955	0.014304	0.037594	TRUE	Gm10516
Zfp87	158.8998	-1.133767344	0.432074	-2.62401	0.00869	0.024781	TRUE	Zfp87
500026H17F	6.69835	-3.051191271	1.284314	-2.37574	0.017514	0.044511	TRUE	1500026H17Rik
Gm26744	12.40455	-4.111281831	1.126944	-3.64817	0.000264	0.001198	TRUE	Gm26744
830052D11F	7.706112	-5.08483569	1.461671	-3.47878	0.000504	0.002105	TRUE	A830052D11Rik
Gm26782	55.85498	-1.268737761	0.43734	-2.90103	0.003719	0.012075	TRUE	Gm26782
Rian	3016.554	-2.010460741	0.183645	-10.9476	6.83E-28	7.68E-26	TRUE	Rian
700112J16R	5.594365	-4.61767261	1.61438	-2.86034	0.004232	0.013464	TRUE	1700112J16Rik
Mir124a-1h	1589.31	-2.342228522	0.29806	-7.85824	3.90E-15	1.20E-13	TRUE	Mir124a-1hg
930578M01F	131.036	-1.081466126	0.375172	-2.88259	0.003944	0.012679	TRUE	4930578M01Rik
230323A14F	12.11143	-5.733557142	1.352053	-4.24063	2.23E-05	0.000133	TRUE	B230323A14Rik
Mirt1	14.69095	-2.832935955	0.895552	-3.16334	0.00156	0.005695	TRUE	Mirt1
Gm16701	53.44642	-4.216654117	0.679102	-6.20917	5.33E-10	8.16E-09	TRUE	Gm16701
Gm26911	22.34519	-4.562993981	1.181937	-3.86061	0.000113	0.000565	TRUE	Gm26911

Tunar	56.37049	-3.057272847	0.536033	-5.70352	1.17E-08	1.45E-07	TRUE		Tunar
Gm27000	5.181332	-4.514903455	1.569479	-2.87669	0.004019	0.012883	TRUE		Gm27000
Gm27032	294.7162	-2.058812253	0.490354	-4.19863	2.69E-05	0.000158	TRUE		Gm27032
Ccdc166	88.41454	-1.703741172	0.394447	-4.31932	1.57E-05	9.74E-05	TRUE		Ccdc166
Sowahc	152.3855	-1.558166952	0.451007	-3.45486	0.000551	0.002275	TRUE		Sowahc
330012L14R	56.18097	-1.694600435	0.472008	-3.5902	0.00033	0.001456	TRUE		B830012L14Rik
Gm18014	3.804506	-4.063111451	1.644483	-2.47075	0.013483	0.035812	TRUE		Gm18014
Gm27217	13.90402	-2.312577072	0.851658	-2.71538	0.00662	0.019641	TRUE		Gm27217
Kctd12	128.9441	-2.402086836	0.479293	-5.01172	5.39E-07	4.72E-06	TRUE		Kctd12
Gm27199	31.44055	-5.140883499	1.147767	-4.47903	7.50E-06	5.01E-05	TRUE		Gm27199
Otx2os1	38.67343	-7.407926967	1.28075	-5.78405	7.29E-09	9.27E-08	TRUE		Otx2os1
Gm2164	4.562798	-4.332404514	1.635679	-2.64869	0.00808	0.023262	TRUE		Gm2164
Jmjd7	6.077292	-3.046133088	1.283441	-2.37341	0.017625	0.044754	TRUE		Jmjd7
Gm27544	34.27804	-4.416732439	0.833122	-5.30142	1.15E-07	1.16E-06	TRUE		Gm27544
Gm28703	5.467544	-4.593900292	1.634878	-2.80993	0.004955	0.015399	TRUE		Gm28703
Gm29325	3.557079	-3.965826559	1.673502	-2.36978	0.017799	0.04512	TRUE		Gm29325
Gm28528	6.339087	-4.805500813	1.518502	-3.16463	0.001553	0.005672	TRUE		Gm28528
Gm9451	4.154461	-4.192231016	1.615777	-2.59456	0.009471	0.026619	TRUE		Gm9451
Ppnr	36.52453	-3.583795185	0.735374	-4.87343	1.10E-06	8.92E-06	TRUE		Ppnr
Gm29374	20.46444	-4.785582035	1.056128	-4.53125	5.86E-06	4.02E-05	TRUE		Gm29374
310013P06R	193.5406	-1.150759194	0.34577	-3.32811	0.000874	0.003426	TRUE		2810013P06Rik
Gm10421	34.68908	-7.255720977	1.275788	-5.68725	1.29E-08	1.58E-07	TRUE		Gm10421
130024G19R	51.23111	-5.989094341	1.049014	-5.70926	1.13E-08	1.40E-07	TRUE		B130024G19Rik
Ppp1ccb	82.46317	-1.851422578	0.373321	-4.95933	7.07E-07	6.03E-06	TRUE		Ppp1ccb
Gm28512	52.01605	-2.05019951	0.69343	-2.95661	0.00311	0.010345	TRUE		Gm28512
Gm29093	10.72393	-2.08734559	0.880406	-2.37089	0.017745	0.045028	TRUE		Gm29093
Slc18a3	4.116814	-4.183611245	1.676059	-2.4961	0.012557	0.033709	TRUE		Slc18a3
Mir124-2hg	4505.778	-1.643704741	0.294847	-5.57477	2.48E-08	2.87E-07	TRUE		Mir124-2hg
Gm29478	42.12263	-6.541831341	1.200853	-5.44765	5.10E-08	5.56E-07	TRUE		Gm29478
Gm29170	91.03425	-1.105835712	0.449876	-2.45809	0.013968	0.03687	TRUE		Gm29170
Gm28455	17.46636	-2.625387759	0.826178	-3.17775	0.001484	0.005453	TRUE		Gm28455
700120C14R	9.892718	-2.189903212	0.929106	-2.357	0.018423	0.046403	TRUE		1700120C14Rik
230077H06R	23.63042	-3.453032646	0.945689	-3.65134	0.000261	0.001185	TRUE		A230077H06Rik
Gm19744	13.6397	-3.291381166	0.959922	-3.4288	0.000606	0.002481	TRUE		Gm19744
Gm15459	122.1002	-1.028136473	0.398557	-2.57965	0.00989	0.027629	TRUE		Gm15459
Snhg14	1253.115	-1.283541996	0.201416	-6.37259	1.86E-10	3.02E-09	TRUE		Snhg14
Eif4a-ps4	985.4687	-1.496156053	0.236954	-6.31412	2.72E-10	4.32E-09	TRUE		Eif4a-ps4
Dnah7c	14.53059	-2.432181131	0.882703	-2.75538	0.005862	0.017784	TRUE		Dnah7c
Csnk2a3	132.455	-1.178078346	0.376833	-3.12626	0.00177	0.00635	TRUE		Csnk2a3
Gm28652	4.693075	-4.367992866	1.592785	-2.74236	0.0061	0.018381	TRUE		Gm28652
Gm19461	7.700246	-2.849419937	1.202281	-2.37001	0.017788	0.045096	TRUE		Gm19461
Gm29237	4.666021	-4.365392827	1.749528	-2.49518	0.012589	0.033779	TRUE		Gm29237
Gm5441	26.15053	-6.848887325	1.300422	-5.26667	1.39E-07	1.38E-06	TRUE		Gm5441
Gm20125	11.545	-5.666516215	1.356024	-4.17877	2.93E-05	0.00017	TRUE		Gm20125
Gm29106	8.157531	-2.475635157	1.052256	-2.35269	0.018638	0.046848	TRUE		Gm29106
430092D12R	66.54476	-1.833035524	0.558808	-3.28026	0.001037	0.003975	TRUE		9430092D12Rik
930447F24R	46.68678	-3.043045189	0.619326	-4.91348	8.95E-07	7.43E-06	TRUE		4930447F24Rik
Snrpn	925.9945	-2.156979111	0.261915	-8.23542	1.79E-16	6.36E-15	TRUE		Snrpn
Pcdha3	16.67722	-3.594891881	0.909913	-3.95081	7.79E-05	0.000406	TRUE		Pcdha3
Gm37629	7.064275	-4.957719558	1.454434	-3.40869	0.000653	0.002648	TRUE		Gm37629
Gm19938	115.5206	-2.966069085	0.43861	-6.76243	1.36E-11	2.62E-10	TRUE		Gm19938
Gm37573	12.51341	-3.268919841	1.100825	-2.96952	0.002983	0.009997	TRUE		Gm37573
730094K22R	40.95768	-6.486219734	1.251993	-5.18071	2.21E-07	2.09E-06	TRUE		A730094K22Rik
Gm37925	9.036526	-3.532699281	1.219293	-2.89734	0.003763	0.012192	TRUE		Gm37925
Gm37226	18.56103	-6.355230078	1.380061	-4.60504	4.12E-06	2.92E-05	TRUE		Gm37226
Gm16701.1	24.86201	-3.953111787	0.879513	-4.49466	6.97E-06	4.69E-05	TRUE		Gm16701.1
Gm37040	11.35801	-5.647314327	1.457211	-3.87543	0.000106	0.000536	TRUE		Gm37040
Gm37965	51.05473	-7.811759011	1.237619	-6.31193	2.76E-10	4.37E-09	TRUE		Gm37965
Gm37899	28.61838	-2.017285772	0.638178	-3.16101	0.001572	0.00573	TRUE		Gm37899
Dchs2	11.40418	-2.929313238	1.224404	-2.39244	0.016737	0.042844	TRUE		Dchs2
Pcdhac2	29.94976	-5.410342145	1.089394	-4.96638	6.82E-07	5.83E-06	TRUE		Pcdhac2
Gm32444	79.65189	-8.451162044	1.264208	-6.68494	2.31E-11	4.33E-10	TRUE		Gm32444
Pcdhga11	68.59483	-1.844839382	0.460641	-4.00494	6.20E-05	0.000332	TRUE		Pcdhga11
Gm38253	5.265752	-4.536146073	1.540158	-2.94525	0.003227	0.010659	TRUE		Gm38253
Gm37477	113.0171	-5.352115936	1.452509	-3.68474	0.000229	0.001057	TRUE		Gm37477
Gm37812	50.54306	-7.794315176	1.28143	-6.08251	1.18E-09	1.70E-08	TRUE		Gm37812
900097C17R	3433.927	-2.089583491	0.352716	-5.92427	3.14E-09	4.21E-08	TRUE		2900097C17Rik
Pcdhgc3	208.553	-3.305546156	0.335048	-9.86589	5.85E-23	4.08E-21	TRUE		Pcdhgc3
Zc3h11a	91.9688	-1.009124366	0.428553	-2.35473	0.018536	0.046643	TRUE		Zc3h11a
Gm17244	6.714696	-3.210178468	1.32965	-2.4143	0.015765	0.040808	TRUE		Gm17244
Gm37121	18.64345	-2.073345478	0.720012	-2.8796	0.003982	0.012778	TRUE		Gm37121
Gm37309	19.9963	-2.337469691	0.79271	-2.94871	0.003191	0.010561	TRUE		Gm37309

Pcdhgb8	91.79442	-1.349007652	0.360115	-3.74604	0.00018	0.000851	TRUE	Pcdhgb8
Pcdhgb6	88.32561	-1.872951667	0.447371	-4.18658	2.83E-05	0.000165	TRUE	Pcdhgb6
Gm37303	17.71567	-2.657298715	0.845038	-3.14459	0.001663	0.006011	TRUE	Gm37303
130012C08F	5.193414	-4.518117426	1.570583	-2.87671	0.004018	0.012883	TRUE	C130012C08Rik
Gm37090	134.969	-1.773560619	0.318883	-5.56179	2.67E-08	3.06E-07	TRUE	Gm37090
Pcdhac1	9.774098	-5.430603683	1.436867	-3.77948	0.000157	0.000756	TRUE	Pcdhac1
Gm37180	10.92309	-2.93903202	1.006434	-2.92024	0.003498	0.011462	TRUE	Gm37180
Gm37581	21.66802	-2.254651599	0.737902	-3.05549	0.002247	0.007846	TRUE	Gm37581
Gm15853	9.850518	-2.414260408	0.957363	-2.52178	0.011676	0.031777	TRUE	Gm15853
Gm37834	20.27086	-5.42370343	1.299986	-4.17212	3.02E-05	0.000175	TRUE	Gm37834
Pcdha6	7.43002	-5.033715169	1.452209	-3.46625	0.000528	0.002192	TRUE	Pcdha6
Gm37283	5.432663	-4.574582282	1.548882	-2.95347	0.003142	0.010436	TRUE	Gm37283
Gm37805	9.136983	-5.333112398	1.44723	-3.68505	0.000229	0.001056	TRUE	Gm37805
Gm37613	3.831142	-4.075829116	1.673299	-2.4358	0.014859	0.038808	TRUE	Gm37613
Gm19026	50.2721	-1.259733067	0.478716	-2.63148	0.008501	0.024306	TRUE	Gm19026
Gm37249	35.82261	-1.102866445	0.458988	-2.40282	0.016269	0.041847	TRUE	Gm37249
Gm10748	21.01514	-3.337001319	0.906396	-3.68162	0.000232	0.001068	TRUE	Gm10748
Tigd5	146.0257	-1.213568906	0.307278	-3.94942	7.83E-05	0.000408	TRUE	Tigd5
Gm37186	5.125643	-4.500463779	1.615355	-2.78605	0.005335	0.016414	TRUE	Gm37186
Gm38029	13.18707	-5.860272634	1.344086	-4.36004	1.30E-05	8.24E-05	TRUE	Gm38029
Gm32391	6.616082	-3.040204585	1.272745	-2.3887	0.016908	0.043219	TRUE	Gm32391
Gm37773	49.44925	-7.763400435	1.249168	-6.21486	5.14E-10	7.90E-09	TRUE	Gm37773
Pcdha2	6.687487	-4.884028564	1.561893	-3.12699	0.001766	0.006338	TRUE	Pcdha2
Gm38102	50.05027	-2.242754905	0.666246	-3.36626	0.000762	0.003034	TRUE	Gm38102
Gm38101	5.948637	-4.710777384	1.529718	-3.07951	0.002073	0.007305	TRUE	Gm38101
Gm37397	6.028213	-3.763666007	1.435478	-2.62189	0.008744	0.024902	TRUE	Gm37397
Gm37459	10.17915	-4.313203183	1.404077	-3.07191	0.002127	0.007465	TRUE	Gm37459
Gm37457	122.8807	-5.84152469	0.769447	-7.59184	3.15E-14	8.59E-13	TRUE	Gm37457
Gm38042	49.61949	-3.891246636	0.608149	-6.39851	1.57E-10	2.58E-09	TRUE	Gm38042
Pcdha7	13.20771	-5.864441185	1.474034	-3.9785	6.94E-05	0.000367	TRUE	Pcdha7
Pcdhga3	46.44004	-2.707620638	0.569835	-4.75159	2.02E-06	1.53E-05	TRUE	Pcdhga3
Gm37928	167.32	-5.600814232	0.593732	-9.43324	3.98E-21	2.31E-19	TRUE	Gm37928
Gm37964	4.453703	-4.292286861	1.654842	-2.59378	0.009493	0.026673	TRUE	Gm37964
Gm37963	35.62247	-2.170410168	0.680225	-3.19072	0.001419	0.005239	TRUE	Gm37963
Gm37165	3.716212	-4.034277284	1.722188	-2.34253	0.019153	0.047884	TRUE	Gm37165
230334C09F	132.2099	-2.003068961	0.538686	-3.71844	0.0002	0.000938	TRUE	B230334C09Rik
Gm43485	4.363546	-4.266328467	1.619728	-2.63398	0.008439	0.024145	TRUE	Gm43485
Gm19391	20.4841	-2.927906005	0.800758	-3.65642	0.000256	0.001166	TRUE	Gm19391
Sox2ot	883.2926	-1.327829931	0.217737	-6.09833	1.07E-09	1.56E-08	TRUE	Sox2ot
Gm19817	126.3695	-1.193377844	0.508477	-2.34697	0.018927	0.047397	TRUE	Gm19817
Gm43300	43.32709	-2.049177704	0.762751	-2.68656	0.007219	0.021124	TRUE	Gm43300
Gm43251	11.40391	-5.647957069	1.35484	-4.16872	3.06E-05	0.000177	TRUE	Gm43251
932422M17F	33.15952	-2.108062698	0.650398	-3.24119	0.00119	0.004491	TRUE	4932422M17Rik
Gm42829	11.31215	-3.106376152	1.082903	-2.86856	0.004123	0.013161	TRUE	Gm42829
Gm43789	6.831542	-3.086767205	1.270157	-2.43023	0.015089	0.039318	TRUE	Gm43789
Gm43679	10.48391	-3.316346467	1.066621	-3.10921	0.001876	0.00668	TRUE	Gm43679
Gm42525	6.690242	-3.194102107	1.263017	-2.52895	0.011441	0.031227	TRUE	Gm42525
Gm42609	49.68772	-3.342079399	0.619551	-5.39436	6.88E-08	7.28E-07	TRUE	Gm42609
Gm42444	6.864708	-3.882567769	1.417	-2.73999	0.006144	0.018485	TRUE	Gm42444
Gm43826	8.052482	-5.151589193	1.508514	-3.41501	0.000638	0.002594	TRUE	Gm43826
Lef1os1	10.96922	-2.741687873	1.048259	-2.61547	0.008911	0.025289	TRUE	Lef1os1
Gm42664	41.32555	-2.277201321	0.60837	-3.74312	0.000182	0.000861	TRUE	Gm42664
Gm42994	3.651332	-4.00152062	1.681972	-2.37906	0.017357	0.044192	TRUE	Gm42994
Lhfpl3	4.042003	-4.157426234	1.673436	-2.48437	0.012978	0.03464	TRUE	Lhfpl3
Gm43680	53.14327	-1.281009108	0.511189	-2.50594	0.012213	0.033015	TRUE	Gm43680
Gm42455	4.305318	-4.244829497	1.611148	-2.63466	0.008422	0.024106	TRUE	Gm42455
Gm42608	4.734333	-4.385698013	1.644032	-2.66765	0.007638	0.022174	TRUE	Gm42608
900064F13R	6.748414	-4.886762736	1.507174	-3.24233	0.001186	0.004476	TRUE	2900064F13Rik
130093G08F	84.29274	-8.535398974	1.220993	-6.99054	2.74E-12	5.85E-11	TRUE	C130093G08Rik
Peg13	1979.105	-3.810242555	0.253146	-15.0515	3.37E-51	2.20E-48	TRUE	Peg13
Gm43681	73.94539	-1.144009441	0.41329	-2.76806	0.005639	0.017204	TRUE	Gm43681
Gm43460	13.20597	-2.421628456	0.840277	-2.88194	0.003952	0.012699	TRUE	Gm43460
Gm42741	18.41893	-1.74927194	0.712205	-2.45614	0.014044	0.037057	TRUE	Gm42741
Gm7993	6.279877	-3.098483709	1.290136	-2.40167	0.01632	0.04196	TRUE	Gm7993
Gm9936	13.29706	-5.869895065	1.331312	-4.4091	1.04E-05	6.72E-05	TRUE	Gm9936
Gm43268	43.85982	-3.448189898	0.740294	-4.65786	3.20E-06	2.33E-05	TRUE	Gm43268
Gm44129	7.522274	-5.047770822	1.441376	-3.50205	0.000462	0.001949	TRUE	Gm44129
Gm44440	8.60204	-5.243380948	1.441762	-3.63679	0.000276	0.001243	TRUE	Gm44440
300002P13R	32.15527	-1.199655634	0.504896	-2.37604	0.017499	0.04448	TRUE	3300002P13Rik
Gm44441	4.342868	-4.259959199	1.6213	-2.6275	0.008602	0.024559	TRUE	Gm44441
Gm44286	50.8463	-2.472180634	0.499839	-4.94595	7.58E-07	6.40E-06	TRUE	Gm44286
Gm44439	97.78922	-2.562493539	0.694552	-3.68942	0.000225	0.00104	TRUE	Gm44439

Gm18609	9.365041	-3.586594351	1.220477	-2.93868	0.003296	0.010865	TRUE	Gm18609
Gm43951	41.94121	-1.810730504	0.574389	-3.15245	0.001619	0.00587	TRUE	Gm43951
330102E08R	56.40235	-1.415125689	0.607295	-2.33021	0.019795	0.049217	TRUE	9330102E08Rik
Gm43872	5.4945	-4.60056162	1.589766	-2.89386	0.003805	0.012299	TRUE	Gm43872
Minpp1-ps	8.112518	-5.160511656	1.438295	-3.58794	0.000333	0.001466	TRUE	Minpp1-ps
Gm44437	109.302	-1.545318135	0.460962	-3.35238	0.000801	0.003175	TRUE	Gm44437
Gm42372	258.7528	-2.050954391	0.40186	-5.10365	3.33E-07	3.03E-06	TRUE	Gm42372
Gm44509	77.82293	-1.82820305	0.367508	-4.97459	6.54E-07	5.61E-06	TRUE	Gm44509
Gm45003	12.93871	-3.127978634	1.14873	-2.72299	0.006469	0.019299	TRUE	Gm45003
Gm44510	4.50778	-4.302702193	1.682405	-2.55747	0.010544	0.029134	TRUE	Gm44510
Particl	97.69336	-1.150148809	0.435823	-2.63903	0.008314	0.023841	TRUE	Particl
Gm44769	29.23591	-2.38485306	0.784687	-3.03924	0.002372	0.008227	TRUE	Gm44769
Gm44802	5.943429	-4.713941436	1.590617	-2.96359	0.003041	0.01016	TRUE	Gm44802
30015G15R	12.54168	-5.786268784	1.33904	-4.32121	1.55E-05	9.67E-05	TRUE	9130015G15Rik
Gm39119	6.199802	-4.766836893	1.518329	-3.13953	0.001692	0.006107	TRUE	Gm39119
Gm32061	48.17522	-7.728174737	1.244344	-6.21064	5.28E-10	8.10E-09	TRUE	Gm32061
Gm44812	24.63463	-3.563608771	1.180038	-3.01991	0.002528	0.008685	TRUE	Gm44812
Gm35842	15.6908	-6.108814816	1.317478	-4.63675	3.54E-06	2.55E-05	TRUE	Gm35842
Gm45201	18.7561	-2.789419058	0.795097	-3.50827	0.000451	0.001908	TRUE	Gm45201
Gm44913	11.49244	-2.664102775	0.913247	-2.91718	0.003532	0.011559	TRUE	Gm44913
Gm44586	23.09554	-3.223611383	0.770644	-4.18301	2.88E-05	0.000167	TRUE	Gm44586
Gm44593	10.93097	-5.587044966	1.360597	-4.10632	4.02E-05	0.000226	TRUE	Gm44593
Gm44799	194.9178	-1.369769766	0.499746	-2.74093	0.006127	0.018448	TRUE	Gm44799
Gm29683	21.52353	-6.56320423	1.301432	-5.04306	4.58E-07	4.07E-06	TRUE	Gm29683
Gm44644	4.752096	-4.382661531	1.588485	-2.75902	0.005797	0.017607	TRUE	Gm44644
Gm44798	3.929643	-4.114365949	1.656542	-2.48371	0.013002	0.034695	TRUE	Gm44798
00027M19R	35.24724	-4.103575945	0.734557	-5.58646	2.32E-08	2.71E-07	TRUE	2900027M19Rik
Gm32849	9.382191	-5.368320474	1.388863	-3.86526	0.000111	0.000556	TRUE	Gm32849
Gm45104	10.84071	-3.03219352	1.069782	-2.8344	0.004591	0.014421	TRUE	Gm45104
Gm45133	76.25379	-1.613908075	0.581913	-2.77345	0.005547	0.01696	TRUE	Gm45133
Gm44562	22.81861	-3.706971114	0.85567	-4.33224	1.48E-05	9.25E-05	TRUE	Gm44562
Gm45591	74.12657	-3.035464943	0.831641	-3.64997	0.000262	0.00119	TRUE	Gm45591
Gm44801	5.813822	-4.675310044	1.500311	-3.11623	0.001832	0.006537	TRUE	Gm44801
330162G02R	355.2828	-1.83319071	0.386135	-4.74754	2.06E-06	1.56E-05	TRUE	9330162G02Rik
930435N07R	14.54795	-2.831439161	0.935277	-3.02738	0.002467	0.008495	TRUE	4930435N07Rik
Gm44831	22.79511	-3.602522353	0.988963	-3.64273	0.00027	0.00122	TRUE	Gm44831
Gm45447	21.90904	-2.058940924	0.653291	-3.15164	0.001624	0.005882	TRUE	Gm45447
Gm45426	7.117393	-4.970263071	1.451513	-3.4242	0.000617	0.002519	TRUE	Gm45426
Gm45847	65.1162	-2.874130852	0.433637	-6.62796	3.40E-11	6.22E-10	TRUE	Gm45847
Gm45605	28.70368	-1.77823875	0.614062	-2.89586	0.003781	0.012236	TRUE	Gm45605
Gm45278	4.11891	-4.181928107	1.623991	-2.57509	0.010021	0.027909	TRUE	Gm45278
Gm39244	43.68123	-6.630971826	1.168558	-5.67449	1.39E-08	1.69E-07	TRUE	Gm39244
Chmp1b	274.1188	-1.142266955	0.27944	-4.0877	4.36E-05	0.000243	TRUE	Chmp1b
Gm45601	4.298345	-4.242031042	1.647939	-2.57414	0.010049	0.027956	TRUE	Gm45601
Brd3os	112.1457	-1.311428082	0.306972	-4.27215	1.94E-05	0.000118	TRUE	Brd3os
Gm45441	4.152463	-4.18685193	1.650952	-2.53602	0.011212	0.030708	TRUE	Gm45441
030029H02R	68.34964	-2.431520285	0.568921	-4.27392	1.92E-05	0.000117	TRUE	C030029H02Rik
Gm45623	8.410185	-5.212955794	1.534362	-3.39747	0.00068	0.002747	TRUE	Gm45623
Gm4247	19.42755	-1.739821198	0.696978	-2.49623	0.012552	0.0337	TRUE	Gm4247
Gm36325	3.571633	-3.974738286	1.70101	-2.33669	0.019455	0.048574	TRUE	Gm36325
130073E24R	15.38114	-6.083460127	1.367769	-4.44772	8.68E-06	5.73E-05	TRUE	C130073E24Rik
Gm45258	5.008242	-4.467018749	1.621463	-2.75493	0.00587	0.017804	TRUE	Gm45258
Gm45516	6.088746	-4.740846118	1.499704	-3.16119	0.001571	0.00573	TRUE	Gm45516
Gm6013	8.507621	-3.434487175	1.219926	-2.81532	0.004873	0.015183	TRUE	Gm6013
C78859	34.47736	-1.999893165	0.624544	-3.20217	0.001364	0.005062	TRUE	C78859
Gm39662	3.965514	-4.121064652	1.644736	-2.50561	0.012224	0.033037	TRUE	Gm39662
Gm20276	4.628096	-4.348890618	1.56954	-2.77081	0.005592	0.017082	TRUE	Gm20276
Gm2981	4.52068	-4.316917584	1.647554	-2.6202	0.008788	0.024995	TRUE	Gm2981
Gm48743	10.05777	-5.471668933	1.428852	-3.82942	0.000128	0.000633	TRUE	Gm48743
Gm18378	4.254198	-4.229513822	1.623256	-2.60557	0.009172	0.025914	TRUE	Gm18378
Gm49759	215.3747	-1.520913512	0.449226	-3.38563	0.00071	0.002853	TRUE	Gm49759
Gm48284	10.91061	-5.58301734	1.367	-4.08414	4.42E-05	0.000246	TRUE	Gm48284
230014E18R	18.28921	-3.42786823	0.870863	-3.93618	8.28E-05	0.000428	TRUE	E230014E18Rik
Gm47640	29.34873	-1.617095566	0.58109	-2.78286	0.005388	0.016547	TRUE	Gm47640
Rab7-ps1	14.95165	-2.954024577	0.969705	-3.04631	0.002317	0.008052	TRUE	Rab7-ps1
Gm48727	6.730331	-4.89129935	1.480616	-3.30356	0.000955	0.003702	TRUE	Gm48727
Gm47472	12.49778	-5.785061961	1.518685	-3.80926	0.000139	0.000681	TRUE	Gm47472
Gm5182	88.76417	-1.265161215	0.372744	-3.39418	0.000688	0.002776	TRUE	Gm5182
Gm48893	4.122396	-4.183324174	1.627192	-2.57088	0.010144	0.028168	TRUE	Gm48893
Gm5136	7.041487	-3.135511921	1.255167	-2.49808	0.012487	0.033614	TRUE	Gm5136
Gm33680	15.53303	-6.0963319	1.318017	-4.62538	3.74E-06	2.68E-05	TRUE	Gm33680
Rmst	313.8632	-3.279237656	0.59989	-5.4664	4.59E-08	5.05E-07	TRUE	Rmst

Gm48536	16.2247	-2.974111753	0.837112	-3.55282	0.000381	0.001644	TRUE	Gm48536
930532I03R	13.15769	-4.114863696	1.135765	-3.62299	0.000291	0.001304	TRUE	4930532I03Rik
930555G07F	3.711711	-4.034883053	1.716175	-2.35109	0.018718	0.046999	TRUE	4930555G07Rik
830082N09F	146.2687	-1.928341461	0.319431	-6.0368	1.57E-09	2.21E-08	TRUE	A830082N09Rik
Gm48759	4.79909	-4.401650355	1.644418	-2.67672	0.007435	0.021648	TRUE	Gm48759
Gm48751	16.76524	-6.208354296	1.361627	-4.55951	5.13E-06	3.55E-05	TRUE	Gm48751
Sfta3-ps	30.70023	-2.676550936	1.108868	-2.41377	0.015788	0.040853	TRUE	Sfta3-ps
Gm33869	41.96413	-5.907716974	1.126715	-5.24331	1.58E-07	1.54E-06	TRUE	Gm33869
Gm48542	6.150977	-3.788624005	1.430412	-2.64862	0.008082	0.023264	TRUE	Gm48542
Gm48744	9.276453	-5.355476137	1.470539	-3.64184	0.000271	0.001223	TRUE	Gm48744
Gm47338	3.638389	-4.001428086	1.717723	-2.3295	0.019833	0.049292	TRUE	Gm47338
Gm48804	17.30572	-3.10690993	0.936833	-3.3164	0.000912	0.003553	TRUE	Gm48804
Gm20337	10.08985	-3.701463982	1.173342	-3.15463	0.001607	0.005838	TRUE	Gm20337
430024I08R	83.21106	-1.901435687	0.482227	-3.94303	8.05E-05	0.000418	TRUE	E430024I08Rik
Gm48700	34.66098	-2.896713916	0.692714	-4.18169	2.89E-05	0.000168	TRUE	Gm48700
Gm49654	124.9075	-3.119153525	0.431646	-7.22618	4.97E-13	1.16E-11	TRUE	Gm49654
Tubb2a-ps2	21.97545	-4.007116171	0.872657	-4.59186	4.39E-06	3.09E-05	TRUE	Tubb2a-ps2
Gm34923	186.353	-8.042342374	0.959555	-8.38132	5.23E-17	1.97E-15	TRUE	Gm34923
Gm29675	18.99903	-2.057765497	0.735831	-2.79652	0.005166	0.015982	TRUE	Gm29675
930404H11F	4.578686	-4.334406054	1.584327	-2.7358	0.006223	0.018674	TRUE	4930404H11Rik
Gm36236	4.356604	-4.254902644	1.649345	-2.57975	0.009887	0.027624	TRUE	Gm36236
Gm48062	205.7339	-1.634080401	0.473082	-3.45412	0.000552	0.00228	TRUE	Gm48062
Gm49331	65.67597	-1.022903993	0.404266	-2.53027	0.011397	0.031134	TRUE	Gm49331
Gm20075	140.8285	-1.091359273	0.351936	-3.10102	0.001929	0.006849	TRUE	Gm20075
Gm36298	37.13947	-2.426438734	0.624036	-3.8883	0.000101	0.000512	TRUE	Gm36298
Gm3227	50.20935	-2.377250063	0.896577	-2.65147	0.008014	0.023103	TRUE	Gm3227
110006O06F	66.92089	-2.976503606	0.544949	-5.46198	4.71E-08	5.16E-07	TRUE	3110006O06Rik
Gm49336	571.78	-1.442376002	0.401312	-3.59415	0.000325	0.001436	TRUE	Gm49336
933429O19F	6.195843	-4.767194073	1.52104	-3.13417	0.001723	0.006203	TRUE	4933429O19Rik
Gm49152	10.71114	-5.554423276	1.395495	-3.98025	6.88E-05	0.000364	TRUE	Gm49152
Gm5089.1	83.36137	-3.11162214	0.768132	-4.0509	5.10E-05	0.000279	TRUE	Gm5089.1
Gm49153	7.879269	-5.117014738	1.425768	-3.58895	0.000332	0.001461	TRUE	Gm49153
Gm49204	51.5797	-2.871256145	0.635326	-4.51934	6.20E-06	4.22E-05	TRUE	Gm49204
Gm48957	44.40185	-6.673122967	1.178166	-5.66399	1.48E-08	1.79E-07	TRUE	Gm48957
Gm49092	24.91131	-2.538696889	0.652377	-3.89145	9.96E-05	0.000506	TRUE	Gm49092
Gm10832	8.684566	-2.530883667	1.02948	-2.45841	0.013955	0.036847	TRUE	Gm10832
Tmem249	3.614225	-3.992263798	1.672941	-2.38638	0.017015	0.043445	TRUE	Tmem249
730414N17F	11.89985	-3.071175068	0.960327	-3.19805	0.001384	0.005124	TRUE	5730414N17Rik
Gm49601	124.6131	-1.549316015	0.41425	-3.74005	0.000184	0.00087	TRUE	Gm49601
Gm49659	38.83513	-1.471345108	0.535497	-2.74762	0.006003	0.018146	TRUE	Gm49659
930483P17R	18.32678	-4.198564735	1.220214	-3.44084	0.00058	0.002384	TRUE	4930483P17Rik
Morf4l1-ps1	266.4533	-1.880346837	0.538737	-3.49029	0.000483	0.002025	TRUE	Morf4l1-ps1
130008D07F	219.6199	-3.817196525	0.685797	-5.56607	2.61E-08	3.00E-07	TRUE	E130008D07Rik
Gm49949	21.73619	-3.235335893	0.811274	-3.98797	6.66E-05	0.000354	TRUE	Gm49949
Gm49883	100.5185	-1.386576253	0.378431	-3.66401	0.000248	0.001135	TRUE	Gm49883
Gm18649	13.84185	-4.277509909	1.127693	-3.79315	0.000149	0.00072	TRUE	Gm18649
Gm7818	17.67551	-2.36281666	1.011473	-2.33601	0.01949	0.048619	TRUE	Gm7818
Ptp4a1.1	5.480997	-3.536492894	1.425848	-2.48027	0.013128	0.034982	TRUE	Ptp4a1.1
Gm16386	70.4049	-2.465773814	0.601187	-4.10151	4.10E-05	0.00023	TRUE	Gm16386
Ntn3	121.0656	-1.897982065	0.516339	-3.67584	0.000237	0.001089	TRUE	Ntn3
720468P15R	5.076331	-4.482111376	1.539515	-2.91138	0.003598	0.011736	TRUE	6720468P15Rik
Gm50431	94.80752	-1.799070633	0.359156	-5.00917	5.47E-07	4.78E-06	TRUE	Gm50431
630028I04R	4.228356	-4.212730003	1.661151	-2.53603	0.011212	0.030708	TRUE	9630028I04Rik
AC156546.1	176.0401	-2.375412781	0.360225	-6.59425	4.27E-11	7.68E-10	TRUE	AC156546.1
Gm29695	391.7314	-1.598941594	0.234402	-6.82136	9.02E-12	1.78E-10	TRUE	Gm29695
AC099699.1	43.77878	-1.484957999	0.538577	-2.75719	0.00583	0.017696	TRUE	AC099699.1
