# Supplementary material for: Temporal-spatial Generation of Astrocytes in the Developing Diencephalon
Source: Neurosci Bull. 2023 Oct 16;40(1):1–16. doi: 10.1007/s12264-023-01131-9 (PMC10774245; doi:10.1007/s12264-023-01131-9)
Supplement: Supplementary file 4 — Supplementary file4 (PDF 926 kb) [file 12264_2023_1131_MOESM4_ESM.pdf]

Table S4. List of genes that were down-regulated in the dorsal wall of 3V compared with those in the dorsal wall of LV

SYMBOL	baseMean	log2FoldChange	lfcSE	stat	pvalue	padj	significant	LV-d_vs_3V-d_downregulated gene
Cdc45	278.0303	1.244480825	0.26971	4.61422	3.95E-06	2.81E-05	TRUE	Cdc45
Scml2	78.86873	1.449156597	0.47705	3.03778	0.00238	0.00826	TRUE	Scml2
Ccnd2	5914.36	1.805002586	0.17896	10.0862	6.36E-24	4.89E-22	TRUE	Ccnd2
Scpep1	223.1167	1.048821819	0.23237	4.51351	6.38E-06	4.32E-05	TRUE	Scpep1
Hddc2	196.2607	1.278942911	0.32826	3.89608	9.78E-05	0.0005	TRUE	Hddc2
Lck	34.91802	2.03804471	0.51738	3.93919	8.18E-05	0.00042	TRUE	Lck
Cttnbp2	1395.353	1.273217857	0.25384	5.0159	5.28E-07	4.63E-06	TRUE	Cttnbp2
1-Sep	497.1178	1.844564025	0.32412	5.69099	1.26E-08	1.54E-07	TRUE	1-Sep
Itga5	306.6865	1.89987746	0.26404	7.19529	6.23E-13	1.44E-11	TRUE	Itga5
Sox9	1836.23	2.602662521	0.27211	9.56472	1.13E-21	6.88E-20	TRUE	Sox9
Hk2	648.9087	2.499584392	0.23444	10.6621	1.53E-26	1.47E-24	TRUE	Hk2
Loxl3	97.22933	2.009330892	0.305	6.58799	4.46E-11	7.96E-10	TRUE	Loxl3
Slc5a5	26.72088	2.04256388	0.70486	2.89783	0.00376	0.01218	TRUE	Slc5a5
Bcl11a	1937.578	1.776085504	0.27246	6.51866	7.09E-11	1.22E-09	TRUE	Bcl11a
Hip1r	519.6754	1.043439436	0.24355	4.28429	1.83E-05	0.00011	TRUE	Hip1r
Nsun5	458.2244	1.311845228	0.39123	3.35311	0.0008	0.00317	TRUE	Nsun5
Cfp	1067.325	1.902747819	0.38935	4.88702	1.02E-06	8.39E-06	TRUE	Cfp
Oas1c	9.503164	3.119949683	0.83594	3.73226	0.00019	0.00089	TRUE	Oas1c
Uhrf1	1588.308	1.248743923	0.26522	4.70837	2.50E-06	1.86E-05	TRUE	Uhrf1
Itgb7	56.61176	1.671757913	0.48527	3.44499	0.00057	0.00235	TRUE	Itgb7
Myg1	872.6774	1.652749141	0.32557	5.07654	3.84E-07	3.46E-06	TRUE	Myg1
Rarg	121.9057	2.536231489	0.44913	5.64704	1.63E-08	1.95E-07	TRUE	Rarg
Efnb2	1041.173	1.07322529	0.35352	3.03585	0.0024	0.0083	TRUE	Efnb2
Col18a1	859.3036	1.694694527	0.2285	7.41662	1.20E-13	3.04E-12	TRUE	Col18a1
Col1a1	19.85703	1.905094543	0.68431	2.78397	0.00537	0.0165	TRUE	Col1a1
Foxm1	1743.149	1.934476985	0.22522	8.5894	8.74E-18	3.57E-16	TRUE	Foxm1
Tulp3	717.3887	1.252628338	0.23824	5.25786	1.46E-07	1.44E-06	TRUE	Tulp3
Gtf2h4	292.367	1.159212153	0.26333	4.40212	1.07E-05	6.92E-05	TRUE	Gtf2h4
Acap1	43.23256	1.872780554	0.53431	3.50504	0.00046	0.00193	TRUE	Acap1
Ddx18	1097.05	1.955122794	0.36155	5.40754	6.39E-08	6.81E-07	TRUE	Ddx18
Tcirg1	489.1015	2.810264075	0.40424	6.95195	3.60E-12	7.57E-11	TRUE	Tcirg1
Tspan33	91.57192	1.841443705	0.41739	4.4118	1.03E-05	6.64E-05	TRUE	Tspan33
Aif1l	570.7079	1.835931131	0.31077	5.90765	3.47E-09	4.63E-08	TRUE	Aif1l
Ltbp1	94.83717	1.575447553	0.44449	3.54442	0.00039	0.00169	TRUE	Ltbp1
Trmt1	1815.23	1.873078824	0.38786	4.82922	1.37E-06	1.09E-05	TRUE	Trmt1
Nfix	5115.534	4.489433013	0.26362	17.0301	4.91E-65	5.68E-62	TRUE	Nfix
Slc1a5	840.6463	2.005341737	0.21101	9.50368	2.03E-21	1.21E-19	TRUE	Slc1a5
Spa17	53.99784	1.165269203	0.37806	3.08222	0.00205	0.00725	TRUE	Spa17
Npas1	45.53681	2.026445668	0.63974	3.16761	0.00154	0.00562	TRUE	Npas1
Ltbp2	15.02069	5.581693063	1.30139	4.28903	1.79E-05	0.00011	TRUE	Ltbp2
Trappc6a	190.4439	1.116206886	0.27917	3.99834	6.38E-05	0.00034	TRUE	Trappc6a
Spag5	1659.317	2.524460863	0.208	12.137	6.73E-34	1.40E-31	TRUE	Spag5
Unc119	984.6254	1.098256192	0.25719	4.27027	1.95E-05	0.00012	TRUE	Unc119
Rab34	1059.77	1.516701817	0.35042	4.32826	1.50E-05	9.40E-05	TRUE	Rab34
Ccne1	213.3426	1.540033347	0.25353	6.07444	1.24E-09	1.78E-08	TRUE	Ccne1
Smg9	1125.494	1.661238576	0.38326	4.33446	1.46E-05	9.17E-05	TRUE	Smg9
Mov10	217.6274	2.512277411	0.33089	7.59258	3.14E-14	8.55E-13	TRUE	Mov10
Tead3	930.9168	2.354630379	0.32812	7.1762	7.17E-13	1.64E-11	TRUE	Tead3
Dbf4	722.0162	1.375009052	0.18383	7.47961	7.45E-14	1.92E-12	TRUE	Dbf4
Tm9sf1	498.2761	1.218527055	0.23518	5.18125	2.20E-07	2.09E-06	TRUE	Tm9sf1
Rec8	277.2584	1.753968068	0.29063	6.03504	1.59E-09	2.22E-08	TRUE	Rec8
Mdp1	789.552	1.067589523	0.3022	3.53272	0.00041	0.00176	TRUE	Mdp1
Tmem161a	685.4552	1.551386453	0.25071	6.18801	6.09E-10	9.28E-09	TRUE	Tmem161a
Tchp	693.7237	1.252613231	0.29118	4.30188	1.69E-05	0.0001	TRUE	Tchp
Akap8l	3446.696	1.58872337	0.38501	4.1264	3.68E-05	0.00021	TRUE	Akap8l
Gtf2f1	1871.66	1.412933832	0.30248	4.6711	3.00E-06	2.20E-05	TRUE	Gtf2f1
Pspn	18.8185	2.219313466	0.67497	3.28801	0.00101	0.00388	TRUE	Pspn
Prkd1	325.0762	1.008452437	0.34276	2.94219	0.00326	0.01075	TRUE	Prkd1
Pex6	1301.513	1.207595641	0.24839	4.86174	1.16E-06	9.40E-06	TRUE	Pex6
Gnmt	45.95867	1.990671605	0.4667	4.26541	2.00E-05	0.00012	TRUE	Gnmt
Pdxk-ps	13.87495	3.996584773	1.37176	2.91348	0.00357	0.01167	TRUE	Pdxk-ps
Top3a	366.6288	1.486404383	0.22842	6.50728	7.65E-11	1.31E-09	TRUE	Top3a
Hdgfl2	3709.626	1.517725324	0.34248	4.43157	9.35E-06	6.11E-05	TRUE	Hdgfl2
Chaf1a	590.2586	1.648308482	0.21845	7.54545	4.51E-14	1.20E-12	TRUE	Chaf1a
Mcm2	856.5271	1.08062227	0.4096	2.63824	0.00833	0.02389	TRUE	Mcm2
Tpra1	341.8178	1.197560116	0.26791	4.46999	7.82E-06	5.20E-05	TRUE	Tpra1
Arrdc2	376.0455	1.626671016	0.38461	4.22936	2.34E-05	0.00014	TRUE	Arrdc2
Timm44	1057.01	1.030005861	0.25245	4.07997	4.50E-05	0.00025	TRUE	Timm44
Apoe	781.0287	1.737751964	0.3551	4.8937	9.90E-07	8.12E-06	TRUE	Apoe
Stk11	3953.737	1.359784459	0.27799	4.89144	1.00E-06	8.21E-06	TRUE	Stk11
Lipe	247.3137	2.502072913	0.36166	6.91831	4.57E-12	9.44E-11	TRUE	Lipe
Irf3	1011.496	1.969191917	0.31551	6.24137	4.34E-10	6.73E-09	TRUE	Irf3

Bcl2l12	164.1789	2.575817941	0.31159	8.26675	1.38E-16	4.92E-15	TRUE		Bcl2l12
Grik5	7449.232	1.340375024	0.38397	3.49083	0.00048	0.00202	TRUE		Grik5
Cd79a	12.0614	3.043669592	0.8663	3.5134	0.00044	0.00188	TRUE		Cd79a
Pih1d1	1285.717	1.059334469	0.40029	2.64643	0.00813	0.0234	TRUE		Pih1d1
Paf1	1589.701	1.037081031	0.27536	3.76629	0.00017	0.00079	TRUE		Paf1
Timm50	1568.638	1.029496812	0.30131	3.41673	0.00063	0.00258	TRUE		Timm50
Crhr2	7.322033	3.107709829	0.91963	3.37929	0.00073	0.00291	TRUE		Crhr2
Coq8b	717.2373	1.396677848	0.39787	3.51041	0.00045	0.0019	TRUE		Coq8b
Kif20a	293.6578	1.215188941	0.26381	4.60631	4.10E-06	2.91E-05	TRUE		Kif20a
Gcdh	571.4754	1.606147697	0.32594	4.92768	8.32E-07	6.96E-06	TRUE		Gcdh
Dnase2a	334.4136	2.818805302	0.51097	5.51663	3.46E-08	3.88E-07	TRUE		Dnase2a
Syce2	194.5823	1.930814116	0.3632	5.31609	1.06E-07	1.08E-06	TRUE		Syce2
Ppan	1559.282	2.360326589	0.3408	6.92575	4.34E-12	9.04E-11	TRUE		Ppan
Trmt10a	141.756	1.067757744	0.30554	3.49469	0.00047	0.002	TRUE		Trmt10a
Atn1	236.8284	1.243679633	0.3951	3.14774	0.00165	0.00595	TRUE		Atn1
Hif3a	167.4297	1.925273312	0.34927	5.51229	3.54E-08	3.97E-07	TRUE		Hif3a
Pde1c	214.1221	1.002624911	0.3478	2.88276	0.00394	0.01267	TRUE		Pde1c
Ndrg2	820.9442	2.510541229	0.34054	7.37218	1.68E-13	4.15E-12	TRUE		Ndrg2
Etfb	45.99541	1.741809894	0.44975	3.87281	0.00011	0.00054	TRUE		Etfb
Stxbp2	404.8432	1.754879283	0.33199	5.28598	1.25E-07	1.25E-06	TRUE		Stxbp2
Aqp1	15.47389	2.26896106	0.67472	3.3628	0.00077	0.00307	TRUE		Aqp1
Myo9b	3143.458	1.561004758	0.25609	6.09551	1.09E-09	1.58E-08	TRUE		Myo9b
Mtfp1	36.5327	1.417322663	0.57062	2.48382	0.013	0.03469	TRUE		Mtfp1
Plod3	1101.1	1.232405089	0.31594	3.90082	9.59E-05	0.00049	TRUE		Plod3
Srpk1	3158.129	1.070358637	0.2156	4.96444	6.89E-07	5.88E-06	TRUE		Srpk1
Bcan	782.166	4.283989965	1.10408	3.88013	0.0001	0.00053	TRUE		Bcan
Rrnad1	727.9988	1.172143591	0.22735	5.15558	2.53E-07	2.36E-06	TRUE		Rrnad1
Thop1	1408.269	1.441287378	0.32247	4.46948	7.84E-06	5.22E-05	TRUE		Thop1
Apba3	331.7638	1.050286419	0.25899	4.05528	5.01E-05	0.00027	TRUE		Apba3
Matk	179.7221	1.965982928	0.36466	5.39123	7.00E-08	7.39E-07	TRUE		Matk
Nmrk2	6.595555	5.198590141	2.01707	2.5773	0.00996	0.02778	TRUE		Nmrk2
Ccdc130	307.1205	1.400141445	0.33841	4.13739	3.51E-05	0.0002	TRUE		Ccdc130
Eif2ak4	408.8671	1.16734806	0.24274	4.80902	1.52E-06	1.19E-05	TRUE		Eif2ak4
Ccn4	9.705959	8.017619378	1.61922	4.95152	7.36E-07	6.24E-06	TRUE		Ccn4
Man2b1	370.7799	1.527126752	0.38111	4.0071	6.15E-05	0.00033	TRUE		Man2b1
Slc1a3	635.2948	3.053932935	0.2884	10.5892	3.35E-26	3.10E-24	TRUE		Slc1a3
Mlxipl	52.63114	1.036594129	0.43838	2.36462	0.01805	0.0456	TRUE		Mlxipl
Bud23	722.9618	1.17775388	0.2328	5.05917	4.21E-07	3.77E-06	TRUE		Bud23
Mcm5	1132.599	1.9984115	0.21673	9.2208	2.95E-20	1.59E-18	TRUE		Mcm5
Mef2c	228.6364	1.740816382	0.29455	5.91019	3.42E-09	4.57E-08	TRUE		Mef2c
Tmod4	36.54758	1.661226996	0.44597	3.72501	0.0002	0.00092	TRUE		Tmod4
Apoa2	17.12164	2.669127112	0.89467	2.98338	0.00285	0.00962	TRUE		Apoa2
Pan2	1871.13	1.371955997	0.27004	5.08048	3.76E-07	3.39E-06	TRUE		Pan2
Gpr108	415.4147	1.369254457	0.30804	4.44505	8.79E-06	5.79E-05	TRUE		Gpr108
Smpd4	1443.5	1.181574384	0.23693	4.98709	6.13E-07	5.29E-06	TRUE		Smpd4
Itgae	31.49151	1.751156766	0.56149	3.11877	0.00182	0.00649	TRUE		Itgae
Kptn	571.2159	1.091493817	0.33055	3.30205	0.00096	0.00372	TRUE		Kptn
Eps15l1	1954.189	1.07397381	0.30207	3.55538	0.00038	0.00163	TRUE		Eps15l1
Tmbim1	126.3708	1.655075043	0.35661	4.64111	3.47E-06	2.51E-05	TRUE		Tmbim1
Upk1a	37.04145	1.878938335	0.5836	3.21956	0.00128	0.0048	TRUE		Upk1a
Gcat	39.94985	3.062751681	0.52505	5.83328	5.43E-09	7.08E-08	TRUE		Gcat
Elovl1	276.0631	1.43291329	0.38257	3.74551	0.00018	0.00085	TRUE		Elovl1
Hyi	549.6231	1.524882439	0.31665	4.81565	1.47E-06	1.16E-05	TRUE		Hyi
Nsmf	1702.916	1.145359275	0.24238	4.72539	2.30E-06	1.73E-05	TRUE		Nsmf
Pdk1	286.3313	1.189996071	0.25622	4.64436	3.41E-06	2.47E-05	TRUE		Pdk1
Cyba	65.81234	1.29347554	0.45554	2.83942	0.00452	0.01422	TRUE		Cyba
Cdt1	486.7253	2.154688342	0.24148	8.92276	4.55E-19	2.13E-17	TRUE		Cdt1
P4htm	326.4789	1.46892092	0.41265	3.55975	0.00037	0.00161	TRUE		P4htm
Gmnn	198.2964	1.51465354	0.50071	3.02501	0.00249	0.00855	TRUE		Gmnn
Zfp184	444.6886	1.313630677	0.27276	4.81609	1.46E-06	1.16E-05	TRUE		Zfp184
Mettl1	1143.042	3.184790461	0.46681	6.82241	8.95E-12	1.78E-10	TRUE		Mettl1
Saal1	424.6272	1.125446821	0.29863	3.76867	0.00016	0.00079	TRUE		Saal1
Vars	4398.769	2.104812208	0.28371	7.419	1.18E-13	2.99E-12	TRUE		Vars
Vwa7	43.64695	2.279116815	0.53103	4.29188	1.77E-05	0.00011	TRUE		Vwa7
Msh5	177.6648	2.112834991	0.32577	6.48565	8.83E-11	1.49E-09	TRUE		Msh5
Pole	1027.921	2.488338234	0.20299	12.2587	1.51E-34	3.31E-32	TRUE		Pole
Casq1	65.82026	2.669967577	0.38459	6.94236	3.86E-12	8.07E-11	TRUE		Casq1
Gtpbp3	474.4996	1.14600095	0.24005	4.77398	1.81E-06	1.39E-05	TRUE		Gtpbp3
Rad51c	204.9988	1.102927233	0.26397	4.1783	2.94E-05	0.00017	TRUE		Rad51c
Dio2	9.890052	6.301910712	1.74665	3.60799	0.00031	0.00137	TRUE		Dio2
Cpt1c	1946.148	1.678239263	0.36105	4.64821	3.35E-06	2.43E-05	TRUE		Cpt1c
Ankrd26	707.1241	1.46766249	0.28394	5.16887	2.36E-07	2.21E-06	TRUE		Ankrd26
Aldh16a1	498.4132	1.630886331	0.30083	5.42123	5.92E-08	6.36E-07	TRUE		Aldh16a1
Prrg2	180.4446	1.379246338	0.30184	4.56952	4.89E-06	3.41E-05	TRUE		Prrg2
Tcap	42.43191	2.455625396	0.65414	3.75398	0.00017	0.00083	TRUE		Tcap

Rplp1	6914.258	1.092976619	0.26531	4.11961	3.80E-05	0.00021	TRUE	Rplp1
Fgfrl1	182.0572	3.028501485	0.29609	10.2284	1.48E-24	1.23E-22	TRUE	Fgfrl1
930432K21R	75.16022	1.054581164	0.39404	2.67631	0.00744	0.02167	TRUE	4930432K21Rik
Spib	5.374392	3.213706146	1.37952	2.32958	0.01983	0.04929	TRUE	Spib
Cers4	1902.774	1.384468831	0.34354	4.03003	5.58E-05	0.0003	TRUE	Cers4
700109H08R	82.3244	1.217953059	0.42349	2.87598	0.00403	0.01291	TRUE	1700109H08Rik
Elk3	194.1648	1.517666084	0.23989	6.32662	2.51E-10	3.99E-09	TRUE	Elk3
Nfib	8836.302	3.139012972	0.28177	11.1404	7.97E-29	1.01E-26	TRUE	Nfib
Dqx1	59.42731	2.386943427	0.46804	5.0999	3.40E-07	3.08E-06	TRUE	Dqx1
Syn2	131.9084	1.614806687	0.37238	4.33646	1.45E-05	9.10E-05	TRUE	Syn2
Fpgs	236.8109	1.258185686	0.29693	4.23736	2.26E-05	0.00014	TRUE	Fpgs
Kif19a	181.6525	1.31641381	0.41015	3.20962	0.00133	0.00494	TRUE	Kif19a
Zmynd10	32.23389	1.977971248	0.67176	2.94448	0.00323	0.01068	TRUE	Zmynd10
Hyal1	164.4303	1.717424697	0.36057	4.76315	1.91E-06	1.46E-05	TRUE	Hyal1
Slc38a3	495.4475	2.262837773	0.31498	7.18399	6.77E-13	1.55E-11	TRUE	Slc38a3
Nxf1	2676.595	1.015730364	0.23596	4.30467	1.67E-05	0.0001	TRUE	Nxf1
Stx5a	1787.078	1.280643844	0.31928	4.011	6.05E-05	0.00032	TRUE	Stx5a
Rdm1	346.5257	2.722052118	0.28669	9.49487	2.20E-21	1.32E-19	TRUE	Rdm1
Eya4	99.48041	3.455974387	0.35515	9.73099	2.22E-22	1.44E-20	TRUE	Eya4
Rbm25	13493.79	2.480325946	0.42677	5.8119	6.18E-09	7.99E-08	TRUE	Rbm25
Tnfrsf22	35.48156	1.40301648	0.5771	2.43114	0.01505	0.03923	TRUE	Tnfrsf22
Cars	1023.62	1.426939559	0.36124	3.95017	7.81E-05	0.00041	TRUE	Cars
Kdelr3	20.41138	1.334551402	0.57431	2.32374	0.02014	0.04991	TRUE	Kdelr3
Vipr2	57.70042	2.511890897	0.4331	5.79979	6.64E-09	8.55E-08	TRUE	Vipr2
Pabpc4	4209.809	1.39184071	0.36359	3.82801	0.00013	0.00064	TRUE	Pabpc4
Pinlyp	4.528623	6.853173475	1.71982	3.98483	6.75E-05	0.00036	TRUE	Pinlyp
Ubxn11	138.5864	1.124197476	0.37326	3.01182	0.0026	0.00888	TRUE	Ubxn11
Tjap1	1325.915	1.603356828	0.24318	6.5932	4.30E-11	7.73E-10	TRUE	Tjap1
Kif11	1322.231	1.428464694	0.29015	4.92314	8.52E-07	7.09E-06	TRUE	Kif11
Mlkl	4.580979	2.882145247	1.21084	2.38028	0.0173	0.04406	TRUE	Mlkl
Etv5	158.6719	3.489801859	0.4495	7.76381	8.24E-15	2.44E-13	TRUE	Etv5
Enkd1	935.3409	3.000539199	0.42892	6.99556	2.64E-12	5.66E-11	TRUE	Enkd1
Igf2bp1	2939.898	1.109445279	0.28773	3.85589	0.00012	0.00057	TRUE	Igf2bp1
B4galnt2	7.19103	2.985746334	0.9906	3.01409	0.00258	0.00882	TRUE	B4galnt2
Ly6g6e	10.07511	5.160795297	1.4595	3.53601	0.00041	0.00174	TRUE	Ly6g6e
Tmem259	3862.551	1.778076958	0.35543	5.00265	5.65E-07	4.92E-06	TRUE	Tmem259
Rnf168	1859.646	1.684579107	0.27915	6.03473	1.59E-09	2.23E-08	TRUE	Rnf168
Bicc1	74.87073	2.023113722	0.37265	5.42901	5.67E-08	6.13E-07	TRUE	Bicc1
Gip	6.064192	3.502099896	1.42947	2.44994	0.01429	0.03756	TRUE	Gip
Mertk	29.78361	2.334386255	0.65911	3.54171	0.0004	0.00171	TRUE	Mertk
Cmc2	201.9775	1.115090723	0.31189	3.57525	0.00035	0.00153	TRUE	Cmc2
Tex261	837.3156	1.056852341	0.30586	3.45531	0.00055	0.00227	TRUE	Tex261
Dll1	951.2192	1.120411787	0.22108	5.06795	4.02E-07	3.61E-06	TRUE	Dll1
Fhod1	299.508	1.424302283	0.2251	6.32746	2.49E-10	3.97E-09	TRUE	Fhod1
Slc9a5	1016.413	1.187395677	0.28266	4.2008	2.66E-05	0.00016	TRUE	Slc9a5
Msh3	489.3643	1.128290811	0.19006	5.93656	2.91E-09	3.93E-08	TRUE	Msh3
Rabl6	3756.982	1.835077518	0.39703	4.62206	3.80E-06	2.72E-05	TRUE	Rabl6
Slc25a13	91.5827	1.238989643	0.49333	2.51151	0.01202	0.03255	TRUE	Slc25a13
Tsr3	696.8219	1.280280944	0.36894	3.47013	0.00052	0.00216	TRUE	Tsr3
Cyp2j9	30.4001	4.339684479	0.64846	6.69229	2.20E-11	4.13E-10	TRUE	Cyp2j9
Pcolce2	20.29478	1.644946382	0.59115	2.7826	0.00539	0.01656	TRUE	Pcolce2
Trabd	1125.712	1.113608983	0.29232	3.80953	0.00014	0.00068	TRUE	Trabd
Ager	599.3164	3.273658342	0.42132	7.77008	7.84E-15	2.34E-13	TRUE	Ager
Egfl8	65.8788	2.135290027	0.47895	4.45826	8.26E-06	5.47E-05	TRUE	Egfl8
Nat9	324.4882	1.81437652	0.46188	3.92821	8.56E-05	0.00044	TRUE	Nat9
Lama5	497.1739	4.016252849	0.20703	19.399	7.87E-84	2.73E-80	TRUE	Lama5
Plekho1	3477.721	1.826102394	0.33522	5.44755	5.11E-08	5.56E-07	TRUE	Plekho1
Gnrh1	23.16031	1.93936054	0.64103	3.02537	0.00248	0.00854	TRUE	Gnrh1
Prpsap1	1727.393	1.271759394	0.284	4.47808	7.53E-06	5.03E-05	TRUE	Prpsap1
Fam184b	83.67232	2.9888248	0.39837	7.50259	6.26E-14	1.62E-12	TRUE	Fam184b
Gtf2ird2	809.3866	1.213396555	0.31094	3.90237	9.53E-05	0.00049	TRUE	Gtf2ird2
Celsr1	1925.564	4.048679074	0.29122	13.9025	6.11E-44	2.45E-41	TRUE	Celsr1
Lonrf3	17.56297	2.255663637	0.87844	2.56779	0.01023	0.02839	TRUE	Lonrf3
Mpped2	2967.112	4.252846769	0.21683	19.614	1.17E-85	4.89E-82	TRUE	Mpped2
Foxred2	286.6526	1.152260334	0.20829	5.53188	3.17E-08	3.59E-07	TRUE	Foxred2
Etaa1	225.42	1.131588391	0.36852	3.07063	0.00214	0.00749	TRUE	Etaa1
Sdc4	42.69956	1.847860062	0.4811	3.84092	0.00012	0.00061	TRUE	Sdc4
Brca1	324.2493	1.469999093	0.22015	6.67732	2.43E-11	4.54E-10	TRUE	Brca1
Nt5c3b	1039.792	1.1482265	0.23391	4.90875	9.17E-07	7.60E-06	TRUE	Nt5c3b
Aldoc	1417.428	6.403726742	0.43313	14.7848	1.84E-49	1.06E-46	TRUE	Aldoc
Nek8	153.9661	1.110470279	0.32958	3.3693	0.00075	0.003	TRUE	Nek8
Pipox	134.967	1.25787911	0.26968	4.66427	3.10E-06	2.27E-05	TRUE	Pipox
Cdc6	235.9853	2.051635715	0.31767	6.45843	1.06E-10	1.77E-09	TRUE	Cdc6
Atad5	487.1807	1.46295708	0.20001	7.31442	2.59E-13	6.22E-12	TRUE	Atad5
Rhbdl3	1687.684	1.5033039	0.22829	6.58497	4.55E-11	8.11E-10	TRUE	Rhbdl3

Ada	30.51279	1.377190704	0.5654	2.43577	0.01486	0.03881	TRUE	Ada
Afmid	41.01921	2.075076187	0.40316	5.14704	2.65E-07	2.46E-06	TRUE	Afmid
Wfdc2	22.57368	1.636580261	0.64406	2.54105	0.01105	0.03034	TRUE	Wfdc2
Etv4	30.46087	1.681725435	0.61652	2.72777	0.00638	0.01906	TRUE	Etv4
Slc12a7	860.1357	1.085525942	0.20936	5.18485	2.16E-07	2.05E-06	TRUE	Slc12a7
Ctsa	1132.895	1.473055469	0.32064	4.59412	4.35E-06	3.06E-05	TRUE	Ctsa
Slc12a4	148.8988	2.982725545	0.38881	7.6715	1.70E-14	4.79E-13	TRUE	Slc12a4
Dhx58	35.07442	1.724887219	0.51596	3.34306	0.00083	0.00327	TRUE	Dhx58
Mybl2	647.6665	1.82724471	0.25699	7.11008	1.16E-12	2.60E-11	TRUE	Mybl2
Ddx27	836.2418	1.092227649	0.24162	4.52042	6.17E-06	4.20E-05	TRUE	Ddx27
Hist1h2bc	61.89118	1.604040538	0.47356	3.38721	0.00071	0.00284	TRUE	Hist1h2bc
Mfng	2134.57	1.534530028	0.26484	5.7942	6.86E-09	8.77E-08	TRUE	Mfng
Anxa6	226.3865	1.178137574	0.3711	3.17471	0.0015	0.0055	TRUE	Anxa6
Cep95	505.5079	1.351127689	0.27938	4.83623	1.32E-06	1.05E-05	TRUE	Cep95
Ncor1	8010.201	1.828570773	0.41348	4.42242	9.76E-06	6.35E-05	TRUE	Ncor1
Ybx2	506.5596	1.179594468	0.34867	3.38314	0.00072	0.00288	TRUE	Ybx2
Slc2a4	156.2968	1.35268201	0.2557	5.29006	1.22E-07	1.23E-06	TRUE	Slc2a4
310408A11F	410.745	1.037478662	0.22905	4.52952	5.91E-06	4.05E-05	TRUE	2810408A11Rik
Dusp14	69.5832	3.986977195	0.52997	7.52308	5.35E-14	1.41E-12	TRUE	Dusp14
Tada2a	656.0693	1.655874458	0.22587	7.33094	2.29E-13	5.54E-12	TRUE	Tada2a
Cdk5rap3	1898.861	1.410354963	0.34314	4.1101	3.95E-05	0.00022	TRUE	Cdk5rap3
Cd68	174.9287	1.779942238	0.35516	5.01162	5.40E-07	4.72E-06	TRUE	Cd68
Smyd4	219.4933	1.449826888	0.32933	4.4023	1.07E-05	6.92E-05	TRUE	Smyd4
Avpi1	35.27539	1.810895702	0.4791	3.77977	0.00016	0.00076	TRUE	Avpi1
Wwc1	860.7885	1.556502203	0.20494	7.59501	3.08E-14	8.41E-13	TRUE	Wwc1
Mrpl45	774.0632	1.278889865	0.37679	3.39415	0.00069	0.00278	TRUE	Mrpl45
Irf1	19.72476	3.257633435	0.6579	4.95154	7.36E-07	6.24E-06	TRUE	Irf1
Cxcl16	96.59091	1.257042129	0.50762	2.47634	0.01327	0.03532	TRUE	Cxcl16
Pelp1	1377.036	1.473149477	0.31424	4.68793	2.76E-06	2.04E-05	TRUE	Pelp1
E2f2	544.5258	1.801482163	0.21199	8.49787	1.93E-17	7.54E-16	TRUE	E2f2
Hars2	915.5523	1.004366905	0.24008	4.18346	2.87E-05	0.00017	TRUE	Hars2
Chtf18	670.3245	2.526723623	0.28378	8.90375	5.40E-19	2.49E-17	TRUE	Chtf18
Ppp1r12c	3263.386	1.892019548	0.3381	5.59603	2.19E-08	2.57E-07	TRUE	Ppp1r12c
Psmc3ip	218.8238	1.09857648	0.37886	2.89973	0.00373	0.01211	TRUE	Psmc3ip
D8Ertd738e	1426.932	1.356766238	0.32171	4.21731	2.47E-05	0.00015	TRUE	D8Ertd738e
Ddx39b	7324.153	1.00157406	0.28209	3.5506	0.00038	0.00166	TRUE	Ddx39b
Tlcd1	103.1299	1.341790836	0.33105	4.05315	5.05E-05	0.00028	TRUE	Tlcd1
Arhgef25	4404.591	1.965551679	0.35893	5.47608	4.35E-08	4.81E-07	TRUE	Arhgef25
Xab2	1298.704	1.313088869	0.25791	5.09135	3.56E-07	3.21E-06	TRUE	Xab2
Trip10	215.0693	1.762231704	0.28304	6.22616	4.78E-10	7.37E-09	TRUE	Trip10
Gyg	79.98421	1.197620175	0.45165	2.65167	0.00801	0.02309	TRUE	Gyg
Rcn3	397.221	1.18333385	0.32488	3.64233	0.00027	0.00122	TRUE	Rcn3
Mrpl24	827.7865	1.071514792	0.24814	4.3182	1.57E-05	9.79E-05	TRUE	Mrpl24
Rmnd1	621.4788	1.654339886	0.33591	4.92492	8.44E-07	7.04E-06	TRUE	Rmnd1
Fbxo5	278.5775	1.1059705	0.35756	3.09314	0.00198	0.00701	TRUE	Fbxo5
Trdn	3.102838	6.562645634	2.1043	3.11869	0.00182	0.00649	TRUE	Trdn
Hint3	107.5626	1.496362153	0.36983	4.04613	5.21E-05	0.00028	TRUE	Hint3
Nr2e1	943.283	2.171305606	0.30773	7.05577	1.72E-12	3.78E-11	TRUE	Nr2e1
Cep57l1	206.4006	1.185263998	0.24018	4.93483	8.02E-07	6.74E-06	TRUE	Cep57l1
Smpd2	599.3341	1.830317187	0.33905	5.39841	6.72E-08	7.13E-07	TRUE	Smpd2
Tube1	175.2753	1.507399875	0.23076	6.53224	6.48E-11	1.13E-09	TRUE	Tube1
Smpdl3a	89.64714	1.316540201	0.32704	4.02556	5.68E-05	0.00031	TRUE	Smpdl3a
Ptprk	406.4028	2.04991267	0.34074	6.01606	1.79E-09	2.48E-08	TRUE	Ptprk
Ppp1r12a	3004.308	1.619204156	0.37031	4.37254	1.23E-05	7.82E-05	TRUE	Ppp1r12a
10-Sep	135.0085	1.243255461	0.36157	3.43853	0.00058	0.0024	TRUE	10-Sep
Cdk1	578.9052	1.269639501	0.30907	4.10799	3.99E-05	0.00022	TRUE	Cdk1
Cep290	355.5516	1.64105887	0.41361	3.96761	7.26E-05	0.00038	TRUE	Cep290
Myb	98.59547	1.089649954	0.29607	3.68037	0.00023	0.00107	TRUE	Myb
Mtfr2	114.1328	2.045122115	0.30984	6.60064	4.09E-11	7.41E-10	TRUE	Mtfr2
Cep83	646.7429	1.023867857	0.24104	4.24766	2.16E-05	0.00013	TRUE	Cep83
Socs2	1178.619	1.18348311	0.3771	3.13835	0.0017	0.00613	TRUE	Socs2
Ccar1	6855.312	1.703052923	0.31387	5.42593	5.77E-08	6.22E-07	TRUE	Ccar1
Pcsk4	86.74465	2.025579513	0.38386	5.27681	1.31E-07	1.31E-06	TRUE	Pcsk4
Pwwp3a	2575.803	1.681904388	0.26474	6.35304	2.11E-10	3.40E-09	TRUE	Pwwp3a
Gabrp	4.782754	7.150824815	1.99574	3.58304	0.00034	0.00149	TRUE	Gabrp
Tcf3	3772.748	1.102421731	0.18355	6.00603	1.90E-09	2.63E-08	TRUE	Tcf3
Spata48	16.4986	2.503057995	0.8328	3.00559	0.00265	0.00904	TRUE	Spata48
Hmg20b	1727.444	2.237948036	0.3694	6.0583	1.38E-09	1.95E-08	TRUE	Hmg20b
Ncln	1849.648	1.451513045	0.3162	4.59054	4.42E-06	3.11E-05	TRUE	Ncln
Appl2	734.6536	1.155900055	0.23296	4.96175	6.99E-07	5.96E-06	TRUE	Appl2
Rhbdf1	289.91	1.536914056	0.32172	4.77723	1.78E-06	1.37E-05	TRUE	Rhbdf1
Nprl3	505.8175	1.173853735	0.32013	3.66685	0.00025	0.00112	TRUE	Nprl3
Shc2	261.0173	1.03025608	0.38293	2.69048	0.00713	0.02092	TRUE	Shc2
Polrmt	700.9357	1.336796152	0.29881	4.47368	7.69E-06	5.13E-05	TRUE	Polrmt
Rad50	377.3354	1.873767826	0.34018	5.50823	3.62E-08	4.05E-07	TRUE	Rad50

Clk4	1806.294	1.145882441	0.2216	5.17094	2.33E-07	2.19E-06	TRUE	Clk4
Pdlim4	194.2311	1.06989376	0.27689	3.86396	0.00011	0.00056	TRUE	Pdlim4
Slu7	1077.615	1.106761502	0.38934	2.84265	0.00447	0.01409	TRUE	Slu7
Ascc2	1744.655	1.785166776	0.31681	5.6348	1.75E-08	2.09E-07	TRUE	Ascc2
Tns3	177.3763	1.70171939	0.39628	4.29419	1.75E-05	0.00011	TRUE	Tns3
310033P09R	1629.817	2.876251554	0.52952	5.43177	5.58E-08	6.04E-07	TRUE	2310033P09Rik
Trim11	614.8708	1.171958441	0.2399	4.88528	1.03E-06	8.45E-06	TRUE	Trim11
Aebp1	20.54633	1.939184503	0.70143	2.76462	0.0057	0.01736	TRUE	Aebp1
Fam114a2	2902.457	1.832005061	0.33163	5.52428	3.31E-08	3.73E-07	TRUE	Fam114a2
Myo19	352.8518	1.011031179	0.2247	4.49939	6.82E-06	4.60E-05	TRUE	Myo19
Ggnbp2	2536.847	1.420719053	0.36465	3.89616	9.77E-05	0.0005	TRUE	Ggnbp2
Shmt1	212.0362	1.283039758	0.33474	3.83292	0.00013	0.00063	TRUE	Shmt1
Srebf1	1582.483	1.600961349	0.23958	6.6823	2.35E-11	4.40E-10	TRUE	Srebf1
Twistnb	549.0721	1.290277972	0.29603	4.35859	1.31E-05	8.29E-05	TRUE	Twistnb
Ntsr2	10.66001	4.265202889	1.01685	4.19452	2.73E-05	0.00016	TRUE	Ntsr2
Arsg	27.59976	1.561691371	0.51756	3.01739	0.00255	0.00874	TRUE	Arsg
Rnaseh1	256.4437	1.047010145	0.27784	3.76834	0.00016	0.00079	TRUE	Rnaseh1
Itsn2	256.6854	1.502509993	0.26826	5.60087	2.13E-08	2.51E-07	TRUE	Itsn2
Id2	174.6044	1.847525375	0.40496	4.56221	5.06E-06	3.51E-05	TRUE	Id2
Dus4l	109.9343	1.944621087	0.27024	7.1959	6.20E-13	1.43E-11	TRUE	Dus4l
Bcap29	109.7443	1.050092299	0.38269	2.74396	0.00607	0.01831	TRUE	Bcap29
Adcy3	446.414	1.943093112	0.36809	5.27887	1.30E-07	1.30E-06	TRUE	Adcy3
Dnmt3a	5573.194	1.30100943	0.44899	2.89765	0.00376	0.01219	TRUE	Dnmt3a
Pxdn	2979.649	1.181280154	0.19846	5.95225	2.64E-09	3.59E-08	TRUE	Pxdn
Taf15	2962.281	1.025249006	0.16897	6.06772	1.30E-09	1.85E-08	TRUE	Taf15
Nle1	379.4026	1.327904287	0.28107	4.72443	2.31E-06	1.73E-05	TRUE	Nle1
Mrc2	623.1135	2.172820255	0.23311	9.3209	1.15E-20	6.43E-19	TRUE	Mrc2
Ftsj3	1151.768	1.605703352	0.25961	6.18497	6.21E-10	9.44E-09	TRUE	Ftsj3
Grin2c	33.39688	1.269480675	0.53834	2.35813	0.01837	0.04629	TRUE	Grin2c
Nup85	1107.692	1.042928288	0.1706	6.11339	9.75E-10	1.43E-08	TRUE	Nup85
Mif4gd	106.3311	1.870911513	0.34831	5.37145	7.81E-08	8.16E-07	TRUE	Mif4gd
Slc25a19	523.3198	1.586302572	0.29652	5.34973	8.81E-08	9.11E-07	TRUE	Slc25a19
Recql5	784.2967	1.196923088	0.34862	3.43336	0.0006	0.00244	TRUE	Recql5
Itgb4	38.76212	2.592798362	0.50912	5.09269	3.53E-07	3.19E-06	TRUE	Itgb4
Unk	1695.489	1.166770298	0.24627	4.73777	2.16E-06	1.63E-05	TRUE	Unk
Mrpl38	712.0368	1.184019874	0.25743	4.59943	4.24E-06	2.99E-05	TRUE	Mrpl38
Tsen54	531.0588	1.471069333	0.34385	4.27826	1.88E-05	0.00012	TRUE	Tsen54
Rhbdf2	20.29893	2.451746968	0.81519	3.00757	0.00263	0.00899	TRUE	Rhbdf2
Kif1c	909.7062	1.047859953	0.27395	3.82505	0.00013	0.00064	TRUE	Kif1c
Pld2	57.53766	1.791085134	0.40308	4.44344	8.85E-06	5.83E-05	TRUE	Pld2
Luc7l3	19064.6	2.760057357	0.44858	6.15287	7.61E-10	1.14E-08	TRUE	Luc7l3
Xylt2	686.5561	1.683045391	0.35468	4.7452	2.08E-06	1.58E-05	TRUE	Xylt2
Scrn2	249.8226	1.258101772	0.30965	4.06297	4.85E-05	0.00027	TRUE	Scrn2
Aurkb	629.6734	1.453441696	0.33126	4.38759	1.15E-05	7.33E-05	TRUE	Aurkb
Ctc1	2123.866	1.177908255	0.29814	3.95086	7.79E-05	0.00041	TRUE	Ctc1
Top2a	3660.62	1.325600885	0.24626	5.38299	7.33E-08	7.72E-07	TRUE	Top2a
Kat2a	1438.378	1.431182765	0.25868	5.53258	3.16E-08	3.58E-07	TRUE	Kat2a
Nmt1	2100.185	1.110468347	0.36007	3.08406	0.00204	0.0072	TRUE	Nmt1
Map3k14	237.237	1.028650762	0.37206	2.76475	0.0057	0.01736	TRUE	Map3k14
Foxg1	2398.323	6.737853862	0.5867	11.4843	1.58E-30	2.54E-28	TRUE	Foxg1
Ston2	381.8565	2.34618195	0.27814	8.43534	3.30E-17	1.27E-15	TRUE	Ston2
Tshr	25.86723	4.718612801	0.65988	7.15075	8.63E-13	1.96E-11	TRUE	Tshr
Dnaaf2	48.71302	1.452476657	0.40646	3.57351	0.00035	0.00153	TRUE	Dnaaf2
Pole2	84.86941	1.99269632	0.37382	5.33061	9.79E-08	1.00E-06	TRUE	Pole2
930512B01R	24.27782	2.775821274	0.58532	4.74237	2.11E-06	1.60E-05	TRUE	4930512B01Rik
Ptpn21	326.8262	1.767051752	0.23453	7.53431	4.91E-14	1.30E-12	TRUE	Ptpn21
Gstz1	543.5206	1.468391115	0.25934	5.66195	1.50E-08	1.81E-07	TRUE	Gstz1
Timm9	370.3195	1.077210021	0.22123	4.86919	1.12E-06	9.10E-06	TRUE	Timm9
Rdh12	23.92699	2.552080232	0.62705	4.06995	4.70E-05	0.00026	TRUE	Rdh12
Zfp36l1	479.7612	1.108291454	0.43154	2.56825	0.01022	0.02836	TRUE	Zfp36l1
Smoc1	217.3936	1.671563447	0.40123	4.16606	3.10E-05	0.00018	TRUE	Smoc1
Cdca7l	78.75593	1.572559114	0.57464	2.73662	0.00621	0.01864	TRUE	Cdca7l
Ccdc88c	2064.218	2.383503079	0.23656	10.0758	7.07E-24	5.42E-22	TRUE	Ccdc88c
Tshz3	359.7696	1.86345953	0.40733	4.57484	4.77E-06	3.33E-05	TRUE	Tshz3
Dcaf4	608.1149	1.08669251	0.31687	3.42943	0.0006	0.00248	TRUE	Dcaf4
Papln	16.69726	3.47740653	0.80753	4.30625	1.66E-05	0.0001	TRUE	Papln
Acot2	58.93962	1.07976795	0.45632	2.36624	0.01797	0.04545	TRUE	Acot2
Coq6	532.3805	1.352137962	0.27362	4.94175	7.74E-07	6.52E-06	TRUE	Coq6
Abcd4	1556.279	2.838581158	0.39642	7.1606	8.03E-13	1.83E-11	TRUE	Abcd4
Tgfb3	73.70151	1.294334213	0.44671	2.8975	0.00376	0.01219	TRUE	Tgfb3
Cyp46a1	1456.83	3.37984688	0.31919	10.5888	3.36E-26	3.10E-24	TRUE	Cyp46a1
Hhipl1	393.5416	1.587901107	0.23712	6.6965	2.13E-11	4.03E-10	TRUE	Hhipl1
Amn	27.87186	1.637440681	0.61531	2.66116	0.00779	0.02255	TRUE	Amn
Mtr	279.1547	1.179275521	0.26631	4.42818	9.50E-06	6.20E-05	TRUE	Mtr
Gli3	973.8154	3.70023427	0.32376	11.4289	3.00E-30	4.66E-28	TRUE	Gli3

Sfrp4	45.50136	2.590451599	0.48546	5.33604	9.50E-08	9.76E-07	TRUE		Sfrp4
Zkscan3	2381.533	1.049158732	0.18936	5.54059	3.01E-08	3.43E-07	TRUE		Zkscan3
Aspn	33.32635	3.504128207	0.62014	5.65056	1.60E-08	1.92E-07	TRUE		Aspn
Eci2	803.9269	1.210458919	0.2507	4.82831	1.38E-06	1.09E-05	TRUE		Eci2
Rpp40	38.32153	1.148546313	0.43232	2.6567	0.00789	0.02281	TRUE		Rpp40
Riok1	780.157	1.185316861	0.27464	4.31593	1.59E-05	9.87E-05	TRUE		Riok1
Shc3	129.0856	1.389299929	0.34996	3.96994	7.19E-05	0.00038	TRUE		Shc3
Sema4d	262.6316	1.054334458	0.23072	4.56969	4.88E-06	3.41E-05	TRUE		Sema4d
Gadd45g	5138.1	2.544179468	0.38805	6.55625	5.52E-11	9.71E-10	TRUE		Gadd45g
Ror2	113.8941	4.25174982	0.38028	11.1807	5.07E-29	6.73E-27	TRUE		Ror2
Mxd3	761.56	1.535744991	0.30487	5.03744	4.72E-07	4.18E-06	TRUE		Mxd3
Pdlim7	1237.623	1.14325252	0.29596	3.86286	0.00011	0.00056	TRUE		Pdlim7
Ddx41	1259.089	1.337796448	0.31322	4.27115	1.94E-05	0.00012	TRUE		Ddx41
Fam193b	8353.379	1.896815457	0.34157	5.55328	2.80E-08	3.21E-07	TRUE		Fam193b
Ahrr	42.85083	2.05019832	0.40214	5.09817	3.43E-07	3.11E-06	TRUE		Ahrr
Irx4	2.821853	6.428316424	2.17028	2.96197	0.00306	0.0102	TRUE		Irx4
Gtf2h2	567.441	1.263670649	0.34579	3.65442	0.00026	0.00117	TRUE		Gtf2h2
Depdc1b	347.3875	2.027831604	0.58359	3.47478	0.00051	0.00213	TRUE		Depdc1b
Dhfr	340.0879	1.619127688	0.42835	3.77993	0.00016	0.00076	TRUE		Dhfr
Ppwd1	240.3707	1.542160003	0.23409	6.58803	4.46E-11	7.96E-10	TRUE		Ppwd1
Cenpk	118.0871	1.555744206	0.32353	4.80868	1.52E-06	1.19E-05	TRUE		Cenpk
Srek1ip1	787.3384	1.59046134	0.33433	4.75709	1.96E-06	1.50E-05	TRUE		Srek1ip1
Rgs7bp	120.9559	1.151235687	0.27171	4.23693	2.27E-05	0.00014	TRUE		Rgs7bp
Rnf180	145.7536	1.13501229	0.43412	2.61451	0.00894	0.02534	TRUE		Rnf180
Atxn7	573.9856	2.185506667	0.24337	8.98002	2.71E-19	1.31E-17	TRUE		Atxn7
Fezf2	574.2611	4.690247953	0.50328	9.31935	1.17E-20	6.51E-19	TRUE		Fezf2
930452B06R	76.53444	1.890248605	0.35375	5.34349	9.12E-08	9.40E-07	TRUE		4930452B06Rik
Gpx8	117.11	1.704118712	0.3591	4.74549	2.08E-06	1.58E-05	TRUE		Gpx8
Kat6b	1655.327	1.189241217	0.22587	5.26515	1.40E-07	1.39E-06	TRUE		Kat6b
Comtd1	18.76395	1.691745736	0.60221	2.80924	0.00497	0.01543	TRUE		Comtd1
Nr1d2	120.1099	1.602410767	0.32705	4.89958	9.60E-07	7.91E-06	TRUE		Nr1d2
Nudt13	318.6328	2.157314526	0.33085	6.52055	7.01E-11	1.21E-09	TRUE		Nudt13
Ecd	1320.465	1.532572352	0.35256	4.34698	1.38E-05	8.72E-05	TRUE		Ecd
Mss51	78.52783	1.30796825	0.34364	3.80619	0.00014	0.00069	TRUE		Mss51
Samd4	194.9935	2.56234737	0.33245	7.70743	1.28E-14	3.69E-13	TRUE		Samd4
Peli2	396.7177	1.607182882	0.25345	6.34124	2.28E-10	3.65E-09	TRUE		Peli2
Ppif	246.1268	1.006155378	0.29052	3.46329	0.00053	0.00221	TRUE		Ppif
Btd	110.5742	1.3415898	0.3253	4.12415	3.72E-05	0.00021	TRUE		Btd
Phf7	180.8983	1.054122006	0.35092	3.00392	0.00267	0.00909	TRUE		Phf7
Rnaseh2b	339.7483	1.30471463	0.31425	4.15189	3.30E-05	0.00019	TRUE		Rnaseh2b
Gucy1b2	87.04493	4.185065696	0.40976	10.2135	1.72E-24	1.43E-22	TRUE		Gucy1b2
Prkcd	414.0528	1.202480497	0.29286	4.10601	4.03E-05	0.00023	TRUE		Prkcd
Tkt	2103.594	1.106466573	0.21544	5.13594	2.81E-07	2.60E-06	TRUE		Tkt
Pinx1	301.024	1.964874475	0.3517	5.58686	2.31E-08	2.70E-07	TRUE		Pinx1
Ska3	235.2988	1.313058331	0.26873	4.88614	1.03E-06	8.42E-06	TRUE		Ska3
Zdhhc20	1943.495	1.439781036	0.2951	4.87899	1.07E-06	8.71E-06	TRUE		Zdhhc20
Zc3h13	2477.389	1.562975997	0.31629	4.94154	7.75E-07	6.53E-06	TRUE		Zc3h13
Rgcc	136.4958	3.962092998	0.50943	7.77746	7.40E-15	2.22E-13	TRUE		Rgcc
Naa16	1014.814	1.721348398	0.35618	4.83276	1.35E-06	1.07E-05	TRUE		Naa16
Diaph3	131.0925	2.036333255	0.3508	5.80476	6.45E-09	8.32E-08	TRUE		Diaph3
Esco2	212.2891	1.355267643	0.57267	2.36659	0.01795	0.04542	TRUE		Esco2
Clu	113.9066	1.677505647	0.39755	4.21958	2.45E-05	0.00014	TRUE		Clu
Bora	587.9719	1.781762103	0.2597	6.86076	6.85E-12	1.38E-10	TRUE		Bora
Sorbs3	215.7215	1.417154536	0.3824	3.70598	0.00021	0.00098	TRUE		Sorbs3
Hr	37.83017	1.772465384	0.4179	4.24131	2.22E-05	0.00013	TRUE		Hr
Fgf17	36.09596	2.043991701	0.69073	2.95918	0.00308	0.01028	TRUE		Fgf17
Dok2	69.22878	2.257930743	0.53341	4.23298	2.31E-05	0.00014	TRUE		Dok2
Dct	283.0738	6.711526964	0.61049	10.9936	4.10E-28	4.80E-26	TRUE		Dct
Gpr180	271.7813	1.324532142	0.2373	5.58165	2.38E-08	2.77E-07	TRUE		Gpr180
Nipbl	2665.733	1.18812193	0.34234	3.47057	0.00052	0.00216	TRUE		Nipbl
Dab2	53.54084	2.215486934	0.38816	5.7077	1.15E-08	1.41E-07	TRUE		Dab2
Haus4	301.4071	1.797578602	0.20432	8.798	1.39E-18	6.11E-17	TRUE		Haus4
Ajuba	634.954	2.403104912	0.22487	10.6867	1.18E-26	1.16E-24	TRUE		Ajuba
Acin1	9751.02	1.285038519	0.31841	4.03575	5.44E-05	0.0003	TRUE		Acin1
Ngdn	445.2793	1.080862081	0.26769	4.0378	5.40E-05	0.00029	TRUE		Ngdn
Dhrs4	420.8402	1.651567886	0.25172	6.56115	5.34E-11	9.43E-10	TRUE		Dhrs4
Carmil3	8299.893	2.597080876	0.36685	7.07942	1.45E-12	3.21E-11	TRUE		Carmil3
Tgm1	20.45633	1.501628255	0.60502	2.48195	0.01307	0.03485	TRUE		Tgm1
Rai14	1405.47	1.584801405	0.17684	8.9618	3.19E-19	1.52E-17	TRUE		Rai14
Mtdh	2323.892	1.108908339	0.2844	3.8991	9.66E-05	0.00049	TRUE		Mtdh
Myo10	939.665	1.071377981	0.35094	3.05288	0.00227	0.0079	TRUE		Myo10
Shcbp1	241.6574	1.521357131	0.2911	5.22631	1.73E-07	1.68E-06	TRUE		Shcbp1
Pop1	427.5878	1.456304588	0.21364	6.81657	9.32E-12	1.84E-10	TRUE		Pop1
Atad2	922.6415	2.103891296	0.25449	8.2672	1.37E-16	4.91E-15	TRUE		Atad2
Tbc1d31	286.2471	1.607480119	0.34751	4.62571	3.73E-06	2.68E-05	TRUE		Tbc1d31

Mtbp	234.76	1.665037522	0.26137	6.37053	1.88E-10	3.06E-09	TRUE		Mtbp
Wnt7b	844.8972	1.046666949	0.32797	3.1913	0.00142	0.00523	TRUE		Wnt7b
Gtse1	360.9078	1.197739134	0.20243	5.91687	3.28E-09	4.40E-08	TRUE		Gtse1
Tef	974.2662	1.115437917	0.21883	5.09727	3.45E-07	3.12E-06	TRUE		Tef
Adsl	622.8762	1.06310626	0.18424	5.77034	7.91E-09	9.99E-08	TRUE		Adsl
Tab1	1833.064	1.475841499	0.33245	4.43933	9.02E-06	5.93E-05	TRUE		Tab1
Dscc1	95.02619	1.522085873	0.2892	5.26305	1.42E-07	1.40E-06	TRUE		Dscc1
Sh3bp1	49.39024	3.282101327	0.69991	4.68929	2.74E-06	2.03E-05	TRUE		Sh3bp1
Adamts20	69.17959	2.430965033	0.45046	5.39667	6.79E-08	7.20E-07	TRUE		Adamts20
Naga	367.2399	1.528936844	0.33893	4.51101	6.45E-06	4.37E-05	TRUE		Naga
Xrcc6	429.0317	1.432305816	0.25678	5.578	2.43E-08	2.82E-07	TRUE		Xrcc6
Pmm1	461.0056	1.737543729	0.27121	6.40655	1.49E-10	2.46E-09	TRUE		Pmm1
Col2a1	3218.439	2.393992001	0.2377	10.0714	7.39E-24	5.64E-22	TRUE		Col2a1
Pde1b	2334.804	1.849913623	0.16985	10.8915	1.27E-27	1.37E-25	TRUE		Pde1b
Ppp1r1a	852.0008	1.441377605	0.23386	6.16342	7.12E-10	1.07E-08	TRUE		Ppp1r1a
Litaf	520.8491	1.651674714	0.26416	6.25257	4.04E-10	6.30E-09	TRUE		Litaf
Bcl6	25.52638	4.221680923	0.65483	6.44702	1.14E-10	1.91E-09	TRUE		Bcl6
Hes1	594.7786	3.686598843	0.25682	14.3547	9.95E-47	5.19E-44	TRUE		Hes1
Tmem44	2117.573	1.365293637	0.2783	4.90585	9.30E-07	7.69E-06	TRUE		Tmem44
Lsg1	807.0484	1.013556243	0.23997	4.2237	2.40E-05	0.00014	TRUE		Lsg1
Eef2kmt	257.9723	2.433895584	0.50281	4.84058	1.29E-06	1.04E-05	TRUE		Eef2kmt
Gpt	532.0371	1.527348192	0.33762	4.52393	6.07E-06	4.14E-05	TRUE		Gpt
Adck5	815.7316	1.524655125	0.32567	4.68161	2.85E-06	2.10E-05	TRUE		Adck5
Hgh1	483.6669	1.042264765	0.29981	3.47639	0.00051	0.00212	TRUE		Hgh1
Dgat1	960.4107	1.091559705	0.29782	3.66519	0.00025	0.00113	TRUE		Dgat1
Hsf1	1061.279	1.114130371	0.27291	4.08237	4.46E-05	0.00025	TRUE		Hsf1
Fbxl6	1324.047	1.573713206	0.30115	5.22572	1.73E-07	1.68E-06	TRUE		Fbxl6
Gpaa1	1532.552	1.700502989	0.31363	5.42192	5.90E-08	6.34E-07	TRUE		Gpaa1
Oplah	182.3419	1.191317483	0.39602	3.00821	0.00263	0.00897	TRUE		Oplah
Tsta3	1327.813	1.889824897	0.37183	5.08254	3.72E-07	3.36E-06	TRUE		Tsta3
Naprt	101.3324	2.300778206	0.61996	3.71117	0.00021	0.00096	TRUE		Naprt
Gsdmd	118.1467	3.026950793	0.44775	6.76042	1.38E-11	2.65E-10	TRUE		Gsdmd
Rhpn1	604.43	1.345108691	0.44859	2.99855	0.00271	0.00923	TRUE		Rhpn1
Arc	41.21227	1.457990617	0.51587	2.8263	0.00471	0.01474	TRUE		Arc
Cep97	951.2472	1.142500918	0.21871	5.22386	1.75E-07	1.70E-06	TRUE		Cep97
Lmf2	982.1493	1.647580684	0.20521	8.02885	9.84E-16	3.23E-14	TRUE		Lmf2
Chkb	149.1221	2.63609339	0.63221	4.16967	3.05E-05	0.00018	TRUE		Chkb
Zcrb1	1388.099	1.195407447	0.30315	3.94324	8.04E-05	0.00042	TRUE		Zcrb1
Alcam	589.5646	4.257283913	0.26186	16.2582	1.96E-59	1.70E-56	TRUE		Alcam
Dubr	221.4291	1.289372446	0.30974	4.16277	3.14E-05	0.00018	TRUE		Dubr
Bbx	909.4589	2.022539177	0.32981	6.13238	8.66E-10	1.28E-08	TRUE		Bbx
Nectin3	604.209	1.148152098	0.25149	4.56532	4.99E-06	3.47E-05	TRUE		Nectin3
Ccdc80	124.5844	3.303670168	0.3093	10.6812	1.25E-26	1.22E-24	TRUE		Ccdc80
Prkdc	424.4796	1.397097718	0.27921	5.00368	5.62E-07	4.90E-06	TRUE		Prkdc
Nde1	775.7302	1.078414158	0.2529	4.26421	2.01E-05	0.00012	TRUE		Nde1
Boc	1263.587	3.006570078	0.26238	11.4589	2.12E-30	3.35E-28	TRUE		Boc
Ccdc191	472.863	2.73037994	0.42078	6.4889	8.65E-11	1.46E-09	TRUE		Ccdc191
Zbtb20	4739.793	1.045284521	0.31771	3.29006	0.001	0.00386	TRUE		Zbtb20
Pmm2	731.4719	1.164804488	0.32553	3.57815	0.00035	0.00151	TRUE		Pmm2
Tmem30c	27.13054	1.872861438	0.47474	3.94506	7.98E-05	0.00041	TRUE		Tmem30c
Dlg1	581.2126	1.618652479	0.23972	6.7524	1.45E-11	2.80E-10	TRUE		Dlg1
Igsf11	142.15	3.567906352	0.31724	11.2468	2.40E-29	3.34E-27	TRUE		Igsf11
Tnk2	2998.556	1.747322117	0.23693	7.37492	1.64E-13	4.07E-12	TRUE		Tnk2
Fstl1	1932.791	1.04246222	0.37713	2.76422	0.00571	0.01738	TRUE		Fstl1
Hgd	11.62296	2.943067564	0.76354	3.85449	0.00012	0.00058	TRUE		Hgd
Ccdc14	226.7968	1.384120396	0.28444	4.86607	1.14E-06	9.22E-06	TRUE		Ccdc14
Clcn2	1119.862	1.012595754	0.31015	3.26481	0.0011	0.00417	TRUE		Clcn2
Dgkg	9.592363	8.086381952	1.5116	5.34954	8.82E-08	9.11E-07	TRUE		Dgkg
Masp1	1412.806	2.997329977	0.22172	13.5187	1.21E-41	4.08E-39	TRUE		Masp1
Adamts1	29.81617	2.271727838	0.7192	3.15869	0.00158	0.00576	TRUE		Adamts1
Slc15a2	533.7385	3.769572929	0.47982	7.85625	3.96E-15	1.22E-13	TRUE		Slc15a2
Parp9	35.33813	2.445218255	0.5481	4.46124	8.15E-06	5.40E-05	TRUE		Parp9
Chaf1b	546.1292	1.718386244	0.25314	6.78839	1.13E-11	2.22E-10	TRUE		Chaf1b
Son	22708.78	1.828318354	0.38005	4.81069	1.50E-06	1.18E-05	TRUE		Son
Lmbr1l	1917.661	1.277319009	0.35657	3.58228	0.00034	0.00149	TRUE		Lmbr1l
Prpf40b	3019.641	1.241254103	0.30348	4.09001	4.31E-05	0.00024	TRUE		Prpf40b
Csad	556.6255	1.219822794	0.26511	4.60111	4.20E-06	2.97E-05	TRUE		Csad
Amhr2	18.79955	2.249812973	0.76048	2.95843	0.00309	0.0103	TRUE		Amhr2
Npff	50.80497	2.185188591	0.39189	5.57609	2.46E-08	2.85E-07	TRUE		Npff
Rttn	233.6888	1.882533277	0.26346	7.14549	8.97E-13	2.03E-11	TRUE		Rttn
Cep89	1289.759	2.119965274	0.38588	5.49383	3.93E-08	4.36E-07	TRUE		Cep89
Msrb2	51.1954	2.491677963	0.41849	5.95394	2.62E-09	3.55E-08	TRUE		Msrb2
Rfc2	1581.698	1.174263348	0.22974	5.11122	3.20E-07	2.92E-06	TRUE		Rfc2
Gps2	1468.183	1.843169429	0.35313	5.21952	1.79E-07	1.73E-06	TRUE		Gps2
P3h3	2034.922	1.902444567	0.35498	5.35927	8.36E-08	8.69E-07	TRUE		P3h3

Serinc2	1699.069	1.276478314	0.28365	4.50019	6.79E-06	4.58E-05	TRUE	Serinc2
Kcnk5	96.33762	2.756645229	0.34334	8.02882	9.84E-16	3.23E-14	TRUE	Kcnk5
Acy1	226.9143	1.036724394	0.2506	4.13694	3.52E-05	0.0002	TRUE	Acy1
Mx2	6.746357	3.295678948	1.19221	2.76434	0.0057	0.01737	TRUE	Mx2
Trip6	559.0357	2.222645702	0.25369	8.76126	1.93E-18	8.35E-17	TRUE	Trip6
Slc17a9	27.41272	1.880179676	0.64517	2.91424	0.00357	0.01165	TRUE	Slc17a9
Nfatc4	880.7512	2.461951391	0.22926	10.7388	6.69E-27	6.87E-25	TRUE	Nfatc4
Gnb3	33.22035	2.106347491	0.7744	2.71997	0.00653	0.01944	TRUE	Gnb3
Prph	50.35562	5.181176325	0.66949	7.73902	1.00E-14	2.92E-13	TRUE	Prph
Cdca3	1344.961	1.972493191	0.18418	10.7094	9.19E-27	9.21E-25	TRUE	Cdca3
Sfi1	741.1706	1.060214792	0.33824	3.13451	0.00172	0.0062	TRUE	Sfi1
Tiam2	547.6429	4.905865603	0.51869	9.45815	3.13E-21	1.85E-19	TRUE	Tiam2
Rsph3b	167.7548	1.59462141	0.3815	4.17985	2.92E-05	0.00017	TRUE	Rsph3b
Tnfrsf12a	31.80136	2.017597687	0.64595	3.12344	0.00179	0.0064	TRUE	Tnfrsf12a
Cldn6	3.184273	5.607739025	1.96097	2.85968	0.00424	0.01349	TRUE	Cldn6
Pkmyt1	746.9388	2.409946435	0.31975	7.53686	4.81E-14	1.27E-12	TRUE	Pkmyt1
Slc25a27	1505.672	1.073995911	0.23914	4.4911	7.09E-06	4.75E-05	TRUE	Slc25a27
Mep1a	4.316384	6.766382149	1.75373	3.85828	0.00011	0.00057	TRUE	Mep1a
Spats1	41.73229	1.592761968	0.49047	3.24744	0.00116	0.00441	TRUE	Spats1
Aars2	702.3391	1.455248603	0.27194	5.35127	8.73E-08	9.04E-07	TRUE	Aars2
Sgo1	277.6448	1.437157011	0.28053	5.12302	3.01E-07	2.76E-06	TRUE	Sgo1
Slc29a1	350.493	2.30089248	0.29884	7.69953	1.37E-14	3.91E-13	TRUE	Slc29a1
Enpp4	57.78677	2.898405318	0.5912	4.90255	9.46E-07	7.81E-06	TRUE	Enpp4
Rrp36	516.2109	1.373171222	0.22533	6.09406	1.10E-09	1.59E-08	TRUE	Rrp36
Tfeb	21.41722	2.196601396	0.63777	3.44418	0.00057	0.00236	TRUE	Tfeb
Foxp4	4733.984	3.002361178	0.25188	11.9197	9.35E-33	1.79E-30	TRUE	Foxp4
Brd4	5823.668	1.355458144	0.34572	3.92073	8.83E-05	0.00045	TRUE	Brd4
Cbs	96.43837	3.997343664	0.3513	11.3788	5.33E-30	7.88E-28	TRUE	Cbs
Myom1	42.05055	1.724344489	0.42054	4.10032	4.13E-05	0.00023	TRUE	Myom1
Cyp4f13	187.6192	2.431749904	0.48945	4.96837	6.75E-07	5.78E-06	TRUE	Cyp4f13
Ndc80	355.6187	1.814233193	0.26927	6.73748	1.61E-11	3.09E-10	TRUE	Ndc80
Clip4	400.0718	2.529629933	0.28602	8.84421	9.22E-19	4.15E-17	TRUE	Clip4
Prkd3	535.5579	1.153475037	0.25782	4.47392	7.68E-06	5.12E-05	TRUE	Prkd3
Vit	193.2194	1.872020206	0.33815	5.53611	3.09E-08	3.51E-07	TRUE	Vit
Eif2ak2	173.9967	1.770781784	0.28956	6.11533	9.64E-10	1.41E-08	TRUE	Eif2ak2
Prss41	10.64314	4.92808828	1.27027	3.87957	0.0001	0.00053	TRUE	Prss41
Slc3a1	27.88456	1.348406041	0.54617	2.46884	0.01356	0.03597	TRUE	Slc3a1
Dnase1l2	303.1504	1.644025278	0.4345	3.78371	0.00015	0.00074	TRUE	Dnase1l2
E4f1	1153.705	1.3474252	0.3263	4.12946	3.64E-05	0.00021	TRUE	E4f1
Ift140	625.1501	1.080299258	0.24599	4.39172	1.12E-05	7.22E-05	TRUE	Ift140
Telo2	1218.281	1.548547712	0.35743	4.3325	1.47E-05	9.24E-05	TRUE	Telo2
Sox8	168.4164	3.650955814	0.28615	12.7589	2.78E-37	7.07E-35	TRUE	Sox8
Nme4	208.4753	1.507898228	0.27745	5.43483	5.48E-08	5.95E-07	TRUE	Nme4
Mrpl28	1070.761	1.09201341	0.29136	3.74799	0.00018	0.00085	TRUE	Mrpl28
Plin3	198.7568	1.119294098	0.34443	3.24974	0.00116	0.00437	TRUE	Plin3
Pdzph1	21.44495	2.576275686	0.66298	3.88591	0.0001	0.00052	TRUE	Pdzph1
Tmem178	274.0669	1.237743428	0.24967	4.95754	7.14E-07	6.08E-06	TRUE	Tmem178
Thumpd2	142.6805	1.679375976	0.37552	4.47209	7.75E-06	5.16E-05	TRUE	Thumpd2
Pkdcc	761.5724	3.506762386	0.20871	16.8018	2.37E-63	2.47E-60	TRUE	Pkdcc
Rock1	1236.548	1.281963856	0.25576	5.01232	5.38E-07	4.71E-06	TRUE	Rock1
Cyp4f41-ps	25.44846	1.894540439	0.62559	3.0284	0.00246	0.00847	TRUE	Cyp4f41-ps
Adamts10	2329.162	1.519415785	0.26493	5.73511	9.75E-09	1.21E-07	TRUE	Adamts10
Kifc5b	671.4171	2.789755575	0.29626	9.41658	4.66E-21	2.69E-19	TRUE	Kifc5b
Apom	97.88164	2.491027227	0.54236	4.59295	4.37E-06	3.08E-05	TRUE	Apom
Psors1c2	33.9702	3.128054911	0.76245	4.10262	4.08E-05	0.00023	TRUE	Psors1c2
Dhx16	1135.61	1.164270758	0.24115	4.82796	1.38E-06	1.10E-05	TRUE	Dhx16
Rpp21	310.1727	1.121380307	0.38523	2.91096	0.0036	0.01175	TRUE	Rpp21
H2-M5	834.5868	1.931131485	0.36639	5.27064	1.36E-07	1.35E-06	TRUE	H2-M5
Ankhd1	3832.465	2.600060234	0.39654	6.5568	5.50E-11	9.68E-10	TRUE	Ankhd1
Impa2	69.20368	2.800272609	0.34895	8.02497	1.02E-15	3.33E-14	TRUE	Impa2
Psmg2	382.2752	1.350673697	0.22306	6.05515	1.40E-09	1.99E-08	TRUE	Psmg2
Sall3	285.4403	1.630689292	0.48799	3.34163	0.00083	0.00329	TRUE	Sall3
Atp9b	1565.266	1.292794871	0.30532	4.2342	2.29E-05	0.00014	TRUE	Atp9b
Megf10	397.2823	2.25055298	0.26189	8.59357	8.43E-18	3.45E-16	TRUE	Megf10
Tcof1	2162.47	2.377487484	0.43107	5.51535	3.48E-08	3.90E-07	TRUE	Tcof1
Asrgl1	204.9676	1.332453027	0.29396	4.53272	5.82E-06	3.99E-05	TRUE	Asrgl1
Incenp	2328.064	2.20839258	0.19992	11.0466	2.28E-28	2.74E-26	TRUE	Incenp
Lpxn	25.2356	1.720212704	0.684	2.51494	0.01191	0.0323	TRUE	Lpxn
Gna14	6.550176	5.651402106	1.42432	3.96778	7.25E-05	0.00038	TRUE	Gna14
Atl3	381.6149	1.218174436	0.21128	5.76564	8.13E-09	1.02E-07	TRUE	Atl3
Cdca5	301.9606	1.283733558	0.43618	2.94313	0.00325	0.01072	TRUE	Cdca5
Tnfrsf25	57.26384	1.769262	0.70423	2.51234	0.01199	0.03249	TRUE	Tnfrsf25
Kif20b	437.5698	1.900215034	0.26807	7.0884	1.36E-12	3.01E-11	TRUE	Kif20b
Pcgf5	107.9857	2.342813934	0.60474	3.87411	0.00011	0.00054	TRUE	Pcgf5
Tjp2	281.7593	1.853906221	0.265	6.99586	2.64E-12	5.66E-11	TRUE	Tjp2

Rad9a	484.3771	1.963096694	0.2577	7.61773	2.58E-14	7.11E-13	TRUE	Rad9a
Rps6kb2	1330.218	1.684465959	0.35019	4.81016	1.51E-06	1.19E-05	TRUE	Rps6kb2
Pola2	759.3141	2.031212845	0.21777	9.32733	1.09E-20	6.12E-19	TRUE	Pola2
Chka	1389.73	1.197688885	0.24052	4.97955	6.37E-07	5.48E-06	TRUE	Chka
Doc2g	475.2288	3.152256233	0.53522	5.88961	3.87E-09	5.13E-08	TRUE	Doc2g
Rce1	545.6725	1.066578582	0.35528	3.0021	0.00268	0.00914	TRUE	Rce1
Slc29a2	259.6519	1.721235394	0.26504	6.49417	8.35E-11	1.43E-09	TRUE	Slc29a2
Efemp2	304.368	1.661648729	0.28868	5.75593	8.62E-09	1.08E-07	TRUE	Efemp2
Lrp5	238.9998	1.918823563	0.33697	5.69432	1.24E-08	1.52E-07	TRUE	Lrp5
Rnaseh2c	572.8809	1.127631068	0.22056	5.11261	3.18E-07	2.90E-06	TRUE	Rnaseh2c
Ehbp1l1	596.5101	1.295200467	0.37263	3.47586	0.00051	0.00212	TRUE	Ehbp1l1
Fam89b	4.51763	2.799907668	1.18343	2.36592	0.01799	0.04547	TRUE	Fam89b
Ltbp3	2879.146	3.741379261	0.25015	14.9566	1.41E-50	8.91E-48	TRUE	Ltbp3
Vegfb	482.1853	1.524086552	0.22517	6.76864	1.30E-11	2.52E-10	TRUE	Vegfb
Fermt3	205.471	1.809765511	0.26146	6.92167	4.46E-12	9.25E-11	TRUE	Fermt3
Rcor2	6748.857	1.137261693	0.26516	4.289	1.79E-05	0.00011	TRUE	Rcor2
Mark2	3612.909	1.481139664	0.40674	3.6415	0.00027	0.00122	TRUE	Mark2
Acsl5	104.1402	1.131230557	0.29911	3.78195	0.00016	0.00075	TRUE	Acsl5
Cep55	337.7413	1.584446686	0.33502	4.72939	2.25E-06	1.70E-05	TRUE	Cep55
Plce1	681.7463	4.194342951	0.22314	18.7966	8.05E-79	2.10E-75	TRUE	Plce1
Hells	509.3176	1.774763852	0.29519	6.01221	1.83E-09	2.54E-08	TRUE	Hells
Col17a1	14.27234	2.192459958	0.76101	2.88097	0.00396	0.01273	TRUE	Col17a1
Pcyt2	3263.784	1.918517197	0.39596	4.84529	1.26E-06	1.01E-05	TRUE	Pcyt2
Sirt7	1089.116	1.372904314	0.33662	4.07845	4.53E-05	0.00025	TRUE	Sirt7
Cenpx	411.1504	1.054209864	0.21004	5.019	5.19E-07	4.56E-06	TRUE	Cenpx
Lrrc45	2988.626	1.83548756	0.37859	4.84825	1.25E-06	1.00E-05	TRUE	Lrrc45
Dus1l	760.3095	1.364609881	0.28832	4.73305	2.21E-06	1.67E-05	TRUE	Dus1l
Mms19	1013.841	1.160951472	0.29773	3.89932	9.65E-05	0.00049	TRUE	Mms19
Hoga1	26.94281	1.737035806	0.47856	3.62973	0.00028	0.00127	TRUE	Hoga1
Pi4k2a	1290.229	1.090313651	0.21707	5.02279	5.09E-07	4.48E-06	TRUE	Pi4k2a
Twnk	597.5206	1.160603935	0.23782	4.88019	1.06E-06	8.66E-06	TRUE	Twnk
Poll	443.3812	1.6069071	0.27713	5.79836	6.70E-09	8.61E-08	TRUE	Poll
Nfkb2	58.02836	1.948757663	0.45443	4.2884	1.80E-05	0.00011	TRUE	Nfkb2
Limd1	917.5342	1.341006881	0.24171	5.54797	2.89E-08	3.30E-07	TRUE	Limd1
Pfkfb1	54.70056	1.630037495	0.62196	2.6208	0.00877	0.02496	TRUE	Pfkfb1
Flnb	1092.853	1.734989444	0.24275	7.14729	8.85E-13	2.01E-11	TRUE	Flnb
Banp	1751.292	1.336407525	0.39275	3.40267	0.00067	0.0027	TRUE	Banp
Atp10a	147.0531	1.398702701	0.38589	3.62461	0.00029	0.0013	TRUE	Atp10a
Rdh5	90.94797	1.704840531	0.36981	4.61007	4.03E-06	2.86E-05	TRUE	Rdh5
Cdk2	649.1478	2.056020995	0.30318	6.78154	1.19E-11	2.32E-10	TRUE	Cdk2
Baiap2	339.8767	1.401941916	0.41356	3.38994	0.0007	0.00281	TRUE	Baiap2
Tepsin	998.8225	1.650322093	0.36202	4.5586	5.15E-06	3.57E-05	TRUE	Tepsin
Prim1	825.811	2.203879035	0.25252	8.72758	2.60E-18	1.12E-16	TRUE	Prim1
Myo1a	116.4211	2.491450596	0.47919	5.19934	2.00E-07	1.91E-06	TRUE	Myo1a
Shmt2	1092.767	2.020922832	0.1756	11.509	1.19E-30	1.94E-28	TRUE	Shmt2
Ddit3	361.3206	2.01167145	0.40222	5.00143	5.69E-07	4.95E-06	TRUE	Ddit3
St8sia5	29.05028	2.414487226	0.59031	4.09021	4.31E-05	0.00024	TRUE	St8sia5
Atp23	59.68873	1.009412389	0.38577	2.61664	0.00888	0.02522	TRUE	Atp23
Fuom	98.62529	1.254485051	0.41428	3.02808	0.00246	0.00848	TRUE	Fuom
Adam8	443.9991	1.61243954	0.33616	4.79666	1.61E-06	1.26E-05	TRUE	Adam8
Lmntd2	176.939	1.490673049	0.37587	3.96595	7.31E-05	0.00038	TRUE	Lmntd2
Pidd1	690.749	2.348224683	0.28048	8.37225	5.65E-17	2.12E-15	TRUE	Pidd1
Pnpla2	231.6197	1.002395399	0.34019	2.94661	0.00321	0.01062	TRUE	Pnpla2
Farp1	1363.493	1.263205703	0.24051	5.25218	1.50E-07	1.48E-06	TRUE	Farp1
Tk1	1039.843	2.540723482	0.1809	14.0452	8.25E-45	3.66E-42	TRUE	Tk1
Rbfox3	2435.975	1.830966852	0.39429	4.64372	3.42E-06	2.48E-05	TRUE	Rbfox3
Ccdc51	158.673	1.300023995	0.26699	4.86915	1.12E-06	9.10E-06	TRUE	Ccdc51
Col7a1	133.7616	1.89368938	0.36894	5.13276	2.86E-07	2.64E-06	TRUE	Col7a1
Mcrip2	44.21468	1.596869944	0.47331	3.37381	0.00074	0.00296	TRUE	Mcrip2
Wdr24	929.0058	1.306587252	0.35116	3.72076	0.0002	0.00093	TRUE	Wdr24
Tyms	1166.54	2.252562036	0.21457	10.4979	8.83E-26	7.96E-24	TRUE	Tyms
Larp1b	120.9835	1.198186597	0.47541	2.52033	0.01172	0.03187	TRUE	Larp1b
Jade1	419.4623	1.234656903	0.30486	4.04991	5.12E-05	0.00028	TRUE	Jade1
Taf3	840.2554	1.671091881	0.36716	4.55134	5.33E-06	3.68E-05	TRUE	Taf3
Clec3b	25.37333	1.545780594	0.61724	2.50434	0.01227	0.03312	TRUE	Clec3b
Exosc7	392.3665	1.269832859	0.21585	5.8829	4.03E-09	5.32E-08	TRUE	Exosc7
Slc25a10	490.2004	1.338199043	0.26597	5.03133	4.87E-07	4.31E-06	TRUE	Slc25a10
Nrp1	1176.767	1.62290861	0.29674	5.46911	4.52E-08	4.98E-07	TRUE	Nrp1
Nudt5	492.2342	1.002859713	0.25008	4.01014	6.07E-05	0.00033	TRUE	Nudt5
Pdgfa	274.9329	1.574344963	0.60853	2.58712	0.00968	0.02714	TRUE	Pdgfa
Uimc1	528.6157	1.339670919	0.27079	4.9472	7.53E-07	6.36E-06	TRUE	Uimc1
Myo5b	8.875827	2.421565986	0.92313	2.62322	0.00871	0.02483	TRUE	Myo5b
Cwf19l2	1608.741	2.701855146	0.40321	6.70087	2.07E-11	3.91E-10	TRUE	Cwf19l2
Mybl1	182.0534	1.275623049	0.33869	3.7663	0.00017	0.00079	TRUE	Mybl1
Terf1	261.2763	1.209253711	0.2619	4.61716	3.89E-06	2.78E-05	TRUE	Terf1

Fastkd2	337.8749	1.521711993	0.37528	4.05488	5.02E-05	0.00027	TRUE	Fastkd2
Boll	8.61678	4.091437529	0.99036	4.13125	3.61E-05	0.0002	TRUE	Boll
Ccdc150	22.19636	2.008664171	0.70624	2.84417	0.00445	0.01403	TRUE	Ccdc150
Nop58	1425.067	1.053431622	0.20019	5.26218	1.42E-07	1.41E-06	TRUE	Nop58
Casp8	40.52213	1.333202725	0.44969	2.96474	0.00303	0.01013	TRUE	Casp8
Ercc5	1209.795	1.08122372	0.23724	4.55754	5.18E-06	3.58E-05	TRUE	Ercc5
Eif5b	4903.154	1.961581381	0.37249	5.26615	1.39E-07	1.38E-06	TRUE	Eif5b
Prim2	290.3116	1.230527448	0.24888	4.94428	7.64E-07	6.45E-06	TRUE	Prim2
Smap1	674.6047	1.74965857	0.3849	4.54578	5.47E-06	3.77E-05	TRUE	Smap1
Rnf25	902.2876	1.49811096	0.33864	4.42394	9.69E-06	6.31E-05	TRUE	Rnf25
Ctdsp1	473.0769	2.014421174	0.25947	7.76364	8.25E-15	2.44E-13	TRUE	Ctdsp1
Fn1	1000.063	2.228742025	0.1988	11.2111	3.60E-29	4.95E-27	TRUE	Fn1
Bard1	181.6568	1.831841032	0.28731	6.3758	1.82E-10	2.96E-09	TRUE	Bard1
Ankzf1	660.7411	1.628021984	0.31291	5.20288	1.96E-07	1.88E-06	TRUE	Ankzf1
Tuba4a	104.4459	4.07291768	0.46889	8.68629	3.74E-18	1.58E-16	TRUE	Tuba4a
Spata3	4.641212	6.178019911	1.77257	3.48534	0.00049	0.00206	TRUE	Spata3
810459M11R	170.3317	5.661336224	0.58494	9.67854	3.72E-22	2.35E-20	TRUE	2810459M11Rik
Rnpepl1	1266.811	1.174270525	0.27201	4.31706	1.58E-05	9.83E-05	TRUE	Rnpepl1
Pask	301.0577	1.590543933	0.2592	6.13646	8.44E-10	1.25E-08	TRUE	Pask
Dtymk	1455.048	1.57753867	0.19492	8.09331	5.81E-16	1.95E-14	TRUE	Dtymk
Gin1	223.5437	1.653021695	0.31263	5.28753	1.24E-07	1.25E-06	TRUE	Gin1
Lct	38.24082	3.196537317	0.62151	5.14315	2.70E-07	2.51E-06	TRUE	Lct
Mcm6	1076.242	1.095758447	0.30304	3.61586	0.0003	0.00134	TRUE	Mcm6
Tsn	3996.011	1.133643205	0.31005	3.6563	0.00026	0.00117	TRUE	Tsn
Dbi	915.126	1.719264066	0.34518	4.98083	6.33E-07	5.45E-06	TRUE	Dbi
Csrp1	262.782	1.380849016	0.32323	4.27198	1.94E-05	0.00012	TRUE	Csrp1
Ube2t	146.3349	1.452315762	0.3266	4.44676	8.72E-06	5.75E-05	TRUE	Ube2t
Rab29	31.91448	1.557273151	0.51179	3.04281	0.00234	0.00814	TRUE	Rab29
Tor1aip1	394.9312	1.278199763	0.29121	4.38925	1.14E-05	7.29E-05	TRUE	Tor1aip1
Kif26b	657.4173	1.447005917	0.2828	5.11663	3.11E-07	2.85E-06	TRUE	Kif26b
Cnih3	77.54104	2.944987543	0.42688	6.89888	5.24E-12	1.07E-10	TRUE	Cnih3
Dusp23	80.88677	1.616798267	0.52974	3.05206	0.00227	0.00792	TRUE	Dusp23
Dcaf8	2426.923	1.120214941	0.18327	6.11253	9.81E-10	1.44E-08	TRUE	Dcaf8
Vangl2	4374.629	1.381051023	0.29536	4.67587	2.93E-06	2.16E-05	TRUE	Vangl2
Prrx1	124.6814	3.953430019	0.29551	13.3782	8.11E-41	2.52E-38	TRUE	Prrx1
Sec16b	11.25675	2.61520053	1.047	2.49781	0.0125	0.03362	TRUE	Sec16b
Axdnd1	19.26193	3.723153186	0.85343	4.36257	1.29E-05	8.16E-05	TRUE	Axdnd1
Cenpf	1320.74	1.360404645	0.22191	6.13041	8.77E-10	1.29E-08	TRUE	Cenpf
Slc30a10	1953.72	5.712597579	0.22035	25.925	######	######	TRUE	Slc30a10
Eprs	2832.21	1.2660891	0.29672	4.26695	1.98E-05	0.00012	TRUE	Eprs
Batf3	19.09462	3.388625999	0.73463	4.61269	3.97E-06	2.83E-05	TRUE	Batf3
Tatdn3	177.997	1.218764224	0.33098	3.68233	0.00023	0.00107	TRUE	Tatdn3
Angel2	1530.299	1.035135998	0.23549	4.3957	1.10E-05	7.10E-05	TRUE	Angel2
Traf5	33.38929	3.766613016	0.54594	6.89932	5.23E-12	1.07E-10	TRUE	Traf5
Dusp12	158.3932	1.117759147	0.31589	3.53841	0.0004	0.00173	TRUE	Dusp12
Mcm10	367.8013	1.895835176	0.25446	7.45037	9.31E-14	2.38E-12	TRUE	Mcm10
Nuf2	536.5325	1.394601357	0.21838	6.38601	1.70E-10	2.79E-09	TRUE	Nuf2
Dnajc1	1085.637	2.223114372	0.23096	9.62555	6.24E-22	3.88E-20	TRUE	Dnajc1
Mllt10	1191.163	1.220250113	0.23903	5.10505	3.31E-07	3.01E-06	TRUE	Mllt10
Nek6	828.4731	1.67002069	0.26323	6.34427	2.23E-10	3.58E-09	TRUE	Nek6
Pkn3	281.9726	3.248092102	0.29705	10.9344	7.89E-28	8.75E-26	TRUE	Pkn3
Slc2a8	328.9002	1.067986645	0.39215	2.7234	0.00646	0.01928	TRUE	Slc2a8
Ttf1	567.9617	1.384993658	0.3217	4.3052	1.67E-05	0.0001	TRUE	Ttf1
Ddx31	233.5999	1.476461194	0.26742	5.5211	3.37E-08	3.79E-07	TRUE	Ddx31
Nr4a2	247.5612	2.830131377	0.24771	11.4251	3.13E-30	4.84E-28	TRUE	Nr4a2
Gpd2	236.9305	1.158891863	0.30356	3.81761	0.00013	0.00066	TRUE	Gpd2
Col5a1	180.2775	1.705105349	0.3671	4.64486	3.40E-06	2.47E-05	TRUE	Col5a1
Tor1b	989.7503	1.243652499	0.20047	6.20372	5.51E-10	8.43E-09	TRUE	Tor1b
Phf19	62.61791	2.304446679	0.37517	6.14241	8.13E-10	1.21E-08	TRUE	Phf19
Gca	82.60565	3.184882566	0.55213	5.76834	8.01E-09	1.01E-07	TRUE	Gca
Brd3	8715.26	1.501824774	0.43075	3.48654	0.00049	0.00205	TRUE	Brd3
Agpat2	42.85903	1.386052578	0.56924	2.43494	0.01489	0.03888	TRUE	Agpat2
Entr1	1645.795	1.002672039	0.26602	3.76919	0.00016	0.00079	TRUE	Entr1
Card9	58.11617	1.384838203	0.57088	2.42582	0.01527	0.03972	TRUE	Card9
Nacc2	417.9762	1.826920501	0.29127	6.27218	3.56E-10	5.59E-09	TRUE	Nacc2
Ccdc183	13.36739	1.816666741	0.63869	2.84437	0.00445	0.01403	TRUE	Ccdc183
Mamdc4	497.1895	1.582678706	0.37146	4.26065	2.04E-05	0.00012	TRUE	Mamdc4
Traf2	518.4024	1.278433178	0.24992	5.11538	3.13E-07	2.86E-06	TRUE	Traf2
Neb	41.8627	1.978790899	0.44897	4.40739	1.05E-05	6.77E-05	TRUE	Neb
Sapcd2	859.9734	2.186344744	0.24608	8.88487	6.40E-19	2.93E-17	TRUE	Sapcd2
Dpp7	304.6646	1.585164185	0.32976	4.80696	1.53E-06	1.20E-05	TRUE	Dpp7
Ssna1	1102.912	1.007215855	0.30312	3.32286	0.00089	0.00348	TRUE	Ssna1
Wdsub1	130.3126	2.008766959	0.30037	6.68755	2.27E-11	4.26E-10	TRUE	Wdsub1
Pkp4	1185.932	1.133169526	0.33336	3.3992	0.00068	0.00273	TRUE	Pkp4
Hat1	329.218	1.188873806	0.37189	3.19686	0.00139	0.00514	TRUE	Hat1

Slc43a1	199.0034	2.521799633	0.24105	10.4617	1.30E-25	1.15E-23	TRUE		Slc43a1
Med19	982.1428	1.343814268	0.38566	3.48441	0.00049	0.00206	TRUE		Med19
Kif18a	199.7121	1.180667566	0.3482	3.39081	0.0007	0.00281	TRUE		Kif18a
Lpcat4	316.9901	1.345220559	0.31967	4.20816	2.57E-05	0.00015	TRUE		Lpcat4
Ccdc34	1164.682	1.650775921	0.32834	5.02759	4.97E-07	4.39E-06	TRUE		Ccdc34
Pax6	6715.628	2.04520766	0.28775	7.10765	1.18E-12	2.64E-11	TRUE		Pax6
Nat10	1155.426	1.725608219	0.28326	6.09189	1.12E-09	1.61E-08	TRUE		Nat10
Meis2	3360.281	2.445544789	0.29954	8.16423	3.23E-16	1.12E-14	TRUE		Meis2
Mdk	3049.155	1.671614471	0.29226	5.7197	1.07E-08	1.32E-07	TRUE		Mdk
Pacsin3	429.358	2.602041092	0.1893	13.7457	5.40E-43	2.05E-40	TRUE		Pacsin3
Itpka	14.12198	3.382382296	0.83307	4.06015	4.90E-05	0.00027	TRUE		Itpka
Tyro3	856.3308	1.663256496	0.18595	8.94443	3.74E-19	1.76E-17	TRUE		Tyro3
Rtf1	5171.229	1.364243566	0.37002	3.68693	0.00023	0.00105	TRUE		Rtf1
Rad51	356.0625	1.495319258	0.37239	4.0155	5.93E-05	0.00032	TRUE		Rad51
Spef1	1510.553	1.14413655	0.26992	4.23886	2.25E-05	0.00013	TRUE		Spef1
Knstrn	848.0085	1.571346956	0.19387	8.10511	5.27E-16	1.78E-14	TRUE		Knstrn
Ivd	1777.096	2.263078591	0.27204	8.31887	8.88E-17	3.28E-15	TRUE		Ivd
Fsip1	11.80798	2.564241684	0.8696	2.94878	0.00319	0.01056	TRUE		Fsip1
Mcm8	257.9748	2.233911068	0.25629	8.71619	2.88E-18	1.22E-16	TRUE		Mcm8
Bub1	548.2134	1.592547407	0.23286	6.83912	7.97E-12	1.59E-10	TRUE		Bub1
Fbln7	24.62044	2.45055067	0.52099	4.70366	2.56E-06	1.90E-05	TRUE		Fbln7
Zc3h8	151.3427	1.121291155	0.34068	3.29136	0.001	0.00384	TRUE		Zc3h8
Polr1b	532.0574	1.19470521	0.19889	6.00698	1.89E-09	2.61E-08	TRUE		Polr1b
Nop56	3105.071	1.757425065	0.29161	6.02673	1.67E-09	2.33E-08	TRUE		Nop56
Cpxm1	313.5847	1.526043621	0.39623	3.85141	0.00012	0.00058	TRUE		Cpxm1
Vps16	1685.427	1.584794295	0.31902	4.96773	6.77E-07	5.79E-06	TRUE		Vps16
Mgme1	191.0679	1.45251902	0.26767	5.42656	5.74E-08	6.20E-07	TRUE		Mgme1
Acss1	513.13	3.044853839	0.23782	12.8032	1.57E-37	4.10E-35	TRUE		Acss1
Gins1	483.4207	2.04096452	0.20054	10.1773	2.50E-24	2.02E-22	TRUE		Gins1
Rbck1	2137.263	1.07188292	0.28852	3.71514	0.0002	0.00095	TRUE		Rbck1
Cdk5rap1	138.7872	1.486914557	0.36623	4.06009	4.91E-05	0.00027	TRUE		Cdk5rap1
Snta1	97.42471	1.617196454	0.39515	4.09264	4.26E-05	0.00024	TRUE		Snta1
Necab3	235.4804	2.629340352	0.33283	7.89986	2.79E-15	8.71E-14	TRUE		Necab3
E2f1	1093.126	1.825084703	0.201	9.07988	1.09E-19	5.55E-18	TRUE		E2f1
Fam210b	503.2504	2.130117719	0.46002	4.6305	3.65E-06	2.62E-05	TRUE		Fam210b
Aurka	618.8824	1.828340123	0.2006	9.11445	7.91E-20	4.10E-18	TRUE		Aurka
Pag1	242.7202	1.590197917	0.32392	4.90924	9.14E-07	7.58E-06	TRUE		Pag1
Rbm38	333.2521	1.728653333	0.2231	7.74827	9.32E-15	2.74E-13	TRUE		Rbm38
Stx16	2234.295	1.177531047	0.26515	4.44101	8.95E-06	5.89E-05	TRUE		Stx16
Nfatc2	191.1097	3.504307607	0.30692	11.4176	3.42E-30	5.24E-28	TRUE		Nfatc2
E2f5	246.9288	1.340841447	0.2333	5.7473	9.07E-09	1.13E-07	TRUE		E2f5
Arfgap1	1871.163	1.257467148	0.34331	3.66273	0.00025	0.00114	TRUE		Arfgap1
Zfp639	539.6832	1.109480423	0.2101	5.28082	1.29E-07	1.28E-06	TRUE		Zfp639
Tnik	982.8261	1.094258791	0.32657	3.35073	0.00081	0.00319	TRUE		Tnik
Pld1	25.78238	1.320622896	0.56121	2.35319	0.01861	0.0468	TRUE		Pld1
Trpc4	26.24254	2.240064125	0.52606	4.25822	2.06E-05	0.00012	TRUE		Trpc4
Supt20	1354.934	1.126512321	0.25597	4.40102	1.08E-05	6.95E-05	TRUE		Supt20
Mbnl1	452.928	1.307006312	0.23232	5.62593	1.85E-08	2.19E-07	TRUE		Mbnl1
Ccna1	27.15338	2.497483493	0.58609	4.26123	2.03E-05	0.00012	TRUE		Ccna1
Ccnl1	1349.948	1.188498589	0.21425	5.54734	2.90E-08	3.31E-07	TRUE		Ccnl1
Veph1	77.49665	1.128541945	0.42835	2.63462	0.00842	0.02411	TRUE		Veph1
Ptgfrn	732.948	1.568707582	0.39731	3.94827	7.87E-05	0.00041	TRUE		Ptgfrn
Notch2	785.6338	2.997328802	0.24097	12.4385	1.62E-35	3.74E-33	TRUE		Notch2
Prpf38b	1966.713	1.029272379	0.18701	5.50391	3.71E-08	4.13E-07	TRUE		Prpf38b
Sypl2	163.5274	4.996179579	0.44595	11.2033	3.93E-29	5.35E-27	TRUE		Sypl2
Ddx20	495.2002	1.03470797	0.19049	5.43176	5.58E-08	6.04E-07	TRUE		Ddx20
Crtc2	874.2792	1.349988061	0.25797	5.23318	1.67E-07	1.62E-06	TRUE		Crtc2
Jtb	422.3575	1.184647477	0.20854	5.68065	1.34E-08	1.63E-07	TRUE		Jtb
Nup210l	171.309	1.735411948	0.34676	5.00458	5.60E-07	4.88E-06	TRUE		Nup210l
Slc50a1	401.7005	1.155432866	0.32242	3.58367	0.00034	0.00149	TRUE		Slc50a1
Tmem144	44.06444	3.844080401	0.59636	6.44588	1.15E-10	1.92E-09	TRUE		Tmem144
Vcam1	143.3069	1.94352447	0.41636	4.66786	3.04E-06	2.24E-05	TRUE		Vcam1
Larp7	2265.633	2.008397488	0.45343	4.42935	9.45E-06	6.17E-05	TRUE		Larp7
Tdo2	4.787461	5.2234387	1.923	2.7163	0.0066	0.0196	TRUE		Tdo2
Ctso	43.86028	1.253362942	0.38635	3.24412	0.00118	0.00445	TRUE		Ctso
Pdgfc	48.73798	2.050617819	0.65393	3.13582	0.00171	0.00617	TRUE		Pdgfc
Alpk1	181.4593	1.492147123	0.28585	5.22007	1.79E-07	1.73E-06	TRUE		Alpk1
Efna4	567.045	3.164109581	0.39744	7.96125	1.70E-15	5.47E-14	TRUE		Efna4
Thbs3	1351.923	1.174013699	0.25155	4.6672	3.05E-06	2.24E-05	TRUE		Thbs3
Pmf1	669.2654	1.688776613	0.27641	6.10957	9.99E-10	1.46E-08	TRUE		Pmf1
Iqgap3	543.5639	2.07238683	0.23109	8.96791	3.02E-19	1.45E-17	TRUE		Iqgap3
Gpatch4	559.1182	2.148450501	0.27719	7.75079	9.13E-15	2.69E-13	TRUE		Gpatch4
Fbxw7	1538.453	1.551038855	0.38037	4.07769	4.55E-05	0.00025	TRUE		Fbxw7
Chd1l	270.3393	2.780540568	0.30571	9.09542	9.42E-20	4.84E-18	TRUE		Chd1l
Acp6	207.5325	2.812189774	0.39188	7.1761	7.17E-13	1.64E-11	TRUE		Acp6

Tars2	1006.234	1.639116753	0.29	5.65208	1.59E-08	1.90E-07	TRUE		Tars2
Ecm1	223.8178	1.322232172	0.34321	3.8525	0.00012	0.00058	TRUE		Ecm1
Rorc	55.79332	3.95179107	0.46653	8.47057	2.44E-17	9.48E-16	TRUE		Rorc
Dapp1	46.29596	3.554654786	0.56075	6.33908	2.31E-10	3.69E-09	TRUE		Dapp1
Nfkb1	342.8535	1.035920534	0.24914	4.15806	3.21E-05	0.00018	TRUE		Nfkb1
Depdc1a	171.7679	1.49872343	0.46554	3.21934	0.00128	0.0048	TRUE		Depdc1a
Ccn1	159.8981	3.738827825	0.33902	11.0285	2.79E-28	3.34E-26	TRUE		Ccn1
Gem	23.17879	2.223382548	0.6822	3.25911	0.00112	0.00425	TRUE		Gem
Rmdn1	337.3859	1.612750551	0.32888	4.90375	9.40E-07	7.77E-06	TRUE		Rmdn1
Pnisr	5236.514	1.370938881	0.37164	3.68888	0.00023	0.00104	TRUE		Pnisr
Anp32b	2441.367	1.158833438	0.21006	5.51659	3.46E-08	3.88E-07	TRUE		Anp32b
Tnc	143.005	1.570166325	0.37922	4.14055	3.46E-05	0.0002	TRUE		Tnc
Pappa	50.13065	2.236063314	0.73743	3.03224	0.00243	0.00838	TRUE		Pappa
Wdr31	166.5923	1.003200232	0.32652	3.07244	0.00212	0.00745	TRUE		Wdr31
Bspry	14.19724	2.005289235	0.78434	2.55665	0.01057	0.02919	TRUE		Bspry
Kif24	151.5396	1.123435672	0.29919	3.75491	0.00017	0.00083	TRUE		Kif24
Atp8b5	36.73314	3.434989402	0.62849	5.46544	4.62E-08	5.08E-07	TRUE		Atp8b5
Ccdc107	248.3479	1.308964669	0.21635	6.05023	1.45E-09	2.04E-08	TRUE		Ccdc107
Plpp3	857.7107	2.368425143	0.2783	8.51035	1.73E-17	6.81E-16	TRUE		Plpp3
Itgb3bp	103.4031	1.005457809	0.34887	2.88204	0.00395	0.0127	TRUE		Itgb3bp
Cdkn2c	177.5684	1.714091642	0.50809	3.37357	0.00074	0.00296	TRUE		Cdkn2c
Nfia	3147.758	3.202550377	0.26824	11.9392	7.39E-33	1.43E-30	TRUE		Nfia
Cc2d1b	1357.667	1.669285596	0.34592	4.82559	1.40E-06	1.11E-05	TRUE		Cc2d1b
Orc1	80.60516	2.467258954	0.36774	6.70918	1.96E-11	3.71E-10	TRUE		Orc1
Echdc2	504.5699	3.337877869	0.45768	7.29309	3.03E-13	7.26E-12	TRUE		Echdc2
Edn2	3.980397	5.136106317	1.82668	2.81172	0.00493	0.01532	TRUE		Edn2
Tfap2c	1111.467	9.33546246	0.53861	17.3324	2.68E-67	3.72E-64	TRUE		Tfap2c
P3h1	277.3374	1.072037905	0.28163	3.80654	0.00014	0.00069	TRUE		P3h1
Kif2c	844.3103	1.684765928	0.20272	8.31091	9.50E-17	3.46E-15	TRUE		Kif2c
Mutyh	223.4868	1.675513684	0.26239	6.38556	1.71E-10	2.79E-09	TRUE		Mutyh
Ccdc163	554.2442	2.457759351	0.36034	6.82058	9.07E-12	1.79E-10	TRUE		Ccdc163
Nasp	5581.572	1.72513644	0.20813	8.28867	1.15E-16	4.15E-15	TRUE		Nasp
Rad54l	555.5444	2.653161914	0.2437	10.8871	1.33E-27	1.43E-25	TRUE		Rad54l
Stil	344.8974	1.784857156	0.38569	4.6277	3.70E-06	2.66E-05	TRUE		Stil
Hspg2	599.9367	1.313978113	0.24644	5.33182	9.72E-08	9.98E-07	TRUE		Hspg2
Zcchc17	1266.414	1.471445949	0.3709	3.96719	7.27E-05	0.00038	TRUE		Zcchc17
Adgrb2	1857.457	1.006949506	0.28413	3.54396	0.00039	0.00169	TRUE		Adgrb2
Hpca	81.71754	1.413808428	0.42273	3.34445	0.00082	0.00326	TRUE		Hpca
Hdac1	1545.472	1.103418978	0.1815	6.07942	1.21E-09	1.73E-08	TRUE		Hdac1
Csmd2	2328.012	2.486424363	0.38853	6.39962	1.56E-10	2.57E-09	TRUE		Csmd2
Zfp593	21.21959	1.682944057	0.64213	2.62089	0.00877	0.02495	TRUE		Zfp593
Tekt2	251.9891	2.254114818	0.45113	4.99658	5.84E-07	5.06E-06	TRUE		Tekt2
Sytl1	70.76683	2.323427289	0.59741	3.88917	0.0001	0.00051	TRUE		Sytl1
Cdca8	1102.521	1.321869797	0.22277	5.93386	2.96E-09	3.99E-08	TRUE		Cdca8
Rpa2	590.3675	1.860708926	0.29855	6.23243	4.59E-10	7.10E-09	TRUE		Rpa2
Smpdl3b	12.77564	3.389233178	0.99511	3.40589	0.00066	0.00267	TRUE		Smpdl3b
Ptpru	2098.155	4.920027135	0.24686	19.9304	2.22E-88	1.16E-84	TRUE		Ptpru
Mecr	536.5648	2.472290993	0.42234	5.85375	4.81E-09	6.28E-08	TRUE		Mecr
Necap2	731.4563	1.242656229	0.30918	4.01924	5.84E-05	0.00031	TRUE		Necap2
Xrcc2	214.5157	2.183551684	0.32348	6.75016	1.48E-11	2.84E-10	TRUE		Xrcc2
Zbtb48	821.3229	1.77386381	0.3185	5.56951	2.55E-08	2.95E-07	TRUE		Zbtb48
Per3	100.2857	1.556329234	0.33744	4.61217	3.98E-06	2.83E-05	TRUE		Per3
Fastk	1178.345	1.066947878	0.25022	4.26399	2.01E-05	0.00012	TRUE		Fastk
Slc4a2	562.4715	1.297123697	0.24258	5.34729	8.93E-08	9.22E-07	TRUE		Slc4a2
Kmt2e	5251.702	1.097874557	0.35589	3.0849	0.00204	0.00719	TRUE		Kmt2e
Agtrap	136.1061	2.345426814	0.45136	5.19636	2.03E-07	1.93E-06	TRUE		Agtrap
Miip	2035.652	2.441093143	0.34601	7.05494	1.73E-12	3.80E-11	TRUE		Miip
Morn1	100.952	1.316235154	0.3487	3.77473	0.00016	0.00077	TRUE		Morn1
Pank4	1548.179	1.893083896	0.31943	5.92649	3.09E-09	4.16E-08	TRUE		Pank4
Mib2	1284.239	1.163818364	0.24489	4.75236	2.01E-06	1.53E-05	TRUE		Mib2
Cdk11b	4464.904	2.202851077	0.39098	5.63414	1.76E-08	2.09E-07	TRUE		Cdk11b
Ccnl2	4464.098	1.137827206	0.26382	4.3129	1.61E-05	1.00E-04	TRUE		Ccnl2
Dvl1	2520.421	1.196994634	0.24263	4.93345	8.08E-07	6.78E-06	TRUE		Dvl1
Tnfrsf4	62.7264	1.66564869	0.57448	2.89941	0.00374	0.01212	TRUE		Tnfrsf4
Prom1	699.0718	1.165214422	0.17351	6.7155	1.87E-11	3.56E-10	TRUE		Prom1
Evc	732.394	2.128212481	0.23972	8.87806	6.80E-19	3.10E-17	TRUE		Evc
Fosl2	26.6887	2.188264783	0.90679	2.41321	0.01581	0.04091	TRUE		Fosl2
Cenpa	1158.846	1.481449572	0.20794	7.12432	1.05E-12	2.35E-11	TRUE		Cenpa
Klf3	233.1952	2.796103248	0.41291	6.77177	1.27E-11	2.47E-10	TRUE		Klf3
Zcchc4	513.3063	1.001949946	0.2389	4.19396	2.74E-05	0.00016	TRUE		Zcchc4
Rfc1	616.4159	1.322390187	0.26309	5.02633	5.00E-07	4.41E-06	TRUE		Rfc1
Cckar	51.19238	1.461101296	0.59364	2.46124	0.01385	0.03664	TRUE		Cckar
Lias	878.4618	1.618398804	0.32386	4.99718	5.82E-07	5.05E-06	TRUE		Lias
Epha5	4258.508	3.791789458	0.40677	9.32167	1.15E-20	6.42E-19	TRUE		Epha5
Rest	188.1043	1.151406636	0.42704	2.69624	0.00701	0.02063	TRUE		Rest

Ibsp	4.589752	3.768168612	1.5427	2.44258	0.01458	0.03819	TRUE	Ibsp
Coq2	947.803	2.01910724	0.24804	8.14026	3.94E-16	1.35E-14	TRUE	Coq2
Crybb3	49.68172	3.29542286	0.51576	6.38951	1.66E-10	2.72E-09	TRUE	Crybb3
Shroom3	1151.667	2.24142677	0.38435	5.83176	5.48E-09	7.13E-08	TRUE	Shroom3
Ddx55	810.1284	1.345244067	0.33366	4.03177	5.54E-05	0.0003	TRUE	Ddx55
Arl6ip4	1131.998	1.106669592	0.31121	3.55599	0.00038	0.00163	TRUE	Arl6ip4
Pitpnm2	1074.74	1.376128265	0.1875	7.33919	2.15E-13	5.23E-12	TRUE	Pitpnm2
Abcb9	140.402	2.73099352	0.39542	6.90649	4.97E-12	1.02E-10	TRUE	Abcb9
Kntc1	450.0646	1.761631545	0.21863	8.05777	7.77E-16	2.59E-14	TRUE	Kntc1
Diablo	241.3565	2.059570336	0.40273	5.11402	3.15E-07	2.88E-06	TRUE	Diablo
Sfswap	3755.231	1.358121626	0.29144	4.66006	3.16E-06	2.31E-05	TRUE	Sfswap
Wdr66	37.57936	1.902604509	0.50094	3.79804	0.00015	0.00071	TRUE	Wdr66
Psph	156.8612	1.12923649	0.28975	3.89732	9.73E-05	0.0005	TRUE	Psph
Rhof	131.387	1.449125737	0.41388	3.50134	0.00046	0.00195	TRUE	Rhof
Acad10	98.81821	1.297866619	0.29998	4.32654	1.51E-05	9.46E-05	TRUE	Acad10
Morn3	8.277036	2.682947848	1.11699	2.40194	0.01631	0.04194	TRUE	Morn3
Pus1	288.7742	1.038515598	0.25131	4.13236	3.59E-05	0.0002	TRUE	Pus1
Cit	1182.496	2.318738344	0.28607	8.10542	5.26E-16	1.78E-14	TRUE	Cit
Chek2	488.6011	3.452652145	0.30797	11.2108	3.61E-29	4.95E-27	TRUE	Chek2
Cabp1	1009.144	5.058869316	0.43698	11.577	5.39E-31	8.99E-29	TRUE	Cabp1
Acads	482.0451	1.721400126	0.26088	6.59852	4.15E-11	7.50E-10	TRUE	Acads
Ints1	3146.833	1.350259231	0.32568	4.14592	3.38E-05	0.00019	TRUE	Ints1
Psmg3	288.3995	1.139220402	0.3416	3.33491	0.00085	0.00335	TRUE	Psmg3
Lfng	402.6449	1.964188403	0.31971	6.14374	8.06E-10	1.20E-08	TRUE	Lfng
Ung	286.3383	2.412143748	0.27911	8.64228	5.51E-18	2.28E-16	TRUE	Ung
Oas1b	9.716568	6.219622426	1.5339	4.05477	5.02E-05	0.00027	TRUE	Oas1b
Phf14	6404.49	1.98219934	0.4615	4.29511	1.75E-05	0.00011	TRUE	Phf14
Eln	255.4296	2.611144598	0.24619	10.606	2.79E-26	2.63E-24	TRUE	Eln
Rbm28	1940.399	1.442933202	0.24899	5.79522	6.82E-09	8.73E-08	TRUE	Rbm28
Lrwd1	811.4634	1.140051534	0.33745	3.37846	0.00073	0.00292	TRUE	Lrwd1
Ephb4	447.7495	1.575681493	0.20524	7.67722	1.63E-14	4.60E-13	TRUE	Ephb4
Gigyf1	3610.265	1.338344824	0.27136	4.93202	8.14E-07	6.82E-06	TRUE	Gigyf1
Tsc22d4	737.5235	1.218082939	0.21736	5.6039	2.10E-08	2.46E-07	TRUE	Tsc22d4
Mcm7	2414.687	1.081155413	0.16584	6.51916	7.07E-11	1.22E-09	TRUE	Mcm7
Ccdc136	1515.06	2.67083139	0.33147	8.05743	7.79E-16	2.59E-14	TRUE	Ccdc136
Sspo	34.62685	2.084038125	0.65041	3.20418	0.00135	0.00503	TRUE	Sspo
Herc6	33.22718	3.202969859	0.53339	6.00495	1.91E-09	2.64E-08	TRUE	Herc6
Slc23a4	37.27622	1.71742168	0.56616	3.03347	0.00242	0.00835	TRUE	Slc23a4
Prdm5	151.4782	2.049764539	0.31419	6.52396	6.85E-11	1.19E-09	TRUE	Prdm5
Pradc1	196.3044	1.672502119	0.26935	6.20949	5.32E-10	8.15E-09	TRUE	Pradc1
Adamts9	45.86851	2.304492259	0.65359	3.52591	0.00042	0.0018	TRUE	Adamts9
Ino80b	397.1624	1.002789841	0.25802	3.88646	0.0001	0.00052	TRUE	Ino80b
Gm20696	7.279472	2.898761864	1.09386	2.65003	0.00805	0.02318	TRUE	Gm20696
Abtb1	1124.04	1.538309774	0.38047	4.04316	5.27E-05	0.00029	TRUE	Abtb1
Klf15	54.65866	1.759514115	0.38945	4.51794	6.24E-06	4.24E-05	TRUE	Klf15
Wnt7a	507.7726	3.361858326	0.25227	13.3264	1.63E-40	4.98E-38	TRUE	Wnt7a
Itpr1	219.0948	2.546297617	0.28458	8.94771	3.63E-19	1.72E-17	TRUE	Itpr1
Bhlhe40	47.78893	1.81596046	0.77358	2.34747	0.0189	0.04734	TRUE	Bhlhe40
Lag3	155.0554	1.364740517	0.42283	3.22761	0.00125	0.00468	TRUE	Lag3
Rad52	426.6636	1.133520485	0.33164	3.41797	0.00063	0.00257	TRUE	Rad52
Wnt5b	116.7903	4.263205906	0.31395	13.5792	5.32E-42	1.85E-39	TRUE	Wnt5b
Ccdc77	407.7341	2.101184776	0.25381	8.27856	1.25E-16	4.49E-15	TRUE	Ccdc77
Dusp16	323.1022	2.159531615	0.28898	7.47293	7.84E-14	2.01E-12	TRUE	Dusp16
Ogg1	118.0876	1.071778226	0.26614	4.02707	5.65E-05	0.0003	TRUE	Ogg1
Timp4	144.967	4.136011828	0.55239	7.48742	7.02E-14	1.81E-12	TRUE	Timp4
Cand2	996.9515	1.612504265	0.22442	7.18525	6.71E-13	1.54E-11	TRUE	Cand2
Rad51ap1	419.8844	1.804607381	0.24	7.51908	5.52E-14	1.44E-12	TRUE	Rad51ap1
Gipr	369.3737	3.847017449	0.44794	8.58821	8.83E-18	3.60E-16	TRUE	Gipr
Dmpk	853.1911	1.598745419	0.2408	6.63933	3.15E-11	5.78E-10	TRUE	Dmpk
Ttyh1	2626.038	1.842274298	0.23201	7.94063	2.01E-15	6.40E-14	TRUE	Ttyh1
Tmem238	27.59681	1.771793823	0.50167	3.53176	0.00041	0.00177	TRUE	Tmem238
Rpl28	5672.112	1.106859302	0.28455	3.8898	0.0001	0.00051	TRUE	Rpl28
Csrp3	2.959346	6.490717791	2.1332	3.04272	0.00234	0.00814	TRUE	Csrp3
Snrpa1	643.5384	1.265288284	0.24165	5.23606	1.64E-07	1.60E-06	TRUE	Snrpa1
Pcsk6	27.22975	1.635733539	0.55064	2.97062	0.00297	0.00997	TRUE	Pcsk6
Crtc3	333.8557	1.027279208	0.19654	5.22682	1.72E-07	1.67E-06	TRUE	Crtc3
Rhcg	34.44735	3.813193076	0.82894	4.6001	4.22E-06	2.99E-05	TRUE	Rhcg
Ttc23	296.1714	2.353503562	0.21191	11.106	1.17E-28	1.46E-26	TRUE	Ttc23
Rasgrp4	25.03387	1.629809407	0.54843	2.97175	0.00296	0.00995	TRUE	Rasgrp4
Ryr1	773.0095	3.406660362	0.32758	10.3994	2.49E-25	2.17E-23	TRUE	Ryr1
Fbxo17	53.36497	1.767444433	0.39759	4.44539	8.77E-06	5.79E-05	TRUE	Fbxo17
Mfge8	997.0425	2.838306268	0.35712	7.94769	1.90E-15	6.06E-14	TRUE	Mfge8
Xylt1	203.0401	2.790846889	0.35046	7.96337	1.67E-15	5.38E-14	TRUE	Xylt1
Sox6os	15.86798	3.708626588	0.73341	5.05667	4.27E-07	3.81E-06	TRUE	Sox6os
Mylpf	47.28151	1.573149012	0.58188	2.70359	0.00686	0.02023	TRUE	Mylpf

Kif22	1171.734	1.80368672	0.18343	9.83325	8.10E-23	5.53E-21	TRUE	Kif22
Mvp	209.7782	1.418669693	0.29919	4.74178	2.12E-06	1.60E-05	TRUE	Mvp
Tbx6	53.46456	2.043694543	0.58208	3.51103	0.00045	0.00189	TRUE	Tbx6
Cln3	320.0428	1.335360682	0.3374	3.95775	7.57E-05	0.0004	TRUE	Cln3
Pold3	1196.618	1.517598472	0.19005	7.9854	1.40E-15	4.55E-14	TRUE	Pold3
Syt3	196.0224	1.071037514	0.33222	3.2239	0.00126	0.00473	TRUE	Syt3
Myh14	45.80104	1.43661213	0.50837	2.8259	0.00471	0.01475	TRUE	Myh14
Lat	46.11456	2.406349745	0.57193	4.20741	2.58E-05	0.00015	TRUE	Lat
Arhgap17	642.0861	1.021453459	0.28361	3.60168	0.00032	0.0014	TRUE	Arhgap17
Rbbp6	4753.315	1.811748104	0.35197	5.1475	2.64E-07	2.46E-06	TRUE	Rbbp6
Dkkl1	74.76833	1.906910739	0.56167	3.39509	0.00069	0.00277	TRUE	Dkkl1
Tead2	4233.758	1.898740436	0.17748	10.6982	1.04E-26	1.03E-24	TRUE	Tead2
Cd37	38.32083	1.828614664	0.53151	3.44042	0.00058	0.00239	TRUE	Cd37
Stx4a	456.4906	1.486463	0.24032	6.18537	6.20E-10	9.42E-09	TRUE	Stx4a
Phkg2	726.3532	1.21678581	0.27125	4.48591	7.26E-06	4.86E-05	TRUE	Phkg2
Bcat2	613.2796	1.161931432	0.37169	3.12607	0.00177	0.00635	TRUE	Bcat2
Fgfr2	730.4216	1.781049547	0.19724	9.03006	1.72E-19	8.49E-18	TRUE	Fgfr2
Plk1	640.4481	1.391236445	0.23453	5.93202	2.99E-09	4.04E-08	TRUE	Plk1
Rexo5	185.8136	1.823600533	0.28463	6.40694	1.48E-10	2.46E-09	TRUE	Rexo5
Knop1	3243.254	1.630523872	0.38522	4.23271	2.31E-05	0.00014	TRUE	Knop1
Mki67	4308.501	1.651190812	0.17418	9.47993	2.54E-21	1.51E-19	TRUE	Mki67
Swap70	182.6357	2.047206284	0.28135	7.27649	3.43E-13	8.16E-12	TRUE	Swap70
Wee1	329.9029	1.522218369	0.37757	4.03161	5.54E-05	0.0003	TRUE	Wee1
Rbm10	3018.238	1.299817461	0.30215	4.30195	1.69E-05	0.0001	TRUE	Rbm10
Nadsyn1	191.9234	1.304655664	0.32658	3.99486	6.47E-05	0.00034	TRUE	Nadsyn1
Rbmx2	867.1623	2.380235242	0.43239	5.50477	3.70E-08	4.12E-07	TRUE	Rbmx2
Gpc4	224.5708	4.859651457	0.29192	16.647	3.18E-62	3.16E-59	TRUE	Gpc4
Prickle3	51.59695	1.817228935	0.39093	4.64843	3.34E-06	2.43E-05	TRUE	Prickle3
Plp2	134.6905	1.183499509	0.45357	2.60928	0.00907	0.02568	TRUE	Plp2
Glod5	4.741647	5.880877311	2.48066	2.37069	0.01775	0.04504	TRUE	Glod5
Efnb1	1557.523	1.849244008	0.33761	5.4774	4.32E-08	4.78E-07	TRUE	Efnb1
Awat2	5.401786	3.758599311	1.32797	2.83033	0.00465	0.01459	TRUE	Awat2
Magt1	660.9974	1.69287643	0.20955	8.07845	6.56E-16	2.20E-14	TRUE	Magt1
Hmgn5	1048.233	1.26486964	0.1751	7.22386	5.05E-13	1.18E-11	TRUE	Hmgn5
Cenpi	225.3278	1.693925979	0.25251	6.70848	1.97E-11	3.73E-10	TRUE	Cenpi
Col4a6	39.70689	3.423061565	0.44826	7.63639	2.23E-14	6.20E-13	TRUE	Col4a6
Col4a5	78.18259	2.104208902	0.45904	4.58395	4.56E-06	3.20E-05	TRUE	Col4a5
Phka2	644.58	1.423337427	0.38173	3.72864	0.00019	0.00091	TRUE	Phka2
Slc7a3	30.06475	2.059458094	0.5089	4.04688	5.19E-05	0.00028	TRUE	Slc7a3
Tsx	22.31971	2.152702068	0.66083	3.25757	0.00112	0.00427	TRUE	Tsx
Mtm1	85.24384	1.172808259	0.32988	3.55529	0.00038	0.00163	TRUE	Mtm1
Gabre	128.231	3.729636555	0.31393	11.8803	1.50E-32	2.84E-30	TRUE	Gabre
Vegfd	12.58912	3.065433806	1.04619	2.93011	0.00339	0.01113	TRUE	Vegfd
Naa10	490.3694	1.4902773	0.25716	5.79519	6.82E-09	8.73E-08	TRUE	Naa10
Irak1	1359.751	1.151100012	0.2818	4.0848	4.41E-05	0.00025	TRUE	Irak1
Dkc1	766.4184	1.109854874	0.20123	5.51538	3.48E-08	3.90E-07	TRUE	Dkc1
Atp11a	1267.315	1.220923337	0.17913	6.81568	9.38E-12	1.85E-10	TRUE	Atp11a
Nek3	302.3873	1.451620158	0.38913	3.73042	0.00019	0.0009	TRUE	Nek3
Eif4ebp1	40.64132	1.297616648	0.46237	2.80643	0.00501	0.01555	TRUE	Eif4ebp1
Ankrd10	3342.733	1.166634787	0.20938	5.57192	2.52E-08	2.91E-07	TRUE	Ankrd10
Sfrp1	1468.477	5.778271475	0.37859	15.2626	1.36E-52	9.75E-50	TRUE	Sfrp1
Fgfr1	1237.828	1.309263763	0.16848	7.77101	7.79E-15	2.32E-13	TRUE	Fgfr1
Slc7a2	52.4961	3.63140364	0.41435	8.76404	1.88E-18	8.16E-17	TRUE	Slc7a2
Irf2	111.494	1.014748332	0.29105	3.48654	0.00049	0.00205	TRUE	Irf2
Sall1	1254.367	5.16336064	0.22222	23.2351	######	######	TRUE	Sall1
Gins3	176.9061	1.726886924	0.30804	5.6061	2.07E-08	2.44E-07	TRUE	Gins3
Setd6	318.5971	1.678189673	0.27363	6.13295	8.63E-10	1.27E-08	TRUE	Setd6
Orc6	726.5708	1.073084892	0.21422	5.0093	5.46E-07	4.78E-06	TRUE	Orc6
Gab1	304.8387	1.535855147	0.27443	5.59653	2.19E-08	2.57E-07	TRUE	Gab1
Zfp821	3185.345	1.116292877	0.33901	3.29285	0.00099	0.00382	TRUE	Zfp821
Bbs2	538.0777	1.041753073	0.34803	2.9933	0.00276	0.00936	TRUE	Bbs2
Cenpn	317.8828	1.627947212	0.23994	6.78485	1.16E-11	2.27E-10	TRUE	Cenpn
Mt3	279.9382	3.671049636	0.458	8.01537	1.10E-15	3.59E-14	TRUE	Mt3
Mt1	93.46337	5.644331855	0.45778	12.3298	6.26E-35	1.42E-32	TRUE	Mt1
Nudt7	20.99405	1.877094699	0.77178	2.43216	0.01501	0.03913	TRUE	Nudt7
Slc27a1	2884.637	2.342043781	0.28455	8.23059	1.86E-16	6.61E-15	TRUE	Slc27a1
Mvb12a	581.3034	1.438965414	0.32968	4.36477	1.27E-05	8.08E-05	TRUE	Mvb12a
Gins2	191.2767	1.716443447	0.34521	4.97218	6.62E-07	5.68E-06	TRUE	Gins2
Gse1	456.442	1.368909678	0.36312	3.76984	0.00016	0.00078	TRUE	Gse1
Taf1c	800.4769	1.665404861	0.32504	5.12369	3.00E-07	2.75E-06	TRUE	Taf1c
Ifi30	266.2389	2.693386815	0.43195	6.23539	4.51E-10	6.98E-09	TRUE	Ifi30
Pbx4	411.602	1.171257255	0.27244	4.29912	1.71E-05	0.00011	TRUE	Pbx4
Atp13a1	2659.205	1.442231058	0.30578	4.71658	2.40E-06	1.80E-05	TRUE	Atp13a1
Tradd	78.41709	1.171002475	0.36923	3.17144	0.00152	0.00556	TRUE	Tradd
Dus2	323.836	1.874802398	0.30738	6.09934	1.07E-09	1.55E-08	TRUE	Dus2

Maml2	435.0696	1.727501477	0.37904	4.55756	5.18E-06	3.58E-05	TRUE	Maml2
Taf1d	1489.496	2.257765311	0.32094	7.03485	1.99E-12	4.34E-11	TRUE	Taf1d
Adat1	333.9426	1.627083092	0.2142	7.59617	3.05E-14	8.34E-13	TRUE	Adat1
Ctrb1	5.512739	2.989884009	1.11487	2.68183	0.00732	0.02136	TRUE	Ctrb1
Acad8	470.7305	1.654865207	0.24224	6.83153	8.40E-12	1.67E-10	TRUE	Acad8
Jam3	524.3106	1.231122892	0.20203	6.09389	1.10E-09	1.59E-08	TRUE	Jam3
Usp2	83.50572	1.309914544	0.37686	3.47589	0.00051	0.00212	TRUE	Usp2
Oaf	61.62703	2.319856825	0.48319	4.80108	1.58E-06	1.23E-05	TRUE	Oaf
Tirap	303.8362	2.381468733	0.48346	4.92585	8.40E-07	7.01E-06	TRUE	Tirap
Chek1	224.6402	1.618546751	0.43954	3.68233	0.00023	0.00107	TRUE	Chek1
Vps11	888.5367	1.086845885	0.31756	3.4225	0.00062	0.00253	TRUE	Vps11
Robo3	410.7261	1.659007154	0.30681	5.40721	6.40E-08	6.81E-07	TRUE	Robo3
Mcam	304.4787	1.305045397	0.2646	4.93211	8.13E-07	6.82E-06	TRUE	Mcam
Smarca4	15062.25	1.217095545	0.36893	3.29896	0.00097	0.00375	TRUE	Smarca4
Ldlr	896.406	1.452838373	0.1858	7.8194	5.31E-15	1.62E-13	TRUE	Ldlr
Dock6	646.6736	2.144682352	0.30131	7.11776	1.10E-12	2.47E-11	TRUE	Dock6
Aqp9	4.348124	5.277156121	1.84198	2.86494	0.00417	0.0133	TRUE	Aqp9
Sltm	4727.788	1.658112405	0.3392	4.88834	1.02E-06	8.34E-06	TRUE	Sltm
Mns1	341.8609	2.061969787	0.22743	9.06627	1.23E-19	6.23E-18	TRUE	Mns1
Ice2	217.7234	1.037533118	0.27365	3.79143	0.00015	0.00072	TRUE	Ice2
Rp9	1555.43	1.889429756	0.43257	4.36789	1.25E-05	7.97E-05	TRUE	Rp9
Cln6	129.2484	1.543620956	0.32312	4.7773	1.78E-06	1.37E-05	TRUE	Cln6
Phip	6445.191	1.521655684	0.32988	4.61269	3.97E-06	2.83E-05	TRUE	Phip
Kif23	825.4754	2.010798159	0.20834	9.65148	4.85E-22	3.02E-20	TRUE	Kif23
Bckdhb	189.9304	1.472827648	0.28163	5.22957	1.70E-07	1.65E-06	TRUE	Bckdhb
Zw10	780.3481	1.58919349	0.24699	6.43427	1.24E-10	2.07E-09	TRUE	Zw10
Tmprss5	52.84365	2.176160557	0.60194	3.61525	0.0003	0.00134	TRUE	Tmprss5
Acsbg1	132.0299	3.026329872	0.3698	8.18359	2.76E-16	9.59E-15	TRUE	Acsbg1
Man2c1	1491.151	1.515074002	0.33631	4.50495	6.64E-06	4.48E-05	TRUE	Man2c1
Commd4	1253.815	1.200582675	0.3145	3.81745	0.00013	0.00066	TRUE	Commd4
700017B05R	114.6007	1.916914876	0.33384	5.74202	9.36E-09	1.17E-07	TRUE	1700017B05Rik
Pstpip1	46.23902	1.567187887	0.46564	3.36563	0.00076	0.00304	TRUE	Pstpip1
Lrrc1	444.5673	2.278378525	0.21512	10.5913	3.27E-26	3.04E-24	TRUE	Lrrc1
Car12	27.77282	3.327079717	0.52753	6.30693	2.85E-10	4.51E-09	TRUE	Car12
Plscr4	26.94106	2.665123706	0.50742	5.25234	1.50E-07	1.48E-06	TRUE	Plscr4
Parp16	69.44802	1.213411905	0.46715	2.59748	0.00939	0.02644	TRUE	Parp16
Zwilch	334.1175	2.233625641	0.34379	6.49709	8.19E-11	1.40E-09	TRUE	Zwilch
Rasa2	511.8339	1.159934954	0.33026	3.51219	0.00044	0.00189	TRUE	Rasa2
Cmtm7	121.257	1.528411126	0.32333	4.72716	2.28E-06	1.71E-05	TRUE	Cmtm7
Eomes	4249.007	5.57591234	0.36327	15.3493	3.58E-53	2.66E-50	TRUE	Eomes
Kif9	54.81255	1.003691385	0.41954	2.39239	0.01674	0.04284	TRUE	Kif9
Stac	18.74993	2.113346021	0.90625	2.33197	0.0197	0.04904	TRUE	Stac
Scn5a	116.8014	2.887222012	0.42922	6.72673	1.74E-11	3.31E-10	TRUE	Scn5a
Csrnp1	21.45666	2.170158738	0.60239	3.60259	0.00032	0.0014	TRUE	Csrnp1
Rpsa	29125.44	1.230273961	0.3008	4.08998	4.31E-05	0.00024	TRUE	Rpsa
Vipr1	42.95401	2.30975726	0.66346	3.48138	0.0005	0.00209	TRUE	Vipr1
Cck	8.947065	5.115874026	1.75957	2.90745	0.00364	0.01186	TRUE	Cck
Cep63	626.0529	1.090877869	0.31062	3.51193	0.00044	0.00189	TRUE	Cep63
Trak1	1759.367	1.173189874	0.19098	6.14308	8.09E-10	1.20E-08	TRUE	Trak1
Topbp1	1151.773	1.68367348	0.26531	6.34617	2.21E-10	3.54E-09	TRUE	Topbp1
Uba5	1737.911	1.069506078	0.28232	3.7883	0.00015	0.00073	TRUE	Uba5
Nphp3	124.8244	1.406921703	0.38397	3.66413	0.00025	0.00113	TRUE	Nphp3
Hemk1	404.6789	1.123473495	0.34803	3.22805	0.00125	0.00468	TRUE	Hemk1
Mon1a	634.7481	1.149806389	0.3402	3.37982	0.00073	0.00291	TRUE	Mon1a
Traip	366.8758	1.629335249	0.21707	7.50601	6.10E-14	1.59E-12	TRUE	Traip
Srek1	4155.988	1.891636005	0.35801	5.28379	1.27E-07	1.27E-06	TRUE	Srek1
Fhl3	89.23066	2.147250181	0.343	6.26014	3.85E-10	6.02E-09	TRUE	Fhl3
Pygm	44.16908	2.240043185	0.60777	3.68569	0.00023	0.00105	TRUE	Pygm
Syde1	218.3271	2.217521686	0.30362	7.30352	2.80E-13	6.74E-12	TRUE	Syde1
Inppl1	1269.228	2.763283973	0.2055	13.4469	3.21E-41	1.03E-38	TRUE	Inppl1
Gab3	46.3691	2.616071544	0.91915	2.8462	0.00442	0.01396	TRUE	Gab3
Troap	508.0581	2.247735126	0.23646	9.50596	1.98E-21	1.19E-19	TRUE	Troap
Lama1	316.2492	1.246413246	0.2576	4.83849	1.31E-06	1.04E-05	TRUE	Lama1
Arap1	110.0272	1.189127005	0.39732	2.99289	0.00276	0.00938	TRUE	Arap1
Fanca	418.2698	2.156755383	0.19272	11.191	4.51E-29	6.07E-27	TRUE	Fanca
Inha	383.7633	1.261689079	0.30927	4.07957	4.51E-05	0.00025	TRUE	Inha
Cip2a	616.5232	1.356748678	0.24568	5.52243	3.34E-08	3.77E-07	TRUE	Cip2a
Lmo7	58.8023	3.445703668	0.40947	8.41507	3.93E-17	1.50E-15	TRUE	Lmo7
Tbc1d4	177.1382	2.65777918	0.40181	6.61445	3.73E-11	6.79E-10	TRUE	Tbc1d4
Slc7a6os	816.754	1.015243169	0.19287	5.26379	1.41E-07	1.40E-06	TRUE	Slc7a6os
Ttf2	329.1337	1.328977305	0.30816	4.31262	1.61E-05	0.0001	TRUE	Ttf2
Szt2	2329.239	1.14814501	0.29194	3.93282	8.40E-05	0.00043	TRUE	Szt2
Shf	860.5496	1.264800961	0.30229	4.184	2.86E-05	0.00017	TRUE	Shf
Rtp4	21.52106	2.126216094	0.62149	3.42114	0.00062	0.00254	TRUE	Rtp4
Palmd	105.7316	2.079120596	0.28622	7.26394	3.76E-13	8.89E-12	TRUE	Palmd

Frrs1	180.5297	3.481031268	0.38169	9.12006	7.51E-20	3.92E-18	TRUE		Frrs1
Crlf2	89.84729	1.815219047	0.35039	5.18058	2.21E-07	2.09E-06	TRUE		Crlf2
Larp4b	3074.152	1.488965025	0.33028	4.50814	6.54E-06	4.42E-05	TRUE		Larp4b
Asprv1	29.86047	1.773903219	0.58166	3.04975	0.00229	0.00798	TRUE		Asprv1
Acsm1	4.686974	6.885996706	2.75838	2.49639	0.01255	0.03369	TRUE		Acsm1
Idua	221.8096	1.280465907	0.25311	5.05894	4.22E-07	3.77E-06	TRUE		Idua
Angptl1	134.6172	1.876229312	0.48655	3.85621	0.00012	0.00057	TRUE		Angptl1
Dph5	197.8111	1.149034062	0.23204	4.95196	7.35E-07	6.24E-06	TRUE		Dph5
Reep4	386.0812	1.74522755	0.25498	6.84452	7.67E-12	1.54E-10	TRUE		Reep4
Myo5c	7.18809	2.672749858	1.10764	2.413	0.01582	0.04093	TRUE		Myo5c
Pik3c3	1261.927	2.237855685	0.39321	5.69122	1.26E-08	1.54E-07	TRUE		Pik3c3
Qsox1	624.8486	1.607147718	0.26331	6.10375	1.04E-09	1.51E-08	TRUE		Qsox1
Fcsk	439.855	1.674204335	0.25569	6.54773	5.84E-11	1.02E-09	TRUE		Fcsk
Foxn3	1945.869	1.349249929	0.27629	4.88343	1.04E-06	8.53E-06	TRUE		Foxn3
Sfxn5	781.9341	3.447312919	0.19663	17.5321	8.16E-69	1.31E-65	TRUE		Sfxn5
Emx1	1170.449	12.14869609	0.75527	16.0853	3.24E-58	2.70E-55	TRUE		Emx1
Spr	361.7075	1.239442888	0.27596	4.49131	7.08E-06	4.75E-05	TRUE		Spr
Gadd45gip1	110.7802	1.247732913	0.34066	3.66268	0.00025	0.00114	TRUE		Gadd45gip1
Recql4	476.0121	2.729620439	0.21518	12.6855	7.12E-37	1.77E-34	TRUE		Recql4
Tmem131l	2666.387	1.437370039	0.25476	5.64208	1.68E-08	2.00E-07	TRUE		Tmem131l
Cdk10	832.7752	1.365720041	0.3079	4.43562	9.18E-06	6.02E-05	TRUE		Cdk10
Ppargc1b	45.14619	3.10751867	0.70169	4.4286	9.48E-06	6.19E-05	TRUE		Ppargc1b
Usp36	1287.278	1.635898208	0.33889	4.82721	1.38E-06	1.10E-05	TRUE		Usp36
Chmp2a	1509.202	1.265542817	0.39354	3.21582	0.0013	0.00485	TRUE		Chmp2a
Aspm	687.0951	1.4731571	0.2654	5.55066	2.85E-08	3.25E-07	TRUE		Aspm
Slc16a2	234.7192	1.224965909	0.25886	4.73215	2.22E-06	1.67E-05	TRUE		Slc16a2
Gria2	3102.106	1.153755016	0.25135	4.59028	4.43E-06	3.11E-05	TRUE		Gria2
Tesk2	113.4243	1.809436341	0.32012	5.65231	1.58E-08	1.90E-07	TRUE		Tesk2
Cpsf1	1934.953	1.02832893	0.21683	4.74251	2.11E-06	1.60E-05	TRUE		Cpsf1
Fancd2	206.8976	1.481362732	0.27615	5.36437	8.12E-08	8.46E-07	TRUE		Fancd2
Lyl1	32.38041	1.673387706	0.69022	2.42441	0.01533	0.03984	TRUE		Lyl1
Phka1	239.9291	2.905063339	0.3219	9.02471	1.80E-19	8.86E-18	TRUE		Phka1
Fstl5	120.2026	1.358327592	0.44575	3.0473	0.00231	0.00803	TRUE		Fstl5
Ccs	506.7108	1.538686812	0.38635	3.98262	6.82E-05	0.00036	TRUE		Ccs
Scn11a	23.75907	5.203781474	0.74328	7.00108	2.54E-12	5.47E-11	TRUE		Scn11a
Mks1	303.2517	1.328552883	0.31168	4.26251	2.02E-05	0.00012	TRUE		Mks1
Emid1	185.46	2.860893223	0.296	9.66515	4.24E-22	2.66E-20	TRUE		Emid1
Polq	311.7385	1.579259549	0.33094	4.77206	1.82E-06	1.40E-05	TRUE		Polq
Slc25a37	235.375	1.776534915	0.23427	7.58317	3.37E-14	9.15E-13	TRUE		Slc25a37
Kif4	769.6723	1.828320353	0.19159	9.543	1.39E-21	8.43E-20	TRUE		Kif4
Slc26a2	151.2009	1.310613841	0.49618	2.64143	0.00826	0.0237	TRUE		Slc26a2
Brip1	124.8404	1.836683089	0.37355	4.91688	8.79E-07	7.31E-06	TRUE		Brip1
Smc4	2232.306	1.91556411	0.19575	9.78566	1.30E-22	8.61E-21	TRUE		Smc4
Kcnh5	23.53622	2.619239	0.70008	3.74135	0.00018	0.00087	TRUE		Kcnh5
Zfp707	345.2766	1.229231619	0.37349	3.29123	0.001	0.00384	TRUE		Zfp707
Scara3	129.7013	3.517126771	0.41304	8.51522	1.66E-17	6.55E-16	TRUE		Scara3
Caskin2	861.2819	1.747586094	0.23109	7.56252	3.95E-14	1.06E-12	TRUE		Caskin2
Diaph2	147.2981	1.127137269	0.37426	3.01164	0.0026	0.00889	TRUE		Diaph2
Gjc1	1078.017	1.239366192	0.17599	7.04207	1.89E-12	4.15E-11	TRUE		Gjc1
Ccpg1	74.03361	2.457379627	0.57249	4.29247	1.77E-05	0.00011	TRUE		Ccpg1
Pla2g3	36.85586	1.665669363	0.61972	2.68777	0.00719	0.02106	TRUE		Pla2g3
Pik3ip1	265.7349	1.157110485	0.23575	4.90822	9.19E-07	7.61E-06	TRUE		Pik3ip1
Gpr142	3.022095	6.483677357	2.36454	2.74204	0.00611	0.01839	TRUE		Gpr142
Dnaic2	28.61539	1.278227073	0.45896	2.78507	0.00535	0.01645	TRUE		Dnaic2
Adgrb1	4027.352	1.707722574	0.34659	4.92725	8.34E-07	6.97E-06	TRUE		Adgrb1
Pcdh11x	58.79001	2.115897124	0.43897	4.82011	1.43E-06	1.14E-05	TRUE		Pcdh11x
700006J14R	8.499666	3.201407228	1.00904	3.17272	0.00151	0.00554	TRUE		1700006J14Rik
Tle2	268.6697	1.664371102	0.45585	3.65113	0.00026	0.00119	TRUE		Tle2
BC030867	213.9439	2.903707106	0.36231	8.01439	1.11E-15	3.62E-14	TRUE		BC030867
Gna11	2426.826	1.016403296	0.32653	3.11278	0.00185	0.00661	TRUE		Gna11
Gpsm3	51.50261	1.799746516	0.54404	3.30812	0.00094	0.00365	TRUE		Gpsm3
Rab24	443.6387	2.113787343	0.44251	4.77684	1.78E-06	1.37E-05	TRUE		Rab24
Ncaph	664.1999	1.806031254	0.23313	7.74691	9.42E-15	2.76E-13	TRUE		Ncaph
Rubcnl	4.813308	4.829016702	1.52686	3.16271	0.00156	0.00571	TRUE		Rubcnl
Zbtb7a	609.8952	1.025247531	0.27787	3.68965	0.00022	0.00104	TRUE		Zbtb7a
Baz1a	1052.837	2.669263983	0.25769	10.3585	3.83E-25	3.27E-23	TRUE		Baz1a
Ncapd3	622.6053	1.009387931	0.21766	4.63742	3.53E-06	2.55E-05	TRUE		Ncapd3
Kri1	455.0795	1.409552794	0.35622	3.95703	7.59E-05	0.0004	TRUE		Kri1
Egln3	30.73911	2.247050251	0.65734	3.41842	0.00063	0.00257	TRUE		Egln3
Wnk4	222.4038	1.170527698	0.32438	3.60848	0.00031	0.00137	TRUE		Wnk4
Gcfc2	227.8196	1.279207404	0.32252	3.9663	7.30E-05	0.00038	TRUE		Gcfc2
Secisbp2	563.308	1.248417461	0.22669	5.50728	3.64E-08	4.07E-07	TRUE		Secisbp2
Tanc1	425.8735	4.461762959	0.35424	12.5954	2.24E-36	5.36E-34	TRUE		Tanc1
Fam124a	475.7748	1.560725577	0.2265	6.89073	5.55E-12	1.13E-10	TRUE		Fam124a
Lars2	118853.8	3.300059157	0.58206	5.66958	1.43E-08	1.74E-07	TRUE		Lars2

Abraxas1	145.5446	1.609147156	0.44338	3.62931	0.00028	0.00128	TRUE	Abraxas1
Lcat	34.01669	2.363190833	0.62303	3.79304	0.00015	0.00072	TRUE	Lcat
Amh	78.80161	2.097275322	0.59905	3.501	0.00046	0.00195	TRUE	Amh
Helq	455.5331	1.36759972	0.2398	5.7032	1.18E-08	1.45E-07	TRUE	Helq
Arx	1539.994	1.15525274	0.22085	5.23088	1.69E-07	1.64E-06	TRUE	Arx
Fbxo33	267.4861	1.051771908	0.23661	4.44523	8.78E-06	5.79E-05	TRUE	Fbxo33
Nfkbiz	52.1003	1.298757912	0.36411	3.56692	0.00036	0.00157	TRUE	Nfkbiz
Parpbp	76.71093	1.611962195	0.40434	3.98668	6.70E-05	0.00036	TRUE	Parpbp
Emsy	3787.192	1.489225545	0.30426	4.89451	9.86E-07	8.09E-06	TRUE	Emsy
Crb2	818.5423	1.627151544	0.22937	7.09389	1.30E-12	2.92E-11	TRUE	Crb2
Gnptg	1069.91	1.046831264	0.31027	3.37396	0.00074	0.00296	TRUE	Gnptg
Krt17	6.88228	4.215147304	1.14816	3.67123	0.00024	0.00111	TRUE	Krt17
Mpv17l2	229.9258	1.414452416	0.3257	4.34277	1.41E-05	8.86E-05	TRUE	Mpv17l2
Ankrd11	4708.631	1.104839807	0.27738	3.98315	6.80E-05	0.00036	TRUE	Ankrd11
600002K03R	294.4585	2.119476998	0.45173	4.69192	2.71E-06	2.01E-05	TRUE	1600002K03Rik
Cnot3	1804.599	1.043505544	0.26342	3.96145	7.45E-05	0.00039	TRUE	Cnot3
Grhpr	131.1456	1.236674347	0.28218	4.38262	1.17E-05	7.48E-05	TRUE	Grhpr
Zcchc7	900.5347	1.331466428	0.265	5.02435	5.05E-07	4.45E-06	TRUE	Zcchc7
Tnfsf9	41.66355	1.288215725	0.49666	2.59377	0.00949	0.02667	TRUE	Tnfsf9
Thrsp	27.77294	2.410703834	0.52598	4.58328	4.58E-06	3.21E-05	TRUE	Thrsp
Alg8	312.3076	1.636850486	0.28295	5.78504	7.25E-09	9.22E-08	TRUE	Alg8
Dok3	470.5418	2.079022934	0.39951	5.20399	1.95E-07	1.87E-06	TRUE	Dok3
Usp35	565.6184	1.860408122	0.36613	5.08122	3.75E-07	3.38E-06	TRUE	Usp35
Xylb	196.4304	2.309452569	0.2449	9.43035	4.09E-21	2.37E-19	TRUE	Xylb
Mlc1	255.2618	2.521883873	0.324	7.78368	7.04E-15	2.12E-13	TRUE	Mlc1
Polr3g	60.84612	2.253732098	0.4043	5.5744	2.48E-08	2.88E-07	TRUE	Polr3g
Ddx11	633.6745	2.468327432	0.23587	10.4649	1.25E-25	1.12E-23	TRUE	Ddx11
Alg12	264.4391	1.288043953	0.31888	4.03933	5.36E-05	0.00029	TRUE	Alg12
Misp	84.20324	2.037607955	0.53544	3.80549	0.00014	0.00069	TRUE	Misp
Pawr	50.13592	2.127619876	0.57691	3.68796	0.00023	0.00105	TRUE	Pawr
Rnf126	1654.24	1.135940531	0.29196	3.89078	9.99E-05	0.00051	TRUE	Rnf126
H2-Q4	54.03852	2.593486861	0.39534	6.5602	5.37E-11	9.48E-10	TRUE	H2-Q4
Ints6l	1251.881	1.360490543	0.22953	5.92729	3.08E-09	4.15E-08	TRUE	Ints6l
Parp2	588.4502	1.350389361	0.21745	6.21019	5.29E-10	8.12E-09	TRUE	Parp2
Zranb3	289.8447	2.416225279	0.24087	10.031	1.11E-23	8.44E-22	TRUE	Zranb3
Lmo1	277.2495	2.13395612	0.25967	8.21805	2.07E-16	7.31E-15	TRUE	Lmo1
Rfxank	207.1403	1.255568497	0.28343	4.42998	9.42E-06	6.15E-05	TRUE	Rfxank
Hist1h1c	100.2007	1.71129332	0.42439	4.03233	5.52E-05	0.0003	TRUE	Hist1h1c
Sapcd1	45.37666	2.070269371	0.5331	3.88343	0.0001	0.00052	TRUE	Sapcd1
Rif1	1046.053	1.644717676	0.26514	6.20319	5.53E-10	8.45E-09	TRUE	Rif1
Ska1	215.2943	2.184425232	0.3395	6.4343	1.24E-10	2.07E-09	TRUE	Ska1
Macrod1	48.67724	2.387663256	0.50279	4.74881	2.05E-06	1.55E-05	TRUE	Macrod1
Snapc4	1241.581	1.746320806	0.37714	4.63049	3.65E-06	2.62E-05	TRUE	Snapc4
P2ry14	44.80732	2.677775625	0.50815	5.26968	1.37E-07	1.36E-06	TRUE	P2ry14
Cep135	275.5935	2.134189563	0.26041	8.19552	2.50E-16	8.71E-15	TRUE	Cep135
530077C05R	71.49289	1.295501422	0.36886	3.51217	0.00044	0.00189	TRUE	9530077C05Rik
Wtip	481.9499	1.613238377	0.30032	5.37169	7.80E-08	8.16E-07	TRUE	Wtip
Ermard	302.1686	1.333208794	0.35105	3.7978	0.00015	0.00071	TRUE	Ermard
Upf3b	1664.906	1.559097574	0.3002	5.19349	2.06E-07	1.96E-06	TRUE	Upf3b
Etl4	954.7495	7.030956824	0.27659	25.4203	######	######	TRUE	Etl4
Clcn7	1273.851	1.348030293	0.3	4.49339	7.01E-06	4.71E-05	TRUE	Clcn7
Aaas	480.3651	1.684641227	0.21766	7.73976	9.96E-15	2.90E-13	TRUE	Aaas
Cc2d1a	1093.066	1.470878693	0.37487	3.92367	8.72E-05	0.00045	TRUE	Cc2d1a
Kif15	701.4675	1.288324988	0.21169	6.0858	1.16E-09	1.67E-08	TRUE	Kif15
Anln	336.548	1.173935542	0.32558	3.60572	0.00031	0.00138	TRUE	Anln
Slitrk2	152.8515	1.286819894	0.50812	2.5325	0.01133	0.03098	TRUE	Slitrk2
Pnpla7	137.7015	1.551163092	0.3579	4.33406	1.46E-05	9.19E-05	TRUE	Pnpla7
Hspb6	50.77695	1.386806938	0.36542	3.79511	0.00015	0.00072	TRUE	Hspb6
Syde2	37.91146	1.619977719	0.46627	3.47434	0.00051	0.00213	TRUE	Syde2
Proser3	536.4038	2.08456466	0.30816	6.76455	1.34E-11	2.59E-10	TRUE	Proser3
Smad6	70.5958	1.916196215	0.45442	4.21675	2.48E-05	0.00015	TRUE	Smad6
Dna2	333.3445	2.079579481	0.2599	8.00161	1.23E-15	4.00E-14	TRUE	Dna2
Arhgef26	98.4699	1.829811197	0.3978	4.59979	4.23E-06	2.99E-05	TRUE	Arhgef26
Fzd8	644.2501	3.387526611	0.31012	10.9233	8.92E-28	9.78E-26	TRUE	Fzd8
Pml	303.4215	1.73461445	0.28028	6.18894	6.06E-10	9.23E-09	TRUE	Pml
Wdr62	412.5085	2.314264653	0.23647	9.78677	1.28E-22	8.54E-21	TRUE	Wdr62
Mmaa	514.6877	1.030894175	0.31416	3.28138	0.00103	0.00396	TRUE	Mmaa
Inhbb	217.3789	4.510203206	0.44986	10.0259	1.17E-23	8.79E-22	TRUE	Inhbb
Zcwpw1	153.3231	1.360528643	0.33838	4.02071	5.80E-05	0.00031	TRUE	Zcwpw1
Arhgap10	87.78952	1.336070993	0.56418	2.36815	0.01788	0.04526	TRUE	Arhgap10
Ppp1r14a	110.3861	1.261564302	0.34446	3.66245	0.00025	0.00114	TRUE	Ppp1r14a
Mycn	562.5076	1.238830261	0.34631	3.57727	0.00035	0.00152	TRUE	Mycn
330009J07R	1048.652	1.894073304	0.19278	9.82495	8.79E-23	5.95E-21	TRUE	E330009J07Rik
Zfp692	1660.314	2.335128607	0.35555	6.56762	5.11E-11	9.06E-10	TRUE	Zfp692
Kin	544.5106	1.250114399	0.25534	4.89596	9.78E-07	8.04E-06	TRUE	Kin

Ldlrap1	45.74501	1.434578439	0.44586	3.21752	0.00129	0.00483	TRUE		Ldlrap1
Tacc3	922.5213	1.431567718	0.21861	6.54848	5.81E-11	1.02E-09	TRUE		Tacc3
Capn15	1057.941	1.376069903	0.29992	4.58819	4.47E-06	3.14E-05	TRUE		Capn15
Map4k1	79.55375	1.675826407	0.48553	3.45154	0.00056	0.0023	TRUE		Map4k1
Srrt	4183.815	1.232528722	0.26504	4.65032	3.31E-06	2.41E-05	TRUE		Srrt
Cep85	537.337	1.195489077	0.20676	5.78199	7.38E-09	9.37E-08	TRUE		Cep85
Arid5a	66.75055	1.232785785	0.41471	2.97261	0.00295	0.00992	TRUE		Arid5a
Tedc1	600.0125	1.952274333	0.19351	10.0886	6.20E-24	4.79E-22	TRUE		Tedc1
Dtl	474.0444	2.117147044	0.39548	5.35337	8.63E-08	8.95E-07	TRUE		Dtl
Thoc2	5206.602	2.126842357	0.40293	5.27846	1.30E-07	1.30E-06	TRUE		Thoc2
Mavs	90.53228	1.388081548	0.37853	3.66708	0.00025	0.00112	TRUE		Mavs
Plekhg2	1540.37	1.244173604	0.23078	5.39123	7.00E-08	7.39E-07	TRUE		Plekhg2
Wdhd1	746.8856	1.643559325	0.21305	7.71425	1.22E-14	3.51E-13	TRUE		Wdhd1
Kcnh3	87.44605	6.616633136	0.69353	9.54051	1.42E-21	8.61E-20	TRUE		Kcnh3
Spag1	269.1045	1.162406172	0.29062	3.99976	6.34E-05	0.00034	TRUE		Spag1
Atoh8	29.77095	2.411990637	0.5326	4.52869	5.94E-06	4.06E-05	TRUE		Atoh8
Inf2	228.3026	3.058355761	0.45503	6.72116	1.80E-11	3.44E-10	TRUE		Inf2
Ckap2	367.089	1.3192382	0.34816	3.78917	0.00015	0.00073	TRUE		Ckap2
Limch1	1101.64	2.06520808	0.23885	8.64662	5.30E-18	2.20E-16	TRUE		Limch1
Mrps26	841.3128	1.064184897	0.29269	3.63585	0.00028	0.00125	TRUE		Mrps26
Actr5	392.3568	1.159778688	0.33023	3.51201	0.00044	0.00189	TRUE		Actr5
Dzip1l	237.292	1.370924059	0.26768	5.12151	3.03E-07	2.78E-06	TRUE		Dzip1l
Rpl10a	6165.639	1.087531259	0.25805	4.21446	2.50E-05	0.00015	TRUE		Rpl10a
630003M21I	140.7564	1.434137316	0.42977	3.337	0.00085	0.00333	TRUE		D630003M21Rik
Aim2	322.9759	1.658708826	0.27657	5.99751	2.00E-09	2.76E-08	TRUE		Aim2
Ankrd9	88.25106	2.443898089	0.32336	7.55774	4.10E-14	1.10E-12	TRUE		Ankrd9
Nsrp1	2614.459	2.698135427	0.46898	5.75326	8.75E-09	1.10E-07	TRUE		Nsrp1
Card19	32.00674	2.132946995	0.48171	4.42787	9.52E-06	6.20E-05	TRUE		Card19
Ninj1	79.43409	1.694360115	0.38279	4.4263	9.59E-06	6.24E-05	TRUE		Ninj1
Itprid1	28.63951	6.623188243	0.98837	6.70111	2.07E-11	3.91E-10	TRUE		Itprid1
Neurod6	1051.241	8.112530529	1.11665	7.26504	3.73E-13	8.83E-12	TRUE		Neurod6
Sh3rf3	411.2368	2.061287484	0.43813	4.70471	2.54E-06	1.89E-05	TRUE		Sh3rf3
Rmi2	324.6377	3.286843944	0.28087	11.7024	1.24E-31	2.17E-29	TRUE		Rmi2
Ccdc138	219.6368	1.340612663	0.23565	5.68901	1.28E-08	1.56E-07	TRUE		Ccdc138
Mindy4	174.4994	1.0693767	0.31548	3.38967	0.0007	0.00282	TRUE		Mindy4
Igsf8	3893.67	1.476392221	0.30782	4.7963	1.62E-06	1.26E-05	TRUE		Igsf8
Mrm3	124.3181	1.050388056	0.36488	2.87869	0.00399	0.01281	TRUE		Mrm3
Cntnap5c	58.13265	4.200087639	0.78783	5.33124	9.75E-08	1.00E-06	TRUE		Cntnap5c
Kmt2c	2813.372	1.083339087	0.37224	2.91031	0.00361	0.01177	TRUE		Kmt2c
Cntln	435.734	2.094788958	0.32827	6.38127	1.76E-10	2.86E-09	TRUE		Cntln
Galnt11	561.3812	1.046386339	0.33132	3.15827	0.00159	0.00577	TRUE		Galnt11
Cdon	2780.775	3.836495212	0.23433	16.3721	3.02E-60	2.74E-57	TRUE		Cdon
Snrk	1835.894	1.242081556	0.43242	2.87238	0.00407	0.01302	TRUE		Snrk
Notch3	1094.73	1.566791762	0.21382	7.32746	2.35E-13	5.67E-12	TRUE		Notch3
Ttc39b	114.7307	2.250307239	0.50074	4.49397	6.99E-06	4.70E-05	TRUE		Ttc39b
Spag4	261.9328	1.215500651	0.32542	3.73512	0.00019	0.00089	TRUE		Spag4
Iqca1l	148.7059	6.345762517	0.57239	11.0863	1.46E-28	1.79E-26	TRUE		Iqca1l
Serpinf2	6.888353	2.378265025	1.0143	2.34473	0.01904	0.04766	TRUE		Serpinf2
Gask1a	30.42245	2.162687275	0.48318	4.47597	7.61E-06	5.08E-05	TRUE		Gask1a
Cep250	2695.135	1.699194638	0.30873	5.50383	3.72E-08	4.13E-07	TRUE		Cep250
Aox4	4.797265	3.086477986	1.3109	2.35448	0.01855	0.04667	TRUE		Aox4
Ncapd2	4297.777	1.280571886	0.1819	7.03978	1.93E-12	4.21E-11	TRUE		Ncapd2
Neurod2	6083.953	5.806375906	0.40643	14.2864	2.66E-46	1.26E-43	TRUE		Neurod2
Fau	4385.336	1.123487046	0.28795	3.90165	9.55E-05	0.00049	TRUE		Fau
Nop2	788.2619	1.327585792	0.26299	5.04806	4.46E-07	3.97E-06	TRUE		Nop2
Ccdc155	62.05906	2.179986984	0.55761	3.9095	9.25E-05	0.00047	TRUE		Ccdc155
Afg1l	79.25257	1.229496093	0.39216	3.1352	0.00172	0.00618	TRUE		Afg1l
700066M21F	179.9688	1.481712067	0.31641	4.68293	2.83E-06	2.09E-05	TRUE		1700066M21Rik
Satb2	153.5762	2.459532503	0.27433	8.96563	3.09E-19	1.48E-17	TRUE		Satb2
Ttk	320.8221	1.80872432	0.2869	6.30438	2.89E-10	4.58E-09	TRUE		Ttk
Pmepa1	1509.088	2.761344122	0.32431	8.51449	1.67E-17	6.58E-16	TRUE		Pmepa1
Tbkbp1	1669.034	1.651377348	0.27546	5.995	2.03E-09	2.80E-08	TRUE		Tbkbp1
Car14	122.521	3.120586663	0.31963	9.76325	1.62E-22	1.06E-20	TRUE		Car14
Ciart	130.8688	2.375730562	0.49737	4.7766	1.78E-06	1.37E-05	TRUE		Ciart
Ift172	1036.568	1.010249437	0.31791	3.17783	0.00148	0.00545	TRUE		Ift172
Pln	31.72178	2.133364101	0.51502	4.14231	3.44E-05	0.0002	TRUE		Pln
Tctn1	823.8743	1.380052251	0.34894	3.955	7.65E-05	0.0004	TRUE		Tctn1
Pold1	1545.814	2.261046364	0.24931	9.06914	1.20E-19	6.08E-18	TRUE		Pold1
Rnh1	550.0213	1.480844745	0.36995	4.00281	6.26E-05	0.00033	TRUE		Rnh1
Lpar1	314.5175	3.663644851	0.29958	12.2291	2.17E-34	4.62E-32	TRUE		Lpar1
Trps1	399.9783	2.187432123	0.24403	8.96376	3.14E-19	1.50E-17	TRUE		Trps1
Rtel1	1068.339	1.315486145	0.24972	5.26774	1.38E-07	1.37E-06	TRUE		Rtel1
Josd2	560.0003	1.010858223	0.27525	3.67251	0.00024	0.0011	TRUE		Josd2
Vill	62.13372	3.270663987	0.56136	5.82635	5.67E-09	7.36E-08	TRUE		Vill
Stap2	82.77652	1.763952426	0.56275	3.13455	0.00172	0.0062	TRUE		Stap2

Tmem214	1328.773	1.084435301	0.33027	3.28347	0.00103	0.00393	TRUE		Tmem214
Vars2	831.3621	1.631686225	0.31415	5.19402	2.06E-07	1.96E-06	TRUE		Vars2
Man2a2	1324.704	1.050245719	0.2114	4.96813	6.76E-07	5.78E-06	TRUE		Man2a2
Fam117a	88.29091	1.898439215	0.37839	5.01715	5.24E-07	4.60E-06	TRUE		Fam117a
Zfp653	1052.332	1.990053252	0.35644	5.5832	2.36E-08	2.75E-07	TRUE		Zfp653
Plcl2	1544.698	1.392572421	0.42708	3.26066	0.00111	0.00423	TRUE		Plcl2
Rccd1	479.3871	1.746966362	0.20485	8.52818	1.49E-17	5.89E-16	TRUE		Rccd1
Eme1	344.7503	2.087780289	0.24743	8.43782	3.23E-17	1.25E-15	TRUE		Eme1
Mtg2	741.7418	1.348444781	0.35252	3.82513	0.00013	0.00064	TRUE		Mtg2
Sart1	2398.323	1.021192562	0.24005	4.25401	2.10E-05	0.00013	TRUE		Sart1
Polg	867.9701	1.009537874	0.18131	5.56797	2.58E-08	2.97E-07	TRUE		Polg
Fanci	71.94184	1.176785369	0.47243	2.49094	0.01274	0.0341	TRUE		Fanci
Rlbp1	535.0617	4.623585724	0.3119	14.8241	1.02E-49	6.27E-47	TRUE		Rlbp1
Zdhhc1	902.9647	1.350533545	0.33156	4.07332	4.63E-05	0.00026	TRUE		Zdhhc1
Isg20	20.61955	2.350349961	0.6178	3.80439	0.00014	0.00069	TRUE		Isg20
Pomt1	668.8286	1.106210089	0.27992	3.95182	7.76E-05	0.0004	TRUE		Pomt1
Npepl1	734.2308	1.227330312	0.26458	4.63878	3.50E-06	2.53E-05	TRUE		Npepl1
Hexdc	717.7772	1.686946211	0.36289	4.64866	3.34E-06	2.43E-05	TRUE		Hexdc
Exosc2	566.8228	1.022020492	0.20388	5.01289	5.36E-07	4.70E-06	TRUE		Exosc2
Cdh6	388.9142	1.508789934	0.36991	4.07875	4.53E-05	0.00025	TRUE		Cdh6
Neil3	583.5866	2.13942103	0.25539	8.37714	5.42E-17	2.04E-15	TRUE		Neil3
Prdm16	589.7579	4.606222731	0.24354	18.9136	8.81E-80	2.62E-76	TRUE		Prdm16
Prrx2	44.773	3.733324964	0.53948	6.92028	4.51E-12	9.33E-11	TRUE		Prrx2
Asb6	770.5405	1.110847811	0.22515	4.93373	8.07E-07	6.77E-06	TRUE		Asb6
Mmd2	632.6617	1.664901947	0.32258	5.16116	2.45E-07	2.29E-06	TRUE		Mmd2
Flicr	51.10581	1.595545775	0.38746	4.118	3.82E-05	0.00022	TRUE		Flicr
Trmt9b	37.26763	1.474287181	0.51117	2.88414	0.00392	0.01263	TRUE		Trmt9b
Prex1	953.7945	1.703382236	0.27565	6.17945	6.43E-10	9.76E-09	TRUE		Prex1
Ap5z1	719.6543	1.52679787	0.31008	4.92386	8.49E-07	7.07E-06	TRUE		Ap5z1
Plekhg5	477.937	2.042518368	0.28291	7.21969	5.21E-13	1.21E-11	TRUE		Plekhg5
Exo1	322.9559	1.801216738	0.35852	5.02399	5.06E-07	4.46E-06	TRUE		Exo1
Thap3	832.7291	1.953723767	0.407	4.80036	1.58E-06	1.24E-05	TRUE		Thap3
Xkr5	37.97454	1.360426588	0.48545	2.8024	0.00507	0.01572	TRUE		Xkr5
Trub2	1024.734	1.50568616	0.30363	4.95893	7.09E-07	6.04E-06	TRUE		Trub2
Slc44a3	66.6723	1.333654635	0.48531	2.74805	0.006	0.01813	TRUE		Slc44a3
Slc39a5	28.82173	1.783740348	0.59205	3.01284	0.00259	0.00886	TRUE		Slc39a5
Eva1c	110.5761	4.445079855	0.52085	8.53435	1.41E-17	5.61E-16	TRUE		Eva1c
Slc26a11	168.3347	2.284120669	0.3238	7.05402	1.74E-12	3.82E-11	TRUE		Slc26a11
Etfbkmt	71.35522	1.149859942	0.37896	3.03424	0.00241	0.00833	TRUE		Etfbkmt
Rsbn1l	830.2779	1.588123451	0.32701	4.85648	1.19E-06	9.62E-06	TRUE		Rsbn1l
Timeless	1538.333	2.404724436	0.25968	9.26027	2.04E-20	1.12E-18	TRUE		Timeless
Saa4	4.686974	6.885996706	2.75838	2.49639	0.01255	0.03369	TRUE		Saa4
Bub1b	863.4456	1.482767571	0.22891	6.47749	9.33E-11	1.57E-09	TRUE		Bub1b
Abcc8	254.9041	4.196432092	0.42594	9.85224	6.70E-23	4.60E-21	TRUE		Abcc8
430038I01R	66.35681	1.313068197	0.42004	3.12605	0.00177	0.00635	TRUE		9430038I01Rik
Ikzf5	74.00875	1.256606545	0.36648	3.42885	0.00061	0.00248	TRUE		Ikzf5
Alkbh3	222.282	1.376800516	0.38902	3.53912	0.0004	0.00172	TRUE		Alkbh3
Daam2	254.3913	2.36516739	0.23812	9.93277	3.00E-23	2.13E-21	TRUE		Daam2
Klhdc4	1092.869	1.171245353	0.26474	4.42407	9.69E-06	6.30E-05	TRUE		Klhdc4
Accs	487.4645	1.33692461	0.24238	5.5158	3.47E-08	3.90E-07	TRUE		Accs
Cdk6	855.8223	1.972859335	0.18004	10.9576	6.11E-28	6.95E-26	TRUE		Cdk6
Hey1	424.8171	2.425203828	0.31772	7.63326	2.29E-14	6.34E-13	TRUE		Hey1
Ddx58	30.41186	1.24962101	0.52154	2.396	0.01657	0.04252	TRUE		Ddx58
Cchcr1	772.831	2.469414007	0.28888	8.54809	1.25E-17	5.02E-16	TRUE		Cchcr1
Skiv2l	2028.022	1.213776416	0.29915	4.05742	4.96E-05	0.00027	TRUE		Skiv2l
Slc25a28	1029.981	2.157145393	0.31287	6.89478	5.39E-12	1.10E-10	TRUE		Slc25a28
Hipk4	94.03114	2.399910945	0.31392	7.64491	2.09E-14	5.83E-13	TRUE		Hipk4
Plekha4	26.83861	1.962327696	0.59346	3.3066	0.00094	0.00366	TRUE		Plekha4
Mybbp1a	2497.882	1.321422558	0.24136	5.47484	4.38E-08	4.84E-07	TRUE		Mybbp1a
Dgkz	2110.691	1.623365713	0.27352	5.93502	2.94E-09	3.97E-08	TRUE		Dgkz
Bptf	5079.269	1.116911064	0.30805	3.62574	0.00029	0.00129	TRUE		Bptf
Dxo	612.606	1.056299472	0.24788	4.26129	2.03E-05	0.00012	TRUE		Dxo
Ltbp4	1075.445	1.986207174	0.17258	11.5087	1.19E-30	1.94E-28	TRUE		Ltbp4
Pvr	55.26224	2.679658581	0.53669	4.99289	5.95E-07	5.15E-06	TRUE		Pvr
Abhd11	330.1157	1.473527711	0.33899	4.34683	1.38E-05	8.72E-05	TRUE		Abhd11
Slc26a7	10.59692	4.545781721	1.20538	3.77125	0.00016	0.00078	TRUE		Slc26a7
Kazn	599.3655	1.179181424	0.18916	6.23391	4.55E-10	7.04E-09	TRUE		Kazn
Apobec1	45.59571	4.402573383	0.57547	7.65041	2.00E-14	5.60E-13	TRUE		Apobec1
Pck2	511.8206	1.75124033	0.27979	6.25906	3.87E-10	6.05E-09	TRUE		Pck2
Gemin8	240.4552	1.540540171	0.32671	4.71536	2.41E-06	1.81E-05	TRUE		Gemin8
Dnph1	228.2338	1.303853952	0.40592	3.21211	0.00132	0.00491	TRUE		Dnph1
Sh3bgr	23.13285	3.015237169	0.71069	4.24268	2.21E-05	0.00013	TRUE		Sh3bgr
Tbl3	1697.783	1.824581821	0.36523	4.9957	5.86E-07	5.08E-06	TRUE		Tbl3
Col16a1	164.3951	3.944458478	0.44292	8.9055	5.31E-19	2.46E-17	TRUE		Col16a1
Ap1g2	349.4035	2.094455334	0.35167	5.95571	2.59E-09	3.52E-08	TRUE		Ap1g2

Ppp1r13l	153.023	2.037951626	0.28746	7.08964	1.34E-12	3.00E-11	TRUE	Ppp1r13l
Six5	463.9746	2.614240389	0.24758	10.5593	4.60E-26	4.23E-24	TRUE	Six5
Sft2d2	503.4625	3.253628228	0.46431	7.00745	2.43E-12	5.25E-11	TRUE	Sft2d2
Erf	321.1619	1.899862851	0.33797	5.62137	1.89E-08	2.24E-07	TRUE	Erf
Ulk4	312.1863	2.464182317	0.3273	7.52887	5.12E-14	1.35E-12	TRUE	Ulk4
Arhgef1	3974.452	1.791534754	0.31865	5.62229	1.88E-08	2.23E-07	TRUE	Arhgef1
Rcc2	17516.96	2.205108965	0.40136	5.49406	3.93E-08	4.36E-07	TRUE	Rcc2
Rps19	6953.618	1.243894839	0.28225	4.40712	1.05E-05	6.77E-05	TRUE	Rps19
Cmtm8	62.19826	3.219852511	0.35878	8.97449	2.85E-19	1.38E-17	TRUE	Cmtm8
Mslnl	5.085013	3.584687782	1.24672	2.87529	0.00404	0.01293	TRUE	Mslnl
Pif1	644.4865	1.732006539	0.19012	9.11006	8.23E-20	4.26E-18	TRUE	Pif1
Fzd7	77.95719	1.252674099	0.42062	2.97815	0.0029	0.00977	TRUE	Fzd7
Tspyl2	1487.17	1.165603485	0.28326	4.11502	3.87E-05	0.00022	TRUE	Tspyl2
Zfp598	1880.418	1.186165727	0.24353	4.87066	1.11E-06	9.04E-06	TRUE	Zfp598
Brca2	509.8605	1.989645558	0.28659	6.94252	3.85E-12	8.07E-11	TRUE	Brca2
Spem1	44.99424	1.562302254	0.43758	3.57031	0.00036	0.00155	TRUE	Spem1
Hectd2	57.39785	1.908888943	0.64918	2.94047	0.00328	0.01081	TRUE	Hectd2
Prkd2	295.1627	1.01223985	0.27762	3.64618	0.00027	0.00121	TRUE	Prkd2
Chrnb1	27.00631	1.940153043	0.6086	3.1879	0.00143	0.00528	TRUE	Chrnb1
Chd7	10892.95	1.884611522	0.40177	4.69081	2.72E-06	2.02E-05	TRUE	Chd7
Rgl2	3375.026	1.489057814	0.3269	4.55514	5.23E-06	3.62E-05	TRUE	Rgl2
Ccdc9	1965.29	1.229378073	0.31389	3.91656	8.98E-05	0.00046	TRUE	Ccdc9
BC055324	157.5015	1.39948503	0.29779	4.69962	2.61E-06	1.94E-05	TRUE	BC055324
Nthl1	108.6584	1.635072487	0.30692	5.32733	9.97E-08	1.02E-06	TRUE	Nthl1
Kif14	308.3752	1.257566041	0.24274	5.18065	2.21E-07	2.09E-06	TRUE	Kif14
Sox5	1667.412	1.719049252	0.33801	5.08576	3.66E-07	3.30E-06	TRUE	Sox5
Disp3	725.624	1.569771926	0.24258	6.47126	9.72E-11	1.63E-09	TRUE	Disp3
Hspb8	13.99455	2.469177898	0.87012	2.83774	0.00454	0.01429	TRUE	Hspb8
Mrps27	294.3036	1.000754856	0.3393	2.94948	0.00318	0.01054	TRUE	Mrps27
Ddx24	1600.916	1.046832041	0.33757	3.10106	0.00193	0.00685	TRUE	Ddx24
BC006965	64.34023	3.004240485	0.49823	6.02987	1.64E-09	2.29E-08	TRUE	BC006965
Mpped1	1071.2	5.279510934	0.31046	17.0053	7.50E-65	8.23E-62	TRUE	Mpped1
Alg13	198.1826	1.3898689	0.36577	3.79987	0.00014	0.0007	TRUE	Alg13
Coro2b	1142.92	1.721674912	0.1731	9.94606	2.62E-23	1.88E-21	TRUE	Coro2b
Slc24a4	15.26726	6.639044763	1.33835	4.96062	7.03E-07	5.99E-06	TRUE	Slc24a4
Mapk1ip1	699.2824	2.104547186	0.3474	6.05792	1.38E-09	1.96E-08	TRUE	Mapk1ip1
Cir1	946.8392	1.883624755	0.40707	4.62731	3.70E-06	2.66E-05	TRUE	Cir1
Abca8a	4.02054	6.931845589	1.96926	3.52002	0.00043	0.00184	TRUE	Abca8a
Sytl3	6.524861	3.099590313	1.26006	2.45988	0.0139	0.03673	TRUE	Sytl3
Haus1	254.3772	1.153195916	0.24129	4.7793	1.76E-06	1.36E-05	TRUE	Haus1
Mcm3	812.6173	1.692177162	0.38721	4.37017	1.24E-05	7.90E-05	TRUE	Mcm3
Il17f	22.96279	4.687985204	0.73124	6.41099	1.45E-10	2.40E-09	TRUE	Il17f
Slc16a6	103.0835	2.394499629	0.42624	5.61777	1.93E-08	2.29E-07	TRUE	Slc16a6
Metap1d	1213.272	2.080805021	0.39344	5.28875	1.23E-07	1.24E-06	TRUE	Metap1d
Pigs	748.7699	1.078977977	0.19416	5.55704	2.74E-08	3.14E-07	TRUE	Pigs
Mettl8	242.3398	1.132788522	0.30399	3.7264	0.00019	0.00091	TRUE	Mettl8
Ncapg2	341.1024	1.211447441	0.32926	3.67929	0.00023	0.00108	TRUE	Ncapg2
Wdr60	317.3632	1.2079212	0.30637	3.94265	8.06E-05	0.00042	TRUE	Wdr60
Rassf4	138.212	1.875701012	0.32034	5.85538	4.76E-09	6.23E-08	TRUE	Rassf4
Armc5	411.2325	1.077881458	0.34045	3.16603	0.00155	0.00565	TRUE	Armc5
Bag2	113.5857	1.218868207	0.42352	2.87792	0.004	0.01284	TRUE	Bag2
Ammecr1	263.6074	1.111955592	0.28984	3.83639	0.00012	0.00062	TRUE	Ammecr1
Tmc7	162.3198	1.415557334	0.31336	4.5173	6.26E-06	4.25E-05	TRUE	Tmc7
Setd1a	456.1165	1.019278922	0.30725	3.31747	0.00091	0.00354	TRUE	Setd1a
Prox2	71.48246	1.206831901	0.3634	3.32092	0.0009	0.0035	TRUE	Prox2
Ikbke	24.91857	1.83770585	0.70265	2.61539	0.00891	0.02529	TRUE	Ikbke
Gnl3	554.1733	1.062145412	0.22987	4.6206	3.83E-06	2.74E-05	TRUE	Gnl3
Dmrt3	91.6851	7.128679312	0.70587	10.0991	5.58E-24	4.34E-22	TRUE	Dmrt3
Nfkbil1	272.2729	1.607185543	0.34269	4.68987	2.73E-06	2.03E-05	TRUE	Nfkbil1
Mfap4	1366.664	3.171282689	0.35903	8.83304	1.02E-18	4.56E-17	TRUE	Mfap4
Tbc1d8b	28.58252	1.561400933	0.50213	3.10953	0.00187	0.00667	TRUE	Tbc1d8b
Clspn	639.1785	2.012924656	0.31014	6.49034	8.56E-11	1.45E-09	TRUE	Clspn
AA986860	28.87991	1.774007248	0.64786	2.73825	0.00618	0.01856	TRUE	AA986860
Mier2	1295.304	1.549107131	0.24394	6.35048	2.15E-10	3.45E-09	TRUE	Mier2
Hirip3	1938.861	1.878834524	0.20736	9.06058	1.30E-19	6.53E-18	TRUE	Hirip3
Pbxip1	545.3978	2.107966092	0.40492	5.20591	1.93E-07	1.85E-06	TRUE	Pbxip1
Safb2	6812.452	1.972980104	0.32136	6.13942	8.28E-10	1.23E-08	TRUE	Safb2
Ypel3	1356.919	1.079669244	0.2794	3.8643	0.00011	0.00056	TRUE	Ypel3
Jph1	89.2864	3.178285917	0.37216	8.54012	1.34E-17	5.35E-16	TRUE	Jph1
Stn1	345.8807	1.533723578	0.26942	5.69279	1.25E-08	1.53E-07	TRUE	Stn1
Atpaf2	349.704	1.405967318	0.22679	6.19947	5.67E-10	8.64E-09	TRUE	Atpaf2
Muc1	577.5052	2.578461506	0.33563	7.68247	1.56E-14	4.43E-13	TRUE	Muc1
Zc3h6	256.8243	1.293912589	0.36868	3.50962	0.00045	0.0019	TRUE	Zc3h6
Foxo4	331.8516	1.14132491	0.30767	3.70959	0.00021	0.00097	TRUE	Foxo4
Nemp2	506.6005	1.996993919	0.24868	8.03053	9.71E-16	3.20E-14	TRUE	Nemp2

Gm16433	29.8227	2.1444785	0.55769	3.84526	0.00012	0.0006	TRUE	Gm16433
Spice1	359.8687	1.540233221	0.22433	6.866	6.60E-12	1.34E-10	TRUE	Spice1
Dpy19l1	486.0756	1.652129254	0.39773	4.15394	3.27E-05	0.00019	TRUE	Dpy19l1
Fam89a	34.6216	2.144857248	0.51125	4.19535	2.72E-05	0.00016	TRUE	Fam89a
Hpdl	30.60338	1.216242059	0.49772	2.44361	0.01454	0.03809	TRUE	Hpdl
Upf2	2151.946	2.080006143	0.41492	5.01306	5.36E-07	4.69E-06	TRUE	Upf2
Fam129c	127.052	1.34810059	0.40306	3.34468	0.00082	0.00325	TRUE	Fam129c
Trim56	165.901	1.27019069	0.4071	3.12012	0.00181	0.00646	TRUE	Trim56
Epop	384.5375	1.199303128	0.33314	3.59999	0.00032	0.00141	TRUE	Epop
Zfp536	944.7077	1.083482833	0.20584	5.26365	1.41E-07	1.40E-06	TRUE	Zfp536
Hscb	49.24095	1.402999426	0.44501	3.15275	0.00162	0.00587	TRUE	Hscb
Adamts3	14.39003	2.014749277	0.77441	2.60167	0.00928	0.02616	TRUE	Adamts3
Rbm20	91.89971	2.778011321	0.48422	5.73706	9.63E-09	1.20E-07	TRUE	Rbm20
610009L18R	19.53332	1.812980722	0.68627	2.64178	0.00825	0.02369	TRUE	0610009L18Rik
Pld6	50.43766	1.223684805	0.49436	2.47527	0.01331	0.03542	TRUE	Pld6
Ly6g5b	23.2252	1.785605411	0.56687	3.14993	0.00163	0.00591	TRUE	Ly6g5b
Mgat5b	2863.765	1.013173751	0.30732	3.29681	0.00098	0.00378	TRUE	Mgat5b
Ccdc84	365.1992	1.421144543	0.32145	4.42107	9.82E-06	6.38E-05	TRUE	Ccdc84
Emx2	468.3144	3.062275819	0.33755	9.07195	1.17E-19	5.94E-18	TRUE	Emx2
Spata31d1c	4.029401	6.705238432	2.22905	3.00811	0.00263	0.00898	TRUE	Spata31d1d
Cep164	1382.981	1.963398346	0.38577	5.08956	3.59E-07	3.24E-06	TRUE	Cep164
Pura	733.8854	1.619073776	0.32542	4.97526	6.52E-07	5.60E-06	TRUE	Pura
900052L18R	114.8959	2.296308038	0.43636	5.2624	1.42E-07	1.41E-06	TRUE	2900052L18Rik
Gls2	49.29659	1.558950492	0.54261	2.87304	0.00407	0.013	TRUE	Gls2
Slc35g1	42.87017	1.637735209	0.40469	4.04694	5.19E-05	0.00028	TRUE	Slc35g1
Rsbn1	1204.493	1.77690856	0.48017	3.70055	0.00022	0.001	TRUE	Rsbn1
Prss53	9.950787	2.808457047	0.94041	2.98643	0.00282	0.00954	TRUE	Prss53
310024B03R	29.29885	1.380628941	0.55282	2.49742	0.01251	0.03365	TRUE	1810024B03Rik
310030O07R	750.5513	1.195375314	0.22718	5.26185	1.43E-07	1.41E-06	TRUE	1810030O07Rik
Foxo1	118.0173	1.209096575	0.29198	4.14107	3.46E-05	0.0002	TRUE	Foxo1
Cdc25c	565.6805	2.447583198	0.2311	10.5912	3.27E-26	3.04E-24	TRUE	Cdc25c
Nkx2-3	42.99027	2.610469277	0.74103	3.52277	0.00043	0.00182	TRUE	Nkx2-3
Il20rb	100.5895	1.871222892	0.34927	5.35758	8.43E-08	8.76E-07	TRUE	Il20rb
Pcsk9	30.24713	1.377421919	0.45987	2.99523	0.00274	0.00932	TRUE	Pcsk9
Mab21l3	27.37506	1.53631359	0.58199	2.63977	0.0083	0.0238	TRUE	Mab21l3
700001O22R	108.5	1.232966725	0.35021	3.52062	0.00043	0.00183	TRUE	1700001O22Rik
Trp53i13	358.3954	1.722129475	0.21939	7.84965	4.17E-15	1.28E-13	TRUE	Trp53i13
Alkbh2	74.58272	2.117163033	0.44964	4.70861	2.49E-06	1.86E-05	TRUE	Alkbh2
Shisa2	435.6951	5.312806799	0.40954	12.9725	1.75E-38	4.81E-36	TRUE	Shisa2
Garem2	681.2118	1.379010934	0.19556	7.0516	1.77E-12	3.88E-11	TRUE	Garem2
Dnd1	64.96897	1.592868055	0.45638	3.49022	0.00048	0.00202	TRUE	Dnd1
Zfp612	700.1547	1.179814759	0.30461	3.87317	0.00011	0.00054	TRUE	Zfp612
Palb2	152.7098	1.608764464	0.30144	5.33688	9.46E-08	9.72E-07	TRUE	Palb2
Kcnj10	127.4223	4.308767665	0.58725	7.33719	2.18E-13	5.30E-12	TRUE	Kcnj10
Hjurp	5364.449	1.528975642	0.15791	9.68266	3.57E-22	2.26E-20	TRUE	Hjurp
Zfp36	100.2539	1.813689455	0.41594	4.36046	1.30E-05	8.23E-05	TRUE	Zfp36
930503L19R	410.0029	1.317865033	0.21831	6.03676	1.57E-09	2.21E-08	TRUE	4930503L19Rik
Sstr3	82.35575	5.337758916	0.4711	11.3304	9.28E-30	1.34E-27	TRUE	Sstr3
Zfp367	223.0975	1.407370163	0.44972	3.12945	0.00175	0.00629	TRUE	Zfp367
Ttc41	164.8175	1.404668365	0.38611	3.63797	0.00027	0.00124	TRUE	Ttc41
Tst	83.64167	2.462616878	0.41029	6.00214	1.95E-09	2.69E-08	TRUE	Tst
Gm9795	4.56242	5.300330027	1.74609	3.03554	0.0024	0.0083	TRUE	Gm9795
Rpsa-ps2	99.34364	1.482130224	0.3977	3.72678	0.00019	0.00091	TRUE	Rpsa-ps2
S1pr1	119.0314	1.748438989	0.43013	4.06486	4.81E-05	0.00026	TRUE	S1pr1
Rpl18a	12199.45	1.136499304	0.30636	3.70966	0.00021	0.00097	TRUE	Rpl18a
Sox3	435.8914	3.500956086	0.47502	7.3702	1.70E-13	4.21E-12	TRUE	Sox3
Cenph	367.5737	2.551276924	0.2197	11.6128	3.55E-31	6.01E-29	TRUE	Cenph
330549D23R	260.7025	1.863554778	0.41448	4.49616	6.92E-06	4.66E-05	TRUE	6330549D23Rik
Cenpe	1272.994	1.816367516	0.19907	9.12415	7.23E-20	3.80E-18	TRUE	Cenpe
Zfp956	313.2239	1.437871695	0.30639	4.693	2.69E-06	2.00E-05	TRUE	Zfp956
Krt14	3.670808	6.801756737	2.04731	3.32229	0.00089	0.00349	TRUE	Krt14
Esf1	996.2044	1.341140965	0.25951	5.16789	2.37E-07	2.22E-06	TRUE	Esf1
Plekha7	348.6084	2.484387194	0.29677	8.37142	5.69E-17	2.12E-15	TRUE	Plekha7
Col27a1	276.0842	1.449060718	0.53349	2.71621	0.0066	0.01961	TRUE	Col27a1
Prr15	28.08681	1.869198852	0.61184	3.05502	0.00225	0.00785	TRUE	Prr15
Mms22l	420.3731	2.045844733	0.26269	7.78804	6.81E-15	2.05E-13	TRUE	Mms22l
Tssc4	867.3431	1.024645777	0.21739	4.7135	2.44E-06	1.82E-05	TRUE	Tssc4
Whamm	294.9443	1.705802091	0.3507	4.86405	1.15E-06	9.30E-06	TRUE	Whamm
Zfp36l2	141.9617	1.018208861	0.38093	2.67296	0.00752	0.02186	TRUE	Zfp36l2
Ccdc9b	183.6696	1.879162735	0.28233	6.65588	2.82E-11	5.21E-10	TRUE	Ccdc9b
Tmem268	108.5809	1.907175941	0.34965	5.45452	4.91E-08	5.37E-07	TRUE	Tmem268
Mcmdc2	53.42759	1.818863824	0.37072	4.90631	9.28E-07	7.68E-06	TRUE	Mcmdc2
Fut10	116.9586	1.153012266	0.48169	2.39366	0.01668	0.04274	TRUE	Fut10
Adamts6	214.2332	2.965025197	0.27586	10.7482	6.04E-27	6.30E-25	TRUE	Adamts6
E2f8	169.6126	1.361266953	0.46839	2.90625	0.00366	0.0119	TRUE	E2f8

Ankle1	520.7286	2.037382019	0.24915	8.17739	2.90E-16	1.01E-14	TRUE		Ankle1
Gjb2	8.961644	7.984566301	1.49963	5.32437	1.01E-07	1.04E-06	TRUE		Gjb2
Rbp1	109.1873	1.908428405	0.53191	3.58787	0.00033	0.00147	TRUE		Rbp1
430212C06R	7.669739	4.249103814	1.35224	3.14227	0.00168	0.00605	TRUE		B430212C06Rik
Unc119b	2962.338	2.139185813	0.47274	4.52508	6.04E-06	4.12E-05	TRUE		Unc119b
Ticrr	455.9605	2.093482038	0.27751	7.54376	4.57E-14	1.21E-12	TRUE		Ticrr
C87436	302.4039	1.252642075	0.253	4.95116	7.38E-07	6.25E-06	TRUE		C87436
110032F04R	72.70957	3.675153206	0.56626	6.49023	8.57E-11	1.45E-09	TRUE		1110032F04Rik
Mipol1	104.6446	2.095974463	0.48206	4.34792	1.37E-05	8.68E-05	TRUE		Mipol1
Tmem164	1332.342	1.011243884	0.37403	2.70365	0.00686	0.02023	TRUE		Tmem164
Fam221a	35.02659	1.550909424	0.50548	3.06819	0.00215	0.00755	TRUE		Fam221a
Dmrta2	562.1409	3.903119683	0.28544	13.6738	1.46E-42	5.42E-40	TRUE		Dmrta2
Dync2h1	810.3791	1.558133329	0.32309	4.82265	1.42E-06	1.12E-05	TRUE		Dync2h1
Hist1h2be	28.93403	1.918725449	0.51824	3.70239	0.00021	0.00099	TRUE		Hist1h2be
Sfn	52.49687	1.189124005	0.4147	2.86741	0.00414	0.01321	TRUE		Sfn
Tgif1	94.49517	2.066884797	0.43277	4.77592	1.79E-06	1.38E-05	TRUE		Tgif1
Ctdspl	747.1191	2.875797698	0.35706	8.05403	8.01E-16	2.66E-14	TRUE		Ctdspl
Erfe	21.29697	2.481367009	0.56889	4.36179	1.29E-05	8.18E-05	TRUE		Erfe
Zfp30	747.1221	1.242140738	0.37111	3.34708	0.00082	0.00323	TRUE		Zfp30
Cldn4	3.371827	6.606945268	2.79564	2.3633	0.01811	0.04573	TRUE		Cldn4
Mis18bp1	478.6053	1.696620118	0.24996	6.78747	1.14E-11	2.23E-10	TRUE		Mis18bp1
Fgfbp3	163.911	2.14991005	0.57116	3.76409	0.00017	0.0008	TRUE		Fgfbp3
Cd3eap	1477.41	2.578222726	0.43807	5.88547	3.97E-09	5.25E-08	TRUE		Cd3eap
Rpsa-ps10	804.7286	1.784627431	0.42678	4.18164	2.89E-05	0.00017	TRUE		Rpsa-ps10
Tmem262	8.941569	3.619794467	1.40546	2.57552	0.01001	0.02788	TRUE		Tmem262
Fancb	63.32089	1.582358153	0.61723	2.56366	0.01036	0.02868	TRUE		Fancb
Lix1	245.1932	1.664093951	0.35747	4.65524	3.24E-06	2.36E-05	TRUE		Lix1
Flrt1	310.4571	2.159077888	0.52279	4.12995	3.63E-05	0.00021	TRUE		Flrt1
330154K18R	10.58286	4.308305403	1.50536	2.86197	0.00421	0.0134	TRUE		9330154K18Rik
Sstr2	368.3899	3.206777761	0.38706	8.28487	1.18E-16	4.26E-15	TRUE		Sstr2
Ankrd16	1271.93	1.075468188	0.25019	4.29854	1.72E-05	0.00011	TRUE		Ankrd16
Npm2	88.58041	2.55959136	0.52478	4.87746	1.07E-06	8.77E-06	TRUE		Npm2
Dipk1c	78.41228	1.862998377	0.31228	5.96587	2.43E-09	3.32E-08	TRUE		Dipk1c
Hes5	673.9457	2.528040743	0.29114	8.68332	3.84E-18	1.62E-16	TRUE		Hes5
Pirt	7.016059	4.684688839	1.51494	3.09233	0.00199	0.00702	TRUE		Pirt
Lmod1	64.47666	2.297682235	0.65177	3.5253	0.00042	0.0018	TRUE		Lmod1
Nwd1	84.19144	1.451152738	0.44142	3.28745	0.00101	0.00389	TRUE		Nwd1
Bcl11b	1186.593	1.836575221	0.28082	6.54007	6.15E-11	1.07E-09	TRUE		Bcl11b
Syngr2	128.2551	1.617809378	0.31525	5.1318	2.87E-07	2.65E-06	TRUE		Syngr2
Omd	56.02727	3.2868245	0.42709	7.69577	1.41E-14	4.02E-13	TRUE		Omd
Gli2	818.5672	2.216138864	0.27481	8.06428	7.37E-16	2.46E-14	TRUE		Gli2
Cyp4f16	482.5487	1.419708983	0.35129	4.04136	5.31E-05	0.00029	TRUE		Cyp4f16
Ccdc57	366.7737	2.111617605	0.38583	5.47298	4.43E-08	4.88E-07	TRUE		Ccdc57
Tyw5	100.0528	1.088468774	0.30822	3.5315	0.00041	0.00177	TRUE		Tyw5
Nog	101.9851	2.063189776	0.55998	3.68442	0.00023	0.00106	TRUE		Nog
Rhno1	1109.683	1.333241859	0.21002	6.34826	2.18E-10	3.50E-09	TRUE		Rhno1
Tpcn2	246.1152	2.238897212	0.40168	5.57377	2.49E-08	2.88E-07	TRUE		Tpcn2
Ggnbp1	50.13467	2.042694012	0.54815	3.72651	0.00019	0.00091	TRUE		Ggnbp1
130114P18R	339.0919	4.764328409	0.34919	13.6438	2.20E-42	7.76E-40	TRUE		E130114P18Rik
Cyb561d1	490.5717	1.000456275	0.23843	4.196	2.72E-05	0.00016	TRUE		Cyb561d1
Pkd1l3	24.15227	1.436695103	0.59353	2.42058	0.0155	0.04021	TRUE		Pkd1l3
Slc25a47	355.4518	1.838226341	0.39433	4.66162	3.14E-06	2.29E-05	TRUE		Slc25a47
Neurog1	175.7679	2.104925976	0.37551	5.60555	2.08E-08	2.44E-07	TRUE		Neurog1
Cdca2	1497.31	2.879810421	0.30119	9.56153	1.16E-21	7.07E-20	TRUE		Cdca2
Ccdc125	67.83159	1.716250997	0.45022	3.81206	0.00014	0.00067	TRUE		Ccdc125
Yjefn3	90.89778	1.014115492	0.39904	2.54138	0.01104	0.03033	TRUE		Yjefn3
H2al3	2.905458	6.127967225	2.45432	2.49681	0.01253	0.03369	TRUE		H2al3
Atxn7l2	1600.332	1.984688362	0.24375	8.14244	3.87E-16	1.32E-14	TRUE		Atxn7l2
Tex50	11.5521	2.246617896	0.79424	2.82864	0.00467	0.01465	TRUE		Tex50
Zfp219	5122.577	1.149644954	0.23458	4.90086	9.54E-07	7.87E-06	TRUE		Zfp219
Sorl1	518.1356	1.989130124	0.23922	8.315	9.18E-17	3.37E-15	TRUE		Sorl1
933408B17R	137.3098	1.262544814	0.32502	3.8845	0.0001	0.00052	TRUE		4933408B17Rik
Dtx3l	73.45472	2.674512266	0.52946	5.05142	4.39E-07	3.91E-06	TRUE		Dtx3l
Hist1h1a	29.15508	3.297945681	0.65461	5.03801	4.70E-07	4.17E-06	TRUE		Hist1h1a
Polr1a	642.5325	1.296594816	0.2193	5.91234	3.37E-09	4.52E-08	TRUE		Polr1a
Zfp644	1502.178	1.105794941	0.34499	3.20532	0.00135	0.00501	TRUE		Zfp644
610318N02R	832.5641	1.202988085	0.31522	3.81629	0.00014	0.00066	TRUE		2610318N02Rik
Plekhf2	147.6063	1.141961186	0.38668	2.95324	0.00314	0.01044	TRUE		Plekhf2
Glt1d1	150.0569	1.194097039	0.36314	3.2883	0.00101	0.00388	TRUE		Glt1d1
Amz1	89.62025	2.05536856	0.33369	6.15951	7.30E-10	1.10E-08	TRUE		Amz1
Gpr171	9.138206	3.545861442	0.94861	3.73796	0.00019	0.00088	TRUE		Gpr171
Haspin	84.53712	1.076671247	0.43316	2.48563	0.01293	0.03453	TRUE		Haspin
Evc2	300.9719	2.340327136	0.24367	9.60431	7.67E-22	4.76E-20	TRUE		Evc2
Fzd2	777.802	1.981273127	0.24859	7.97017	1.58E-15	5.11E-14	TRUE		Fzd2
Gm9843	57.66172	1.614093327	0.42906	3.76194	0.00017	0.0008	TRUE		Gm9843

Ndufaf6	85.53857	1.542077875	0.30093	5.12435	2.99E-07	2.74E-06	TRUE	Ndufaf6
130071C03F	2320.837	2.166037457	0.24275	8.92284	4.54E-19	2.13E-17	TRUE	C130071C03Rik
Lgals3	22.78747	4.255453264	1.04664	4.06581	4.79E-05	0.00026	TRUE	Lgals3
Kif7	509.6486	1.918231838	0.22839	8.39876	4.51E-17	1.71E-15	TRUE	Kif7
Tcf19	468.3568	1.871469766	0.19791	9.4564	3.19E-21	1.87E-19	TRUE	Tcf19
Lypd6	52.50135	3.996563957	0.47105	8.4844	2.17E-17	8.45E-16	TRUE	Lypd6
Fbxl22	280.4009	2.005973687	0.34572	5.80235	6.54E-09	8.43E-08	TRUE	Fbxl22
Hyls1	412.9805	1.371639784	0.24578	5.5807	2.40E-08	2.78E-07	TRUE	Hyls1
Zfp61	717.9551	1.324780866	0.27655	4.79045	1.66E-06	1.29E-05	TRUE	Zfp61
930563M21F	96.71123	3.509976323	0.4602	7.62705	2.40E-14	6.63E-13	TRUE	4930563M21Rik
Gm5084	6.928515	2.717214958	1.13738	2.38901	0.01689	0.04319	TRUE	Gm5084
Gja1	148.6434	1.333574221	0.49477	2.69535	0.00703	0.02066	TRUE	Gja1
P4ha3	35.26977	1.507423211	0.52095	2.89358	0.00381	0.01231	TRUE	P4ha3
Rpusd3	53.18986	2.245949063	0.54192	4.1444	3.41E-05	0.00019	TRUE	Rpusd3
Plcb1	250.4093	2.007265805	0.24214	8.28974	1.13E-16	4.12E-15	TRUE	Plcb1
Gen1	636.7097	2.212698053	0.25189	8.78448	1.57E-18	6.86E-17	TRUE	Gen1
Msrb3	82.4216	1.897386743	0.57782	3.28367	0.00102	0.00393	TRUE	Msrb3
Msantd1	95.12738	1.45088531	0.4719	3.07454	0.00211	0.00741	TRUE	Msantd1
Zgrf1	523.3718	2.438111008	0.19399	12.5681	3.16E-36	7.49E-34	TRUE	Zgrf1
630028B13F	105.1359	6.785405133	0.51555	13.1616	1.46E-39	4.23E-37	TRUE	9630028B13Rik
Usp42	1482.875	1.231827284	0.3178	3.87606	0.00011	0.00053	TRUE	Usp42
Mtln	82.2842	1.204002096	0.30481	3.95001	7.81E-05	0.00041	TRUE	Mtln
900026A02F	395.159	3.006156533	0.35253	8.52736	1.50E-17	5.92E-16	TRUE	2900026A02Rik
Kif18b	317.9166	1.683920755	0.28001	6.01385	1.81E-09	2.51E-08	TRUE	Kif18b
Tacstd2	3.371827	6.606945268	2.79564	2.3633	0.01811	0.04573	TRUE	Tacstd2
Bbs12	114.5556	1.705111972	0.27603	6.17724	6.52E-10	9.87E-09	TRUE	Bbs12
Mafg	2331.928	1.112459607	0.39097	2.8454	0.00444	0.01399	TRUE	Mafg
Arhgef39	511.1729	2.381052672	0.27	8.81864	1.16E-18	5.15E-17	TRUE	Arhgef39
Zfp579	3206.125	1.157895796	0.31571	3.6676	0.00024	0.00112	TRUE	Zfp579
Otud6a	11.10267	4.326558183	1.22644	3.52773	0.00042	0.00179	TRUE	Otud6a
Hist1h1e	141.7989	2.659506494	0.57411	4.63239	3.61E-06	2.60E-05	TRUE	Hist1h1e
Kctd14	68.88201	2.55562941	0.45844	5.57461	2.48E-08	2.87E-07	TRUE	Kctd14
Xrcc1	1477.995	2.046619862	0.21957	9.32114	1.15E-20	6.43E-19	TRUE	Xrcc1
Sox6	1004.435	1.894079623	0.23093	8.20209	2.36E-16	8.27E-15	TRUE	Sox6
Prdm9	104.8644	1.093582653	0.3643	3.00187	0.00268	0.00914	TRUE	Prdm9
Tagap1	682.8283	1.591790815	0.31254	5.09305	3.52E-07	3.19E-06	TRUE	Tagap1
Pard3b	334.8891	2.058163796	0.42621	4.82897	1.37E-06	1.09E-05	TRUE	Pard3b
730043M19F	425.4655	3.485500417	0.28459	12.2474	1.73E-34	3.76E-32	TRUE	F730043M19Rik
Sema5b	3493.65	1.722768341	0.26468	6.50884	7.57E-11	1.30E-09	TRUE	Sema5b
Foxo6	514.4715	2.867090486	0.33131	8.65386	4.98E-18	2.07E-16	TRUE	Foxo6
Rps10	98.01847	1.134943091	0.35275	3.21746	0.00129	0.00483	TRUE	Rps10
Cemip	72.42758	2.29404072	0.52213	4.39359	1.11E-05	7.16E-05	TRUE	Cemip
Actn2	46.28295	3.247349967	0.56054	5.7933	6.90E-09	8.80E-08	TRUE	Actn2
Rft1	181.795	1.09206655	0.33788	3.23209	0.00123	0.00462	TRUE	Rft1
Ezr	979.0418	2.63495685	0.25356	10.3918	2.70E-25	2.34E-23	TRUE	Ezr
Rexo4	899.0086	1.2342326	0.25895	4.76623	1.88E-06	1.44E-05	TRUE	Rexo4
Ccdc171	82.97281	1.250249825	0.33703	3.70959	0.00021	0.00097	TRUE	Ccdc171
Bmpr1b	85.60861	1.440032741	0.36911	3.90138	9.56E-05	0.00049	TRUE	Bmpr1b
Gm8174	14.13949	4.271942029	0.8175	5.22561	1.74E-07	1.68E-06	TRUE	Gm8174
Spdya	32.28349	2.397572185	0.45705	5.24573	1.56E-07	1.53E-06	TRUE	Spdya
930048N14F	13.55801	2.301224442	0.95852	2.40081	0.01636	0.04205	TRUE	D930048N14Rik
Hist1h1d	112.5934	3.25606424	0.71638	4.54518	5.49E-06	3.78E-05	TRUE	Hist1h1d
Hook2	463.1668	1.475326357	0.28233	5.22548	1.74E-07	1.68E-06	TRUE	Hook2
Gm9885	32.46738	2.412611098	0.43375	5.56217	2.66E-08	3.06E-07	TRUE	Gm9885
830454E08F	15.09355	1.61096259	0.67769	2.37712	0.01745	0.04438	TRUE	5830454E08Rik
Jun	925.9285	2.781866768	0.25922	10.7319	7.21E-27	7.33E-25	TRUE	Jun
Gm9888	10.07553	2.571908616	0.85697	3.00115	0.00269	0.00916	TRUE	Gm9888
Swt1	494.4588	1.876270572	0.26759	7.01178	2.35E-12	5.09E-11	TRUE	Swt1
Gm9889	3.206187	4.827068938	1.91962	2.5146	0.01192	0.03232	TRUE	Gm9889
130026L21F	52.35989	1.775154918	0.42493	4.17753	2.95E-05	0.00017	TRUE	C130026L21Rik
Nrk	19.46738	1.681410247	0.62353	2.69661	0.007	0.02061	TRUE	Nrk
Prkcb	605.296	1.838377204	0.28992	6.34093	2.28E-10	3.65E-09	TRUE	Prkcb
Lamb2	1248.159	2.675260784	0.27268	9.81083	1.01E-22	6.82E-21	TRUE	Lamb2
Rnaseh2a	561.5158	1.654512976	0.23652	6.99525	2.65E-12	5.67E-11	TRUE	Rnaseh2a
Gas1	689.4176	3.66184852	0.43725	8.37464	5.54E-17	2.08E-15	TRUE	Gas1
Creb5	1633.952	5.637545317	0.32664	17.2594	9.51E-67	1.24E-63	TRUE	Creb5
Yap1	403.5938	1.744709832	0.41832	4.17076	3.04E-05	0.00018	TRUE	Yap1
Socs3	174.1406	1.455826739	0.24159	6.02608	1.68E-09	2.34E-08	TRUE	Socs3
330013P04F	34.12238	4.349782242	0.64528	6.74089	1.57E-11	3.02E-10	TRUE	E330013P04Rik
Mterf1b	70.66382	1.216329928	0.36431	3.33871	0.00084	0.00331	TRUE	Mterf1b
Dand5	82.40431	1.403016052	0.39885	3.51761	0.00044	0.00185	TRUE	Dand5
Trmt1l	837.0318	1.118509975	0.22179	5.04309	4.58E-07	4.07E-06	TRUE	Trmt1l
Nrgn	41.68445	2.188787722	0.4839	4.52324	6.09E-06	4.15E-05	TRUE	Nrgn
Epp13	9.32925	3.05766257	1.12095	2.72775	0.00638	0.01906	TRUE	Epp13
Phgdh	741.701	2.04362692	0.26687	7.65773	1.89E-14	5.31E-13	TRUE	Phgdh

Adamts18	140.6208	2.883831554	0.45635	6.3193	2.63E-10	4.18E-09	TRUE	Adamts18
Hhipl2	33.43238	1.628751878	0.55983	2.90937	0.00362	0.0118	TRUE	Hhipl2
Tcf4	15809.08	3.441305937	0.20054	17.1602	5.27E-66	6.46E-63	TRUE	Tcf4
Ebf4	713.5239	2.496348385	0.36608	6.81908	9.16E-12	1.81E-10	TRUE	Ebf4
Ier2	394.6109	1.63107576	0.25016	6.52022	7.02E-11	1.21E-09	TRUE	Ier2
Gm9915	11.95841	5.521547452	1.20896	4.56719	4.94E-06	3.44E-05	TRUE	Gm9915
Chd8	3635.077	1.085348573	0.34017	3.19058	0.00142	0.00524	TRUE	Chd8
Gm9922	6.41868	5.640543973	1.56267	3.60955	0.00031	0.00137	TRUE	Gm9922
Lipg	83.76015	5.165036634	0.41129	12.5581	3.59E-36	8.40E-34	TRUE	Lipg
Lgals4	48.45622	1.167041648	0.45693	2.55408	0.01065	0.02938	TRUE	Lgals4
Tdrd9	25.11508	3.002769138	0.78377	3.83117	0.00013	0.00063	TRUE	Tdrd9
930004D18R	272.8113	1.023783665	0.30417	3.3658	0.00076	0.00304	TRUE	A930004D18Rik
Krt15	4.433021	7.05057061	1.98394	3.55382	0.00038	0.00164	TRUE	Krt15
Syne3	6.603578	4.662578188	1.80951	2.57671	0.00997	0.02781	TRUE	Syne3
Fam83e	30.67775	3.20907471	0.56277	5.7023	1.18E-08	1.45E-07	TRUE	Fam83e
930012O16R	8.580589	4.967758167	1.36269	3.64557	0.00027	0.00121	TRUE	A930012O16Rik
Gzmm	63.46327	2.103319172	0.60045	3.5029	0.00046	0.00194	TRUE	Gzmm
Fgfr3	1642.807	2.11684277	0.21599	9.80079	1.12E-22	7.49E-21	TRUE	Fgfr3
130007C19R	84.61373	2.189160365	0.37983	5.76352	8.24E-09	1.04E-07	TRUE	D130007C19Rik
930005H10R	90.9207	2.131786071	0.41276	5.16467	2.41E-07	2.26E-06	TRUE	A930005H10Rik
Sytl5	24.01901	5.77029334	1.05858	5.45097	5.01E-08	5.47E-07	TRUE	Sytl5
Kcnn2	129.636	2.125730205	0.28948	7.34335	2.08E-13	5.08E-12	TRUE	Kcnn2
Ankrd24	738.6967	1.418194476	0.3538	4.00851	6.11E-05	0.00033	TRUE	Ankrd24
Zfp771	1117.978	1.463240496	0.32276	4.53349	5.80E-06	3.98E-05	TRUE	Zfp771
Actn4	2501.183	1.127364634	0.32453	3.47379	0.00051	0.00214	TRUE	Actn4
Rnd1	135.3333	1.309659855	0.33105	3.95607	7.62E-05	0.0004	TRUE	Rnd1
330416C01R	18.79891	2.686078242	0.78836	3.40717	0.00066	0.00266	TRUE	5330416C01Rik
Naaladl1	107.8099	1.307638522	0.26889	4.86315	1.16E-06	9.34E-06	TRUE	Naaladl1
Nfic	603.3067	1.566121161	0.23526	6.65692	2.80E-11	5.18E-10	TRUE	Nfic
Ankrd17	3306.358	1.075053464	0.31585	3.40364	0.00066	0.00269	TRUE	Ankrd17
Tns1	553.8024	2.4847589	0.34146	7.2769	3.42E-13	8.14E-12	TRUE	Tns1
Fbxo6	275.1116	1.179612729	0.31647	3.72737	0.00019	0.00091	TRUE	Fbxo6
Maf	441.2727	3.197308831	0.57507	5.55987	2.70E-08	3.10E-07	TRUE	Maf
Srsf11	9060.95	1.849784376	0.41138	4.49659	6.91E-06	4.65E-05	TRUE	Srsf11
Pprc1	630.5884	1.100295745	0.21394	5.14303	2.70E-07	2.51E-06	TRUE	Pprc1
Lair1	66.7815	2.781849063	0.50115	5.55091	2.84E-08	3.25E-07	TRUE	Lair1
Zfp459	4.436667	2.814185979	1.16873	2.4079	0.01604	0.04137	TRUE	Zfp459
Hmcn2	17.50252	2.036590152	0.78909	2.58094	0.00985	0.02753	TRUE	Hmcn2
Kbtbd11	580.2403	1.054224579	0.22237	4.74096	2.13E-06	1.61E-05	TRUE	Kbtbd11
Usp26	4.686974	6.885996706	2.75838	2.49639	0.01255	0.03369	TRUE	Usp26
Tcf7l1	594.5999	4.224914177	0.22726	18.5903	3.85E-77	8.91E-74	TRUE	Tcf7l1
Zfp1	423.9251	1.033351655	0.33327	3.10061	0.00193	0.00686	TRUE	Zfp1
Ppp4r1l-ps	1451.655	1.289702034	0.33773	3.81872	0.00013	0.00066	TRUE	Ppp4r1l-ps
Tmem69	264.1665	1.102040032	0.29299	3.76137	0.00017	0.00081	TRUE	Tmem69
Tmem150a	153.6872	1.062247656	0.41954	2.53196	0.01134	0.03102	TRUE	Tmem150a
Aasdh	315.7911	1.081814219	0.31603	3.42314	0.00062	0.00253	TRUE	Aasdh
Sag	48.12025	1.559546601	0.49481	3.15184	0.00162	0.00588	TRUE	Sag
Grik2	1055.881	1.537379434	0.2073	7.41634	1.20E-13	3.04E-12	TRUE	Grik2
AI504432	1001.816	1.554163699	0.37744	4.11761	3.83E-05	0.00022	TRUE	AI504432
Nanos3	71.66504	1.086766826	0.3837	2.83231	0.00462	0.01451	TRUE	Nanos3
Cnot10	1426.433	1.434921395	0.27114	5.29224	1.21E-07	1.22E-06	TRUE	Cnot10
Cars2	336.0019	1.07130367	0.26577	4.03101	5.55E-05	0.0003	TRUE	Cars2
Usp34	3689.176	1.69425195	0.43191	3.92265	8.76E-05	0.00045	TRUE	Usp34
Actr3b	169.1865	1.870438668	0.27249	6.86427	6.68E-12	1.35E-10	TRUE	Actr3b
AI987944	149.4054	2.319765376	0.40443	5.73587	9.70E-09	1.21E-07	TRUE	AI987944
Lig1	1732.089	1.716232888	0.24208	7.08947	1.35E-12	3.00E-11	TRUE	Lig1
Med12l	107.3236	1.576367872	0.38867	4.05575	5.00E-05	0.00027	TRUE	Med12l
Ccdc18	204.8369	2.839706454	0.43778	6.4866	8.78E-11	1.48E-09	TRUE	Ccdc18
Fkbp2	797.9341	1.154016906	0.3411	3.38322	0.00072	0.00288	TRUE	Fkbp2
930024E05R	143.0991	3.571562025	0.29891	11.9485	6.61E-33	1.29E-30	TRUE	A930024E05Rik
Hmga2	358.7955	2.787734037	0.42438	6.56902	5.06E-11	8.99E-10	TRUE	Hmga2
Cep162	559.9033	1.160564128	0.24886	4.66348	3.11E-06	2.28E-05	TRUE	Cep162
Gm7536	289.2387	2.127294294	0.50975	4.17323	3.00E-05	0.00017	TRUE	Gm7536
Cntrl	1558.04	2.007305182	0.30163	6.65495	2.83E-11	5.24E-10	TRUE	Cntrl
Chd6	3662.431	1.346075431	0.32761	4.10871	3.98E-05	0.00022	TRUE	Chd6
Dmac2	305.2929	1.113581579	0.27616	4.03233	5.52E-05	0.0003	TRUE	Dmac2
Bbof1	88.62469	1.539039562	0.44328	3.47196	0.00052	0.00215	TRUE	Bbof1
Bcl2	408.6566	1.108349309	0.2375	4.66678	3.06E-06	2.24E-05	TRUE	Bcl2
Chst3	131.9277	2.490446455	0.31899	7.8074	5.84E-15	1.78E-13	TRUE	Chst3
Lmcd1	63.39906	4.49340564	0.4156	10.8118	3.03E-27	3.20E-25	TRUE	Lmcd1
Brd9	4533.713	1.864767956	0.40962	4.55238	5.30E-06	3.66E-05	TRUE	Brd9
Zfp595	23.42007	1.604874013	0.6636	2.41844	0.01559	0.04043	TRUE	Zfp595
Rpl36	2703.228	1.4902252	0.33728	4.41841	9.94E-06	6.46E-05	TRUE	Rpl36
Unc13d	39.92495	2.106988003	0.59636	3.53307	0.00041	0.00176	TRUE	Unc13d
Itpk1	824.2722	1.922050413	0.25866	7.43091	1.08E-13	2.75E-12	TRUE	Itpk1

Smco4	61.56994	1.857917439	0.62326	2.98094	0.00287	0.00969	TRUE	Smco4
Espl1	1059.501	2.096492562	0.25447	8.23873	1.74E-16	6.21E-15	TRUE	Espl1
Dock1	233.5063	1.143740494	0.34787	3.28781	0.00101	0.00388	TRUE	Dock1
Smim4	224.0972	1.122422098	0.39478	2.84313	0.00447	0.01407	TRUE	Smim4
Gm6612	8.734127	5.398052974	1.34815	4.00404	6.23E-05	0.00033	TRUE	Gm6612
Hist1h2bg	20.28657	2.897095308	0.62606	4.62752	3.70E-06	2.66E-05	TRUE	Hist1h2bg
Rrp1b	629.4138	1.398259026	0.28651	4.88029	1.06E-06	8.66E-06	TRUE	Rrp1b
Gpr182	117.6159	2.050201971	0.46325	4.4257	9.61E-06	6.26E-05	TRUE	Gpr182
Fam133b	1430.98	1.965733423	0.38087	5.16116	2.45E-07	2.29E-06	TRUE	Fam133b
Gpc6	85.26064	1.336610182	0.4574	2.92221	0.00348	0.01139	TRUE	Gpc6
Serhl	47.15408	2.154387048	0.40145	5.36647	8.03E-08	8.37E-07	TRUE	Serhl
Rpl28-ps1	236.2518	1.487995094	0.33822	4.39952	1.08E-05	6.99E-05	TRUE	Rpl28-ps1
Fcer1g	23.31106	1.472965304	0.61858	2.38121	0.01726	0.04397	TRUE	Fcer1g
Rnf169	1086.783	1.445661998	0.34044	4.24648	2.17E-05	0.00013	TRUE	Rnf169
Hist1h1b	48.69306	3.091890586	0.48359	6.39362	1.62E-10	2.66E-09	TRUE	Hist1h1b
Opn1sw	26.0382	4.022461128	0.76946	5.22763	1.72E-07	1.67E-06	TRUE	Opn1sw
Zfp516	336.2373	1.478863391	0.22402	6.60161	4.07E-11	7.36E-10	TRUE	Zfp516
Hdhd5	575.9765	1.706386247	0.28306	6.02838	1.66E-09	2.31E-08	TRUE	Hdhd5
630013D21R	6.664158	3.221769698	1.1692	2.75554	0.00586	0.01778	TRUE	9630013D21Rik
Rad51b	37.60507	1.662387378	0.53663	3.09785	0.00195	0.00691	TRUE	Rad51b
Ntrk3	1131.034	1.406868925	0.26174	5.37514	7.65E-08	8.01E-07	TRUE	Ntrk3
Tonsl	724.7002	2.401650871	0.33678	7.13123	9.95E-13	2.25E-11	TRUE	Tonsl
Fxn	332.1485	2.298772835	0.22516	10.2094	1.80E-24	1.48E-22	TRUE	Fxn
Nkapl	71.6391	1.489982096	0.41327	3.60532	0.00031	0.00138	TRUE	Nkapl
Mamld1	409.8753	1.031051713	0.22786	4.52488	6.04E-06	4.12E-05	TRUE	Mamld1
Nhs	264.8832	1.928686883	0.22007	8.76405	1.88E-18	8.16E-17	TRUE	Nhs
Tcea2	437.9207	1.277163437	0.30184	4.23129	2.32E-05	0.00014	TRUE	Tcea2
Kcnh7	450.379	1.412614245	0.38909	3.63055	0.00028	0.00127	TRUE	Kcnh7
Fdps	116.0791	1.346657226	0.35086	3.83818	0.00012	0.00061	TRUE	Fdps
Nrm	357.0099	1.402832275	0.22972	6.10681	1.02E-09	1.48E-08	TRUE	Nrm
Sclt1	341.8426	1.400410446	0.29289	4.78136	1.74E-06	1.35E-05	TRUE	Sclt1
Tnip2	76.8102	1.158967865	0.3896	2.97477	0.00293	0.00986	TRUE	Tnip2
430015G10R	1616.155	1.88655074	0.22095	8.53831	1.36E-17	5.43E-16	TRUE	9430015G10Rik
Gm10073	44.11887	1.870533478	0.4338	4.31197	1.62E-05	0.0001	TRUE	Gm10073
Hist1h4a	15.81706	2.008129611	0.8543	2.35062	0.01874	0.04704	TRUE	Hist1h4a
Gemin2	163.9217	1.145900708	0.27236	4.20728	2.58E-05	0.00015	TRUE	Gemin2
Gm10076	24.31332	1.678387028	0.63592	2.63931	0.00831	0.02382	TRUE	Gm10076
Pop5	327.4345	1.270324695	0.25163	5.04833	4.46E-07	3.97E-06	TRUE	Pop5
Pyroxd2	80.97265	2.455408628	0.36392	6.74715	1.51E-11	2.90E-10	TRUE	Pyroxd2
Pantr1	1476.375	1.85119866	0.3012	6.14605	7.94E-10	1.18E-08	TRUE	Pantr1
Gm10080	26.52051	2.281442751	0.63791	3.57645	0.00035	0.00152	TRUE	Gm10080
Tor3a	35.18896	2.010858776	0.57	3.52784	0.00042	0.00179	TRUE	Tor3a
Mfap2	1302.042	1.454168246	0.29888	4.86532	1.14E-06	9.25E-06	TRUE	Mfap2
Eno3	835.5503	1.922377856	0.47222	4.07092	4.68E-05	0.00026	TRUE	Eno3
Nr1h2	2291.65	1.618730059	0.34883	4.64051	3.48E-06	2.51E-05	TRUE	Nr1h2
Hist1h4i	248.7945	1.652033289	0.27267	6.05866	1.37E-09	1.95E-08	TRUE	Hist1h4i
Plaat3	27.96718	3.56986301	0.5498	6.49299	8.41E-11	1.43E-09	TRUE	Plaat3
Hist1h4c	35.92206	3.608893835	0.79835	4.52046	6.17E-06	4.20E-05	TRUE	Hist1h4c
Mrps9	598.9769	1.200990783	0.21024	5.71251	1.11E-08	1.38E-07	TRUE	Mrps9
Csmd1	138.2734	1.532959222	0.2494	6.14651	7.92E-10	1.18E-08	TRUE	Csmd1
Hist1h4h	44.00938	2.700543048	0.7061	3.82461	0.00013	0.00064	TRUE	Hist1h4h
Clasrp	2586.66	1.521451606	0.31667	4.80448	1.55E-06	1.22E-05	TRUE	Clasrp
Stk19	867.6391	1.523302216	0.40683	3.74434	0.00018	0.00086	TRUE	Stk19
Exosc5	372.9346	1.224025772	0.28566	4.28492	1.83E-05	0.00011	TRUE	Exosc5
Cxcl12	78.58575	1.718328354	0.57542	2.9862	0.00282	0.00954	TRUE	Cxcl12
Acvr2b	3174.331	2.142772027	0.4367	4.90671	9.26E-07	7.66E-06	TRUE	Acvr2b
Hist1h4d	38.76905	1.711164257	0.42565	4.02008	5.82E-05	0.00031	TRUE	Hist1h4d
Sox21	243.4374	3.019950238	0.47057	6.41769	1.38E-10	2.30E-09	TRUE	Sox21
Krt5	8.713601	8.008643571	1.52066	5.26657	1.39E-07	1.38E-06	TRUE	Krt5
Cep128	188.8454	1.628975349	0.26685	6.10439	1.03E-09	1.50E-08	TRUE	Cep128
Adgrg5	12.18551	5.516987855	1.41116	3.90955	9.25E-05	0.00047	TRUE	Adgrg5
U2af1	1733.558	1.23546349	0.20917	5.90643	3.50E-09	4.66E-08	TRUE	U2af1
Hist1h2ab	16.51875	3.372552403	0.77832	4.3331	1.47E-05	9.22E-05	TRUE	Hist1h2ab
Tmem91	83.62897	1.87839378	0.45186	4.15699	3.22E-05	0.00019	TRUE	Tmem91
Ppp1r1b	38.85888	6.625886751	0.881	7.5209	5.44E-14	1.43E-12	TRUE	Ppp1r1b
Cyp2d22	79.33789	2.673955818	0.32264	8.28777	1.15E-16	4.17E-15	TRUE	Cyp2d22
Bod1l	2371.583	1.489251635	0.35193	4.23167	2.32E-05	0.00014	TRUE	Bod1l
Akr1b10	79.82456	1.114410031	0.42206	2.64042	0.00828	0.02376	TRUE	Akr1b10
Ttr	27.57843	5.09307207	1.07022	4.75892	1.95E-06	1.49E-05	TRUE	Ttr
Rufy4	53.83802	3.307394237	0.6659	4.96681	6.81E-07	5.82E-06	TRUE	Rufy4
Myl1	11.87818	3.308712392	1.01329	3.26532	0.00109	0.00417	TRUE	Myl1
Suclg2	130.6423	1.193880101	0.494	2.41675	0.01566	0.04056	TRUE	Suclg2
Patj	267.6549	1.30958033	0.2459	5.3257	1.01E-07	1.03E-06	TRUE	Patj
Ccdc62	94.80099	1.245881847	0.35138	3.54568	0.00039	0.00169	TRUE	Ccdc62
Rcc1l	523.2592	1.762488342	0.28051	6.28309	3.32E-10	5.22E-09	TRUE	Rcc1l

Hist1h2af	14.88027	2.006717037	0.72752	2.75831	0.00581	0.01764	TRUE	Hist1h2af
Pgghg	241.9524	1.094046338	0.30714	3.56206	0.00037	0.0016	TRUE	Pgghg
Ccn6	12.03402	1.834123048	0.76635	2.39332	0.0167	0.04277	TRUE	Ccn6
Ifnlr1	10.18058	2.137060551	0.83679	2.55386	0.01065	0.0294	TRUE	Ifnlr1
Tnfaip8	59.85078	1.458477794	0.40941	3.56242	0.00037	0.00159	TRUE	Tnfaip8
Erbb2	309.9081	2.697752756	0.30911	8.72762	2.60E-18	1.12E-16	TRUE	Erbb2
Borcs7	461.7371	1.271849504	0.37171	3.42159	0.00062	0.00254	TRUE	Borcs7
Nsl1	434.2397	1.868479687	0.20908	8.93658	4.01E-19	1.89E-17	TRUE	Nsl1
430002A10R	20.45134	1.670103438	0.55826	2.99163	0.00277	0.00941	TRUE	9430002A10Rik
Ppox	1279.384	1.684069028	0.32443	5.19087	2.09E-07	1.99E-06	TRUE	Ppox
Tcp11	49.47227	1.732196216	0.53804	3.21948	0.00128	0.0048	TRUE	Tcp11
Phactr2	255.5175	1.528564099	0.34276	4.45963	8.21E-06	5.44E-05	TRUE	Phactr2
Impdh2	1100.977	1.010429718	0.16734	6.03817	1.56E-09	2.19E-08	TRUE	Impdh2
Mtap	213.2421	1.043135484	0.39591	2.63479	0.00842	0.0241	TRUE	Mtap
Nrg1	533.2786	2.126886874	0.26157	8.1312	4.25E-16	1.44E-14	TRUE	Nrg1
Zfp993	49.02026	3.814873576	0.72311	5.27568	1.32E-07	1.32E-06	TRUE	Zfp993
Naa15	7274.112	2.213563043	0.4219	5.2466	1.55E-07	1.52E-06	TRUE	Naa15
B9d2	226.2398	1.035333748	0.3106	3.33335	0.00086	0.00337	TRUE	B9d2
Syne2	3411.373	1.232447349	0.20216	6.09646	1.08E-09	1.57E-08	TRUE	Syne2
Rps15	12111.94	1.461677836	0.37604	3.88703	0.0001	0.00051	TRUE	Rps15
Snrnp70	13383.92	1.085127141	0.27226	3.98557	6.73E-05	0.00036	TRUE	Snrnp70
Ccdc102a	218.4207	2.641041702	0.34836	7.58145	3.42E-14	9.26E-13	TRUE	Ccdc102a
Unc5d	371.7617	3.013270983	0.3058	9.85371	6.61E-23	4.54E-21	TRUE	Unc5d
Slc22a21	32.98777	1.417258658	0.54204	2.61467	0.00893	0.02533	TRUE	Slc22a21
Zbtb18	2000.023	1.390435049	0.28904	4.81059	1.50E-06	1.18E-05	TRUE	Zbtb18
Sfxn4	148.5373	1.223607607	0.29512	4.14615	3.38E-05	0.00019	TRUE	Sfxn4
Sp3os	40.66829	1.039608041	0.42043	2.47273	0.01341	0.03565	TRUE	Sp3os
Gm10136	42.0607	2.399843054	0.57989	4.13845	3.50E-05	0.0002	TRUE	Gm10136
Utp14a	809.9695	1.620183843	0.31101	5.20939	1.89E-07	1.82E-06	TRUE	Utp14a
Gpatch1	483.1472	1.097330201	0.30903	3.55085	0.00038	0.00166	TRUE	Gpatch1
Ppcdc	300.0677	1.399170763	0.26806	5.21968	1.79E-07	1.73E-06	TRUE	Ppcdc
Rps6-ps1	54.63352	2.793530278	0.60126	4.64614	3.38E-06	2.45E-05	TRUE	Rps6-ps1
AA0111838	1060.692	1.471415253	0.34711	4.23911	2.24E-05	0.00013	TRUE	CAAA01118383.1
Mro	30.99956	1.837692331	0.61083	3.00854	0.00263	0.00897	TRUE	Mro
Mtx1	448.5556	1.321760981	0.30784	4.29362	1.76E-05	0.00011	TRUE	Mtx1
Fbln2	81.18066	1.220110309	0.33001	3.69715	0.00022	0.00101	TRUE	Fbln2
Cenpj	277.3633	1.041357488	0.25242	4.12543	3.70E-05	0.00021	TRUE	Cenpj
430402I18R	36.90752	3.569861478	0.53191	6.71137	1.93E-11	3.66E-10	TRUE	4430402I18Rik
Hist1h4k	38.00021	3.746261917	0.48888	7.66291	1.82E-14	5.10E-13	TRUE	Hist1h4k
Siva1	314.415	1.323997598	0.31979	4.14021	3.47E-05	0.0002	TRUE	Siva1
mt-Rnr1	57649.89	2.920609269	0.51887	5.62882	1.81E-08	2.15E-07	TRUE	mt-Rnr1
mt-Rnr2	125162.7	2.924264519	0.5403	5.41229	6.22E-08	6.66E-07	TRUE	mt-Rnr2
mt-Nd1	51109.44	1.638926756	0.35068	4.67356	2.96E-06	2.18E-05	TRUE	mt-Nd1
mt-Nd2	23593.54	1.43107105	0.33488	4.27343	1.92E-05	0.00012	TRUE	mt-Nd2
mt-Co1	181008.1	1.381950528	0.32509	4.25096	2.13E-05	0.00013	TRUE	mt-Co1
mt-Co2	293.1239	1.023036594	0.37956	2.69534	0.00703	0.02066	TRUE	mt-Co2
mt-Co3	385.6005	1.572491802	0.34031	4.62082	3.82E-06	2.74E-05	TRUE	mt-Co3
mt-Nd3	323.0157	1.555748891	0.2508	6.20322	5.53E-10	8.45E-09	TRUE	mt-Nd3
mt-Nd4	33797.44	1.541888032	0.32983	4.67476	2.94E-06	2.17E-05	TRUE	mt-Nd4
mt-Nd6	9832.866	1.622009088	0.36728	4.41622	1.00E-05	6.51E-05	TRUE	mt-Nd6
mt-Cytb	80937.53	1.462400062	0.33107	4.41717	1.00E-05	6.49E-05	TRUE	mt-Cytb
mt-Tp	226.5884	1.030344127	0.36189	2.8471	0.00441	0.01393	TRUE	mt-Tp
Gm25131	18.09729	2.166508607	0.67224	3.22282	0.00127	0.00475	TRUE	Gm25131
Gm22299	6.782999	3.241479364	0.96483	3.35965	0.00078	0.0031	TRUE	Gm22299
Gm22685	9.417222	2.190389867	0.80576	2.71842	0.00656	0.01952	TRUE	Gm22685
Gm24920	54.46574	2.459896262	0.6857	3.58743	0.00033	0.00147	TRUE	Gm24920
Gm24406	7.906973	2.381797821	0.89692	2.65554	0.00792	0.02287	TRUE	Gm24406
Rn7sk	2633.708	3.165511402	0.5576	5.67704	1.37E-08	1.67E-07	TRUE	Rn7sk
Snord104	34.0795	1.128151283	0.45223	2.49461	0.01261	0.03382	TRUE	Snord104
Snora31	33.44291	1.337413945	0.49891	2.68067	0.00735	0.02142	TRUE	Snora31
Gm22581	25.29654	2.368746977	0.50933	4.65074	3.31E-06	2.40E-05	TRUE	Gm22581
Gm23346	29.2827	3.271998969	0.68566	4.77204	1.82E-06	1.40E-05	TRUE	Gm23346
Gm25517	32.53494	3.404973082	0.5866	5.80463	6.45E-09	8.32E-08	TRUE	Gm25517
Snord35a	21.31159	2.585443164	0.64384	4.01568	5.93E-05	0.00032	TRUE	Snord35a
Gm25630	11.56079	5.383584428	1.26059	4.27067	1.95E-05	0.00012	TRUE	Gm25630
mt-Nd4l	56.08694	1.540721192	0.40399	3.81373	0.00014	0.00067	TRUE	mt-Nd4l
Tacc1	1201.035	2.017221692	0.17076	11.8133	3.33E-32	6.04E-30	TRUE	Tacc1
Mup3	4.686974	6.885996706	2.75838	2.49639	0.01255	0.03369	TRUE	Mup3
Arid3c	4.463138	3.336166053	1.34075	2.48827	0.01284	0.03432	TRUE	Arid3c
Trim12a	16.45603	1.497740526	0.64291	2.32963	0.01983	0.04929	TRUE	Trim12a
Zfyve26	550.1808	1.10293138	0.22968	4.80212	1.57E-06	1.23E-05	TRUE	Zfyve26
Hmgn3	433.6777	2.05428827	0.29219	7.03058	2.06E-12	4.47E-11	TRUE	Hmgn3
Zfp932	611.5474	1.14048477	0.17626	6.47038	9.78E-11	1.64E-09	TRUE	Zfp932
Tecpr1	2594.462	1.482805305	0.29669	4.99775	5.80E-07	5.04E-06	TRUE	Tecpr1
Cldn9	4.519763	7.094051303	1.97596	3.59019	0.00033	0.00146	TRUE	Cldn9

Gm10180	12.32115	2.004599423	0.75043	2.67128	0.00756	0.02195	TRUE	Gm10180
Vsig10	46.65612	1.39162076	0.59496	2.33902	0.01933	0.0483	TRUE	Vsig10
H2-K2	2.733034	5.418675593	1.98574	2.72879	0.00636	0.01903	TRUE	H2-K2
Ppp1r3c	25.65384	2.646622761	0.84595	3.12857	0.00176	0.00631	TRUE	Ppp1r3c
Lyar	730.3838	1.51904978	0.35536	4.27462	1.91E-05	0.00012	TRUE	Lyar
Trmt2b	86.96965	1.829128243	0.49161	3.72071	0.0002	0.00093	TRUE	Trmt2b
Hmx1	5.535929	7.096510597	2.13688	3.32097	0.0009	0.0035	TRUE	Hmx1
Hist1h4j	35.33263	2.87777648	0.72497	3.96949	7.20E-05	0.00038	TRUE	Hist1h4j
Gm10222	33.50865	2.140740515	0.65188	3.28396	0.00102	0.00393	TRUE	Gm10222
Rpl31-ps8	1156.372	1.468255791	0.34389	4.26958	1.96E-05	0.00012	TRUE	Rpl31-ps8
Map7d3	13.49408	2.153868573	0.72834	2.95722	0.0031	0.01033	TRUE	Map7d3
Zfp991	117.6524	2.389984551	0.44943	5.31778	1.05E-07	1.07E-06	TRUE	Zfp991
Cenpm	477.82	2.46201505	0.31816	7.73839	1.01E-14	2.93E-13	TRUE	Cenpm
Ccdc134	695.1357	1.65435296	0.3283	5.03918	4.68E-07	4.15E-06	TRUE	Ccdc134
Ninl	929.7201	2.946236821	0.25194	11.6943	1.36E-31	2.37E-29	TRUE	Ninl
Pick1	1015.238	1.871550458	0.38967	4.80285	1.56E-06	1.23E-05	TRUE	Pick1
Lgals1	46.89237	1.133624907	0.37599	3.01503	0.00257	0.0088	TRUE	Lgals1
Ddrgk1	731.1904	1.052247866	0.32469	3.24077	0.00119	0.0045	TRUE	Ddrgk1
Spr-ps1	15.9126	3.807863131	0.96984	3.92629	8.63E-05	0.00044	TRUE	Spr-ps1
Aup1	1612.875	2.172606362	0.3686	5.89425	3.76E-09	4.99E-08	TRUE	Aup1
Cep152	473.9021	2.004750366	0.22997	8.71754	2.84E-18	1.21E-16	TRUE	Cep152
Zfp467	364.3473	1.066247324	0.26222	4.06619	4.78E-05	0.00026	TRUE	Zfp467
Celsr2	5499.901	1.181733206	0.27348	4.32102	1.55E-05	9.67E-05	TRUE	Celsr2
Psrc1	1449.421	2.468798452	0.26463	9.32941	1.06E-20	6.01E-19	TRUE	Psrc1
Clk2	1785.16	1.160270042	0.26813	4.32723	1.51E-05	9.44E-05	TRUE	Clk2
Syt11	17295.32	1.910179379	0.42274	4.51852	6.23E-06	4.23E-05	TRUE	Syt11
Zfp114	60.03601	1.185273619	0.47448	2.49806	0.01249	0.03361	TRUE	Zfp114
Hist1h3a	42.2912	2.120739968	0.60913	3.48158	0.0005	0.00209	TRUE	Hist1h3a
Hist1h2bf	15.64021	3.248705699	0.79586	4.08201	4.46E-05	0.00025	TRUE	Hist1h2bf
Hist1h2ae	34.63988	1.258890826	0.48501	2.59562	0.00944	0.02655	TRUE	Hist1h2ae
Hist1h3e	47.84167	2.294400463	0.60241	3.80869	0.00014	0.00068	TRUE	Hist1h3e
Hist1h2bj	43.2747	1.806099175	0.61758	2.92447	0.00345	0.01132	TRUE	Hist1h2bj
Hist1h2ag	32.72941	1.819824049	0.49318	3.68997	0.00022	0.00104	TRUE	Hist1h2ag
Hist1h4n	7.991345	2.725254584	1.16022	2.34891	0.01883	0.04723	TRUE	Hist1h4n
Hist1h4m	12.72534	3.398858421	0.9325	3.64489	0.00027	0.00121	TRUE	Hist1h4m
Hist1h3c	103.921	1.647195452	0.41669	3.95303	7.72E-05	0.0004	TRUE	Hist1h3c
Tmem100	4.602307	3.170805372	1.23339	2.57081	0.01015	0.02817	TRUE	Tmem100
Slfn9	215.9433	1.853803231	0.28569	6.48886	8.65E-11	1.46E-09	TRUE	Slfn9
Gm10277	337.7018	1.695150618	0.37566	4.51243	6.41E-06	4.34E-05	TRUE	Gm10277
Atxn1l	584.675	1.262405537	0.44283	2.85076	0.00436	0.01379	TRUE	Atxn1l
Spdl1	198.1028	1.459426279	0.25176	5.79678	6.76E-09	8.67E-08	TRUE	Spdl1
Fat1	566.1818	1.558257635	0.32192	4.84048	1.30E-06	1.04E-05	TRUE	Fat1
Snora57	10.60328	2.566971349	0.736	3.48773	0.00049	0.00204	TRUE	Snora57
n-R5s33	2.980203	6.176027722	2.38583	2.58863	0.00964	0.02703	TRUE	n-R5s33
000002C10R	224.4923	1.716713156	0.30852	5.56431	2.63E-08	3.03E-07	TRUE	3000002C10Rik
Trcg1	4.939902	5.339368507	1.60019	3.33671	0.00085	0.00333	TRUE	Trcg1
930581F22R	81.8678	1.564084978	0.33113	4.72349	2.32E-06	1.74E-05	TRUE	4930581F22Rik
Rnf213	135.5028	1.740243475	0.352	4.94383	7.66E-07	6.46E-06	TRUE	Rnf213
Xntrpc	137.1675	3.91319093	0.61071	6.40756	1.48E-10	2.45E-09	TRUE	Xntrpc
Il18bp	177.6295	1.002645366	0.34363	2.91777	0.00353	0.01154	TRUE	Il18bp
Adamtsl3	24.1189	4.03990293	1.19285	3.38677	0.00071	0.00284	TRUE	Adamtsl3
Tmem132b	279.8125	1.233665178	0.4798	2.57122	0.01013	0.02814	TRUE	Tmem132b
Slc17a7	318.0354	4.997523939	0.47986	10.4145	2.13E-25	1.86E-23	TRUE	Slc17a7
Mn1	1515.248	1.678634776	0.24763	6.77877	1.21E-11	2.36E-10	TRUE	Mn1
Zfp992	51.93046	3.259151498	0.48881	6.6675	2.60E-11	4.84E-10	TRUE	Zfp992
Rpl31-ps10	7.585615	4.197523053	1.63082	2.57387	0.01006	0.02797	TRUE	Rpl31-ps10
Sars2	530.326	1.459856628	0.25302	5.76964	7.94E-09	1.00E-07	TRUE	Sars2
Trabd2b	36.68547	1.724622079	0.67632	2.55002	0.01077	0.02968	TRUE	Trabd2b
Ccdc173	153.3528	1.915600419	0.43708	4.38269	1.17E-05	7.48E-05	TRUE	Ccdc173
Gm12689	119.4892	3.664954119	0.62258	5.88677	3.94E-09	5.21E-08	TRUE	Gm12689
Impdh2-ps	299.7399	1.045953375	0.26734	3.91251	9.13E-05	0.00047	TRUE	Impdh2-ps
Zfp827	3001.233	1.015158463	0.35173	2.88622	0.0039	0.01256	TRUE	Zfp827
Nr2c2ap	31.97631	2.980640788	0.7726	3.85794	0.00011	0.00057	TRUE	Nr2c2ap
700029J07R	96.02499	1.763860045	0.47854	3.68595	0.00023	0.00105	TRUE	1700029J07Rik
Wfikkn1	67.567	1.282715072	0.44739	2.86711	0.00414	0.01322	TRUE	Wfikkn1
C1qtnf9	23.28419	3.0613052	0.95389	3.20929	0.00133	0.00495	TRUE	C1qtnf9
Map3k5	30.20873	1.731461353	0.68421	2.53058	0.01139	0.03112	TRUE	Map3k5
Gm6736	11.56372	2.956726295	0.96227	3.07266	0.00212	0.00745	TRUE	Gm6736
Dtnb	533.9172	1.39094429	0.31953	4.35306	1.34E-05	8.49E-05	TRUE	Dtnb
Hist1h2ai	18.29079	1.860491286	0.61023	3.04884	0.0023	0.00799	TRUE	Hist1h2ai
510002D24R	762.277	3.019330232	0.45753	6.59913	4.14E-11	7.48E-10	TRUE	2510002D24Rik
Tut1	966.6126	1.977885709	0.2874	6.88197	5.90E-12	1.20E-10	TRUE	Tut1
Eml3	332.7965	1.496384526	0.25361	5.9004	3.63E-09	4.83E-08	TRUE	Eml3
Mcc	160.9229	1.979650645	0.36446	5.43167	5.58E-08	6.04E-07	TRUE	Mcc
720489N17R	118.5933	1.17370335	0.29303	4.00539	6.19E-05	0.00033	TRUE	6720489N17Rik

Ccnf	913.8302	1.925992058	0.19312	9.97319	2.00E-23	1.45E-21	TRUE		Ccnf
Taf1a	279.2086	1.254710338	0.32846	3.81996	0.00013	0.00066	TRUE		Taf1a
Phf20l1	4461.054	2.304441122	0.32506	7.08935	1.35E-12	3.00E-11	TRUE		Phf20l1
Zfp783	298.0281	1.405965589	0.24017	5.85406	4.80E-09	6.27E-08	TRUE		Zfp783
Gm10399	4.966226	3.448663002	1.16888	2.95041	0.00317	0.01052	TRUE		Gm10399
Rpl37rt	205.8925	1.156787624	0.31763	3.64195	0.00027	0.00122	TRUE		Rpl37rt
Gm6712	18.66542	1.548142892	0.54921	2.81883	0.00482	0.01504	TRUE		Gm6712
430403G16F	19.29959	1.496409486	0.59333	2.52204	0.01167	0.03177	TRUE		5430403G16Rik
Acrbp	742.9729	2.317900872	0.47778	4.85143	1.23E-06	9.85E-06	TRUE		Acrbp
933439C10F	454.6757	1.583133977	0.33916	4.66784	3.04E-06	2.24E-05	TRUE		4933439C10Rik
Noxred1	61.94887	1.857538113	0.46934	3.95781	7.56E-05	0.0004	TRUE		Noxred1
Adam4	27.44039	1.889942411	0.65273	2.89546	0.00379	0.01225	TRUE		Adam4
Gm15401	2.253883	6.099164946	2.32259	2.62601	0.00864	0.02465	TRUE		Gm15401
130019P16F	47.87225	1.920335982	0.5417	3.54503	0.00039	0.00169	TRUE		9130019P16Rik
Gm1141	36.09563	2.146361042	0.41568	5.16355	2.42E-07	2.27E-06	TRUE		Gm1141
Gm10478	214.6928	1.896777558	0.40153	4.72392	2.31E-06	1.74E-05	TRUE		Gm10478
500004C02F	107.8366	2.613901657	0.3743	6.98341	2.88E-12	6.13E-11	TRUE		2500004C02Rik
Wdr90	1509.334	2.054618192	0.24742	8.30402	1.01E-16	3.66E-15	TRUE		Wdr90
330041H03F	119.8011	1.837101678	0.31828	5.77194	7.84E-09	9.90E-08	TRUE		D330041H03Rik
Pnldc1	96.88668	1.538591574	0.43763	3.51573	0.00044	0.00186	TRUE		Pnldc1
730003I15R	36.2674	1.29218685	0.42555	3.03647	0.00239	0.00829	TRUE		D730003I15Rik
130036L24F	34.5504	1.377548702	0.53682	2.56612	0.01028	0.02851	TRUE		C130036L24Rik
Fbxo36	38.23908	1.462282869	0.4434	3.29791	0.00097	0.00377	TRUE		Fbxo36
Gm10563	51.35303	3.432703536	0.75187	4.56553	4.98E-06	3.47E-05	TRUE		Gm10563
Cenps	323.5186	2.084002445	0.2613	7.97548	1.52E-15	4.91E-14	TRUE		Cenps
Btbd19	54.462	1.769124663	0.49398	3.58137	0.00034	0.0015	TRUE		Btbd19
Insyn2a	165.5555	1.26179115	0.39205	3.21846	0.00129	0.00481	TRUE		Insyn2a
Trim68	57.70593	2.393521172	0.4396	5.44474	5.19E-08	5.64E-07	TRUE		Trim68
AW146154	48.59999	2.148447539	0.47497	4.52334	6.09E-06	4.15E-05	TRUE		AW146154
430095P16F	14.96112	3.628260255	0.81082	4.47482	7.65E-06	5.10E-05	TRUE		G430095P16Rik
Ceacam1	12.16776	4.16655868	1.05064	3.96575	7.32E-05	0.00038	TRUE		Ceacam1
Ovgp1	157.7486	1.398458445	0.31532	4.43499	9.21E-06	6.03E-05	TRUE		Ovgp1
Ccdc61	664.6935	2.132743588	0.28137	7.57993	3.46E-14	9.35E-13	TRUE		Ccdc61
Spc24	1030.122	2.308207923	0.22836	10.1076	5.11E-24	4.00E-22	TRUE		Spc24
Kcng1	93.01016	3.5312969	0.38918	9.07371	1.15E-19	5.86E-18	TRUE		Kcng1
Zfas1	321.2483	1.166392493	0.20532	5.68078	1.34E-08	1.63E-07	TRUE		Zfas1
930518I15R	78.02544	1.045534562	0.39515	2.6459	0.00815	0.02343	TRUE		4930518I15Rik
430550D23F	65.53375	1.693818143	0.4738	3.57493	0.00035	0.00153	TRUE		6430550D23Rik
Zfp950	536.1268	1.02319739	0.21665	4.7228	2.33E-06	1.75E-05	TRUE		Zfp950
Slc4a11	120.1605	2.828333778	0.48493	5.83251	5.46E-09	7.10E-08	TRUE		Slc4a11
Gm4149	28.27592	2.044265777	0.7981	2.56141	0.01042	0.02885	TRUE		Gm4149
Itpripl1	96.21514	1.061350264	0.4236	2.50556	0.01223	0.03304	TRUE		Itpripl1
BC025920	287.6187	1.951665552	0.33881	5.76043	8.39E-09	1.05E-07	TRUE		BC025920
Platr25	413.8689	1.215703786	0.20571	5.90969	3.43E-09	4.58E-08	TRUE		Platr25
Zfp934	124.4473	1.289834933	0.37541	3.43582	0.00059	0.00242	TRUE		Zfp934
Zfp808	57.45385	1.231824006	0.52155	2.36185	0.01818	0.04588	TRUE		Zfp808
Fam122a	375.276	1.058644436	0.35507	2.98151	0.00287	0.00968	TRUE		Fam122a
Mir670hg	140.2326	2.769328149	0.41289	6.70711	1.99E-11	3.76E-10	TRUE		Mir670hg
Hist1h2bb	33.63819	3.16725185	0.72376	4.37613	1.21E-05	7.70E-05	TRUE		Hist1h2bb
Mrpl23-ps1	23.6657	4.46906225	1.441	3.10137	0.00193	0.00684	TRUE		Mrpl23-ps1
Rprm	468.2312	2.301481038	0.35265	6.52622	6.74E-11	1.17E-09	TRUE		Rprm
Prcd	11.309	4.288446642	1.11796	3.83597	0.00013	0.00062	TRUE		Prcd
Gm14488	81.28238	3.460163745	0.56995	6.07105	1.27E-09	1.82E-08	TRUE		Gm14488
Smim6	36.56474	1.651656939	0.61041	2.7058	0.00681	0.02011	TRUE		Smim6
Gm11681	4.857499	7.18674488	1.91186	3.75903	0.00017	0.00081	TRUE		Gm11681
Gm17151	13.03694	2.144085303	0.81472	2.63168	0.0085	0.0243	TRUE		Gm17151
Gm23935	212661.3	3.335849001	0.58122	5.73939	9.50E-09	1.19E-07	TRUE		Gm23935
Gm24270	49021.09	3.265846959	0.56356	5.79504	6.83E-09	8.73E-08	TRUE		Gm24270
Mir670	7.876897	4.591057758	1.02813	4.46544	7.99E-06	5.30E-05	TRUE		Mir670
Mir763	24.71301	5.115086209	0.94337	5.42213	5.89E-08	6.34E-07	TRUE		Mir763
Snora17	92.5794	1.715602536	0.34191	5.01775	5.23E-07	4.59E-06	TRUE		Snora17
Gm24282	39.52879	3.002313552	0.58727	5.11234	3.18E-07	2.90E-06	TRUE		Gm24282
Snord83b	9.591153	1.905676992	0.76473	2.49195	0.0127	0.03404	TRUE		Snord83b
AW011738	136.3786	1.635201456	0.44405	3.68247	0.00023	0.00106	TRUE		AW011738
Plekhn1	1464.608	1.467860989	0.25335	5.79381	6.88E-09	8.79E-08	TRUE		Plekhn1
Perm1	267.7257	1.721268606	0.35422	4.85934	1.18E-06	9.50E-06	TRUE		Perm1
Cfap74	71.69964	1.386318077	0.37364	3.7103	0.00021	0.00096	TRUE		Cfap74
Zfp984	66.52764	1.264864761	0.46601	2.71425	0.00664	0.0197	TRUE		Zfp984
Aunip	80.331	2.599228265	0.44262	5.8724	4.30E-09	5.64E-08	TRUE		Aunip
Gm11627	67.97138	5.798543089	0.59193	9.79603	1.17E-22	7.82E-21	TRUE		Gm11627
Aoc2	336.9857	1.271497146	0.30969	4.1057	4.03E-05	0.00023	TRUE		Aoc2
Cisd3	117.7208	2.136323855	0.34916	6.11845	9.45E-10	1.39E-08	TRUE		Cisd3
030028A08F	204.9437	1.149821147	0.42448	2.70879	0.00675	0.01996	TRUE		D030028A08Rik
Haus5	138.7186	2.446377072	0.32666	7.48917	6.93E-14	1.79E-12	TRUE		Haus5
Rad54b	266.469	2.370421422	0.33522	7.07116	1.54E-12	3.40E-11	TRUE		Rad54b

Zfp970	60.39889	1.447206885	0.36628	3.95111	7.78E-05	0.00041	TRUE	Zfp970
D130040H23R	171.6229	1.361689743	0.332	4.1015	4.10E-05	0.00023	TRUE	D130040H23Rik
Gm14010	13.90022	3.189846392	0.98253	3.24655	0.00117	0.00442	TRUE	Gm14010
Kcnip3	358.8997	1.866148564	0.27428	6.80391	1.02E-11	2.00E-10	TRUE	Kcnip3
BC005561	2570.089	2.867381522	0.44237	6.48187	9.06E-11	1.53E-09	TRUE	BC005561
Fam228a	48.35831	1.67493518	0.49404	3.39026	0.0007	0.00281	TRUE	Fam228a
Mphosph8	1482.435	1.556876284	0.35093	4.43643	9.15E-06	6.00E-05	TRUE	Mphosph8
Tex52	54.79092	2.627174375	0.46118	5.69669	1.22E-08	1.50E-07	TRUE	Tex52
Naa80	281.3607	1.153246911	0.29049	3.97003	7.19E-05	0.00038	TRUE	Naa80
Mroh2a	908.123	2.042091368	0.31463	6.49054	8.55E-11	1.45E-09	TRUE	Mroh2a
Gm13420	26.4377	2.62660314	0.53376	4.92092	8.61E-07	7.17E-06	TRUE	Gm13420
Kifc1	883.8628	1.980185173	0.21457	9.22867	2.74E-20	1.48E-18	TRUE	Kifc1
Zbtb9	68.37241	1.664558234	0.48279	3.44781	0.00057	0.00233	TRUE	Zbtb9
Fam71f2	31.12761	2.462774068	0.66422	3.70778	0.00021	0.00097	TRUE	Fam71f2
Rps19-ps3	101.2118	2.294643994	0.41222	5.56655	2.60E-08	2.99E-07	TRUE	Rps19-ps3
Gm12260	26.57685	2.897394293	0.60729	4.77099	1.83E-06	1.41E-05	TRUE	Gm12260
Gm14239	39.89189	3.395650411	0.71817	4.72817	2.27E-06	1.71E-05	TRUE	Gm14239
Gm9143	12.95367	3.326645461	1.06635	3.11965	0.00181	0.00647	TRUE	Gm9143
Gm11625	28.06361	5.16108431	1.04175	4.95423	7.26E-07	6.17E-06	TRUE	Gm11625
Rpl10-ps6	24.63105	3.633351305	0.73973	4.91175	9.03E-07	7.49E-06	TRUE	Rpl10-ps6
Rpl38-ps2	109.2986	1.172114309	0.35902	3.26478	0.0011	0.00417	TRUE	Rpl38-ps2
Hmgb1-ps6	20.83933	1.930571997	0.64797	2.9794	0.00289	0.00974	TRUE	Hmgb1-ps6
Gm12846	98.0541	3.657773429	0.76729	4.76716	1.87E-06	1.43E-05	TRUE	Gm12846
Gm15427	1796.916	1.162756273	0.325	3.57772	0.00035	0.00152	TRUE	Gm15427
Gm12568	31.61459	4.596454285	0.93303	4.92635	8.38E-07	6.99E-06	TRUE	Gm12568
Gm15268	3.473428	6.392210575	1.96706	3.24962	0.00116	0.00437	TRUE	Gm15268
Gm15791	5.743023	5.313355462	1.61953	3.2808	0.00104	0.00397	TRUE	Gm15791
Gm14021	82.20883	2.580014342	0.61516	4.19407	2.74E-05	0.00016	TRUE	Gm14021
Gm13878	10.5885	2.364571709	0.82316	2.87257	0.00407	0.01301	TRUE	Gm13878
Rps13-ps4	48.88406	1.39573354	0.37672	3.70501	0.00021	0.00098	TRUE	Rps13-ps4
Gm13689	148.89	3.761291033	0.67872	5.54178	2.99E-08	3.41E-07	TRUE	Gm13689
Gm12411	52.05556	1.585372599	0.558	2.84117	0.00449	0.01415	TRUE	Gm12411
Gm13717	90.94633	4.246794292	0.55193	7.69444	1.42E-14	4.05E-13	TRUE	Gm13717
Gm15953	35.49921	1.671534644	0.61943	2.69849	0.00697	0.02052	TRUE	Gm15953
Gm13423	112.6476	2.40833527	0.38325	6.28391	3.30E-10	5.20E-09	TRUE	Gm13423
Gm13785	27.00617	2.139362188	0.59516	3.5946	0.00032	0.00143	TRUE	Gm13785
Gm15495	17.43846	4.131864411	1.48183	2.78835	0.0053	0.01632	TRUE	Gm15495
Anp32b-ps1	5.377922	2.484267284	1.05026	2.36537	0.01801	0.04552	TRUE	Anp32b-ps1
Rps11-ps2	23.70132	1.280841612	0.5393	2.37502	0.01755	0.04458	TRUE	Rps11-ps2
Rpl23a-ps2	45.15319	2.616639844	0.48669	5.37636	7.60E-08	7.97E-07	TRUE	Rpl23a-ps2
Gm7153	63.1828	1.902174339	0.46675	4.07537	4.59E-05	0.00025	TRUE	Gm7153
Gm11196	22.00678	1.891193243	0.58537	3.23079	0.00123	0.00464	TRUE	Gm11196
Gm14276	120.412	1.76536573	0.287	6.15112	7.69E-10	1.15E-08	TRUE	Gm14276
Gm715	44.94618	2.398167406	0.47925	5.00401	5.61E-07	4.89E-06	TRUE	Gm715
Gm16523	83.32904	1.950103185	0.45442	4.29143	1.78E-05	0.00011	TRUE	Gm16523
Gm9670	46.79226	1.955826253	0.74605	2.62158	0.00875	0.02492	TRUE	Gm9670
Gm14036	68.19295	4.075749173	0.56355	7.23226	4.75E-13	1.11E-11	TRUE	Gm14036
Fau-ps1	13.05635	2.044256669	0.78142	2.61607	0.00889	0.02525	TRUE	Fau-ps1
Rps6-ps3	44.52129	2.950818018	0.74454	3.9633	7.39E-05	0.00039	TRUE	Rps6-ps3
Gm13349	14.7884	2.358359741	0.76225	3.09394	0.00198	0.00699	TRUE	Gm13349
Rpl31-ps6	13.99346	1.770772442	0.65733	2.69389	0.00706	0.02072	TRUE	Rpl31-ps6
Gm12987	16.32499	2.147646374	0.85071	2.52453	0.01159	0.03157	TRUE	Gm12987
Gm12366	72.02563	2.242491594	0.48113	4.6609	3.15E-06	2.30E-05	TRUE	Gm12366
Gm14537	21.2895	3.454395361	0.70896	4.87249	1.10E-06	8.96E-06	TRUE	Gm14537
Gm13525	21.09948	2.602332892	0.77065	3.37681	0.00073	0.00293	TRUE	Gm13525
Gm12934	10.38552	2.096208861	0.79972	2.62117	0.00876	0.02495	TRUE	Gm12934
Gm2810	121.9537	2.870749857	0.60185	4.7699	1.84E-06	1.41E-05	TRUE	Gm2810
Gm12618	10.86954	4.744463827	1.62591	2.91803	0.00352	0.01153	TRUE	Gm12618
Gm11736	7.159248	3.439226606	1.24215	2.76876	0.00563	0.01717	TRUE	Gm11736
Gm15385	29.24651	2.531497005	0.76616	3.30414	0.00095	0.0037	TRUE	Gm15385
Gm12917	32.43799	1.295300356	0.52808	2.45285	0.01417	0.03733	TRUE	Gm12917
Gm5303	8.845793	6.750078498	1.66528	4.05343	5.05E-05	0.00028	TRUE	Gm5303
Gm13828	4.385131	6.795488748	1.76849	3.84255	0.00012	0.0006	TRUE	Gm13828
Gm9432	11.67387	2.37113017	0.97808	2.42427	0.01534	0.03985	TRUE	Gm9432
Gm7831	8.014739	6.651134505	1.53189	4.34178	1.41E-05	8.89E-05	TRUE	Gm7831
Gm13868	31.8101	1.459015875	0.4925	2.96245	0.00305	0.01019	TRUE	Gm13868
Gm12653	16.39951	3.778944039	1.08075	3.49659	0.00047	0.00198	TRUE	Gm12653
Gm5466	233.3973	1.092324507	0.33341	3.27622	0.00105	0.00403	TRUE	Gm5466
Gm14776	82.35062	2.230314744	0.61727	3.61319	0.0003	0.00135	TRUE	Gm14776
Gm15267	10.53506	2.157544132	0.85229	2.53147	0.01136	0.03106	TRUE	Gm15267
Gm13407	20.71248	1.775619215	0.63971	2.77567	0.00551	0.01686	TRUE	Gm13407
Gm16016	12.03622	1.892925954	0.77415	2.44515	0.01448	0.03794	TRUE	Gm16016
Gm16007	4.005281	4.753894547	1.61014	2.95247	0.00315	0.01046	TRUE	Gm16007
Gm3828	5.763552	4.06610939	1.60167	2.53868	0.01113	0.03051	TRUE	Gm3828
Gm15712	53.08697	2.385353431	0.46427	5.13783	2.78E-07	2.58E-06	TRUE	Gm15712

Gm12577	62.13507	6.833173215	1.90371	3.58939	0.00033	0.00146	TRUE	Gm12577
Gm15696	44.63178	2.605338884	0.55865	4.66366	3.11E-06	2.27E-05	TRUE	Gm15696
Gm11761	36.43613	2.739569485	0.62346	4.3941	1.11E-05	7.15E-05	TRUE	Gm11761
Gm14843	47.80021	2.667201197	0.60764	4.38944	1.14E-05	7.28E-05	TRUE	Gm14843
Gm12346	717.2511	2.23045782	0.44653	4.99506	5.88E-07	5.10E-06	TRUE	Gm12346
Gm14049	25.4436	2.333806207	0.72513	3.21845	0.00129	0.00481	TRUE	Gm14049
Gm13302	12.33568	3.586679992	0.79876	4.4903	7.11E-06	4.77E-05	TRUE	Gm13302
Gm6276	24.4773	4.879550464	0.82323	5.92732	3.08E-09	4.15E-08	TRUE	Gm6276
Gm14984	40.78248	5.919994523	1.14686	5.1619	2.44E-07	2.29E-06	TRUE	Gm14984
Esrp2	6.577181	3.724821527	1.5619	2.38481	0.01709	0.04359	TRUE	Esrp2
Gm9085	45.58349	1.848191655	0.57797	3.19773	0.00139	0.00513	TRUE	Gm9085
Gm12696	66.41807	1.074130989	0.38911	2.76048	0.00577	0.01755	TRUE	Gm12696
Gm15488	3.829158	6.628527277	2.2107	2.99838	0.00271	0.00923	TRUE	Gm15488
Gm11702	24.32199	3.667726529	0.80436	4.55983	5.12E-06	3.55E-05	TRUE	Gm11702
Gm5228	4.872357	7.020413796	2.15036	3.26476	0.0011	0.00417	TRUE	Gm5228
Gm11599	8.772439	2.792098866	1.0837	2.57646	0.00998	0.02782	TRUE	Gm11599
Rpl3-ps1	520.5969	1.237390458	0.33109	3.73738	0.00019	0.00088	TRUE	Rpl3-ps1
Znf41-ps	86.17842	3.011517898	0.48339	6.23001	4.66E-10	7.19E-09	TRUE	Znf41-ps
Gm13370	82.74772	2.232301626	0.46457	4.80512	1.55E-06	1.21E-05	TRUE	Gm13370
Gm15666	118.4748	1.023637941	0.3534	2.89656	0.00377	0.01221	TRUE	Gm15666
Gm11510	121.7386	2.082242647	0.49324	4.22156	2.43E-05	0.00014	TRUE	Gm11510
Mir1907	7.51734	3.405588533	1.1092	3.07031	0.00214	0.0075	TRUE	Mir1907
n-R5s188	2.171063	5.09055591	2.11467	2.40726	0.01607	0.04142	TRUE	n-R5s188
Gm24371	64.59831	3.391117569	0.63861	5.31019	1.10E-07	1.11E-06	TRUE	Gm24371
Gm22613	37.07059	4.600301941	0.53033	8.67444	4.16E-18	1.74E-16	TRUE	Gm22613
Gm25291	12.64867	1.649320875	0.64466	2.55844	0.01051	0.02906	TRUE	Gm25291
Gm15879	70.04278	2.778197123	0.62176	4.46829	7.88E-06	5.24E-05	TRUE	Gm15879
Ino80dos	51.0636	1.268513862	0.45157	2.80912	0.00497	0.01543	TRUE	Ino80dos
Gm11267	11.77634	2.801887046	1.10106	2.54471	0.01094	0.03007	TRUE	Gm11267
Gm12289	38.91165	1.969034149	0.60386	3.26076	0.00111	0.00423	TRUE	Gm12289
Gm15594	26.49376	2.622243175	0.67372	3.8922	9.93E-05	0.0005	TRUE	Gm15594
430010I23R	30.8249	1.946830817	0.67972	2.86415	0.00418	0.01332	TRUE	B430010I23Rik
Gm16267	4.277682	3.393830313	1.40534	2.41495	0.01574	0.04075	TRUE	Gm16267
Gm13134	10.33363	8.148135346	1.62451	5.01575	5.28E-07	4.63E-06	TRUE	Gm13134
230035I16R	27.91607	2.256714765	0.64629	3.49177	0.00048	0.00202	TRUE	C230035I16Rik
Slc2a4rg-ps	594.4222	1.427782396	0.33914	4.21002	2.55E-05	0.00015	TRUE	Slc2a4rg-ps
Gm11750	10.87259	2.627012382	0.87479	3.00303	0.00267	0.00911	TRUE	Gm11750
Gm16024	39.12932	2.290613691	0.608	3.76747	0.00016	0.00079	TRUE	Gm16024
Prdm16os	62.53031	6.931394728	0.73036	9.4904	2.30E-21	1.37E-19	TRUE	Prdm16os
931413K12R	146.4258	2.067418969	0.49633	4.16538	3.11E-05	0.00018	TRUE	4931413K12Rik
Snhg15	78.50086	1.696260592	0.29957	5.66227	1.49E-08	1.81E-07	TRUE	Snhg15
Gm6542	140.5674	2.591365418	0.52167	4.96747	6.78E-07	5.80E-06	TRUE	Gm6542
Gm12195	38.19707	1.370418389	0.56389	2.4303	0.01509	0.03931	TRUE	Gm12195
Nr6a1os	38.71436	1.215624934	0.5049	2.40764	0.01606	0.04139	TRUE	Nr6a1os
Gm13974	4.009309	4.773393622	1.70981	2.79176	0.00524	0.01618	TRUE	Gm13974
610206C17R	6.371268	4.194172078	1.56253	2.68422	0.00727	0.02123	TRUE	2610206C17Rik
Gm12150	20.59459	2.215482018	0.65125	3.40187	0.00067	0.00271	TRUE	Gm12150
930545L23R	6.168762	4.029464056	1.36313	2.95604	0.00312	0.01036	TRUE	4930545L23Rik
Dmrta2os	70.29405	4.141502154	0.4925	8.40919	4.13E-17	1.58E-15	TRUE	Dmrta2os
130006D01R	70.86068	1.307994943	0.52366	2.49782	0.0125	0.03362	TRUE	E130006D01Rik
Gm15965	13.74006	2.199129706	0.73714	2.98335	0.00285	0.00962	TRUE	Gm15965
Gm15445	57.13334	2.224578154	0.52083	4.27121	1.94E-05	0.00012	TRUE	Gm15445
010003L21R	145.437	1.537546707	0.45798	3.3572	0.00079	0.00313	TRUE	3010003L21Rik
Snhg17	571.7819	1.31541425	0.27806	4.73071	2.24E-06	1.69E-05	TRUE	Snhg17
Firre	1583.547	1.470123972	0.19905	7.38563	1.52E-13	3.78E-12	TRUE	Firre
732490B19R	7.800876	3.068757426	1.07174	2.86334	0.00419	0.01335	TRUE	4732490B19Rik
Gm15398	8.840997	1.919867843	0.81278	2.3621	0.01817	0.04585	TRUE	Gm15398
930526A20R	61.75561	2.041053205	0.59788	3.41382	0.00064	0.0026	TRUE	4930526A20Rik
Gm13568	9.13312	2.941707887	1.03992	2.82879	0.00467	0.01465	TRUE	Gm13568
Gm15792	20.48228	2.948998207	0.63181	4.66755	3.05E-06	2.24E-05	TRUE	Gm15792
700123M08R	43.73656	1.589872557	0.5323	2.98677	0.00282	0.00954	TRUE	1700123M08Rik
Gm8813	9.395747	6.102925289	1.82621	3.34185	0.00083	0.00328	TRUE	Gm8813
Gm15718	29.1097	1.193774752	0.44503	2.68249	0.00731	0.02133	TRUE	Gm15718
430405H02R	363.0319	2.534059224	0.45384	5.58362	2.36E-08	2.75E-07	TRUE	5430405H02Rik
Gm15412	16.38554	2.147992026	0.7984	2.69037	0.00714	0.02092	TRUE	Gm15412
930470G03R	24.60476	1.973215473	0.67686	2.91524	0.00355	0.01162	TRUE	4930470G03Rik
Zfp703	222.4751	1.305292386	0.3301	3.95427	7.68E-05	0.0004	TRUE	Zfp703
Gm13052	73.46087	3.66524428	0.55881	6.55901	5.42E-11	9.55E-10	TRUE	Gm13052
Gm15638	10.5226	2.743312011	0.82663	3.31866	0.0009	0.00353	TRUE	Gm15638
Gm12781	8.438448	3.725237047	1.3284	2.80431	0.00504	0.01564	TRUE	Gm12781
Gm15556	9.395399	4.504968642	1.09673	4.10764	4.00E-05	0.00022	TRUE	Gm15556
610307P16R	329.2217	1.413072673	0.28507	4.95699	7.16E-07	6.09E-06	TRUE	2610307P16Rik
Gm13403	4.375085	5.786680051	1.68103	3.44235	0.00058	0.00237	TRUE	Gm13403
Gm2788	5.696483	2.588828596	1.10677	2.33908	0.01933	0.0483	TRUE	Gm2788
Gm7805	2.785737	4.602990829	1.86589	2.46691	0.01363	0.03613	TRUE	Gm7805

Gm11832	18.59962	1.789395057	0.68044	2.62977	0.00854	0.02441	TRUE	Gm11832
Bach2os	18.14054	2.134501606	0.64732	3.29746	0.00098	0.00377	TRUE	Bach2os
430041J12R	89.68208	2.25828161	0.38891	5.80677	6.37E-09	8.23E-08	TRUE	9430041J12Rik
Gm13609	4.471651	5.271695043	1.77484	2.97023	0.00298	0.00998	TRUE	Gm13609
Gm13783	4.646045	5.946359048	2.01299	2.95399	0.00314	0.01042	TRUE	Gm13783
Gm16599	15.4585	2.85277183	0.87664	3.25421	0.00114	0.00431	TRUE	Gm16599
Snhg12	170.9515	1.321643862	0.28581	4.62427	3.76E-06	2.70E-05	TRUE	Snhg12
Gm11269	4.741647	5.880877311	2.48066	2.37069	0.01775	0.04504	TRUE	Gm11269
630016G05F	238.8482	5.336603199	0.45377	11.7606	6.23E-32	1.11E-29	TRUE	G630016G05Rik
Gm16087	7.256445	5.724780873	1.65726	3.45436	0.00055	0.00228	TRUE	Gm16087
Gm15564	705.8133	3.357093731	0.62143	5.40221	6.58E-08	6.99E-07	TRUE	Gm15564
700033N17F	68.76029	2.689470091	0.46279	5.81145	6.19E-09	8.01E-08	TRUE	2700033N17Rik
Gm13270	25.46483	3.107699034	0.86163	3.60675	0.00031	0.00138	TRUE	Gm13270
Gm12108	12.54623	2.608949915	0.72546	3.59625	0.00032	0.00143	TRUE	Gm12108
Gm11722	104.4986	2.589235771	0.52881	4.89638	9.76E-07	8.03E-06	TRUE	Gm11722
933421A08F	37.82644	3.341755587	0.55224	6.05128	1.44E-09	2.03E-08	TRUE	4933421A08Rik
Prkag2os1	103.1041	1.302436644	0.39803	3.27222	0.00107	0.00408	TRUE	Prkag2os1
Gm13778	24.39878	1.596543941	0.64901	2.45997	0.0139	0.03672	TRUE	Gm13778
Cd101	6.286949	3.2538129	1.06102	3.06667	0.00216	0.00758	TRUE	Cd101
Gm16126	10.73193	6.288800154	1.60584	3.9162	9.00E-05	0.00046	TRUE	Gm16126
Gm12064	19.58477	2.449557988	0.87503	2.79941	0.00512	0.01585	TRUE	Gm12064
Hnrnpa1l2-p	10.79829	4.004148714	1.08024	3.70672	0.00021	0.00098	TRUE	Hnrnpa1l2-ps
Gm13110	12.18885	4.685305955	0.88012	5.32349	1.02E-07	1.04E-06	TRUE	Gm13110
110082J24R	47.30486	1.806012161	0.55377	3.26132	0.00111	0.00422	TRUE	3110082J24Rik
Gm11739	128.0347	2.006228642	0.49466	4.05575	5.00E-05	0.00027	TRUE	Gm11739
Snhg20	495.3356	1.212691394	0.28679	4.2285	2.35E-05	0.00014	TRUE	Snhg20
Gm15963	50.66324	1.899541696	0.56012	3.39129	0.0007	0.0028	TRUE	Gm15963
Gm15708	80.47231	1.492535618	0.36051	4.14005	3.47E-05	0.0002	TRUE	Gm15708
Gm13404	25.32274	1.712179475	0.63456	2.69823	0.00697	0.02053	TRUE	Gm13404
Gm15903	58.44678	1.32239098	0.41527	3.18442	0.00145	0.00534	TRUE	Gm15903
Gm11823	4.686974	6.885996706	2.75838	2.49639	0.01255	0.03369	TRUE	Gm11823
Gm13889	197.1211	2.365627657	0.35599	6.64526	3.03E-11	5.56E-10	TRUE	Gm13889
130024F11R	55.3054	5.293827696	0.55176	9.59439	8.44E-22	5.20E-20	TRUE	9130024F11Rik
Eldr	84.54232	1.475067092	0.45285	3.25732	0.00112	0.00427	TRUE	Eldr
Gm15857	3.803561	3.827665403	1.55017	2.46918	0.01354	0.03595	TRUE	Gm15857
Gm15423	12.14855	4.370680553	1.07978	4.04777	5.17E-05	0.00028	TRUE	Gm15423
Emx2os	81.8856	2.715685356	0.36791	7.38141	1.57E-13	3.90E-12	TRUE	Emx2os
Gm11884	3.844106	6.734817572	1.88486	3.57311	0.00035	0.00154	TRUE	Gm11884
Gm16152	57.38539	2.560336014	0.51722	4.95016	7.42E-07	6.28E-06	TRUE	Gm16152
Plcxd2	512.6976	1.523443664	0.27829	5.47437	4.39E-08	4.85E-07	TRUE	Plcxd2
610035D17F	189.4589	2.165158412	0.25966	8.33834	7.53E-17	2.79E-15	TRUE	2610035D17Rik
Gm13832	10.72808	2.678798971	0.74434	3.59891	0.00032	0.00141	TRUE	Gm13832
810021B22F	24.61887	1.733848535	0.6988	2.48117	0.0131	0.03491	TRUE	1810021B22Rik
Scpep1os	11.9133	2.177543379	0.75296	2.89197	0.00383	0.01236	TRUE	Scpep1os
Junos	32.26055	2.700758103	0.49027	5.50876	3.61E-08	4.04E-07	TRUE	Junos
Gm15892	17.88288	1.722279699	0.59021	2.91806	0.00352	0.01153	TRUE	Gm15892
AV099323	11.4121	3.039811848	0.92847	3.27399	0.00106	0.00405	TRUE	AV099323
Gm7598	13.32598	2.235477667	0.73301	3.04971	0.00229	0.00798	TRUE	Gm7598
Gm14664	7.873069	4.763799725	1.20223	3.96248	7.42E-05	0.00039	TRUE	Gm14664
Gm11266	261.7503	3.916039568	0.49381	7.93022	2.19E-15	6.92E-14	TRUE	Gm11266
Gm12971	21.22891	2.189071674	0.69854	3.13377	0.00173	0.00621	TRUE	Gm12971
Gm15910	44.8278	3.218190624	0.46786	6.8786	6.04E-12	1.23E-10	TRUE	Gm15910
Gm13427	19.66334	3.22143848	0.8767	3.6745	0.00024	0.00109	TRUE	Gm13427
Gm15202	4.084074	5.719974842	2.05341	2.7856	0.00534	0.01643	TRUE	Gm15202
030037D09F	60.89449	1.369243027	0.36524	3.74885	0.00018	0.00084	TRUE	C030037D09Rik
Hectd2os	4.01274	3.573822909	1.41077	2.53324	0.0113	0.03092	TRUE	Hectd2os
Epb41l4aos	166.8479	1.325954374	0.29523	4.49121	7.08E-06	4.75E-05	TRUE	Epb41l4aos
Gm16121	34.89635	4.776233198	0.88325	5.40757	6.39E-08	6.81E-07	TRUE	Gm16121
230111E07F	4.734045	3.577050518	1.24918	2.86353	0.00419	0.01334	TRUE	9230111E07Rik
Gm12246	24.56616	2.698635388	0.62233	4.33635	1.45E-05	9.10E-05	TRUE	Gm12246
Gm6058	1002.048	4.569803557	0.65985	6.92557	4.34E-12	9.04E-11	TRUE	Gm6058
Tmem250-p	436.2196	1.318020049	0.25923	5.08438	3.69E-07	3.33E-06	TRUE	Tmem250-ps
Gm22442	24.06804	1.724916924	0.70171	2.45816	0.01397	0.03687	TRUE	Gm22442
Gm23232	19.96229	2.975919209	0.73412	4.0537	5.04E-05	0.00028	TRUE	Gm23232
Gm25220	28.43912	1.79657932	0.55519	3.23595	0.00121	0.00456	TRUE	Gm25220
Scarna2	131.6853	2.224566762	0.50727	4.38535	1.16E-05	7.40E-05	TRUE	Scarna2
Gm25911	841.2084	3.139591292	0.6119	5.13086	2.88E-07	2.66E-06	TRUE	Gm25911
Gm24009	78.90704	2.639789878	0.38658	6.82851	8.58E-12	1.71E-10	TRUE	Gm24009
Gm24187	4377.614	3.177234193	0.57508	5.52483	3.30E-08	3.72E-07	TRUE	Gm24187
Mir1963	11.98245	6.525577812	1.27847	5.10421	3.32E-07	3.02E-06	TRUE	Mir1963
Gm23119	9.405194	2.243642899	0.8422	2.66402	0.00772	0.02238	TRUE	Gm23119
Scarna6	7.848912	2.308521216	0.8531	2.70603	0.00681	0.0201	TRUE	Scarna6
Gm22140	2.116389	6.018135708	2.36848	2.54093	0.01106	0.03035	TRUE	Gm22140
Gm22009	60.55138	2.079430587	0.40922	5.08147	3.75E-07	3.37E-06	TRUE	Gm22009
Gm24732	7.482882	2.281748354	0.85553	2.66706	0.00765	0.0222	TRUE	Gm24732

Gm16565	14.92678	2.506315579	0.72778	3.44378	0.00057	0.00236	TRUE	Gm16565
Pitpnm2os2	14.28217	2.479168047	0.75247	3.2947	0.00099	0.0038	TRUE	Pitpnm2os2
Gm16102	12.04706	2.513748752	0.92927	2.70509	0.00683	0.02015	TRUE	Gm16102
Gm43517	42.02577	7.372850053	0.94773	7.77951	7.28E-15	2.18E-13	TRUE	Gm43517
Gm16006	9.320972	3.948033216	1.17218	3.36812	0.00076	0.00301	TRUE	Gm16006
Gm15849	21.45127	1.832678528	0.64321	2.84926	0.00438	0.01385	TRUE	Gm15849
Rnf8	361.8306	1.365103996	0.27383	4.98525	6.19E-07	5.34E-06	TRUE	Rnf8
Cmc4	57.52325	1.164415883	0.40287	2.89033	0.00385	0.01242	TRUE	Cmc4
Gm3076	3.946232	3.800111675	1.45681	2.60851	0.00909	0.02573	TRUE	Gm3076
Gm8580	30.87142	1.82324392	0.77796	2.34363	0.0191	0.04778	TRUE	Gm8580
630014O11R	2.905458	6.127967225	2.45432	2.49681	0.01253	0.03369	TRUE	D630014O11Rik
Gm15995	11.43745	3.533064905	0.95916	3.68351	0.00023	0.00106	TRUE	Gm15995
Itga10	155.5748	1.477951231	0.37222	3.97067	7.17E-05	0.00038	TRUE	Itga10
Gm14027	26.6547	4.070328736	0.84561	4.81346	1.48E-06	1.17E-05	TRUE	Gm14027
Prr22	84.81809	2.355519683	0.48076	4.89961	9.60E-07	7.91E-06	TRUE	Prr22
Mir99ahg	3050.45	2.25421252	0.36151	6.2355	4.50E-10	6.98E-09	TRUE	Mir99ahg
Rps11-ps1	167.8877	3.06612706	0.45324	6.76484	1.33E-11	2.58E-10	TRUE	Rps11-ps1
Gm4675	7.465028	2.647645528	1.11885	2.3664	0.01796	0.04544	TRUE	Gm4675
Gm17137	65.85408	2.19851534	0.52884	4.15721	3.22E-05	0.00019	TRUE	Gm17137
Gm17571	5.362847	2.694439603	1.10993	2.42758	0.0152	0.03957	TRUE	Gm17571
Tex9	320.5092	1.255576944	0.25001	5.02205	5.11E-07	4.50E-06	TRUE	Tex9
Gm8378	16.26798	4.684615671	0.82852	5.65418	1.57E-08	1.88E-07	TRUE	Gm8378
Gm17201	221.1477	1.136597967	0.30453	3.73225	0.00019	0.00089	TRUE	Gm17201
Gm3235	64.82793	1.737418683	0.39081	4.44573	8.76E-06	5.78E-05	TRUE	Gm3235
Gm17110	79.72876	3.657956429	0.50343	7.26601	3.70E-13	8.78E-12	TRUE	Gm17110
Pabpc4l	27.98931	2.610142125	0.66512	3.92432	8.70E-05	0.00045	TRUE	Pabpc4l
Gm17212	59.87546	3.611253498	0.67376	5.35986	8.33E-08	8.67E-07	TRUE	Gm17212
Gm4535	20.18362	3.336026442	0.881	3.78666	0.00015	0.00074	TRUE	Gm4535
Gm2832	4.774407	5.880935789	2.48096	2.37043	0.01777	0.04506	TRUE	Gm2832
Phf11c	68.71623	2.652240951	0.62383	4.25153	2.12E-05	0.00013	TRUE	Phf11c
Gm17036	14.49187	2.735059447	0.76576	3.57169	0.00035	0.00154	TRUE	Gm17036
Vgll3	14.29942	1.504015184	0.62008	2.42551	0.01529	0.03973	TRUE	Vgll3
Gm7701	140.7781	2.813398294	0.59838	4.70166	2.58E-06	1.92E-05	TRUE	Gm7701
810468N07R	29.36493	1.825455336	0.53693	3.39981	0.00067	0.00273	TRUE	2810468N07Rik
Gm17066	526.7925	1.68636934	0.35046	4.81193	1.49E-06	1.18E-05	TRUE	Gm17066
Cldn20	7.016982	4.118594554	1.28628	3.20195	0.00136	0.00506	TRUE	Cldn20
Gm6548	83.07054	2.289369136	0.37788	6.05845	1.37E-09	1.95E-08	TRUE	Gm6548
Gm17092	30.74927	1.679911551	0.60794	2.7633	0.00572	0.01742	TRUE	Gm17092
H2-Q2	6.077808	5.417904014	1.89523	2.8587	0.00425	0.01352	TRUE	H2-Q2
Siah3	312.4388	2.854858337	0.63098	4.52448	6.05E-06	4.13E-05	TRUE	Siah3
Gm17541	4.001254	4.811725585	1.70972	2.81434	0.00489	0.01522	TRUE	Gm17541
Gm4707	169.6966	2.472769721	0.31215	7.92181	2.34E-15	7.38E-14	TRUE	Gm4707
Gm8251	57.81568	3.921530964	0.64639	6.06681	1.30E-09	1.86E-08	TRUE	Gm8251
Rpl36-ps12	17.98524	1.875901711	0.73799	2.54192	0.01102	0.03029	TRUE	Rpl36-ps12
Gm17354	165.8341	1.056351837	0.2822	3.74324	0.00018	0.00086	TRUE	Gm17354
930036N10R	4.272194	4.29632563	1.45355	2.95574	0.00312	0.01037	TRUE	B930036N10Rik
Gm4468	26.31393	2.980250658	0.57035	5.22534	1.74E-07	1.68E-06	TRUE	Gm4468
Gbp11	7.000743	7.603457688	1.54851	4.91017	9.10E-07	7.55E-06	TRUE	Gbp11
Gm20457	48.67066	1.219779441	0.40784	2.99082	0.00278	0.00943	TRUE	Gm20457
110038B12R	566.0585	1.097686615	0.24302	4.51694	6.27E-06	4.26E-05	TRUE	1110038B12Rik
Gm20515	39.88749	3.206912539	0.76315	4.20221	2.64E-05	0.00016	TRUE	Gm20515
Gm20467	8.893663	5.992200058	1.37513	4.35755	1.32E-05	8.32E-05	TRUE	Gm20467
Neat1	59.44673	1.507929324	0.47188	3.19561	0.0014	0.00516	TRUE	Neat1
Cdk3	134.504	2.821202129	0.42831	6.58678	4.49E-11	8.02E-10	TRUE	Cdk3
Malat1	18189.2	1.208741401	0.40856	2.95853	0.00309	0.0103	TRUE	Malat1
Gpank1	322.4485	1.441311483	0.28259	5.10043	3.39E-07	3.08E-06	TRUE	Gpank1
Gm17907	82.51606	1.677088208	0.33401	5.02105	5.14E-07	4.52E-06	TRUE	Gm17907
Gm20468	5.140035	4.517365446	1.88564	2.39567	0.01659	0.04255	TRUE	Gm20468
Gm8738	99.30093	2.009093237	0.41875	4.79781	1.60E-06	1.25E-05	TRUE	Gm8738
BC051226	89.90966	1.038042526	0.34008	3.05231	0.00227	0.00792	TRUE	BC051226
Gm23638	85.04479	3.288306871	0.58583	5.61312	1.99E-08	2.34E-07	TRUE	Gm23638
Gm25641	5.899844	3.66275637	1.2601	2.90672	0.00365	0.01188	TRUE	Gm25641
Mir3093	44.32781	3.675362577	0.50709	7.24791	4.23E-13	9.97E-12	TRUE	Mir3093
Mir9-3	8.301683	5.541797586	1.35581	4.08744	4.36E-05	0.00024	TRUE	Mir9-3
Gm22933	47.24732	2.450733301	0.52277	4.68793	2.76E-06	2.04E-05	TRUE	Gm22933
330562C20R	46.01653	2.151302791	0.76061	2.82841	0.00468	0.01466	TRUE	6330562C20Rik
Zfhx2os	141.3032	5.480812769	0.6058	9.04725	1.47E-19	7.31E-18	TRUE	Zfhx2os
AA465934	62.07169	2.098344529	0.43587	4.8141	1.48E-06	1.17E-05	TRUE	AA465934
Gm20705	14.51202	4.183464022	1.14343	3.6587	0.00025	0.00116	TRUE	Gm20705
Rab26os	21.89823	1.711001468	0.67883	2.52051	0.01172	0.03186	TRUE	Rab26os
Prox2os	36.76234	2.10159825	0.47884	4.38896	1.14E-05	7.29E-05	TRUE	Prox2os
Eif4e3	873.6635	1.357002875	0.36371	3.73099	0.00019	0.0009	TRUE	Eif4e3
Pou5f2	76.89696	1.043280983	0.39319	2.65339	0.00797	0.02299	TRUE	Pou5f2
Rpl41	8125.238	1.248023797	0.30548	4.08543	4.40E-05	0.00025	TRUE	Rpl41
Gm27857	10.10068	2.965024561	1.15217	2.57344	0.01007	0.028	TRUE	Gm27857

Ppp2r3d	1910.822	1.102442746	0.33289	3.3117	0.00093	0.00361	TRUE	Ppp2r3d
Gm5117	69.20302	4.603161966	0.76847	5.99005	2.10E-09	2.88E-08	TRUE	Gm5117
Gm24539	31.27567	4.465496681	0.64507	6.92251	4.44E-12	9.21E-11	TRUE	Gm24539
Gm26268	25.1716	4.199605683	0.88746	4.73217	2.22E-06	1.67E-05	TRUE	Gm26268
Gm10406	58.51311	1.06841992	0.37917	2.81782	0.00484	0.01508	TRUE	Gm10406
Gm21885	24.94426	2.248597191	0.64898	3.46481	0.00053	0.0022	TRUE	Gm21885
Gm23966	11.87956	2.204431928	0.76795	2.87054	0.0041	0.01309	TRUE	Gm23966
Hist1h2ao	20.03444	2.198887867	0.8362	2.62963	0.00855	0.02442	TRUE	Hist1h2ao
Gm24924	65.26755	2.52961849	0.55645	4.546	5.47E-06	3.76E-05	TRUE	Gm24924
Hist1h2bl	12.50721	2.838222987	0.83007	3.41925	0.00063	0.00256	TRUE	Hist1h2bl
AC087559.3	2.410063	5.201548266	2.09256	2.48574	0.01293	0.03452	TRUE	AC087559.3
Gm3468	107.5853	1.981811578	0.30547	6.48767	8.72E-11	1.47E-09	TRUE	Gm3468
Rnaset2b	6.474335	2.298946017	0.96217	2.38934	0.01688	0.04315	TRUE	Rnaset2b
Gm22615	15.9553	3.595544798	0.71797	5.00792	5.50E-07	4.81E-06	TRUE	Gm22615
Gm21846	11.59175	2.186033382	0.91834	2.38041	0.01729	0.04406	TRUE	Gm21846
Gm23713	12.52802	2.241848568	0.92395	2.42637	0.01525	0.03967	TRUE	Gm23713
Gm22716	11.01838	2.122202692	0.73183	2.89985	0.00373	0.01211	TRUE	Gm22716
Gm21859	1.945953	5.564114331	2.27322	2.44768	0.01438	0.03774	TRUE	Gm21859
Rps2-ps6	53.41298	1.311649552	0.39254	3.34147	0.00083	0.00329	TRUE	Rps2-ps6
Gm26244	32.96936	3.360250494	0.59726	5.62608	1.84E-08	2.19E-07	TRUE	Gm26244
AA0114733	249.3881	1.289903459	0.46633	2.76607	0.00567	0.0173	TRUE	CAAA01147332.1
Nim1k	67.00053	1.597939152	0.4119	3.8794	0.0001	0.00053	TRUE	Nim1k
Gm26269	32.80842	2.357049264	0.60628	3.88774	0.0001	0.00051	TRUE	Gm26269
Kcnj11	99.52201	3.302209443	0.46559	7.09247	1.32E-12	2.94E-11	TRUE	Kcnj11
Gm22513	4.774407	5.880935789	2.48096	2.37043	0.01777	0.04506	TRUE	Gm22513
Gm24095	33.49464	3.45000335	0.64895	5.3163	1.06E-07	1.08E-06	TRUE	Gm24095
Gm10591	35.87013	3.480867726	0.57113	6.09475	1.10E-09	1.59E-08	TRUE	Gm10591
530416G11F	8.97136	2.82978783	0.90987	3.1101	0.00187	0.00666	TRUE	7530416G11Rik
Gm5559	1334.878	1.408584418	0.30636	4.59788	4.27E-06	3.01E-05	TRUE	Gm5559
CR974586.5	12.53666	4.315984204	0.98331	4.38926	1.14E-05	7.29E-05	TRUE	CR974586.5
Mfsd4b4	148.838	1.227454393	0.34826	3.52451	0.00042	0.00181	TRUE	Mfsd4b4
Gm28373	13.23953	3.218321702	0.80576	3.99412	6.49E-05	0.00035	TRUE	Gm28373
Gm4755	6.697245	5.282638106	1.593	3.31616	0.00091	0.00355	TRUE	Gm4755
Rps19-ps6	42.36641	1.874454099	0.54946	3.41142	0.00065	0.00262	TRUE	Rps19-ps6
Snhg18	89.59938	1.724265503	0.3835	4.49611	6.92E-06	4.66E-05	TRUE	Snhg18
930556M19F	49.11626	3.911929714	0.57124	6.84814	7.48E-12	1.50E-10	TRUE	4930556M19Rik
Gm10827	36.61867	3.161779416	0.50437	6.26876	3.64E-10	5.71E-09	TRUE	Gm10827
930509E16R	11.3873	3.937462335	1.18257	3.32958	0.00087	0.00341	TRUE	4930509E16Rik
Gm26789	96.77981	1.340145612	0.47348	2.83042	0.00465	0.01458	TRUE	Gm26789
Mir9-3hg	4191.196	3.760145105	0.34187	10.9989	3.87E-28	4.56E-26	TRUE	Mir9-3hg
Pvt1	7.838993	2.88788023	1.08539	2.66068	0.0078	0.02257	TRUE	Pvt1
Gm17491	305.8234	1.959923728	0.36253	5.40622	6.44E-08	6.84E-07	TRUE	Gm17491
600020E01R	638.245	1.721116617	0.37564	4.58178	4.61E-06	3.23E-05	TRUE	1600020E01Rik
Pantr2	132.1806	3.142995929	0.33598	9.3546	8.39E-21	4.78E-19	TRUE	Pantr2
Gm26604	110.2128	4.486551736	0.43602	10.2898	7.83E-25	6.64E-23	TRUE	Gm26604
700086O06F	81.16813	1.224830526	0.45192	2.71028	0.00672	0.01989	TRUE	1700086O06Rik
Gm19705	21.34331	2.873212728	0.94718	3.03342	0.00242	0.00835	TRUE	Gm19705
Gm10524	245.7934	2.773279709	0.53774	5.15728	2.51E-07	2.34E-06	TRUE	Gm10524
230114K14F	33.10353	1.648075023	0.4796	3.43636	0.00059	0.00242	TRUE	9230114K14Rik
Gm3839	3.007147	6.353983077	1.92906	3.29382	0.00099	0.00381	TRUE	Gm3839
Gm3764	17069.5	5.002462713	0.33782	14.8082	1.30E-49	7.72E-47	TRUE	Gm3764
630072L19F	20.08545	3.617707427	0.75487	4.79247	1.65E-06	1.28E-05	TRUE	A630072L19Rik
Gm4890	29.32959	1.331554103	0.53635	2.48262	0.01304	0.03479	TRUE	Gm4890
AU022754	43.6449	2.426944523	0.54928	4.41841	9.94E-06	6.46E-05	TRUE	AU022754
Gm26803	243.7624	3.354931949	0.4322	7.76238	8.33E-15	2.46E-13	TRUE	Gm26803
AI849053	63.07531	7.321480473	0.7241	10.1111	4.93E-24	3.88E-22	TRUE	AI849053
Gm26532	13.49034	2.705231462	0.8139	3.32378	0.00089	0.00347	TRUE	Gm26532
Gm17276	12.38613	2.857157115	1.00026	2.85641	0.00428	0.0136	TRUE	Gm17276
330048O09F	10.64523	2.936760984	1.06844	2.74864	0.00598	0.0181	TRUE	A330048O09Rik
Gm26814	180.8439	1.359924059	0.3383	4.01988	5.82E-05	0.00031	TRUE	Gm26814
930016D06F	1603.399	2.286714476	0.33965	6.73248	1.67E-11	3.19E-10	TRUE	D930016D06Rik
Gm26831	93.68525	3.85730612	0.49825	7.74172	9.81E-15	2.87E-13	TRUE	Gm26831
130046B21F	29.68238	1.587401671	0.53528	2.96558	0.00302	0.01011	TRUE	B130046B21Rik
AU020206	271.858	2.652676348	0.33543	7.90818	2.61E-15	8.20E-14	TRUE	AU020206
AW047730	196.7466	4.810042903	0.52053	9.24074	2.45E-20	1.34E-18	TRUE	AW047730
Gm26631	55.05183	1.558225403	0.50764	3.06957	0.00214	0.00751	TRUE	Gm26631
Gm26736	166.1993	2.439186518	0.31739	7.68517	1.53E-14	4.34E-13	TRUE	Gm26736
Gm26586	37.28048	1.306470139	0.51844	2.52	0.01174	0.03189	TRUE	Gm26586
Gm26807	69.39005	1.925536555	0.46844	4.11049	3.95E-05	0.00022	TRUE	Gm26807
130060C02F	14.70776	2.753652401	0.92414	2.9797	0.00289	0.00973	TRUE	C130060C02Rik
430590A07F	105.7666	1.713040792	0.41101	4.16788	3.07E-05	0.00018	TRUE	6430590A07Rik
230029C05F	65.11098	1.205317705	0.44289	2.72146	0.0065	0.01936	TRUE	E230029C05Rik
Dleu2	1690.974	3.390990519	0.34676	9.77903	1.39E-22	9.16E-21	TRUE	Dleu2
Gm26660	149.123	2.806651763	0.28967	9.68928	3.35E-22	2.13E-20	TRUE	Gm26660
Gm2061	69.56703	4.657275637	0.44929	10.3658	3.55E-25	3.04E-23	TRUE	Gm2061

Gm20033	133.6529	3.457285067	0.38369	9.01055	2.05E-19	1.00E-17	TRUE		Gm20033
Gm26761	4.146802	4.906015339	2.0155	2.43414	0.01493	0.03896	TRUE		Gm26761
Gm26644	36.44426	5.959457837	0.84669	7.03854	1.94E-12	4.24E-11	TRUE		Gm26644
Gm26739	8.455253	2.492341387	0.96792	2.57494	0.01003	0.02792	TRUE		Gm26739
810429I04R	40.17412	1.203322576	0.485	2.48107	0.0131	0.03492	TRUE		2810429I04Rik
630040K05R	5.606601	3.054334187	1.26373	2.41692	0.01565	0.04055	TRUE		F630040K05Rik
Gm4673	230.8661	2.204218736	0.51533	4.27728	1.89E-05	0.00012	TRUE		Gm4673
010110K18R	34.35457	1.290885873	0.43168	2.99038	0.00279	0.00944	TRUE		2010110K18Rik
030442K20R	37.57277	1.566118356	0.49643	3.15474	0.00161	0.00584	TRUE		6030442K20Rik
Gm26777	72.49928	3.402703531	0.41466	8.20592	2.29E-16	8.03E-15	TRUE		Gm26777
Gm6556	9.264909	2.204917586	0.81731	2.69777	0.00698	0.02055	TRUE		Gm6556
630001P10R	7.80478	3.807517503	1.27893	2.97711	0.00291	0.0098	TRUE		9630001P10Rik
610038B21R	10.2053	4.411814463	1.71751	2.56873	0.01021	0.02832	TRUE		0610038B21Rik
031434O11R	168.9981	2.392825486	0.57063	4.19332	2.75E-05	0.00016	TRUE		5031434O11Rik
Gm26801	103.7602	3.449408241	0.47802	7.21596	5.36E-13	1.25E-11	TRUE		Gm26801
Gm26531	15.91751	2.593777049	0.679	3.82	0.00013	0.00066	TRUE		Gm26531
Gm26917	40178.42	2.802380619	0.56246	4.9824	6.28E-07	5.41E-06	TRUE		Gm26917
Gm9522	9.056185	4.072348732	0.96312	4.22828	2.35E-05	0.00014	TRUE		Gm9522
Gm7909	17.46547	3.655374952	0.69192	5.28293	1.27E-07	1.27E-06	TRUE		Gm7909
700099C18R	438.4334	2.840440705	0.32583	8.71757	2.84E-18	1.21E-16	TRUE		2700099C18Rik
Gm6085	53.90762	3.5364501	0.66174	5.34416	9.08E-08	9.37E-07	TRUE		Gm6085
Gm5914	16.962	2.301049142	0.73589	3.12689	0.00177	0.00634	TRUE		Gm5914
Rassf10	28.77356	2.384812317	0.80583	2.95945	0.00308	0.01028	TRUE		Rassf10
Gm27010	176.0733	2.344100787	0.28067	8.35169	6.73E-17	2.50E-15	TRUE		Gm27010
Gm26964	71.80127	2.279748185	0.55037	4.14222	3.44E-05	0.0002	TRUE		Gm26964
Gm4258	74.58791	1.398639839	0.47454	2.94736	0.00321	0.0106	TRUE		Gm4258
330093E20R	7.910235	2.821602301	0.94564	2.98382	0.00285	0.00961	TRUE		A330093E20Rik
Lockd	407.4551	2.916853375	0.25632	11.3797	5.28E-30	7.85E-28	TRUE		Lockd
330468F06R	11.9877	4.426910869	1.02281	4.32817	1.50E-05	9.40E-05	TRUE		5830468F06Rik
Gm27202	39.68702	6.121221703	0.7944	7.70545	1.30E-14	3.74E-13	TRUE		Gm27202
Gm12117	20.97554	2.871753907	0.66313	4.33058	1.49E-05	9.31E-05	TRUE		Gm12117
Gm1113	9.968952	4.919588622	1.2761	3.85517	0.00012	0.00058	TRUE		Gm1113
Gm27209	193.888	1.530073042	0.38548	3.96923	7.21E-05	0.00038	TRUE		Gm27209
Mir7028	9.512386	2.861226441	0.93824	3.04956	0.00229	0.00798	TRUE		Mir7028
Gm27653	5.035504	5.097562807	1.65968	3.07142	0.00213	0.00747	TRUE		Gm27653
Mir6236	209.2108	3.329631842	0.61672	5.39897	6.70E-08	7.12E-07	TRUE		Mir6236
Rn7s1	7.79324	5.045184808	1.6633	3.03324	0.00242	0.00835	TRUE		Rn7s1
Mir8110	2.047643	5.971341068	2.43232	2.45499	0.01409	0.03715	TRUE		Mir8110
Gm19569	22.36807	1.439511711	0.52541	2.73981	0.00615	0.01849	TRUE		Gm19569
Mir6965	6.911415	4.783278469	1.29055	3.70639	0.00021	0.00098	TRUE		Mir6965
Gm27243	42.79348	2.169080485	0.60618	3.57825	0.00035	0.00151	TRUE		Gm27243
Rn7s2	3.176398	5.170821484	1.95159	2.64955	0.00806	0.02321	TRUE		Rn7s2
Gm29486	45.64728	3.380461053	0.54845	6.16365	7.11E-10	1.07E-08	TRUE		Gm29486
Xndc1	443.0302	2.198281413	0.23613	9.30952	1.28E-20	7.12E-19	TRUE		Xndc1
Hist1h3d	25.02324	3.040058913	0.54918	5.53561	3.10E-08	3.52E-07	TRUE		Hist1h3d
900052N01R	10.34069	4.807188509	1.48473	3.23776	0.0012	0.00454	TRUE		2900052N01Rik
Gm28800	30.55209	2.606152742	0.52795	4.9364	7.96E-07	6.69E-06	TRUE		Gm28800
Hmgb1-rs16	155.0184	3.514761999	0.57245	6.13982	8.26E-10	1.23E-08	TRUE		Hmgb1-rs16
810402E24R	261.1158	1.863203287	0.28448	6.54955	5.77E-11	1.01E-09	TRUE		2810402E24Rik
Gm28119	288.1471	2.114770965	0.50112	4.22009	2.44E-05	0.00014	TRUE		Gm28119
Hist1h3f	10.30655	2.168357993	0.88485	2.45053	0.01426	0.03751	TRUE		Hist1h3f
Gm8292	57.04005	1.421057818	0.41426	3.43033	0.0006	0.00247	TRUE		Gm8292
Gm29257	16.10481	3.740960413	0.76942	4.86207	1.16E-06	9.39E-06	TRUE		Gm29257
600014E21R	192.5112	5.214547911	0.47933	10.8787	1.46E-27	1.55E-25	TRUE		2600014E21Rik
Ovol3	10.3109	2.860855739	0.83879	3.41071	0.00065	0.00263	TRUE		Ovol3
Gm29483	3.933799	6.825556059	2.70626	2.52214	0.01166	0.03176	TRUE		Gm29483
Gm29019	6.282133	3.318084436	1.37766	2.40849	0.01602	0.04131	TRUE		Gm29019
Gm8173	36.66275	1.721865229	0.60319	2.85461	0.00431	0.01366	TRUE		Gm8173
700025F24R	4.089724	3.857547498	1.44727	2.6654	0.00769	0.0223	TRUE		1700025F24Rik
Gm20379	87.82084	2.34606644	0.48115	4.87592	1.08E-06	8.83E-06	TRUE		Gm20379
Gm29055	34.77663	2.953539814	0.80396	3.67372	0.00024	0.0011	TRUE		Gm29055
Gm29260	546.9593	12.0693753	1.06032	11.3828	5.10E-30	7.64E-28	TRUE		Gm29260
Gm10925	229.9422	1.256418275	0.34171	3.67687	0.00024	0.00109	TRUE		Gm10925
810049E08R	55.65841	2.916270514	0.39118	7.45514	8.98E-14	2.30E-12	TRUE		2810049E08Rik
Gm29666	80.3758	2.139593099	0.33557	6.37605	1.82E-10	2.96E-09	TRUE		Gm29666
Gm28991	3.878999	5.863286877	2.38733	2.456	0.01405	0.03706	TRUE		Gm28991
Gm28437	195.2371	1.366584777	0.41758	3.27265	0.00107	0.00407	TRUE		Gm28437
Gm29455	13.57604	4.754874317	1.10444	4.30523	1.67E-05	0.0001	TRUE		Gm29455
Gm28209	8.909107	4.815523807	1.30156	3.69982	0.00022	0.001	TRUE		Gm28209
Gm29216	64.07864	1.684531519	0.57577	2.92571	0.00344	0.01128	TRUE		Gm29216
BC055402	4.953101	7.014895137	1.67308	4.1928	2.76E-05	0.00016	TRUE		BC055402
Gm3052	105.1785	2.470058866	0.45643	5.41171	6.24E-08	6.67E-07	TRUE		Gm3052
Gm20342	432.2044	1.540154558	0.29989	5.13577	2.81E-07	2.60E-06	TRUE		Gm20342
Kcnq1ot1	2721.707	1.060296901	0.22379	4.73795	2.16E-06	1.63E-05	TRUE		Kcnq1ot1
Gm5835	4.879265	3.273073542	1.20417	2.71812	0.00657	0.01952	TRUE		Gm5835

30401M01	151.9577	1.055414671	0.30031	3.51445	0.00044	0.00187	TRUE	9130401M01Rik
Gm28438	42.82037	1.502051266	0.41932	3.58212	0.00034	0.00149	TRUE	Gm28438
Hist1h3i	27.44033	3.020320037	0.60951	4.95535	7.22E-07	6.14E-06	TRUE	Hist1h3i
Gm29480	27.95492	1.394068832	0.5686	2.45176	0.01422	0.03742	TRUE	Gm29480
Gm28661	166.5612	1.898974732	0.41256	4.60288	4.17E-06	2.95E-05	TRUE	Gm28661
Rpsa-ps1	10.59349	2.717969847	1.07276	2.53362	0.01129	0.0309	TRUE	Rpsa-ps1
Gm37106	75.03502	2.750810955	0.62492	4.40189	1.07E-05	6.93E-05	TRUE	Gm37106
Gm37472	110.2837	3.587904591	0.52548	6.82781	8.62E-12	1.71E-10	TRUE	Gm37472
Gm37474	27.67335	2.104225962	0.81613	2.57831	0.00993	0.02771	TRUE	Gm37474
Gm6119	7.832141	4.63484468	1.1511	4.02644	5.66E-05	0.00031	TRUE	Gm6119
Gm37716	88.9378	2.613557426	0.4361	5.99297	2.06E-09	2.83E-08	TRUE	Gm37716
430037O13	19.66717	3.639268357	0.90714	4.0118	6.03E-05	0.00032	TRUE	9430037O13Rik
Gm38260	12.46097	2.656663821	0.88767	2.99284	0.00276	0.00938	TRUE	Gm38260
Gm38257	18.15318	1.898120216	0.76228	2.49005	0.01277	0.03417	TRUE	Gm38257
Gm37144	151.1405	2.319043056	0.44803	5.17611	2.27E-07	2.14E-06	TRUE	Gm37144
130050O07	235.2144	1.478808876	0.4293	3.44468	0.00057	0.00235	TRUE	A130050O07Rik
Gm31258	66.16225	2.747537199	0.69124	3.97478	7.04E-05	0.00037	TRUE	Gm31258
Gm38192	42.83537	1.13653282	0.42172	2.69501	0.00704	0.02067	TRUE	Gm38192
Gm37626	31.68212	2.909943265	0.60461	4.81293	1.49E-06	1.17E-05	TRUE	Gm37626
430053O09	800.5644	2.733581483	0.47942	5.7018	1.19E-08	1.46E-07	TRUE	9430053O09Rik
Gm38142	27.34441	3.609378317	0.93014	3.88048	0.0001	0.00053	TRUE	Gm38142
00022M07	374.2341	2.88373157	0.47691	6.04664	1.48E-09	2.09E-08	TRUE	2900022M07Rik
Gm37463	15.49775	1.668118139	0.65657	2.54066	0.01106	0.03037	TRUE	Gm37463
Gm29856	14.68983	4.539547106	0.84857	5.34965	8.81E-08	9.11E-07	TRUE	Gm29856
Gm21955	4.200274	3.718874738	1.31206	2.83438	0.00459	0.01442	TRUE	Gm21955
Gm37783	9.817306	2.196089385	0.90953	2.41452	0.01576	0.04079	TRUE	Gm37783
Gm37694	572.029	2.521265802	0.43498	5.79629	6.78E-09	8.69E-08	TRUE	Gm37694
Gm37968	56.15813	1.890677696	0.4583	4.12537	3.70E-05	0.00021	TRUE	Gm37968
Gm37969	34.2811	4.202036553	0.96798	4.34105	1.42E-05	8.92E-05	TRUE	Gm37969
Gm38384	222.6864	1.973008843	0.3244	6.08201	1.19E-09	1.71E-08	TRUE	Gm38384
930004J17R	322.4884	2.556491007	0.41684	6.133	8.62E-10	1.27E-08	TRUE	A930004J17Rik
Gm37219	3.696184	4.660556201	1.86785	2.49514	0.01259	0.03378	TRUE	Gm37219
Gm38262	8.63999	4.388145931	1.54653	2.83742	0.00455	0.0143	TRUE	Gm38262
830444F18R	65.59999	1.814082322	0.52283	3.46972	0.00052	0.00217	TRUE	5830444F18Rik
Gm7694	446.8611	1.389326475	0.20267	6.85504	7.13E-12	1.43E-10	TRUE	Gm7694
Gm38164	25.62075	2.059961421	0.69304	2.97234	0.00296	0.00993	TRUE	Gm38164
Gm38252	39.117	1.447543473	0.53538	2.70378	0.00686	0.02023	TRUE	Gm38252
Gm37383	26.66257	3.077855532	0.77739	3.95922	7.52E-05	0.00039	TRUE	Gm37383
130023A14	541.1004	1.921932156	0.41549	4.62571	3.73E-06	2.68E-05	TRUE	C130023A14Rik
Gm38115	35.58516	1.92008809	0.68495	2.80323	0.00506	0.01568	TRUE	Gm38115
Gm37019	23.33945	3.043424621	0.93158	3.26696	0.00109	0.00415	TRUE	Gm37019
Gm37017	22.6879	3.454449147	0.94616	3.65102	0.00026	0.00119	TRUE	Gm37017
Gm33366	30.98675	2.593181272	0.69725	3.71914	0.0002	0.00094	TRUE	Gm33366
430014B02R	34.73181	1.594510004	0.56247	2.83481	0.00459	0.01441	TRUE	E430014B02Rik
330074H02	49.01163	2.038906102	0.58826	3.46599	0.00053	0.00219	TRUE	A330074H02Rik
Gm37503	17.1334	2.98596502	1.04203	2.86553	0.00416	0.01328	TRUE	Gm37503
Gm37124	253.1172	1.483741903	0.3362	4.41322	1.02E-05	6.60E-05	TRUE	Gm37124
Gm38124	88.36387	1.474970841	0.35251	4.18419	2.86E-05	0.00017	TRUE	Gm38124
Gm38120	55.89995	1.665309387	0.60959	2.73186	0.0063	0.01887	TRUE	Gm38120
Gm37390	8.75498	2.865564134	0.96433	2.97157	0.00296	0.00995	TRUE	Gm37390
Gm2238	14.25351	1.725028171	0.69177	2.49366	0.01264	0.0339	TRUE	Gm2238
Gm37745	23.35467	1.984217931	0.75644	2.62309	0.00871	0.02483	TRUE	Gm37745
Gm37159	6.763637	3.477957916	1.27795	2.72152	0.0065	0.01936	TRUE	Gm37159
Gm37519	64.60099	3.034318462	0.578	5.24971	1.52E-07	1.50E-06	TRUE	Gm37519
10080O07	137.6585	1.711475575	0.50055	3.41921	0.00063	0.00256	TRUE	3110080O07Rik
Gm37274	457.6787	1.857746988	0.29564	6.28382	3.30E-10	5.20E-09	TRUE	Gm37274
Gm38359	21.13618	1.65163596	0.69439	2.37855	0.01738	0.04424	TRUE	Gm38359
Gm38157	276.9764	1.767632514	0.49998	3.53538	0.00041	0.00174	TRUE	Gm38157
Gm37239	4.39113	6.814684677	1.77896	3.83071	0.00013	0.00063	TRUE	Gm37239
Gm37931	52.05301	2.038096444	0.50053	4.07187	4.66E-05	0.00026	TRUE	Gm37931
Gm37534	99.93151	2.258083693	0.54929	4.11094	3.94E-05	0.00022	TRUE	Gm37534
720464F23R	27.0265	1.586277838	0.58565	2.70859	0.00676	0.01997	TRUE	6720464F23Rik
Gm37999	67.52576	1.457366296	0.45015	3.2375	0.00121	0.00454	TRUE	Gm37999
Gm10657	6.466882	2.400327561	1.03178	2.3264	0.02	0.04964	TRUE	Gm10657
Gm37666	59.0751	1.762574252	0.64233	2.74403	0.00607	0.01831	TRUE	Gm37666
Gm38220	61.77218	3.019860712	0.45162	6.68666	2.28E-11	4.28E-10	TRUE	Gm38220
810405F15R	7.919822	2.888271197	1.10364	2.61703	0.00887	0.0252	TRUE	2810405F15Rik
030445P17R	133.2409	2.151808	0.53978	3.98645	6.71E-05	0.00036	TRUE	8030445P17Rik
Gm38067	44.40681	1.487919411	0.54434	2.73346	0.00627	0.01879	TRUE	Gm38067
Gm37116	30.29429	2.289443943	0.54543	4.19753	2.70E-05	0.00016	TRUE	Gm37116
Gm38365	31.71986	2.610076527	0.54455	4.79305	1.64E-06	1.28E-05	TRUE	Gm38365
Gm37195	11.84858	5.471458197	1.54874	3.53285	0.00041	0.00176	TRUE	Gm37195
Gm37242	10.64777	2.699754488	1.12084	2.4087	0.01601	0.0413	TRUE	Gm37242
Gm37706	145.0621	2.358789285	0.56919	4.14415	3.41E-05	0.0002	TRUE	Gm37706
00037B21R	51.71329	3.5813469	0.56454	6.34388	2.24E-10	3.59E-09	TRUE	2900037B21Rik

Gm37529	57.35974	2.282730408	0.42185	5.41121	6.26E-08	6.69E-07	TRUE	Gm37529
Gm37531	102.7733	2.388885233	0.50507	4.72979	2.25E-06	1.69E-05	TRUE	Gm37531
Gm37524	33.70719	1.921468537	0.70982	2.70698	0.00679	0.02006	TRUE	Gm37524
Pcdhga6	273.9593	1.927613515	0.42214	4.56629	4.96E-06	3.46E-05	TRUE	Pcdhga6
Gm37064	3.748067	4.04496584	1.51098	2.67705	0.00743	0.02163	TRUE	Gm37064
Gm37060	26.36893	1.77465243	0.59721	2.97157	0.00296	0.00995	TRUE	Gm37060
)30460B20R	44.47969	2.333832544	0.53098	4.3953	1.11E-05	7.11E-05	TRUE	6030460B20Rik
Gm37608	45.12988	2.309223056	0.6875	3.35885	0.00078	0.00311	TRUE	Gm37608
Gm37612	56.72249	2.209529057	0.60149	3.67344	0.00024	0.0011	TRUE	Gm37612
Gm37607	258.0357	2.407494748	0.48917	4.9216	8.58E-07	7.15E-06	TRUE	Gm37607
Gm37416	278.6385	4.641782972	0.33286	13.9451	3.37E-44	1.38E-41	TRUE	Gm37416
Gm37420	20.23711	3.083085103	0.7142	4.31683	1.58E-05	9.83E-05	TRUE	Gm37420
Gm37452	123.5015	2.844983027	0.49923	5.69871	1.21E-08	1.48E-07	TRUE	Gm37452
Gm38071	582.7911	2.164531083	0.35844	6.03884	1.55E-09	2.18E-08	TRUE	Gm38071
Gm37897	8.16211	4.867896266	1.33516	3.64593	0.00027	0.00121	TRUE	Gm37897
Gm36963	1074.016	2.109178053	0.39223	5.37745	7.55E-08	7.93E-07	TRUE	Gm36963
Gm37720	34.86175	2.242992644	0.63512	3.53161	0.00041	0.00177	TRUE	Gm37720
Gm37678	17.28471	2.04306308	0.76067	2.68589	0.00723	0.02115	TRUE	Gm37678
Gm37677	47.61469	1.649102144	0.58358	2.82583	0.00472	0.01475	TRUE	Gm37677
Gm20045	214.8606	1.230397285	0.27017	4.55422	5.26E-06	3.63E-05	TRUE	Gm20045
Gm37447	63.47238	1.105851504	0.40649	2.72049	0.00652	0.01941	TRUE	Gm37447
Gm38026	8.307721	6.75579332	1.5949	4.23587	2.28E-05	0.00014	TRUE	Gm38026
333412K13R	15.31281	2.009053407	0.77177	2.60317	0.00924	0.02606	TRUE	4833412K13Rik
Gm37562	14.94022	2.589774217	0.76187	3.39923	0.00068	0.00273	TRUE	Gm37562
Gm37954	41.44913	3.975638091	0.70256	5.65876	1.52E-08	1.84E-07	TRUE	Gm37954
Gm36955	32.84889	2.095560761	0.51243	4.08948	4.32E-05	0.00024	TRUE	Gm36955
130019J16R	546.3506	1.964535895	0.36532	5.37752	7.55E-08	7.93E-07	TRUE	D130019J16Rik
Gm37399	12.79472	2.268406827	0.91557	2.47759	0.01323	0.03522	TRUE	Gm37399
Gm37401	18.85643	3.994323583	0.81009	4.93072	8.19E-07	6.86E-06	TRUE	Gm37401
Gm37986	43.87272	2.250866862	0.52888	4.25594	2.08E-05	0.00013	TRUE	Gm37986
Gm37051	32.17159	2.054789682	0.55123	3.72763	0.00019	0.00091	TRUE	Gm37051
Gm9442	47.7812	2.396500804	0.54022	4.43616	9.16E-06	6.00E-05	TRUE	Gm9442
Gm20172	73.70244	2.206676134	0.54426	4.05447	5.02E-05	0.00027	TRUE	Gm20172
Gm38297	57.89535	2.626609523	0.61079	4.30033	1.71E-05	0.00011	TRUE	Gm38297
530064N14R	52.3288	1.378164812	0.53339	2.58378	0.00977	0.02736	TRUE	A530064N14Rik
130089K02R	220.6118	1.672433896	0.40712	4.10795	3.99E-05	0.00022	TRUE	C130089K02Rik
Gm36401	50.05764	1.317357337	0.52204	2.52348	0.01162	0.03165	TRUE	Gm36401
Gm37033	52.83694	1.843576364	0.45217	4.0772	4.56E-05	0.00025	TRUE	Gm37033
Gm38012	16.42893	2.865292876	0.68811	4.16401	3.13E-05	0.00018	TRUE	Gm38012
Gm37423	42.64698	1.287745929	0.55415	2.32381	0.02014	0.04991	TRUE	Gm37423
Gm37736	13.80922	4.816393729	1.13399	4.24731	2.16E-05	0.00013	TRUE	Gm37736
Gm37163	24.15096	2.290617511	0.66033	3.46888	0.00052	0.00217	TRUE	Gm37163
Gm37333	201.9465	1.626855908	0.445	3.65584	0.00026	0.00117	TRUE	Gm37333
Gm3527	3.206187	4.827068938	1.91962	2.5146	0.01192	0.03232	TRUE	Gm3527
330115B05R	14.14441	4.454773562	0.97553	4.5665	4.96E-06	3.45E-05	TRUE	F830115B05Rik
Gm42675	32.93316	3.197371812	0.83323	3.83734	0.00012	0.00062	TRUE	Gm42675
Gm43774	64.01666	1.982726134	0.37355	5.30778	1.11E-07	1.12E-06	TRUE	Gm43774
Gm42461	8.232035	2.047520684	0.84723	2.41673	0.01566	0.04056	TRUE	Gm42461
Gm42939	22.00698	2.590818782	0.60316	4.29542	1.74E-05	0.00011	TRUE	Gm42939
)33437G19R	17.12535	2.483704253	0.88766	2.79803	0.00514	0.01591	TRUE	4933437G19Rik
Gm43162	33.92434	2.182807644	0.63722	3.4255	0.00061	0.00251	TRUE	Gm43162
Gm42783	100.0817	2.10621914	0.3682	5.7203	1.06E-08	1.32E-07	TRUE	Gm42783
330487J09R	249.9605	2.597120912	0.39501	6.5748	4.87E-11	8.66E-10	TRUE	5830487J09Rik
Gm43201	192.9702	1.313910169	0.40259	3.26363	0.0011	0.00419	TRUE	Gm43201
.10039M20R	120.2362	7.215055327	0.58946	12.24	1.90E-34	4.08E-32	TRUE	3110039M20Rik
Gm42639	82.53816	2.100637172	0.43351	4.84561	1.26E-06	1.01E-05	TRUE	Gm42639
Gm42830	22.92778	2.009771542	0.66565	3.01925	0.00253	0.0087	TRUE	Gm42830
Gm42908	9.526042	2.644545439	1.08968	2.4269	0.01523	0.03963	TRUE	Gm42908
310432F15R	22.56794	2.197829139	0.72534	3.03005	0.00245	0.00843	TRUE	2810432F15Rik
Gm43075	2.259881	6.11114094	2.37237	2.57596	0.01	0.02785	TRUE	Gm43075
Gm42676	79.89855	4.749406554	0.48796	9.73321	2.18E-22	1.42E-20	TRUE	Gm42676
Gm43336	47.43572	1.277562763	0.51772	2.46769	0.0136	0.03607	TRUE	Gm43336
·30422M14R	93.78911	1.328267137	0.47226	2.81258	0.00491	0.01529	TRUE	8430422M14Rik
Gm43412	20.58724	1.947152929	0.59666	3.26344	0.0011	0.00419	TRUE	Gm43412
Gm43362	141.8989	1.60414118	0.30128	5.3245	1.01E-07	1.04E-06	TRUE	Gm43362
Gm33370	4.686974	6.885996706	2.75838	2.49639	0.01255	0.03369	TRUE	Gm33370
Gm42693	59.92121	1.138540596	0.44632	2.55094	0.01074	0.02961	TRUE	Gm42693
Gm43696	126.8526	1.888724765	0.4124	4.57985	4.65E-06	3.26E-05	TRUE	Gm43696
)30018K13R	10.63695	6.001394092	1.54883	3.8748	0.00011	0.00054	TRUE	C030018K13Rik
Gm43719	30.58989	1.717473125	0.47975	3.57994	0.00034	0.0015	TRUE	Gm43719
Rpl36a-ps2	115.6479	1.053021749	0.40941	2.57202	0.01011	0.02809	TRUE	Rpl36a-ps2
Gm43768	10.95302	2.241194854	0.83483	2.68462	0.00726	0.02121	TRUE	Gm43768
Gm43692	52.67135	1.150478257	0.43526	2.6432	0.00821	0.0236	TRUE	Gm43692
Gm43359	202.5956	2.089776235	0.43213	4.83594	1.33E-06	1.06E-05	TRUE	Gm43359
Gm42614	13.87944	2.732286007	0.85073	3.21171	0.00132	0.00491	TRUE	Gm42614

Gm43702	4.650073	5.946322992	2.01267	2.95444	0.00313	0.01041	TRUE	Gm43702
430073D23I	22.15162	2.313917407	0.6982	3.3141	0.00092	0.00358	TRUE	A430073D23Rik
Gm42995	16.26108	2.425670398	0.8999	2.69549	0.00703	0.02065	TRUE	Gm42995
430021H15I	66.45699	1.748250989	0.4415	3.95978	7.50E-05	0.00039	TRUE	E430021H15Rik
Gm43136	6.741181	3.09559103	1.19806	2.58383	0.00977	0.02736	TRUE	Gm43136
Gm34248	38.09733	3.181790218	0.57773	5.50737	3.64E-08	4.07E-07	TRUE	Gm34248
Gm43513	60.34081	2.863546977	0.56298	5.08642	3.65E-07	3.29E-06	TRUE	Gm43513
Gm43187	70.61479	1.658705574	0.47888	3.46373	0.00053	0.00221	TRUE	Gm43187
Gm42480	8.752412	2.338415671	0.94993	2.46166	0.01383	0.0366	TRUE	Gm42480
Gm42659	144.3203	1.485754187	0.3284	4.52415	6.06E-06	4.13E-05	TRUE	Gm42659
Gm35394	25.15055	2.764440417	0.62331	4.43507	9.20E-06	6.03E-05	TRUE	Gm35394
Gm42876	28.41938	1.560844858	0.59636	2.61727	0.00886	0.02519	TRUE	Gm42876
Gm43305	64.95422	2.443876768	0.92262	2.64884	0.00808	0.02326	TRUE	Gm43305
Gm43465	32.65609	4.147124261	0.54338	7.63203	2.31E-14	6.40E-13	TRUE	Gm43465
Gm42440	33.38849	2.458059567	0.777	3.16354	0.00156	0.00569	TRUE	Gm42440
Gm42443	93.59236	2.844633612	0.58445	4.86716	1.13E-06	9.18E-06	TRUE	Gm42443
Gm42819	9.583956	3.392400109	1.00849	3.36383	0.00077	0.00306	TRUE	Gm42819
330425B07I	2.767964	6.03846994	2.4667	2.44799	0.01437	0.03771	TRUE	5330425B07Rik
Gm43088	40.16694	1.575055682	0.40611	3.87838	0.00011	0.00053	TRUE	Gm43088
Gm43006	13.98026	1.554615764	0.66085	2.35244	0.01865	0.04687	TRUE	Gm43006
Gm43292	18.69896	2.539305077	0.59036	4.3013	1.70E-05	0.0001	TRUE	Gm43292
Gm3970	88.4706	2.658370636	0.54221	4.90281	9.45E-07	7.80E-06	TRUE	Gm3970
Gm43541	24.14257	2.353242954	0.79823	2.94808	0.0032	0.01058	TRUE	Gm43541
Gm6745	37.19765	2.586354896	0.60453	4.27828	1.88E-05	0.00012	TRUE	Gm6745
Gm43189	3.04895	6.137888038	2.0791	2.95219	0.00316	0.01047	TRUE	Gm43189
Gm43628	6.117019	2.667738639	1.12661	2.36794	0.01789	0.04528	TRUE	Gm43628
Gm43570	29.1671	3.581827873	0.55042	6.50739	7.65E-11	1.31E-09	TRUE	Gm43570
Gm40123	8.690259	3.437975178	1.07761	3.19038	0.00142	0.00524	TRUE	Gm40123
Gm42432	71.9983	1.423082094	0.4782	2.97589	0.00292	0.00983	TRUE	Gm42432
Gm43411	5.07096	3.610749744	1.33921	2.69618	0.00701	0.02063	TRUE	Gm43411
Gm42993	53.90093	1.493166939	0.54578	2.73586	0.00622	0.01867	TRUE	Gm42993
AI506816	1309.391	2.274639	0.34658	6.56317	5.27E-11	9.32E-10	TRUE	AI506816
530083M17I	80.46298	3.019995084	0.48772	6.19211	5.94E-10	9.05E-09	TRUE	A530083M17Rik
Gm42463	149.108	1.034046608	0.41541	2.48923	0.0128	0.03423	TRUE	Gm42463
Gm4332	135.0045	1.372879502	0.40453	3.39375	0.00069	0.00278	TRUE	Gm4332
Gm43560	108.998	2.086759997	0.53846	3.87545	0.00011	0.00054	TRUE	Gm43560
Gm43773	77.0454	2.265103823	0.44272	5.11637	3.11E-07	2.85E-06	TRUE	Gm43773
Gm42892	22.56189	2.866724528	0.69693	4.11334	3.90E-05	0.00022	TRUE	Gm42892
CT010467.1	675713.5	3.337700847	0.54142	6.16476	7.06E-10	1.06E-08	TRUE	CT010467.1
Gm43420	23.2886	2.210676814	0.73815	2.99487	0.00275	0.00933	TRUE	Gm43420
Gm43463	11.72125	1.830473755	0.78505	2.33168	0.01972	0.04907	TRUE	Gm43463
Gm42717	23.25223	1.959770071	0.58142	3.37065	0.00075	0.00299	TRUE	Gm42717
230096K16I	23.45725	2.914116207	0.61996	4.70046	2.60E-06	1.93E-05	TRUE	C230096K16Rik
Gm18867	2.672362	6.322802392	2.31479	2.73148	0.0063	0.01889	TRUE	Gm18867
Gm42658	59.63064	2.052637784	0.38828	5.28645	1.25E-07	1.25E-06	TRUE	Gm42658
Gm42992	792.1018	2.571380705	0.43274	5.94215	2.81E-09	3.81E-08	TRUE	Gm42992
Gm43506	13.96653	2.250256961	0.81831	2.74987	0.00596	0.01805	TRUE	Gm43506
Gm43549	127.5224	3.200335251	0.4755	6.73046	1.69E-11	3.23E-10	TRUE	Gm43549
Gm4869	14.99898	3.231750927	0.87058	3.71216	0.00021	0.00096	TRUE	Gm4869
Gm43361	54.58807	1.63271297	0.56787	2.87514	0.00404	0.01293	TRUE	Gm43361
Gm42991	358.7876	1.013633211	0.34865	2.90729	0.00365	0.01187	TRUE	Gm42991
Gm5857	37.43957	3.018739448	0.61899	4.87685	1.08E-06	8.79E-06	TRUE	Gm5857
Gm42820	34.67441	3.293496705	0.55019	5.98605	2.15E-09	2.95E-08	TRUE	Gm42820
Gm42970	304.126	1.211830178	0.37051	3.27068	0.00107	0.0041	TRUE	Gm42970
Gm43378	14.93709	3.439382578	0.87349	3.93751	8.23E-05	0.00043	TRUE	Gm43378
230303O12I	94.90476	2.214851291	0.37824	5.85572	4.75E-09	6.22E-08	TRUE	B230303O12Rik
Gm42814	22.20474	4.436449715	0.98439	4.50681	6.58E-06	4.45E-05	TRUE	Gm42814
230071H17I	52.21926	2.551854114	0.55373	4.60849	4.06E-06	2.88E-05	TRUE	C230071H17Rik
Gm43813	238.98	2.035770204	0.43681	4.66053	3.15E-06	2.30E-05	TRUE	Gm43813
Gm42524	3.467429	6.445496755	2.31225	2.78755	0.00531	0.01635	TRUE	Gm42524
Gm42729	37.32324	1.716281606	0.46017	3.72968	0.00019	0.0009	TRUE	Gm42729
Gm42798	175.4201	2.135306609	0.37409	5.70796	1.14E-08	1.41E-07	TRUE	Gm42798
Gm43281	40.4506	3.369056641	0.69546	4.84438	1.27E-06	1.02E-05	TRUE	Gm43281
Gm42467	36.91047	1.930850234	0.57148	3.37869	0.00073	0.00291	TRUE	Gm42467
Gm42583	135.4666	1.421128941	0.46816	3.03557	0.0024	0.0083	TRUE	Gm42583
Gtf3c2	1500.387	1.617811327	0.25312	6.39152	1.64E-10	2.69E-09	TRUE	Gtf3c2
Gm20186	865.1658	3.637252582	0.47543	7.65049	2.00E-14	5.60E-13	TRUE	Gm20186
930005G22I	26.44117	1.484350893	0.53944	2.75166	0.00593	0.01796	TRUE	A930005G22Rik
Gm42854	17.63965	3.327529979	1.18517	2.80764	0.00499	0.0155	TRUE	Gm42854
Gm43860	87.75217	1.104272414	0.39873	2.76949	0.00561	0.01714	TRUE	Gm43860
Gm43843	96.08619	1.389734876	0.40749	3.4105	0.00065	0.00263	TRUE	Gm43843
N4bp2os	39.05543	1.516572256	0.61372	2.4711	0.01347	0.03579	TRUE	N4bp2os
720475M21I	7.856395	2.681171202	1.10965	2.41623	0.01568	0.04061	TRUE	6720475M21Rik
Gm42853	1036.256	2.18250862	0.40002	5.45601	4.87E-08	5.33E-07	TRUE	Gm42853
030017G13I	27.30193	1.679911117	0.49783	3.37449	0.00074	0.00295	TRUE	C030017G13Rik

Gm42742	4.905792	2.824246236	1.1543	2.44671	0.01442	0.03781	TRUE	Gm42742
Gm42726	154.8196	3.205609998	0.54109	5.92434	3.14E-09	4.21E-08	TRUE	Gm42726
Gm43274	71.26099	1.768909526	0.59906	2.95282	0.00315	0.01045	TRUE	Gm43274
Gm43059	119.9905	1.815838721	0.40876	4.44226	8.90E-06	5.86E-05	TRUE	Gm43059
Gm42728	29.51077	2.721374495	0.61232	4.44435	8.82E-06	5.81E-05	TRUE	Gm42728
Gm42507	98.67313	1.314603421	0.41229	3.1885	0.00143	0.00527	TRUE	Gm42507
Gm42531	42.42409	2.241992246	0.51011	4.39513	1.11E-05	7.12E-05	TRUE	Gm42531
Gm42897	22.96641	2.435599566	0.66279	3.67477	0.00024	0.00109	TRUE	Gm42897
Mpv17	390.1537	1.301328691	0.35009	3.71708	0.0002	0.00094	TRUE	Mpv17
Gm42549	22.91295	1.871731124	0.56346	3.32183	0.00089	0.00349	TRUE	Gm42549
Gm40309	125.8744	2.044468811	0.47648	4.29075	1.78E-05	0.00011	TRUE	Gm40309
Gm44416	48.93765	2.227828512	0.45958	4.84758	1.25E-06	1.00E-05	TRUE	Gm44416
Gm44291	124.3116	1.198780356	0.38253	3.13381	0.00173	0.00621	TRUE	Gm44291
330037G11F	66.36263	1.248473503	0.44949	2.77752	0.00548	0.01678	TRUE	E330037G11Rik
Gm44199	239.8798	2.207091815	0.45563	4.84401	1.27E-06	1.02E-05	TRUE	Gm44199
Gm44264	32.59863	2.334075526	0.73024	3.1963	0.00139	0.00515	TRUE	Gm44264
Gm44093	41.30118	1.684188702	0.7018	2.39983	0.0164	0.04214	TRUE	Gm44093
Gm44041	55.31259	2.439443361	0.62576	3.89836	9.68E-05	0.00049	TRUE	Gm44041
Gm44256	55.04027	1.558509608	0.6046	2.57777	0.00994	0.02774	TRUE	Gm44256
Gm6749	3.832675	4.194172038	1.51901	2.76112	0.00576	0.01752	TRUE	Gm6749
Gm44068	20.62664	2.611854493	0.79199	3.29784	0.00097	0.00377	TRUE	Gm44068
Gm44062	8.294305	4.535077772	1.47213	3.08061	0.00207	0.00728	TRUE	Gm44062
Gm44080	18.70695	5.25764249	1.36274	3.85813	0.00011	0.00057	TRUE	Gm44080
130021K23F	63.67023	1.253456501	0.40456	3.09833	0.00195	0.0069	TRUE	B130021K23Rik
Gm3279	3.116495	4.601347051	1.75814	2.61717	0.00887	0.0252	TRUE	Gm3279
Gm5314	4.686974	6.885996706	2.75838	2.49639	0.01255	0.03369	TRUE	Gm5314
Gm7932	4.890352	7.040542246	2.10656	3.34219	0.00083	0.00328	TRUE	Gm7932
Gm44152	9.869848	3.104779737	0.9367	3.31458	0.00092	0.00357	TRUE	Gm44152
Gm43915	64.90577	1.717440252	0.66105	2.59805	0.00938	0.0264	TRUE	Gm43915
Gm43965	219.3764	2.53852694	0.33609	7.55312	4.25E-14	1.14E-12	TRUE	Gm43965
Gm44292	107.687	1.945286067	0.43023	4.52148	6.14E-06	4.18E-05	TRUE	Gm44292
Gm44167	13.80469	1.840416503	0.76349	2.41053	0.01593	0.04115	TRUE	Gm44167
Gm44737	8.690046	2.721810031	0.87474	3.11156	0.00186	0.00663	TRUE	Gm44737
430431A17F	109.8463	2.881193215	0.49124	5.86516	4.49E-09	5.89E-08	TRUE	5430431A17Rik
Gm44673	49.74286	2.926698892	0.48032	6.09319	1.11E-09	1.60E-08	TRUE	Gm44673
Gm45477	81.22763	1.720127955	0.43413	3.96227	7.42E-05	0.00039	TRUE	Gm45477
Gm6905	1.972898	5.912030024	2.41471	2.44834	0.01435	0.0377	TRUE	Gm6905
430064I24R	35.25678	1.740299954	0.54514	3.19238	0.00141	0.00521	TRUE	9430064I24Rik
Snhg1	329.2805	1.33029921	0.23694	5.61441	1.97E-08	2.33E-07	TRUE	Snhg1
Gm30146	3.309079	6.642757869	2.09238	3.17473	0.0015	0.0055	TRUE	Gm30146
Gm45025	9.875054	2.855825782	0.96397	2.96257	0.00305	0.01019	TRUE	Gm45025
Gm45203	336.936	2.077429106	0.4166	4.98658	6.15E-07	5.30E-06	TRUE	Gm45203
Gm44738	4.088102	5.71991296	2.05302	2.7861	0.00533	0.01641	TRUE	Gm44738
Gm45033	19.31555	2.009083196	0.63826	3.14775	0.00165	0.00595	TRUE	Gm45033
720469O03F	21.1753	4.896540902	1.35655	3.60954	0.00031	0.00137	TRUE	6720469O03Rik
310001K20F	4.795536	5.962394719	1.63115	3.65532	0.00026	0.00117	TRUE	2310001K20Rik
Gm44639	104.9935	2.540445942	0.54086	4.69703	2.64E-06	1.96E-05	TRUE	Gm44639
Gm20274	41.36019	1.324640579	0.51542	2.57003	0.01017	0.02822	TRUE	Gm20274
Gm44645	981.74	2.57283699	0.32907	7.81842	5.35E-15	1.63E-13	TRUE	Gm44645
Gm35040	670.0093	3.111300951	0.25309	12.2933	9.84E-35	2.20E-32	TRUE	Gm35040
Gm44633	166.8271	3.776099139	0.30536	12.3661	3.99E-35	9.13E-33	TRUE	Gm44633
Gm44867	25.07847	3.983301826	0.65525	6.07904	1.21E-09	1.73E-08	TRUE	Gm44867
Gm44758	83.3002	1.386062482	0.53245	2.60318	0.00924	0.02606	TRUE	Gm44758
Gm45153	11.06595	8.15716047	1.51127	5.39754	6.76E-08	7.17E-07	TRUE	Gm45153
Gm44636	26.19335	1.903081607	0.69016	2.75744	0.00583	0.01768	TRUE	Gm44636
430434F05R	40.15064	2.309374241	0.74089	3.11703	0.00183	0.00652	TRUE	5430434F05Rik
Gm30437	16.46217	3.751000566	0.87553	4.28425	1.83E-05	0.00011	TRUE	Gm30437
130083A15F	29.53522	1.980397035	0.60419	3.2778	0.00105	0.00401	TRUE	C130083A15Rik
Gm17909	2.857657	6.266175182	2.42694	2.58192	0.00983	0.02748	TRUE	Gm17909
Gm44917	2.767964	6.03846994	2.4667	2.44799	0.01437	0.03771	TRUE	Gm44917
230020D15F	46.45742	2.290348995	0.58284	3.92961	8.51E-05	0.00044	TRUE	E230020D15Rik
Gm44901	192.8131	2.251702794	0.41833	5.38256	7.34E-08	7.73E-07	TRUE	Gm44901
Gm45179	42.92878	1.848218309	0.59825	3.0894	0.00201	0.00709	TRUE	Gm45179
Gm44916	105.9048	2.40826757	0.37484	6.42478	1.32E-10	2.20E-09	TRUE	Gm44916
Gm45027	16.68434	4.836157198	0.97315	4.96959	6.71E-07	5.75E-06	TRUE	Gm45027
Gm45120	189.1512	3.383363647	0.56776	5.95915	2.54E-09	3.45E-08	TRUE	Gm45120
Gm44781	7.304619	2.89988991	1.16117	2.4974	0.01251	0.03365	TRUE	Gm44781
Gm36371	67.55904	1.955766499	0.48268	4.05189	5.08E-05	0.00028	TRUE	Gm36371
930431P19F	45.27111	1.297860171	0.47712	2.7202	0.00652	0.01943	TRUE	4930431P19Rik
Gm45148	116.4622	1.74657185	0.41765	4.18193	2.89E-05	0.00017	TRUE	Gm45148
Gm45200	31.29261	2.324455839	0.58906	3.94607	7.94E-05	0.00041	TRUE	Gm45200
Gm44744	4.491251	4.743413315	1.61792	2.93179	0.00337	0.01108	TRUE	Gm44744
Dcst2	26.55055	2.58673091	0.61483	4.20725	2.58E-05	0.00015	TRUE	Dcst2
Smim38	15.60038	2.255695732	0.79676	2.83109	0.00464	0.01456	TRUE	Smim38
Samd4b	2137.943	1.372296444	0.32659	4.20187	2.65E-05	0.00016	TRUE	Samd4b

Gm19410	7.909359	3.462597335	1.07747	3.21365	0.00131	0.00488	TRUE		Gm19410
Gm45073	40.47014	3.826446499	0.50555	7.5689	3.76E-14	1.01E-12	TRUE		Gm45073
Gm44763	29.46599	2.066920106	0.62066	3.3302	0.00087	0.00341	TRUE		Gm44763
Gm45289	45.18864	2.056301006	0.63735	3.22633	0.00125	0.0047	TRUE		Gm45289
Gm45510	25.7828	1.648905852	0.62734	2.62842	0.00858	0.0245	TRUE		Gm45510
Svet1	379.6586	4.930737461	0.45043	10.9467	6.89E-28	7.68E-26	TRUE		Svet1
Gm45342	13.83729	2.438573178	0.94627	2.57703	0.00997	0.02779	TRUE		Gm45342
Gm45483	73.07645	2.03401954	0.42281	4.81072	1.50E-06	1.18E-05	TRUE		Gm45483
Gm45299	5.303619	6.008840905	2.08267	2.88516	0.00391	0.0126	TRUE		Gm45299
Gm45479	60.40348	2.621269624	0.45508	5.75998	8.41E-09	1.06E-07	TRUE		Gm45479
Gm45509	46.74276	2.162630926	0.6254	3.458	0.00054	0.00225	TRUE		Gm45509
Gm45338	19.63003	2.355281835	0.74534	3.16	0.00158	0.00575	TRUE		Gm45338
Olfr1279	2.460124	6.19472915	2.46168	2.51647	0.01185	0.03217	TRUE		Olfr1279
Gm45643	15.3648	4.930717816	1.14545	4.30462	1.67E-05	0.0001	TRUE		Gm45643
Gm9911	7.445835	5.276253459	1.36385	3.86863	0.00011	0.00055	TRUE		Gm9911
Gm18706	5.356721	7.336555279	1.8366	3.99464	6.48E-05	0.00035	TRUE		Gm18706
Gm38948	122.904	2.435395015	0.45292	5.37705	7.57E-08	7.95E-07	TRUE		Gm38948
Gm45570	37.59394	2.371143329	0.61431	3.85983	0.00011	0.00057	TRUE		Gm45570
Gm45292	17.72071	1.517796101	0.64938	2.33729	0.01942	0.04851	TRUE		Gm45292
Gm6145	17.68247	3.529992188	0.99456	3.54932	0.00039	0.00166	TRUE		Gm6145
Gm45394	21.39915	4.268572796	0.80951	5.27306	1.34E-07	1.34E-06	TRUE		Gm45394
831440D22R	25.78188	2.111279395	0.72421	2.9153	0.00355	0.01162	TRUE		4831440D22Rik
Gm45501	177.2619	1.532895681	0.33884	4.52396	6.07E-06	4.14E-05	TRUE		Gm45501
Gm45560	38.57195	1.631230506	0.45162	3.61195	0.0003	0.00136	TRUE		Gm45560
Gm45494	5.317344	4.275502194	1.26532	3.37898	0.00073	0.00291	TRUE		Gm45494
Gm45532	20.57544	3.798089828	0.77076	4.92772	8.32E-07	6.96E-06	TRUE		Gm45532
Gm20219	101.3723	1.391454799	0.43441	3.20312	0.00136	0.00505	TRUE		Gm20219
Gm45486	5.461184	7.013214244	1.95882	3.58033	0.00034	0.0015	TRUE		Gm45486
Gm45652	14.47952	4.765291234	0.99813	4.77423	1.80E-06	1.39E-05	TRUE		Gm45652
930086L07R	147.6202	1.717124008	0.482	3.5625	0.00037	0.00159	TRUE		B930086L07Rik
Gm45380	92.95898	2.728921516	0.57637	4.7347	2.19E-06	1.66E-05	TRUE		Gm45380
Gm18991	36.21479	3.86821446	0.6702	5.77177	7.84E-09	9.91E-08	TRUE		Gm18991
Rnf223	126.637	1.826092081	0.31543	5.78928	7.07E-09	9.00E-08	TRUE		Rnf223
Gm9908	5.220799	5.198315478	2.04119	2.54671	0.01087	0.02994	TRUE		Gm9908
700012D14R	84.5216	1.048724007	0.36073	2.90725	0.00365	0.01187	TRUE		1700012D14Rik
Gm45820	31.32441	2.284565951	0.69254	3.29882	0.00097	0.00376	TRUE		Gm45820
Gm35857	8.720288	3.468691313	1.13334	3.0606	0.00221	0.00772	TRUE		Gm35857
Gm45774	2.102316	5.044495783	2.16849	2.32627	0.02	0.04965	TRUE		Gm45774
Gm45834	6.14579	7.375432408	1.59887	4.6129	3.97E-06	2.83E-05	TRUE		Gm45834
Gm32856	89.48174	2.435903254	0.46602	5.22705	1.72E-07	1.67E-06	TRUE		Gm32856
Gm39822	5.858717	7.225279942	2.65963	2.71664	0.00659	0.01959	TRUE		Gm39822
Gm42047	34814.43	3.307107054	0.44391	7.44997	9.34E-14	2.38E-12	TRUE		Gm42047
Gm45855	20.91146	1.785065925	0.59128	3.01898	0.00254	0.00871	TRUE		Gm45855
Gm33460	148.6334	5.772763659	0.77977	7.40317	1.33E-13	3.33E-12	TRUE		Gm33460
230053D17R	35.04493	2.15473603	0.50134	4.29798	1.72E-05	0.00011	TRUE		C230053D17Rik
Gm6607	70.18571	4.141917235	0.59563	6.9538	3.56E-12	7.49E-11	TRUE		Gm6607
Gm19178	19.85446	3.006298112	0.99928	3.00846	0.00263	0.00897	TRUE		Gm19178
Gm34655	32.21289	1.981415774	0.48889	4.05292	5.06E-05	0.00028	TRUE		Gm34655
Gm48114	35.05422	1.505411561	0.57439	2.62091	0.00877	0.02495	TRUE		Gm48114
Gm9856	48.38581	2.048592773	0.50449	4.06069	4.89E-05	0.00027	TRUE		Gm9856
Gm32468	4.809718	3.375230887	1.45011	2.32757	0.01993	0.0495	TRUE		Gm32468
Zkscan7.1	1021.836	1.835334402	0.45542	4.02995	5.58E-05	0.0003	TRUE		Zkscan7.1
Gm47676	11.80652	3.676392725	1.06993	3.4361	0.00059	0.00242	TRUE		Gm47676
Gm34425	13.17867	5.468115099	1.06837	5.11819	3.08E-07	2.83E-06	TRUE		Gm34425
Gm49338	748.3949	1.313764251	0.45083	2.91409	0.00357	0.01166	TRUE		Gm49338
Gm47087	334.4645	2.792145186	0.48369	5.77262	7.80E-09	9.88E-08	TRUE		Gm47087
Gm48717	57.14668	1.98131376	0.4367	4.53701	5.71E-06	3.92E-05	TRUE		Gm48717
Gm48562	21.5976	1.355041695	0.56817	2.38491	0.01708	0.04359	TRUE		Gm48562
Gm47772	10.8636	2.955966413	1.00597	2.93843	0.0033	0.01087	TRUE		Gm47772
Gm48796	412.9157	1.903563636	0.27197	6.99913	2.58E-12	5.53E-11	TRUE		Gm48796
Gm47643	22.12873	2.414447359	0.61242	3.94248	8.06E-05	0.00042	TRUE		Gm47643
Gm47324	8.604436	2.190390542	0.78769	2.78078	0.00542	0.01664	TRUE		Gm47324
Gm47121	38.69496	2.421799703	0.60576	3.99793	6.39E-05	0.00034	TRUE		Gm47121
Gm47199	34.18837	2.145361801	0.66468	3.22767	0.00125	0.00468	TRUE		Gm47199
Gm47232	22.52338	2.840990041	0.64656	4.39401	1.11E-05	7.15E-05	TRUE		Gm47232
Gm34885	15.96168	3.221681483	1.23138	2.61633	0.00889	0.02524	TRUE		Gm34885
Gm48183	9.612427	2.628676544	0.84828	3.09885	0.00194	0.00689	TRUE		Gm48183
Gm47023	28.84081	2.592267225	0.51499	5.03359	4.81E-07	4.26E-06	TRUE		Gm47023
Gm5176	43.52212	2.004176802	0.65703	3.05035	0.00229	0.00796	TRUE		Gm5176
Gm18119	9.142267	4.790521922	1.38949	3.44769	0.00057	0.00233	TRUE		Gm18119
Gm48808	36.05967	2.832766963	0.55488	5.10516	3.31E-07	3.01E-06	TRUE		Gm48808
Gm48181	328.2197	2.437131651	0.53448	4.55979	5.12E-06	3.55E-05	TRUE		Gm48181
Gm47018	37.84016	1.70645989	0.54639	3.12316	0.00179	0.0064	TRUE		Gm47018
Gm47026	190.57	1.85319428	0.40107	4.62062	3.83E-06	2.74E-05	TRUE		Gm47026
Gm47022	12.89389	2.159440949	0.80094	2.69612	0.00702	0.02063	TRUE		Gm47022

Gm35405	15.5653	3.02081454	1.02238	2.95469	0.00313	0.0104	TRUE	Gm35405
Gm17823	111.9109	1.607120952	0.49903	3.2205	0.00128	0.00478	TRUE	Gm17823
Gm47623	82.28769	3.209067179	0.50114	6.40358	1.52E-10	2.51E-09	TRUE	Gm47623
Gm48353	80.47376	2.115438658	0.47769	4.42849	9.49E-06	6.19E-05	TRUE	Gm48353
Gm8153	25.06467	2.582621973	0.59264	4.35785	1.31E-05	8.31E-05	TRUE	Gm8153
Gm33843	15.60176	4.23727665	1.08911	3.89057	0.0001	0.00051	TRUE	Gm33843
Gm48507	3.425627	6.556303856	1.83672	3.56957	0.00036	0.00156	TRUE	Gm48507
Gm8170	74.46455	1.942520312	0.44629	4.35262	1.35E-05	8.51E-05	TRUE	Gm8170
030006N10R	21.95632	2.187514123	0.58004	3.77133	0.00016	0.00078	TRUE	C030006N10Rik
Gm30122	31.73137	1.449429837	0.62189	2.33069	0.01977	0.04917	TRUE	Gm30122
Gm48182	124.0392	1.715124462	0.40675	4.21663	2.48E-05	0.00015	TRUE	Gm48182
Gm48207	16.93488	2.590708291	1.04467	2.47992	0.01314	0.03501	TRUE	Gm48207
Gm47621	543.3931	3.234237147	0.36429	8.87812	6.80E-19	3.10E-17	TRUE	Gm47621
Gm47340	354.2823	1.507160196	0.42455	3.55001	0.00039	0.00166	TRUE	Gm47340
Gm5173	43.34827	2.290084536	0.51654	4.43352	9.27E-06	6.07E-05	TRUE	Gm5173
933412E12R	17.90744	2.600290789	0.58798	4.42241	9.76E-06	6.35E-05	TRUE	4933412E12Rik
Gm48225	22.91661	1.523558942	0.62153	2.4513	0.01423	0.03745	TRUE	Gm48225
730063M14R	160.4082	1.755559499	0.4682	3.74961	0.00018	0.00084	TRUE	A730063M14Rik
Gm47644	62.08632	1.848592589	0.49315	3.74858	0.00018	0.00084	TRUE	Gm47644
Gm48880	13.73183	2.238607243	0.75364	2.97039	0.00297	0.00998	TRUE	Gm48880
Gm47615	40.30034	2.284501071	0.58673	3.89364	9.88E-05	0.0005	TRUE	Gm47615
Gm34304	4.267381	5.246657332	1.75409	2.99109	0.00278	0.00942	TRUE	Gm34304
Gm48210	20.13892	2.419780676	0.85915	2.81649	0.00486	0.01514	TRUE	Gm48210
Gm18029	25.03849	2.07116393	0.63175	3.27844	0.00104	0.004	TRUE	Gm18029
Gm48146	49.31147	1.106384901	0.4736	2.33612	0.01948	0.04861	TRUE	Gm48146
130012E19R	16.36305	1.713292526	0.66479	2.57718	0.00996	0.02778	TRUE	A130012E19Rik
Gm47098	19.85518	3.010202473	0.89042	3.38065	0.00072	0.0029	TRUE	Gm47098
Gm47704	3.459827	3.740160932	1.4392	2.59878	0.00936	0.02635	TRUE	Gm47704
Gm32834	26.24486	2.930026469	0.64974	4.50952	6.50E-06	4.40E-05	TRUE	Gm32834
Gm48041	25.31578	1.7834345	0.73868	2.41436	0.01576	0.04081	TRUE	Gm48041
Gm47813	20.63932	2.215112864	0.65185	3.39821	0.00068	0.00274	TRUE	Gm47813
Gm7511	37.66983	2.108561599	0.4349	4.84836	1.24E-06	9.99E-06	TRUE	Gm7511
930474N09R	13.96507	3.436611246	1.08234	3.17517	0.0015	0.0055	TRUE	4930474N09Rik
Gm47251	37.25262	1.90514515	0.43639	4.36565	1.27E-05	8.05E-05	TRUE	Gm47251
Gm30409	10.09715	4.041281187	1.54435	2.61682	0.00888	0.02521	TRUE	Gm30409
Gm48551	14.3266	2.233196812	0.81409	2.74318	0.00608	0.01835	TRUE	Gm48551
Gm32635	5.813374	2.851570113	1.22662	2.32474	0.02009	0.04981	TRUE	Gm32635
Gm46378	37.81722	2.378381697	0.5335	4.45803	8.27E-06	5.48E-05	TRUE	Gm46378
Gm46350	102.3847	2.94390448	0.54032	5.44845	5.08E-08	5.54E-07	TRUE	Gm46350
Gm36756	8.326743	2.087676701	0.89795	2.32495	0.02007	0.04979	TRUE	Gm36756
Gm47076	11.2599	1.770031829	0.74012	2.39156	0.01678	0.04293	TRUE	Gm47076
Gm47370	30.09148	2.710532852	0.7507	3.61068	0.00031	0.00136	TRUE	Gm47370
Gm19327	17.46128	4.044862135	0.85179	4.74867	2.05E-06	1.55E-05	TRUE	Gm19327
Gm33195	36.35209	2.838311194	0.5566	5.09937	3.41E-07	3.09E-06	TRUE	Gm33195
Gm48017	5.107896	4.447843948	1.3835	3.21493	0.0013	0.00486	TRUE	Gm48017
Gm19554	161.3187	6.07891823	0.43758	13.8921	7.07E-44	2.78E-41	TRUE	Gm19554
Gm47208	43.46989	2.843883758	0.55473	5.12657	2.95E-07	2.71E-06	TRUE	Gm47208
Gm48838	74.49676	1.96586805	0.46401	4.23666	2.27E-05	0.00014	TRUE	Gm48838
Gm35755	10.52569	6.206196158	1.46769	4.22856	2.35E-05	0.00014	TRUE	Gm35755
Gm48890	157.5615	1.208203935	0.39338	3.07131	0.00213	0.00747	TRUE	Gm48890
Gm48319	56.47703	3.143109893	0.53785	5.84382	5.10E-09	6.66E-08	TRUE	Gm48319
930549C15R	49.4594	2.079000428	0.45616	4.55761	5.17E-06	3.58E-05	TRUE	4930549C15Rik
Gm47424	27.56914	2.986388909	0.9117	3.27564	0.00105	0.00403	TRUE	Gm47424
Gm36423	104.7519	2.25432952	0.46194	4.88011	1.06E-06	8.66E-06	TRUE	Gm36423
Gm48603	27.4799	1.990354073	0.57224	3.47816	0.0005	0.00211	TRUE	Gm48603
Gm29844	24.60383	1.818658039	0.71758	2.53443	0.01126	0.03084	TRUE	Gm29844
Gm48600	73.39653	1.142031871	0.42333	2.69774	0.00698	0.02055	TRUE	Gm48600
Hist1h2bm	16.95023	2.408533586	0.73623	3.27143	0.00107	0.00409	TRUE	Hist1h2bm
630015K15R	15.87231	3.145931771	0.87915	3.57839	0.00035	0.00151	TRUE	9630015K15Rik
Gm47773	69.70694	4.690599248	0.46804	10.0218	1.22E-23	9.13E-22	TRUE	Gm47773
Gm48683	32.63895	3.058881228	0.66249	4.61723	3.89E-06	2.78E-05	TRUE	Gm48683
Gm9042	11.20118	7.143734909	1.50413	4.74942	2.04E-06	1.55E-05	TRUE	Gm9042
630044B11R	4.686974	6.885996706	2.75838	2.49639	0.01255	0.03369	TRUE	C630044B11Rik
530065N20R	2.047643	5.971341068	2.43232	2.45499	0.01409	0.03715	TRUE	A530065N20Rik
Gm48500	5.111451	6.963092282	2.24083	3.10737	0.00189	0.00672	TRUE	Gm48500
Gm31683	7.852522	2.846785846	1.00187	2.84149	0.00449	0.01414	TRUE	Gm31683
Gm18760	3.478329	5.585012201	2.14051	2.6092	0.00908	0.02568	TRUE	Gm18760
Gm48397	4.64526	7.12043116	1.94482	3.66123	0.00025	0.00115	TRUE	Gm48397
Gm36101	28.93353	7.847800733	1.1882	6.60475	3.98E-11	7.22E-10	TRUE	Gm36101
Gm21388	5.95406	4.485503474	1.20612	3.71896	0.0002	0.00094	TRUE	Gm21388
Gm41192	31.25717	4.650480007	0.75537	6.15652	7.44E-10	1.11E-08	TRUE	Gm41192
Gm4118	13.10637	3.479373442	1.42526	2.44122	0.01464	0.03833	TRUE	Gm4118
Gm49226	33.1177	1.607245168	0.56416	2.84891	0.00439	0.01386	TRUE	Gm49226
Gm49172	359.6086	1.685962165	0.36137	4.66544	3.08E-06	2.26E-05	TRUE	Gm49172
Gm36899	27.3388	2.599320207	0.65297	3.98076	6.87E-05	0.00036	TRUE	Gm36899

Eef1akmt4	51.18603	1.171954003	0.43449	2.69729	0.00699	0.02057	TRUE		Eef1akmt4
Gm48996	5.982992	4.78731802	1.72292	2.77861	0.00546	0.01673	TRUE		Gm48996
530050E16R	54.08921	2.050806654	0.45624	4.49498	6.96E-06	4.68E-05	TRUE		9630050E16Rik
930017N22F	323.485	1.71074952	0.28886	5.92233	3.17E-09	4.26E-08	TRUE		9930017N22Rik
Gm6330	22.61695	2.444617684	0.75197	3.25094	0.00115	0.00436	TRUE		Gm6330
Gm48966	10.1102	2.302868603	0.77338	2.97767	0.0029	0.00978	TRUE		Gm48966
Gm49221	35.75245	8.018793486	1.34127	5.97851	2.25E-09	3.08E-08	TRUE		Gm49221
Gm49266	117.5745	2.231878599	0.41491	5.37924	7.48E-08	7.87E-07	TRUE		Gm49266
Gm3219	13.00721	1.756669434	0.66774	2.63077	0.00852	0.02435	TRUE		Gm3219
Gm49025	4.053082	4.692017366	1.92271	2.44032	0.01467	0.03839	TRUE		Gm49025
Gm10791	257.4443	2.119582217	0.34403	6.16097	7.23E-10	1.09E-08	TRUE		Gm10791
Gm49307	27.81992	5.598187007	0.83352	6.71631	1.86E-11	3.55E-10	TRUE		Gm49307
Gm10389	21.71818	2.14519439	0.56994	3.76387	0.00017	0.0008	TRUE		Gm10389
Gm49310	6.633434	3.098519917	0.9808	3.15917	0.00158	0.00576	TRUE		Gm49310
Bc1	185.9324	1.170894941	0.42536	2.75272	0.00591	0.01791	TRUE		Bc1
Gm49066	37.80922	1.769459407	0.55813	3.17035	0.00152	0.00558	TRUE		Gm49066
Gm49309	67.5197	2.649143474	0.65264	4.05914	4.93E-05	0.00027	TRUE		Gm49309
L30002K18F	47.65374	1.194316641	0.36662	3.25761	0.00112	0.00427	TRUE		9130002K18Rik
Gm49478	28.007	2.401895886	0.97039	2.47518	0.01332	0.03542	TRUE		Gm49478
930001M01I	6.009519	3.704512451	1.43071	2.58928	0.00962	0.02699	TRUE		A930001M01Rik
530056E24R	112.8586	2.671305036	0.38915	6.86452	6.67E-12	1.35E-10	TRUE		9530056E24Rik
Gm18494	2.158192	5.760582746	2.14208	2.68925	0.00716	0.02098	TRUE		Gm18494
Gm49539	492.7539	4.662480507	0.5902	7.89986	2.79E-15	8.71E-14	TRUE		Gm49539
Gm49503	108.2517	3.413892767	0.46461	7.34794	2.01E-13	4.92E-12	TRUE		Gm49503
Gm49474	150.3756	1.768619971	0.4414	4.00681	6.15E-05	0.00033	TRUE		Gm49474
Gm49549	10.23569	2.49680032	0.96056	2.59931	0.00934	0.02632	TRUE		Gm49549
Gm49413	183.3182	3.234922549	0.58221	5.55631	2.76E-08	3.15E-07	TRUE		Gm49413
Gm49484	31.23905	2.363581839	0.59079	4.00074	6.31E-05	0.00034	TRUE		Gm49484
Gm49435	22.51561	3.444446536	0.70767	4.86729	1.13E-06	9.18E-06	TRUE		Gm49435
Lncppara	32.57142	3.081154072	0.52434	5.87622	4.20E-09	5.53E-08	TRUE		Lncppara
Gm49525	38.19016	1.181882942	0.49887	2.36914	0.01783	0.04518	TRUE		Gm49525
AC154200.1	168.5665	1.927072189	0.35608	5.41189	6.24E-08	6.67E-07	TRUE		AC154200.1
Gm30124	46.03517	2.318458295	0.50639	4.5784	4.69E-06	3.28E-05	TRUE		Gm30124
Gm8670	5.286318	4.833157985	2.05575	2.35105	0.01872	0.047	TRUE		Gm8670
Gm49774	28.10796	2.285729671	0.77492	2.94965	0.00318	0.01054	TRUE		Gm49774
530089N07F	179.5673	2.513757422	0.54277	4.63135	3.63E-06	2.62E-05	TRUE		A630089N07Rik
Gm49712	11.60785	2.113814579	0.81037	2.60844	0.0091	0.02573	TRUE		Gm49712
Gm49586	8.652524	3.528336309	1.27094	2.77617	0.0055	0.01684	TRUE		Gm49586
Gm49594	582.016	2.346900995	0.53392	4.3956	1.10E-05	7.11E-05	TRUE		Gm49594
Gm6475	131.5056	4.727963804	0.55209	8.56372	1.09E-17	4.40E-16	TRUE		Gm6475
Gm49797	531.7126	1.562274177	0.30576	5.10947	3.23E-07	2.94E-06	TRUE		Gm49797
Gm49717	6.033164	6.484229488	1.70528	3.80243	0.00014	0.0007	TRUE		Gm49717
Gm8387	13.41314	2.98715586	0.79517	3.75662	0.00017	0.00082	TRUE		Gm8387
Gm41611	39.54512	3.017674086	0.61369	4.91729	8.77E-07	7.30E-06	TRUE		Gm41611
Gm49932	11.26603	2.73080235	0.91338	2.98977	0.00279	0.00945	TRUE		Gm49932
Gm49839	66.03517	1.602579627	0.39505	4.05663	4.98E-05	0.00027	TRUE		Gm49839
Gm49960	22.64088	2.187430223	0.66069	3.31084	0.00093	0.00362	TRUE		Gm49960
Gm32432	12.20824	2.647672193	0.88498	2.9918	0.00277	0.0094	TRUE		Gm32432
Gm49980	1193.139	3.822023893	0.50048	7.63678	2.23E-14	6.19E-13	TRUE		Gm49980
AC154542.1	20.55032	1.759913958	0.7533	2.33628	0.01948	0.04861	TRUE		AC154542.1
Gm50037	8.863252	3.319859817	0.8725	3.80501	0.00014	0.00069	TRUE		Gm50037
Gm50069	57.13461	1.249917665	0.40454	3.08973	0.002	0.00708	TRUE		Gm50069
Gm550	59.60311	2.168551257	0.46931	4.62075	3.82E-06	2.74E-05	TRUE		Gm550
Gm50232	229.1474	2.208162013	0.46167	4.78295	1.73E-06	1.34E-05	TRUE		Gm50232
Gm25432	47.6831	2.198603076	0.41235	5.33187	9.72E-08	9.98E-07	TRUE		Gm25432
Gm50360	8.272098	4.519867968	1.20896	3.73865	0.00019	0.00087	TRUE		Gm50360
Gm50213	10.93009	5.698466424	1.31988	4.3174	1.58E-05	9.82E-05	TRUE		Gm50213
AC109619.1	263.216	2.774238581	0.27719	10.0086	1.40E-23	1.03E-21	TRUE		AC109619.1
030004G16F	10.53511	3.39412448	1.11165	3.05324	0.00226	0.00789	TRUE		C030004G16Rik
Gm6402	122.0017	2.424306095	0.53306	4.54791	5.42E-06	3.74E-05	TRUE		Gm6402
Gm8663	28.99767	3.676751279	0.96928	3.79328	0.00015	0.00072	TRUE		Gm8663
Gm46620	768.6029	2.935817756	0.45944	6.39001	1.66E-10	2.72E-09	TRUE		Gm46620
Rbfaos	11.55082	2.007950571	0.71586	2.80494	0.00503	0.01561	TRUE		Rbfaos
AC166172.1	126.9304	2.041502334	0.54302	3.75954	0.00017	0.00081	TRUE		AC166172.1
833438C02F	30.69498	3.174130469	0.60857	5.21568	1.83E-07	1.77E-06	TRUE		4833438C02Rik
Gm34455	4.686974	6.885996706	2.75838	2.49639	0.01255	0.03369	TRUE		Gm34455
Gm46649	31.59318	2.479570452	0.49721	4.98693	6.13E-07	5.30E-06	TRUE		Gm46649
Gm50131	4.001254	4.811725585	1.70972	2.81434	0.00489	0.01522	TRUE		Gm50131
Gm50172	4.28944	6.644247058	1.99846	3.32469	0.00089	0.00346	TRUE		Gm50172
Tmem179b	121.8186	1.044960174	0.26438	3.95255	7.73E-05	0.0004	TRUE		Tmem179b
Gm8373	65.02068	3.275232429	0.67942	4.82064	1.43E-06	1.13E-05	TRUE		Gm8373
AL844494.1	6.450458	5.505205679	1.42116	3.87373	0.00011	0.00054	TRUE		AL844494.1
AL844494.3	29.29525	2.967542102	0.58584	5.06543	4.07E-07	3.65E-06	TRUE		AL844494.3
