# Supplementary material for: Temporal-spatial Generation of Astrocytes in the Developing Diencephalon
Source: Neurosci Bull. 2023 Oct 16;40(1):1–16. doi: 10.1007/s12264-023-01131-9 (PMC10774245; doi:10.1007/s12264-023-01131-9)
Supplement: Supplementary file 5 — Supplementary file5 (PDF 182 kb) [file 12264_2023_1131_MOESM5_ESM.pdf]

Table S5. A custom geneset that represents positive regulation of astrogenesis and astrocyte development

Gene name	Entrez ID	Citation	Title
Atg5	11793	EMBO Rep. 2014 Oct;15(10):1053-61. doi: 10.15252/embr.201338343. Epub 2014 S	Autophagy-related gene Atg5 is essential for astrocyte differentiation in the developing mouse cortex.
CSL	71832	J Neurosci Res. 2002 Sep 15;69(6):848-60.	Notch signaling promotes astrogliogenesis via direct CSL-mediated glial gene activation.
CTGF(CCN2)	14219	PLoS One. 2015 Aug 4;10(8):e0133689. doi: 10.1371/journal.pone.0133689. eCollec	Connective-Tissue Growth Factor (CTGF/CCN2) Induces Astrogenesis and Fibronectin Expression of Embryonic Neural Cells In Vitro
GLI3	14634	J Neurosci. 2013 Oct 30; 33(44): 17490–17505.	Titration of GLI3 Repressor Activity by Sonic Hedgehog Signaling Is Critical for Maintaining Multiple Adult Neural Stem Cell and Astrocyte Functions
Id3	15903	EMBO J. 2015 Nov 12;34(22):2804-19. doi: 10.15252/embj.201591118. Epub 2015	The balance of Id3 and E47 determines neural stem/precursor cell differentiation into astrocytes.
Abr	109934	Development. 2001 Nov;128(21):4217-27.	Abnormal function of astroglia lacking Abr and Bcr RacGAPs.
ADAM-17	11491	Stem Cells. 2011 Oct;29(10):1628-39. doi: 10.1002/stem.710.	ADAM-17/tumor necrosis factor-α-converting enzyme inhibits neurogenesis and promotes gliogenesis from neural stem cells.
Aldh1a1	11668	Brain Pathol. 2012 Nov;22(6):788-97. doi: 10.1111/j.1750-3639.2012.00592.x. Epub	ALDH1A1 is a marker of astrocytic differentiation during brain development and correlates with better survival in glioblastoma patients.
Aldh1l1	107747	Glia. 2013 Sep;61(9):1518-32. doi: 10.1002/glia.22538. Epub 2013 Jul 10.	Expression profiling of Aldh1l1-precursors in the developing spinal cord reveals glial lineage-specific genes and direct Sox9-Nfe2l1 interactions.
Apoe	11816	Proc Natl Acad Sci U S A. 2016 Sep 6;113(36):10186-91. doi: 10.1073/pnas.1609896	Novel allele-dependent role for APOE in controlling the rate of synapse pruning by astrocytes
Aqp4	11829	CNS Neurosci Ther. 2014 May;20(5):391-402. doi: 10.1111/cns.12222. Epub 2014 Ja	Aquaporin-4 knockout exacerbates corticosterone-induced depression by inhibiting astrocyte function and hippocampal neurogenesis.
Arrb1	109689	Sci Rep. 2015 Oct 26;5:15506. doi: 10.1038/srep15506.	Astroglial β-Arrestin1-mediated Nuclear Signaling Regulates the Expansion of Neural Precursor Cells in Adult Hippocampus.
Astn1	11899	Science. 1996 Apr 19;272(5260):417-9	CNS gene encoding astrotactin, which supports neuronal migration along glial fibers.
Astn2	56079	J Neurosci. 2010 Jun 23;30(25):8529-40. doi: 10.1523/JNEUROSCI.0032-10.2010.	Astn2, a novel member of the astrotactin gene family, regulates the trafficking of ASTN1 during glial-guided neuronal migration.
Atg7	74244	Exp Neurobiol. 2019 Apr;28(2):229-246. doi: 10.5607/en.2019.28.2.229. Epub 2019	Autophagy Mediates Astrogenesis in Adult Hippocampal Neural Stem Cells
BMP2	12156	J Neurosci. 2016 May 25;36(21):5833-49. doi: 10.1523/JNEUROSCI.4487-15.2016.	Neogenin Promotes BMP2 Activation of YAP and Smad1 and Enhances Astrocytic Differentiation in Developing Mouse Neocortex.
BMP4	12159	Front Neurosci. 2014 Apr 10;8:74. doi: 10.3389/fnins.2014.00074. eCollection 2014.	Orphan nuclear receptor TLX regulates astrogenesis by modulating BMP signaling
Caveolin-1	12389	Biochemical and Biophysical Research CommunicationsVolume 407, Issue 3, 15 April	Caveolin-1 promote astroglial differentiation of neural stem/progenitor cells through modulating Notch1/NICD and Hes1 expressions
CD44	12505	PLoS One. 2016 May 10;11(5):e0155053. doi: 10.1371/journal.pone.0155053. eColle	Cleavage of Hyaluronan and CD44 Adhesion Molecule Regulate Astrocyte Morphology via Rac1 Signalling.
Cdkn1c	12577	Development. 2012 Sep;139(18):3306-15. doi: 10.1242/dev.074518. Epub 2012 Aug	p57kip2 regulates glial fate decision in adult neural stem cells.
Chl1	12661	Glia. 2010 Feb;58(3):315-28. doi: 10.1002/glia.20925.	Phosphatidylinositol 3-kinase/protein kinase Cdelta activation induces close homolog of adhesion molecule L1 (CHL1) expression in cultured astrocytes.

Creb3l1	26427	Nat Commun. 2012 Jul 24;3:967. doi: 10.1038/ncomms1971.	Unfolded protein response, activated by OASIS family transcription factors, promotes astrocyte differentiation
Cryba1	12957	Sci Rep. 2015 Mar 4;5:8755. doi: 10.1038/srep08755.	βA3/A1-crystallin is a critical mediator of STAT3 signaling in optic nerve astrocytes.
DISC1	244667	Development. 2016 Aug 1;143(15):2732-40. doi: 10.1242/dev.133066. Epub 2016 Ju	DISC1 regulates astrogenesis in the embryonic brain via modulation of RAS/MEK/ERK signaling through RASSF7
Dp71	13405	Glia. 2016 May;64(5):716-29. doi: 10.1002/glia.22956. Epub 2015 Dec 29.	Altered astrocyte morphology and vascular development in dystrophin-Dp71-null mice
Egfr	13649	Glia. 2015 Mar;63(3):412-22. doi: 10.1002/glia.22761. Epub 2014 Oct 18.	Emx2 expression levels in NSCs modulate astrogenesis rates by regulating EgfR and Fgf9
Egr1	13653	J Clin Invest. 1994 Apr;93(4):1820-7.	Astrocyte growth is regulated by neuropeptides through Tis 8 and basic fibroblast growth factor.
Epac2	56508	BMB Rep. 2016 Feb;49(2):128-33.	Epac2 contributes to PACAP-induced astrocytic differentiation through calcium ion influx in neural precursor cells.
EphA4	13838	J Neurosci. 2017 Mar 22;37(12):3331-3341. doi: 10.1523/JNEUROSCI.3738-16.201	EphA4 Regulates Neuroblast and Astrocyte Organization in a Neurogenic Niche.
ERK1/ERK2	26417/26413	Int J Dev Neurosci. 2013 Dec;31(8):783-9. doi: 10.1016/j.ijdevneu.2013.09.008. Epu	The role of ERK1/2 in the regulation of proliferation and differentiation of astrocytes in developing brain
Fabp5	16592	Stem Cells. 2012 Jul;30(7):1532-43. doi: 10.1002/stem.1124.	The effects of Fabp7 and Fabp5 on postnatal hippocampal neurogenesis in the mouse.
Fabp7	12140	Glia. 2005 May;50(3):187-97.	Differentiation of radial glia from radial precursor cells and transformation into astrocytes in the developing rat spinal cord.
Fbxw7	50754	J Biol Chem. 2011 Apr 15;286(15):13754-64. doi: 10.1074/jbc.M110.194936. Epub 2	Fbxw7-dependent degradation of Notch is required for control of "stemness" and neuronal-glial differentiation in neural stem cells.
Fgf15	14170	Proc Natl Acad Sci U S A. 2020 Feb 4;117(5):2671-2682. doi: 10.1073/pnas.1913053	Retinal inputs signal astrocytes to recruit interneurons into visual thalamus.
Fgf2	14173	Glia. 2011 May;59(5):708-19. doi: 10.1002/glia.21141. Epub 2011 Feb 14.	Fibroblast growth factor 2 regulates astrocyte differentiation in a region-specific manner in the hindbrain.
Fgf9	14180	Glia. 2015 Mar;63(3):412-22. doi: 10.1002/glia.22761. Epub 2014 Oct 18.	Emx2 expression levels in NSCs modulate astrogenesis rates by regulating EgfR and Fgf9
Fgfr3	14184	Development. 2003 Jan;130(1):93-102.	Fgfr3 expression by astrocytes and their precursors: evidence that astrocytes and oligodendrocytes originate in distinct neuroepithelial domains.
FoxJ1	15223	Development. 2009 Dec;136(23):4021-31. doi: 10.1242/dev.041129.	FoxJ1-dependent gene expression is required for differentiation of radial glia into ependymal cells and a subset of astrocytes in the postnatal brain
FOXP1	108655	Stem Cell Reports. 2017 Nov 14;9(5):1530-1545. doi: 10.1016/j.stemcr.2017.10.012.	FOXP1 Promotes Embryonic Neural Stem Cell Differentiation by Repressing Jagged1 Expression.
Gabbr1	54393	Nature. 2023 May;617(7960):369-376.doi: 10.1038/s41586-023-06010-x. Epub 2023	Inhibitory input directs astrocyte morphogenesis through glial GABABR
Gbx2	14472	Stem Cells Dev. 2019 May 15;28(10):633-648. doi: 10.1089/scd.2019.0033. Epub 20	Generation of Anterior Hindbrain-Specific, Glial-Restricted Progenitor-Like Cells from Human Pluripotent Stem Cells.
Gli1	14632	Hum Mol Genet. 2014 Dec 15;23(24):6512-27. doi: 10.1093/hmg/ddu370. Epub 2014	Deficiency of patched 1-induced Gli1 signal transduction results in astrogenesis in Swedish mutated APP transgenic mice
Glul	14645	Int J Dev Neurosci. 1999 Jun;17(3):173-84.	Selective deletion of glutamine synthetase in the mouse cerebral cortex induces glial dysfunction and vascular impairment that precede epilepsy and neurodegeneration.
GPR37L1	171469	Glia. 2023 Apr 8.doi: 10.1002/glia.24375. Online ahead of print.	GPR37L1 controls maturation and organization of cortical astrocytes during development

H2az1	51788	Nucleic Acids Res. 2018 Sep 28; 46(17): 8817–8831.Published online 2018 Jul 3. doi	H2AZ1 crosstalk with H3K56-acetylation controls gliogenesis through the transcription of folate receptor
Hes1	15205	Dev Dyn. 2003 Apr;226(4):675-89.	Hes1 but not Hes5 regulates an astrocyte versus oligodendrocyte fate choice in glial restricted precursors.
Hes5	15208	Development. 2017 Sep 1;144(17):3156-3167. doi: 10.1242/dev.147256.	Hes5 regulates the transition timing of neurogenesis and gliogenesis in mammalian neocortical development
Id1	15901	Cell Stem Cell. 2009 Nov 6;5(5):515-26. doi: 10.1016/j.stem.2009.08.017.	High levels of Id1 expression define B1 type adult neural stem cells.
Id3	15903	EMBO J. 2015 Nov 12;34(22):2804-19. doi: 10.15252/embj.201591118. Epub 2015	The balance of Id3 and E47 determines neural stem/precursor cell differentiation into astrocytes.
Igf2	16002	Development. 2023 Jan 1;150(1):dev200563.doi: 10.1242/dev.200563. Epub 2023 Ja	IGF2 interacts with the imprinted gene Cdkn1c to promote terminal differentiation of neural stem cells
Il27	246779	Biochem Biophys Res Commun. 2007 Dec 21;364(3):483-7. Epub 2007 Oct 17	Interleukin 27 induces differentiation of neural C6-precursor cells into astrocytes.
Il6st	16195	Elife. 2015 Aug 13;4:e06885. doi: 10.7554/eLife.06885.	Ngn1 inhibits astrogliogenesis through induction of miR-9 during neuronal fate specification.
Jag1	16449	Cell Rep. 2013 Jul 11;4(1):40-9. doi: 10.1016/j.celrep.2013.06.005. Epub 2013 Jul 3.	BACE1 regulates hippocampal astrogenesis via the Jagged1-Notch pathway
Kcnk10	72258	PLoS One. 2015 Apr 17;10(4):e0125195. doi: 10.1371/journal.pone.0125195. eColle	Up-regulation of TREK-2 potassium channels in cultured astrocytes requires de novo protein synthesis: relevance to localization of TREK-2 channels in astrocytes after transient cerebral ischemia
Kir4.1	16513	Nat Neurosci. 2014 May;17(5):694-703. doi: 10.1038/nn.3691. Epub 2014 Mar 30.	Astrocyte Kir4.1 ion channel deficits contribute to neuronal dysfunction in Huntington's disease model mice.
Klf4	16600	Mol Cell Biol. 2012 Nov;32(21):4297-305. doi: 10.1128/MCB.00838-12. Epub 2012	Role of Kruppel-like factor 4 in neurogenesis and radial neuronal migration in the developing cerebral cortex.
LC3	66734	Exp Neurobiol. 2019 Apr;28(2):229-246. doi: 10.5607/en.2019.28.2.229. Epub 2019	Autophagy Mediates Astrogenesis in Adult Hippocampal Neural Stem Cells
Lepr	16847	Mol Metab. 2015 Sep 4;4(11):881-9. doi: 10.1016/j.molmet.2015.08.005. eCollection	Leptin potentiates astrogenesis in the developing hypothalamus
leptin	16846	Mol Metab. 2015 Sep 4;4(11):881-9. doi: 10.1016/j.molmet.2015.08.005. eCollection	Leptin potentiates astrogenesis in the developing hypothalamus
LIF	16878	Brain Res Dev Brain Res. 1995 Jul 14;87(2):220-3.	Effects of leukemia inhibitory factor on the differentiation of astrocyte progenitor cells from embryonic mouse cerebral hemispheres.
Lrp4	228357	Nat Neurosci. 2016 Aug;19(8):1010-8. doi: 10.1038/nn.4326. Epub 2016 Jun 13.	Lrp4 in astrocytes modulates glutamatergic transmission.
MAP3K13	71751	Cell Rep. 2018 Mar 27;22(13):3587-3597. doi: 10.1016/j.celrep.2018.02.102.	Leucine Zipper-Bearing Kinase Is a Critical Regulator of Astrocyte Reactivity in the Adult Mammalian CNS.
MeCP2	17257	eNeuro. 2018 Feb 19;5(1). pii: ENEURO.0194-17.2018. doi: 10.1523/ENEURO.019	MeCP2 Deficiency Leads to Loss of Glial Kir4.1.
NDRG2	29811	J Cell Physiol. 2019 Nov;234(11):20847-20858. doi: 10.1002/jcp.28689. Epub 2019	N-myc downstream-regulated gene 2 controls astrocyte morphology via Rho-GTPase signaling
NFIA	18027	Neuron. 2006 Dec 21;52(6):953-68.	The transcription factor NFIA controls the onset of gliogenesis in the developing spinal cord.
NF-κB	18033	Glia. 2018 Dec;66(12):2659-2672. doi: 10.1002/glia.23518. Epub 2018 Oct 19.	Nuclear factor-kappaB regulates multiple steps of gliogenesis in the developing murine cerebral cortex
Nkx2.1	21869	Sci Rep. 2017 Mar 7;7:43093. doi: 10.1038/srep43093.	Nkx2.1 regulates the generation of telencephalic astrocytes during embryonic development.
Nkx6.1	18096	PLoS One. 2014 Oct 6;9(10):e109171. doi: 10.1371/journal.pone.0109171. eCollecti	Control of astrocyte progenitor specification, migration and maturation by Nkx6.1 homeodomain transcription factor.

Notch1	18128	Science. 2014 Oct 10;346(6206):237-41. doi: 10.1126/science.346.6206.237. Epub 2	A latent neurogenic program in astrocytes regulated by Notch signaling in the mouse.
NPAS3	27386	Cell Rep. 2022 Aug 30;40(9):111289.doi: 10.1016/j.celrep.2022.111289.	Npas3 deficiency impairs cortical astrogenesis and induces autistic-like behaviors
Olig2	50913	Dev Biol. 2008 Aug 15;320(2):456-68. doi: 10.1016/j.ydbio.2008.06.001. Epub 2008	Regional- and temporal-dependent changes in the differentiation of Olig2 progenitors in the forebrain, and the impact on astrocyte development in the dorsal pallium.
P2Y1	18441	J Neurosci Res. 2014 Aug;92(8):1078-90. doi: 10.1002/jnr.23384. Epub 2014 Mar 31	Fibronectin enhances spinal cord astrocyte proliferation by elevating P2Y1 receptor expression.
P53	22059	Glia. 2012 Oct; 60(10): 1579–1589.	P53 is required for the developmental restriction in Muller glial proliferation in mouse retina
PACAP/PAC	11516/13537	Glia. 2007 Feb;55(3):317-27. doi: 10.1002/glia.20461.	PACAP/PAC1 autocrine system promotes proliferation and astrogenesis in neural progenitor cells
PRMT1	15469	J Neurochem. 2017 Sep;142(6):901-907. doi: 10.1111/jnc.14123. Epub 2017 Aug 2.	PRMT1 regulates astrocytic differentiation of embryonic neural stem/precursor cells
Prox1	19130	Stem Cells. 2015 Sep;33(9):2762-72. doi: 10.1002/stem.2055. Epub 2015 Jun 23.	Morphine Promotes Astrocyte-Preferential Differentiation of Mouse Hippocampal Progenitor Cells via PKCε-Dependent ERK Activation and TRBP Phosphorylation.
Ptch1	19206	Neurochem Res. 2016 Feb;41(1-2):278-89. doi: 10.1007/s11064-015-1791-y. Epub 20	Hedgehog Signaling Modulates the Release of Gliotransmitters from Cultured Cerebellar Astrocytes.
RASSF7	66985	Development. 2016 Aug 1;143(15):2732-40. doi: 10.1242/dev.133066. Epub 2016 Ju	DISC1 regulates astrogenesis in the embryonic brain via modulation of RAS/MEK/ERK signaling through RASSF7
RNF20	109331	Cell Death Differ. 2018 Feb;25(2):294-306. doi: 10.1038/cdd.2017.157. Epub 2017 O	RNF20 controls astrocytic differentiation through epigenetic regulation of STAT3 in the developing brain
Rtn4	68585	PLoS One. 2008 Mar 26;3(3):e1856. doi: 10.1371/journal.pone.0001856	Nogo-66 promotes the differentiation of neural progenitors into astroglial lineage cells through mTOR-STAT3 pathway.
S100b	20203	J Biol Chem. 2009 Mar 27;284(13):8797-811. doi: 10.1074/jbc.M805897200. Epub 2	S100B Protein Regulates Astrocyte Shape and Migration via Interaction with Src Kinase: IMPLICATIONS FOR ASTROCYTE DEVELOPMENT, ACTIVATION, AND TUMOR GROWTH.
Shh	20423	Elife. 2019 Jun 13;8. pii: e45545. doi: 10.7554/eLife.45545.	Sonic hedgehog signaling in astrocytes mediates cell type-specific synaptic organization.
Slc17a6	140919	J Neurosci. 2004 Mar 17;24(11):2633-42.	Vesicular glutamate transporter-dependent glutamate release from astrocytes.
Slc17a7	72961	J Neurosci. 2014 Aug 13;34(33):10950-62. doi: 10.1523/JNEUROSCI.1167-14.2014.	VGluT1+ neuronal glutamatergic signaling regulates postnatal developmental maturation of cortical protoplasmic astroglia.
Slc17a8	216227	Glia. 2012 Sep;60(9):1289-300. doi: 10.1002/glia.22348. Epub 2012 May 9.	A distinct set of synaptic-like microvesicles in atroglial cells contain VGLUT3.
Slc1a2	20511	J Neurochem. 2017 Dec;143(5):489-506. doi: 10.1111/jnc.14135. Epub 2017 Sep 5.	Brain endothelial cells induce astrocytic expression of the glutamate transporter GLT-1 by a Notch-dependent mechanism.
Slc1a3	20512	J Neurosci. 1997 Dec 1;17(23):9212-9.	Glutamate transporter GLAST is expressed in the radial glia-astrocyte lineage of developing mouse spinal cord.
Slc7a11	26570	Glia. 2018 May;66(5):951-970. doi: 10.1002/glia.23294. Epub 2018 Jan 19.	The cystine-glutamate exchanger (xCT, Slc7a11) is expressed in significant concentrations in a subpopulation of astrocytes in the mouse brain.
SMAD1	17125	J Neurosci. 2016 May 25;36(21):5833-49. doi: 10.1523/JNEUROSCI.4487-15.2016.	Neogenin Promotes BMP2 Activation of YAP and Smad1 and Enhances Astrocytic Differentiation in Developing Mouse Neocortex.
Smad4	17128	EMBO J. 2021 Nov 2;40(21):e107532.doi: 10.15252/embj.2020107532. Epub 2021	Molecular diversity of diencephalic astrocytes reveals adult astrogenesis regulated by Smad4
Sox9	6662	Genes Dev. 2003 Jul 1; 17(13): 1677–1689.doi: 10.1101/gad.259003	The Sox9 transcription factor determines glial fate choice in the developing spinal cord

Sparc	20692	Development. 2022 Mar 1;149(5):dev199985.doi: 10.1242/dev.199985. Epub 2022 M	Molecular divergence of mammalian astrocyte progenitor cells at early gliogenesis
Sparcl1	13602	Development. 2022 Mar 1;149(5):dev199985.doi: 10.1242/dev.199985. Epub 2022 M	Molecular divergence of mammalian astrocyte progenitor cells at early gliogenesis
STAT3	20848	PLoS One. 2014; 9(1): e86851.	STAT3 but Not STAT1 Is Required for Astrocyte Differentiation
Tal1	21349	Nature. 2005 Nov 17;438(7066):360-3.	Specification of astrocytes by bHLH protein SCL in a restricted region of the neural tube.
Tgfb1	21803	Dev Neurosci. 2012;34(1):68-81. doi: 10.1159/000338108. Epub 2012 May 25.	Activation of MAPK/PI3K/SMAD Pathways by TGF-beta(1) Controls Differentiation of Radial Glia into Astrocytes in vitro
Thbs4	21828	Nature. 2013 May 16;497(7449):369-73. doi: 10.1038/nature12069. Epub 2013 Apr 2	Protective astrogenesis from the SVZ niche after injury is controlled by Notch modulator Thbs4
Tnc	21923	Development. 2011 Dec;138(24):5321-31. doi: 10.1242/dev.067413. Epub 2011 Nov	The extracellular matrix molecule tenascin C modulates expression levels and territories of key patterning genes during spinal cord astrocyte specification.
Tnfrsf19	29820	Eur J Neurosci. 2006 Jun;23(12):3149-60.	Characterization of TROY-expressing cells in the developing and postnatal CNS: the possible role in neuronal and glial cell development.
Trkb	18212	Elife. 2019 Aug 21;8. pii: e44667. doi: 10.7554/eLife.44667.	Astrocyte morphogenesis is dependent on BDNF signaling via astrocytic TrkB.T1
Tsku	244152	Dev Growth Differ. 2020 Feb;62(2):108-117. doi: 10.1111/dgd.12649. Epub 2020 Ja	Tsukushi is essential for proper maintenance and terminal differentiation of mouse hippocampal neural stem cells
Vim	22352	J Neurocytol. 1993 Jul;22(7):558-71.	Immunotyping of radial glia and their glial derivatives during development of the rat spinal cord.
Wnt3	22415	Cell Transplant. 2019 Dec;28(12):1686-1699. doi: 10.1177/0963689719883578. Epu	HBO Promotes the Differentiation of Neural Stem Cells via Interactions Between the Wnt3/β-Catenin and BMP2 Signaling Pathways.
YAP1	10413	Cereb Cortex. 2016 May;26(5):2299-2310. doi: 10.1093/cercor/bhv292. Epub 2015 D	YAP Is a Critical Inducer of SOCS3, Preventing Reactive Astrogliosis.
Zbtb20	56490	Nat Commun. 2016 Mar 22;7:11102. doi: 10.1038/ncomms11102.	Zbtb20 promotes astrocytogenesis during neocortical development.
ZEB2	24136	J Neurosci. 2018 Feb 7;38(6):1575-1587. doi: 10.1523/JNEUROSCI.2674-17.2018.	Transcriptional Regulator ZEB2 Is Essential for Bergmann Glia Development.
