# Supplementary material for: Temporal-spatial Generation of Astrocytes in the Developing Diencephalon
Source: Neurosci Bull. 2023 Oct 16;40(1):1–16. doi: 10.1007/s12264-023-01131-9 (PMC10774245; doi:10.1007/s12264-023-01131-9)
Supplement: Supplementary file 6 — Supplementary file6 (PDF 2515 kb) [file 12264_2023_1131_MOESM6_ESM.pdf]

Supplementary Materials

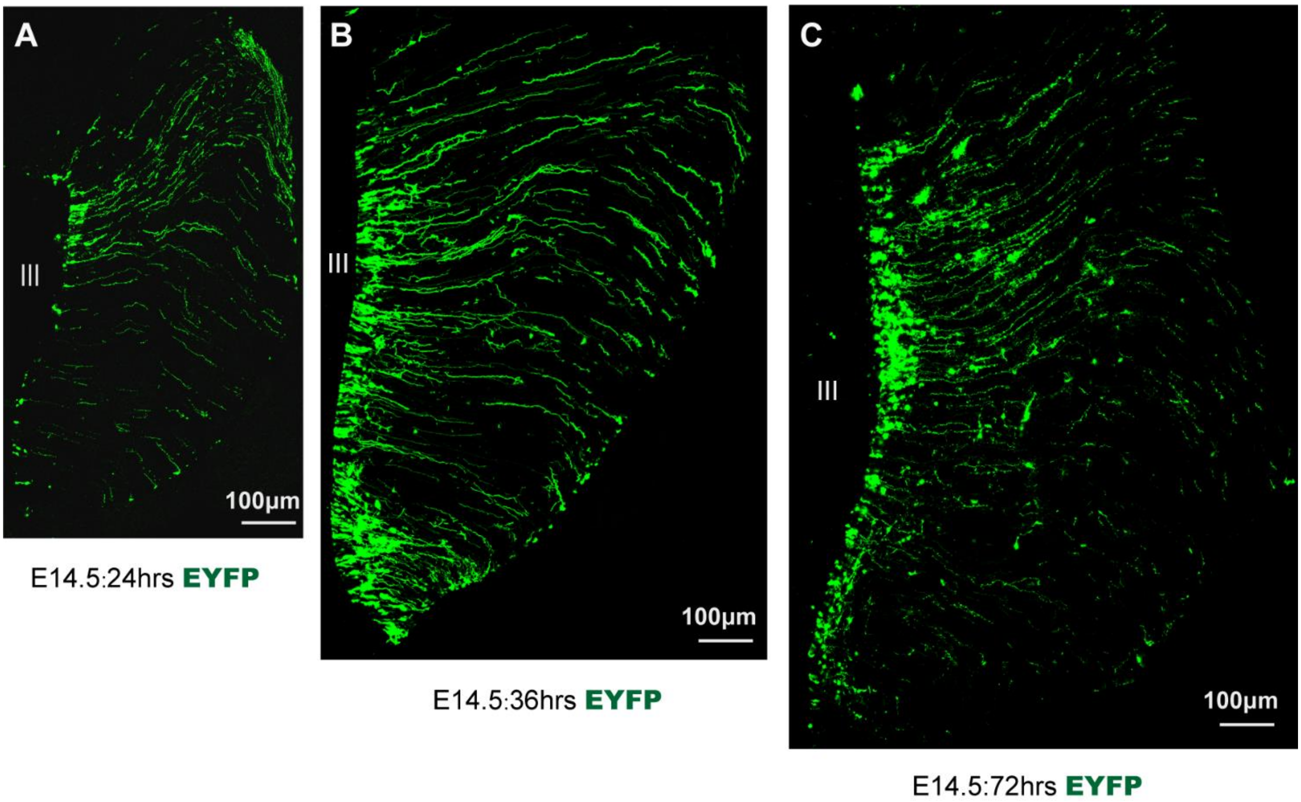

Fig. S1 IUE with CAG-EYFP plasmid in the developing diencephalon at E14.5. **A–C** Images of EYFP-expressing cells in the diencephalon at (**A**) 24 h, (**B**) 36 h, and (**C**) 72 h post-IUE.

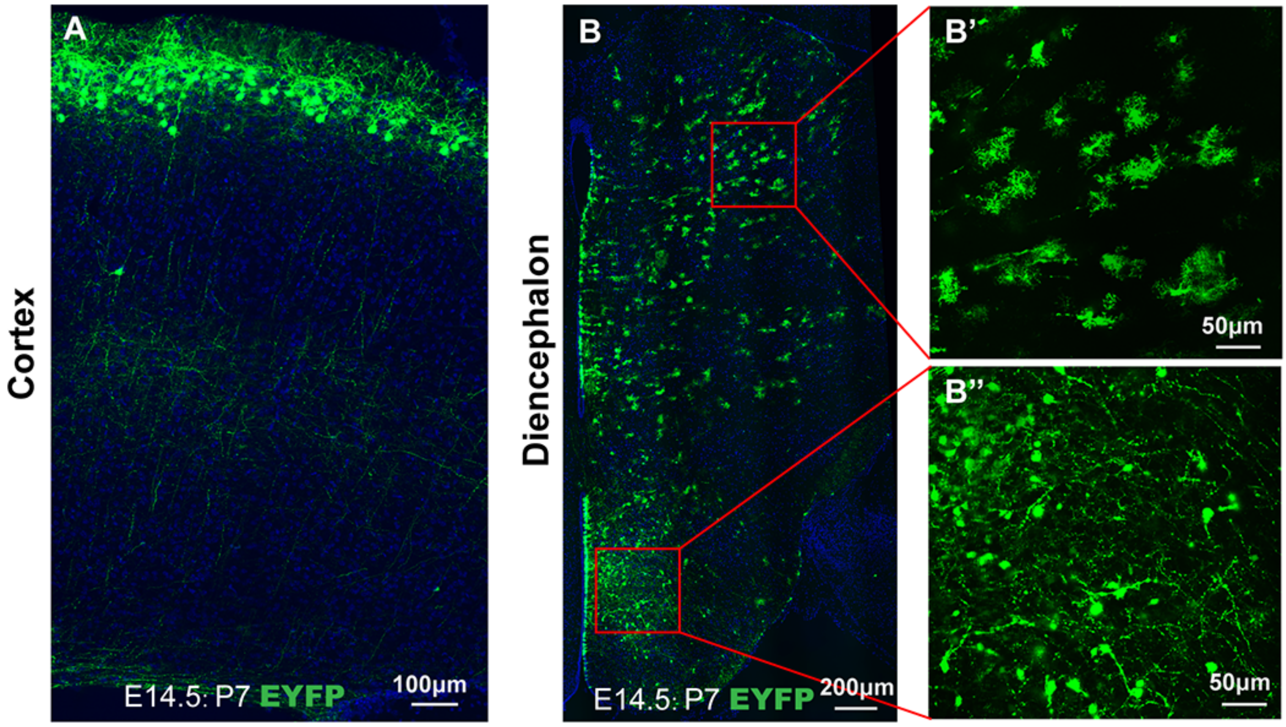

Fig. S2 Tracing of EYFP-expressing cells after IUE at E14.5. **A** NPCs labeled at E14.5 by IUE in the dorsal wall of the LV migrate to the superficial layer (Layer II/III) of the cortex by P7. **B** Two distinct populations of EYFP-expressing cells in the dorsal diencephalon (higher-magnification view in B') and ventral diencephalon (higher-magnification view in B'') at P7. Sections with a blue signal are counterstained with Hoechst.

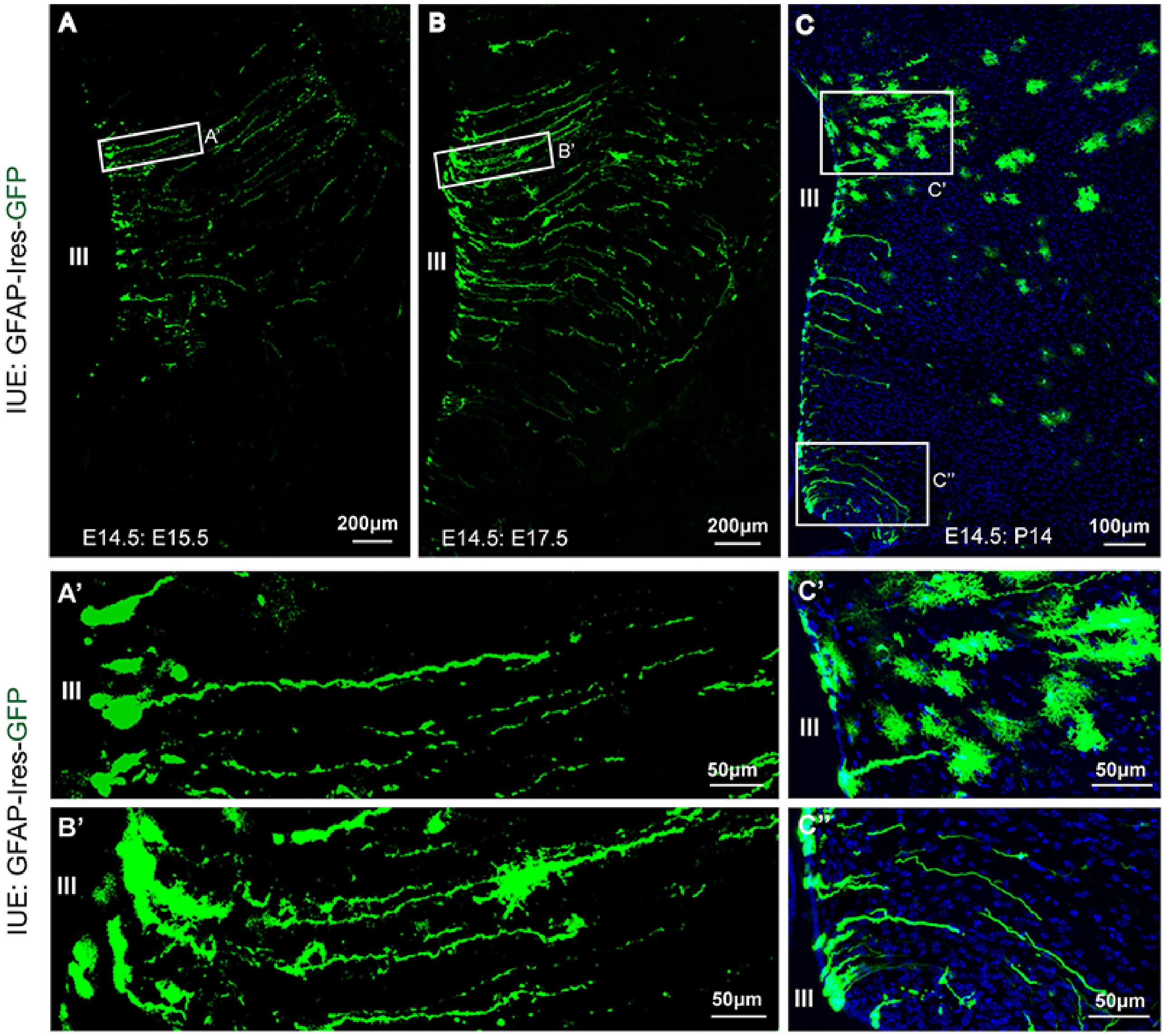

Fig. S3 Tracing of astrocyte specification in the diencephalon with hGFAP-EGFP plasmid by IUE at E14.5. **A–C** Brain sections from (**A**) E15.5, (**B**) E17.5, and (**C**) P14 mice that underwent IUE at E14.5 showed the GFP-expressing cells in the developing diencephalon. Higher-magnification views as indicated in A, B, and C are shown in A', B', C', and C''. Sections with a blue signal are counterstained with Hoechst.

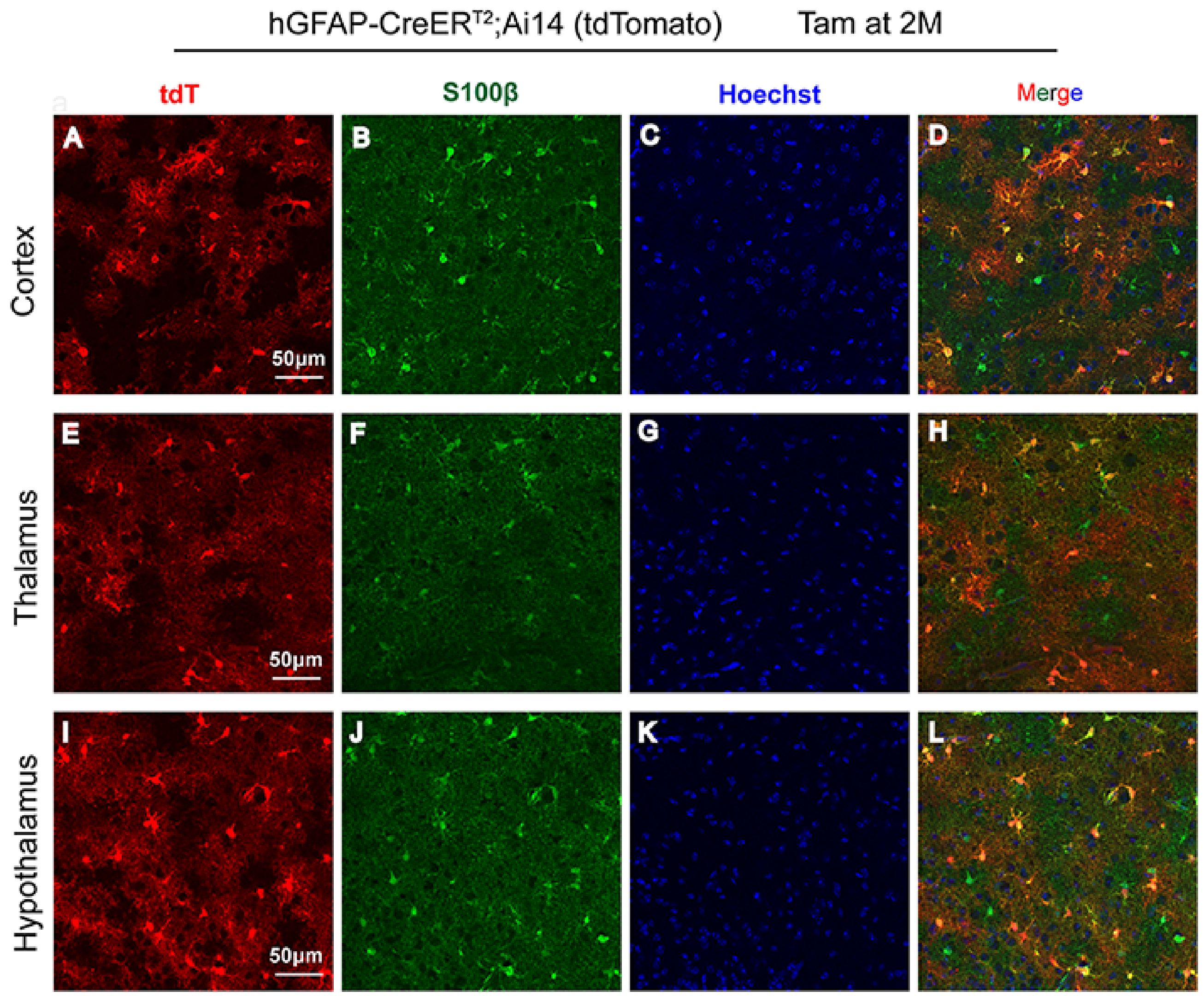

Fig. S4 Astrocyte specificity of *hGFAP-CreER^{T2};Ai14* transgenic mice induced by tamoxifen at 2 months old. **A–L** Immunostaining with S100β of brain sections from electroporated mice in the cortex (**A–D**), thalamus (**E–H**), and hypothalamus (**I–L**), as indicated.

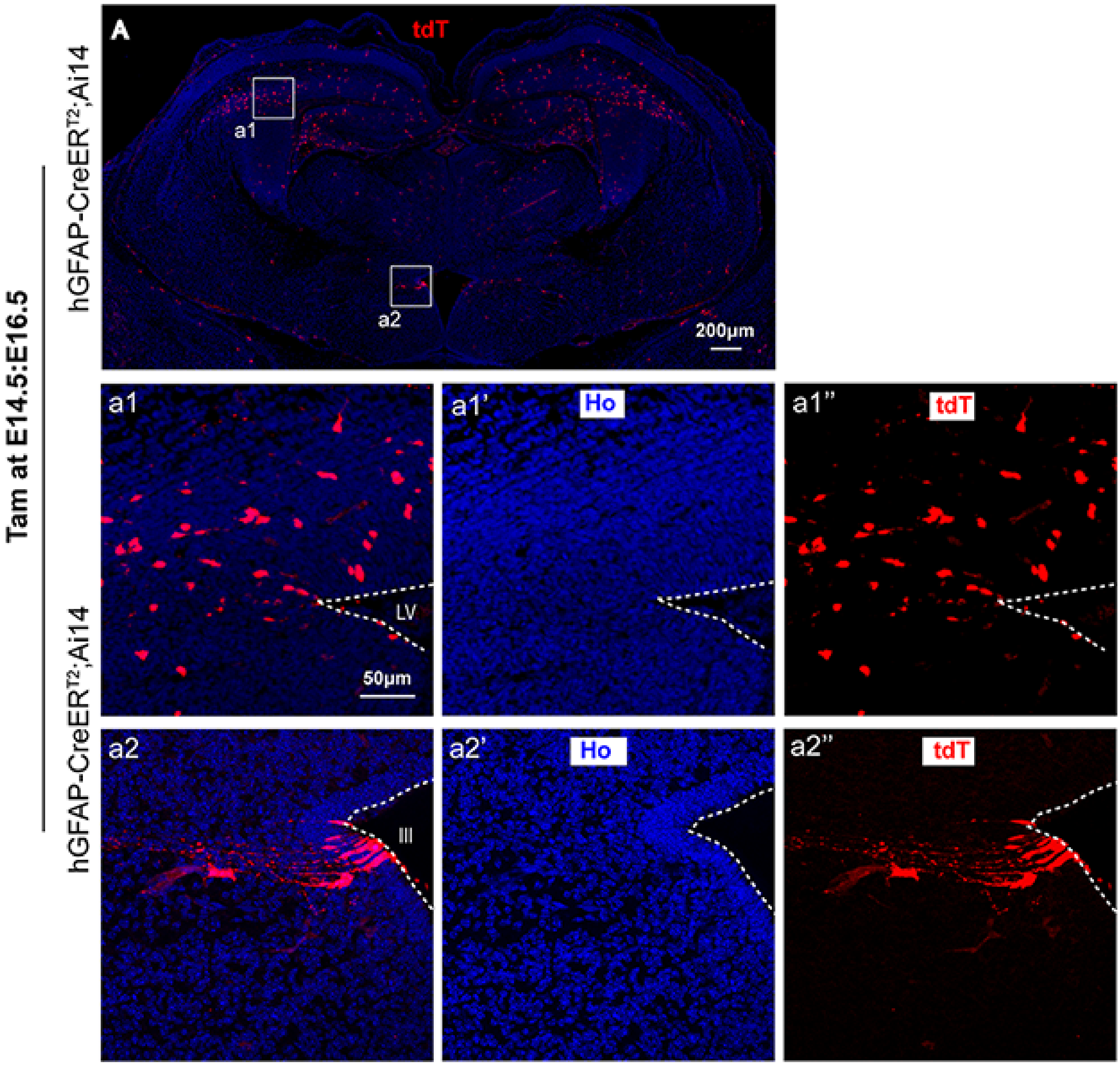

Fig. S5 Genetic labeling of the early progenitors of *hGFAP-CreER^{T2};Ai14* transgenic mice. **A** E16.5 brain section showing the tdT-expressing cells after tamoxifen administration beginning at E14.5. Higher-magnification views as indicated show the tdT^{+} cells in the dorsal wall of the 3V (**a1**) and the dorsal VZ/SVZ of the LV (**a2**). Ho, Hoechst staining.

Table S1. List of genes that were up-regulated in the dorsal wall compared with the ventral wall of the 3V.

Table S2. List of genes that were down-regulated in the dorsal wall compared with the ventral wall of the 3V.

Table S3. List of genes that were up-regulated in the dorsal wall of the 3V compared with those in the dorsal wall of the LV.

Table S4. List of genes that were down-regulated in the dorsal wall of the 3V compared with those in the dorsal wall of the LV.

Table S5. A custom geneset that represents positive regulation of astrogenesis and astrocyte development.
